# An Enantioselective Alkene Aminoarylation to Form Chiral Indolines via Electrostatically‐Directed Palladium Catalysis

**DOI:** 10.1002/anie.8602143

**Published:** 2026-06-22

**Authors:** Max Kadarauch, Paul T. Jirsch, H. Erik Diepers, Hamzah Sharif, Robert J. Phipps

**Affiliations:** ^1^ Yusuf Hamied Department of Chemistry University of Cambridge Cambridge UK

**Keywords:** chiral phosphine, heterocycles, indolines, palladium

## Abstract

Most design strategies in asymmetric transition metal catalysis invoke repulsive interactions between ligand and substrate. Yet those that incorporate attractive interactions can offer advantages, such as rate acceleration and greater generality, due to the fundamentally different mode of operation. Here we deploy electrostatically‐directed Pd catalysis using the chiral sulfonated ligand sSPhos to realize an asymmetric aminoarylation of *ortho*‐allyl anilines under mild conditions. A wide variety of substituted aryl bromides and anilines provide access to 2‐benzylindolines with high enantioselectivity. Our study uncovers trends relating the electronics of both coupling partners to *ee*. These trends allowed tuning of the sulfonamide protecting group to enable good results across a broader range of substrates.

## Graphical abstract

Enantioselective aminoarylation of *ortho*‐allyl anilines is achieved under mild conditions. Key to success is the electrostatically‐directed nature of the key aminopalladation step, achieved by using the sulfonated chiral ligand sSPhos on palladium. A detailed study of electronic trends allows tuning of the sulfonamide protecting group to enable good results across a broad range of substrates.

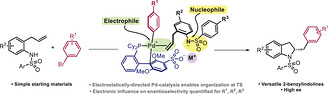

## 1 Introduction

**FIGURE 1:**
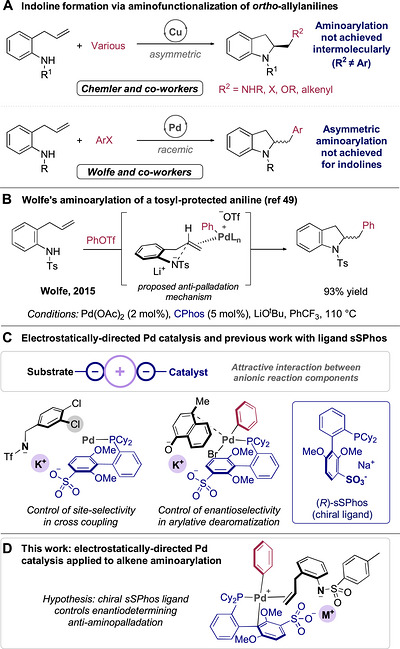
Background on the cyclization of *ortho*‐allylanilines to form indolines, and this work.

Indolines, consisting of a benzene ring fused to a pyrrolidine, are an important heterocyclic motif. Alternatively regarded as 2,3‐hydrogenated indoles, stereochemistry can feature on the aliphatic portion of the pyrrolidine ring. Indolines occur extensively in natural products, originating biosynthetically from indoles and ultimately from tryptophan [1]. Various members of the broad family of so‐called “indole alkaloids” actually contain indolines as structural elements, rather than indoles; the family name reflects their biosynthetic origin [2]. The indoline motif is prevalent in both natural product‐derived therapeutics and de novo designed drugs [3, 4, 5]. For this reason, many methods to access these structures have been developed, with particular attention to control of stereochemistry when substituents are present at the 2‐ and/or 3‐ positions [6]. Although asymmetric reduction of indoles constitutes a major route toward enantioenriched indolines [7, 8, 9, 10, 11, 12, 13, 14, 15, 16, 17], a more retrosynthetically simplifying approach involves control of stereochemistry during formation of the indoline scaffold from an aniline derivative in an annulation process [18, 19, 20, 21, 22, 23, 24, 25]. Selected recent asymmetric examples include Fischer indolization [26], 5‐*endo*‐trig cyclization onto imines [27], and use of engineered enzymes for C─H amination [28]. Cyclizations of 2‐allylaniline derivatives are particularly useful disconnections: The starting materials can typically be accessed through *aza*‐Claisen rearrangement while aminofunctionalization of the alkene permits significant complexity to be generated in a single step. Chemler and coworkers have carried out extensive research on copper‐catalyzed asymmetric aminofunctionalization reactions for the formation of indolines (Figure 1A, upper) [29]. Using a radical‐based mechanism they have achieved aminooxygenation [30, 31], aminohalogenation [32], aminoalkenylation [33], and diamination [34] processes (Figure 1A, upper). Notably, aminoarylation lies outside the scope of this process: This was only accomplished in a fully intramolecular system, in which the new aryl component is tethered to the 2‐allylaniline derivative, with moderate enantioselectivity [35, 36, 37]. Palladium catalysis using aryl halide electrophiles might offer a more amenable approach to intermolecular aminoarylation [38, 39, 40]. Since their first report in 2004, Wolfe and coworkers have pioneered such Pd‐catalyzed aminoarylation/cyclization reactions [41, 42, 43, 44, 45, 46, 47, 48, 49, 50], which include several reports that can form indolines in high yield (Figure 1A, lower) [41, 43, 45, 49, 50]. Although they have reported an enantioselective aminoarylation to form pyrrolidines [44], none of the indoline‐forming reactions have been rendered enantioselective. This longstanding problem provides a clear goal for those developing new chiral ligands for enantioselective Pd‐catalysis.

Detailed mechanistic studies on the Pd‐catalyzed process from Wolfe and coworkers suggest that an *anti*‐aminopalladation pathway operates when the nitrogen atom possesses an electron‐withdrawing protecting group (e.g., tosyl) [51]. Here, the low nucleophilicity of the nitrogen atom means that only the alkene is bound to the phosphine‐ligated aryl Pd(II), while the deprotonated amine attacks the Pd‐complexed alkene in an *anti*‐manner (Figure 1B) [47, 49]. In this intriguing mechanistic hypothesis, we saw an opportunity to leverage attractive non‐covalent interactions in the outer ligand sphere to address the unsolved challenge of enantioselective aminoarylation to form indolines [52]. We have recently developed the concept of electrostatically‐directed palladium catalysis using sSPhos, an anionic, sulfonated, and chiral variant of the common ligand SPhos. Our approach has involved identifying suitable anionic groups on reactive intermediates that can engage in selectivity‐controlling ionic interactions with the sulfonate group of sSPhos, via a bridging alkali metal cation (Figure 1C, upper). This strategy has permitted control of site‐selectivity in cross‐coupling using racemic sSPhos [53, 54, 55] and control of enantioselectivity in arylative dearomatization of phenols and naphthols using enantiopure sSPhos (Figure 1C, lower) [56, 57, 58]. As well as controlling selectivity, attractive substrate‐ligand interactions can lower the barriers for these processes, providing rate acceleration [57]. Within Wolfe's mechanistic hypothesis, we envisaged that during the transition state for *anti*‐aminopalladation, an interaction between the deprotonated aniline, a metal cation, and the sulfonate group of sSPhos might provide an ordered chiral environment capable of enantiocontrol in the formation of the resulting stereocenter (Figure 1D). We can reveal that this enables a highly enantioselective method for the challenging preparation of 2‐benzyl indolines with broad scope.

## 2 Results and Discussion

We began our studies with conditions very similar to Wolfe's 2015 report, replacing CPhos with (*R*)‐sSPhos (Table 1) [49]. Although promising initial enantioselectivities were obtained for both aryl triflate and bromide, yields were low (entries 1 and 2). We elected to continue optimization on an aryl bromide and found that yield improved along the series LiOH, NaOH, KOH (entries 2–4), although enantioselectivity decreased. A weaker base (K_2_CO_3_) afforded a lower 11% *ee* (entry 5). We continued using NaOH as a compromise between yield and *ee*. The addition of water as a co‐solvent has been shown to promote the formation of the active Pd(0) catalyst from Pd(OAc)_2_ [59], as well as improve enantioselectivity in related transformations [60, 61], including our previous work [56]. We found yield and enantioselectivity were greatly improved using a 5:1 ratio of trifluorotoluene to water in a biphasic system (compare entries 3 and 6). The excellent reactivity under these conditions permitted a reduction of the temperature, with significant and successive *ee* increases obtained at 65 °C and 35° C (entries 7 and 8). Excellent reactivity was still observed at room temperature, although 35 °C was judged preferable to take forward to scope exploration (entry 9). Replacing trifluorotoluene with 1,2‐dichloroethane was beneficial, allowing 90% *ee* to be obtained (entry 10). Increasing concentration to 0.4 M improved the yield, constituting the final optimized conditions (entry 11). The outcome was largely unaffected by the leaving group X with both the triflate and iodide giving 92% *ee* (entries 12 and 13). This is consistent with the (pseudo)halide ligand being dissociated from a cationic Pd complex ahead of the anticipated enantiodetermining anti‐aminopalladation of the bound alkene (Figure 1D). A re‐evaluation of LiOH did not impact the metrics under these final optimized conditions (entry 14). Inverting the coupling partner stoichiometries afforded lower yield, but similar *ee* (entry 15 vs 11) [62]. A crystal structure of **3a** enabled the absolute configuration to be determined. A brief evaluation of other *N*‐protecting groups was carried out under conditions close to the final optimized ones, but poor results were obtained (see the Supporting Information for details) [63]. The *N*‐tosyl group can be readily removed without loss of *ee* (see Supporting Information).

**TABLE 1:** Reaction optimization.

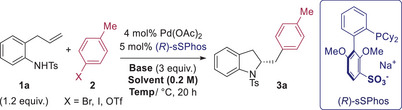						
Entry	X	Base	Solvent^a^	T	Yield^b^ %	*ee* ^c^ %
1	OTf	LiOH	PhCF_3_	100	20	56
2	Br	LiOH	PhCF_3_	100	11	50
3	Br	NaOH	PhCF_3_	100	44	43
4	Br	KOH	PhCF_3_	100	67	20
5	Br	K_2_CO_3_	PhCF_3_	100	79	11
6	Br	NaOH	PhCF_3_:H_2_O (5:1)	100	97	68
7	Br	NaOH	PhCF_3_:H_2_O	65	90	76
8	Br	NaOH	PhCF_3_:H_2_O	35	88	85
9	Br	NaOH	PhCF_3_:H_2_O	RT	89	85
10	Br	NaOH	DCE:H_2_O	35	87	90
11^d^	Br	NaOH	DCE:H_2_O	35	94 (95)	91 (91)
12^d^	OTf	NaOH	DCE:H_2_O	35	90	92
13^d^	I	NaOH	DCE:H_2_O	35	71	92
14^d^	Br	LiOH	DCE:H_2_O	35	91	91
15^d^, ^e^	Br	NaOH	DCE:H_2_O	35	81	90

^a^ In all cases where a mixed solvent system is indicated, the ratio was 5:1 in the order shown.

^b^ Yields determined by ^1^H NMR spectroscopy with reference to internal standard. Value in parenthesis refers to isolated yield.

^c^ *ee* determined by chiral SFC analysis of the crude reaction mixture.

^d^ 0.4 M concentration.

^e^ 1 equiv. alkene **1a** and 1.2 equiv. Ar‐Br **2a**.

**SCHEME 1:**
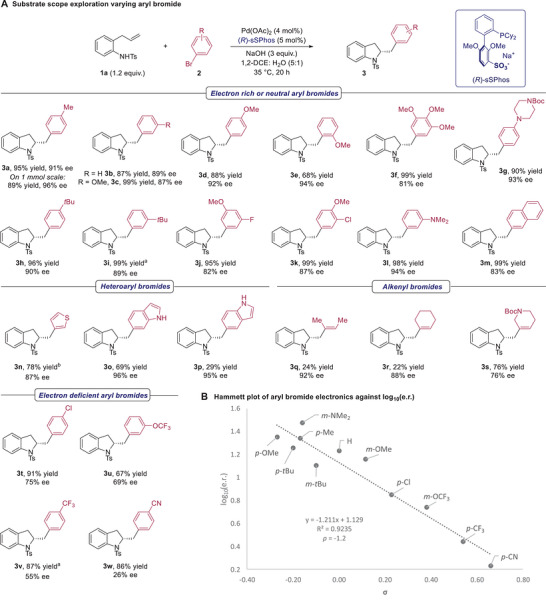
Scope of enantioselective aminoarylation of aniline **1a** using aryl and alkenyl bromides and Hammett plot. Yield and *ee* values correspond to isolated material. (a) Reaction conducted with 2 equiv. **1a**. (b) Reaction conducted with **1a** as the limiting reagent and 1.2 equiv. 3‐bromothiophene.

The scope of the reaction with respect to the aryl bromide was evaluated (Scheme 1A). For the optimization substrate **3a**, performing the reaction on a larger 1 mmol scale improved the *ee* from 91% to 96%, which could be related to mass‐transfer effects in the biphasic system. An unsubstituted phenyl also gave an excellent outcome (**3b**). Methoxy substituents were well tolerated at all positions of the arene (**3c**‐**3e**) and a trimethoxy variant gave only a small reduction in *ee* (**3f**). An aryl bromide incorporating a protected piperazine performed very well (**3g**). *Tert*‐butyl groups could be incorporated at two positions (**3h**, **3i**), aryl bromides containing two substituents could be used (**3j**, **3k**), and dimethylamino‐ (**3l**) and naphthyl‐containing examples worked well (**3m**). Excellent enantioselectivities were also obtained for thiophenyl and indolyl bromides, the latter notably not requiring *N*‐protection (**3n**‐**3p**). We were pleased to observe that three alkenyl bromides that were tested gave high *ee* values, although the yields were generally lower than for the aryl bromides (**3q**‐**3s**). Aryl bromides with a phenolic hydroxyl or an acetamide did not give product formation; it seems likely that their deprotonation under the reaction conditions could be problematic (see Supporting Information for unsuccessful substrates). The aryl bromides presented so far have all been electron‐neutral or electron‐rich. Those containing electron‐withdrawing substituents, although delivering good yields, gave markedly lower enantioselectivities (**3t**‐**3w**). The overall trend prompted us to plot log_10_(e.r.) against the Hammett parameter for the *meta* and *para*‐monosubstituted aryl bromides in the scope. A well‐defined correlation was observed, featuring a negative *ρ* value (Scheme 1B). Whilst the reduced *ee* outcomes for these electron deficient aryl halides initially proved disappointing, the tunability of the sulfonamide protecting group has potential to address this (*vide infra*).

**SCHEME 2:**
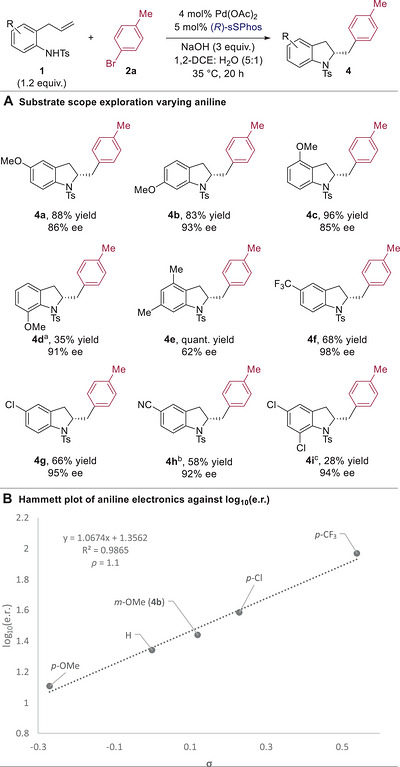
Scope of enantioselective aminoarylation using substituted allylanilines with aryl bromide **2a** and Hammett plot. Yield and *ee* values correspond to isolated material. (a) KOH used instead of NaOH. (b) KOH used at 50° C. (c) KOH used at 80° C.

We next examined the scope of the aniline component and investigated several substituted *ortho*‐allyl, *N*‐tosylanilines in combination with 4‐bromotoluene (Scheme 2). Electron‐rich anilines bearing methoxy groups in all four possible positions reacted with good *ee* (**4a**‐**4d)**. A dimethylsubstitued aniline gave high yield, but lower enantioselectivity (**4e**). Electron withdrawing groups including trifluoromethyl (**4f**) and chloro (**4g**) worked very well. In these cases, while yields were slightly reduced, *ee* values were noticeably increased. A cyano‐substituted substrate proved insoluble under the normal reaction conditions, but switching the base to KOH and a higher reaction temperature allowed 58% yield and 92% *ee* to be obtained for indoline **4h**. Finally, a dichlorinated example gave low yield, but excellent *ee* (**4i**). Formation of a tetrahydroquinoline by incorporating an extra methylene into the starting material was unsuccessful, giving <10% yield of product, nor was the use of internal alkenes (see Supporting Information for details). Given the previously‐observed correlation of *ee* with the electronics of the aryl bromide, we generated a Hammett plot using available data corresponding to the substituent positioned with respect to the aniline amine component [64]. Again, an excellent fit was obtained, this time with a positive *ρ* value (Scheme 2B).

**SCHEME 3:**
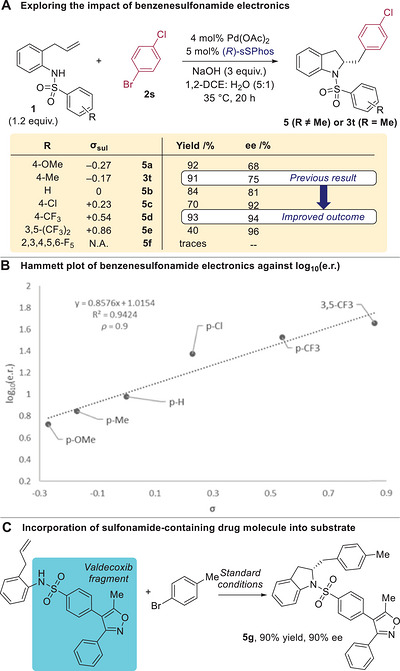
Varying the electronics of the sulfonamide protecting group and Hammett plot, with consequent improvements to enantioselectivity.

The clear correlation between electronics and *ee* observed for two of the key aromatic components of the reaction was striking, prompting us to investigate the electronics of the third—the arenesulfonamide protecting group on the aniline nitrogen. The protecting group is a disposable component of the target indoline that would likely be removed in subsequent steps: A solution to poorly performing substrate combinations based on tuning the protecting group would therefore be practical. We carried out a study using a 4‐chlorobromobenzene coupling partner, as this had given only 75% *ee* with tosyl as the protecting group. A range of protecting group substituents were evaluated, with a clear trend observed: As the sulfonamide aromatic ring became more electron‐deficient, *ee* increased (Scheme 3A). Of the mono and di‐substituted sulfonamides evaluated, the yield for the most electron deficient (3,5‐bistrifluoromethyl) was significantly reduced compared with the others, at 40%, but it gave the highest *ee* at 96%. A highly withdrawing pentafluoro‐substituted analogue afforded only traces of product in the crude ^1^H NMR spectrum. These results suggest that past a certain threshold of protecting group electron‐deficiency, aminoarylation yield declines, likely caused by the diminished nucleophilicity of the deprotonated aniline nitrogen (see later discussion). A Hammett plot of these results again provided an excellent correlation, with a positive *ρ* value, as for the aniline component (Scheme 3B) [65]. We also demonstrate the incorporation of the sulfonamide‐containing pharmaceutical Valdecoxib into the substrate as the *N*‐sulfonyl group; the reaction performs extremely well to give indoline **5g** (Scheme 3C).

**SCHEME 4:**
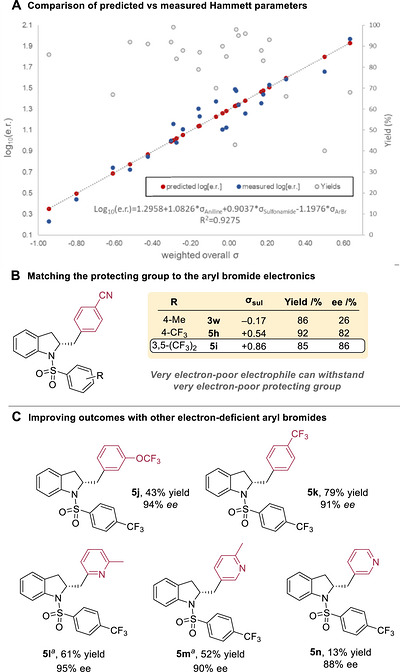
Linear regression and development of a simple model to match the protecting group to the aryl bromide substrate. (a) KOH used instead of NaOH.

Given the excellent Hammett correlations observed, we sought a relationship between the overall electronics of the substrates and *ee*. We used linear regression to obtain an equation for log_10_(e.r.) as a function of the three Hammett parameters (equation depicted in Scheme 4A). This can be visualized in a plot of log_10_(e.r.) (left‐hand y axis) against the weighted overall Hammett (consisting of all three Hammett parameters) which displays an R^2^ value of 0.93 (Scheme 4A). It was also instructive to plot observed yield for each entry (right‐hand y axis). For examples up to log_10_(e.r.) of approximately 1.5, equating to 94% *ee*, yields were generally high. For 1.5< log_10_(e.r.) < 2.0 the yields were noticeably reduced for the three examples examined under the standard reaction conditions (**4f**, **4g**, **5e**). This trend suggested that matching of the protecting group with the substituents on the aniline and aryl bromide may permit the highest *ee* for a given combination to be obtained, up to the point where reactivity is adversely impacted. We next evaluated the *para*‐cyano bromobenzene, which had given low enantioselectivity when paired with the *N*‐tosyl aniline (Scheme 4B, entry 1). Gratifyingly, moving to trifluorotosyl increased this to 82% *ee* in **5h**, still with excellent yield (entry 2). The observed trends suggest that this aryl bromide might tolerate an even more deficient protecting group while maintaining good yield. Indeed, evaluation of a 3,5‐bistrifluoromethyl‐substituted protecting group in **5i** increased *ee* to 86%, without significantly impacting yield in this case. These results suggest that in cases of extreme electronics on the aryl bromide, matching the protecting group can give the best balance between yield and enantioselectivity, for which a trade‐off is apparent when the highest *ee* values are approached. Nevertheless, we anticipated that most moderately electron‐deficient aryl bromides would give good outcomes using a trifluorotosyl‐substituted protecting group. We evaluated the remaining two electron‐deficient aryl bromides that had previously given reduced *ee* using the tosyl projecting group. *Meta*‐trifluoromethoxy (**5j**) and *para‐*trifluoromethyl (**5k**), which had previously given moderate enantioselectivities using a tosyl protecting group (see earlier, Scheme 1), now gave excellent enantioselectivities (Scheme 4C). Enantioselectivities were also high for three electron‐deficient pyridine‐containing heteroaryl bromides (**5l**‐**5n**). We speculate that the low yield of indoline **5n** could arise from deleterious binding of the unhindered pyridine nitrogen to palladium, which would be reduced in **5l** and **5m**.

**SCHEME 5:**
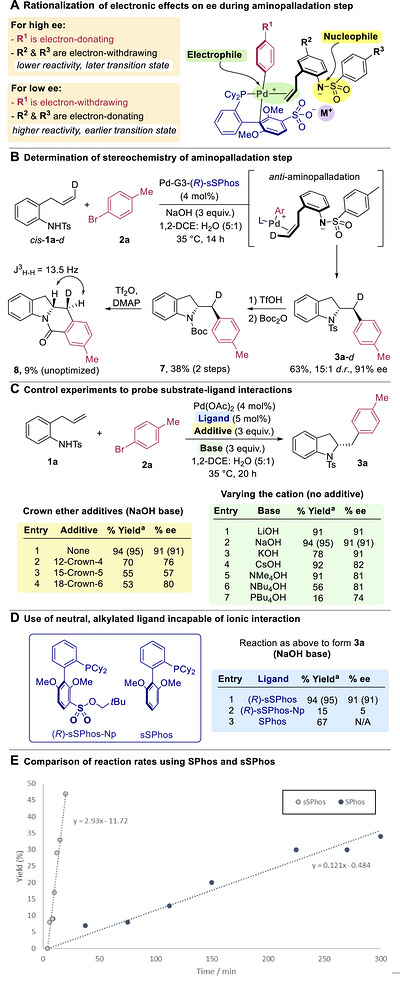
Working hypothesis to explain effect of substrate electronics on *ee*, and mechanistic experiments. (a) Yields determined by ^1^H NMR spectroscopy with reference to internal standard. Values in parentheses refer to isolated yield.

Clear trends have been observed between the electronic character of the three aryl components and enantioselectivity. These observations are consistent with an enantiodetermining aminopalladation, since the electronic character of all three aromatic rings should have an influence during this step. The aryl bromide‐derived arene is ligated to Pd(II) during the aminopalladation; electron‐donating substituents on this component (R^1^) should make the Pd(II) less electrophilic and therefore less reactive toward alkene aminopalladation (Scheme 5A, green oval). Both aromatic rings on the alkene substrate (aniline and sulfonamide) influence the delocalization of negative charge on the anionic nitrogen atom: Electron withdrawing groups (R^2^ and R^3^) should make the nitrogen less nucleophilic and therefore less reactive during the aminopalladation (yellow oval). Our results demonstrate that this combination of electronics results in the highest *ee* outcomes. In contrast, an electron deficient aryl bromide (rendering the Pd center more electrophilic), combined with electron donating groups on the two aromatic rings of the substrate (making the nitrogen more nucleophilic) should accelerate the aminopalladation, appearing to lead to lower *ee*. To rationalize the origin of this effect, we were inspired by mechanistic work of Jacobsen and coworkers on Mn‐catalyzed alkene epoxidation [66]. There, catalysts bearing electron‐donating substituents at the para position of the salen ligand backbone gave the highest *ee*, with a negative correlation between log(e.r.) and the Hammett parameters of these substituents. It was proposed that Mn‐salen complexes bearing electron‐donating substituents would be more stable. According to the Hammond postulate, this should result in a later transition state for oxygen transfer than if the complex were more reactive. Conversely, a more reactive catalyst proceeds via an earlier transition state, in which the opposite is expected. In our case, electronic features on both substrates which slow down the aminopalladation lead to higher *ee*, as demonstrated through the three Hammett correlations. A difference between our system and Jacobsen's is that in the Mn‐catalyzed epoxidation there is no pre‐coordination between catalyst and substrate. A clear rationale therefore exists for why a later transition state should lead to higher *ee*: The chiral catalyst is closer to the substrate and therefore feels more influence from its structural features. In our case, we propose that pre‐coordination of the alkene preceeds aminopalladation, so the rationale for why a later transition state should give higher *ee* is less clear. But the effects of substrate electronics on *ee* are nevertheless consistent with a transition state position argument.

We next sought evidence that the reaction was proceeding via the proposed *anti*‐aminopalladation pathway. We aimed to test a substrate with a single deuterium selectively incorporated into the alkene: An analogous experiment to those carried out by Wolfe and coworkers. Determining which diastereomer of the product is formed permits either a *syn* or *anti* mechanism to be invoked (Scheme 5B) [44, 47, 49, 67, 68]. During initial investigations using substrate *cis*‐**1a** *‐d*, we observed unexpected isomerization to *trans*‐**1a** *‐d* under the reaction conditions, which complicated interpretation. We discovered that use of an (*R*)‐sSPhos‐G3‐precatalyst reduced this isomerization and allowed product **3a** *‐d* to be isolated with 15:1 d.r. and high *ee* [69]. To determine the relative stereochemistry the tosyl group was first exchanged for Boc (**3a** *‐d* to **7**). Treatment of **7** with Tf_2_O and DMAP formed amide **8** whose relative stereochemistry could be determined through NMR experiments [44]. These confirmed that the amine and aryl substituents had added across the alkene in an *anti*‐fashion, consistent with the proposed *anti*‐aminopalladation mechanism. We also performed the analogous reaction using *p*‐cyanobromobenzene, that had given low *ee* in the initial scope (Scheme 1, **3w**), to test whether this low *ee* could be attributed to *syn* and *anti*‐palladation occurring in competition. However, the d.r. outcome was very similar to that obtained with 4‐bromotoluene (**2a**), suggesting that this was not the case (see Supporting Information for details).

We finally carried out experiments to probe the proposed substrate‐ligand interaction, and particularly the role of the alkali metal cation. ^1^H NMR experiments suggest that the substrate undergoes full deprotonation by NaOH under the reaction conditions (see Supporting Information for details). The addition of stoichiometric crown ethers of various sizes led to a reduction in enantioselectivity for all crown ethers tested, suggesting that the substrate‐ligand interaction is disrupted to some extent by binding of the alkali metal cation (Scheme 5C, left). Next, the hydroxide base was varied to probe the size effect of the associated cation (Scheme 5C, right). Enantioselectivity was unchanged for LiOH, NaOH, and KOH (91% *ee*, entries 1–3), suggesting a good fit in the enantiodetermining transition state for these cations. The larger metal cation Cs^+^ and the organic cations ^+^NMe_4_, and ^+^NBu_4_ incurred a drop in *ee*, with ^+^PBu_4_ resulting in a further drop in *ee* to 74%. These observations suggest that when the cation gets too large the preferred transition state is disrupted. We also evaluated a neopentyl sulfonate ester derivative of sSPhos ((*R*)‐sSPhos‐Np), which is incapable of engaging in the proposed electrostatic interactions (Scheme 5D). Enantioselectivity was reduced to 5% *ee* with a significant reduction in yield (entry 2 vs entry 1). Achiral SPhos gave a yield between the two, suggesting that the reaction is promoted by the attractive interactions possible with sSPhos, and inhibited by the steric influence of sSPhos‐Np (entry 3). The acceleration provided by the ligand sulfonate group is apparent when the rate of reaction using sSPhos is compared with SPhos, providing an approximate 24‐fold rate increase (Scheme 5E) [70]. The rate acceleration provided by sSPhos likely contributes to the high yields across a broad range of substrates under mild conditions.

## 3 Conclusions

In summary, we have applied electrostatically‐directed palladium catalysis using chiral ligand sSPhos to an enantioselective aminoarylation of *ortho*‐allyl anilines with aryl bromide electrophiles under mild conditions. This reaction forms 2‐benzylindolines with very high enantioselectivity and we have demonstrated a broad scope with respect to both components. Insight gained from three distinct electronic trends allowed us to improve upon initially low *ee* for electron‐deficient aryl bromides. The resulting hypothesis that electronic tuning of the *N*‐protecting group may solve the problem was supported by employing the trifluorotosyl group, which delivered high *ee* values for these substrates. The electronic trends were consistent with the proposed enantiodetermining *anti*‐aminopalladation; incorporating substituents which slow down this step resulted in higher *ee*, possibly due to a later, more product‐like transition state. Linear regression was used to derive a weighted overall Hammett equation, with which a user can judge the feasibility of a particular combination and the likely *ee* outcome. Experiments provide support for an *anti*‐palladation mechanism and demonstrate the importance of the attractive interactions between ligand and substrate. This study provides an enantioselective approach to useful chiral indoline building blocks that were previously inaccessible, whilst at the same time demonstrating the power of design principles based upon attractive electrostatic interactions [71].

## Author Contributions

**Max Kadarauch**: conceptualization, methodology, investigation, writing – original draft, writing – review and editing, and formal analysis. **Paul T. Jirsch**: methodology, investigation, and writing – review and editing. **H. Erik Diepers**: methodology and investigation. **Hamzah Sharif**: methodology and investigation. **Robert J. Phipps**: writing – review and editing, project administration, writing – original draft, supervision, and funding acquisition.

## Conflicts of Interest

The authors declare no conflicts of interest.

## Supporting information




**Supporting File 1**: Further reaction optimization, procedures, characterization data are provided in the associated PDF file. CDC 2532667 contains the supplementary crystallographic data for this paper. This can be obtained free of charge via www.ccdc.cam.ac.uk/data_request/cif or by contacting the Cambridge Crystallographic Data Centre, 12 union road, Cambridge, CB2 1EW.

**Supporting File 2**: anie73125‐sup‐0002‐DataFile.cif.

## Data Availability

The data that supports the findings of this study are available in the supplementary material of this article.
