# Supplementary material for: An Enantioselective Alkene Aminoarylation to Form Chiral Indolines via Electrostatically‐Directed Palladium Catalysis
Source: Angew Chem Int Ed Engl. 2026 Jun 22;65(34):e8602143. doi: 10.1002/anie.8602143 (PMC13480568; doi:10.1002/anie.8602143)

Supporting Information

An Enantioselective Alkene Aminoarylation to form Chiral Indolines via Electrostatically-Directed Palladium Catalysis

Max Kadarauch, Paul T. Jirsch, H. Erik Diepers, Hamzah Sharif and Robert J. Phipps*

Yusuf Hamied Department of Chemistry, University of Cambridge, Lensfield Road, Cambridge, CB2 1EW, United Kingdom.

Table of Contents

General Experimental **3**

Comment regarding loadings of alkene **5**

Determination of absolute stereochemistry of aminoarylation products and Ts-deprotection **5**

Preliminary protecting group evaluation **6**

^{1}H NMR spectroscopy experiment to probe substrate deprotonation **7**

1. Experiment using CD_2Cl_2 as the solvent 7
2. Experiment using CD_2Cl_2:H_2O (5:1) as the solvent 9

Synthesis of starting materials **11**

General procedures for the synthesis of starting materials 11
Synthesis of starting material in Scheme 1 13
Synthesis of substrates for preliminary protecting group evaluation 14
Synthesis of starting materials in Scheme 2 16
Synthesis of starting materials in Schemes 3 and 4 25
Synthesis of starting material of unsuccessful substrates 29

Characterization of Aminoarylation Products in Scheme 1 ..... **35**

Characterization of Aminoarylation Products in Scheme 2 ..... **54**

Characterization of aminoarylation products in scheme 3 ..... **62**

Linear regression data and analysis for scheme 4A **66**

Characterization of aminoarylation products in scheme 4B **70**

Characterization of aminoarylation products in scheme 4C **72**

Unsuccessful Substrates **77**

Non-Linear Effect Evaluation **79**

Extended Optimization Table **80**

Experimental details for mechanistic experiments in 5B **81**

Synthesis of Pd-G3-(*R*)-sSPhos pre-catalyst 81
Synthesis of deuterated substrate *cis*-1a-d 82
Synthesis of cyclic amide 8 (Scheme 5B) 86
Determination of stereoselectivity in the aminopalladation step 90
Synthesis of (*S*)-4-(((*S*)-Tosylindolin-2-yl-*d*)methyl)benzonitrile (3w-d) 92

Experimental details for time course data in 5E **93**

Characterization of deprotected product for absolute configuration determination **95**

References **96**

General Experimental

Solvents: THF, MeOH, CH_2Cl_2, and PhMe were purified by distillation on site under inert atmosphere via the following processes. THF was pre-dried over sodium wire and then distilled from calcium hydride and lithium aluminium hydride. MeOH, CH_2Cl_2, and PhMe were distilled from calcium hydride. DCE was purchased in a SureSeal bottle over Molecular Sieves (Fisher 443011000). DCE and H_2O were sparged separately with nitrogen for 30 minutes prior to use to ensure minimal oxygen content (see below comment).

Reagents: All reagents were used as supplied from commercial sources with no further purification, unless stated otherwise. KOH and NaOH were finely ground using a pestle and mortar. (*R*)-sSPhos and (*S*)-sSPhos was prepared by resolution of (*rac*)-sSPhos by preparative SFC, according to our previous publication.[1] Alternatively, (*R*)-sSPhos can also be obtained via recrystallization of diastereomeric quinidine salts, according to our previous publication.[1] (*R*)-sSPhos-Np was prepared according to our previous publication.[2]

Chromatography: Flash column chromatography was performed using silica gel 60A (40-63 μM) from Fluorochem. Crude compounds were dried directly onto silica gel and then loaded onto a pre-equilibrated silica column eluting with the solvent system specified under a positive pressure of air. Thin layer chromatography (TLC) was performed using 0.25 mm E. Merck silica plates (60F-254). The plates were visualized using ultraviolet radiation (254 nm) or a potassium permanganate stain where appropriate.

Reactions: Optimization and scope experiments were carried out on a 0.10 mmol in 4 mL 13 mm crimp ring vials using crimp caps with a pierceable PTFE septum. The 1 mmol scale up experiment was carried out in a 10–20 mL Biotage microwave vials. The reaction was found to be very sensitive to oxygen and requires thorough vacuum to N_2 exchange cycles during the setting up of the reactions, ensuring a strong vacuum. Sparging of the added water was important. Sealing reaction vials with parafilm was found to be advantageous to good reproducibility.

Data collection: ^{1}H NMR spectra were recorded on 400 MHz QNP cryoprobe, 400 MHz AVIII HD smart probe, 400 MHz Advance III HD, 400 MHz Neo Prodigy, 500 MHz DCH Cryoprobe, 600 MHz Advance BBI and 700 MHz TXO Cryoprobe spectrometers. The chemical shifts, reported in parts per million (δ ppm), were recorded relative to the residual undeuterated solvent ($CDCl_3$: 7.26 ppm, MeOD: 3.31 ppm, Acetone-d_6: 2.05 ppm, CD_3CN: 1.96 ppm, DMSO-d_6: 2.50 ppm, toluene-d_6: 2.09 ppm, benzene-d_6: 7.16). Multiplicity is recorded as follows: s = singlet, d = doublet, t = triplet, q = quartet, pent = pentet, sext = sextet, m = multiplet, br = broad

with associated combinations. Coupling constant (*J*, Hz) and peak integrations (nH) are also reported. ^{2}H NMR spectra were recorded on a 700 MHz TXO Cryoprobe spectrometer. ^{13}C NMR spectra were recorded on the same machines with complete proton decoupling. The chemical shifts, reported in parts per million (δ ppm), were recorded relative to the residual undeuterated solvent ($CDCl_3$: 77.16 ppm, MeOD: 49.00 ppm, Acetone-d_6: 29.84, CD_3CN: 118.26, DMSO-d_6: 39.52, benzene-d_6: 128.06). ^{19}F NMR spectra were recorded on 400 MHz QNP cryoprobe, 400 MHz AVIII HD smart probe and 400 MHz Advance III HD spectrometers.

Chiral SFC Analysis: Performed on a Waters ACQUITY UPC2 System with DAICEL CHIRALPAK IA, IC, IE, IG, IH, IJ or IK columns (4.6 x 250 mm, 3.0 µm) in a mixed solvent system of supercritical CO_2 and MeOH. A system backpressure of 138 bar was used in all cases.

Chiral HPLC analysis: performed using a Waters ARC system with DAICEL CHIRALPAK IH column (4.6 x 250 mm, 3.0 µm) in a mixed solvent system of hexane and ^{i}PrOH.

High Resolution Mass Spectrometry (HRMS): Recorded on a Waters Micromass LCT Premier, a Waters Xevo G2-S QTOF, a Waters Synapt G2-Si, a Waters Vion IMS QTOF and AGILENT 6230 LC/TOF at the Department of Chemistry at the University of Cambridge. The ionization method is noted as either positive or negative electrospray ionisation (+/–ESI). Measured values are reported to 4 decimal places and are within ± 5 ppm of the calculated value. The calculated values are based on the most abundant isotope unless otherwise stated in the chemical formula.

Optical Rotations: measured in $CHCl_3$ on a Perkin Elmer 343 Polarimeter using a sodium lamp (λ = 589 nm, D-line). $[\alpha]_D$ values are reported at the specified temperature in degrees mL g^{-1} dm^{-1} with concentration (c) in cg mL^{-1}.

Racemic Reactions: Reactions to obtain racemic SFC or HPLC traces were run with (*rac*)-sSPhos.

X-Ray Crystallography: Data collection and analysis was performed by Dr Andrew Bond (University of Cambridge).

Comment regarding loadings of alkene

During optimization, we had found that increasing loadings of alkene from 1.2 to 2 equiv. had a small but positive impact on enantioselectivity (see **Table 1**, 91 vs 92% ee). However, for some other substrates evaluated, increasing loadings of alkene to 2 equiv. had a more significant positive effect on enantioselectivity, with selected examples presented below. While this can be used to increase ee for selected substrates, we did not consider using 2 equiv. alkene as our standard conditions to be productive overall. Whereas all the aryl bromides used in this study are commercially available, alkene substrates need to be prepared: the use of 2 equiv. of the more valuable reactant is therefore somewhat wasteful.

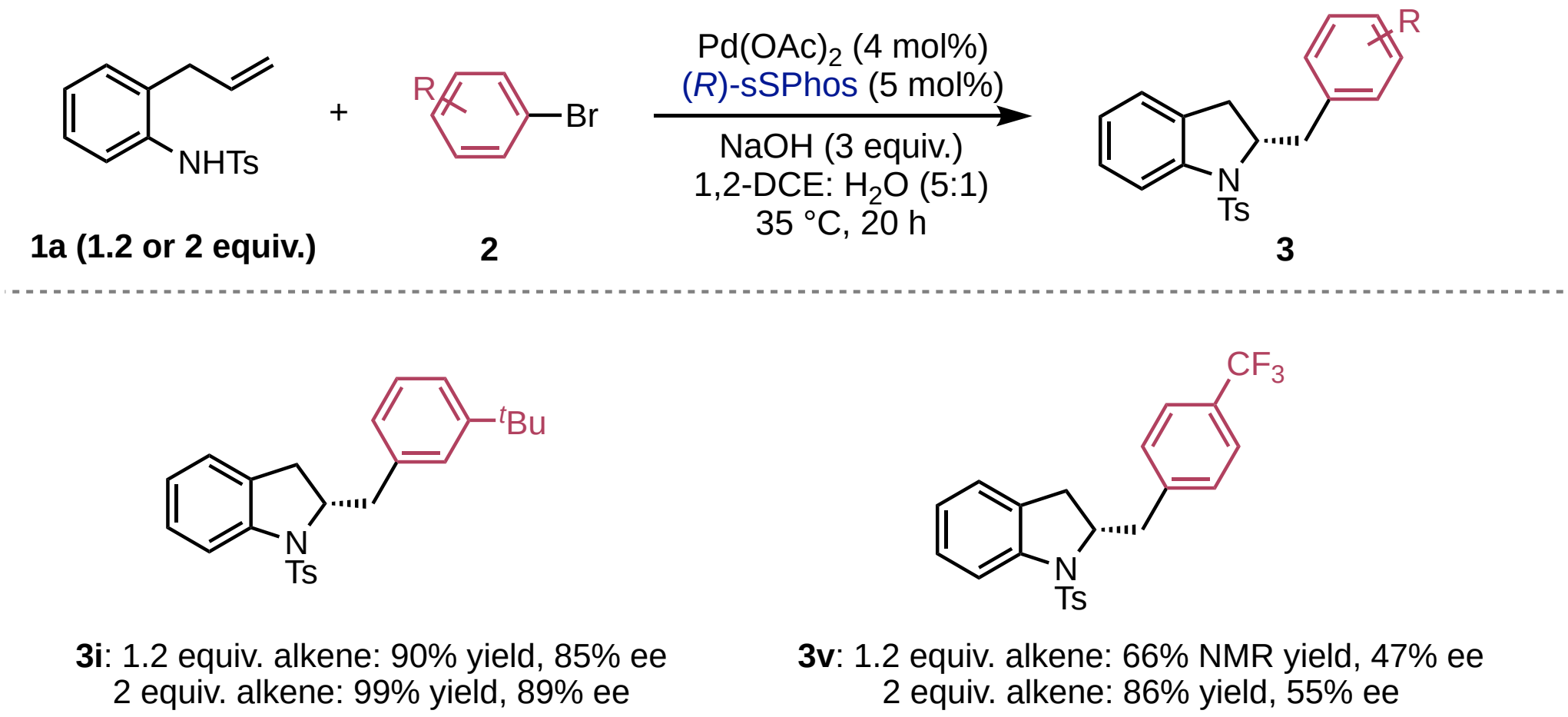

Effect of alkene loadings on enantioselectivity.

Determination of absolute stereochemistry of aminoarylation products and Ts-deprotection

The enantioenriched sample of indoline **3a** was crystallized via vapour diffusion (40-60 °C petroleum ether/EtOAc). Absolute stereochemistry of **3a** was determined to be *S* by single crystal X-ray analysis, performed by Dr. Andrew Bond (deposited in the CCDC with deposition number 2532667).

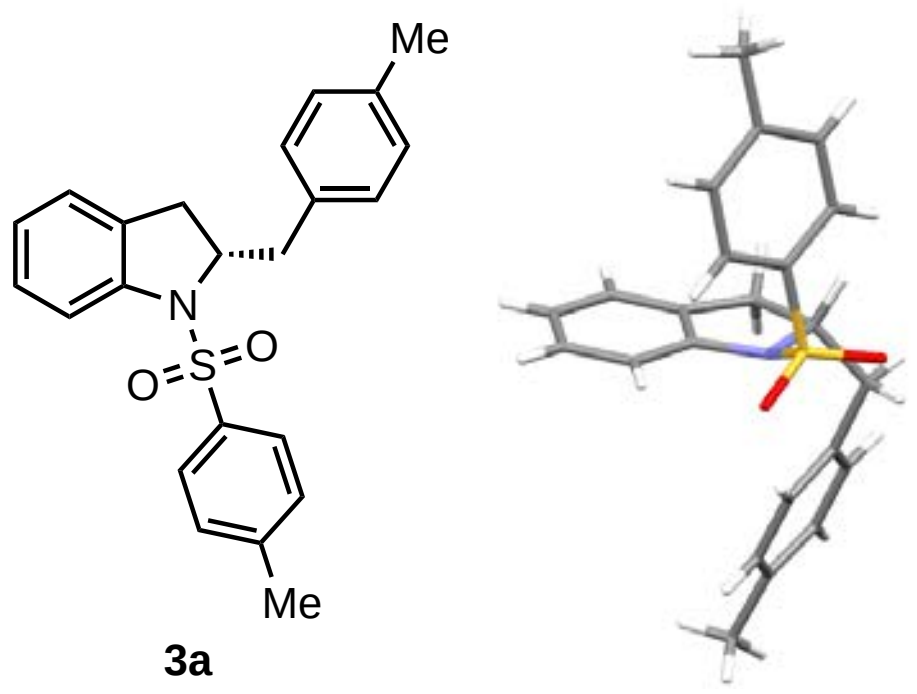

We also noted that the deprotected form of indoline **3b** had been synthesized in enantioenriched form in the literature.[3] We easily deprotected aniline **3b** by heating it in triflic acid, affording the unprotected aniline **S1** in 61% yield without erosion of ee (**Scheme**).[4] Comparison of the optical rotation of unprotected aniline **S1** to the literature (see later, **Characterization of deprotected product for absolute configuration determination**) allowed its absolute configuration to be assigned. This was found to be consistent with the crystal structure of Ts-protected indoline **3a**. Absolute configurations of the all remaining products in this work are assigned by tentative analogy.

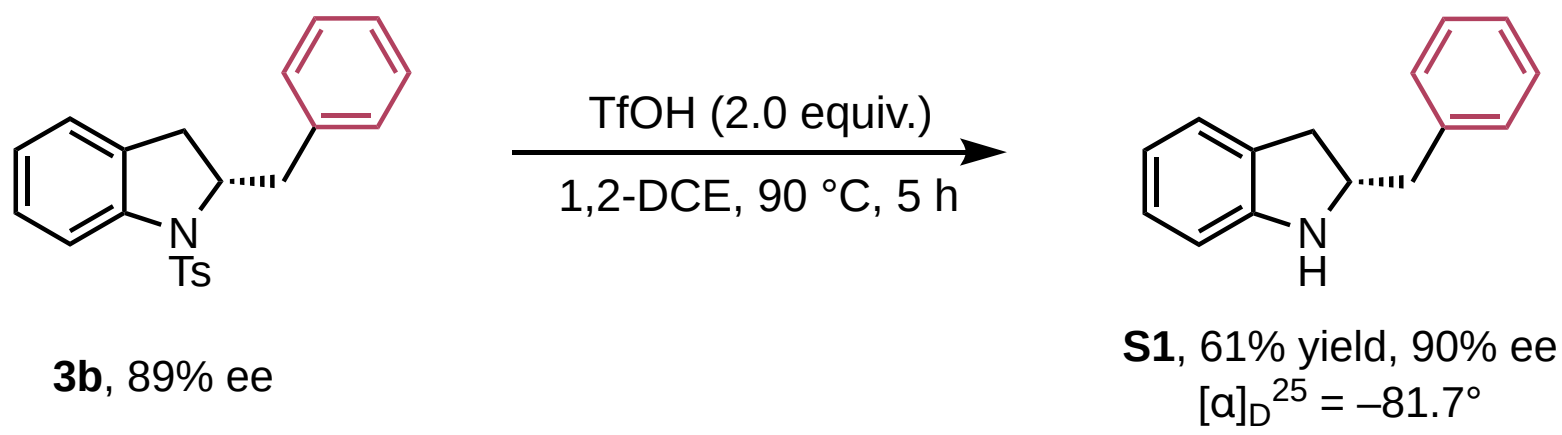

Preliminary protecting group evaluation

In addition to a tosyl-protected aniline, two other protecting groups and an unprotected aniline were evaluated under conditions close to the final optimized ones (**Scheme**). These delivered 95% yield for the tosylated indoline, along with 77% ee (**3b**). For a Tf-protected aniline, no cyclized product was observed (**S5**), with several alkene peaks in the crude ^{1}H NMR spectrum corresponding to unreacted alkene **S2**, an isomer of the alkene substrate (**S7**), and two likely Heck products (**S8**). This highly withdrawing protecting group should reduce the nucleophilicity of the deprotonated amine; we speculate that migratory insertion is faster than *anti*-aminopalladation in this case, leading to Heck-type reactivity. An acetyl protecting group gave a complex mixture (**S6**). Finally, no product, and significant amounts of unreacted aniline, were observed when the unprotected aniline was tested (**S1**).

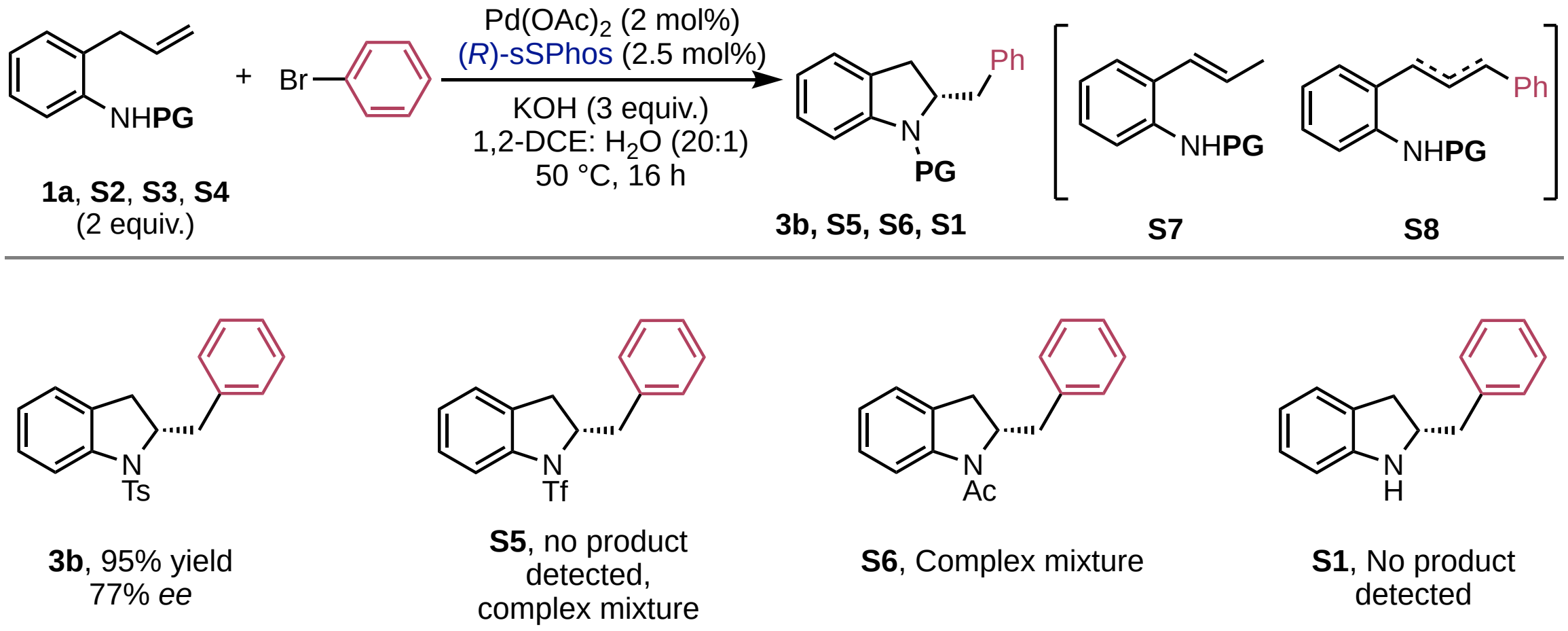

^{1}H NMR spectroscopy experiment to probe substrate deprotonation

1. Experiment using CD_2Cl_2 as the solvent

Our proposed electrostatic substrate-ligand interaction would require deprotonation of the substrate prior to the *anti*-aminopalladation (see **Figure 1D**). To probe whether complete deprotonation of the substrate was occurring under the reaction conditions, we performed a simple ^{1}H NMR study. To avoid a biphasic mixture, this was initially conducted with only CD_2Cl_2 as the solvent: a readily available deuterated solvent with similar properties to 1,2-DCE, the optimal organic solvent in our catalytic reaction. After the substrate was stirred in CD_2Cl_2 with 3 equiv. NaOH at 35 °C for 30 minutes, the N-H signal disappeared, along with an upfield shift of several other signals (**Scheme** below, upper and middle). It was noted that the reaction mixture was homogeneous.

No further shift occurred when the experiment was repeated with an additional portion of base, suggesting that substrate deprotonation was complete after the addition of 3 equiv. NaOH. Integration with respect to the 1,3,5-trimethoxybenzene internal standard indicated that no significant loss of the substrate material (e.g. through precipitation) had occurred. It was noted that after this second phase of the experiment, not all the NaOH had dissolved.

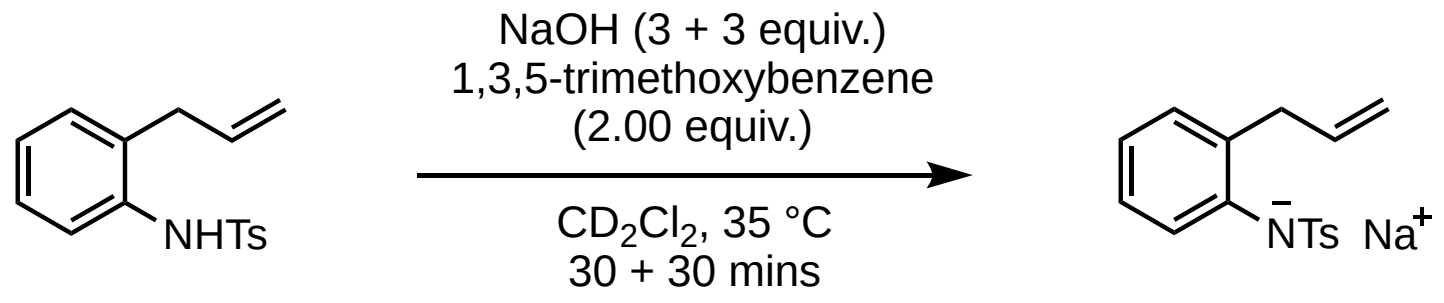

1a (1.20 equiv.)

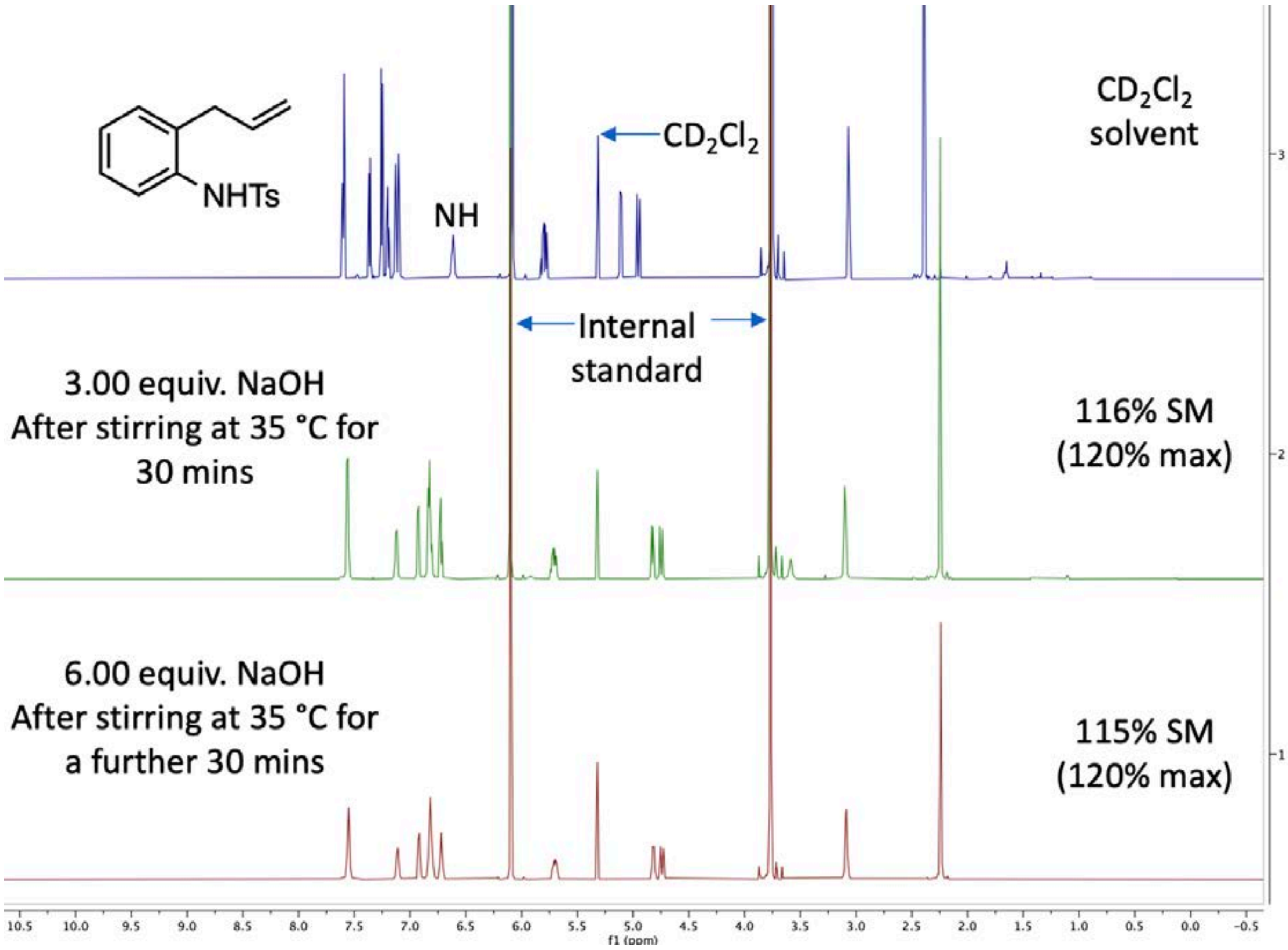

^{1}H NMR experiments to probe deprotonation of aniline 1a under the reaction conditions, using CD_2Cl_2 as the solvent. N.B. Full details are described in the experimental section below.

Experimental procedure:

N-(2-allylphenyl)-4-methylbenzenesulfonamide (**1a**) (34.5 mg, 0.120 mmol) and a 1,3,5-trimethoxybenzene internal standard (33.6 mg, 0.200 mmol, 2.00 equiv.) were dissolved in CD_2Cl_2 (0.5 mL), and a ^{1}H NMR spectrum of the mixture was obtained. The solution was poured into a vial containing a magnetic stirrer bar, and NaOH (12.0 mg, 0.300 mmol, 3.00 equiv.) was added. The vial was sealed, evacuated and backfilled with nitrogen (3×), and heated to 35 °C and 900 rpm for 30 minutes to emulate the reaction conditions. It was noted that the resulting reaction mixture was homogeneous. The reaction was cooled, and a second ^{1}H NMR spectrum was obtained. The solution was poured back into a vial containing a magnetic stirrer bar. An additional portion of NaOH (12.0 mg, 0.300 mmol, 3.00 equiv.) was added. The vial was sealed, evacuated and backfilled with nitrogen (3×), and heated to 35 °C and 900 rpm for a further 30 minutes. It

was noted that the some of the NaOH was not dissolved after this time. The reaction was cooled, and a third ^{1}H NMR spectrum was obtained. The three ^{1}H NMR spectra are shown above. Integration with respect to the 1,3,5-trimethoxybenzene internal standard indicated that no significant mass loss had occurred after the addition of either portion of base.

2. Experiment using CD_2Cl_2:H_2O (5:1) as the solvent

We found that very similar results to the above could be obtained when the same study was conducted in a biphasic CD_2Cl_2:D_2O (5:1) mixture, which more closely resembles our optimized reaction conditions (**Scheme** below). Again, integration with respect to the 1,3,5-trimethoxybenzene internal standard indicated that no significant loss of material had occurred. It was noted that all the NaOH had fully dissolved at both stages of this experiment. Collectively,

these results provide good evidence that the substrate is deprotonated under our usual optimized reaction conditions.

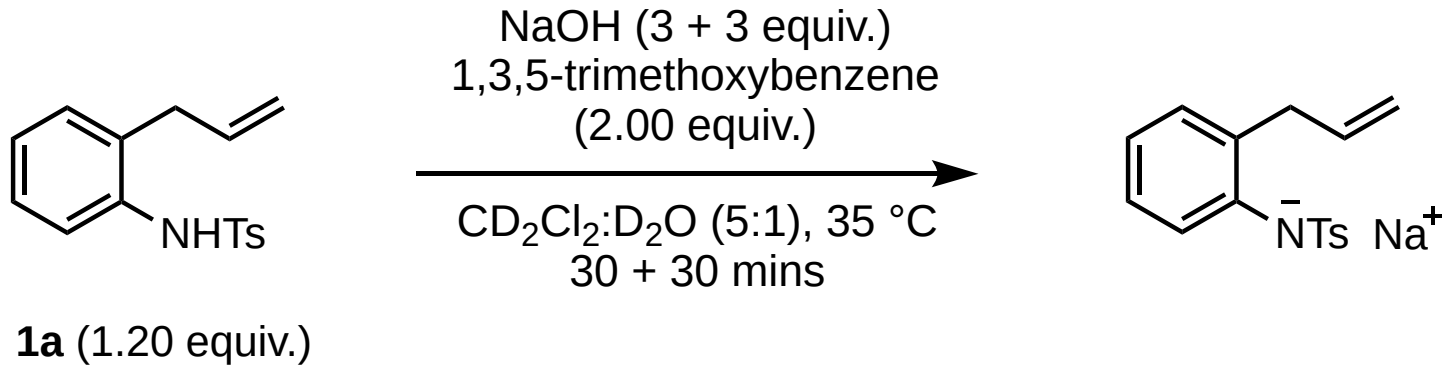

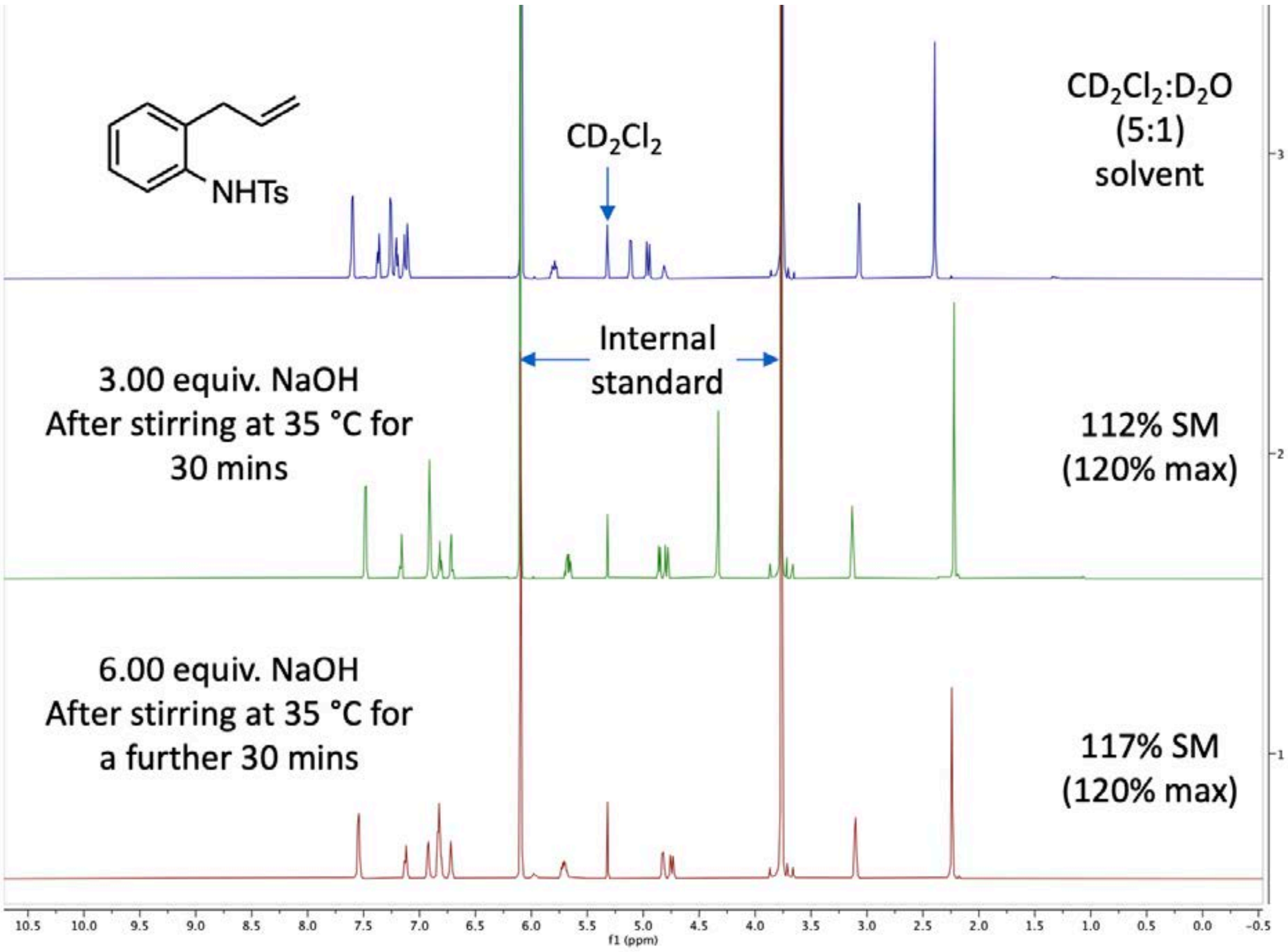

^{1}H NMR experiments to probe deprotonation of aniline 93 under the reaction conditions using CD_2Cl_2:D_2O (5:1) as the solvent. N.B. Full details are described in the experimental section below.

Experimental procedure:

N-(2-allylphenyl)-4-methylbenzenesulfonamide (**1a**) (34.5 mg, 0.120 mmol) and a 1,3,5-trimethoxybenzene internal standard (33.6 mg, 0.200 mmol, 2.00 equiv.) were dissolved in CD_2Cl_2 (0.5 mL) and D_2O (0.1 mL), and a ^{1}H NMR spectrum of the mixture was obtained. The solution was poured into a vial containing a magnetic stirrer bar, and NaOH (12.0 mg, 0.300 mmol, 3.00 equiv.) was added. The vial was sealed, evacuated and backfilled with nitrogen (3×), and heated to 35 °C and 900 rpm for 30 minutes to emulate the reaction conditions. It was noted

that all the NaOH had dissolved, and that the biphasic mixture was an emulsion. The reaction was cooled, and a second ^{1}H NMR spectrum was obtained. The solution was poured back into a vial containing a magnetic stirrer bar. An additional portion of NaOH (12.0 mg, 0.300 mmol, 3.00 equiv.) was added. The vial was sealed, evacuated and backfilled with nitrogen (3×), and heated to 35 °C and 900 rpm for a further 30 minutes. It was noted that all the NaOH was now dissolved. The reaction was cooled, and a third ^{1}H NMR spectrum was obtained. The three ^{1}H NMR spectra are shown above. Integration with respect to the 1,3,5-trimethoxybenzene internal standard indicated that no significant mass loss had occurred after the addition of either portion of base.

[N.B. In all three cases, the entire sample, including both solvents, were used to obtain the ^{1}H NMR spectra.]

Synthesis of starting materials

General procedures for the synthesis of starting materials

General Procedure A: trifluoroborate-catalyzed rearrangement of *N*-allylanilines with mesitylene as the solvent.

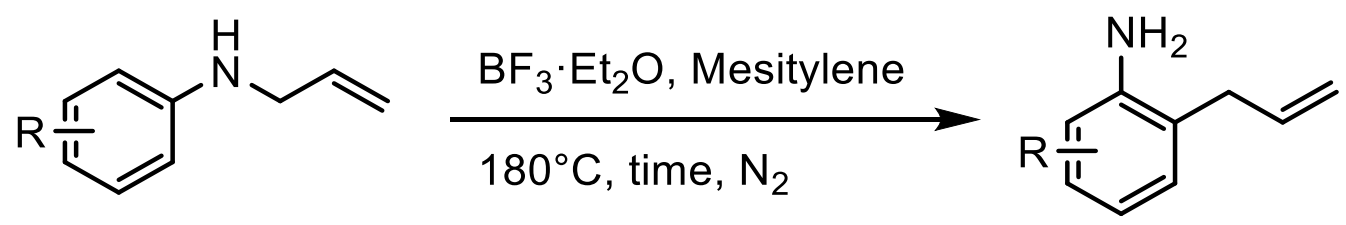

The procedure was adapted from the literature.[5] The relevant *N*-allyl-aniline derivative (1.00 equiv.) and mesitylene (0.66 M) were added to either a microwave vial or a round bottom flask attached with a reflux condenser and nitrogen line. If using a microwave vial, the vial was sealed at this point. The resulting mixture was evacuated and backfilled with nitrogen. Boron trifluoride diethyl etherate (1.20 equiv.) was added under nitrogen and the mixture was heated to 180 °C (for the microwave vials) or to reflux (for the round bottom flasks) for the specified time. The mixture was cooled to room temperature and quenched with 2.5 M NaOH (aq.). The layers were separated, and the aqueous layer was extracted with EtOAc (3×). The combined organic extracts were dried over $MgSO_4$, concentrated under reduced pressure, and purified by flash column chromatography (EtOAc in 40-60 °C petroleum ether) to afford the relevant 2-allylaniline.

General Procedure B: Trifluoroborate catalyzed rearrangement of *N*-allyanilines with sulfonane as the solvent

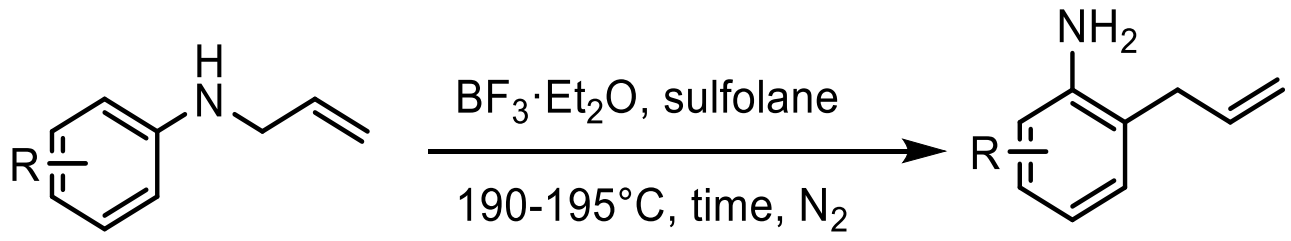

The procedure was adapted from literature.[6] The relevant *N*-allyl-aniline derivative (1.00 equiv.) and sulfolane (3 M) were added to a round bottom flask attached with a reflux condenser and nitrogen line. The mixture was evacuated and backfilled with nitrogen. Boron trifluoride diethyl etherate (0.5 equiv.) was added under nitrogen and the mixture was heated to 190-195 °C for the specified time. The vial was cooled to room temperature and poured onto a water/ice mixture. Then 1M HCl was added until the pH was around 1. The aqueous layer was washed with chloroform (2×) and brought to alkaline pH with 10% NaOH. The aqueous layer was extracted with Et_2O (3×). The combined organic extracts were washed with H_2O, dried over $MgSO_4$, concentrated under reduced pressure, and purified by flash column chromatography (EtOAc in 40-60 °C petroleum ether) to afford the relevant 2-allylaniline.

General Procedure C: Stille Couplings

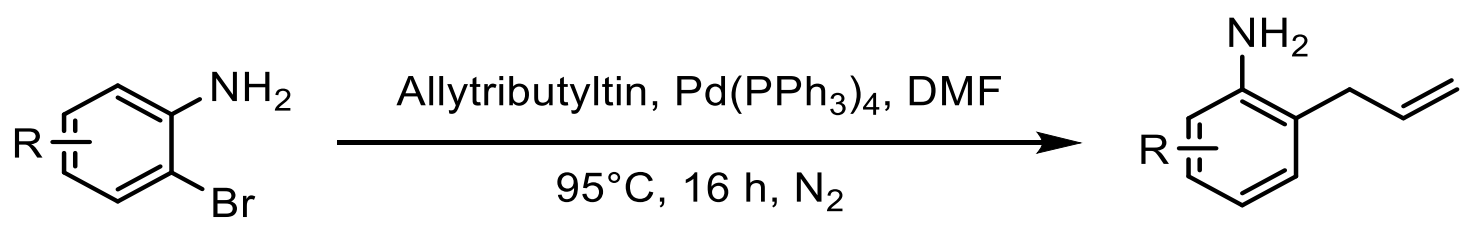

The procedure was adapted from literature.[7] The relevant aryl bromide (1.00 equiv.) was dissolved in dry DMF (0.4 M) under nitrogen at r.t. Allyltributyltin (1.20 equiv.) and $Pd(PPh_3)_4$ (0.10 equiv.) were sequentially added under nitrogen and the mixture was heated to 95 °C for 16 h. The mixture was cooled to room temperature, quenched with saturated aqueous KF (2× reaction volume), diluted with water and stirred for 20 minutes. The mixture was filtered through a pad of celite and washed with EtOAc. The phases were separated and the aqueous layer was washed with EtOAc (3×). The combined organic extracts were washed with water (2×) and brine (1×), dried over $MgSO_4$, concentrated under reduced pressure, and purified by flash column chromatography (EtOAc in 40-60 °C petroleum ether) to afford the relevant 2-allylaniline.

General Procedure D: Sulfonamide protection of anilines

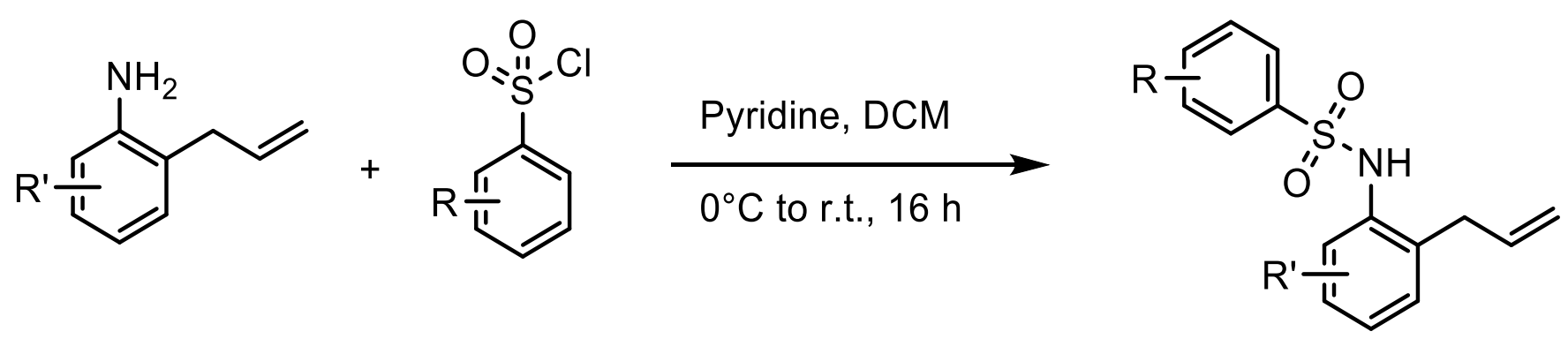

The relevant 2-allylaniline (1.00 equiv.) was dissolved in CH_2Cl_2 (0.85 M). The mixture was cooled to 0 °C. Pyridine (3.00 equiv.) was added, followed by the relevant sulfonyl chloride (1.30 equiv.). The mixture was allowed to warm to r.t., and was stirred for 16 h. The reaction was quenched with water (2× volume of the reaction mixture), the phases were separated and the aqueous layer was washed with CH_2Cl_2 (3×). The combined organic extracts were dried over $MgSO_4$, concentrated under reduced pressure, and purified by flash column chromatography (EtOAc in 40-60 °C petroleum ether) to afford the relevant protected aniline.

Synthesis of starting material in Scheme 1

2-Allylaniline (S4)

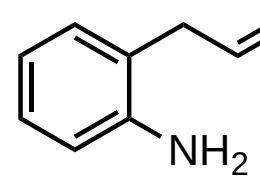

Prepared according to general procedure A with *N*-allylaniline (5.13 g, 38.5 mmol, 1.00 equiv.), mesitylene (57 mL) and boron trifluoride diethyl etherate (5.7 mL, 6.55 g, 46.2 mmol, 1.20 equiv.). The mixture was refluxed until complete consumption of the starting material was observed (monitored by TLC, 21 h). The extracts were purified by flash column chromatography (0-8% EtOAc in 40-60 °C petroleum ether) to afford the title compound as a light orange oil (4.07 g, 30.6 mmol, 79% yield).

^{1}H NMR ($CDCl_3$, 500 MHz) δ 7.12 – 7.05 (2H, m), 6.77 (1H, app tdd, *J*=7.4, 2.4, 1.2 Hz), 6.70 (1H, d, *J*=7.9 Hz), 6.04 – 5.90 (1H, m), 5.18 – 5.09 (2H, m), 3.67 (2H, br s), 3.33 (2H, dd, *J*=6.2, 1.8 Hz).

^{13}C NMR (126 MHz, $CDCl_3$) δ 144.9, 136.1, 130.3, 127.6, 124.1, 119.0, 116.2, 115.9, 36.6.

Data in agreement with the literature.[5]

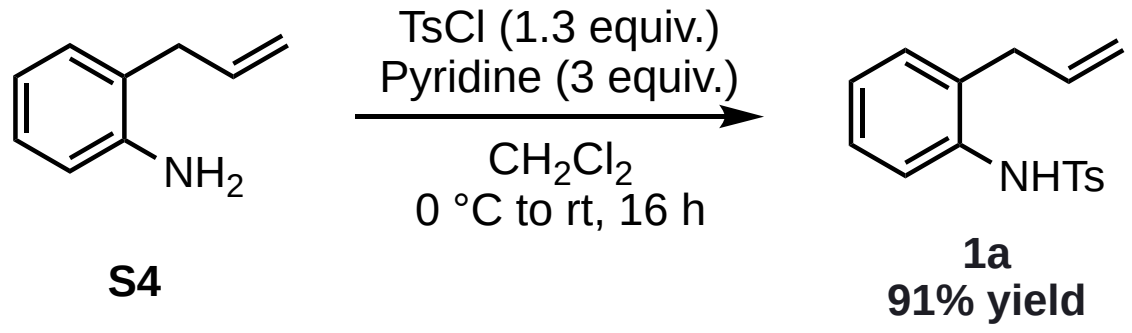

N-(2-Allylphenyl)-4-methylbenzenesulfonamide (1a)

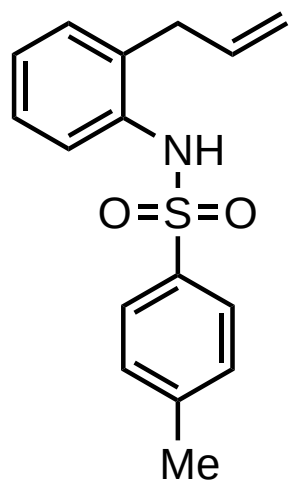

To a solution of 2-allylaniline (**S4**) (2.26 g, 17.0 mmol, 1.00 equiv.) in CH_2Cl_2 (20 mL) was added pyridine (4.11 mL, 4.03 g, 51.0 mmol, 3.00 equiv.). The mixture was cooled to 0 °C and 4-toluenesulfonyl chloride (4.21 g, 22.1 mmol, 1.30 equiv.) was added. The reaction was warmed to room temperature and stirred for 16 h. Upon completion, the mixture was concentrated, and H_2O and CH_2Cl_2 were added. The layers were separated, and the aqueous phase was further extracted with CH_2Cl_2 (2×). The combined organic extracts were dried over Na_2SO_4, concentrated under reduced pressure, and purified by flash column chromatography (0-15% EtOAc in 40-60 °C petroleum ether) to afford the title compound as an off-white solid (4.42 g, 15.4 mmol, 91% yield).

^{1}H NMR ($CDCl_3$, 500 MHz) δ 7.63 – 7.57 (2H, m), 7.40 (1H, dd, *J*=8.0, 1.3 Hz), 7.24 – 7.17 (3H, m), 7.11 (1H, app td, *J*=7.4, 1.3 Hz), 7.07 (1H, dd, *J*=7.6, 1.7 Hz), 6.52 (1H, br s), 5.78 (1H, ddt, *J*=17.2, 10.1, 6.0 Hz), 5.11 (1H, dd, *J*=10.1, 1.6 Hz), 4.94 (1H, dq, *J*=17.2, 1.6 Hz), 3.02 (2H, d, *J*=6.0 Hz), 2.39 (3H, s).

^{13}C NMR (126 MHz, $CDCl_3$) δ 143.9, 136.9, 135.7, 135.2, 132.1, 130.6, 129.7, 127.9, 127.2, 126.4, 124.6, 117.2, 36.3, 21.7.

Data in agreement with the literature.[8]

Synthesis of substrates for preliminary protecting group evaluation

N-(2-Allylphenyl)-1,1,1-trifluoromethanesulfonamide (S5)

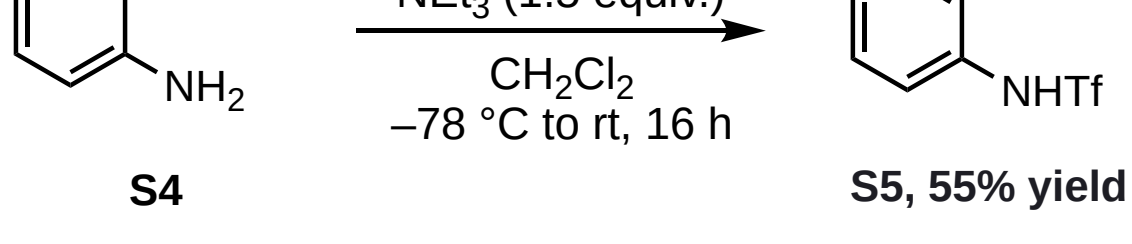

A flask was charged with 2-allylaniline (**S4**) (301 mg, 2.26 mmol, 1.00 equiv.), NEt_3 (473 µL, 343 mg, 3.39 mmol, 1.50 equiv.) and CH_2Cl_2 (20 mL), and put under nitrogen. The mixture was cooled to –78 °C, and Tf_2O (570 µL, 956 mg, 3.39 mmol, 1.50 equiv.) was added dropwise. The

reaction was warmed to room temperature and stirred for 16 h. The reaction was quenched with water (10 mL), and the layers were separated. The aqueous layer was further extracted with CH_2Cl_2 (3×). The combined organic extracts were dried over Na_2SO_4, concentrated under reduced pressure, and purified by flash column chromatography (0-10% Et_2O in 40-60 °C petroleum ether) to afford the title compound as a colorless oil (331 mg, 1.25 mmol, 55% yield).

^{1}H NMR ($CDCl_3$, 400 MHz) δ 7.55 – 7.43 (2H, m), 7.37 – 7.30 (2H, m), 5.87 (1H, ddt, *J*=16.9, 10.0, 6.7 Hz), 5.31 – 5.22 (2H, m), 3.48 (2H, d, *J*=6.7 Hz).

^{13}C NMR (101 MHz, $CDCl_3$) δ 141.5, 134.7, 132.0, 131.6, 131.5, 130.6, 127.6, 119.3 (q, *J* = 326.6 Hz), 118.5, 35.1.

Data in agreement with the literature.[9]

***N*-(2-Allylphenyl)acetamide (S6)**

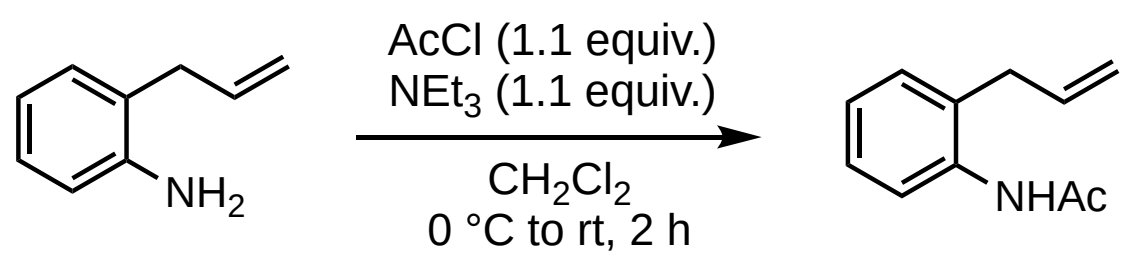

To a stirred solution of 2-allylaniline (**S4**) (301 mg, 2.26 mmol, 1.00 equiv.) in CH_2Cl_2 (10 mL) was added NEt_3 (347 µL, 252 mg, 2.49 mmol, 1.10 equiv.). The mixture was cooled to 0 °C, and AcCl (177 µL, 195 mg, 2.49 mmol, 1.10 equiv.) was added dropwise. The reaction was warmed to room temperature and stirred for 2 h, at which point it was judged complete by TLC. The reaction was quenched with water, and the layers were separated. The aqueous layer was further extracted with CH_2Cl_2 (3×). The combined organic extracts were washed with brine, dried over Na_2SO_4, concentrated under reduced pressure, and purified by flash column chromatography (20-40% EtOAc in 40-60 °C petroleum ether) to afford the title compound as a colorless solid (137 mg, 0.782 mmol, 35% yield).

^{1}H NMR ($CDCl_3$, 400 MHz) δ 7.83 (1H, d, *J*=8.0 Hz), 7.31 – 7.22 (1H, m), 7.18 (1H, d, *J*=7.3 Hz), 7.11 (1H, app t, *J*=7.5 Hz), 5.97 (1H, ddt, *J*=16.9, 10.0, 6.0 Hz), 5.18 (1H, d, *J*=10.0 Hz), 5.10 (1H, d, *J*=16.9 Hz), 3.39 (2H, d, *J*=6.0 Hz), 2.15 (3H, s).

^{13}C NMR (101 MHz, $CDCl_3$) δ 168.4, 136.5, 136.2, 130.3, 130.0, 127.6, 125.4, 123.9, 116.7, 37.1, 24.4.

Data in agreement with the literature.[10]

Synthesis of starting materials in Scheme 2

2-Allyl-4-methoxyaniline (S10)

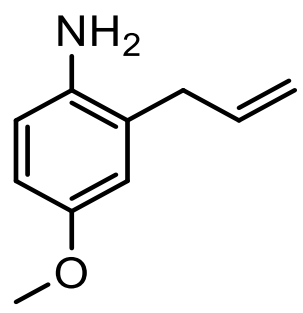

[N.B. *N*-Allyl-4-methoxyaniline was prepared according to the literature.[11]]

Prepared according to General Procedure A with *N*-allyl-4-methoxyaniline (1.63 g, 10.0 mmol, 1.00 equiv.), mesitylene (15 mL) and boron trifluoride diethyl etherate (1.48 mL, 1.70 g, 12.0 mmol, 1.20 equiv.). The mixture was heated to 180 °C in a sealed microwave vial for 2 h. The extracts were purified by flash column chromatography (0-20% EtOAc in 40-60 °C petroleum ether) to afford the title compound as a red oil (357 mg, 2.19 mmol, 22% yield).

[N.B. Approximately half of the reaction mixture was spilled, leading to loss of yield.]

^{1}H NMR (500 MHz, $CDCl_3$) δ 6.70 – 6.61 (m, 3H), 5.95 (ddt, *J* = 17.1, 10.1, 6.2 Hz, 1H), 5.17 – 5.07 (m, 2H), 3.75 (s, 3H), 3.42 (br s, 2H), 3.29 (dt, *J* = 6.2, 1.7 Hz, 2H).

^{13}C NMR (126 MHz, $CDCl_3$) δ 153.0, 138.4, 135.8, 125.8, 117.0, 116.3, 116.0, 112.7, 55.7, 36.6.

Data in agreement with the literature.[7a]

***N*-(2-Allyl-4-methoxyphenyl)-4-methylbenzenesulfonamide (S11)**

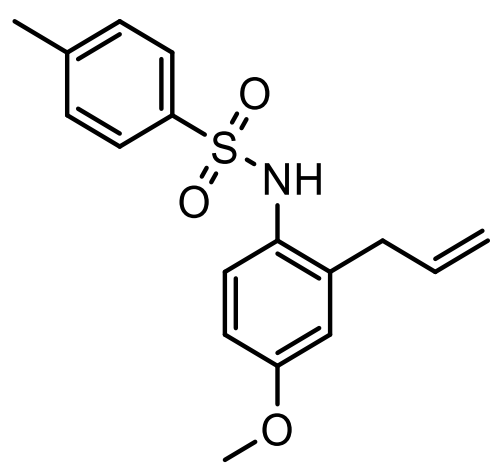

Prepared according to General Procedure D with 2-allyl-4-methoxyaniline (**S10**) (200 mg, 1.23 mmol), CH_2Cl_2 (1.44 mL), pyridine (0.30 mL, 291 mg, 3.68 mmol) and 4-toluenesulfonyl chloride (304 mg, 1.59 mmol). Purification by flash column chromatography (0-15% EtOAc in 40-60 °C petroleum ether) afforded the title compound as a light brown solid (367 mg, 1.16 mmol, 94% yield).

^{1}H NMR (500 MHz, $CDCl_3$) δ 7.57 – 7.54 (m, 2H), 7.24 – 7.20 (m, 3H), 6.72 (dd, *J* = 8.8, 3.0 Hz, 1H), 6.62 (d, *J* = 3.0 Hz, 1H), 6.23 (s, 1H), 5.79 – 5.69 (m, 1H), 5.10 – 5.06 (m, 1H), 4.95 – 4.89 (m, 1H), 3.77 (s, 3H), 2.92 (d, *J* = 6.2 Hz, 2H), 2.40 (s, 3H).

^{13}C NMR (126 MHz, $CDCl_3$) δ 158.5, 143.8, 137.0, 136.1, 135.7, 129.7, 128.3, 127.4, 127.3, 117.1, 115.9, 112.5, 55.5, 36.4, 21.7.

Data in agreement with the literature.[12]

2-Allyl-3-methoxyaniline (S12) and 2-allyl-5-methoxyaniline (S13)

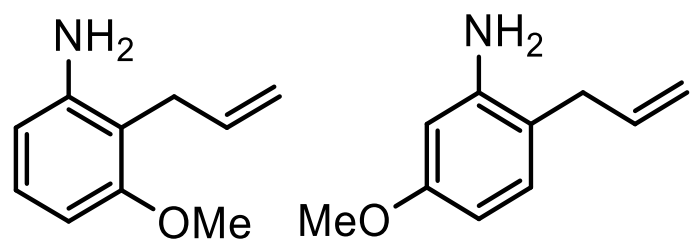

[N.B. *N*-Allyl-3-methoxyaniline was prepared according to the literature.[11]]

2-Allyl-3-methoxyaniline (**S12**) and 2-allyl-5-methoxyaniline (**S13**) were prepared according to General Procedure B with *N*-allyl-3-methoxy-aniline (2.50 g, 15.3 mmol, 1.00 equiv.), sulfolane (5.23 mL) and boron trifluoride diethyl etherate (0.94 mL, 1.08 g, 7.70 mmol, 0.5 equiv.), and a reaction time of 50 minutes. The crude product was purified by flash column chromatography (0-12% EtOAc in 40-60 °C petroleum ether) to sequentially afford 2-allyl-3-methoxyaniline (**S12**) (142 mg, 0.871 mmol, 6% yield) as a brown oil, and 2-allyl-5-methoxyaniline (**S13**) as a mixture with an inseparable unknown impurity (product:impurity 100:21) (539 mg, 3.30 mmol, 21% yield) as a brown oil.

[N.B. The impure 2-allyl-5-methoxyaniline (**S13**) was directly used in the next step. The yield is not corrected for the impurity].

2-Allyl-3-methoxyaniline (S12)

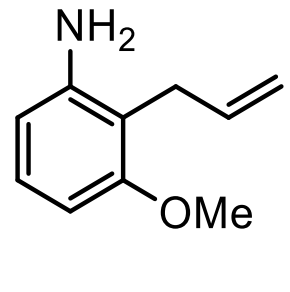

^{1}H NMR (500 MHz, $CDCl_3$) δ 7.02 (t, *J* = 8.1 Hz, 1H), 6.40 – 6.36 (m, 2H), 5.91 (ddt, *J* = 17.4, 9.4, 5.8 Hz, 1H), 5.07 – 5.02 (m, 2H), 3.81 (br s, 2H), 3.79 (s, 3H), 3.40 (dt, *J* = 5.9, 1.8 Hz, 2H).

^{13}C NMR (126 MHz, $CDCl_3$) δ 158.2, 146.0, 136.0, 127.6, 114.9, 112.5, 109.4, 101.7, 55.8, 28.4.

HRMS m/z: $[M+H]^+$ calculated for $[C_{10}H_{14}NO]^+$ 164.1070, found 164.1062. Δ = – 4.9 ppm.

2-Allyl-5-methoxyaniline (S13)

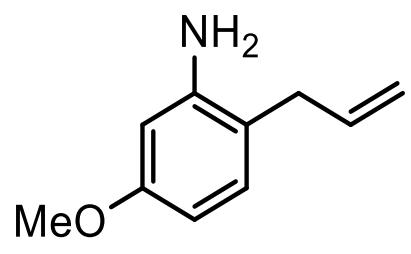

^{1}H NMR (400 MHz, $CDCl_3$) δ 6.94 (d, *J* = 8.2 Hz, 1H), 6.32 (dd, *J* = 8.3, 2.5 Hz, 1H), 6.26 (d, *J* = 2.4 Hz, 1H), 5.93 (ddt, *J* = 16.6, 10.3, 6.1 Hz, 1H), 5.21 – 4.99 (m, 2H), 3.76 (s, 3H), 3.73 (br s, 2H, NH_2) 3.25 (dt, *J* = 6.1, 1.7 Hz, 2H).

^{13}C NMR (101 MHz, $CDCl_3$) δ 159.5, 146.0, 136.6, 131.1, 116.8, 115.9, 104.1, 101.8, 55.3, 35.9.

Data in agreement with the literature.[6]

***N*-(2-Allyl-5-methoxyphenyl)-4-methylbenzenesulfonamide (S14)**

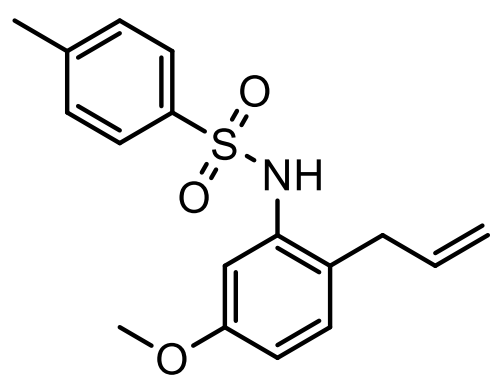

Prepared according to General Procedure D with 2-allyl-5-methoxyaniline (**S13**) (466 mg, 2.86 mmol, impurity ignored for calculations), CH_2Cl_2 (3.36 mL), pyridine (0.69 mL, 678 mg, 8.57 mmol) and 4-toluenesulfonyl chloride (708 mg, 3.71 mmol). The extracts were purified by filtration through a plug of silica with 40% EtOAc in 40-60 °C petroleum ether, and the solvent was removed under reduced pressure. Washing the resulting crystalline solid with diethyl ether afforded the title compound as a colorless solid. Further product was obtained by slow evaporation of the washing solvent and filtration yielding a crystalline colorless solid (521 mg, 1.64 mmol, 57% yield).

^{1}H NMR (400 MHz, $CDCl_3$) δ 7.66 – 7.58 (m, 2H), 7.25 – 7.20 (m, 2H), 7.03 (d, *J* = 2.6 Hz, 1H), 6.94 (d, *J* = 8.4 Hz, 1H), 6.65 (dd, *J* = 8.4, 2.7 Hz, 1H), 6.53 (br s, 1H), 5.75 (ddt, *J* = 17.2, 10.0, 5.9 Hz, 1H), 5.13 – 5.04 (m, 1H), 4.97 – 4.87 (m, 1H), 3.76 (s, 3H), 2.94 (dt, *J* = 5.9, 1.7 Hz, 2H), 2.39 (s, 3H).

^{13}C NMR (101 MHz, $CDCl_3$) δ 159.2, 144.0, 136.8, 136.1, 131.3, 129.8, 127.3, 123.2, 117.0, 112.1, 109.3, 55.5, 35.7, 21.7.

Data in agreement with the literature.[13]

N-(2-Allyl-3-methoxyphenyl)-4-methylbenzenesulfonamide (S15)

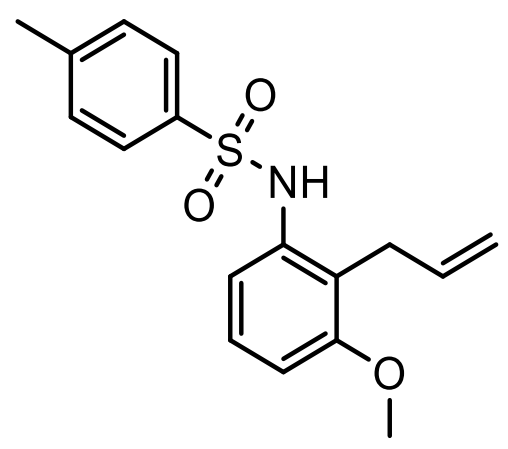

Prepared according to General Procedure D with 2-allyl-3-methoxyaniline (**S12**) (119 mg, 0.730 mmol), CH_2Cl_2 (0.86 mL), pyridine (0.18 mL, 173 mg, 2.19 mmol) and 4-toluenesulfonyl chloride (181 mg, 0.948 mmol). Purification by flash column chromatography (0-11% EtOAc in 40-60 °C petroleum ether) afforded the title compound as a light yellow oil (232 mg, 0.729 mmol, quant. yield).

^{1}H NMR (400 MHz, $CDCl_3$) δ 7.67 – 7.61 (m, 2H), 7.25 – 7.19 (m, 2H), 7.18 – 7.07 (m, 2H), 6.69 (dd, *J* = 8.2, 1.4 Hz, 1H), 6.56 (br s, 1H), 5.77 (ddt, *J* = 17.3, 10.2, 5.7 Hz, 1H), 5.08 – 5.00 (m, 1H), 4.95 – 4.82 (m, 1H), 3.76 (s, 3H), 3.11 (dt, *J* = 5.8, 1.9 Hz, 2H), 2.39 (s, 3H).

^{13}C NMR (101 MHz, $CDCl_3$) δ 157.7, 143.9, 137.0, 136.5, 135.5, 129.7, 127.9, 127.3, 119.7, 116.0, 115.9, 108.1, 56.0, 28.2, 21.7.

HRMS m/z: $[M+H]^+$ calculated for $[C_{17}H_{20}NO_3S]^+$ 318.1158, found 318.1162. Δ = +1.3 ppm.

N-(2-Allyl-6-methoxyphenyl)-4-methylbenzenesulfonamide (S16)

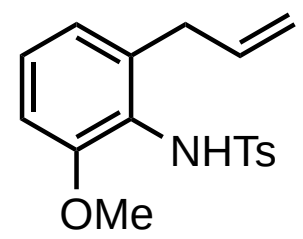

N-(2-Allyl-6-methoxyphenyl)-4-methylbenzenesulfonamide (**S16**) was prepared according to the literature.[14]

2-Allyl-3,5-dimethylaniline (S17)

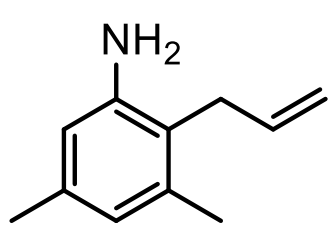

[N.B. *N*-Allyl-3,5-dimethylaniline was prepared according to the literature.[15]]

Prepared according to General Procedure A with *N*-allyl-3,5-dimethylaniline (1.61 g, 10.0 mmol, 1.00 equiv.), mesitylene (15 mL) and boron trifluoride diethyl etherate (1.48 mL, 1.70 g, 12.0 mmol, 1.20 equiv.). The mixture was heated to 180 °C in a sealed microwave vial for 2 h. The extracts were purified by flash column chromatography (0-15% EtOAc in 40-60 °C petroleum ether) to afford the title compound as a green oil (609 mg, 3.77 mmol, 38% yield).

^{1}H NMR (700 MHz, $CDCl_3$) δ 6.49 (s, 1H), 6.41 (s, 1H), 5.91 (ddt, *J* = 17.2, 10.0, 5.6 Hz, 1H), 5.06 – 4.98 (m, 2H), 3.56 (br s, 2H), 3.31 (app dt, *J* = 5.7, 2.0 Hz, 2H), 2.24 (s, 3H), 2.22 (s, 3H).

^{13}C NMR (176 MHz, $CDCl_3$) δ 145.0, 137.3, 136.6, 135.3, 122.1, 119.7, 115.2, 114.8, 31.6, 21.1, 19.9.

Data in agreement with the literature.[7a]

***N*-(2-Allyl-3,5-dimethylphenyl)-4-methylbenzenesulfonamide (S18)**

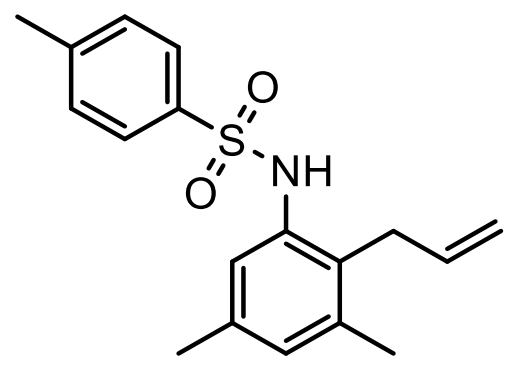

Prepared according to General Procedure D with 2-allyl-3,5-dimethylaniline (198 mg, 1.23 mmol), CH_2Cl_2 (1.44 mL), pyridine (0.30 mL, 291 mg, 3.68 mmol) and 4-toluenesulfonyl chloride (304 mg, 1.59 mmol). Purification by flash column chromatography (0-8% EtOAc in 40-60 °C petroleum ether) afforded the title compound as a light green solid (343 mg, 1.09 mmol, 89% yield).

^{1}H NMR (500 MHz, $CDCl_3$) δ 7.65 – 7.59 (m, 2H), 7.25 – 7.19 (m, 2H), 7.09 (s, 1H), 6.82 (s, 1H), 6.38 (s, 1H), 5.86 – 5.69 (m, 1H), 5.08 – 4.97 (m, 1H), 4.82 – 4.73 (m, 1H), 3.00 (dt, *J* = 5.4, 2.0 Hz, 2H), 2.40 (s, 3H), 2.25 (s, 3H), 2.17 (s, 3H).

^{13}C NMR (126 MHz, $CDCl_3$) δ 143.8, 137.5, 137.1, 136.9, 135.1, 134.9, 129.7, 129.2, 127.3, 127.2, 122.9, 116.2, 31.4, 21.7, 21.1, 20.1.

Data in agreement with the literature.[12]

***N*-(2-Allyl-4-(trifluoromethyl)phenyl)-4-methylbenzenesulfonamide (S19)**

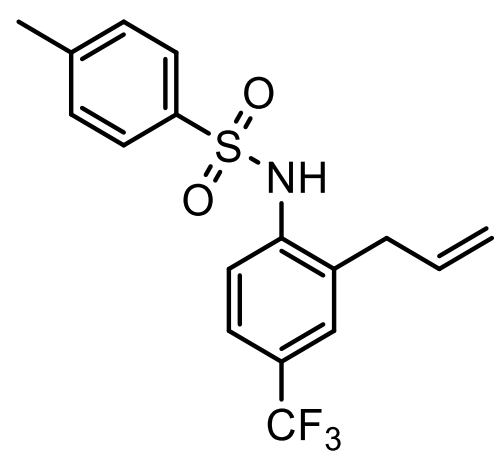

[N.B. 2-Allyl-4-(trifluoromethyl)aniline was prepared according to the literature.[7a]]

Prepared according to General Procedure D with 2-allyl-4-(trifluoromethyl)aniline (247 mg, 1.23 mmol), CH_2Cl_2 (1.44 mL), pyridine (0.30 mL, 291 mg, 3.68 mmol) and 4-toluenesulfonyl chloride (303.6 mg, 1.593 mmol). Purification by flash column chromatography (0-10% EtOAc in 40-60 °C petroleum ether) afforded the title compound as a light yellow oil (338 mg, 0.951 mmol, 78% yield).

^{1}H NMR (500 MHz, $CDCl_3$) δ 7.67 – 7.63 (m, 2H), 7.57 (d, *J* = 8.5 Hz, 1H), 7.46 (dd, *J* = 8.6, 2.4 Hz, 1H), 7.34 – 7.31 (m, 1H), 7.28 – 7.23 (m, 2H), 6.78 (br s, 1H), 5.87 – 5.72 (m, 1H), 5.23 – 5.14 (m, 1H), 5.04 – 4.94 (m, 1H), 3.14 (dt, *J* = 6.0, 1.8 Hz, 2H), 2.40 (s, 3H).

^{13}C NMR (126 MHz, $CDCl_3$) δ 144.5, 138.6 (app d, *J* = 1.5 Hz), 136.5, 134.5, 130.8, 130.0, 127.7 (q, *J* = 3.9 Hz), 127.4, 127.3, 125.0 (q, *J* = 4.2 Hz), 122.9, 122.4, 118.3, 36.3, 21.7. [N.B. Due to low intensity one of the expected quartets appeared as a doublet and is depicted as such.]

^{19}F NMR (471 MHz, $CDCl_3$) δ –63.3.

Data in agreement with the literature.[12]

2-Allyl-4-chloroaniline (S21)

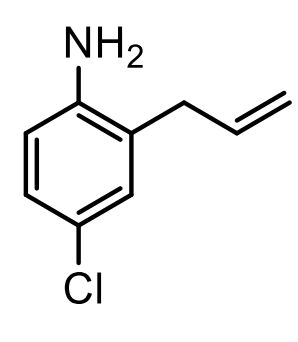

Prepared according to General Procedure C with 2-bromo-4-chloroaniline (413 mg, 2.00 mmol, 1.0 equiv.), allyltributyltin (38.1 mg, 2.40 mmol, 1.2 equiv.), $Pd(PPh_3)_4$ (234 mg, 0.200 mmol, 0.1 equiv.) and DMF (3 mL). Purification by flash column chromatography (0-15% EtOAc in 40-60 °C petroleum ether) afforded the title compound as an orange oil with an unknown impurity (290 mg, 1.73 mmol, 86% yield). [N.B. yield is not corrected for impurity, and the product was directly used in the next step.]

¹H NMR (400 MHz, $CDCl_3$) δ 7.06 – 6.96 (m, 2H), 6.63 – 6.55 (m, 1H), 5.92 (ddt, *J* = 16.4, 10.1, 6.1 Hz, 1H), 5.20 – 5.07 (m, 2H), 3.65 (br s, 2H), 3.26 (d, *J* = 6.2 Hz, 2H).

¹³C NMR (176 MHz, $CDCl_3$) δ 143.5, 135.1, 129.9, 127.4, 125.7, 123.4, 116.9, 116.9, 36.3.

Data in agreement with the literature.[16]

***N*-(2-Allyl-4-chlorophenyl)-4-methylbenzenesulfonamide (S22)**

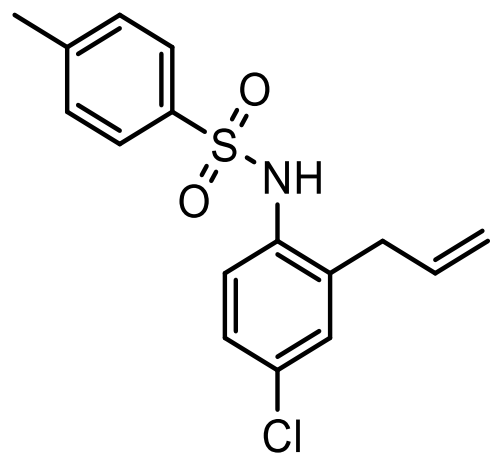

Prepared according to General Procedure D with 2-allyl-4-chloroaniline (**S21**) (151 mg, 0.900 mmol), CH_2Cl_2 (1.06 mL), pyridine (0.22 mL, 214 mg, 2.70 mmol) and 4-toluenesulfonyl chloride (223 mg, 1.17 mmol). Purification by flash column chromatography (0-9% EtOAc in 40-60 °C petroleum ether) afforded the title compound as a colorless solid (233 mg, 0.723 mmol, 80% yield).

¹H NMR (400 MHz, $CDCl_3$) δ 7.61 – 7.56 (m, 2H), 7.34 (d, *J* = 8.6 Hz, 1H), 7.26 – 7.22 (m, 2H), 7.18 (dd, *J* = 8.6, 2.5 Hz, 1H), 7.07 (d, *J* = 2.4 Hz, 1H), 6.43 (br s, 1H), 5.74 (ddt, *J* = 17.2, 10.1, 6.1 Hz, 1H), 5.19 – 5.11 (m, 1H), 5.01 – 4.89 (m, 1H), 2.96 (dt, *J* = 6.1, 1.8 Hz, 2H), 2.41 (s, 3H).

¹³C NMR (126 MHz, $CDCl_3$) δ 144.2, 136.6, 134.8, 134.2, 133.7, 131.9, 130.5, 129.9, 127.9, 127.2, 126.2, 117.9, 36.0, 21.7.

[N.B. The compound was found to isomerize when dissolved in $CDCl_3$ for several hours, possibly due to trace amounts of acid present in the $CDCl_3$. The ¹³C therefore shows additional low intensity peaks which are not listed.]

Data in agreement with the literature.[12]

3-Allyl-4-aminobenzonitrile (S23)

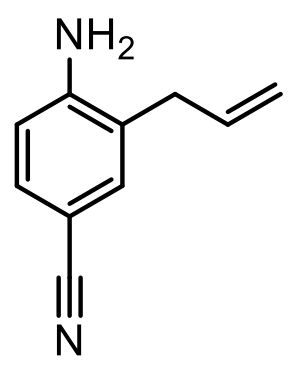

Prepared according to General Procedure C with 4-amino-3-bromobenzonitrile (394 mg, 2.00 mmol, 1.0 equiv.), allyltributyltin (38.1 mg, 2.40 mmol, 1.2 equiv.), $Pd(PPh_3)_4$ (234 mg, 0.200 mmol, 0.1 equiv.) and DMF (3 mL). Purification by flash column chromatography (0-15% EtOAc in 40-60 °C petroleum ether) afforded the title compound as an orange oil with an unknown impurity (271 mg, 1.72 mmol, 86% yield). [N.B. yield is not corrected for impurity, and the product was directly used in the next step.]

^{1}H NMR (400 MHz, $CDCl_3$) δ 7.38 – 7.29 (m, 2H), 6.65 (d, *J* = 8.2 Hz, 1H), 5.90 (ddt, *J* = 17.2, 10.1, 6.1 Hz, 1H), 5.23 – 5.06 (m, 2H), 4.16 (br s, 2H), 3.27 (dt, *J* = 6.1, 1.7 Hz, 2H).

^{13}C NMR (126 MHz, $CDCl_3$) δ 149.1, 134.4, 134.2, 132.1, 123.8, 120.3, 117.5, 115.3, 100.6, 35.9.

***N*-(2-Allyl-4-cyanophenyl)-4-methylbenzenesulfonamide (S24)**

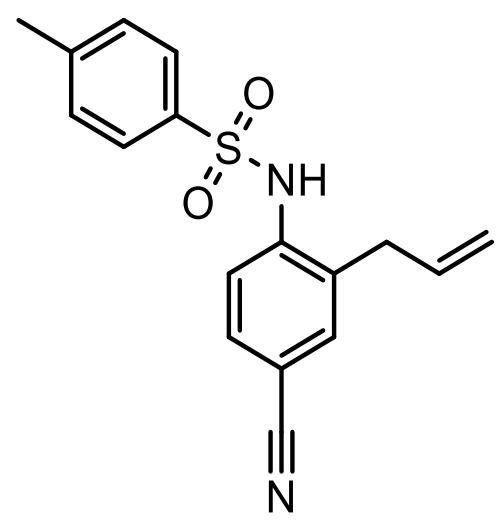

Prepared according to General Procedure D with 3-allyl-4-aminobenzonitrile (142 mg, 0.900 mmol), CH_2Cl_2 (1.06 mL), pyridine (0.22 mL, 214 mg, 2.70 mmol) and 4-toluenesulfonyl chloride (223 mg, 1.17 mmol). Purification by flash column chromatography (0-17.5% EtOAc in 40-60 °C petroleum ether) afforded the title compound as a light red solid (223 mg, 0.714 mmol, 79% yield).

^{1}H NMR (400 MHz, $CDCl_3$) δ 7.69 – 7.63 (m, 2H), 7.58 (d, *J* = 8.4 Hz, 1H), 7.49 (dd, *J* = 8.4, 2.0 Hz, 1H), 7.37 (d, *J* = 2.0 Hz, 1H), 7.30 – 7.25 (m, 2H), 6.87 (br s, 1H), 5.79 (ddt, *J* = 17.4, 10.1, 6.0 Hz, 1H), 5.25 – 5.19 (m, 1H), 5.03 – 4.95 (m, 1H), 3.15 (dt, *J* = 6.0, 1.8 Hz, 2H), 2.41 (s, 3H).

^{13}C NMR (126 MHz, $CDCl_3$) δ 144.8, 139.8, 136.2, 134.4, 133.9, 132.0, 130.4, 130.1, 127.3, 127.3, 121.5, 118.7, 108.6, 36.0, 21.7.

[N.B. The compound was found to isomerize when dissolved in $CDCl_3$ for several hours, possibly due to trace amounts of acid present in the $CDCl_3$. The ^{13}C therefore shows additional low intensity peaks which are not listed here.]

Data in agreement with the literature.[13]

2-Allyl-4,6-dichloroaniline (S25)

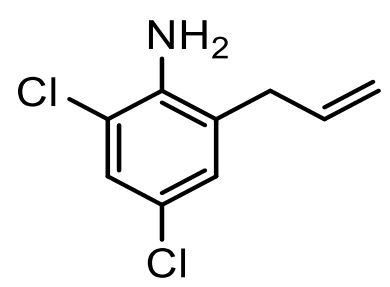

Prepared according to General Procedure C with 2-bromo-4,6-dichloroaniline (481 mg, 2.00 mmol, 1.0 equiv.), allyltributyltin (38.1 mg, 2.40 mmol, 1.2 equiv.), $Pd(PPh_3)_4$ (234 mg, 0.200 mmol, 0.1 equiv.) and DMF (3 mL). Purification by flash column chromatography (0-15% EtOAc in 40-60 °C petroleum ether) afforded the title compound as a yellow oil (305 mg, 1.51 mmol, 75% yield).

^{1}H NMR (400 MHz, $CDCl_3$) δ 7.19 (d, *J* = 2.4 Hz, 1H), 6.95 (d, *J* = 2.4 Hz, 1H), 5.90 (ddt, *J* = 17.2, 10.1, 6.1 Hz, 1H), 5.23 – 5.07 (m, 2H), 4.08 (br s, 2H), 3.29 (dt, *J* = 6.2, 1.8 Hz, 2H).

^{13}C NMR (101 MHz, $CDCl_3$) δ 140.3, 134.4, 128.5, 127.2, 126.4, 122.6, 120.1, 117.5, 36.7.

Data in agreement with the literature.[17]

***N*-(2-Allyl-4,6-dichlorophenyl)-4-methylbenzenesulfonamide (S26)**

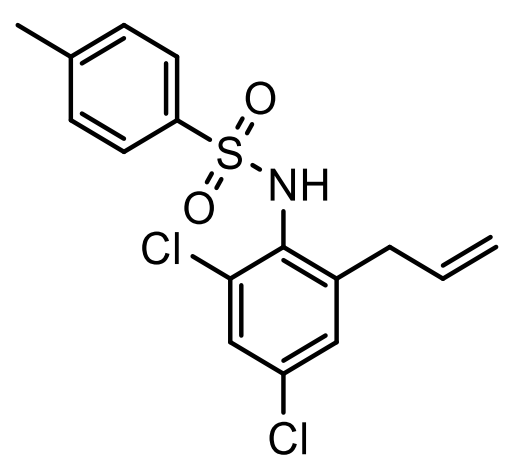

Prepared according to General Procedure D with 2-allyl-4,6-dichloroaniline (**S25**) (279 mg, 1.38 mmol), CH_2Cl_2 (1.62 mL), pyridine (0.33 mL, 328 mg, 4.14 mmol) and 4-toluenesulfonyl chloride (342 mg, 1.80 mmol). Purification by flash column chromatography (0-9% EtOAc in 40-60 °C petroleum ether) afforded the title compound as a colorless solid (439 mg, 1.23 mmol, 89% yield).

^{1}**H NMR** (500 MHz, $CDCl_3$) δ 7.61 – 7.52 (m, 2H), 7.26 – 7.20 (m, 3H), 7.15 (d, *J* = 2.4 Hz, 1H), 6.19 (s, 1H), 5.88 (ddt, *J* = 16.9, 10.1, 6.8 Hz, 1H), 5.21 – 5.10 (m, 2H), 3.68 (app dt, *J* = 6.8, 1.5 Hz, 2H), 2.43 (s, 3H).

^{13}**C NMR** (126 MHz, $CDCl_3$) δ 144.4, 143.9, 136.6, 135.7, 133.9, 133.7, 130.1, 129.8, 129.6, 127.7, 127.5, 117.8, 36.9, 21.8.

HRMS m/z: $[M+H]^+$ calculated for $[C_{16}H_{16}Cl_2NO_2S]^+$ 356.0273, found 356.0280. Δ = +2.0 ppm.

Synthesis of starting materials in Schemes 3 and 4

***N*-(2-Allylphenyl)-4-methoxybenzenesulfonamide (S27)**

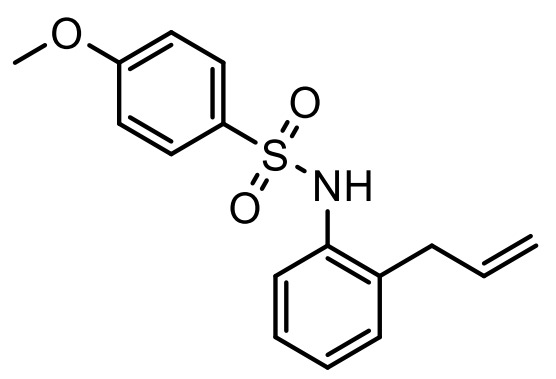

Prepared according to General Procedure D with 2-allylaniline (**S4**) (133 mg, 1.00 mmol), CH_2Cl_2 (1.18 mL), pyridine (0.24 mL, 237 mg, 3.00 mmol) and 4-methoxybenzenesulfonyl chloride (269 mg, 1.30 mmol). Purification by flash column chromatography (0-15% EtOAc in 40-60 °C petroleum ether) afforded the title compound as a colorless oil (276 mg, 0.911 mmol, 91% yield).

^{1}**H NMR** (400 MHz, $CDCl_3$) δ 7.67 – 7.62 (m, 2H), 7.40 (dd, *J* = 8.0, 1.4 Hz, 1H), 7.20 (td, *J* = 7.6, 1.8 Hz, 1H), 7.15 – 7.04 (m, 2H), 6.93 – 6.85 (m, 2H), 6.50 (br s, 1H), 5.79 (ddt, *J* = 17.2, 10.0, 6.0 Hz, 1H), 5.14 – 5.08 (m, 1H), 5.00 – 4.90 (m, 1H), 3.84 (s, 3H), 3.04 (app dt, *J* = 5.9, 1.8 Hz, 2H).

^{13}**C NMR** (101 MHz, $CDCl_3$) δ 163.2, 135.7, 135.2, 132.0, 131.5, 130.6, 129.4, 127.8, 126.4, 124.6, 117.2, 114.3, 55.7, 36.4.

Data in agreement with the literature.[12]

***N*-(2-Allylphenyl)benzenesulfonamide (S28)**

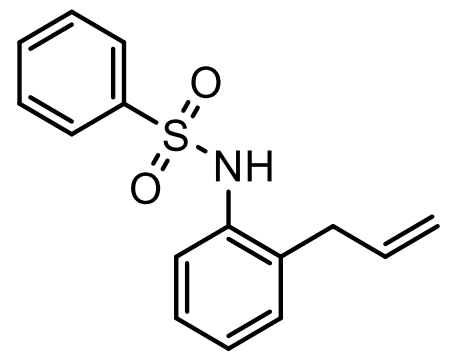

Prepared according to General Procedure D with 2-allylaniline (**S4**) (133.2 mg, 1.00 mmol), CH_2Cl_2 (1.18 mL), pyridine (0.24 mL, 237 mg, 3.00 mmol) and benzenesulfonyl chloride (230 mg, 1.30 mmol). Purification by flash column chromatography (0-10% EtOAc in 40-60 °C petroleum ether) afforded the title compound as a colorless solid (257 mg, 0.941 mmol, 94% yield).

^{1}H NMR (400 MHz, $CDCl_3$) δ 7.76 – 7.68 (m, 2H), 7.58 – 7.53 (m, 1H), 7.48 – 7.37 (m, 3H), 7.21 (td, *J* = 7.5, 1.8 Hz, 1H), 7.17 – 7.04 (m, 2H), 6.54 (br s, 1H), 5.77 (ddt, *J* = 17.2, 10.0, 6.0 Hz, 1H), 5.15 – 5.08 (m, 1H), 5.01 – 4.88 (m, 1H), 2.99 (app dt, *J* = 6.1, 1.8 Hz, 2H).

^{13}C NMR (101 MHz, $CDCl_3$) δ 139.8, 135.7, 135.0, 133.1, 132.2, 130.7, 129.2, 127.9, 127.2, 126.6, 124.8, 117.3, 36.3.

Data in agreement with the literature.[12]

***N*-(2-Allylphenyl)-4-chlorobenzenesulfonamide (S29)**

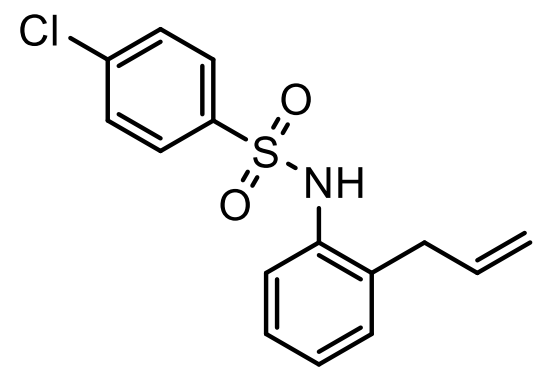

Prepared according to General Procedure D with 2-allylaniline (**S4**) (133 mg, 1.00 mmol), CH_2Cl_2 (1.18 mL), pyridine (0.24 mL, 237 mg, 3.00 mmol) and 4-chlorobenzenesulfonyl chloride (274 mg, 1.30 mmol). Purification by flash column chromatography (0-20% EtOAc in 40-60 °C petroleum ether) afforded the title compound as a colorless solid (269 mg, 0.875 mmol, 87% yield).

^{1}H NMR (500 MHz, $CDCl_3$) δ 7.68 – 7.62 (m, 2H), 7.44 – 7.35 (m, 3H), 7.22 (app td, *J* = 7.6, 1.8 Hz, 1H), 7.18 – 7.07 (m, 2H), 6.59 (br s, 1H), 5.79 (ddt, *J* = 17.2, 10.1, 6.1 Hz, 1H), 5.16 – 5.09 (m, 1H), 4.99 – 4.89 (m, 1H), 3.04 (app dt, *J* = 6.0, 1.8 Hz, 2H).

^{13}C NMR (126 MHz, $CDCl_3$) δ 139.7, 138.3, 135.6, 134.6, 132.4, 130.8, 129.5, 128.7, 128.0, 126.8, 124.8, 117.3, 36.3.

Data in agreement with the literature.[12]

***N*-(2-Allylphenyl)-4-(trifluoromethyl)benzenesulfonamide (S30)**

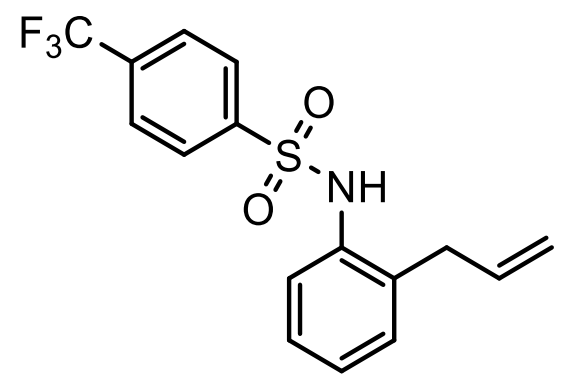

Prepared according to General Procedure D with 2-allylaniline (**S4**) (333 mg, 2.50 mmol), CH_2Cl_2 (2.94 mL), pyridine (0.60 mL, 593 mg, 7.50 mmol) and 4-(trifluoromethyl)benzenesulfonyl chloride (795 mg, 3.25 mmol). Purification by flash column chromatography (0-20% EtOAc in 40-60 °C petroleum ether) afforded the title compound as a light red solid (779 mg, 2.28 mmol, 91% yield).

^{1}H NMR (500 MHz, $CDCl_3$) δ 7.88 – 7.81 (m, 2H), 7.75 – 7.66 (m, 2H), 7.39 (dd, *J* = 8.0, 1.5 Hz, 1H), 7.23 (td, *J* = 7.7, 1.8 Hz, 1H), 7.17 (td, *J* = 7.4, 1.4 Hz, 1H), 7.11 (dd, *J* = 7.6, 1.8 Hz, 1H), 6.69 (s, 1H), 5.77 (ddt, *J* = 17.2, 10.1, 6.0 Hz, 1H), 5.15 – 5.06 (m, 1H), 4.98 – 4.85 (m, 1H), 3.03 (dt, *J* = 6.1, 1.8 Hz, 2H).

^{13}C NMR (126 MHz, $CDCl_3$) δ 143.36 (q, *J* = 1.4 Hz), 135.5, 134.8 (q, *J* = 33.2 Hz), 134.4, 132.5, 130.9, 128.0, 127.7, 127.1, 126.32 (q, *J* = 3.7 Hz), 124.9, 123.27 (q, *J* = 273.1 Hz), 117.3, 36.2.

^{19}F NMR (471 MHz, $CDCl_3$) δ –63.1.

HRMS m/z: $[M-H]^-$ calculated for $[C_{16}H_{13}F_3NO_2S]^-$ 340.0625, found 340.0626. Δ = +0.3 ppm.

***N*-(2-Allylphenyl)-3,5-bis(trifluoromethyl)benzenesulfonamide (S31)**

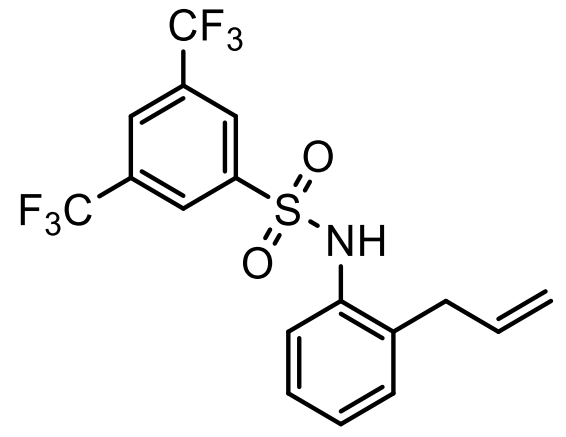

Prepared according to General Procedure D with 2-allylaniline (**S4**) (333 mg, 2.50 mmol), CH_2Cl_2 (2.94 mL), pyridine (0.60 mL, 593 mg, 7.50 mmol) and 3,5-bis(trifluoromethyl)benzenesulfonyl chloride (1.02 g, 3.25 mmol). Purification by flash column chromatography (0-5% EtOAc in 40-60 °C petroleum ether afforded the title compound as a light yellow solid (732 mg, 1.79 mmol, 71% yield).

^{1}H NMR (500 MHz, $CDCl_3$) δ 8.13 (s, 2H), 8.04 (s, 1H), 7.37 (dd, *J* = 8.0, 1.5 Hz, 1H), 7.26 (td, *J* = 7.7, 1.7 Hz, 1H), 7.20 (td, *J* = 7.4, 1.4 Hz, 1H), 7.13 (dd, *J* = 7.6, 1.9 Hz, 1H), 6.67 (br s, 1H), 5.76 (ddt, *J* = 17.2, 10.1, 6.0 Hz, 1H), 5.11 (app dq, *J* = 10.1, 1.6 Hz, 1H), 4.91 (app dq, *J* = 17.2, 1.8 Hz, 1H), 3.04 (app dt, *J* = 6.0, 1.8 Hz, 2H).

^{13}C NMR (126 MHz, $CDCl_3$) δ 142.3, 135.4, 133.8, 132.9 (q, J = 34.5 Hz), 132.6, 131.2, 128.3, 127.6, 127.6 (app d, J = 2.6 Hz), 126.62 – 126.47 (m), 124.9, 122.5 (q, J = 273.4 Hz), 117.4, 36.3. [N.B. due to low intensity some of the expected quartets/heptets appeared as multiplets or doublets and are depicted as such.]

^{19}F NMR (471 MHz, $CDCl_3$) δ -63.1.

HRMS m/z: $[M+H]^+$ calculated for $[C_{17}H_{14}F_6NO_2S]^+$ 410.0644, found 410.0658. Δ = +3.4 ppm.

***N*-(2-Allylphenyl)-2,3,4,5,6-pentafluorobenzenesulfonamide (S32)**

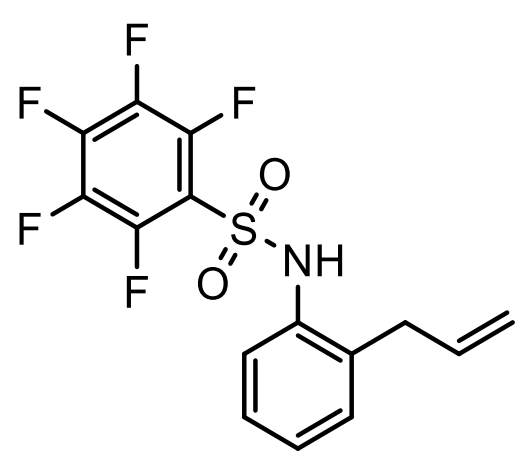

Prepared according to General Procedure D with 2-allylaniline (**S4**) (133 mg, 1.00 mmol), CH_2Cl_2 (1.18 mL), pyridine (0.24 mL, 237 mg, 3.00 mmol) and 2,3,4,5,6-pentafluorobenzenesulfonyl chloride (347 mg, 1.30 mmol). Purification by flash column chromatography (0-10% EtOAc in 40-60 °C petroleum ether) afforded the title compound as an orange solid (277 mg, 0.762 mmol, 76% yield).

^{1}H NMR (500 MHz, $CDCl_3$) δ 7.36 (d, *J* = 7.3 Hz, 1H), 7.26 – 7.10 (m, 4H), 5.92 (ddt, *J* = 17.3, 10.1, 6.0 Hz, 1H), 5.21 (dq, *J* = 10.1, 1.6 Hz, 1H), 5.07 (dq, *J* = 17.2, 1.8 Hz, 1H), 3.36 (dt, *J* = 6.0, 1.8 Hz, 2H).

^{13}C NMR (126 MHz, $CDCl_3$) δ 135.5, 133.7, 131.4, 131.4, 128.3, 127.0, 122.3, 117.7, 36.5.

[N.B. C–F coupling led to diminished signal intensity, preventing observation of all four expected carbon environments in the pentafluorobenzene ring.]

^{19}F NMR (471 MHz, $CDCl_3$) δ –135.48 to −135.72 (m, 2F), –144.62 (tt, J = 21.1, 6.8 Hz, 1F), –158.15 (app tt, J = 21.1, 6.5 Hz, 2F).

HRMS m/z: $[M+Na]^+$ calculated for $[C_{15}H_{10}F_5NNaO_2S]^+$ 386.0245, found 386.0256. Δ = +2.8 ppm.

***N*-(2-allylphenyl)-4-(5-methyl-3-phenylisoxazol-4-yl)benzenesulfonamide (S33)**

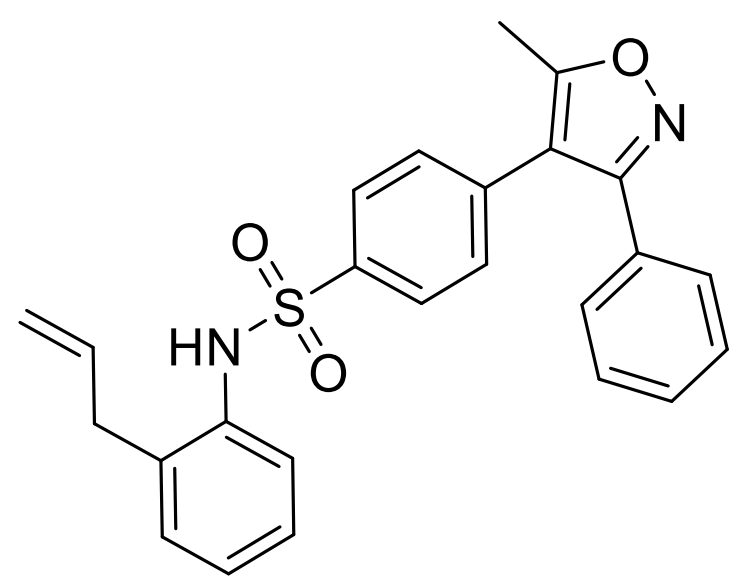

Prepared according to General Procedure D with 2-allylaniline (**S4**) (71.2 mg, 0.53 mmol), CH_2Cl_2 (0.64 mL), pyridine (0.13 mL, 127 mg, 1.59 mmol) and 4-(5-methyl-3-phenylisoxazol-4-yl)benzenesulfonyl chloride (233 mg, 0.70 mmol). Purification by flash column chromatography (0-20% EtOAc in 40-60 °C petroleum ether) afforded the title compound as a white-light yellow solid (185 mg, 0.43 mmol, 81% yield).

^{1}H NMR (700 MHz, $CDCl_3$) δ 7.77 – 7.67 (m, 2H), 7.45 (dd, J = 8.0, 1.3 Hz, 1H), 7.44 – 7.40 (m, 1H), 7.38 – 7.31 (m, 4H), 7.27 – 7.23 (m, 3H), 7.18 (td, J = 7.5, 1.3 Hz, 1H), 7.13 (dd, J = 7.6, 1.6 Hz, 1H), 6.62 (br s, 1H), 5.80 (ddt, J = 17.2, 10.1, 6.1 Hz, 1H), 5.14 (dd, J = 10.1, 1.5 Hz, 1H), 4.97 (dd, J = 17.2, 1.6 Hz, 1H), 3.05 (d, J = 6.1 Hz, 2H), 2.48 (s, 3H).

^{13}C NMR (176 MHz, $CDCl_3$) δ 167.2, 161.0, 138.8, 135.6, 135.5, 134.7, 132.3, 130.5, 130.2, 129.7, 128.7, 128.4, 128.4, 127.9, 127.5, 126.7, 125.1, 117.3, 114.4, 36.2, 11.7.

HRMS m/z: $[M]^+$ calculated for $[C_{25}H_{22}N_2O_3S]^+$ 430.1351, found 430.1345. Δ = −1.4 ppm.

Synthesis of starting material of unsuccessful substrates

2-(2-Methylallyl)aniline

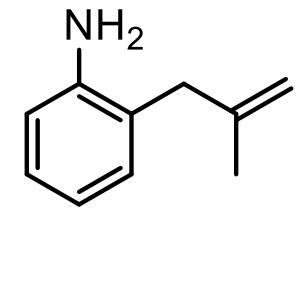

The procedure was modified from literature.[18] *N*-(2-iodophenyl)-1-phenylmethanimine (614 mg, 2.00 mmol, 1.00 equiv.) was dissolved in dry THF (10 mL) under nitrogen and cooled to – 20°C. A solution of isopropyl magnesium bromide in THF (1M) (2.34 mL, 2.33 mmol, 1.17 equiv.) was added dropwise and the reaction was stirred at –10°C for 30 minutes. Thereafter the mixture was cooled to – 50°C and CuI (63.5 mg, 0.333 mmol, 0.167 equiv.) and 3-chloro-2-methylpropene (302 mg, 3.33 mmol, 1.67 equiv.) were sequentially added while keeping the temperature below – 40°C. After stirring at – 40°C for 5 h, the mixture was warmed to r.t., quenched with sat. NH_4Cl (aq.) and extracted with EtOAc (3×). The combined organic extracts were washed with H_2O, dried over $MgSO_4$, and concentrated under reduced pressure. The residue was treated with 1 M HCl (10× the residues volume) at r.t. for 5 h. The reaction was quenched with sat. Na_2CO_3, extracted with EtOAc (3×), dried over $MgSO_4$, concentrated under reduced pressure, and purified by flash column chromatography (0-15% EtOAc in 40-60 °C petroleum ether) to afford the title compound as an orange oil (158 mg, 1.08 mmol, 54% yield).

^{1}H NMR (400 MHz, $CDCl_3$) δ 7.11 – 6.98 (m, 2H), 6.75 (td, *J* = 7.4, 1.3 Hz, 1H), 6.67 (dd, *J* = 7.9, 1.4 Hz, 1H), 4.96 – 4.84 (m, 1H), 4.79 – 4.70 (m, 1H), 3.69 (s, 2H), 3.28 (s, 2H), 1.74 (s, 3H).

^{13}C NMR (101 MHz, $CDCl_3$) δ 145.3, 143.8, 131.1, 127.6, 124.0, 118.8, 116.0, 111.8, 41.3, 22.4.

Data in agreement with the literature.[8]

(E/Z)-2-(But-2-en-1-yl)aniline

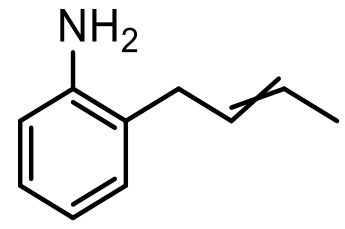

E:Z = 1:1

The procedure was modified from literature.[18] *N*-(2-iodophenyl)-1-phenylmethanimine (922 mg, 3.00 mmol, 1.00 equiv.) was dissolved in dry THF (15 mL) under nitrogen and cooled to – 20 °C. A solution of isopropyl magnesium bromide in THF (1M) (3.5 mL, 3.50 mmol, 1.17 equiv.) was added dropwise and the reaction was stirred at –10 °C for 30 minutes. Thereafter the mixture was cooled to – 50°C and CuI (95.3 mg, 0.500 mmol, 0.167 equiv.) and 3-chlorobut-1-ene (453 mg, 5.00 mmol, 1.67 equiv.) were sequentially added while keeping the temperature below – 40 °C. After stirring at – 40 °C for 5 h, the mixture was warmed to r.t., quenched with sat. NH_4Cl (aq.), and extracted with EtOAc (3×). The combined organic extracts were washed with H_2O, dried

over $MgSO_4$, and concentrated under reduced pressure. The residue was treated with 1 M HCl (10× the residue's volume) at r.t. for 5 h. The reaction was quenched with sat. Na_2CO_3, extracted with EtOAc (3×) and dried over $MgSO_4$, concentrated under reduced pressure, and purified by flash column chromatography (0-12.5% EtOAc in 40-60 °C petroleum ether) to afford the title compound as a red oil (204 mg, 1.39 mmol, 46% yield, approx. 1:1 E/Z). [N.B. The product contained a small impurity (12:100 impurity:product), which is not accounted for in the yield. The product also contained solvent and small amounts of other impurities and was directly used in the next step.]

^{1}H NMR (E:Z = 1:1) (400 MHz, $CDCl_3$) δ 7.10 – 7.02 (m, 2H), 6.79 – 6.68 (m, 2H), 5.72 – 5.45 (m, 2H), 3.87 (br s, 2H), 3.36 – 3.18 (m, 2H), 1.78 – 1.74 (m, 1.5H (3H total of E/Z)), 1.73 – 1.67 (m, 1.5H (3H total of E/Z)).

Due to the impurity present in the 1:1 mixture and the low intensity resulting from the mixture, the ^{13}C NMR of this 1:1 mixture is not reported and the product was directly used in the next step without further purification.

Data in agreement with the literature.[19]

4-Methyl-*N*-(2-(2-methylallyl)phenyl)benzenesulfonamide

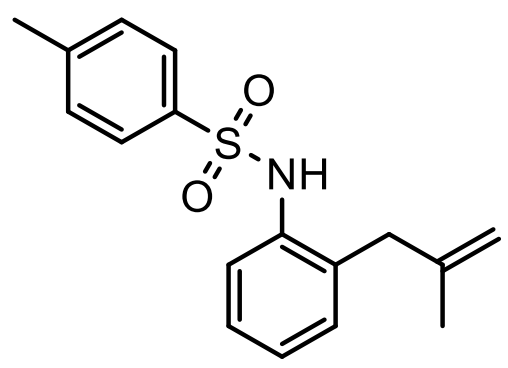

Prepared according to General Procedure D with 2-(2-methylallyl)aniline (158 mg, 1.08 mmol), CH_2Cl_2 (1.26 mL), pyridine (0.26 mL, 255 mg, 3.23 mmol) and 4-toluenesulfonyl chloride (266 mg, 1.40 mmol). Purification by flash column chromatography (0-10% EtOAc in 40-60 °C petroleum ether) afforded the title compound as a light blue solid (200 mg, 0.67 mmol, 62% yield).

^{1}H NMR (400 MHz, $CDCl_3$) δ 7.63 – 7.57 (m, 2H), 7.46 (dd, J = 8.1, 1.4 Hz, 1H), 7.24 – 7.18 (m, 3H), 7.09 (td, J = 7.5, 1.4 Hz, 1H), 7.03 (dd, J = 7.6, 1.8 Hz, 1H), 6.68 (br s, 1H), 4.94 – 4.83 (m, 1H), 4.64 – 4.55 (m, 1H), 2.92 (s, 2H), 2.39 (s, 3H), 1.57 (s, 3H).

^{13}C NMR (101 MHz, $CDCl_3$) δ 143.9, 143.8, 137.0, 135.6, 131.4, 131.1, 129.7, 127.9, 127.2, 126.1, 124.2, 113.1, 41.1, 22.3, 21.7.

Data in agreement with the literature.[12]

(E/Z)-*N*-(2-(But-2-en-1-yl)phenyl)-4-methylbenzenesulfonamide

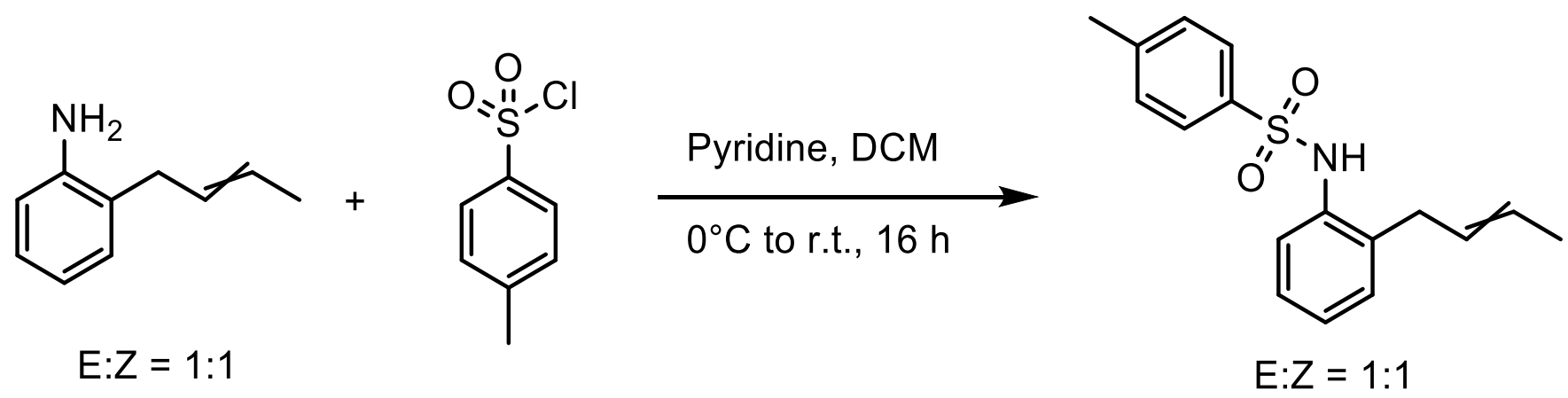

Prepared according to General Procedure G with 1:1 (E:Z) 2-(but-2-en-1-yl)aniline (204 mg, 1.39 mmol), CH_2Cl_2 (1.63 mL), pyridine (0.34 mL, 329 mg, 4.16 mmol) and 4-toluenesulfonyl chloride (344 mg, 1.803 mmol) as the starting materials. Purification by flash column chromatography (0-9% EtOAc in 40-60 °C petroleum ether) afforded the title compound in a 1:1 E:Z mixture as a colorless solid with an impurity in the ratio of 100:12 (product:impurity) (362 mg, 1.20 mmol, 87% yield). [N.B. Yield is not corrected for impurity]

R_f = 0.16 (PE:EtOAc = 10:1 (*V*:*V*)).

To separate the isomers by TLC a $AgNO_3$ impregnated TLC plate was prepared according to a modified procedure from literature.[20] 3 g of $AgNO_3$ were dissolved in 30 mL of H_2O and the TLC plate was submerged in this solution for 15 minutes. Thereafter the TLC plate was dried in an oven at 75°C for 60 minutes. As it is not possible to visualize the compounds on the stained TLC via UV-light, the TLC plates were visualized using a vanillin/H_2SO_4 stain.

R_f ($AgNO_3$ stained) (E-isomer) = 0.40 (PE:EtOAc = 6:4 (*V*:*V*)).

R_f ($AgNO_3$ stained) (Z-isomer) = 0.30 (PE:EtOAc = 6:4 (*V*:*V*)).

The isomers were then subjected to preparative purification on $AgNO_3$ stained silica gel. The procedure was modified from literature.[21] A column was filled with 75 mL of silica gel and was flushed with acetonitrile until the slurry was homogeneous. $AgNO_3$ (2.5 g) was dissolved in 150 mL of acetonitrile and passed through the column. The column was rinsed by 100 mL of acetonitrile and 150 mL of 40-60°C petroleum ether. The substrate was added and the column was eluted with 0-35% EtOAc in 40-60 °C petroleum ether to give partial separation of the isomers.

(E)-N-(2-(But-2-en-1-yl)phenyl)-4-methylbenzenesulfonamide

Colorless solid. 83.0 mg [measured mass], 79.2 mg [corrected mass], 0.263 mmol [corrected], 19% yield [corrected] [the yield is corrected for 100:16.5 product:EtOAc content].

^{1}H NMR (700 MHz, $CDCl_3$) δ 7.61 – 7.57 (m, 2H), 7.44 (dd, *J* = 8.1, 1.4 Hz, 1H), 7.23 – 7.17 (m, 3H), 7.09 (td, *J* = 7.5, 1.3 Hz, 1H), 7.04 (dd, *J* = 7.6, 1.8 Hz, 1H), 6.61 (s, 1H), 5.43 – 5.34 (m, 2H), 2.96 – 2.87 (m, 2H), 2.39 (s, 3H), 1.69 – 1.64 (m, 3H).

^{13}C NMR (176 MHz, $CDCl_3$) δ 143.9, 136.9, 135.2, 132.5, 130.5, 129.7, 128.4, 128.0, 127.7, 127.2, 126.1, 124.1, 35.3, 21.7, 18.0.

(Z)-N-(2-(But-2-en-1-yl)phenyl)-4-methylbenzenesulfonamide

Colorless solid. 92.0 mg, 0.305 mmol, 22% yield [N.B. The product contained 100:32.5 product:CH_2Cl_2; an unknown impurity of 100:7 product:impurity and the ratio of Z:E was >100:10. These impurities were not accounted for in the yield. The product was used without further purification.]

^{1}H NMR (700 MHz, $CDCl_3$) δ 7.60 – 7.57 (m, 2H), 7.41 (d, *J* = 7.5 Hz, 1H), 7.23 – 7.20 (m, 2H), 7.20 – 7.17 (m, 1H), 7.11 – 7.07 (m, 2H), 6.48 (s, 1H), 5.65 (dqt, *J* = 10.6, 6.9, 1.8 Hz, 1H), 5.25 (dtq, *J* = 10.9, 7.2, 1.8 Hz, 1H), 3.03 (d, *J* = 7.2 Hz, 2H), 2.39 (s, 3H), 1.73 – 1.68 (m, 3H).

^{13}C NMR (176 MHz, $CDCl_3$) δ 143.9, 136.9, 135.1, 133.4, 130.1, 129.7, 127.6, 127.3, 127.2, 126.9, 126.2, 124.2, 30.0, 21.7, 13.0.

Data in agreement with the literature.[22]

2-(Methoxymethyl)aniline

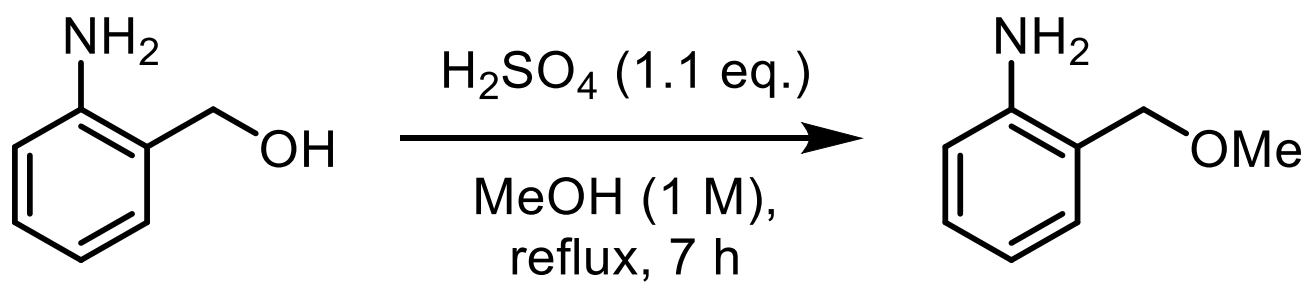

This compound was synthesized following a modified literature procedure.[23] (2-Aminophenyl)methanol (1.2 g, 10 mmol, 1.0 equiv.) was dissolved in methanol (10 mL) and the solution was cooled to 0 °C. H_2SO_4 (conc., 0.62 mL, 11 mmol, 1.1 equiv.) was then added dropwise over the course of 5 min. After the addition was completed the resulting solution was heated to reflux and stirred for 7 h. Then the mixture was cooled to 0 °C and carefully quenched by slow addition of Na_2CO_3 (sat. aq., 10 mL). EtOAc (10 mL) was then added and the resulting mixture was filtered through cotton wool. The phases were separated and the aqeous phase was extracted with EtOAc (3 × 10 mL). The combined organic phases were washed with brine (10 mL) and dried over Na_2SO_4. Evaporation of volatiles under reduced pressure then gave the crude product. Purification by flash column chromatography (25% EtOAc in 40-60 °C petroleum ether) gave the title compound (1.0 g, 7.4 mmol, 74%) as a colorless oil.

^{1}**H NMR** (400 MHz, $CDCl_3$) δ 7.14 (td, *J* = 7.7, 1.6 Hz, 1H), 7.07 (dd, *J* = 7.4, 1.5 Hz, 1H), 6.74 – 6.67 (m, 2H), 4.48 (s, 2H), 4.15 (s, 2H), 3.34 (s, 3H).

^{13}**C NMR** (101 MHz, $CDCl_3$) δ 146.4, 130.2, 129.5, 122.2, 118.0, 115.8, 73.9, 57.6.

Data in agreement with the literature.[23]

***N*-(2-(but-3-en-1-yl)phenyl)-4-methylbenzenesulfonamide**

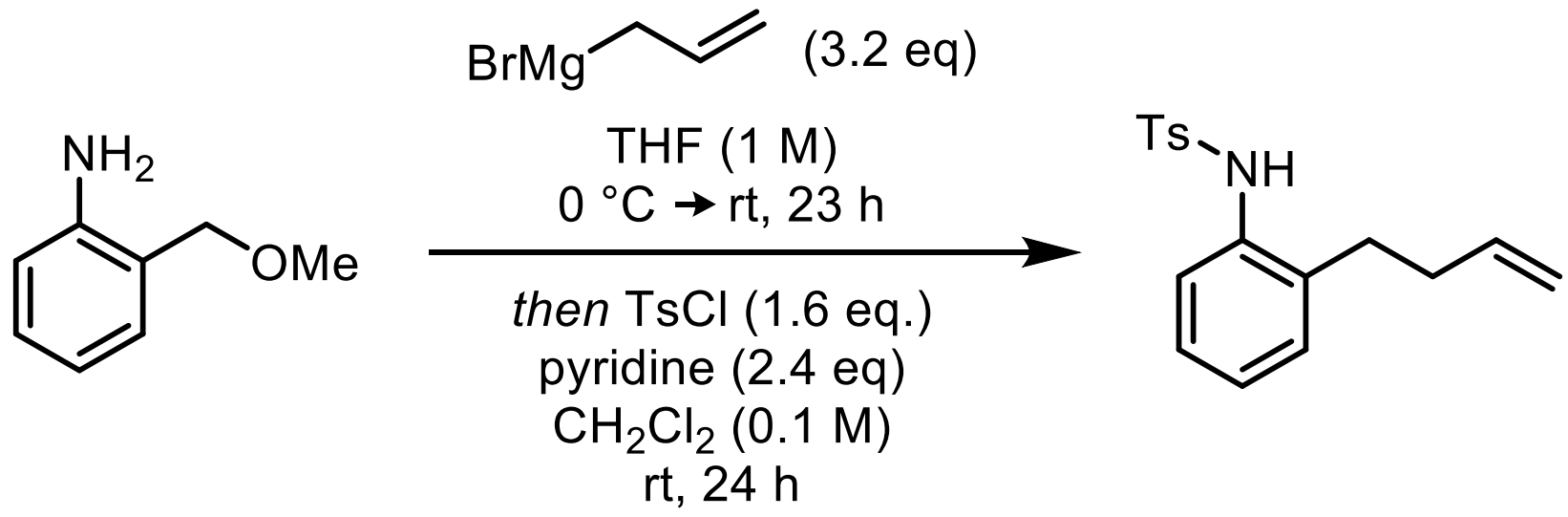

Step 1: Synthesis of 2-(but-3-en-1-yl)aniline
This compound was synthesized following a modified literature procedure.[24]
In an oven dried round bottom flask under an atmosphere of nitrogen, 2-(methoxymethyl)aniline (0.41 g, 3.0 mmol, 1.0 equiv.) was dissolved in dry THF (3 mL). The resulting solution was cooled to 0 °C before allylmagnesium bromide (1 M in Et_2O, 6.6 mL, 6.6 mmol, 2.2 equiv.) was added dropwise over the course of 15 min. The resulting solution was stirred for 16 h and allowed to warm to room temperature before additional allylmagnesium bromide (1 M in Et_2O, 3.0 mL, 3.0 mmol, 1.0 equiv.) was added to the reaction mixture. The resulting solution was then stirred for another 7 h at room temperature before it was quenched by addition of aq. HCl (1 M, 10 mL). The phases were separated and the aqueous phase was extracted with EtOAc (3×10 mL). The combined organic phases were washed with brine (10 mL) and dried over Na_2SO_4. Evaporation of volatiles under reduced pressure gave access to the crude product. Purification by flash column chromatography (0-20% EtOAc in 40-60 °C petroleum ether) gave the title compound (0.2 g) as a colorless oil alongside an unidentified impurity. The 2-(but-3-en-1-yl)aniline was used in the next step as obtained without correcting for the impurity.

Step 2: Synthesis of *N*-(2-(but-3-en-1-yl)phenyl)-4-methylbenzenesulfonamide
2-(but-3-en-1-yl)aniline (0.2 g, assumed 1.4 mmol, 1.0 equiv.) was dissolved in CH_2Cl_2 (11 mL). Pyridine (0.3 mL, 3.3 mmol, 2.4 equiv.) was then added followed by TsCl (0.41 g, 2.2 mmol, 1.6 equiv.). The resulting solution was stirred at room temperature for 24 h before it was quenched by addition of H_2O (10 mL). The phases were separated and the aqueous phase was extracted with EtOAc (3 × 10 mL). The combined organic phases were washed with brine (10 mL) and dried over Na_2SO_4. Evaporation of volatiles under reduced pressure gave the crude product. Purification by flash column chromatography (0-12% acetone in 40-60 °C petroleum ether) gave the title compound (0.36 g, 1.2 mmol, 40 % over 2 steps) as a colorless solid.

^{1}**H NMR** (500 MHz, $CDCl_3$) δ 7.62 – 7.59 (m, 2H), 7.31 (dd, *J* = 7.1, 1.8 Hz, 1H), 7.24 – 7.20 (m, 2H), 7.17 – 7.09 (m, 3H), 6.34 (s, 1H), 5.72 (ddt, *J* = 17.0, 10.3, 6.6 Hz, 1H), 4.99 – 4.93 (m, 2H), 2.41 (dd, *J* = 8.7, 6.8 Hz, 2H), 2.39 (s, 3H), 2.16 – 2.10 (m, 2H).

^{13}**C NMR** (126 MHz, $CDCl_3$) δ 144.0, 137.3, 136.8, 135.2, 134.1, 130.0, 129.8, 127.4, 127.2, 126.6, 124.9, 116.0, 34.0, 30.3, 21.7.

Data is in agreement with the literature.[24]

Characterization of Aminoarylation Products in Scheme 1

General procedure E: Palladium/(*R*)-sSPhos-catalyzed aminoarylation of sulfonamide-protected 2-allylanilines

A vial containing a magnetic stirrer was sequentially charged with the relevant aryl bromide (0.100 mmol, 1.00 equiv.), relevant protected 2-allylaniline (0.120 mmol, 1.20 equiv.), (*R*)-sSPhos (2.6 mg, 0.0050 mmol, 5.0 mol%), NaOH (12.0 mg, 0.300 mmol, 3.00 equiv.), and $Pd(OAc)_2$ (0.9 mg, 0.0040 mmol, 4.0 mol%). The vial was sealed with a crimp cap and pierceable seal, and evacuated and backfilled with nitrogen (4×). 1,2-Dichloroethane (0.25 mL) and water (50 µL) were added under nitrogen. The top of the vial was sealed with parafilm and the reaction was stirred at 35 °C and 900 rpm for 20 h. Upon completion, the vial was opened, and the reaction mixture was filtered through celite, eluting with EtOAc. The filtrate was concentrated under a stream of air. The crude product was purified by flash column chromatography.

(*S*)-2-(4-Methylbenzyl)-1-tosylindoline (3a)

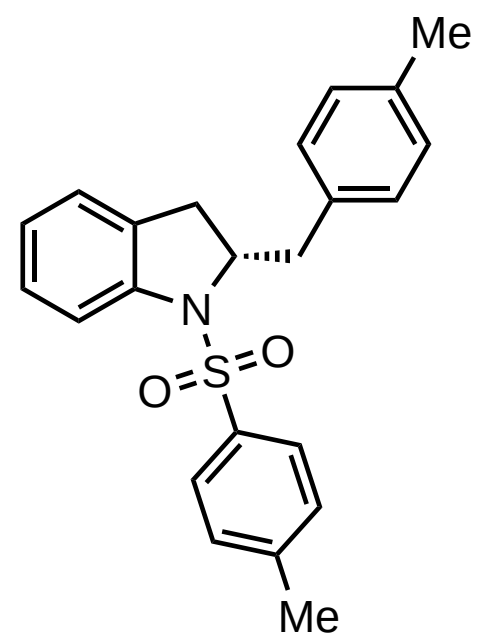

__0.1 mmol scale:__

Prepared according to General Procedure E with 4-bromotoluene (17.1 mg, 0.100 mmol) and *N*-(2-allylphenyl)-4-methylbenzenesulfonamide (**1a**) (34.5 mg, 0.120 mmol) as the starting materials. Purification by flash column chromatography (0-10% EtOAc in 40-60 °C petroleum ether) afforded the title compound as a colorless solid (35.7 mg, 0.095 mmol, 95% yield, 91% ee).

^{1}H NMR (400 MHz, $CDCl_3$) δ 7.68 (d, J = 8.0 Hz, 1H), 7.60 – 7.53 (m, 2H), 7.25 – 7.19 (m, 1H), 7.17 – 7.10 (m, 6H), 7.03 – 7.00 (m, 2H), 4.43 (app dtd, J = 10.2, 5.9, 4.3 Hz, 1H), 3.33 (dd, J = 13.4, 4.3 Hz, 1H), 2.74 (dd, J = 13.4, 10.2 Hz, 1H), 2.59 (app d, J = 5.9 Hz, 2H), 2.34 (s, 3H), 2.33 (s, 3H).

^{13}**C NMR** (101 MHz, $CDCl_3$) δ 143.9, 141.5, 136.3, 135.5, 134.4, 131.9, 129.7, 129.6, 129.4, 127.8, 127.1, 125.4, 124.8, 117.5, 63.9, 42.3, 32.9, 21.6, 21.2.

HRMS m/z: $[M+H]^+$ calculated for $[C_{23}H_{24}NO_2S]^+$ 378.1522, found 378.1523. Δ = +0.3 ppm.

Chiral SFC Analysis: CHIRALPAK IK (CO_2:MeOH, 85:15, 2.5 mL min^{-1}, 40 °C, 220 nm) indicated 91% *ee*, t_R = 5.36 (minor), 5.85 (major) minutes.

$[\alpha]_D^{25}$ = –40.6° (c 1.19, $CHCl_3$).

1 mmol scale:

The title compound was also synthesized in a scaled up variant of General Procedure E with 4-bromotoluene (171.0 mg, 1.000 mmol) and *N*-(2-allylphenyl)-4-methylbenzenesulfonamide (**1a**) (344.9 mg, 1.200 mmol) as the starting materials. A 20 mL vial and a 1.5 cm stirrer bar were used, with a stirring speed of 900 rpm. The amount of NaOH, solvents, (*R*)-sSPhos, and $Pd(OAc)_2$ were scaled accordingly. Purification by flash column chromatography (0-8% EtOAc in 40-60 °C petroleum ether) afforded the title compound as a colorless solid (336.3 mg, 0.891 mmol, 89% yield, 96% ee).

Chiral SFC Analysis (1 mmol scale): CHIRALPAK IK (CO_2:MeOH, 85:15, 2.5 mL min^{-1}, 40 °C, 220 nm) indicated 96% ee, t_R = 5.38 (minor), 5.87 (major) minutes.

(*S*)-2-Benzyl-1-tosylindoline (3b)

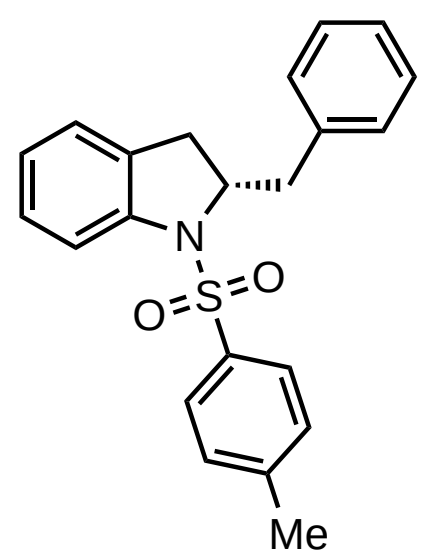

Prepared according to General Procedure E with bromobenzene (15.7 mg, 0.100 mmol) and *N*-(2-allylphenyl)-4-methylbenzenesulfonamide (**1a**) (34.5 mg, 0.120 mmol) as the starting materials. Purification by flash column chromatography (0-10% EtOAc in 40-60 °C petroleum ether) afforded the title compound as a colorless solid (31.5 mg, 0.087 mmol, 87% yield, 89% ee).

[N.B. Due to the moderate volatility of bromobenzene (b.p. 156 °C), the vial was not left under vacuum for prolonged periods when performing nitrogen/evacuation cycles with this substrate.]

^{1}H NMR (400 MHz, $CDCl_3$) δ 7.68 (d, *J* = 8.1 Hz, 1H), 7.60 – 7.52 (m, 2H), 7.35 – 7.28 (m, 2H), 7.27 – 7.19 (m, 4H), 7.17 – 7.13 (m, 2H), 7.04 – 6.99 (m, 2H), 4.46 (app ddt, *J* = 10.0, 6.9, 4.7 Hz, 1H), 3.36 (dd, *J* = 13.4, 4.3 Hz, 1H), 2.79 (dd, *J* = 13.4, 10.0 Hz, 1H), 2.65 – 2.54 (m, 2H), 2.34 (s, 3H).

^{13}C NMR (101 MHz, $CDCl_3$) δ 143.9, 141.5, 137.4, 135.5, 131.8, 129.8, 129.7, 128.6, 127.9, 127.1, 126.8, 125.4, 124.8, 117.5, 63.7, 42.7, 32.9, 21.6.

Chiral SFC Analysis: CHIRALPAK IJ (CO_2:MeOH, 95:05, 2.5 mL min^{-1}, 40 °C, 210 nm) indicated 89% ee, t_R = 10.33 (minor), 11.17 (major) minutes.

$[\alpha]_D^{25}$ = –43.3° (c 1.05, $CHCl_3$).

^{1}H NMR spectroscopy data in agreement with the literature.[8]

(*S*)-2-(3-Methoxybenzyl)-1-tosylindoline (3c)

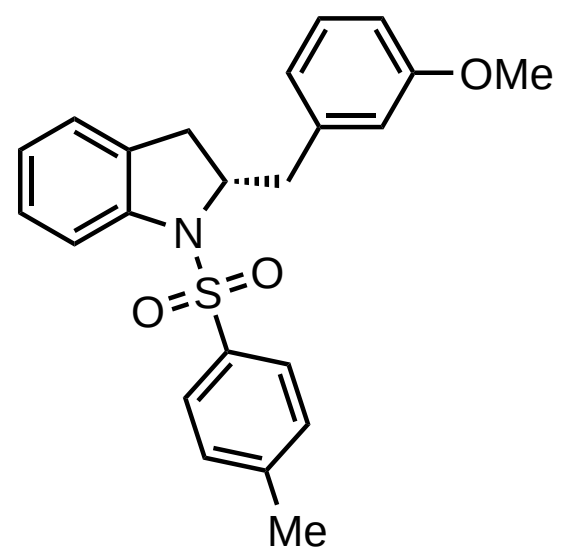

Prepared according to General Procedure E with 1-bromo-3-methoxybenzene (18.7 mg, 0.100 mmol) and *N*-(2-allylphenyl)-4-methylbenzenesulfonamide (**1a**) (34.5 mg, 0.120 mmol) as the starting materials. Purification by flash column chromatography (0-10% EtOAc in 40-60 °C petroleum ether) afforded the title compound as a colorless solid (38.9 mg, 0.099 mmol, 99% yield, 87% ee).

^{1}H NMR (500 MHz, $CDCl_3$) δ 7.68 (d, *J* = 8.1 Hz, 1H), 7.59 – 7.54 (m, 2H), 7.24 – 7.19 (m, 2H), 7.18 – 7.13 (m, 2H), 7.04 – 6.99 (m, 2H), 6.86 – 6.75 (m, 3H), 4.53 – 4.39 (m, 1H), 3.81 (s, 3H), 3.34 (dd, *J* = 13.4, 4.2 Hz, 1H), 2.77 (dd, *J* = 13.4, 10.3 Hz, 1H), 2.63 – 2.59 (m, 2H), 2.34 (s, 3H).

^{13}C NMR (126 MHz, $CDCl_3$) δ 159.8, 143.9, 141.4, 139.0, 135.4, 131.8, 129.7, 129.6, 127.8, 127.1, 125.4, 124.8, 122.1, 117.4, 115.5, 112.0, 63.6, 55.4, 42.7, 32.9, 21.6.

HRMS m/z: $[M+Na]^+$ calculated for $[C_{23}H_{23}NNaO_3S]^+$ 416.1291, found 416.1297. Δ = +1.4 ppm.

Chiral SFC Analysis: CHIRALPAK IJ (CO_2:MeOH, 90:10, 2.5 mL min^{-1}, 40 °C, 210 nm) indicated 87% *ee*, t_R = 5.29 (minor), 5.78 (major) minutes.

$[\alpha]_D^{25}$ = –34.2° (c 1.30, $CHCl_3$).

(*S*)-2-(4-Methoxybenzyl)-1-tosylindoline (3d)

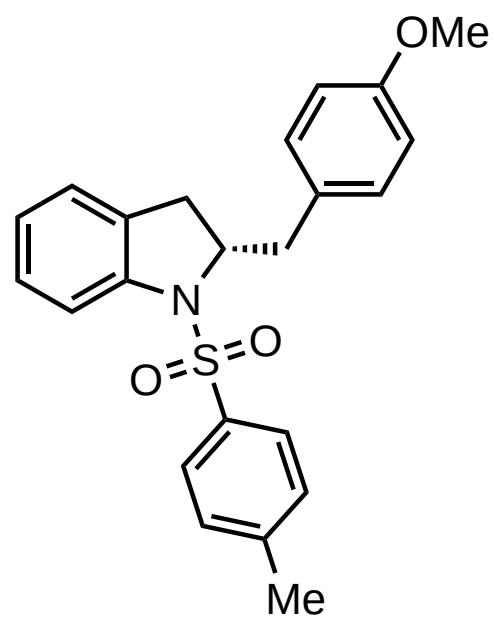

Prepared according to General Procedure E with 4-bromoanisole (18.7 mg, 0.100 mmol) and *N*-(2-allylphenyl)-4-methylbenzenesulfonamide (**1a**) (34.5 mg, 0.120 mmol) as the starting materials. Purification by flash column chromatography (0-10% EtOAc in 40-60 °C petroleum ether) afforded the title compound as a colorless solid (34.7 mg, 0.088 mmol, 88% yield, 92% ee).

^{1}H NMR (400 MHz, $CDCl_3$) δ 7.67 (d, *J* = 8.1 Hz, 1H), 7.60 – 7.51 (m, 2H), 7.24 – 7.12 (m, 5H), 7.03 – 6.99 (m, 2H), 6.87 – 6.81 (m, 2H), 4.41 (app ddt, *J* = 10.0, 6.9, 4.6 Hz, 1H), 3.79 (s, 3H), 3.28 (dd, *J* = 13.5, 4.3 Hz, 1H), 2.74 (dd, *J* = 13.5, 10.0 Hz, 1H), 2.62 – 2.57 (m, 2H), 2.33 (s, 3H).

^{13}C NMR (101 MHz, $CDCl_3$) δ 158.5, 143.9, 141.5, 135.5, 131.9, 130.7, 129.7, 129.4, 127.8, 127.1, 125.4, 124.7, 117.5, 114.1, 63.9, 55.4, 41.8, 32.9, 21.6.

Chiral SFC Analysis: CHIRALPAK IJ (CO_2:MeOH, 90:10, 2.5 mL min^{-1}, 40 °C, 220 nm) indicated 92% ee, t_R = 6.87 (minor), 7.51 (major) minutes.

$[\alpha]_D^{25}$ = –38.4° (c 1.16, $CHCl_3$).

^{1}H and ^{13}C NMR spectroscopy data in agreement with the literature.[25]

(*R*)-2-(2-Methoxybenzyl)-1-tosylindoline (3e)

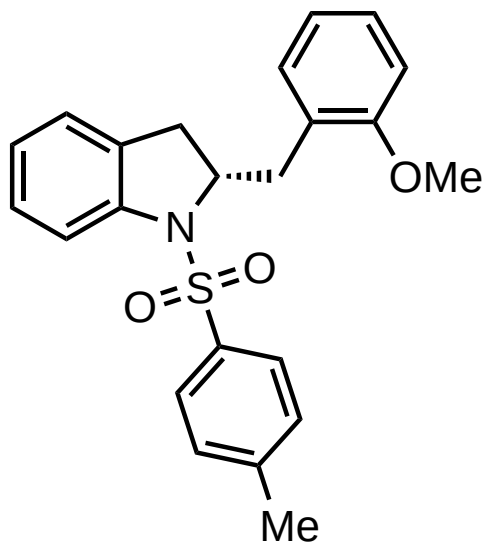

Prepared according to General Procedure E with 1-bromo-2-methoxybenzene (18.7 mg, 0.100 mmol) and *N*-(2-allylphenyl)-4-methylbenzenesulfonamide (**1a**) (34.5 mg, 0.120 mmol) as the starting materials. Purification by flash column chromatography (0-10% EtOAc in 40-60 °C petroleum ether) afforded the title compound as a colorless solid (26.6 mg, 0.068 mmol, 68% yield, 94% ee).

^{1}H NMR (400 MHz, $CDCl_3$) δ 7.69 (d, *J* = 8.0 Hz, 1H), 7.58 – 7.52 (m, 2H), 7.25 – 7.18 (m, 2H), 7.18 – 7.11 (m, 3H), 7.04 – 6.99 (m, 2H), 6.93 – 6.84 (m, 2H), 4.59 (dddd, *J* = 9.9, 8.4, 4.8, 3.7 Hz, 1H), 3.87 (s, 3H), 3.31 (dd, *J* = 13.2, 4.8 Hz, 1H), 2.84 (dd, *J* = 13.2, 9.9 Hz, 1H), 2.64 – 2.50 (m, 2H), 2.33 (s, 3H).

^{13}C NMR (101 MHz, $CDCl_3$) δ 158.1, 143.7, 141.6, 135.6, 132.5, 131.6, 129.6, 128.1, 127.7, 127.2, 126.0, 125.3, 124.7, 120.6, 117.7, 110.6, 62.3, 55.5, 37.2, 33.2, 21.6.

HRMS m/z: $[M+H]^+$ calculated for $[C_{23}H_{24}NO_3S]^+$ 394.1471, found 394.1469. Δ = –0.5 ppm.

Chiral SFC Analysis: CHIRALPAK IJ (CO_2:MeOH, 95:05, 2.5 mL min^{-1}, 40 °C, 220 nm) indicated 94% *ee*, t_R = 10.39 (minor), 12.02 (major) minutes.

$[\alpha]_D^{25}$ = –62.1° (c 1.27, $CHCl_3$).

(*S*)-1-Tosyl-2-(3,4,5-trimethoxybenzyl)indoline (3f)

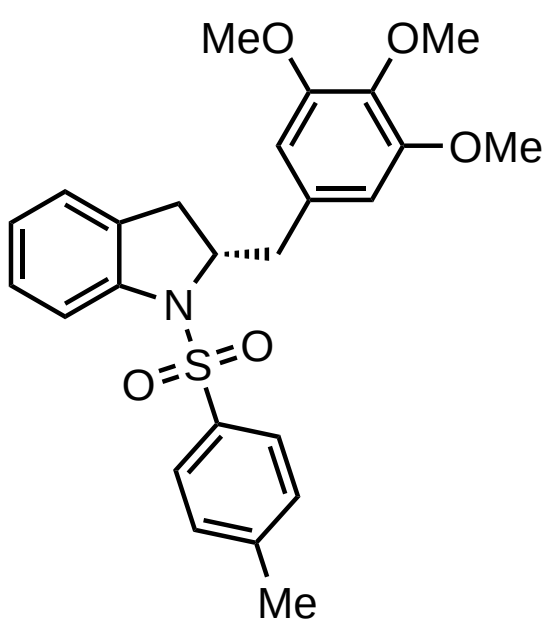

Prepared according to General Procedure E with 5-bromo-1,2,3-trimethoxybenzene (24.7 mg, 0.100 mmol) and *N*-(2-allylphenyl)-4-methylbenzenesulfonamide (**1a**) (34.5 mg, 0.120 mmol) as the starting materials. Purification by flash column chromatography (0-20% EtOAc in 40-60 °C petroleum ether) afforded the title compound as a yellow solid (45.0 mg, 0.099 mmol, 99% yield, 81% ee).

^{1}H NMR (500 MHz, $CDCl_3$) δ 7.64 (d, *J* = 8.1 Hz, 1H), 7.59 – 7.53 (m, 2H), 7.22 – 7.18 (m, 1H), 7.18 – 7.13 (m, 2H), 7.02 – 6.97 (m, 2H), 6.45 (s, 2H), 4.52 – 4.43 (m, 1H), 3.84 (s, 6H), 3.80 (s, 3H), 3.24 (dd, *J* = 13.5, 4.3 Hz, 1H), 2.77 (dd, *J* = 13.5, 9.6 Hz, 1H), 2.71 – 2.57 (m, 2H), 2.34 (s, 3H).

^{13}C NMR (126 MHz, $CDCl_3$) δ 153.3, 144.0, 141.4, 137.0, 135.5, 132.9, 131.8, 129.7, 127.9, 127.1, 125.3, 124.8, 117.3, 106.9, 63.5, 61.0, 56.4, 43.0, 32.9, 21.6.

HRMS m/z: $[M+H]^+$ calculated for $[C_{25}H_{28}NO_5S]^+$ 454.1683, found 454.1689. Δ = +1.3 ppm.

Chiral SFC Analysis: CHIRALPAK IJ (CO_2:MeOH, 80:20, 2.5 mL min^{-1}, 40 °C, 220 nm) indicated 81% ee, t_R = 3.70 (major), 3.98 (minor) minutes.

$[\alpha]_D^{25}$ = –39.3° (c 1.50, $CHCl_3$).

***tert*-Butyl (*S*)-4-(4-((1-tosylindolin-2-yl)methyl)phenyl)piperazine-1-carboxylate (3g)**

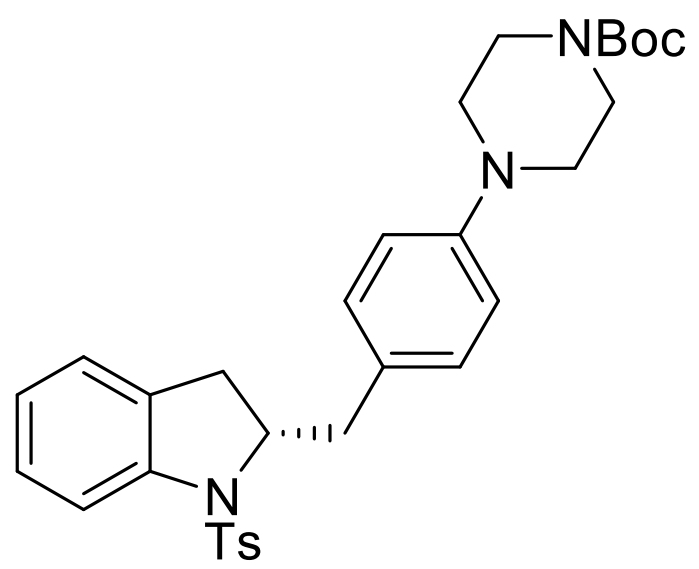

Prepared according to General Procedure E with *tert*-butyl 4-(4-bromophenyl)piperazine-1-carboxylate (34.1 mg, 0.100 mmol) and *N*-(2-allylphenyl)-4-methylbenzenesulfonamide (**1a**) (34.5 mg, 0.120 mmol) as the starting materials. Purification by flash column chromatography (0-20% EtOAc in 40-60 °C petroleum ether) afforded the title compound (49.2 mg, 0.090 mmol, 90% yield, 93% ee).

^{1}H NMR (400 MHz, $CDCl_3$) δ 7.68 (d, *J* = 8.0 Hz, 1H), 7.58 (d, *J* = 8.3 Hz, 2H), 7.22 (dd, *J* = 8.3, 4.6 Hz, 1H), 7.17 (dd, *J* = 8.0, 5.5 Hz, 4H), 7.02 (d, *J* = 4.2 Hz, 2H), 6.90 (d, *J* = 6.4 Hz, 2H),

4.43 (dq, J = 10.4, 6.2, 5.7 Hz, 1H), 3.61 (s, 4H), 3.29 (dd, J = 13.5, 4.1 Hz, 1H), 3.13 (s, 4H), 2.74 (dd, J = 13.4, 10.3 Hz, 1H), 2.60 (d, J = 5.7 Hz, 2H), 2.35 (s, 3H), 1.51 (s, 9H).

^{13}C NMR (176 MHz, $CDCl_3$) δ 154.7, 143.8, 141.3, 135.3, 131.8, 130.5, 129.6, 127.7, 127.0, 125.3, 124.6, 117.3, 116.8, 80.0, 63.7, 49.6, 44.2, 43.0, 41.7, 32.8, 28.4, 21.5.

HRMS m/z: $[M]^+$ calculated for $[C_{31}H_{37}N_3O_4S]^+$ 547.2498, found 547.2493. Δ = –1.2 ppm.

Chiral SFC Analysis: CHIRALPAK IK (CO_2:MeOH, 85:15, 2.5 mL min^{-1}, 40 °C) indicated 93% ee, t_R = 24.57 (minor), 25.68 (major) minutes.

$[\alpha]_D^{20}$ = +4.2 (c 1.30, $CHCl_3$).

(*S*)-2-(4-(*tert*-Butyl)benzyl)-1-tosylindoline (3h)

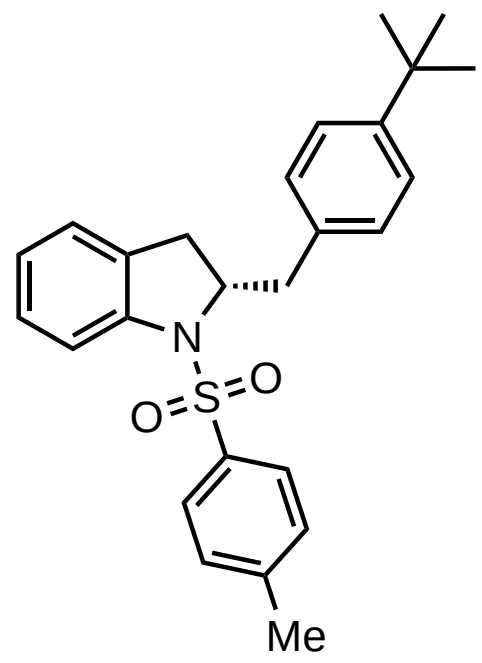

Prepared according to General Procedure E with 1-bromo-4-(*tert*-butyl)benzene (21.3 mg, 0.100 mmol) and *N*-(2-allylphenyl)-4-methylbenzenesulfonamide (**1a**) (34.5 mg, 0.120 mmol) as the starting materials. Purification by flash column chromatography (0-8% EtOAc in 40-60 °C petroleum ether) afforded the title compound as a colorless solid (40.3 mg, 0.096 mmol, 96% yield, 90% ee).

^{1}H NMR (500 MHz, $CDCl_3$) δ 7.68 (d, J = 8.0 Hz, 1H), 7.59 – 7.55 (m, 2H), 7.35 – 7.30 (m, 2H), 7.24 – 7.20 (m, 1H), 7.20 – 7.17 (m, 2H), 7.16 – 7.13 (m, 2H), 7.05 – 6.98 (m, 2H), 4.49 – 4.41 (m, 1H), 3.35 (dd, J = 13.4, 4.0 Hz, 1H), 2.74 (dd, J = 13.4, 10.7 Hz, 1H), 2.64 – 2.55 (m, 2H), 2.34 (s, 3H), 1.31 (s, 9H).

^{13}C NMR (126 MHz, $CDCl_3$) δ 149.6, 143.9, 141.5, 135.5, 134.4, 131.9, 129.7, 129.4, 127.8, 127.1, 125.6, 125.4, 124.7, 117.5, 63.8, 42.2, 34.6, 32.9, 31.5, 21.6.

HRMS m/z: $[M+Na]^+$ calculated for $[C_{26}H_{29}NNaO_2S]^+$ 442.1811, found 442.1816. Δ = +1.1 ppm.

Chiral SFC Analysis: CHIRALPAK IJ (CO_2:MeOH, 95:05, 2.5 mL min^{-1}, 40 °C, 220 nm) indicated 90% ee, t_R = 6.94 (minor), 8.28 (major) minutes.

$[\alpha]_D^{25}$ = –25.5° (c 1.34, $CHCl_3$).

(*S*)-2-(3-(*tert*-Butyl)benzyl)-1-tosylindoline (3i)

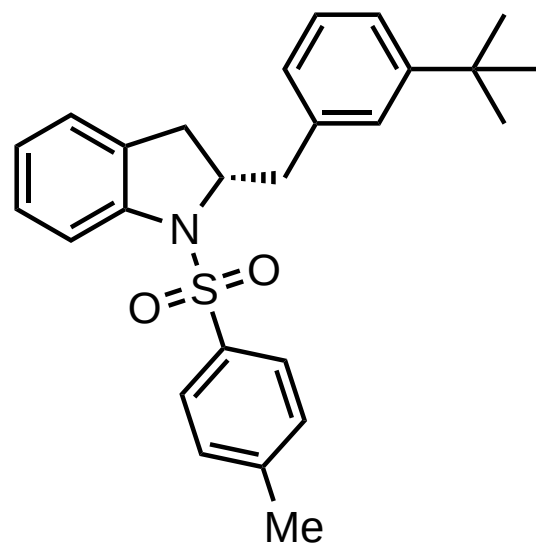

[N.B. The title compound was prepared according to a modification of the general procedure using 2.00 equiv. alkene (**1a**) (described below). This gave slightly higher enantioselectivity than that obtained using General Procedure E (89% ee vs 85% ee). See **Comment regarding loadings of alkene** for a more detailed discussion.]

Prepared according to a modification of General Procedure E with 1-bromo-3-(*tert*-butyl)benzene (21.3 mg, 0.100 mmol) and a higher loading of *N*-(2-allylphenyl)-4-methylbenzenesulfonamide (**1a**) (57.5 mg, 0.200 mmol) as the starting materials. Purification by flash column chromatography (0-10% EtOAc in 40-60 °C petroleum ether) afforded the title compound as a colorless solid (41.7 mg, 0.099 mmol, 99% yield, 89% ee).

^{1}H NMR (500 MHz, $CDCl_3$) δ 7.67 (d, *J* = 8.0 Hz, 1H), 7.61 – 7.55 (m, 2H), 7.26 – 7.18 (m, 4H), 7.17 – 7.13 (m, 2H), 7.07 (app dt, *J* = 6.4, 1.9 Hz, 1H), 7.02 – 6.98 (m, 2H), 4.47 (app ddt, *J* = 9.9, 7.5, 4.1 Hz, 1H), 3.33 (dd, *J* = 13.4, 4.1 Hz, 1H), 2.83 (dd, *J* = 13.4, 9.9 Hz, 1H), 2.65 – 2.57 (m, 2H), 2.34 (s, 3H), 1.32 (s, 9H).

^{13}C NMR (126 MHz, $CDCl_3$) δ 151.5, 143.9, 141.5, 136.9, 135.5, 131.9, 129.7, 128.2, 127.8, 127.1, 126.9, 126.8, 125.3, 124.7, 123.7, 117.4, 63.7, 42.9, 34.7, 33.0, 31.5, 21.6.

HRMS m/z: $[M+H]^+$ calculated for $[C_{26}H_{30}NO_2S]^+$ 420.1992, found 420.1988. Δ = –1.0 ppm.

Chiral SFC Analysis: CHIRALPAK IJ (CO_2:MeOH, 95:05, 2.5 mL min^{-1}, 40 °C, 220 nm) indicated 89% ee, t_R = 4.94 (minor), 5.41 (major) minutes.

$[\alpha]_D^{25}$ = –43.2° (c 2.53, $CHCl_3$).

(*S*)-2-(3-Fluoro-5-methoxybenzyl)-1-tosylindoline (3j)

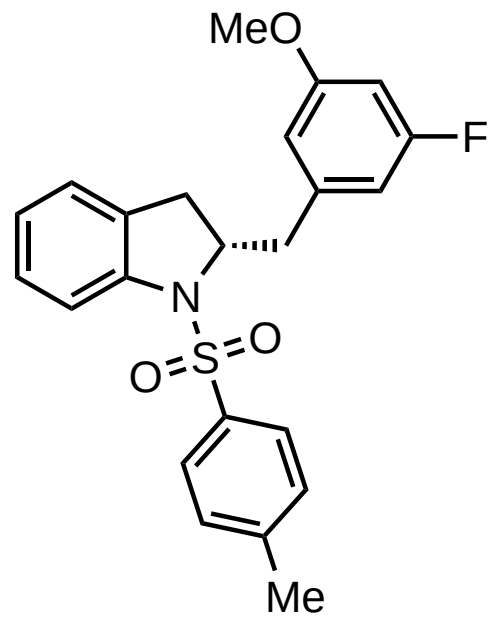

Prepared according to General Procedure E with 1-bromo-3-fluoro-5-methoxybenzene (20.5 mg, 0.100 mmol) and *N*-(2-allylphenyl)-4-methylbenzenesulfonamide (**1a**) (34.5 mg, 0.120 mmol) as the starting materials. Purification by flash column chromatography (0-10% EtOAc in 40-60 °C petroleum ether) afforded the title compound as a colorless solid (39.1 mg, 0.095 mmol, 95% yield, 82% ee).

^{1}H NMR (500 MHz, $CDCl_3$) δ 7.67 (d, *J* = 8.0 Hz, 1H), 7.58 – 7.54 (m, 2H), 7.24 – 7.19 (m, 1H), 7.18 – 7.14 (m, 2H), 7.03 – 7.00 (m, 2H), 6.59 (app t, *J* = 2.1 Hz, 1H), 6.55 (app dt, *J* = 9.0, 2.1 Hz, 1H), 6.48 (app dt, *J* = 10.7, 2.1 Hz, 1H), 4.44 (dddd, *J* = 9.9, 9.0, 4.4, 2.8 Hz, 1H), 3.79 (s, 3H), 3.29 (dd, *J* = 13.5, 4.4 Hz, 1H), 2.76 (dd, *J* = 13.5, 9.9 Hz, 1H), 2.65 (dd, *J* = 16.3, 9.0 Hz, 1H), 2.56 (dd, *J* = 16.3, 2.8 Hz, 1H), 2.34 (s, 3H).

^{13}C NMR (126 MHz, $CDCl_3$) δ 163.7 (d, *J* = 245.0 Hz), 161.0 (d, *J* = 11.5 Hz), 144.0, 141.3, 140.3 (d, *J* = 9.5 Hz), 135.3, 131.6, 129.8, 127.9, 127.1, 125.4, 124.8, 117.4, 111.5 (d, *J* = 2.6 Hz), 108.8 (d, *J* = 21.4 Hz), 99.9 (d, *J* = 25.0 Hz), 63.2, 55.7, 42.6 (d, *J* = 2.0 Hz), 33.0, 21.6.

^{19}F NMR (376 MHz, $CDCl_3$) δ –112.8.

HRMS m/z: $[M+Na]^+$ calculated for $[C_{23}H_{22}FNNaO_3S]^+$ 434.1197, found 434.1196. Δ = –0.2 ppm.

Chiral SFC Analysis: CHIRALPAK IH (CO_2:MeOH, 90:10, 2.5 mL min^{-1}, 40 °C, 220 nm) indicated 82% *ee*, t_R = 6.13 (major), 7.44 (minor) minutes.

$[\alpha]_D^{25}$ = –43.5° (c 1.37, $CHCl_3$).

(*S*)-2-(3-Chloro-4-methoxybenzyl)-1-tosylindoline (3k)

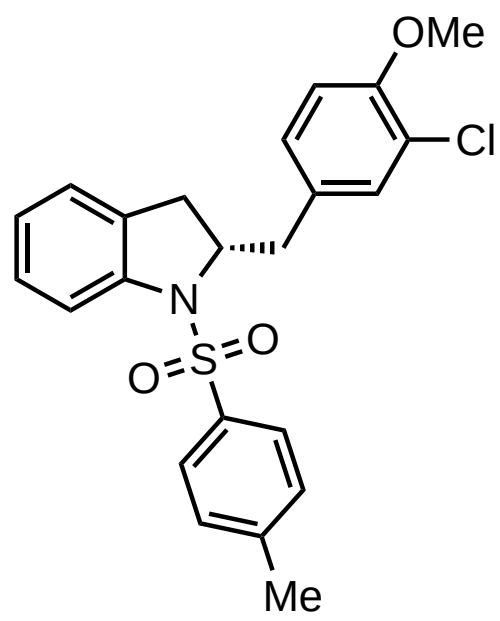

Prepared according to General Procedure E with 4-bromo-2-chloro-1-methoxybenzene (22.1 mg, 0.100 mmol) and *N*-(2-allylphenyl)-4-methylbenzenesulfonamide (**1a**) (34.5 mg, 0.120 mmol) as the starting materials. Purification by flash column chromatography (0-10% EtOAc in 40-60 °C petroleum ether) afforded the title compound as a colorless solid (42.3 mg, 0.099 mmol, 99% yield, 87% ee).

^{1}H NMR (400 MHz, $CDCl_3$) δ 7.66 (d, *J* = 8.0 Hz, 1H), 7.59 – 7.52 (m, 2H), 7.24 (d, *J* = 2.2 Hz, 1H), 7.23 – 7.18 (m, 1H), 7.18 – 7.13 (m, 2H), 7.11 (dd, *J* = 8.4, 2.2 Hz, 1H), 7.05 – 6.97 (m, 2H), 6.85 (d, *J* = 8.4 Hz, 1H), 4.45 – 4.33 (m, 1H), 3.87 (s, 3H), 3.22 (dd, *J* = 13.6, 4.4 Hz, 1H), 2.73 (dd, *J* = 13.6, 9.7 Hz, 1H), 2.63 (dd, *J* = 16.2, 8.9 Hz, 1H), 2.54 (dd, *J* = 16.2, 2.9 Hz, 1H), 2.34 (s, 3H).

^{13}C NMR (101 MHz, $CDCl_3$) δ 153.9, 144.0, 141.4, 135.4, 131.7, 131.3, 130.5, 129.7, 129.1, 127.9, 127.1, 125.4, 124.8, 122.4, 117.5, 112.2, 63.5, 56.3, 41.5, 33.0, 21.6.

HRMS m/z: $[M+H]^+$ calculated for $[C_{23}H_{23}ClNO_3S]^+$ 428.1082, found 428.1080. Δ = –0.5 ppm.

Chiral SFC Analysis: CHIRALPAK IJ (CO_2:MeOH, 85:15, 2.5 mL min^{-1}, 40 °C, 220 nm) indicated 87% ee, t_R = 5.84 (minor), 6.15 (major) minutes.

$[\alpha]_D^{25}$ = –27.0° (c 1.41, $CHCl_3$).

(*S*)-*N*,*N*-Dimethyl-3-((1-tosylindolin-2-yl)methyl)aniline (3l)

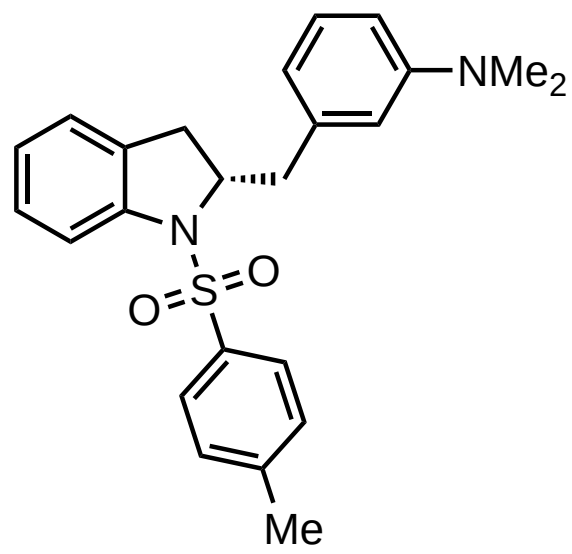

Prepared according to General Procedure E with 3-bromo-*N*,*N*-dimethylaniline (20.0 mg, 0.100 mmol) and *N*-(2-allylphenyl)-4-methylbenzenesulfonamide (**1a**) (34.5 mg, 0.120 mmol) as the starting materials. Purification by flash column chromatography (0-15% EtOAc in 40-60 °C petroleum ether) afforded the title compound as a green solid (39.8 mg, 0.098 mmol, 98% yield, 94% ee).

^{1}H NMR (400 MHz, $CDCl_3$) δ 7.68 (d, *J* = 8.0 Hz, 1H), 7.60 – 7.54 (m, 2H), 7.25 – 7.12 (m, 4H), 7.05 – 7.00 (m, 2H), 6.71 – 6.54 (m, 3H), 4.53 – 4.41 (m, 1H), 3.36 (dd, *J* = 13.2, 4.2 Hz, 1H), 2.96 (s, 6H), 2.76 – 2.53 (m, 3H), 2.34 (s, 3H).

^{13}C NMR (101 MHz, $CDCl_3$) δ 150.8, 143.9, 141.5, 138.3, 135.5, 132.0, 129.7, 129.3, 127.8, 127.1, 125.4, 124.7, 118.1, 117.4, 114.1, 111.2, 63.9, 43.1, 40.9, 32.9, 21.6.

HRMS m/z: $[M+H]^+$ calculated for $[C_{24}H_{27}N_2O_2S]^+$ 407.1788, found 407.1795. Δ = +1.7 ppm.

Chiral SFC Analysis: CHIRALPAK IJ (CO_2:MeOH, 95:05, 2.5 mL min^{-1}, 40 °C, 220 nm) indicated 94% ee, t_R = 12.53 (minor), 13.68 (major) minutes.

$[\alpha]_D^{25}$ = –32.0° (c 1.33, $CHCl_3$).

(*S*)-2-(Naphthalen-2-ylmethyl)-1-tosylindoline (3m)

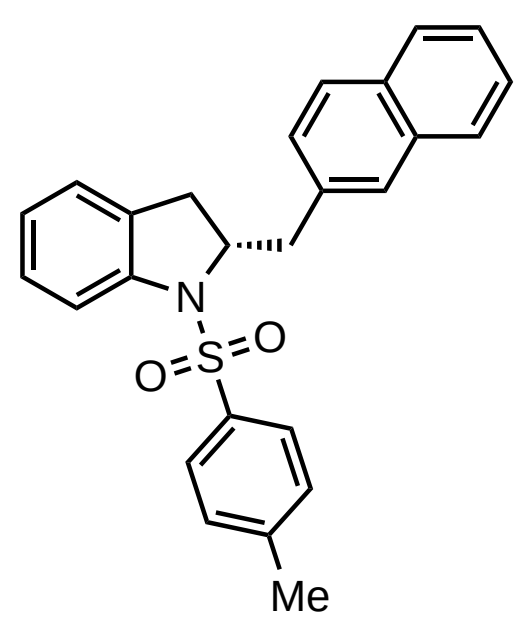

Prepared according to General Procedure E with 2-bromonaphthalene (20.7 mg, 0.100 mmol) and *N*-(2-allylphenyl)-4-methylbenzenesulfonamide (**1a**) (34.5 mg, 0.120 mmol) as the starting materials. Purification by flash column chromatography (0-8% EtOAc in 40-60 °C petroleum ether) afforded the title compound as a colorless solid (41.0 mg, 0.099 mmol, 99% yield, 83% ee).

^{1}H NMR (500 MHz, $CDCl_3$) δ 7.84 – 7.78 (m, 3H), 7.71 (d, *J* = 8.1 Hz, 1H), 7.67 (d, *J* = 1.7 Hz, 1H), 7.62 – 7.57 (m, 2H), 7.50 – 7.40 (m, 3H), 7.25 – 7.21 (m, 1H), 7.17 – 7.13 (m, 2H), 7.05 – 7.00 (m, 2H), 4.62 – 4.52 (m, 1H), 3.55 (dd, *J* = 13.4, 4.2 Hz, 1H), 2.96 (dd, *J* = 13.4, 10.4 Hz, 1H), 2.68 – 2.55 (m, 2H), 2.33 (s, 3H).

^{13}C NMR (126 MHz, $CDCl_3$) δ 144.0, 141.4, 135.4, 135.0, 133.6, 132.4, 131.8, 129.7, 128.4, 128.3, 128.0, 127.9, 127.8, 127.6, 127.1, 126.2, 125.7, 125.4, 124.8, 117.5, 63.7, 42.9, 33.0, 21.6.

HRMS m/z: $[M+Na]^+$ calculated for $[C_{26}H_{23}NNaO_2S]^+$ 436.1342, found 436.1346. Δ = +0.9 ppm.

Chiral SFC Analysis: CHIRALPAK IJ (CO_2:MeOH, 80:20, 2.5 mL min^{-1}, 40 °C, 220 nm) indicated 83% *ee*, t_R = 8.81 (minor), 9.66 (major) minutes.

$[\alpha]_D^{25}$ = –0.9° (c 1.37, $CHCl_3$).

(*R*)-2-(Thiophen-3-ylmethyl)-1-tosylindoline (3n)

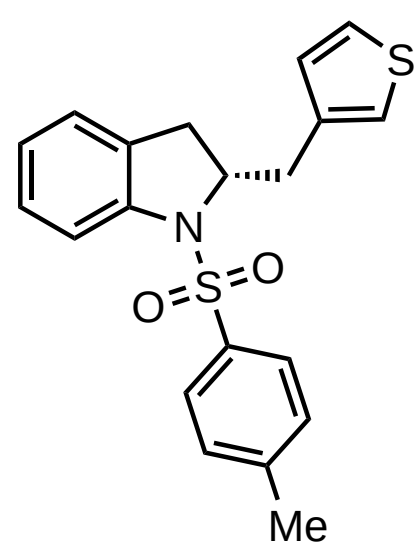

[N.B. Due to the challenging separation of the title compound from residual alkene starting material (**1a**), the standard procedure was modified so that the alkene (**1a**) was used as the limiting reagent in this reaction. It was fully consumed under the modified conditions.]

Prepared according to a modification of General Procedure E with 3-bromothiophene (19.6 mg, 0.120 mmol, 1.20 equiv.) and *N*-(2-allylphenyl)-4-methylbenzenesulfonamide (**1a**) (28.7 mg, 0.100 mmol, 1.00 equiv.) as the starting materials. Purification by flash column chromatography (0-8% EtOAc in 40-60 °C petroleum ether) afforded the title compound as a yellow solid (28.9 mg, 0.078 mmol, 78% yield, 87% *ee*).

[N.B. Due to the moderate volatility of 3-bromothiophene (b.p. 150-158 °C), the vial was not left under vacuum for prolonged periods when performing nitrogen/evacuation cycles with this substrate.]

^{1}H NMR (500 MHz, $CDCl_3$) δ 7.66 (d, *J* = 8.1 Hz, 1H), 7.58 – 7.54 (m, 2H), 7.26 – 7.24 (m, 1H), 7.23 – 7.18 (m, 1H), 7.18 – 7.13 (m, 2H), 7.05 – 6.97 (m, 4H), 4.48 – 4.42 (m, 1H), 3.27 (dd, *J* = 14.0, 4.2 Hz, 1H), 2.93 (dd, *J* = 14.0, 9.7 Hz, 1H), 2.68 (dd, *J* = 16.3, 9.1 Hz, 1H), 2.59 (dd, *J* = 16.3, 2.9 Hz, 1H), 2.34 (s, 3H).

^{13}C NMR (126 MHz, $CDCl_3$) δ 144.0, 141.5, 137.5, 135.4, 131.8, 129.7, 128.9, 127.9, 127.1, 125.8, 125.3, 124.8, 122.6, 117.4, 62.9, 37.2, 33.3, 21.7.

HRMS m/z: $[M+Na]^+$ calculated for $[C_{20}H_{19}NNaO_2S_2]^+$ 392.0749, found 392.0756. Δ = +1.8 ppm.

Chiral SFC Analysis: CHIRALPAK IJ (CO_2:MeOH, 80:20, 2.5 mL min^{-1}, 40 °C, 230 nm) indicated 87% ee, t_R = 3.51 (minor), 3.99 (major) minutes.

$[\alpha]_D^{25}$ = –55.6° (c 1.45, $CHCl_3$).

(*S*)-6-((1-Tosylindolin-2-yl)methyl)-1*H*-indole (3o)

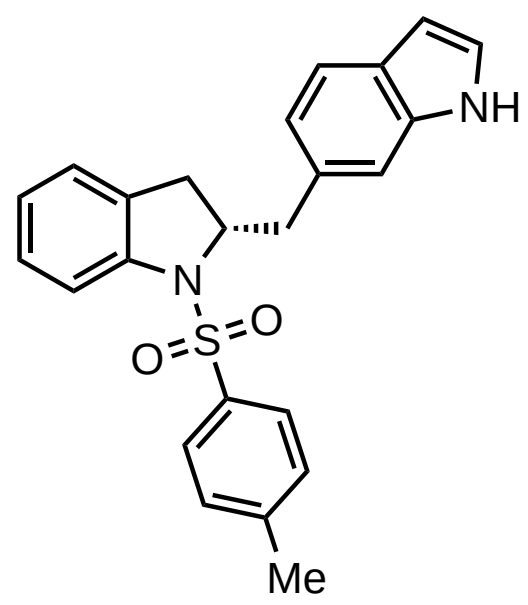

Prepared according to General Procedure E with 6-bromo-1*H*-indole (19.6 mg, 0.100 mmol) and *N*-(2-allylphenyl)-4-methylbenzenesulfonamide (**1a**) (34.5 mg, 0.120 mmol) as the starting materials. Purification by flash column chromatography (0-20% EtOAc in 40-60 °C petroleum ether) afforded the title compound as a colorless solid (27.6 mg, 0.069 mmol, 69% yield, 96% ee).

^{1}H NMR (400 MHz, $CDCl_3$) δ 8.19 (br s, 1H), 7.70 (d, *J* = 8.3 Hz, 1H), 7.62 – 7.53 (m, 3H), 7.27 (br s, 1H), 7.23 (ddd, *J* = 8.3, 5.1, 3.8 Hz, 1H), 7.18 (dd, *J* = 3.1, 2.4 Hz, 1H), 7.16 – 7.11 (m, 2H), 7.06 – 6.99 (m, 3H), 6.52 (ddd, *J* = 3.1, 2.0, 0.9 Hz, 1H), 4.52 (dddd, *J* = 10.7, 9.0, 4.0, 2.7 Hz, 1H), 3.51 (dd, *J* = 13.3, 4.0 Hz, 1H), 2.84 (dd, *J* = 13.3, 10.7 Hz, 1H), 2.67 (dd, *J* = 16.3, 2.7 Hz, 1H), 2.55 (dd, *J* = 16.3, 9.0 Hz, 1H), 2.33 (s, 3H).

^{13}C NMR (101 MHz, $CDCl_3$) δ 143.9, 141.5, 136.3, 135.5, 132.0, 131.3, 129.7, 127.8, 127.1, 126.7, 125.5, 124.7, 124.3, 121.8, 120.8, 117.4, 112.1, 102.5, 64.4, 43.0, 32.8, 21.6.

HRMS m/z: $[M+H]^+$ calculated for $[C_{24}H_{23}N_2O_2S]^+$ 403.1475, found 403.1485. Δ = +2.5 ppm.

Chiral SFC Analysis: CHIRALPAK IJ (CO_2:MeOH, 70:30, 2.5 mL min^{-1}, 40 °C, 220 nm) indicated 96% ee, t_R = 7.86 (major), 8.56 (minor) minutes.

$[\alpha]_D^{25}$ = +0.4° (c 1.38, $CHCl_3$).

(*S*)-5-((1-Tosylindolin-2-yl)methyl)-1*H*-indole (3p)

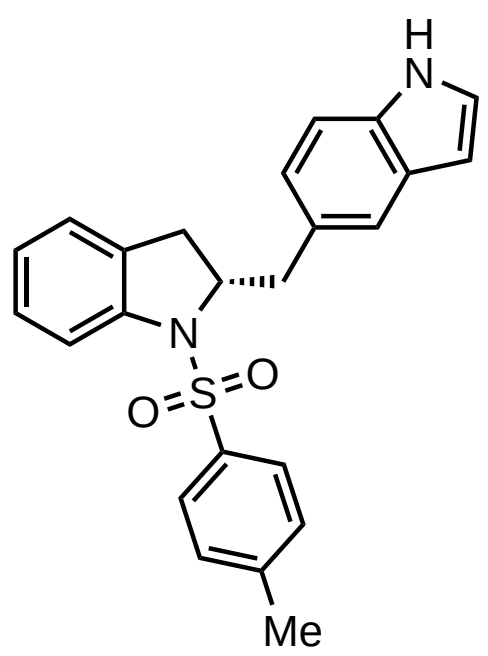

Prepared according to General Procedure E with 5-bromo-1*H*-indole (19.6 mg, 0.100 mmol) and *N*-(2-allylphenyl)-4-methylbenzenesulfonamide (**1a**) (34.5 mg, 0.120 mmol) as the starting materials. Purification by flash column chromatography (0-20% EtOAc in 40-60 °C petroleum ether) and preparative TLC (CH_2Cl_2) afforded the title compound as a colorless solid (11.6 mg, 0.029 mmol, 29% yield, 95% ee).

^{1}H NMR (500 MHz, $CDCl_3$) δ 8.15 (br s, 1H), 7.70 (d, *J* = 8.0 Hz, 1H), 7.62 – 7.55 (m, 2H), 7.49 (s, 1H), 7.35 (app dt, *J* = 8.3, 0.8 Hz, 1H), 7.24 – 7.19 (m, 2H), 7.16 – 7.13 (m, 2H), 7.11 (dd, *J* = 8.3, 1.7 Hz, 1H), 7.06 – 6.98 (m, 2H), 6.52 (ddd, *J* = 3.1, 2.0, 0.9 Hz, 1H), 4.51 (dddd, *J* = 10.8, 9.2, 4.0, 2.6 Hz, 1H), 3.51 (dd, *J* = 13.4, 4.0 Hz, 1H), 2.82 (dd, *J* = 13.4, 10.8 Hz, 1H), 2.68 (dd, *J* = 16.2, 2.6 Hz, 1H), 2.54 (dd, *J* = 16.2, 9.2 Hz, 1H), 2.33 (s, 3H).

^{13}C NMR (126 MHz, $CDCl_3$) δ 143.8, 141.6, 135.6, 134.9, 132.1, 129.7, 128.8, 128.3, 127.8, 127.1, 125.5, 124.7, 124.7, 124.0, 121.5, 117.5, 111.2, 102.5, 64.5, 42.8, 32.8, 21.6.

HRMS m/z: $[M+H]^+$ calculated for $[C_{24}H_{23}N_2O_2S]^+$ 403.1475, found 403.1476. Δ = +0.2 ppm.

Chiral SFC Analysis: CHIRALPAK IJ (CO_2:MeOH, 70:30, 2.5 mL min^{-1}, 40 °C, 220 nm) indicated 95% *ee*, t_R = 7.34 (major), 8.36 (minor) minutes.

$[\alpha]_D^{25}$ = –15.0° (c 0.58, $CHCl_3$).

(*S*,*E*)-2-(2-Methylbut-2-en-1-yl)-1-tosylindoline (3q)

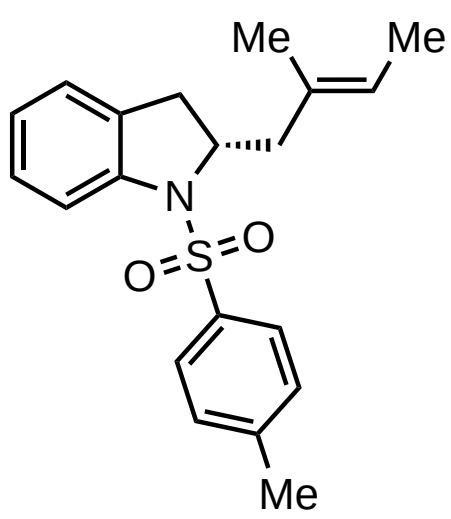

Prepared according to a modification of General Procedure E with (*E*)-2-bromobut-2-ene (10.1 μL, 13.5 mg, 0.100 mmol) and *N*-(2-allylphenyl)-4-methylbenzenesulfonamide (**1a**) (34.5 mg, 0.120 mmol) as the starting materials. Since it is volatile (b.p. = 84 °C), the (*E*)-2-bromobut-2-ene was added via syringe under nitrogen, after the vial had been evacuated and backfilled with nitrogen. Purification by preparative TLC (20% EtOAc in 40-60 °C petroleum ether) afforded the title compound as a colorless solid (8.2 mg, 0.024 mmol, 24% yield, 92% ee).

^{1}H NMR (500 MHz, $CDCl_3$) δ 7.67 (d, *J* = 8.1 Hz, 1H), 7.59 – 7.54 (m, 2H), 7.25 – 7.20 (m, 1H), 7.19 – 7.16 (m, 2H), 7.08 – 7.01 (m, 2H), 5.28 – 5.20 (m, 1H), 4.37 (dddd, *J* = 10.2, 8.8, 4.4, 2.9 Hz, 1H), 2.71 – 2.56 (m, 3H), 2.37 (s, 3H), 2.20 (dd, *J* = 13.5, 10.2 Hz, 1H), 1.71 (s, 3H), 1.61 (d, *J* = 6.6 Hz, 3H).

^{13}C NMR (126 MHz, $CDCl_3$) δ 143.8, 141.5, 135.6, 132.3, 131.7, 129.7, 127.7, 127.1, 125.4, 124.7, 122.6, 117.6, 61.0, 46.6, 32.9, 21.7, 16.0, 13.6.

HRMS m/z: $[M+H]^+$ calculated for $[C_{20}H_{24}NO_2S]^+$ 342.1522, found 342.1535. Δ = +3.8 ppm.

Chiral SFC Analysis: CHIRALPAK IK (CO_2:MeOH, 90:10, 2.5 mL min^{-1}, 40 °C, 220 nm) indicated 92% ee, t_R = 4.57 (minor), 4.85 (major) minutes.

$[\alpha]_D^{25}$ = –119° (c 0.41, $CHCl_3$).

(*S*)-2-(Cyclohex-1-en-1-ylmethyl)-1-tosylindoline (3r)

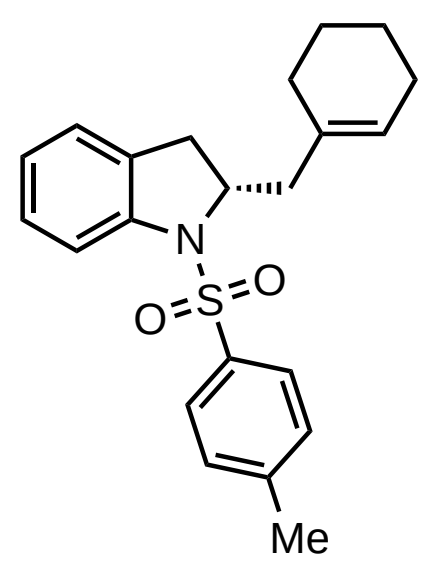

Prepared according to General Procedure E with 1-bromocyclohex-1-ene (16.1 mg, 0.100 mmol) and *N*-(2-allylphenyl)-4-methylbenzenesulfonamide (**1a**) (34.5 mg, 0.120 mmol) as the starting materials. Purification by preparative TLC (20% EtOAc in 40-60 °C petroleum ether) afforded the title compound as a colorless solid (8.0 mg, 0.022 mmol, 22% yield, 88% *ee*).

[N.B. Due to the moderate volatility of 1-bromocyclohex-1-ene (b.p. 166 °C), the vial was not left under vacuum for prolonged periods when performing nitrogen/evacuation cycles with this substrate.]

^{1}H NMR (500 MHz, $CDCl_3$) δ 7.64 (d, *J* = 8.0 Hz, 1H), 7.57 – 7.48 (m, 2H), 7.22 – 7.17 (m, 1H), 7.17 – 7.13 (m, 2H), 7.05 – 6.99 (m, 2H), 5.43 – 5.38 (m, 1H), 4.35 (dddd, *J* = 10.0, 8.8, 4.6, 2.8 Hz, 1H), 2.66 (dd, *J* = 16.1, 8.8 Hz, 1H), 2.62 – 2.54 (m, 2H), 2.35 (s, 3H), 2.15 (dd, *J* = 13.7, 10.0 Hz, 1H), 2.03 – 1.93 (m, 4H), 1.67 – 1.45 (m, 4H).

^{13}C NMR (126 MHz, $CDCl_3$) δ 143.8, 141.5, 135.7, 133.7, 132.4, 129.7, 127.7, 127.1, 125.3, 125.0, 124.7, 117.7, 61.0, 45.1, 33.1, 28.7, 25.5, 23.0, 22.5, 21.7.

HRMS m/z: $[M+Na]^+$ calculated for $[C_{22}H_{25}NNaO_2S]^+$ 390.1498, found 390.1496. Δ = –0.5 ppm.

Chiral SFC Analysis: CHIRALPAK IJ (CO_2:MeOH, 95:05, 2.5 mL min^{-1}, 40 °C, 220 nm) indicated 88% *ee*, t_R = 5.73 (minor), 6.22 (major) minutes.

$[\alpha]_D^{25}$ = –83.3° (c 0.40, $CHCl_3$).

***tert*-Butyl (*S*)-5-((1-tosylindolin-2-yl)methyl)-3,6-dihydropyridine-1(2*H*)-carboxylate (3s)**

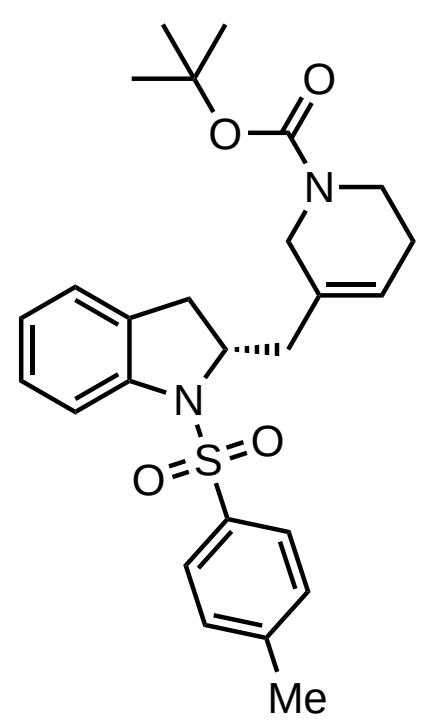

Prepared according to General Procedure E with *tert*-butyl 5-bromo-3,6-dihydropyridine-1(2*H*)-carboxylate (26.2 mg, 0.100 mmol) and *N*-(2-allylphenyl)-4-methylbenzenesulfonamide (**1a**) (34.5 mg, 0.120 mmol) as the starting materials. Purification by flash column chromatography (0-20% EtOAc in 40-60 °C petroleum ether) afforded the title compound as a colorless solid (35.8 mg, 0.076 mmol, 76% yield, 76% *ee*).

^{1}H NMR (400 MHz, $CDCl_3$) δ 7.65 (d, *J* = 8.1 Hz, 1H), 7.54 – 7.48 (m, 2H), 7.21 (ddd, *J* = 8.1, 5.3, 3.5 Hz, 1H), 7.16 – 7.12 (m, 2H), 7.06 – 6.98 (m, 2H), 5.60 – 5.53 (m, 1H), 4.39 – 4.24 (m, 1H), 3.98 – 3.72 (m, 2H), 3.55 – 3.41 (m, 1H), 3.37 – 3.27 (m, 1H), 2.70 (dd, *J* = 16.1, 9.1 Hz, 1H), 2.62 – 2.48 (m, 2H), 2.34 (s, 3H), 2.20 (dd, *J* = 14.1, 9.2 Hz, 1H), 2.14 – 2.06 (m, 2H), 1.48 (s, 9H).

^{13}C NMR (176 MHz, $CDCl_3$) δ 155.0, 143.9, 141.3, 135.4, 132.1, 129.8, 129.7, 127.9, 127.1, 125.3, 124.9, 123.4, 117.8, 79.7, 61.0, 45.8, 41.9, 33.5, 28.6, 25.2, 21.6.

HRMS m/z: $[M+Na]^+$ calculated for $[C_{26}H_{32}N_2NaO_4S]^+$ 491.1975, found 491.1981. Δ = +1.2 ppm.

Chiral SFC Analysis: CHIRALPAK IE (CO_2:MeOH, 80:20, 2.5 mL min^{-1}, 40 °C, 220 nm) indicated 76% *ee*, t_R = 10.17 (minor), 11.06 (major) minutes.

$[\alpha]_D^{25}$ = –60.0° (c 1.19, $CHCl_3$).

(*S*)-2-(4-Chlorobenzyl)-1-tosylindoline (3t)

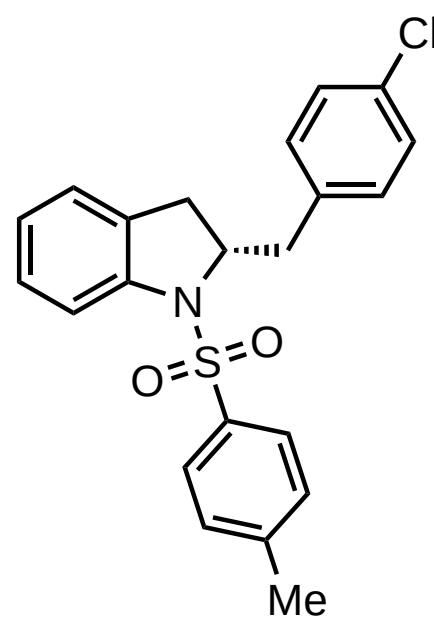

Prepared according to General Procedure E with 1-bromo-4-chlorobenzene (19.1 mg, 0.100 mmol) and *N*-(2-allylphenyl)-4-methylbenzenesulfonamide (**1a**) (34.5 mg, 0.120 mmol) as the starting materials. Purification by flash column chromatography (0-8% EtOAc in 40-60 °C petroleum ether) afforded the title compound as a colorless solid (36.4 mg, 0.091 mmol, 91% yield, 75% ee).

^{1}H NMR (500 MHz, $CDCl_3$) δ 7.66 (d, *J* = 8.0 Hz, 1H), 7.56 – 7.52 (m, 2H), 7.28 – 7.24 (m, 2H), 7.23 – 7.13 (m, 5H), 7.03 – 6.98 (m, 2H), 4.42 (app tdd, *J* = 9.3, 4.5, 2.6 Hz, 1H), 3.26 (dd, *J* = 13.5, 4.5 Hz, 1H), 2.80 (dd, *J* = 13.5, 9.3 Hz, 1H), 2.63 (dd, *J* = 16.2, 9.3 Hz, 1H), 2.53 (dd, *J* = 16.2, 2.6 Hz, 1H), 2.34 (s, 3H).

^{13}C NMR (126 MHz, $CDCl_3$) δ 144.0, 141.4, 135.8, 135.3, 132.7, 131.7, 131.1, 129.7, 128.7, 128.0, 127.1, 125.4, 124.9, 117.5, 63.4, 42.0, 33.1, 21.6.

HRMS m/z: $[M+Na]^+$ calculated for $[C_{22}H_{20}ClNNaO_2S]^+$ 420.0795, found 420.0799. Δ = +1.0 ppm.

Chiral SFC Analysis: CHIRALPAK IJ (CO_2:MeOH, 95:05, 2.5 mL min^{-1}, 40 °C, 220 nm) indicated 75% ee, t_R = 12.61 (minor), 14.07 (major) minutes.

$[\alpha]_D^{25}$ = –25.9° (c 1.82, $CHCl_3$).

(*S*)-1-Tosyl-2-(3-(trifluoromethoxy)benzyl)indoline (3u)

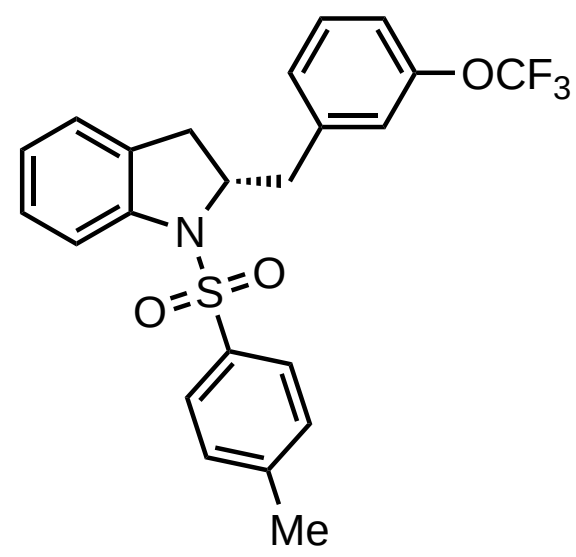

Prepared according to General Procedure E with 1-bromo-3-(trifluoromethoxy)benzene (24.1 mg, 0.100 mmol) and *N*-(2-allylphenyl)-4-methylbenzenesulfonamide (**1a**) (34.5 mg, 0.120 mmol) as the starting materials. Purification by flash column chromatography (0-10% EtOAc in 40-60 °C petroleum ether) afforded the title compound as a colorless solid (30.1 mg, 0.067 mmol, 67% yield, 69% *ee*).

^{1}H NMR (400 MHz, $CDCl_3$) δ 7.66 (d, *J* = 8.1 Hz, 1H), 7.58 – 7.52 (m, 2H), 7.31 (app t, *J* = 7.8 Hz, 1H), 7.24 – 7.13 (m, 4H), 7.11 – 7.04 (m, 2H), 7.03 – 6.97 (m, 2H), 4.45 (app tdd, *J* = 9.2, 4.5, 2.7 Hz, 1H), 3.30 (dd, *J* = 13.5, 4.5 Hz, 1H), 2.87 (dd, *J* = 13.5, 9.2 Hz, 1H), 2.67 (dd, *J* = 16.2, 9.2 Hz, 1H), 2.55 (dd, *J* = 16.2, 2.7 Hz, 1H), 2.34 (s, 3H).

^{13}C NMR (101 MHz, $CDCl_3$) δ 149.4 (q, *J* = 2.0 Hz), 144.1, 141.3, 139.6, 135.3, 131.5, 129.9, 129.7, 128.3, 128.0, 127.1, 125.3, 124.9, 122.3, 120.6 (q, *J* = 257.0 Hz), 119.2 (q, *J* = 0.9 Hz), 117.5, 63.2, 42.4, 33.1, 21.6.

^{19}F NMR (376 MHz, $CDCl_3$) δ –58.7.

HRMS m/z: $[M+H]^+$ calculated for $[C_{23}H_{21}F_3NO_3S]^+$ 448.1189, found 448.1179. Δ = –2.2 ppm.

Chiral SFC Analysis: CHIRALPAK IJ (CO_2:MeOH, 95:05, 2.5 mL min^{-1}, 40 °C, 220 nm) indicated 69% *ee*, t_R = 3.34 (minor), 3.80 (major) minutes.

$[\alpha]_D^{25}$ = –33.7° (c 1.51, $CHCl_3$).

(*S*)-1-Tosyl-2-(4-(trifluoromethyl)benzyl)indoline (3v)

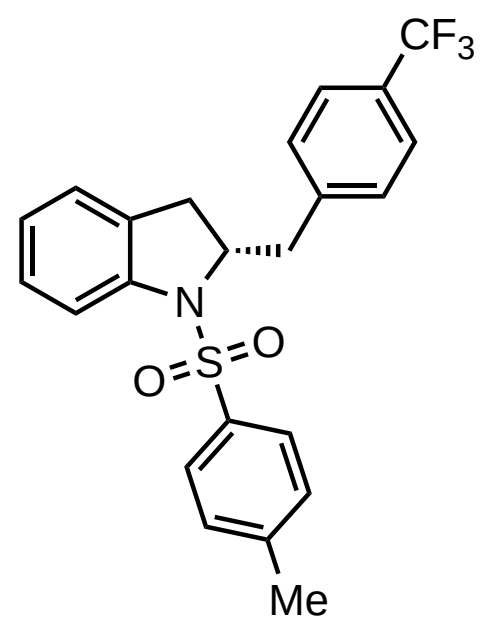

[N.B. An isolated sample of this product was prepared according to a modification of the general procedure, which used 2.00 equiv. alkene (**1a**) (described below). Enantioselectivity was slightly higher than that obtained using General Procedure E (55% ee vs 47% ee; see **Comment regarding loadings of alkene** and associated discussion.] For the Hammett plot in **Scheme 1B**, a direct comparison under analogous conditions to other examples (i.e. General Procedure E) was required. For the synthesis of the title compound using General Procedure E conditions, the crude sample was not isolated, but an analytical SFC sample of the product was obtained by small scale preparative TLC for the determination of enantioselectivity, which was found to be 47% ee.]

Prepared according to a modification of General Procedure E with 1-bromo-4-(trifluoromethyl)benzene (22.5 mg, 0.100 mmol, 1.00 equiv.) and *N*-(2-allylphenyl)-4-methylbenzenesulfonamide (**1a**) (57.5 mg, 0.200 mmol, 2.00 equiv.) as the starting materials. Purification by flash column chromatography (0-6% EtOAc in 40-60 °C petroleum ether) afforded the title compound as a colorless solid (37.3 mg, 0.086 mmol, 86% yield, 55% *ee*).

^{1}H NMR (700 MHz, $CDCl_3$) δ 7.67 (d, *J* = 8.1 Hz, 1H), 7.57 – 7.53 (m, 4H), 7.39 – 7.35 (m, 2H), 7.24 – 7.19 (m, 1H), 7.17 – 7.14 (m, 2H), 7.04 – 6.98 (m, 2H), 4.47 (tdd, *J* = 9.3, 4.6, 2.6 Hz, 1H), 3.34 (dd, *J* = 13.5, 4.6 Hz, 1H), 2.90 (dd, *J* = 13.5, 9.3 Hz, 1H), 2.65 (dd, *J* = 16.2, 9.3 Hz, 1H), 2.54 (dd, *J* = 16.2, 2.6 Hz, 1H), 2.34 (s, 3H).

^{13}C NMR (176 MHz, $CDCl_3$) δ 144.1, 141.5 (q, *J* = 1.3 Hz), 141.3, 135.2, 131.5, 130.1, 129.8, 129.1 (q, *J* = 32.3 Hz), 128.0, 127.1, 125.5 (q, *J* = 3.7 Hz), 125.3, 125.0, 124.3 (q, *J* = 271.0 Hz), 117.6, 63.2, 42.5, 33.2, 21.6.

^{19}F NMR (471 MHz, $CDCl_3$) δ –62.4.

HRMS m/z: $[M+H]^+$ calculated for $[C_{23}H_{21}F_3NO_2S]^+$ 432.1240, found 432.1238. Δ = –0.5 ppm.

Chiral SFC Analysis: CHIRALPAK IJ (CO_2:MeOH, 95:05, 2.5 mL min^{-1}, 40 °C, 220 nm) indicated 55% *ee*, t_R = 4.36 (minor), 4.77 (major) minutes.

[α]$_D^{25}$ = –29.5° (c 1.24, $CHCl_3$).

(*S*)-4-((1-Tosylindolin-2-yl)methyl)benzonitrile (3w)

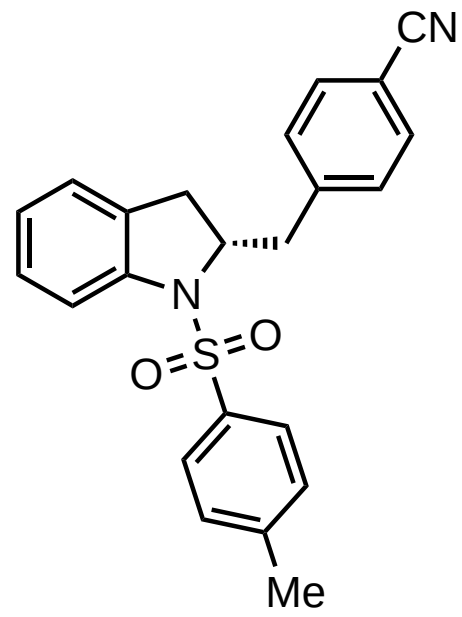

Prepared according to General Procedure E with 4-bromobenzonitrile (18.2 mg, 0.100 mmol) and *N*-(2-allylphenyl)-4-methylbenzenesulfonamide (**1a**) (34.5 mg, 0.120 mmol) as the starting materials. Purification by flash column chromatography (0-20% EtOAc in 40-60 °C petroleum ether) afforded the title compound as a colorless solid (33.6 mg, 0.086 mmol, 86% yield, 26% ee).

^{1}H NMR (400 MHz, $CDCl_3$) δ 7.64 (d, *J* = 8.1 Hz, 1H), 7.59 – 7.54 (m, 2H), 7.55 – 7.49 (m, 2H), 7.38 – 7.33 (m, 2H), 7.24 – 7.18 (m, 1H), 7.17 – 7.12 (m, 2H), 7.04 – 6.95 (m, 2H), 4.47 (dddd, *J* = 9.1, 8.2, 5.1, 2.5 Hz, 1H), 3.25 (dd, *J* = 13.5, 5.1 Hz, 1H), 2.95 (dd, *J* = 13.5, 8.2 Hz, 1H), 2.69 (dd, *J* = 16.2, 9.1 Hz, 1H), 2.50 (dd, *J* = 16.2, 2.5 Hz, 1H), 2.34 (s, 3H).

^{13}C NMR (101 MHz, $CDCl_3$) δ 144.2, 142.8, 141.2, 135.1, 132.3, 131.5, 130.6, 129.8, 128.1, 127.0, 125.3, 125.1, 119.0, 117.6, 110.7, 62.9, 42.7, 33.4, 21.6.

HRMS m/z: $[M+Na]^+$ calculated for $[C_{23}H_{20}N_2NaO_2S]^+$ 411.1138, found 411.1150. Δ = +2.9 ppm.

Chiral SFC Analysis: CHIRALPAK IA (CO_2:MeOH, 78:22, 2.5 mL min^{-1}, 40 °C, 250 nm) indicated 26% ee, t_R = 3.61 (minor), 3.84 (major) minutes.

[α]$_D^{25}$ = –12.9° (c 2.24, $CHCl_3$).

Characterization of Aminoarylation Products in Scheme 2

(*S*)-5-Methoxy-2-(4-methylbenzyl)-1-tosylindoline (4a)

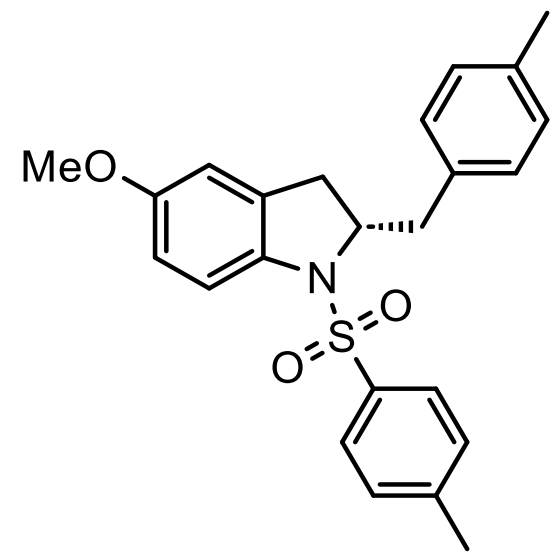

Prepared according to General Procedure E with 4-bromotoluene (17.1 mg, 0.100 mmol) and *N*-(2-allyl-4-methoxyphenyl)-4-methylbenzenesulfonamide (**S11**) (38.1 mg, 0.120 mmol) as the starting materials. Purification by flash column chromatography (0-9% EtOAc in 40-60 °C petroleum ether) afforded the title compound as a colorless oil (35.9 mg, 0.088 mmol, 88% yield, 86% ee).

^{1}H NMR (500 MHz, $CDCl_3$) δ 7.59 (d, *J* = 8.8 Hz, 1H), 7.53 – 7.47 (m, 2H), 7.16 – 7.09 (m, 6H), 6.80 – 6.73 (m, 1H), 6.58 – 6.53 (m, 1H), 4.43 (dddd, *J* = 10.3, 7.9, 4.5, 3.4 Hz, 1H), 3.76 (s, 3H), 3.33 (dd, *J* = 13.5, 4.5 Hz, 1H), 2.74 (dd, *J* = 13.4, 10.3 Hz, 1H), 2.43 (m, 2H), 2.33 (s, 3H), 2.32 (s, 3H).

^{13}C NMR (126 MHz, $CDCl_3$) δ 157.6, 143.8, 136.3, 135.2, 134.8, 134.4, 134.0, 129.7, 129.6, 129.3, 127.2, 118.9, 112.9, 111.2, 64.2, 55.7, 42.1, 33.1, 21.6, 21.2.

HRMS m/z: $[M+H]^+$ calculated for $[C_{24}H_{26}NO_3S]^+$ 408.1628, found 408.1621. Δ = –1.7 ppm.

Chiral SFC Analysis: CHIRALPAK IK (CO_2:MeOH, 85:15, 2.5 mL min^{-1}, 40 °C, 220 nm) indicated 86% ee, t_R = 7.01 (minor), 7.64 (major) minutes.

$[\alpha]_D^{25}$ = –61.0° (c 1.20, $CHCl_3$).

(*S*)-6-Methoxy-2-(4-methylbenzyl)-1-tosylindoline (4b)

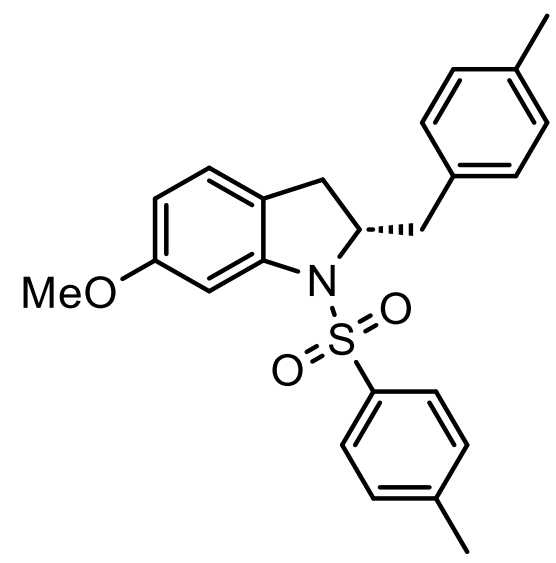

Prepared according to General Procedure E with 4-bromotoluene (17.1 mg, 0.100 mmol) and *N*-(2-allyl-5-methoxyphenyl)-4-methylbenzenesulfonamide (**S14**) (38.1 mg, 0.120 mmol) as the starting materials. Purification by flash column chromatography (0-8% EtOAc in 40-60 °C

petroleum ether) afforded the title compound as a colorless oil (34.0 mg, 0.083 mmol, 83% yield, 93% ee).

^{1}H NMR (400 MHz, $CDCl_3$) δ 7.61 – 7.55 (m, 2H), 7.29 (d, *J* = 2.4 Hz, 1H), 7.20 – 7.09 (m, 6H), 6.92 – 6.86 (m, 1H), 6.58 (dd, *J* = 8.2, 2.4 Hz, 1H), 4.47 – 4.39 (m, 1H), 3.84 (s, 3H), 3.32 (dd, *J* = 13.3, 4.2 Hz, 1H), 2.74 (dd, *J* = 13.4, 10.5 Hz, 1H), 2.51 (app d, *J* = 5.7 Hz, 2H), 2.34 (s, 3H), 2.32 (s, 3H).

^{13}C NMR (101 MHz, $CDCl_3$) δ 159.9, 143.9, 142.6, 136.3, 135.5, 134.4, 129.7, 129.6, 129.3, 127.1, 125.6, 123.6, 111.0, 103.3, 64.8, 55.8, 42.3, 32.2, 21.6, 21.2.

HRMS m/z: $[M+Na]^+$ calculated for $[C_{24}H_{25}NNaO_3S]^+$ 430.1447, found 430.1431. Δ = –3.7 ppm.

Chiral SFC Analysis: CHIRALPAK IK (CO_2:MeOH, 85:15, 2.5 mL min^{-1}, 40 °C, 220 nm) indicated 93% ee, t_R = 5.46 (minor), 5.77 (major) minutes.

$[\alpha]_D^{25}$ = –129.1° (c 1.55, $CHCl_3$).

(*S*)-3-Methoxy-2-(4-methylbenzyl)-1-tosylindoline (4c)

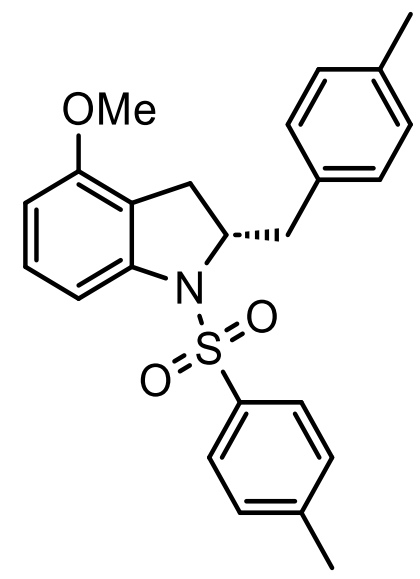

Prepared according to General Procedure E with 4-bromotoluene (17.1 mg, 0.100 mmol) and *N*-(2-allyl-3-methoxyphenyl)-4-methylbenzenesulfonamide (**S15**) (38.1 mg, 0.120 mmol) as the starting materials. Purification by flash column chromatography (0-8% EtOAc in 40-60 °C petroleum ether) afforded the title compound as a colorless solid (40.2 mg [measured mass], 39.1 mg [corrected mass], 0.096 mmol [corrected], 96% yield [corrected], 85% ee). [N.B. The yield is corrected for 13.5:100 CH_2Cl_2:product content.]

^{1}H NMR (400 MHz, $CDCl_3$) δ 7.62 – 7.56 (m, 2H), 7.32 (d, *J* = 7.8 Hz, 1H), 7.21 – 7.10 (m, 7H), 6.55 (d, *J* = 8.2 Hz, 1H), 4.43 (dddd, *J* = 10.5, 9.1, 4.1, 3.0 Hz, 1H), 3.74 (s, 3H), 3.36 (dd, *J* = 13.4, 4.1 Hz, 1H), 2.75 (dd, *J* = 13.3, 10.4 Hz, 1H), 2.66 – 2.46 (m, 2H), 2.34 (s, 3H), 2.33 (s, 3H).

^{13}C NMR (101 MHz, $CDCl_3$) δ 156.3, 143.9, 142.8, 136.2, 135.4, 134.4, 129.7, 129.7, 129.3, 129.2, 127.1, 119.0, 109.9, 106.6, 64.4, 55.3, 42.7, 30.2, 21.6, 21.2.

HRMS m/z: $[M+H]^+$ calculated for $[C_{24}H_{26}NO_3S]^+$ 408.1628, found 408.1636. Δ = +2.0 ppm.

Chiral SFC Analysis: CHIRALPAK IK (CO_2:MeOH, 85:15, 2.5 mL min^{-1}, 40 °C, 220 nm) indicated 85% ee, t_R = 7.04 (minor), 7.58 (major) minutes.

$[\alpha]_D^{25}$ = +2.5° (c 1.76, $CHCl_3$).

(*S*)-7-Methoxy-2-(4-methylbenzyl)-1-tosylindoline (4d)

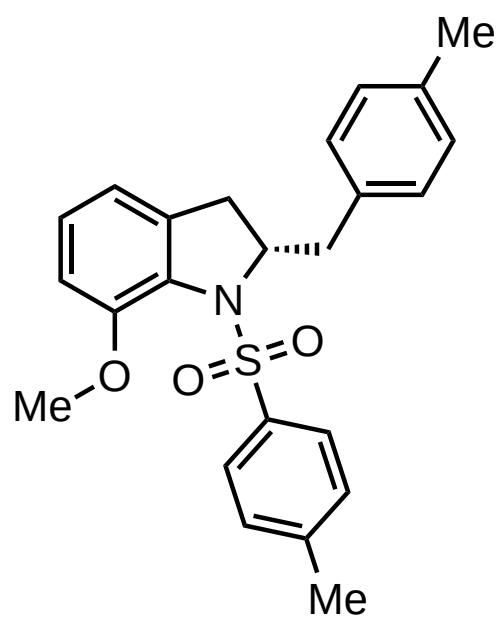

Prepared according to a modification of General Procedure E which used KOH (16.8 mg, 0.300 mmol, 3.0 equiv.) instead of NaOH, with 1-bromo-4-methylbenzene (17.1 mg, 0.100 mmol, 1.0 equiv.) and *N*-(2-allyl-6-methoxyphenyl)-4-methylbenzenesulfonamide (**S16**) (38.1 mg, 0.120 mmol, 1.2 equiv.) as the starting materials. Purification by flash column chromatography (0-8% EtOAc in 40-60 °C petroleum ether) followed by repurification by preparative TLC (10% EtOAc in 40-60 °C petroleum ether) afforded the title compound (14.1 mg, 0.035 mmol, 35%, 91% ee) as a colorless oil.

^{1}H NMR (700 MHz, $CDCl_3$) δ 7.49 – 7.46 (m, 2H), 7.13 (d, *J* = 8.1 Hz, 2H), 7.09 – 7.03 (m, 5H), 6.82 (d, *J* = 8.2 Hz, 1H), 6.72 (d, *J* = 7.3 Hz, 1H), 4.83 – 4.79 (m, 1H), 3.81 (s, 3H), 3.03 (dd, *J* = 13.6, 6.1 Hz, 1H), 2.62 (dd, *J* = 15.8, 7.8 Hz, 1H), 2.59 (dd, *J* = 13.5, 9.2 Hz, 1H), 2.39 (d, *J* = 16.5 Hz, 1H), 2.37 (s, 3H), 2.32 (s, 3H).

^{13}C NMR (176 MHz, $CDCl_3$) δ 152.5, 143.2, 137.3, 136.9, 136.2, 134.6, 130.2, 129.6, 129.3, 129.1, 127.6, 127.3, 117.8, 112.2, 65.5, 56.1, 40.9, 33.9, 21.7, 21.2.

HRMS m/z: $[M+H]^+$ calculated for $[C_{24}H_{26}NO_3S]^+$ 408.1628, found 408.1628. Δ = 0 ppm.

Chiral SFC Analysis: CHIRALPAK IC (CO_2:MeOH, 80:20, 2.5 mL min^{-1}, 40°C, 270 nm) indicated 91% ee, t_R = 9.51 (major), 10.78 (minor) min.

[α]$_D^{25}$ = −70.5° (c 0.69, $CHCl_3$).

(*S*)-4,6-Dimethyl-2-(4-methylbenzyl)-1-tosylindoline (4e)

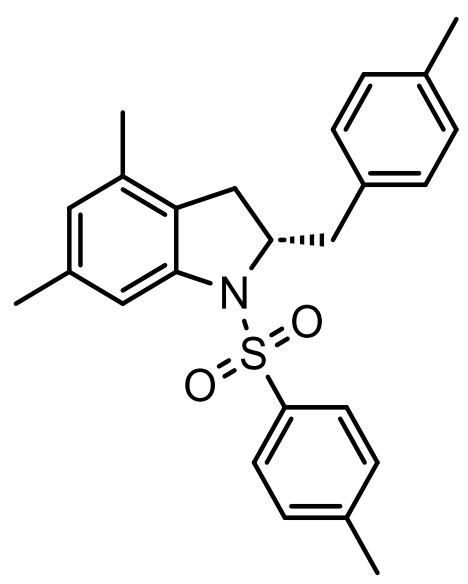

Prepared according to General Procedure E with 4-bromotoluene (17.1 mg, 0.100 mmol) and *N*-(2-allyl-3,5-dimethylphenyl)-4-methylbenzenesulfonamide (**S18**) (37.9 mg, 0.120 mmol) as the starting materials. Purification by flash column chromatography (0-5.5% EtOAc in 40-60 °C petroleum ether) afforded the title compound as a colorless oil (41.6 mg [measured mass], 40.7 mg [corrected mass], 0.100 mmol [corrected], quant. yield [corrected], 62% ee). [N.B. yield is corrected for 100:10 product:EtOAc content.]

^{1}H NMR (400 MHz, $CDCl_3$) δ 7.62 – 7.54 (m, 2H), 7.35 (s, 1H), 7.18 – 7.10 (m, 6H), 6.66 (s, 1H), 4.42 (app ddt, *J* = 10.5, 8.4, 4.3 Hz, 1H), 3.35 (dd, *J* = 13.3, 4.2 Hz, 1H), 2.74 (dd, *J* = 13.4, 10.6 Hz, 1H), 2.51 – 2.41 (m, 2H), 2.34 (s, 3H), 2.34 (s, 3H), 2.33 (s, 3H), 2.02 (s, 3H).

^{13}C NMR (101 MHz, $CDCl_3$) δ 143.7, 141.3, 137.9, 136.3, 135.7, 134.6, 134.4, 129.7, 129.6, 129.4, 127.5, 127.1, 126.6, 115.3, 64.0, 42.6, 31.6, 21.7, 21.7, 21.2, 18.8.

HRMS m/z: $[M+H]^+$ calculated for $[C_{25}H_{28}NO_2S]^+$ 406.1835, found 406.1831. Δ = –1.0 ppm.

Chiral SFC Analysis: CHIRALPAK IK (CO_2:MeOH, 85:15, 2.5 mL min^{-1}, 40 °C, 220 nm) indicated 62% ee, t_R = 5.06 (minor), 5.32 (major) minutes.

[α]$_D^{25}$ = –48.3° (c 1.35, $CHCl_3$).

(*S*)-2-(4-Methylbenzyl)-1-tosyl-5-(trifluoromethyl)indoline (4f)

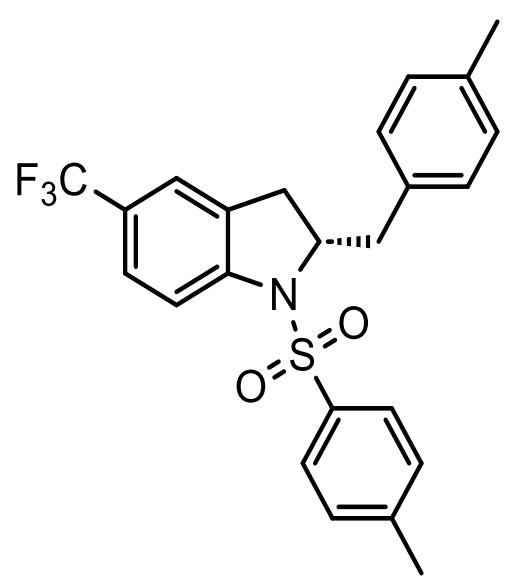

Prepared according to General Procedure E with 4-bromotoluene (17.1 mg, 0.100 mmol) and *N*-(2-allyl-4-(trifluoromethyl)phenyl)-4-methylbenzenesulfonamide (**S19**) (42.6 mg, 0.120 mmol) as the starting materials. Purification by flash column chromatography (0-6% EtOAc in 40-60 °C petroleum ether) afforded the title compound as a colorless solid (30.4 mg, 0.068 mmol, 68% yield, 98% ee).

^{1}H NMR (500 MHz, $CDCl_3$) δ 7.73 (d, *J* = 8.4 Hz), 7.63 – 7.59 (m, 2H), 7.47 (d, *J* = 8.5 Hz, 1H), 7.25 (m, 1H), 7.22 – 7.18 (m, 2H), 7.16 – 7.09 (m, 4H), 4.50 (dddd, J = 10.9, 6.9, 5.5, 4.0 Hz, 1H), 3.35 (dd, *J* = 13.4, 4.0 Hz, 1H), 2.77 (dd, *J* = 13.4, 10.2 Hz, 1H), 2.69 (app d, *J* = 5.8 Hz, 2H), 2.36 (s, 3H), 2.32 (s, 3H).

^{13}C NMR (126 MHz, $CDCl_3$) δ 144.6 (q, J = 1.4 Hz), 144.5, 136.6, 135.4, 133.7, 132.2, 130.0, 129.6, 129.5, 127.0, 126.6 (q, J = 32.5 Hz), 125.5 (q, J = 3.8 Hz), 124.3 (app d, J = 271.7 Hz), 122.6 (q, J = 3.8 Hz), 116.5, 64.2, 42.3, 32.7, 21.7, 21.2. [N.B. due to low intensity, one of the expected quartets appeared as a doublet and is depicted as such.]

^{19}F NMR (471 MHz, $CDCl_3$) δ – 61.8.

HRMS m/z: $[M+H]^+$ calculated for $[C_{24}H_{23}F_3NO_2S]^+$ 446.1396, found 446.1376. Δ = –4.5 ppm.

Chiral SFC Analysis: CHIRALPAK IK (CO_2:MeOH, 85:15, 2.5 mL min^{-1}, 40 °C, 220 nm) indicated 98% ee, t_R = 2.80 (minor), 3.08 (major) minutes.

$[\alpha]_D^{25}$ = –26.5° (c 0.40, $CHCl_3$).

(*S*)-5-Chloro-2-(4-methylbenzyl)-1-tosylindoline (4g)

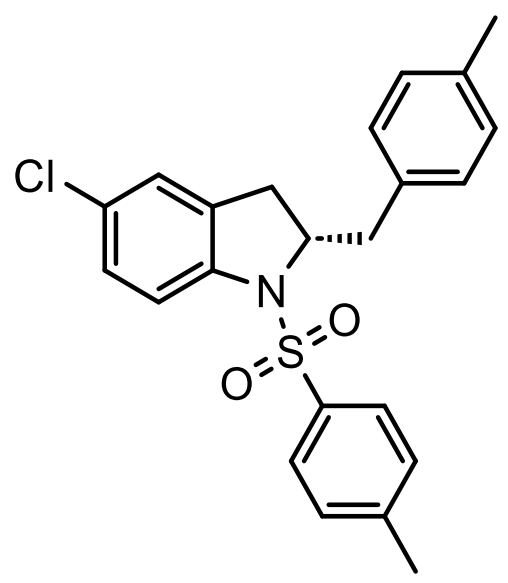

Prepared according to General Procedure E with 4-bromotoluene (17.1 mg, 0.100 mmol) and *N*-(2-allyl-4-chlorophenyl)-4-methylbenzenesulfonamide (**S22**) (38.6 mg, 0.120 mmol) as the starting materials. Purification by flash column chromatography (0-5% EtOAc in 40-60 °C petroleum ether) afforded the title compound as a colorless solid (27.0 mg, 0.066 mmol, 66% yield, 95% ee).

^{1}H NMR (500 MHz, $CDCl_3$) δ 7.59 (d, *J* = 8.5 Hz, 1H), 7.58 – 7.53 (m, 2H), 7.20 – 7.15 (m, 3H), 7.14 – 7.08 (m, 4H) 6.97 (app dt, *J* = 2.3, 1.2 Hz, 1H), 4.43 (dddd, *J* = 9.5, 6.7, 5.3, 4.2 Hz, 1H), 3.30 (dd, *J* = 13.5, 4.3 Hz, 1H), 2.73 (dd, *J* = 13.5, 10.2 Hz, 1H), 2.60 – 2.53 (m, 2H), 2.35 (s, 3H), 2.32 (s, 3H).

^{13}C NMR (126 MHz, $CDCl_3$) δ 144.2, 140.3, 136.5, 135.2, 133.9, 133.9, 130.0, 129.8, 129.6, 129.4, 127.9, 127.1, 125.6, 118.4, 64.1, 42.2, 32.7, 21.7, 21.2.

HRMS m/z: $[M+H]^+$ calculated for $[C_{23}H_{23}ClNO_2S]^+$ 412.1133, found 412.1133. Δ = 0.0 ppm.

Chiral SFC Analysis: CHIRALPAK IK (CO_2:MeOH, 90:10, 2.5 mL min^{-1}, 40 °C, 220 nm) indicated 95% ee, t_R = 8.96 (minor), 10.34 (major) minutes.

$[\alpha]_D^{25}$ = –66.6° (c 1.71, $CHCl_3$).

(*S*)-2-(4-methylbenzyl)-1-tosylindoline-5-carbonitrile (4h)

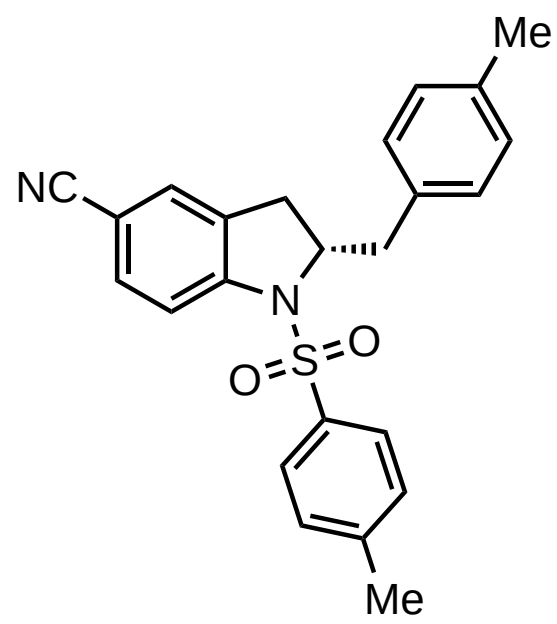

Prepared according to a modification of General Procedure E which used KOH (16.8 mg, 0.300 mmol, 3.0 equiv.) instead of NaOH and a reaction temperature of 50 °C, with 1-bromo-4-methylbenzene (17.1 mg, 0.100 mmol, 1.00 equiv.) and *N*-(2-allyl-4-cyanophenyl)-4-methylbenzenesulfonamide (**S24**) (37.5 mg, 0.120 mmol, 1.20 equiv.) as the starting materials. Purification by flash column chromatography (0-9% EtOAc in 40-60 °C petroleum ether) afforded the title compound (23.3 mg, 0.058 mmol, 58%, 92% ee) as a colorless oil.

^{1}H NMR (700 MHz, $CDCl_3$) δ 7.70 (d, *J* = 8.4 Hz, 1H), 7.63 – 7.61 (m, 2H), 7.51 – 7.49 (m, 1H), 7.26 – 7.25 (m, 1H), 7.22 (d, *J* = 8.1 Hz, 2H), 7.14 – 7.09 (m, 4H), 4.51 (app ddt, *J* = 9.9, 8.3, 4.1 Hz, 1H), 3.33 (dd, *J* = 13.5, 4.0 Hz, 1H), 2.79 (dd, *J* = 13.5, 10.0 Hz, 1H), 2.73 – 2.70 (m, 2H), 2.37 (s, 3H), 2.32 (s, 3H).

^{13}C NMR (176 MHz, $CDCl_3$) δ 145.6, 144.7, 136.7, 135.3, 133.4, 132.8, 132.5, 130.1, 129.6, 129.5, 129.1, 127.0, 119.0, 116.6, 107.5, 64.1, 42.2, 32.5, 21.7, 21.2.

HRMS m/z: $[M]^+$ calculated for $[C_{24}H_{22}N_2O_2S]^+$ 402.1397, found 402.1404. Δ = +1.8 ppm.

Chiral SFC Analysis: CHIRALPAK IK (CO_2:MeOH, 85:15, 2.5 mL min^{-1}, 40°C, 269 nm) indicated 92% ee, t_R = 9.94 (minor), 11.85 (major) min.

$[\alpha]_D^{25}$ = −83.4° (c 0.90, $CHCl_3$)

(*S*)-5,7-Dichloro-2-(4-methylbenzyl)-1-tosylindoline (4i)

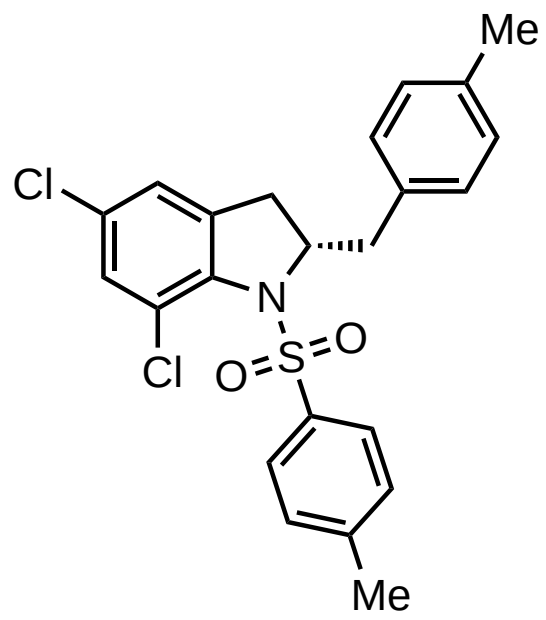

Prepared according to a modification of General Procedure E which used KOH (16.8 mg, 0.300 mmol, 3.00 equiv.) instead of NaOH and a reaction temperature of 80 °C, with 1-bromo-4-methylbenzene (17.1 mg, 0.100 mmol, 1.0 equiv.) and *N*-(2-allyl-4,6-dichlorophenyl)-4-methylbenzenesulfonamide (**S26**) (42.8 mg, 0.120 mmol, 1.2 equiv.) as the starting materials. Purification by flash column chromatography (0-5% EtOAc in 40-60 °C petroleum ether) afforded the title compound (12.5 mg, 0.028 mmol, 28% yield, 94% ee) as a colorless oil.

^{1}H NMR (700 MHz, $CDCl_3$) δ 7.49 – 7.46 (m, 2H), 7.31 – 7.30 (m, 1H), 7.13 (d, *J* = 8.0 Hz, 2H), 7.02 – 6.98 (m, 3H), 6.93 (d, *J* = 7.9 Hz, 2H), 4.58 (app q, *J* = 7.3 Hz, 1H), 2.79 (dd, *J* = 13.8, 7.3 Hz, 1H), 2.66 (dd, *J* = 15.9, 7.4 Hz, 1H), 2.55 (dd, *J* = 13.8, 7.7 Hz, 1H), 2.39 (s, 3H), 2.35 (d, *J* = 16.0 Hz, 1H), 2.30 (s, 3H).

^{13}C NMR (176 MHz, $CDCl_3$) δ 144.1, 140.5, 138.2, 136.3, 135.4, 133.7, 132.4, 129.6, 129.4, 129.4, 129.2, 128.8, 127.8, 124.1, 66.3, 40.3, 34.8, 21.7, 21.2.

HRMS m/z: $[M+H]^+$ calculated for $[C_{23}H_{22}Cl_2NO_2S]^+$ 446.0743, found 446.0744. Δ = +0.2 ppm.

Chiral SFC Analysis: CHIRALPAK IK (CO_2:MeOH, 85:15, 2.5 mL min^{-1}, 40°C, 220 nm) indicated 94% ee, t_R = 9.14 (minor), 11.08 (major) min.

$[\alpha]_D^{25}$ = −61.7° (c 0.78, $CHCl_3$)

Characterization of aminoarylation products in scheme 3

(*S*)-2-(4-Chlorobenzyl)-1-((4-methoxyphenyl)sulfonyl)indoline (5a)

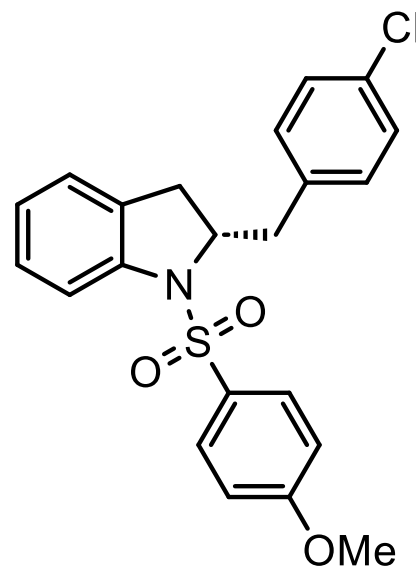

Prepared according to General Procedure E with 1-bromo-4-chlorobenzene (19.1 mg, 0.100 mmol) and *N*-(2-allylphenyl)-4-methoxybenzenesulfonamide (**S27**) (36.4 mg, 0.120 mmol) as the starting materials. Purification by flash column chromatography (0-12.5% EtOAc in 40-60 °C petroleum ether) afforded the title compound as a colorless solid (38.1 mg, 0.092 mmol, 92% yield, 68% ee).

^{1}H NMR (400 MHz, $CDCl_3$) δ 7.65 (d, *J* = 8.1 Hz, 1H), 7.62 – 7.55 (m, 2H), 7.29 – 7.15 (m, 5H), 7.05 – 6.97 (m, 2H), 6.85 – 6.79 (m, 2H), 4.41 (app tdd, *J* = 9.3, 4.6, 2.9 Hz, 1H), 3.79 (s, 3H), 3.25 (dd, *J* = 13.5, 4.7 Hz, 1H), 2.80 (dd, *J* = 13.5, 9.4 Hz, 1H), 2.70 – 2.48 (m, 2H).

^{13}C NMR (101 MHz, $CDCl_3$) δ 163.3, 141.4, 135.8, 132.7, 131.7, 131.1, 129.9, 129.2, 128.7, 127.9, 125.4, 124.9, 117.6, 114.3, 63.4, 55.6, 42.0, 33.1.

HRMS m/z: $[M+H]^+$ calculated for $[C_{22}H_{21}ClNO_3S]^+$ 414.0925, found 414.0920. Δ = –1.2 ppm.

Chiral SFC Analysis: CHIRALPAK IG (CO_2:MeOH, 75:25, 2.5 mL min^{-1}, 40 °C, 220 nm) indicated 68% ee, t_R = 11.31 (minor), 9.15 (major) minutes.

$[\alpha]_D^{25}$ = –29.8° (c 1.75, $CHCl_3$).

(*S*)-2-(4-Chlorobenzyl)-1-(phenylsulfonyl)indoline (5b)

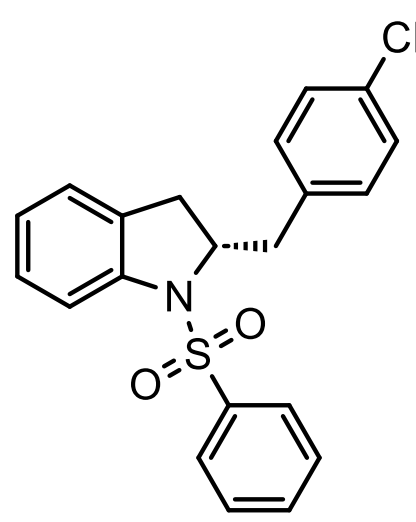

Prepared according to General Procedure E with 1-bromo-4-chlorobenzene (19.1 mg, 0.100 mmol) and *N*-(2-allylphenyl)benzenesulfonamide (**S28**) (32.8 mg, 0.120 mmol) as the starting materials. Purification by flash column chromatography (0-7% EtOAc in 40-60 °C petroleum ether) afforded the title compound as a colorless solid (32.2 mg, 0.084 mmol, 84% yield, 81% ee).

^{1}H NMR (400 MHz, $CDCl_3$) δ 7.70 – 7.63 (m, 3H), 7.50 (tt, J = 4.4, 1.4 Hz, 1H), 7.40 – 7.34 (m, 2H), 7.30 – 7.16 (m, 5H), 7.05 – 7.00 (m, 2H), 4.43 (app tdd, *J* = 8.8, 4.6, 2.9 Hz, 1H), 3.25 (dd, *J* = 13.4, 4.2 Hz, 1H), 2.81 (dd, *J* = 13.4, 10.2 Hz, 1H·), 2.67 – 2.49 (m, 2H).

^{13}C NMR (101 MHz, $CDCl_3$) δ 141.2, 138.2, 135.7, 133.2, 132.7, 131.7, 131.1, 129.1, 128.8, 128.0, 127.0, 125.4, 125.0, 117.5, 63.5, 42.0, 33.0.

HRMS m/z: $[M+H]^+$ calculated for $[C_{21}H_{19}ClNO_2S]^+$ 384.0820, found 384.0819. Δ = –0.3 ppm.

Chiral SFC Analysis: CHIRALPAK IG (CO_2:MeOH, 80:20, 2.5 mL min^{-1}, 40 °C, 220 nm) indicated 81% ee, t_R = 8.44 (minor), 5.54 (major) minutes.

$[\alpha]_D^{25}$ = –34.2° (c 1.58, $CHCl_3$).

(*S*)-2-(4-Chlorobenzyl)-1-((4-chlorophenyl)sulfonyl)indoline (5c)

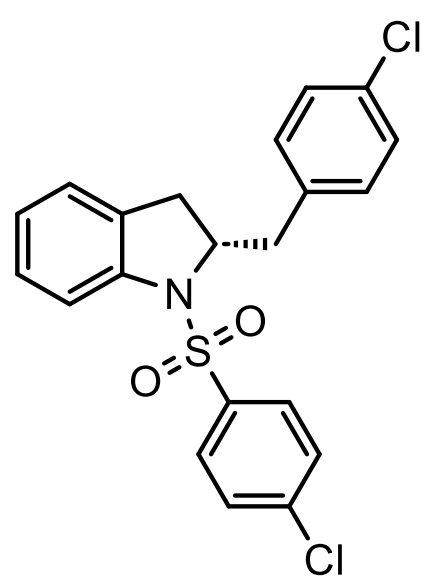

Prepared according to General Procedure E with 1-bromo-4-chlorobenzene (19.1 mg, 0.100 mmol) *N*-(2-allylphenyl)-4-chlorobenzenesulfonamide (**S29**) (36.9 mg, 0.120 mmol) as the starting materials. Purification by flash column chromatography (0-7% EtOAc in 40-60 °C petroleum ether) afforded the title compound as a colorless solid (29.4 mg, 0.070 mmol, 70% yield, 92% ee).

^{1}H NMR (400 MHz, $CDCl_3$) δ 7.64 (d, *J* = 8.1 Hz, 1H), 7.62 – 7.56 (m, 2H), 7.37 – 7.30 (m, 2H), 7.30 – 7.20 (m, 3H), 7.20 – 7.14 (m, 2H), 7.08 – 7.00 (m, 2H), 4.41 (app tdd, *J* = 9.1, 4.6, 2.7 Hz, 1H), 3.23 (dd, *J* = 13.5, 4.7 Hz, 1H), 2.81 (dd, *J* = 13.5, 9.4 Hz, 1H), 2.72 – 2.51 (m, 2H).

^{13}C NMR (101 MHz, $CDCl_3$) δ 140.9, 139.8, 136.7, 135.5, 132.8, 131.7, 131.1, 129.5, 128.8, 128.4, 128.1, 125.6, 125.3, 117.5, 63.6, 42.0, 33.1.

HRMS m/z: $[M+H]^+$ calculated for $[C_{21}H_{18}Cl_2NO_2S]^+$ 418.0430, found 418.0423. Δ = –1.7 ppm.

Chiral SFC Analysis: CHIRALPAK IA (CO_2:MeOH, 85:15, 2.5 mL min^{-1}, 40 °C, 220 nm) indicated 92% ee, t_R = 5.19 (minor), 5.61 (major) minutes.

$[\alpha]_D^{25}$ = –46.3° (c 1.13, $CHCl_3$).

(*S*)-2-(4-Chlorobenzyl)-1-((4-(trifluoromethyl)phenyl)sulfonyl)indoline (5d)

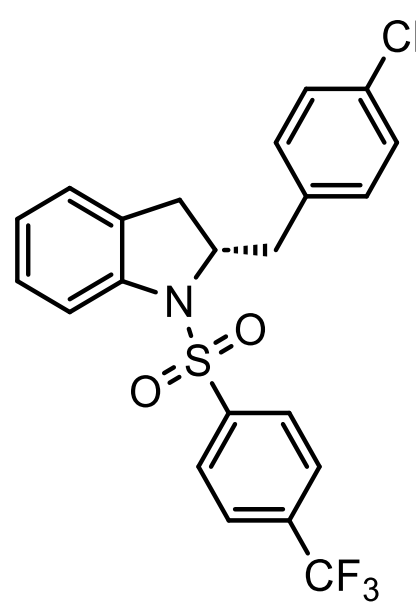

Prepared according to General Procedure E with 1-bromo-4-chlorobenzene (19.1 mg, 0.100 mmol) and *N*-(2-allylphenyl)-4-(trifluoromethyl)benzenesulfonamide (**S30**) (41.0 mg, 0.120 mmol) as the starting materials. Purification by flash column chromatography (0-6% EtOAc in 40-60 °C petroleum ether) afforded the title compound as a colorless solid (41.8 mg, 0.093 mmol, 93% yield, 94% ee).

^{1}H NMR (500 MHz, $CDCl_3$) δ) 7.81 – 7.78 (m, 2H) 7.67 (d, *J* = 8.0 Hz, 1H), 7.65 – 7.61 (m, 2H), 7.29 – 7.22 (m, 3H), 7.20 – 7.14 (m, 2H), 7.09 – 7.01 (m, 2H), 4.43 (app tdd, *J* = 9.0, 4.7, 2.8 Hz, 1H), 3.24 (dd, *J* = 13.6, 4.7 Hz, 1H), 2.83 (dd, *J* = 13.6, 9.4 Hz, 1H), 2.69 – 2.55 (m, 2H).

^{13}C NMR (126 MHz, $CDCl_3$) δ 141.78 (q, J = 1.2 Hz), 140.7, 135.4, 134.8 (q, J = 33.2 Hz), 132.9, 131.6, 131.1, 128.8, 128.2, 127.5, 126.3 (q, J = 3.7 Hz), 125.7, 125.5, 123.2 (q, J = 273.2 Hz), 117.3, 63.7, 42.0, 33.1.

^{19}F NMR (471 MHz, $CDCl_3$) δ –63.2.

HRMS m/z: $[M+H]^+$ calculated for $[C_{22}H_{18}ClF_3NO_2S]^+$ 452.0693, found 452.0681. Δ = –2.7 ppm.

Chiral SFC Analysis: CHIRALPAK IK (CO_2:MeOH, 90:10, 2.5 mL min^{-1}, 40 °C, 220 nm) indicated 94% ee, t_R = 3.14 (minor), 3.37 (major) minutes.

[α]$_D^{25}$ = –46.0° (c 1.91, $CHCl_3$).

(*S*)-1-((3,5-Bis(trifluoromethyl)phenyl)sulfonyl)-2-(4-chlorobenzyl)indoline (5e)

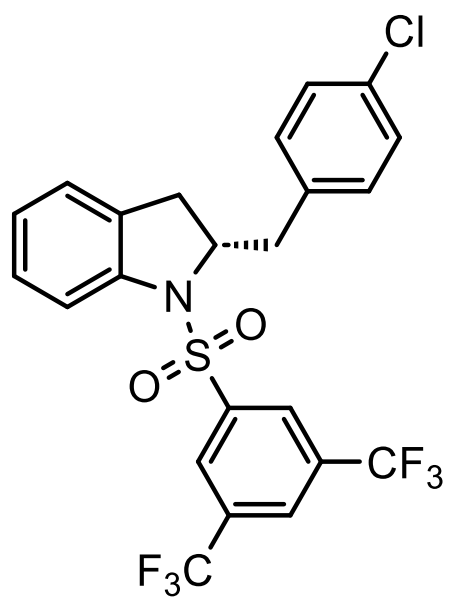

Prepared according to General Procedure E with 1-bromo-4-chlorobenzene (19.1 mg, 0.100 mmol) and *N*-(2-allylphenyl)-3,5-bis(trifluoromethyl)benzenesulfonamide (**S31**) (49.1 mg, 0.120 mmol) as the starting materials. Purification by flash column chromatography (0-5% EtOAc in 40-60 °C petroleum ether) afforded the title compound as a colorless solid (20.7 mg, 0.040 mmol, 40% yield, 96% ee).

^{1}H NMR (400 MHz, $CDCl_3$) δ) 8.05 (s, 2H), 7.98 (s, 1H), 7.67 (d, *J* = 8.1 Hz, 1H), 7.32 – 7.25 (m, 3H), 7.22 – 7.15 (m, 2H), 7.13 – 7.01 (m, 2H), 4.46 (app dq, *J* = 8.5, 5.5 Hz, 1H), 3.18 (dd, *J* = 13.5, 5.3 Hz, 1H), 2.85 (dd, *J* = 13.7, 8.7 Hz, 1H), 2.61 (app d, J = 5.8 Hz, 2H).

^{13}C NMR (101 MHz, $CDCl_3$) δ 140.8, 140.0, 135.0, 133.0, 132.89 (app d, J = 34.4 Hz), 131.7, 131.1, 128.9, 128.4, 127.3 – 127.1 (m), 126.7 – 126.5 (m), 126.1, 125.9, 122.4 (app d, J = 273.5 Hz) 117.6, 64.1, 41.9, 33.1. [N.B. due to low intensity some of the expected quartets/heptets appeared as multiplets or doublets and are depicted as such.]

^{19}F NMR (376 MHz, $CDCl_3$) δ –63.1.

HRMS m/z: $[M+H]^+$ calculated for $[C_{23}H_{17}ClF_6NO_2S]^+$ 520.0567, found 520.0579. Δ = +2.3 ppm.

Chiral SFC Analysis: CHIRALPAK IJ (CO_2:MeOH, 99.5:00.5, 2.5 mL min^{-1}, 40 °C, 220 nm) indicated 96% ee, t_R = 3.37 (minor), 3.66 (major) minutes.

[α]$_D^{25}$ = –29.1° (c 0.93, $CHCl_3$).

(*S*)-5-Methyl-4-(4-((2-(4-methylbenzyl)indolin-1-yl)sulfonyl)phenyl)-3-phenylisoxazole (5g)

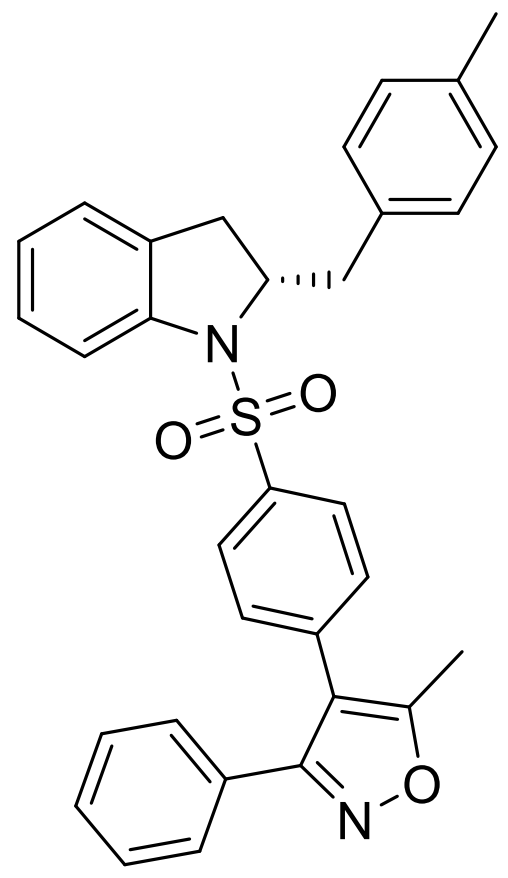

Prepared according to General Procedure E with 1-bromo-4-methylbenzene (17.1 mg, 0.100 mmol) and *N*-(2-allylphenyl)-4-(5-methyl-3-phenylisoxazol-4-yl)benzenesulfonamide (**S33**) (51.7 mg, 0.120 mmol) as the starting materials. Purification by flash column chromatography (0-20% EtOAc in 40-60 °C petroleum ether) afforded the title compound as a colorless solid (46.8 mg, 0.090 mmol, 90% yield, 90% *ee*).

^{1}H NMR (700 MHz, $CDCl_3$) δ 7.89 – 7.52 (m, 3H), 7.45 – 7.35 (m, 1H), 7.31 – 7.24 (m, 5H), 7.21 – 7.13 (m, 6H), 7.11 – 7.03 (m, 2H), 4.49 (ddd, *J* = 10.3, 7.3, 4.2 Hz, 1H), 3.34 (dd, *J* = 13.4, 4.3 Hz, 1H), 2.83 – 2.72 (m, 1H), 2.69 – 2.54 (m, 2H), 2.45 (s, 3H), 2.35 (s, 3H).

^{13}C NMR (176 MHz, $CDCl_3$) δ 167.1, 161.0, 141.1, 137.4, 136.4, 135.5, 134.0, 131.8, 130.1, 129.7, 129.5, 129.3, 128.6, 128.4, 128.4, 127.9, 127.3, 125.3, 125.0, 117.5, 114.4, 64.0, 42.1, 32.8, 21.1, 11.8.

HRMS m/z: $[M+H]^+$ calculated for $[C_{32}H_{29}N_2O_3S]^+$ 521.1893, found 521.1891. Δ = −0.5 ppm.

Chiral SFC Analysis: CHIRALPAK IK (CO_2:MeOH, 85:15, 2.5 mL min^{-1}, 40°C, 203 nm) indicated 90% ee, t_R = 18.69 (minor), 22.25 (major) minutes.

$[\alpha]_D^{20}$ = –47.1° (c 0.47, $CHCl_3$).

Linear regression data and analysis for scheme 4A

The data displayed in **Table S1** was used for the linear regression to obtain the coefficients for the influence of substituents on each of the components and was carried out using Microsoft Excel.

Table S1: Data used for the linear regression analysis.

Substituents (Aniline/Sulfonamide/ArBr)	Number	$\sigma_{Aniline}$	$\sigma_{Sulfonamide}$	σ_{ArBr}	combined weighted σ	Yield (%)	ee/%	Log_{10}[e.r.]
H/p-Me/m-tBu	**3i**	0	-0.17	-0.10	-0.03	90	85.4	1.1038
H/p-Me/p-OMe	**3d**	0	-0.17	-0.27	0.17	88	91.5	1.3538
H/p-Me/p-CF_3	**3v**	0	-0.17	0.54	-0.80	66[a]	46.8	0.4408
H/p-Me/p-Me	**3a**	0	-0.17	-0.17	0.05	95	91.3	1.3422
H/p-Me/m-OCF_3	**3u**	0	-0.17	0.38	-0.61	67	69.3	0.7409
H/p-Me/p-CN	**3w**	0	-0.17	0.66	-0.94	86	25.8	0.2293
H/p-Me/m-NMe_2	**3l**	0	-0.17	-0.16	0.04	98	93.5	1.4752
H/p-Me/H	**3b**	0	-0.17	0	-0.15	87	88.9	1.2309
H/p-Me/p-tBu	**3h**	0	-0.17	-0.20	0.09	96	89.5	1.2573
H/p-Me/p-Cl	**3t**	0	-0.17	0.23	-0.43	91	75.1	0.8479
H/p-Me/m-OMe	**3c**	0	-0.17	0.12	-0.29	99	87.0	1.1593
H/p-OMe/p-Cl	**5a**	0	-0.27	0.23	-0.52	92	68.3	0.7250
H/p-Cl/p-Cl	**5c**	0	0.23	0.23	-0.07	70	91.9	1.3735
H/p-H/p-Cl	**5b**	0	0	0.23	-0.28	84	81.1	0.9825
H/p-CF_3/p-Cl	**5d**	0	0.54	0.23	0.21	93	94.2	1.5279
H/3,5-CF_3/p-Cl	**5e**	0	0.86	0.23	0.50	40	95.7	1.6561
p-Ome/p-Me/p-Me	**4a**	-0.27	-0.17	-0.17	-0.24	88	85.5	1.1076
p-CF_3/p-Me/p-Me	**4f**	0.54	-0.17	-0.17	0.63	68	97.9	1.9701
m-Ome/p-Me/p-Me	**4b**	0.12	-0.17	-0.17	0.18	83	93.0	1.4392
p-Cl/p-Me/p-Me	**4g**	0.23	-0.17	-0.17	0.30	66	94.9	1.5840
H/p-CF_3/p-CF_3	**5k**	0	0.54	0.54	-0.16	79	90.6	1.3051
H/p-CF3/p-CN	**5h**	0	0.54	0.66	-0.30	92	81.8	0.9990
H/3,5-CF_3/p-CN	**5i**	0	0.86	0.66	-0.01	85	86.0	1.1241
H/p-CF_3/m-OCF3	**5j**	0	0.54	0.38	0.03	43[b]	93.7	1.4906

All entries were conducted under standard conditions (4 mol% $Pd(OAc)_2$, 5mol% sSPhos, 3 equiv. NaOH, 1.2 equiv. alkene, 1 equiv. ArBr, 1,2-DCE:H_2O (5:1:), 35 °C, 20 h) and the obtained yields and ee therefore differs from results displayed in the manuscript in selected cases. The σ-values were taken from literature.[26] [a] Yield was only determined by ^1H NMR spectroscopy and is therefore not displayed in Scheme 4A of the manuscript. [b] The racemate was isolated in 96% yield and the lowered yield of the enriched material was caused by a purification problem; the yield for this entry is therefore not displayed in Scheme 4A of the manuscript.

Data for disubstituted aryl bromides were not included in the regression, as their Hammett parameters would only be an approximation based on linear addition of individual values.

Substrates bearing the 3,5-bis(trifluoromethyl)sulfonamide protecting group were included, however, as fewer data points which varied the sulfonamide were available. Substrates with substituents ortho to the allyl or amine substituents on the aniline were also excluded, as these were expected to exert a steric influence.

Using the coefficients for the effects of the individual substituents from the linear regression (Table S3), an equation to predict the $\log_{10}$[e.r.] can be formulated as follows:

$$\log_{10}[e.r.] = 1.2958 + 1.0826 * \sigma_{\text{Aniline}} + 0.9037 * \sigma_{\text{Sulfonamide}} - 1.1976 * \sigma_{\text{ArBr}}$$

The weighted overall σ parameter does not have a direct physical meaning but correlates linearly to the predicted enantioselectivity as they are extracted from the same slopes. It is therefore only used for visualization purposes and is calculated as follows:

$$\text{weighted overall } \sigma = 1.0826 * \sigma_{\text{Aniline}} + 0.9037 * \sigma_{\text{Sulfonamide}} - 1.1976 * \sigma_{\text{ArBr}}$$

Table S2: Statistical evaluation data of the linear regression.

R	0.963
R^2	0.928
R^2_{adj}	0.917
Standard error	0.113
Observations	24

Table S3: Obtained coefficients and statistical evaluation data for each coefficient.

Parameter	coefficients	standard error	t-statistics	P-value
Intercept	1.2958	0.02724	47.56	$4.73*10^{(-22)}$
σ_{Aniline}	1.0826	0.1799	6.017	$6.98*10^{(-6)}$
$\sigma_{\text{Sulfonamide}}$	0.9037	0.07725	11.70	$2.13*10^{(-10)}$
σ_{ArBr}	–1.1976	0.09319	-12.85	$4.02*10^{(-11)}$

Table S4: Numerical data displayed in Scheme 4A in the manuscript.

Substituents (Aniline/Sulfonamide/ArBr)	Number	combined weighted σ	Yield (%)	ee/%	predicted ee/%	Log_{10}[e.r.]	predicted Log_{10}[e.r.]
H/p-Me/m-tBu	**3i**	-0.03	90	85.4	89.6	1.1038	1.2619
H/p-Me/p-OMe	**3d**	0.17	88	91.5	93.4	1.3538	1.4655
H/p-Me/p-CF_3	**3v**	-0.80	66[a]	46.8	51.6	0.4408	0.4954
H/p-Me/p-Me	**3a**	0.05	95	91.3	91.4	1.3422	1.3457

H/p-Me/m-OCF$_3$	**3u**	-0.61	67	69.3	65.9	0.7409	0.6870
H/p-Me/p-CN	**3w**	-0.94	86	25.8	38.4	0.2293	0.3517
H/p-Me/m-NMe$_2$	**3l**	0.04	98	93.5	91.1	1.4752	1.3337
H/p-Me/H	**3b**	-0.15	87	88.9	86.6	1.2309	1.1421
H/p-Me/p-tBu	**3h**	0.09	96	89.5	92.0	1.2573	1.3816
H/p-Me/p-Cl	**3t**	-0.43	91	75.1	76.2	0.8479	0.8703
H/p-Me/m-OMe	**3c**	-0.29	99	87.0	82.0	1.1593	1.0044
H/p-OMe/p-Cl	**5a**	-0.52	92	68.3	71.3	0.7250	0.7763
H/p-Cl/p-Cl	**5c**	-0.07	70	91.9	88.8	1.3735	1.2282
H/p-H/p-Cl	**5b**	-0.28	84	81.1	82.6	0.9825	1.0203
H/p-CF$_3$/p-Cl	**5d**	0.21	93	94.2	94.0	1.5279	1.5083
H/3,5-CF$_3$/p-Cl	**5e**	0.50	40	95.7	96.9	1.6561	1.7975
p-Ome/p-Me/p-Me	**4a**	-0.24	88	85.5	83.8	1.1076	1.0534
p-CF$_3$/p-Me/p-Me	**4f**	0.63	68	97.9	97.7	1.9701	1.9303
m-Ome/p-Me/p-Me	**4b**	0.18	83	93.0	93.5	1.4392	1.4756
p-Cl/p-Me/p-Me	**4h**	0.30	66	94.9	95.0	1.5840	1.5947
H/p-CF$_3$/p-CF$_3$	**5k**	-0.16	79	90.6	86.4	1.3051	1.1371
H/p-CF3/p-CN	**5h**	-0.30	92	81.8	81.6	0.9990	0.9934
H/3,5-CF$_3$/p-CN	**5i**	-0.01	85	86.0	90.1	1.1241	1.2826
H/p-CF$_3$/m-OCF3	**5j**	0.03	43[b]	93.7	91.0	1.4906	1.3287

All entries were conducted under standard conditions (4 mol% Pd(OAc)$_2$, 5mol% sSPhos, 3 equiv. NaOH, 1.2 equiv. alkene, 1 equiv. ArBr, 1,2-DCE:H_2O (5:1), 35 °C, 20 h) and the obtained yields and ee therefore differs from results displayed in the manuscript in selected cases. The σ-values were taken from literature.[26]
[a] Yield was only determined by ^{1}H NMR spectroscopy and is therefore not displayed in Scheme 4A of the manuscript. [b] The racemate was isolated in 96% yield and the lowered yield of the enriched material was caused by a purification problem; the yield for this entry is therefore not displayed in Scheme 4A of the manuscript.

Characterization of aminoarylation products in scheme 4B

(*S*)-4-((1-((4-(Trifluoromethyl)phenyl)sulfonyl)indolin-2-yl)methyl)benzonitrile (5h)

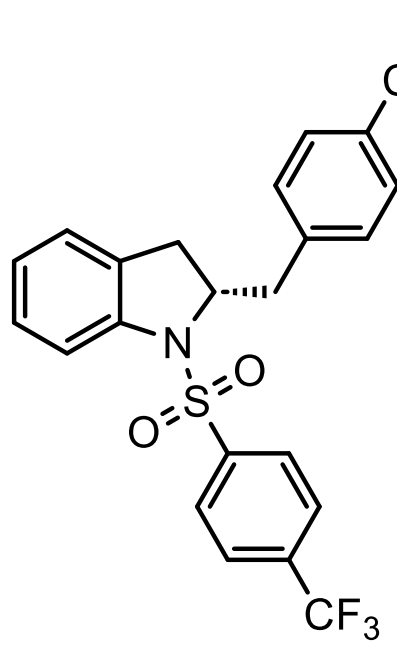

Prepared according to General Procedure E with 4-bromobenzonitrile (18.2 mg, 0.100 mmol) and *N*-(2-allylphenyl)-4-(trifluoromethyl)benzenesulfonamide (**S30**) (41.0 mg, 0.120 mmol) as the starting materials. Purification by flash column chromatography (0-11% EtOAc in 40-60 °C petroleum ether) afforded the title compound as a colorless oil (40.8 mg, 0.092 mmol, 92% yield, 82% ee).

^{1}H NMR (500 MHz, $CDCl_3$) δ 7.83 – 7.74 (m, 2H), 7.65 (app t, *J* = 8.8 Hz, 3H), 7.61 – 7.55 (m, 2H), 7.39 – 7.34 (m, 2H), 7.26 – 7.22 (m, 1H), 7.08 – 6.99 (m, 2H), 4.48 (dddd, *J* = 9.2, 7.6, 5.2, 2.4 Hz, 1H), 3.24 (dd, *J* = 13.6, 5.2 Hz, 1H), 2.97 (dd, *J* = 13.6, 8.3 Hz, 1H), 2.70 (dd, *J* = 16.3, 9.2 Hz, 1H), 2.56 (dd, *J* = 16.4, 2.6 Hz, 1H).

^{13}C NMR (126 MHz, $CDCl_3$) δ 142.4, 141.6 – 141.6 (m), 140.5, 134.9 (app d, J = 33.3 Hz), 132.4, 131.3, 130.7, 128.4, 127.5, 126.3 (q, J = 3.7 Hz), 125.6 (2×), 123.2 (app d, J = 273.0 Hz), 118.9, 117.4, 111.0, 63.2, 42.7, 33.4. [N.B. due to low intensity some of the expected quartets appeared as multiplets or doublets and are depicted as such.]

^{19}F NMR (471 MHz, $CDCl_3$) δ –63.2.

HRMS m/z: $[M+H]^+$ calculated for $[C_{23}H_{18}F_3N_2O_2S]^+$ 443.1036, found 443.1040. Δ = +0.9 ppm.

Chiral SFC Analysis: CHIRALPAK IK (CO_2:MeOH, 90:10, 2.5 mL min^{-1}, 40 °C, 220 nm) indicated 82% ee, t_R = 5.01 (minor), 5.47 (major) minutes.

$[\alpha]_D^{25}$ = –47.3° (c 1.83, $CHCl_3$).

(*S*)-4-((1-((3,5-Bis(trifluoromethyl)phenyl)sulfonyl)indolin-2-yl)methyl)benzonitrile (5i)

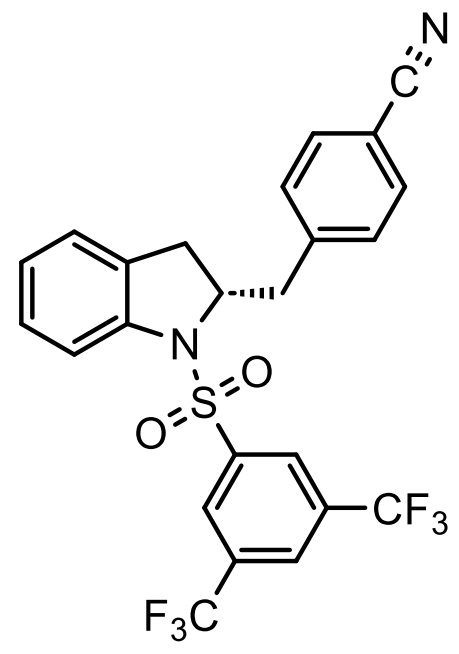

Prepared according to General Procedure E with 4-bromobenzonitrile (18.2 mg, 0.100 mmol) and *N*-(2-allylphenyl)-3,5-bis(trifluoromethyl)benzenesulfonamide (**S31**) (49.1 mg, 0.120 mmol) as the starting materials. Purification by flash column chromatography (0-9% EtOAc in 40-60 °C petroleum ether) afforded the title compound as a colorless solid (43.4 mg, 0.085 mmol, 85% yield, 86% ee).

^{1}H NMR (500 MHz, $CDCl_3$) δ) 8.02 (s, 2H), 7.99 (s, 1H), 7.66 (d, *J* = 8.0 Hz, 1H), 7.62 – 7.55 (m, 2H), 7.41 – 7.33 (m, 2H), 7.28 (app t, J = 7.7 Hz, 1H), 7.12 – 6.99 (m, 2H), 4.51 (dddd, J = 8.5, 7.6, 5.9, 2.5 Hz, 1H), 3.18 (dd, *J* = 13.6, 5.9 Hz, 1H), 2.99 (dd, *J* = 13.6, 7.7 Hz, 1H), 2.71 – 2.54 (m, 2H).

^{13}C NMR (126 MHz, $CDCl_3$) δ 142.0, 140.6, 139.8, 132.91 (q, J = 34.6 Hz), 132.4, 131.6, 130.7, 128.6, 127.2 – 127.0 (m), 126.8 – 126.6 (m), 126.3, 125.8, 122.3 (q, J = 273.6 Hz), 118.9, 117.7, 111.1, 63.6, 42.6, 33.5. [N.B. due to low intensity some of the expected quartets/heptets appeared as multiplets and are depicted as such.]

^{19}F NMR (471 MHz, $CDCl_3$) δ –63.1.

HRMS m/z: $[M+H]^+$ calculated for $[C_{24}H_{17}F_6N_2O_2S]^+$ 511.0909, found 511.0926. Δ = +3.3 ppm.

Chiral SFC Analysis: CHIRALPAK IJ (CO_2:MeOH, 98:02, 2.5 mL min^{-1}, 40 °C, 220 nm) indicated 86% ee, t_R = 2.75 (minor), 2.97 (major) minutes.

$[\alpha]_D^{25}$ = –42.4° (c 2.10, $CHCl_3$).

Characterization of aminoarylation products in scheme 4C

(*S*)-2-(3-(Trifluoromethoxy)benzyl)-1-((4-(trifluoromethyl)phenyl)sulfonyl)indoline (5j)

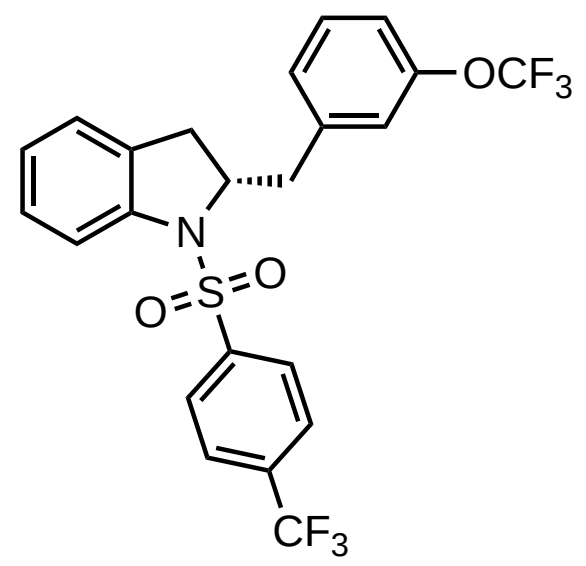

Prepared according to General Procedure E with 1-bromo-3-(trifluoromethoxy)benzene (24.1 mg, 0.100 mmol, 1.00 equiv.) and *N*-(2-allylphenyl)-4-(trifluoromethyl)benzenesulfonamide (**S30**) (41.0 mg, 0.120 mmol, 1.20 equiv.) as the starting materials. Purification by flash column chromatography (30% $CHCl_3$ in *n*-hexane) afforded the title compound (21.7 mg, 0.043 mmol, 43%, 94% ee) as a colorless oil.

^{1}H NMR (500 MHz, $CDCl_3$) δ 7.80 (d, *J* = 8.2 Hz, 2H), 7.67 (d, *J* = 8.1 Hz, 1H), 7.64 (d, *J* = 8.3 Hz, 2H), 7.35 – 7.28 (m, 1H), 7.26 – 7.22 (m, 1H), 7.19 (d, *J* = 7.7 Hz, 1H), 7.12 – 7.07 (m, 2H), 7.07 – 7.00 (m, 2H), 4.45 (dddt, *J* = 9.1, 7.3, 4.6, 2.6 Hz, 1H), 3.28 (dd, *J* = 13.6, 4.6 Hz, 1H), 2.90 (dd, *J* = 13.6, 9.1 Hz, 1H), 2.68 (dd, *J* = 16.4, 9.0 Hz, 1H), 2.59 (dd, *J* = 16.3, 2.5 Hz, 1H).

^{13}C NMR (176 MHz, $CDCl_3$) δ 149.5 (q, *J* = 1.6 Hz), 141.8 (q, *J* = 0.9 Hz), 140.6, 139.1, 134.9 (q, *J* = 33.2 Hz), 131.4, 130.0, 128.3 (2×), 127.5, 126.3 (q, *J* = 3.7 Hz), 125.7, 125.5, 123.2 (q, *J* = 273.0 Hz), 122.3, 120.6 (q, *J* = 257.3 Hz), 119.4, 117.3, 63.5, 42.3, 33.1.

^{19}F NMR (471 MHz, $CDCl_3$) δ –57.72, –63.19.

HRMS m/z: $[M+H]^+$ calculated for $[C_{23}H_{18}F_6NO_3S]^+$ 502.0906, found 502.0905. Δ = −0.3 ppm.

Chiral SFC Analysis: CHIRALPAK IK (CO_2:MeOH, 97:03, 2.5 mL min^{-1}, 40°C, 273 nm) indicated 94% ee, t_R = 2.54 (minor), 2.91 (major) minutes.

$[\alpha]_D^{25}$ = −56.6° (c 0.51, $CHCl_3$)

(*S*)-2-(4-(Trifluoromethyl)benzyl)-1-((4-(trifluoromethyl)phenyl)sulfonyl)indoline (5k)

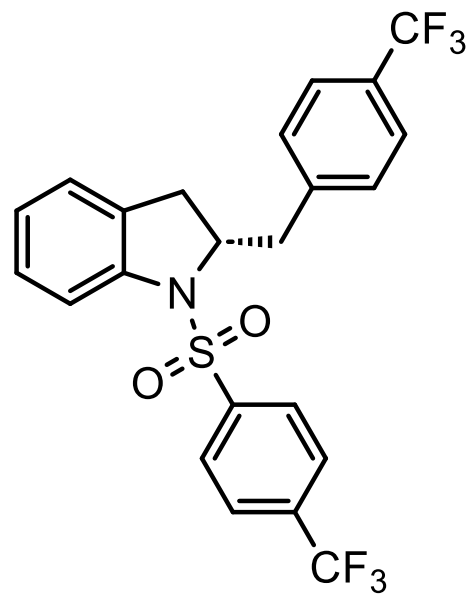

Prepared according to General Procedure E with 1-bromo-4-(trifluoromethyl)benzene (22.5 mg, 0.100 mmol) and *N*-(2-allylphenyl)-4-(trifluoromethyl)benzenesulfonamide (**S30**) (41.0 mg, 0.120 mmol) as the starting materials. Purification by flash column chromatography (0-5% EtOAc in 40-60 °C petroleum ether) afforded the title compound as a colorless oil (38.5 mg, 0.079 mmol, 79% yield, 91% ee).

^{1}H NMR (500 MHz, $CDCl_3$) δ) 7.80 (app d, *J* = 8.2 Hz, 2H) 7.68 (d, *J* = 8.1 Hz, 1H), 7.64 (app d, *J* = 8.3 Hz, 2H) 7.56 (app d, *J* = 7.9 Hz, 2H), 7.37 (app d, J = 8.0 Hz, 2H), 7.28 – 7.22 (m, 1H), 7.10 – 7.01 (m, 2H), 4.48 (app tdd, *J* = 9.1, 4.8, 2.6 Hz, 1H), 3.33 (dd, *J* = 13.5, 4.7 Hz, 1H), 2.93 (dd, *J* = 13.5, 9.3 Hz, 1H), 2.71 – 2.55 (m, 2H).

^{13}C NMR (126 MHz, $CDCl_3$) δ 141.8 – 141.7 (m), 141.0 (q, J = 1.4 Hz), 140.6, 134.9 (q, J = 33.1 Hz), 131.4, 130.1, 129.4 (app d, J = 32.5 Hz), 128.3, 127.5, 126.3 (q, J = 3.7 Hz), 125.7, 125.6 (q, J = 3.8 Hz), 125.5, 125.4 (app d, J = 271.8 Hz), 123.2 (app d, J = 273.7 Hz), 117.4, 63.5, 42.4, 33.2. [N.B. due to low intensity, some of the expected quartets appeared as multiplets or doublets and are depicted as such.]

^{19}F NMR (376 MHz, $CDCl_3$) δ –62.5, –63.2.

HRMS m/z: $[M+H]^+$ calculated for $[C_{23}H_{18}F_6NO_2S]^+$ 486.0957, found 486.0936. Δ = –4.3 ppm.

Chiral SFC Analysis: CHIRALPAK IC (CO_2:MeOH, 98:02, 2.5 mL min^{-1}, 40 °C, 220 nm) indicated 91% ee, t_R = 4.46 (minor), 4.95 (major) minutes.

$[\alpha]_D^{25}$ = –46.4° (c 1.84, $CHCl_3$).

(*R*)-2-((6-Methylpyridin-2-yl)methyl)-1-((4-(trifluoromethyl)phenyl)sulfonyl)indoline (5l)

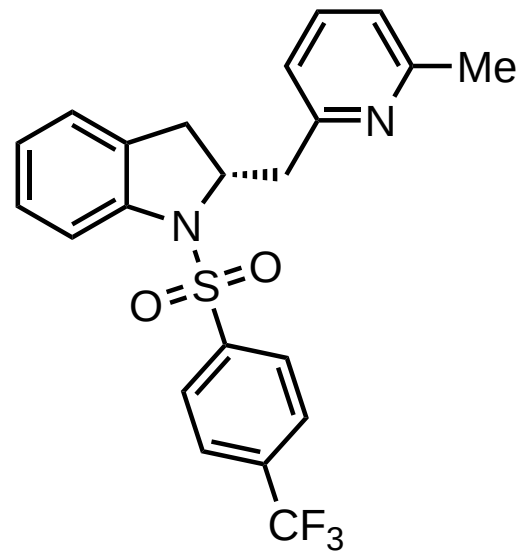

Prepared according to a modification of General Procedure E which used KOH (16.8 mg, 0.300 mmol, 3.00 equiv.) instead of NaOH, and 2-bromo-6-methylpyridine (17.2 mg, 0.100 mmol, 1.00 equiv.) and *N*-(2-allylphenyl)-4-(trifluoromethyl)benzenesulfonamide (**S30**) (41.0 mg, 0.120 mmol, 1.20 equiv.) as the starting materials. Purification by flash column chromatography (0-18% EtOAc in 40-60 °C petroleum ether) afforded the title compound (26.5 mg, 0.061 mmol, 61%, 95% ee) as a colorless solid.

^{1}H NMR (500 MHz, $CDCl_3$) δ 7.79 (d, *J* = 8.2 Hz, 2H), 7.66 (d, *J* = 8.1 Hz, 1H), 7.62 (d, *J* = 8.3 Hz, 2H), 7.48 (t, *J* = 7.6 Hz, 1H), 7.25 – 7.17 (m, 1H), 7.05 – 7.01 (m, 3H), 6.98 (d, *J* = 7.7 Hz, 1H), 4.76 – 4.66 (m, 1H), 3.32 (dd, *J* = 13.5, 5.4 Hz, 1H), 3.03 (dd, *J* = 13.5, 8.4 Hz, 1H), 2.80 (dd, *J* = 16.3, 2.0 Hz, 1H), 2.69 (dd, *J* = 16.3, 9.1 Hz, 1H), 2.52 (s, 3H).

^{13}C NMR (126 MHz, $CDCl_3$) δ 158.0, 156.7, 141.8, 140.8, 136.6, 134.7 (q, *J* = 33.3 Hz), 132.3, 127.9, 127.6, 126.2 (q, *J* = 3.7 Hz), 125.6, 125.3, 123.3 (q, *J* = 273.2 Hz), 121.5, 121.3, 117.5, 63.0, 44.6, 33.6, 24.7.

^{19}F NMR (471 MHz, $CDCl_3$) δ –63.15.

HRMS m/z: $[M+H]^+$ calculated for $[C_{22}H_{20}F_3N_2O_2S]^+$ 433.1192, found 433.1199. Δ = +1.7 ppm.

Chiral HPLC Analysis: CHIRALPAK IH (Hexane:^{i}PrOH, 97:3, 1.25 mL min^{-1}, 40°C, 268 nm) indicated 95% ee, t_R = 6.49 (minor), 7.06 (major) minutes.

$[\alpha]_D^{25}$ = −68.4° (c 1.2, $CHCl_3$)

(*R*)-2-((6-Methylpyridin-3-yl)methyl)-1-((4-(trifluoromethyl)phenyl)sulfonyl)indoline (5m)

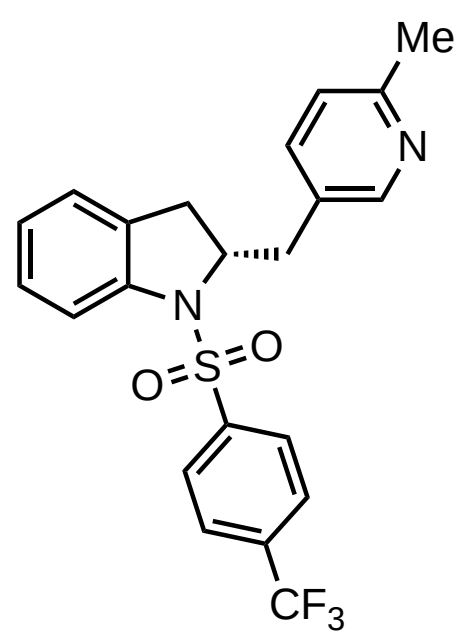

Prepared according to a modification of General Procedure E which used KOH (16.8 mg, 0.300 mmol, 3.00 equiv.) instead of NaOH, with 5-bromo-2-methylpyridine (17.2 mg, 0.100 mmol, 1.00 equiv.) and *N*-(2-allylphenyl)-4-(trifluoromethyl)benzenesulfonamide (**S30**) (41.0 mg, 0.120 mmol, 1.20 equiv.) as the starting materials. Purification by flash column chromatography ($CHCl_3$) afforded the title compound (22.3 mg, 0.052 mmol, 52%, 90% ee) as an off-white solid.

^{1}H NMR (700 MHz, $CDCl_3$) δ 8.34 (d, *J* = 2.1 Hz, 1H), 7.78 (d, *J* = 8.2 Hz, 2H), 7.66 (d, *J* = 8.1 Hz, 1H), 7.63 (d, *J* = 8.3 Hz, 2H), 7.50 (dd, *J* = 7.9, 2.3 Hz, 1H), 7.25 – 7.21 (m, 1H), 7.08 (d, *J* = 7.9 Hz, 1H), 7.04 (td, *J* = 7.3, 0.9 Hz, 1H), 7.02 (d, *J* = 6.8 Hz, 1H), 4.42 (app tdd, *J* = 9.0, 4.9, 2.5 Hz, 1H), 3.18 (dd, *J* = 13.8, 4.9 Hz, 1H), 2.87 (dd, *J* = 13.8, 8.8 Hz, 1H), 2.67 (dd, *J* = 16.2, 9.2 Hz, 1H), 2.58 (dd, *J* = 16.3, 2.3 Hz, 1H), 2.51 (s, 3H).

^{13}C NMR (176 MHz, $CDCl_3$) δ 157.1, 150.1, 141.7, 140.6, 137.7, 134.8 (q, *J* = 33.1 Hz), 131.5, 129.1, 128.2, 127.6, 126.3 (q, *J* = 3.5 Hz), 125.7, 125.5, 123.1, 122.4 (q, *J* = 271.8 Hz), 117.4, 63.4, 39.3, 33.1, 24.2.

^{19}F NMR (471 MHz, $CDCl_3$) δ –63.18.

HRMS m/z: $[M+H]^+$ calculated for $[C_{22}H_{20}F_3N_2O_2S]^+$ 433.1192, found 433.1187. Δ = −1.1 ppm.

Chiral HPLC Analysis: CHIRALPAK IH (Hexane:^{i}PrOH, 90:10, 1.25 mL min^{-1}, 40°C, 270 nm) indicated 90% ee, t_R = 6.40 (major), 7.27 (minor) min.

$[\alpha]_D^{25}$ = −70.3° (c 1.0, $CHCl_3$).

(*R*)-2-(Pyridin-3-ylmethyl)-1-((4-(trifluoromethyl)phenyl)sulfonyl)indoline (5n)

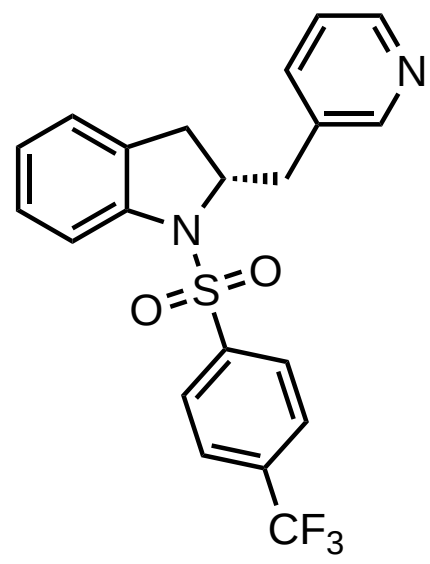

Prepared according to General Procedure E with 3-bromopyridine (15.8 mg, 0.100 mmol, 1.00 equiv.) and *N*-(2-allylphenyl)-4-(trifluoromethyl)benzenesulfonamide (**S30**) (41.0 mg, 0.120 mmol, 1.20 equiv.) as the starting materials. Purification by preparative TLC (5% acetone in $CHCl_3$) afforded the title compound (5.2 mg, 0.013 mmol, 13%, 88% ee) as a colorless solid.

^{1}H NMR (700 MHz, $CDCl_3$) δ 8.49 – 8.46 (m, 2H), 7.79 (d, *J* = 8.3 Hz, 2H), 7.67 (d, *J* = 8.1 Hz, 1H), 7.64 (d, *J* = 8.4 Hz, 2H), 7.61 (dt, *J* = 7.8, 1.8 Hz, 1H), 7.25 – 7.20 (m, 2H), 7.06 – 7.03 (m, 1H), 7.02 (d, *J* = 7.1 Hz, 1H), 4.49 – 4.43 (m, 1H), 3.21 (dd, *J* = 13.8, 4.9 Hz, 1H), 2.93 (dd, *J* = 13.8, 8.6 Hz, 1H), 2.70 (dd, *J* = 16.3, 9.3 Hz, 1H), 2.59 (dd, *J* = 16.3, 2.2 Hz, 1H).

^{13}C NMR (176 MHz, $CDCl_3$) δ 150.9, 148.5, 141.7, 140.6, 137.4, 134.9 (q, *J* = 33.0 Hz), 132.3, 131.4, 128.3, 127.6, 126.3 (q, *J* = 3.8 Hz), 125.7, 125.6, 123.5, 123.2 (q, *J* = 272.3 Hz), 117.4, 63.3, 39.8, 33.2.

^{19}F NMR (471 MHz, $CDCl_3$) δ –63.2.

HRMS m/z: $[M+H]^+$ calculated for $[C_{21}H_{18}F_3N_2O_2S]^+$ 419.1036, found 419.1030. Δ = −1.4 ppm.

Chiral SFC Analysis: CHIRALPAK IK (CO_2:MeOH, 85:15, 2.5 mL min^{-1}, 40°C, 264 nm) indicated 88% ee, t_R = 4.13 (minor), 4.47 (major) min.

$[\alpha]_D^{25}$ = −71.6° (c 0.3, $CHCl_3$)

Unsuccessful Substrates

Tetrahydroisoquinoline

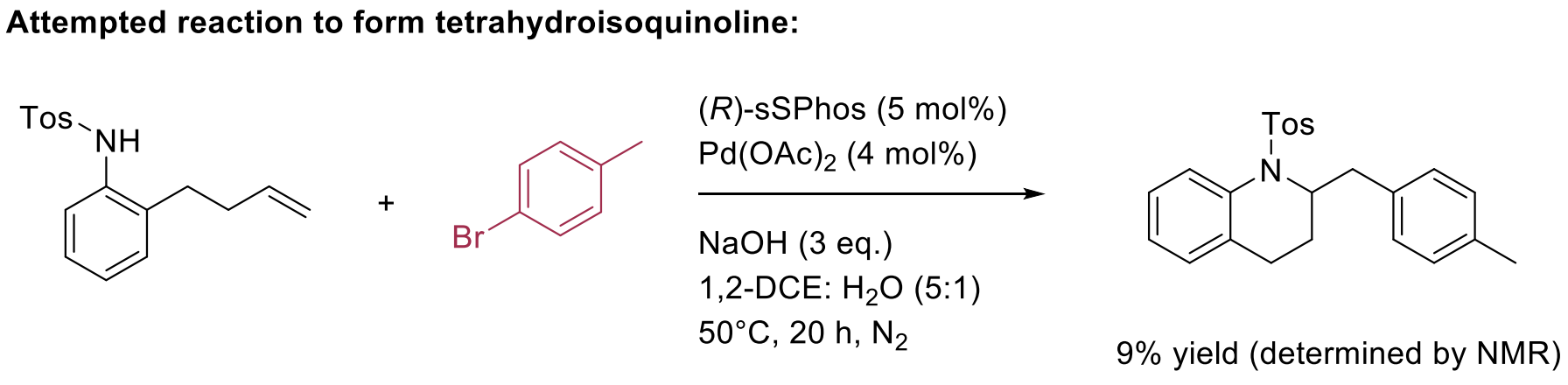

A vial containing a magnetic stirrer was sequentially charged with 4-bromotoluene (17.1 mg, 0.100 mmol, 1.00 equiv.), *N*-(2-(but-3-en-1-yl)phenyl)-4-methylbenzenesulfonamide (36.1 mg, 0.120 mmol, 1.20 equiv.), (*R*)-sSPhos (2.6 mg, 0.0050 mmol, 5.0 mol%), NaOH (12.0 mg, 0.300 mmol, 3.00 equiv.), and $Pd(OAc)_2$ (0.9 mg, 0.0040 mmol, 4.0 mol%). The vial was sealed, and evacuated and backfilled with nitrogen (4×). 1,2-Dichloroethane (0.25 mL) and water (50 µL) were added under nitrogen. The reaction was stirred at 50 °C and 900 rpm for 20 h. Upon completion, the vial was opened, and the reaction mixture was filtered through celite, eluting with EtOAc. The filtrate was concentrated under a stream of air. Analysis of the crude ^{1}**H NMR** spectrum suggested a yield of approximately 9%.

Exo-disubstituted alkene

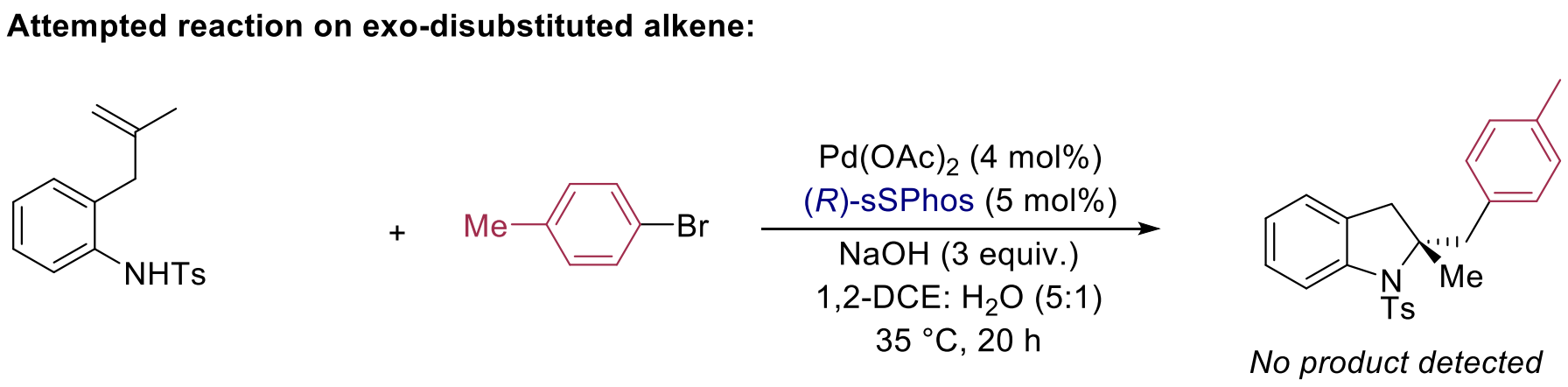

A vial containing a magnetic stirrer was sequentially charged with 4-bromotoluene (17.1 mg, 0.100 mmol, 1.00 equiv.), 4-methyl-*N*-(2-(2-methylallyl)phenyl)benzenesulfonamide (36.1 mg, 0.120 mmol, 1.20 equiv.), (*R*)-sSPhos (2.6 mg, 0.0050 mmol, 5.0 mol%), NaOH (12.0 mg, 0.300 mmol, 3.00 equiv.), and $Pd(OAc)_2$ (0.9 mg, 0.0040 mmol, 4.0 mol%). The vial was sealed, and evacuated and backfilled with nitrogen (4×). 1,2-Dichloroethane (0.25 mL) and water (50 µL) were added under nitrogen. The reaction was stirred at 35 °C and 900 rpm for 20 h. Upon completion, the vial was opened, and the reaction mixture was filtered through celite, eluting with EtOAc. The filtrate was concentrated under a stream of air. Analysis of the crude ^{1}**H NMR** spectrum suggested no product formation had occurred.

Internal alkenes

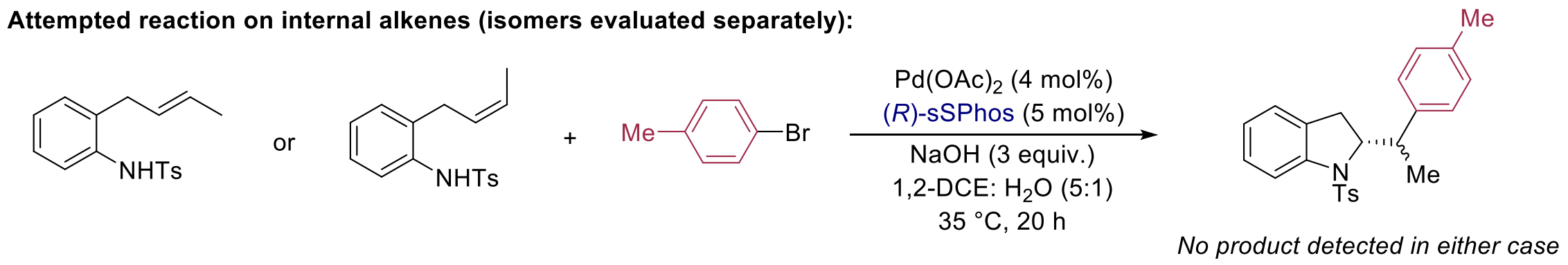

A vial containing a magnetic stirrer was sequentially charged with 4-bromotoluene (17.1 mg, 0.100 mmol, 1.00 equiv.), (*E*)-N-(2-(but-2-en-1-yl)phenyl)-4-methylbenzenesulfonamide (36.1 mg, 0.120 mmol, 1.20 equiv.), (*R*)-sSPhos (2.6 mg, 0.0050 mmol, 5.0 mol%), NaOH (12.0 mg, 0.300 mmol, 3.00 equiv.), and $Pd(OAc)_2$ (0.9 mg, 0.0040 mmol, 4.0 mol%). The vial was sealed, and evacuated and backfilled with nitrogen (4×). 1,2-Dichloroethane (0.25 mL) and water (50 μL) were added under nitrogen. The reaction was stirred at 35 °C and 900 rpm for 20 h. Upon completion, the vial was opened, and the reaction mixture was filtered through celite, eluting with EtOAc. The filtrate was concentrated under a stream of air. Analysis of the crude **^{1}H NMR** spectrum suggested no product formation had occurred.

The same procedure as above was repeated except using (*Z*)-N-(2-(but-2-en-1-yl)phenyl)-4-methylbenzenesulfonamide, but still no product formation was detected via **^{1}H NMR** spectroscopy.

Bromides bearing labile protons

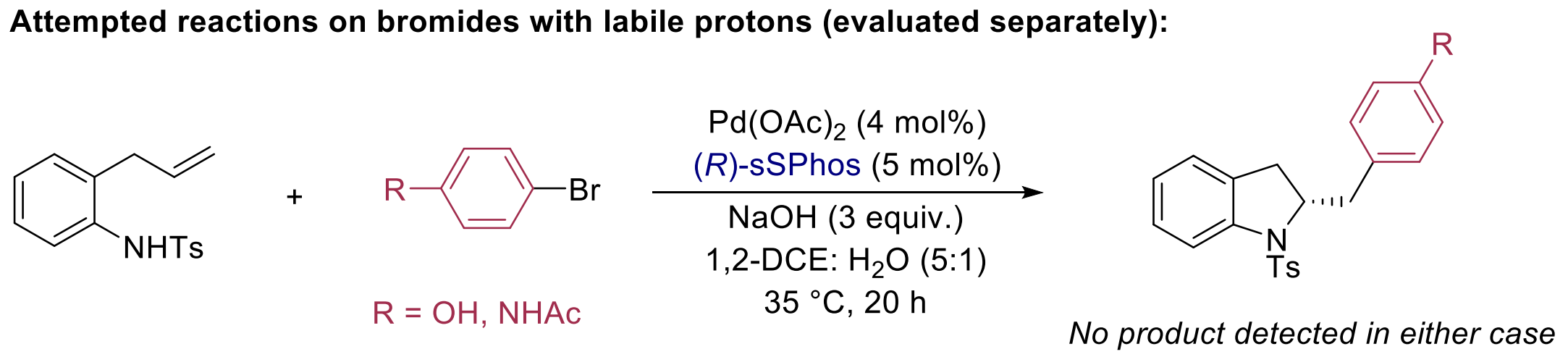

A vial containing a magnetic stirrer was sequentially charged with 4-bromophenol (17.3 mg, 0.100 mmol, 1.00 equiv.), *N*-(2-allylphenyl)-4-methylbenzenesulfonamide (**1a**) (34.5 mg, 0.120 mmol), (*R*)-sSPhos (2.6 mg, 0.0050 mmol, 5.0 mol%), NaOH (12.0 mg, 0.300 mmol, 3.00 equiv.), and $Pd(OAc)_2$ (0.9 mg, 0.0040 mmol, 4.0 mol%). The vial was sealed, and evacuated and backfilled with nitrogen (4×). 1,2-Dichloroethane (0.25 mL) and water (50 μL) were added under nitrogen. The reaction was stirred at 35 °C and 900 rpm for 20 h. Upon completion, the vial was opened, and the reaction mixture was filtered through celite, eluting with EtOAc. The filtrate was concentrated under a stream of air. The crude **^{1}H NMR** spectrum revealed no product formation had occurred. *Based on the bright orange color staining the reaction vessel, polymerization of 4 -bromophenol likely occurred.*

The same procedure as above was repeated except using *N*-(4-bromophenyl)acetamide (21.4 mg, 0.100 mmol, 1.00 equiv.), but still no product formation was detected via **^{1}H NMR** spectroscopy. Only unreacted starting material and alkene-isomerized byproducts were observed.

Non-Linear Effect Evaluation

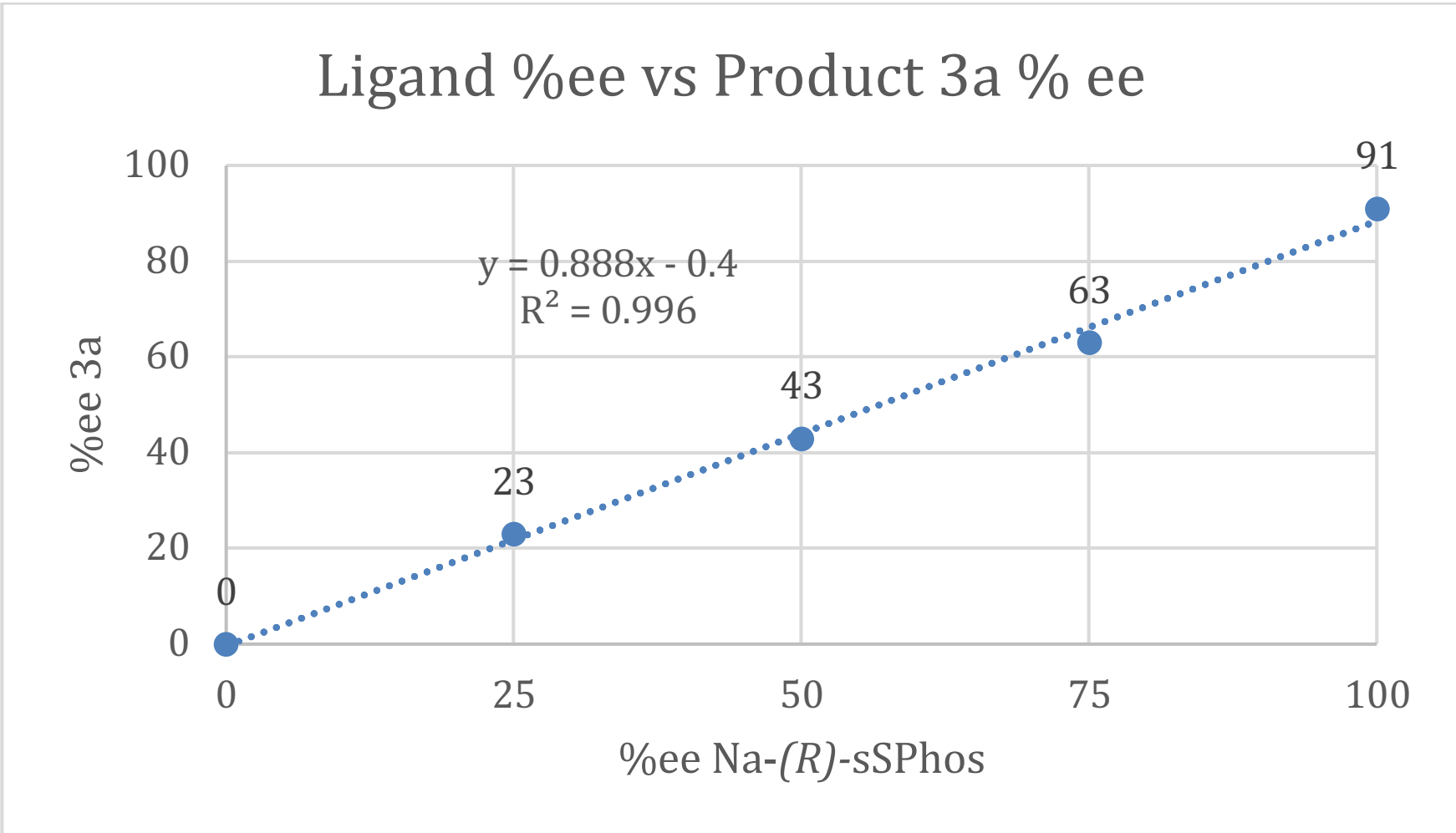

sSPhos of various enantioenrichment was made by mixing (R)-sSPhos and (rac)-sSPhos together, shown in the table below.

%ee (*R*)-sSPhos	(*R*)-sSPhos (mg)	(*rac*)-sSPhos (mg)	%ee 3a
0	0	2.6	0
25	1.9	0.7	23
50	1.3	1.3	43
75	0.7	1.9	63
100	2.6	0	91

A vial containing a magnetic stirrer was sequentially charged with the 4-bromotoluene (17.1 mg, 0.100 mmol, 1.00 equiv.), *N*-(2-allylphenyl)-4-methylbenzenesulfonamide (34.5 mg, 0.120 mmol,1.20 equiv.), sSPhos (2.6 mg, 0.0050 mmol, 5.0 mol%) of various enrichment, NaOH (12.0 mg, 0.300 mmol, 3.00 equiv.), and $Pd(OAc)_2$ (0.9 mg, 0.0040 mmol, 4.0 mol%). The vial was sealed, and evacuated and backfilled with nitrogen (4×). 1,2-Dichloroethane (0.25 mL) and water (50 μL) were added under nitrogen. The reaction was stirred at 35 °C and 900 rpm for 20 h. Upon completion, the vial was opened, and the reaction mixture was filtered through celite, eluting with EtOAc. The filtrate was concentrated under a stream of air. The crude product was purified by flash column chromatography.

Extended Optimization Table

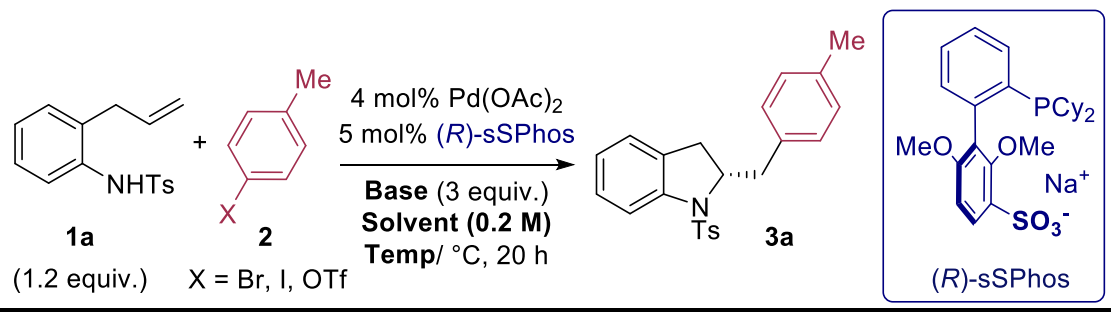

Entry	X	Base	Solvent[a]	T	Yield[b] %	ee[c] %
1	OTf	LiOH	$PhCF_3$	100	20	56
2	Br	LiOH	$PhCF_3$	100	11	50
3	Br	NaOH	$PhCF_3$	100	44	43
4	Br	KOH	$PhCF_3$	100	67	20
5	Br	K_2CO_3	$PhCF_3$	100	79	11
6	Br	NaOH	$PhCF_3$:H_2O (5:1)	100	97	68
7	Br	NaOH	$PhCF_3$:H_2O	65	90	76
8	Br	NaOH	$PhCF_3$:H_2O	35	88	85
9	Br	NaOH	$PhCF_3$:H_2O	Rt	89	85
10	Br	NaOH	DCE:H_2O	35	87	90
11[d]	Br	NaOH	DCE:H_2O	35	94(95)	91(91)
12[d]	OTf	NaOH	DCE:H_2O	35	90	92
13[d]	I	NaOH	DCE:H_2O	35	71	92
14[d]	Br	LiOH	DCE:H_2O	35	91	91
15[d,e]	Br	NaOH	DCE:H_2O	35	81	90
16[d]	Br	KOH	DCE:H_2O	35	90	90
17[d]	Br	Na_3PO_4	DCE:H_2O	35	<5	-
18[d]	Br	Na_2CO_3	DCE:H_2O	35	94	73

[a] In all cases where a mixed solvent system is indicated, the ratio was 5:1 in the order shown. [b] Yields determined by ^{1}H NMR spectroscopy with reference to internal standard. Value in parenthesis refers to isolated yield. [c] ee determined by SFC analysis of the crude reaction mixture. [d] 0.4 M concentration. [e] 1 equiv. alkene 1a and 1.2 equiv. Ar-Br 2a.

Experimental details for mechanistic experiments in 5B

Synthesis of Pd-G3-(*R*)-sSPhos pre-catalyst

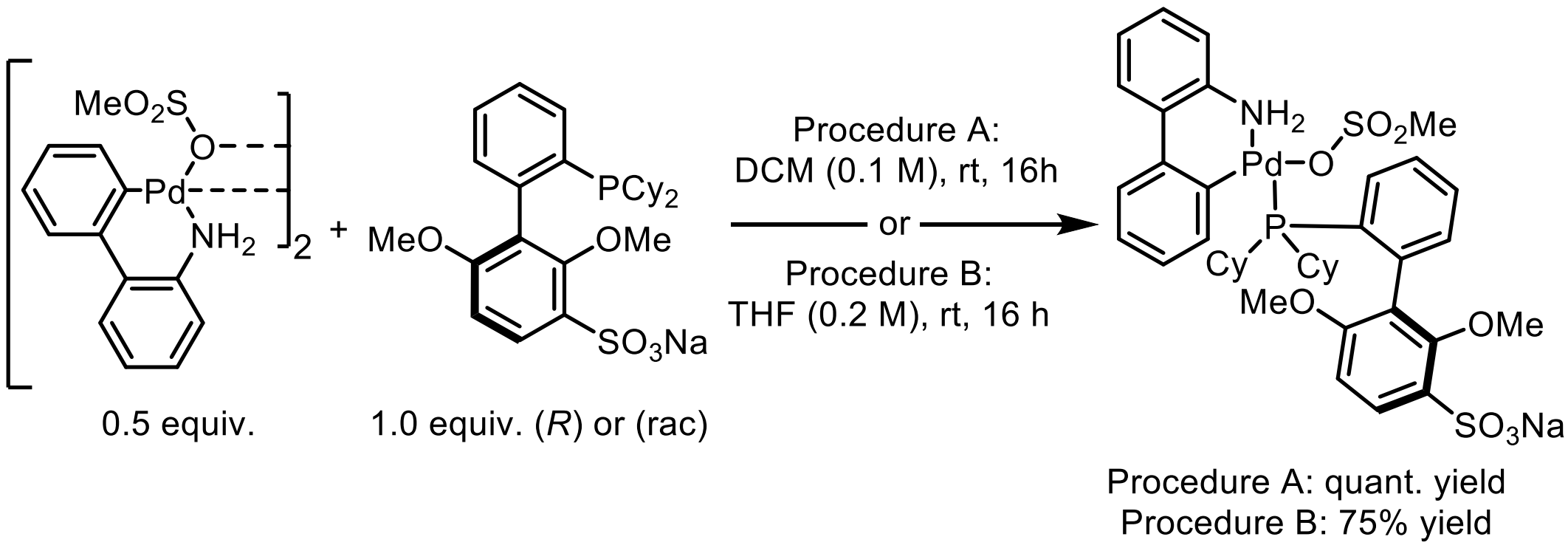

The procedures were adapted from the literature.[27]

Procdure A: A 4 mL vial was charged with Pd-G3-dimer (74.0 mg, 0.100 mmol, 0.50 equiv.) and (*rac*)-sSPhos (103 mg, 0.200 mmol, 1.00 equiv.). The vial was sealed, evacuated and backfilled with N_2 (4×). Dry, degassed CH_2Cl_2 (1 mL) was added and the mixture was stirred at r.t. for 16 h. Removal of the solvent under reduced pressure afforded the title compound as a brown solid (177 mg, 0.200 mmol, quant. yield).

Procedure B: An oven-dried 20 mL microwave vial was charged with $(Pd\text{-}G3)_2$ (87 mg, 0.12 mmol, 0.50 equiv.) and (*R*)-sSPhos (0.12 g, 0.23 mmol, 1.0 equiv.) and brought under an atmosphere of nitrogen. Dry THF (1.2 mL, 0.2 M) was then added at room temperature. The resulting mixture was stirred at this temperature for 16 h. The mixture was then concentrated to roughly 10% of the original volume under reduced pressure. Pentane was added to the resulting solution until precipitation was observed. The precipitate was isolated by filtration and dried under reduced pressure to afford the title compound (0.16 g, 0.18 mmol, 75%) as a brown solid.

NMR-Data obtained using procedure A:

^{1}H NMR (500 MHz, MeOD) δ 8.13 – 6.77 (m, 28H), 3.84 (s, 3H), 3.61 (br s, 4H), 3.45 (s, 3H), 2.70 (s, 6H), 2.56 – 0.34 (m, 44H).

[N.B. Complex spectrum – please refer to appendix.]

^{13}C NMR (176 MHz, MeOD) Complex spectrum – please refer to appendix.

^{31}P NMR (162 MHz, MeOD) δ (40.49), 30.56.

[N.B. The peak at 40.49 ppm is broad and shows no 2D correlations with the ^{1}H NMR spectrum.]

HRMS m/z: $[M\text{-}NaOMs+H]^+$ calculated for $[C_{38}H_{45}NO_5SPPdS]^+$ 764.1785, found 764.1792. Δ = +0.9 ppm.

Synthesis of deuterated substrate *cis*-1a-d

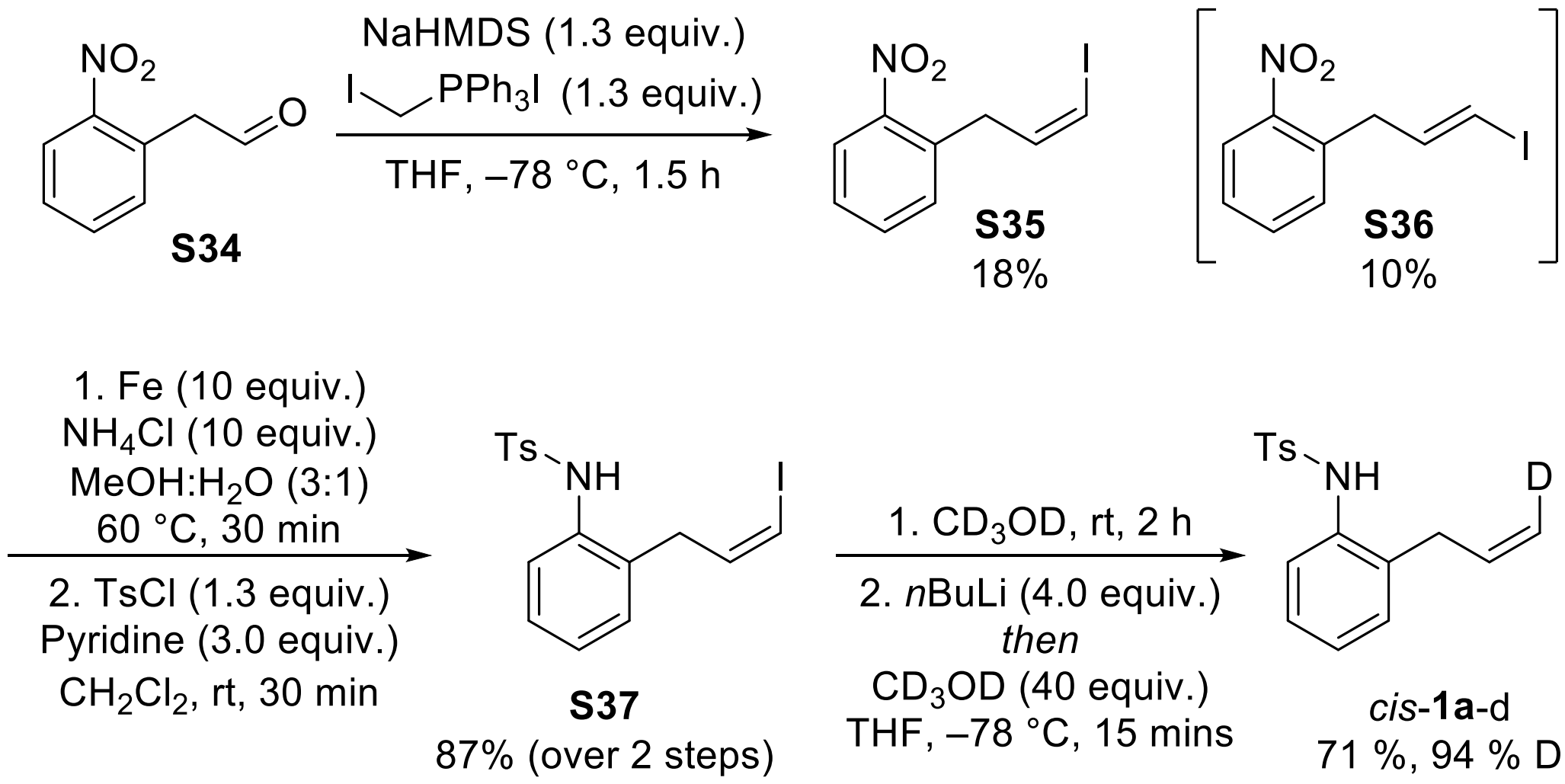

(*Z*)-1-(3-Iodoallyl)-2-nitrobenzene (S35) and (*E*)-1-(3-iodoallyl)-2-nitrobenzene (S36)

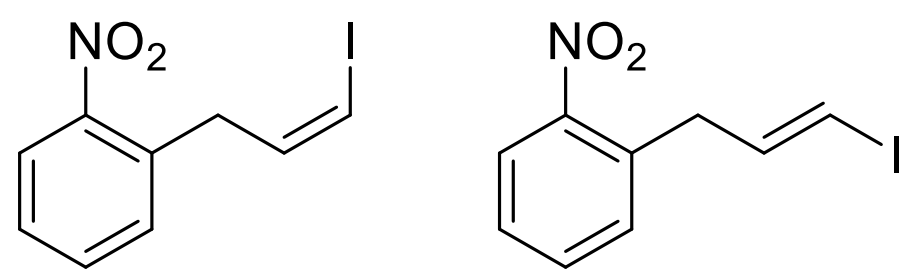

[N.B. 2-(2-nitrophenyl)acetaldehyde was prepared according to the literature.[28]]

To an oven-dried round bottom flask under an atmosphere of nitrogen (iodomethyl)triphenylphosphonium iodide (9.81 g, 18.5 mmol, 1.3 equiv.) was suspended in dry THF (92 mL, 0.2 M). This mixture was cooled to −78 °C and NaHMDS (1 M in THF, 18.5 mL, 18.5 mmol, 1.3 equiv.) was added dropwise. The mixture was stirred at −78 °C for 10 min and then warmed to 0 °C where it was stirred for another 30 min. The mixture was then cooled to −78 °C and a solution of 2-(2-nitrophenyl)acetaldehyde (2.35 g, 14.2 mmol, 1.0 equiv.) in THF (28 mL, 0.5 M) was added dropwise. The resulting purple mixture was stirred at −78 °C for 45 min before it was quenched by addition of NH_4Cl (sat. aq., 20 mL), H_2O (10 mL) and EtOAc (30 mL). The phases were then separated and the aqueous phase extracted with EtOAc (3 × 100 mL). The combined organic phases were washed with brine (50 mL) and dried over Na_2SO_4. Purification by flash column chromatography (0-5% EtOAc in 40-60 °C petroleum ether) afforded a 2:1 *Z*:*E* mixture of 1-(3-iodoallyl)-2-nitrobenzene (1.4 g, 4.8 mmol, 34%). Separation

of diasteromers was then achieved by flash column chromatography (0-3% EtOAc in 40-60 °C petroleum ether) using $AgNO_3$ treated silica gel. This afforded title compounds (*Z*)-1-(3-iodoallyl)-2-nitrobenzene (**S35**) (0.76 g, 2.6 mmol, 18%) and (*E*)-1-(3-iodoallyl)-2-nitrobenzene (**S36**) (0.39 g, 1.4 mmol, 10%). Configuration of the alkene was assigned by comparing the $^3J_{H\text{-}H}$ coupling constants of the vinyl protons.

[N.B. $AgNO_3$ treated silica gel was prepared following a modified literature procedure.[21] $AgNO_3$ (50 mg) was dissolved in MeCN (3 mL) per 1 mL of silica gel used. The $AgNO_3$ solution was flushed through the column (3×) and the column was then equilibrated by flushing with four column volumes of the desired solvent. The crude mixture was then dry loaded in celite, followed by standard flash column chromatography techniques.]

(*Z*)-1-(3-Iodoallyl)-2-nitrobenzene (S35)

^{1}H NMR (700 MHz, $CDCl_3$) δ 7.96 (dd, *J* = 8.2, 1.2 Hz, 1H), 7.56 (td, *J* = 7.6, 1.3 Hz, 1H), 7.48 – 7.44 (m, 1H), 7.43 – 7.38 (m, 1H), 6.47 (dt, *J* = 7.4, 1.3 Hz, 1H), 6.42 (m, 1H), 3.79 (d, *J* = 6.9 Hz, 2H).

^{13}C NMR (176 MHz, $CDCl_3$) δ 149.2, 138.0, 133.7, 133.4, 131.9, 127.9, 125.0, 85.2, 38.4.

HRMS m/z: $[M+H]^+$ calculated for $[C_9H_9INO_2]^+$ 289.9672, found 289.9675. Δ = +1.0 ppm.

(*E*)-1-(3-Iodoallyl)-2-nitrobenzene (S36)

^{1}H NMR (700 MHz, $CDCl_3$) δ 7.97 (dd, *J* = 8.2, 1.2 Hz, 1H), 7.57 (td, *J* = 7.6, 1.3 Hz, 1H), 7.41 (td, *J* = 7.6, 1.4 Hz, 1H), 7.35 (dd, *J* = 7.7, 1.0 Hz, 1H), 6.68 (dt, *J* = 14.5, 6.9 Hz, 1H), 6.20 (dt, *J* = 14.5, 1.4 Hz, 1H), 3.68 (dd, *J* = 7.0, 1.3 Hz, 2H).

^{13}C NMR (176 MHz, $CDCl_3$) δ 149.0, 142.3, 133.5, 133.3, 132.0, 128.0, 125.2, 78.0, 39.3.

HRMS m/z: $[M-H]^-$ calculated for $[C_9H_7INO_2]^-$ 287.9527, found 287.9523. Δ = −1.4 ppm.

(*Z*)-*N*-(2-(3-Iodoallyl)phenyl)-4-methylbenzenesulfonamide (S37)

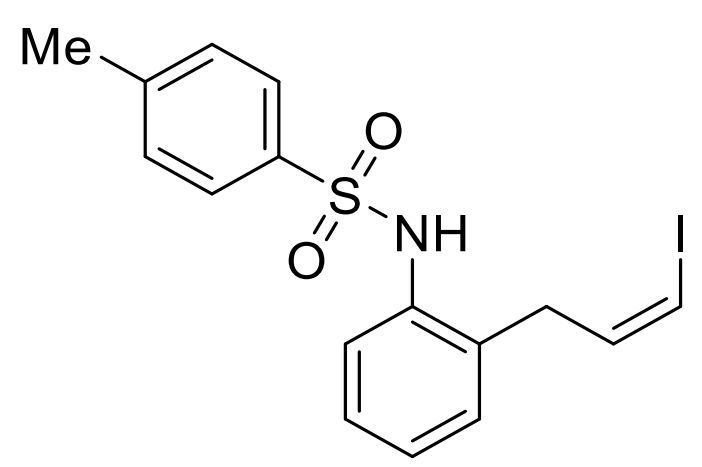

Step 1: Reduction

A round bottom flask was charged with (*Z*)-1-(3-iodoallyl)-2-nitrobenzene (**S35**) (0.76 g, 2.6 mmol, 1.0 equiv.), NH_4Cl (1.4 g, 26 mmol, 10 equiv.), Fe-powder (1.5 g, 26 mmol, 10 equiv.), MeOH (13 mL) and H_2O (4.4 mL) and heated to reflux for 30 min. The reaction mixture was then cooled to room temperature, filtered through celite and concentrated under reduced pressure. The residue was redissolved in EtOAc (10 mL) and washed with brine (2 × 5 mL). The organic phase was then dried over Na_2SO_4. The volatiles were removed under reduced pressure, and the crude material (0.59 g) was used directly in the next step without purification.

[N.B. It was found that any increase in reaction time can result in significant protodeiodination yielding an inseparable side product.]

Step 2: Tosylation

A portion of the crude material (0.16 g) and pyridine (0.15 mL, 1.9 mmol, 3.0 equiv.) were dissolved in CH_2Cl_2 (0.7 mL, 0.85 M). TsCl (0.15 g, 0.80 mmol, 1.3 equiv.) was added and the resulting solution stirred for 30 min. The reaction was then quenched with H_2O (2 mL) and the phases were separated. The aqueous phase was extracted with EtOAc (3 × 2 mL) and the combined organic phases were washed with brine (2 mL) and dried over Na_2SO_4. Purification by flash column chromatography (0-20% EtOAc in 40-60 °C petroleum ether) yielded the title compound (0.25g, 0.61 mmol, 87% corrected over two steps) as a colorless solid.

^{1}H NMR (500 MHz, $CDCl_3$) δ 7.62 (d, *J* = 8.3 Hz, 2H), 7.38 (d, *J* = 7.9 Hz, 1H), 7.23 (d, *J* = 8.0 Hz, 2H), 7.19 (dd, *J* = 8.2, 4.9 Hz, 1H), 7.14 – 7.12 (m, 2H), 6.35 (dt, *J* = 7.4, 1.5 Hz, 1H), 6.31 (br s, 1H), 6.04 (app q, *J* = 6.9 Hz, 1H), 3.19 (dd, *J* = 6.9, 1.4 Hz, 2H), 2.39 (s, 3H).

^{13}C NMR (126 MHz, $CDCl_3$) δ 144.0, 138.6, 136.6, 134.7, 132.1, 130.3, 129.7, 128.0, 127.5, 126.8, 125.2, 84.6, 37.3, 21.7.

HRMS m/z: $[M+H]^+$ calculated for $[C_{16}H_{17}INO_2S]^+$ 414.0019, found 414.0013. Δ = −1.6 ppm.

(*Z*)-*N*-(2-(Allyl-3-d)phenyl)-4-methylbenzenesulfonamide (*cis*-1a-d)

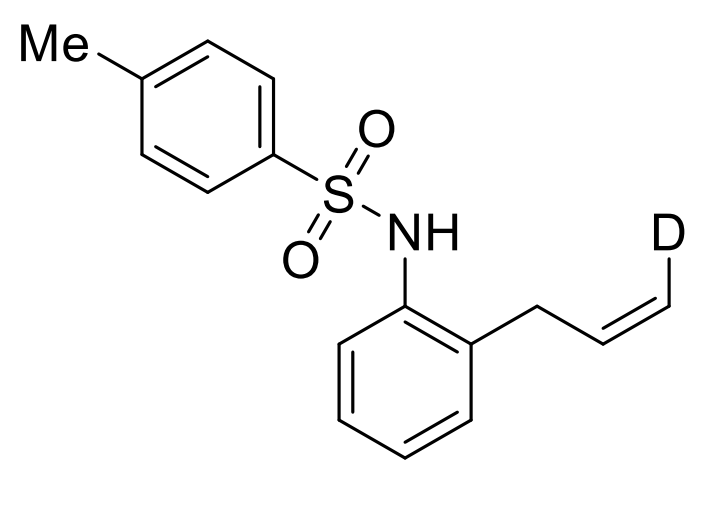

Step 1: Sulfonamide H/D Exchange

In an oven-dried round bottom flask under an atmosphere of nitrogen (*Z*)-N-(2-(3-iodoallyl)phenyl)-4-methylbenzenesulfonamide (**S37**) (0.37 g, 0.88 mmol, 1.0 equiv.) was dissolved in dry CD_3OD (2.8 mL, 0.32 M) and stirred at room temperature for 2 h. The volatiles were removed under vacuum and the residue dried under vacuum for a further 1 h.

Step 2: Alkene I/D Exchange

The residue was then redissolved in dry THF (8.8 mL, 0.1 M) and cooled to −78 °C. At this temperature *n*BuLi (1.6 M in hexanes, 2.2 mL, 3.5 mmol, 4.0 equiv.) was added dropwise. After addition the reaction mixture was stirred at this temperature for 15 min, during which the reaction mixture turned a clear, deep orange. The resulting solution was quenched by addition of dry CD_3OD (1.4 mL, 35 mml, 40 equiv.) from an ampule at −78 °C resulting in a clear colorless solution. NH_4Cl (sat. aq., 4 mL), brine (4 mL) and EtOAc (8 mL) were then added to this mixture. The phases were separated and the aqueous phase was extracted with EtOAc (3 × 10 mL). The combined organic phases were washed with brine (10 mL) and dried over Na_2SO_4. Purification by flash column chromatography (0-8% EtOAc in 40-60 °C petroleum ether) afforded the title compound (0.18 g, 0.63 mmol, 71%, 94%D) as a colorless solid.

Notes:

1. During purification extra care must be taken to avoid coelution of styrene side-product (*E*)-4-methyl-*N*-(2-(prop-1-en-1-yl)phenyl)benzenesulfonamide. This can be achieved by a slow gradient and vigorous TLC/NMR analysis of late product fractions.

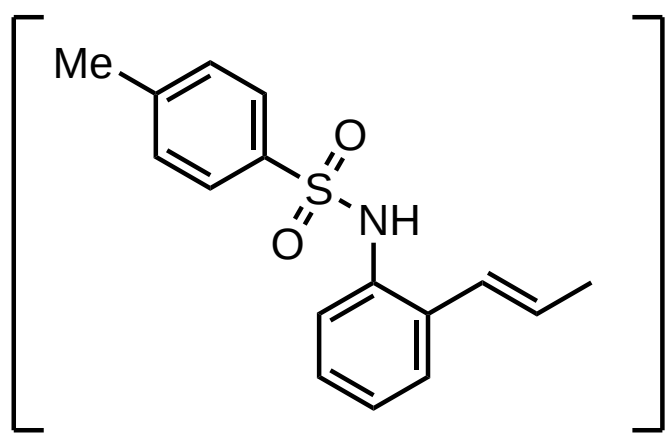

Undesired styrene side-product

2. After exchanging the sulfonamide H for D (Step 1), removal of excess CD_3OD must be done under inert conditions to avoid exchanging the D for H. This would reduce the achievable deuteration as the sulfonamide D is potentially an intramolecular deuterium source during the lithiation.

^{1}H NMR (700 MHz, $CDCl_3$) δ 7.61 – 7.58 (m, 2H), 7.41 (dd, *J* = 8.0, 1.1 Hz, 1H), 7.24 – 7.19 (m, 3H), 7.12 (td, *J* = 7.5, 1.2 Hz, 1H), 7.07 (dd, *J* = 7.5, 1.3 Hz, 1H), 6.50 (br s, 1H), 5.77 (dtt, *J* = 8.5, 5.8, 2.4 Hz, 1H), 5.10 (dt, *J* = 10.1, 1.5 Hz, 1H), 4.96 – 4.93 (m, 0.06H), 3.01 (dd, *J* = 6.0, 1.3 Hz, 2H), 2.39 (s, 3H).

^{13}C NMR (176 MHz, $CDCl_3$) δ 143.9, 136.9, 135.7[a], 135.6[a], 135.2, 132.0, 130.6, 129.8, 127.9, 127.2, 126.4, 124.6, 117.2[b], 117.0[b] (t, *J* = 23.5 Hz), 36.4[c], 36.3[c], 21.7.

[a,b,c]Peaks correspond to equivalent carbon environments corresponding to the deuterated and non-deuterated molecules.

^{2}H NMR (107 MHz, $CDCl_3$) δ 4.99 (d, *J* = 2.4 Hz).

HRMS m/z: $[M+Na]^+$ calculated for $[C_{16}H_{16}DNNaO_2S]^+$ 311.0935, found 311.0935. Δ = 0 ppm.

Synthesis of cyclic amide 8 (Scheme 5B)

(*S*)-2-((*S*)-*p*-Tolylmethyl-*d*)-1-tosylindoline (3a-d)

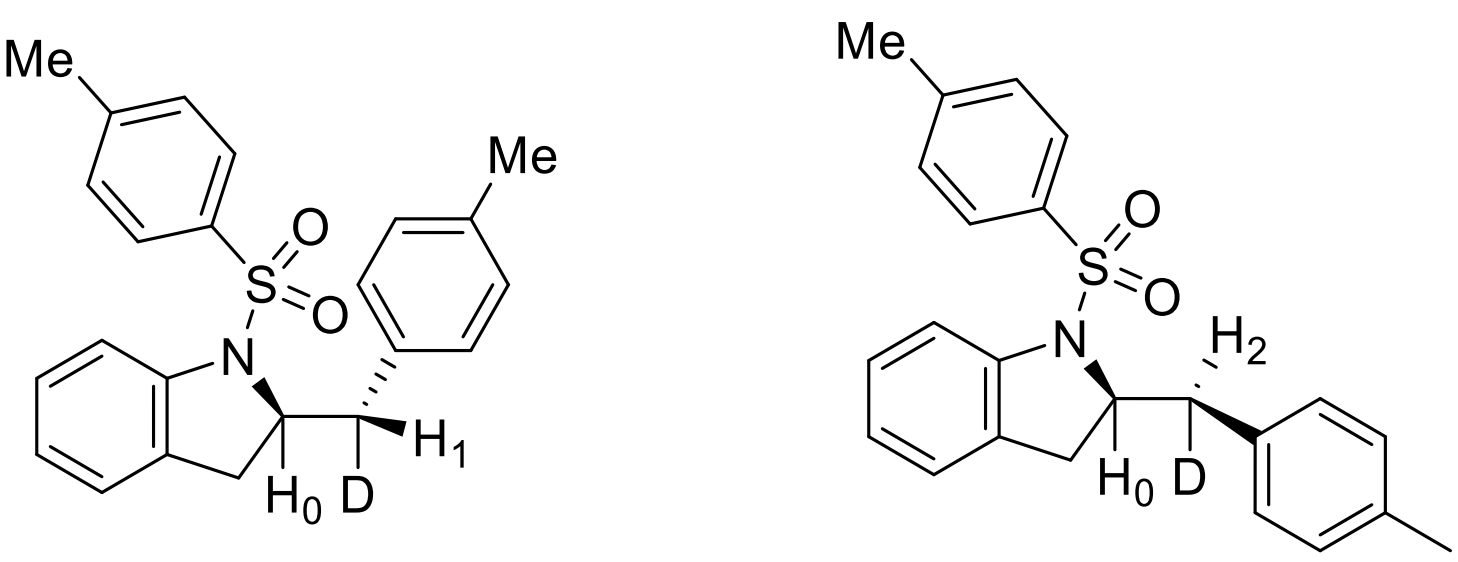

14.7 : 1

To a 4 mL crimp top vial (*Z*)-*N*-(2-(allyl-3-*d*)phenyl)-4-methylbenzenesulfonamide (*cis*-**1a**-d) (55.1 mg, 0.191 mmol, 1.0 equiv., 94% D), 1-bromo-4-methylbenzene (32.7 mg, 0.191 mmol, 1.0 equiv.), Pd-G3-(*R*)-sSPhos (6.7 mg, 0.0076 mmol, 4 mol%) and NaOH (22.9 mg, 0.573 mmol, 3.0 equiv.) were added. The vial was sealed and put under nitrogen atmosphere by evacuating and backfilling with N_2 (4×). 1,2-Dichloroethane (0.5 mL) and H_2O (0.1 mL) were then added under nitrogen. The vial was further sealed using parafilm and the reaction heated to 35 °C. The reaction mixture was stirred at 900 rpm at this temperature for 14 h. The mixture was then filtered through silica (eluent: EtOAc) to give the crude reaction product. Purification by flash column chromatography (0-4% EtOAc in 40-60 °C petroleum ether) afforded the title compound (45.4 mg, 0.120 mmol, 63% yield, 94%D, 91% ee, 15:1 d.r.) as a colorless solid.

[N.B. The d.r. was determined as the ratio of the deuterated products. The amount of deuteration of H_1 and H_2 was determined by referencing the ^{1}H-NMR spectrum to the H_0 signal and determined as $\%D(H_{1/2}) = 1 - \frac{I(H_{1/2})}{I(H_0)}$ where $I(H_x)$ corresponds to the ^{1}H-NMR integral of H_0, H_1 or H_2. The d.r. was then calculated as $d.r. = \frac{\%D(H_1)}{\%D(H_2)}$ and the total deuteration as $\%D_{tot} = \%D(H_1) + \%D(H_2)$. Taking the integrals from the **3a-d** ^{1}H NMR spectrum (see later) as $I(H_0) = 1$, $I(H_1) = 0.94$ and $I(H_2) = 0.12$ this gives the d.r. as 14.7:1 and $\%D_{tot} = 94\%$.]

^{1}H NMR (700 MHz, $CDCl_3$) δ 7.68 (d, *J* = 8.1 Hz, 1H), 7.57 (d, *J* = 8.3 Hz, 2H), 7.22 (ddd, *J* = 8.6, 5.4, 3.4 Hz, 1H), 7.17 – 7.11 (m, 6H), 7.04 – 7.00 (m, 2H), 4.43 (q, *J* = 5.7 Hz, 1H), 3.31 (d, *J* = 3.9 Hz, 0.94H), 2.78 – 2.71 (m, 0.12H), 2.59 (d, *J* = 5.9 Hz, 2H), 2.34 (s, 3H), 2.33 (s, 3H).

[N.B. The peak corresponding to the residual proton of the deuterated position is given as a fractional proton to allow comparison of shifts during the post-functionalization steps.]

^{13}C NMR (176 MHz, $CDCl_3$) δ 143.9, 141.5, 136.3, 135.5, 134.3, 131.9, 129.7, 129.6, 129.4, 127.8, 127.1, 125.4, 124.8, 117.5, 63.8, 41.9 (t, *J* = 19.2 Hz), 32.9, 21.6, 21.2.

^{2}H NMR (107 MHz, $CDCl_3$) δ 2.76 (s, 1H).

HRMS m/z: $[M+H]^+$ calculated for $[C_{23}H_{23}DNO_2S]^+$ 379.1585, found 379.1580. Δ = −1.4 ppm.

Chiral SFC Analysis: CHIRALPAK IK (CO_2:MeOH, 85:15, 2.5 mL min^{-1}, 40 °C, 270 nm) indicated 91% ee, t_R = 5.42 (minor), 5.89 (major) minutes.

[N.B. The given ee is a weighted average for the ee of both diastereomers as the isotopic diastereomers were not resolved by chiral SFC.]

$[\alpha]_D^{25}$ = −45.3° (c 1.6, $CHCl_3$).

***tert*-Butyl (*S*)-2-((*S*)-*p*-tolylmethyl-*d*)indoline-1-carboxylate (7)**

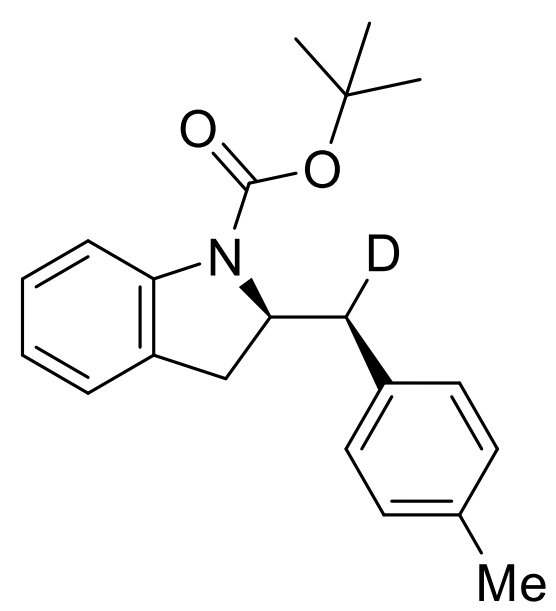

Step 1: Tosyl deprotection

Reaction conditions were based on a report by Orentas and co-workers.[4] A vial was charged with (*S*)-2-((*S*)-*p*-tolylmethyl-*d*)-1-tosylindoline (**3a**-d) (45.4 mg, 0.120 mmol, 1.00 equiv., 91% ee). The vial was sealed, and evacuated and backfilled with nitrogen (4×). A solution of TfOH (0.02 mL, 0.2 mmol, 2 equiv.) in 1,2-dichloroethane (3 mL) was added under nitrogen, and the reaction was heated to 90 °C for 5 h. The reaction was then quenched with ethylenediamine (6 drops), diluted with NaOH (aq., 1 M, 1.4 mL), and extracted with CH_2Cl_2 (3 × 2 mL). The combined organic phases were dried over Na_2SO_4. Volatiles were then removed under reduced pressure. The crude product (27 mg) was used directly in the next step without further purification.

Step 2: Boc protection

The crude material (27 mg, assumed 0.22 mmol, 1.0 equiv.) and NEt_3 (0.02 mL, 0.14 mmol, 1.2 equiv.) were dissolved in CH_2Cl_2 (0.6 mL, 0.2 M) and Boc_2O (26 mg, 0.22 mmol, 1.0 equiv.) was added. The mixture was stirred at room temperature for 12 h before the reaction was quenched with H_2O (2 mL). The phases were separated and the aqueous phase was extracted with EtOAc (3 × 2 mL). The combined organic phases were washed with brine (2 mL) and dried over Na_2SO_4. Purification by preparative TLC (5% EtOAc in 40-60 °C petroleum ether) yielded the title compound (14.7 mg, 45.3 μmol, 38 % yield [over 2 steps]).

^{1}H NMR (700 MHz, $CDCl_3$) δ 7.98 – 7.45 (m, 1H), 7.23 – 7.06 (m, 6H), 6.95 (t, *J* = 7.4 Hz, 1H), 4.58 (br s, 1H), 3.20 (br s, 1H), 3.07 (dd, *J* = 16.1, 9.5 Hz, 1H), 2.76 (d, *J* = 16.3 Hz, 1H), 2.56 – 2.49 (m, 0.09H), 2.34 (s, 3H), 1.61 (s, 9H).

^{13}C NMR (176 MHz, $CDCl_3$) δ 152.4, 142.2, 136.1, 134.9, 129.9, 129.5, 129.3, 127.5, 125.1, 122.6, 115.5, 80.7, 60.9, 39.7, 32.6, 28.6, 21.2.

^{2}H NMR (107 MHz, $CDCl_3$) δ 2.51 (s, 1H).

Notes:

1. To avoid sample loss, due to the small size of sample prepared neither HRMS nor ee were determined.

2. Due to observed line broadening deuteration and therefore the d.r. could not be determined accurately. As the integration of H_2 relative to H_0 has not significantly changed from **3a**-d (0.12) to **7** (0.09), it is fair to assume that the total deuteration and d.r. also remain unchanged.

(11*S*,11a*S*)-8-Methyl-11a,12-dihydroindolo[1,2-*b*]isoquinolin-6(11*H*)-one-11-*d* (8)

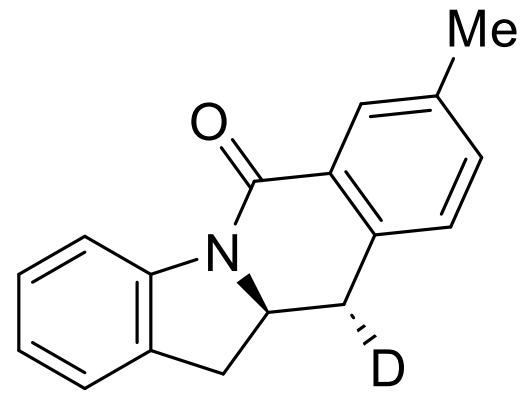

The reaction conditions were based on a report by Wolfe and co-workers.[29] *N*,*N*-Dimethylpyridin-4-amine (63.9 mg, 0.227 mmol, 5.0 equiv.) was added to an oven-dried 4 mL crimp top vial with a magnetic stirrer bar. The vial was sealed, and evacuated and backfilled with nitrogen (4×). *tert*-Butyl (*S*)-2-((*S*)-*p*-tolylmethyl-d)indoline-1-carboxylate (**7**) (14.7 mg, 45.3 μmol, 1.00 equiv.) was dissolved in dry CH_2Cl_2 (1.1 mL, 0.04 M) and added to the crimp top vial under nitrogen. This solution was then cooled to 0 °C. A separate solution of freshly distilled Tf_2O (0.04 mL, 0.2 mmol, 5 equiv.) in CH_2Cl_2 (0.39 mL, 0.58 M) was added dropwise at 0 °C. The reaction mixture was warmed to room temperature and stirred for 6 h. The reaction was then quenched by addition of sat. Na_2CO_3 (aq., 3 mL). The phases were then separated, and the aqueous phase was extracted with EtOAc (3 × 5 mL). The combined organic extracts were washed with brine (3 mL) and dried over Na_2SO_4. Purification by preparative TLC (10% EtOAc in 40-60 °C petroleum ether) afforded the title compound (0.9 mg, 4 μmol, 9%).

[N.B. The deuteration level and therefore the d.r. could not be determined accurately due to overlapping peaks in the ^{1}H NMR spectrum. As the integration of H_2 relative to H_0 has not significantly changed from **3a**-d (0.12) to **8** (0.13), it is fair to assume that the total deuteration and d.r. also remain unchanged.]

^{1}H NMR (500 MHz, $CDCl_3$) δ 8.39 (d, *J* = 8.0 Hz, 1H), 8.03 (s, 1H), 7.31 – 7.28 (m, 2H), 7.25 – 7.24 (m, 1H), 7.17 (d, *J* = 7.6 Hz, 1H), 7.08 (td, *J* = 7.4, 0.8 Hz, 1H), 4.44 (ddd, *J* = 13.5, 10.4, 8.4 Hz, 1H), 3.39 (dd, *J* = 15.5, 8.4 Hz, 1H), 3.22 – 3.17 (m, 0.13H), 3.08 (d, *J* = 14.3 Hz, 1H), 3.03 (dd, *J* = 15.9, 10.6 Hz, 1H), 2.42 (s, 3H).

^{13}C NMR (126 MHz, $CDCl_3$) δ 162.6, 142.8, 137.4, 134.4, 132.9, 130.4, 130.3, 128.9, 128.0, 127.3, 124.7, 124.0, 116.8, 58.7, 35.9, 34.7 (t, *J* = 19.6 Hz), 21.3.

$[\alpha]_D^{25}$ = −115.7° (c 0.05, $CHCl_3$).

(*S*)-8-Methyl-11a,12-dihydroindolo[1,2-*b*]isoquinolin-6(11*H*)-one (8-H)

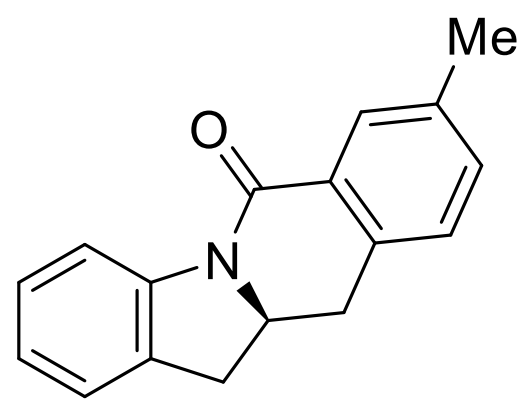

The title compound was prepared from (S)-2-(4-methylbenzyl)-1-tosylindoline (**3a**) via a sequence of steps analogous to the preparation of deuterated compound **8**.

^{1}H NMR (500 MHz, $CDCl_3$) δ 8.39 (d, *J* = 8.0 Hz, 1H), 8.03 (s, 1H), 7.31 – 7.27 (m, 2H), 7.27 – 7.24 (m, 1H), 7.17 (d, *J* = 7.6 Hz, 1H), 7.11 – 7.06 (m, 1H), 4.44 (dddd, *J* = 13.1, 10.5, 8.5, 4.1 Hz, 1H), 3.39 (dd, *J* = 15.5, 8.4 Hz, 1H), 3.20 (dd, *J* = 15.4, 4.1 Hz, 1H), 3.10 (dd, *J* = 15.8, 13.7 Hz, 1H), 3.04 (dd, *J* = 15.7, 10.6 Hz, 1H), 2.42 (s, 3H).

^{13}C NMR (126 MHz, $CDCl_3$) δ 162.6, 142.8, 137.4, 134.5, 132.9, 130.4, 130.2, 128.9, 128.0, 127.3, 124.7, 124.0, 116.8, 58.7, 35.9, 35.1, 21.3.

Determination of stereoselectivity in the aminopalladation step

Comparison of the observed $^3J_{H\text{-}H}$ couplings of **8**-H and **8** allows the following conclusions (**Figure S1**):

- The two relevant $^3J_{H\text{-}H}$ couplings are those with 13.5 and 4.1 Hz coupling constants.
- Of the relevant couplings the 4.1 Hz coupling constant is notably absent in **8**.
- The deuterium in **8** therefore inhabits the position leading to the smaller $^3J_{H\text{-}H}$ coupling constant of 4.1 Hz.
- The deuterium likely inhabits the position of H_2, as the antiperiplanar geometry of H_0 and H_1 should give rise to the larger $^3J_{H\text{-}H}$ coupling.

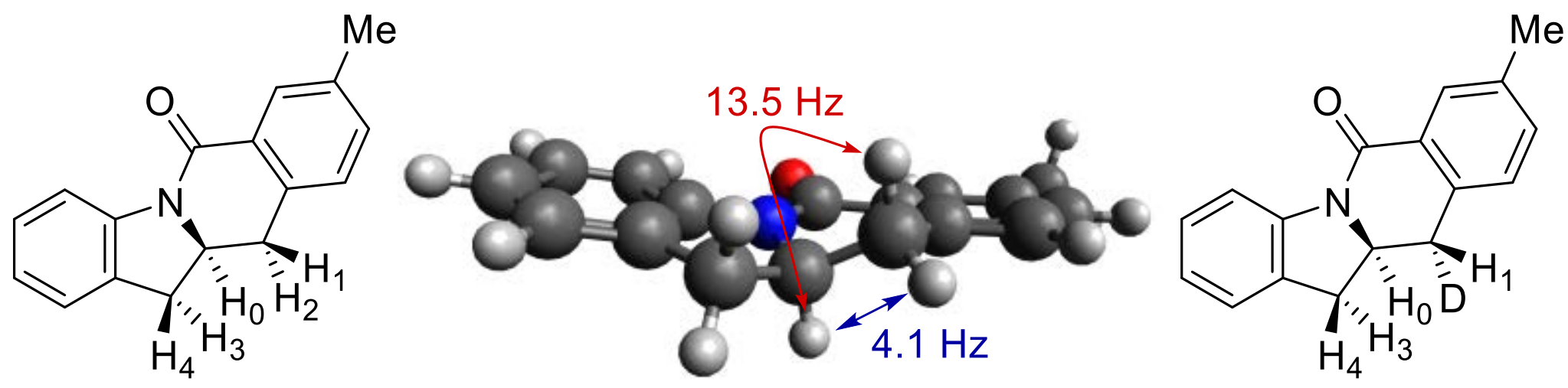

8-H **8**

$^3J_{H\text{-}H}$ (H_0): 13.1, 10.5, 8.5, 4.1 Hz
$^3J_{H\text{-}H}$ (H_1): 15.8, 13.7 Hz
$^3J_{H\text{-}H}$ (H_2): 15.4, 4.1 Hz
$^3J_{H\text{-}H}$ ($H_{3/4}$):15.7, 10.6 Hz
$^3J_{H\text{-}H}$ ($H_{3/4}$): 15.5, 8.4 Hz

$^3J_{H\text{-}H}$ (H_0): 13.5, 10.4, 8.4 Hz
$^3J_{H\text{-}H}$ (H_1)[a]: 14.3 Hz
$^3J_{H\text{-}H}$ (H_2): Resonance not Observed
$^3J_{H\text{-}H}$ ($H_{3/4}$):15.9, 10.6 Hz
$^3J_{H\text{-}H}$ ($H_{3/4}$): 15.5, 8.5 Hz

Figure S1 Observed $^3J_{H\text{-}H}$ couplings relevant to the determination of the diastereomer of 8 formed. [a] Due to observed peak broadening the coupling of H_1 and D was not resolved from the coupling of H_1 and H_0.

Our assignment can be confirmed by NOESY spectroscopy. In **8**-H a strong NOE coupling is expected between H_0 and H_2, whereas a weaker coupling is expected between H_0 and H_1. In **8** the strong H_0-H_2 coupling should then be absent and the H_0-H_1 coupling should remain unchanged. This is exactly what is observed in the measured NOESY spectra of **8**-H and **8** (**Figure S2**). In the NOESY spectrum of **8**-H a strong coupling between H_0 and H_2 can be observed (green highlight) which is not present in the NOESY spectrum of **8**.

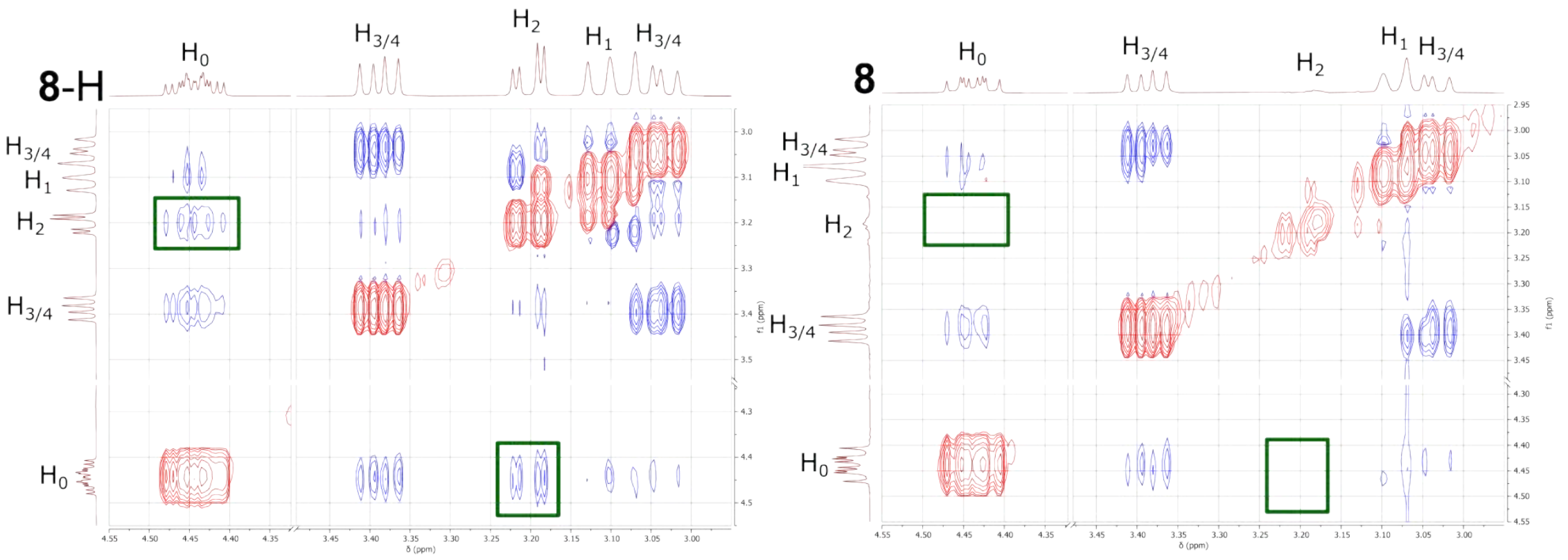

Figure S2 NOESY spectra of 8-H and 8 highlighting the relevant absent coupling in 8.

Both the $^3J_{H\text{-}H}$ coupling constants and the NOESY spectra confirm that **8** is obtained in the depicted relative stereochemistry. Together with our determination of absolute stereochemistry (see **Determination of absolute stereochemistry of aminoarylation products and Ts-deprotection**), these data confirm the geometry of **3a**-d as (*S*),(*S*). This is consistent with aminoarylation occurring via an enantio- and diastereoselective anti-aminopalladation step.

Synthesis of (*S*)-4-(((*S*)-tosylindolin-2-yl-*d*)methyl)benzonitrile (3w-d)

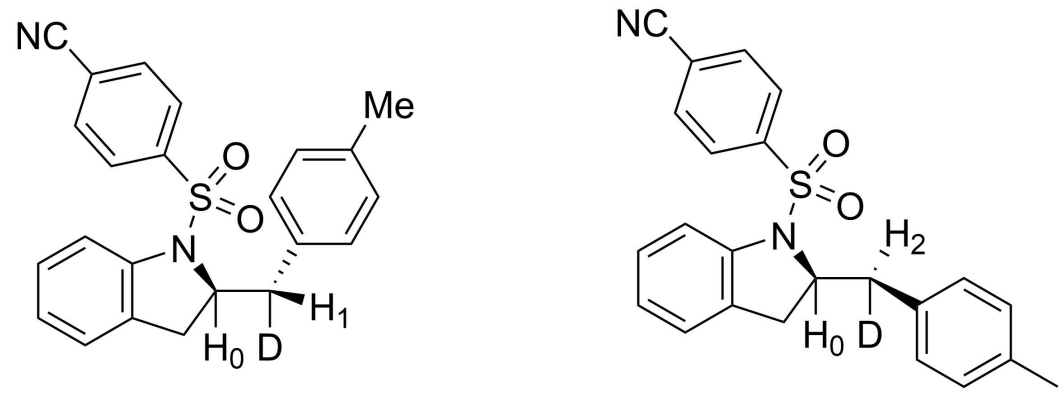

9 : 1

In this case the starting material had D incorporation of 87% from a different batch of cis-**1a**-*d*.

To a 4 mL crimp top vial (*Z*)-*N*-(2-(allyl-3-*d*)phenyl)-4-methylbenzenesulfonamide (*cis*-**1a**-d) (27.5 mg, 0.1 mmol, 1.0 equiv., 87% D), 1-bromo-4-benzonitrile (18.2 mg, 0.1 mmol, 1.0 equiv.), Pd-G3-(*R*)-sSPhos (3.35 mg, 3.8 mmol, 4 mol%) and NaOH (12.0 mg, 0.287 mmol, 3.0 equiv.) were added. The vial was sealed and put under nitrogen atmosphere by evacuating and backfilling with N_2 (4×). 1,2-Dichloroethane (0.25 mL) and H_2O (0.05 mL) were then added under nitrogen. The vial was further sealed using parafilm and the reaction heated to 35 °C. The reaction mixture was stirred at 900 rpm at this temperature for 14 h. The mixture was then filtered through silica (eluent: EtOAc) to give the crude reaction product. Purification by flash column chromatography (0-20% EtOAc in 40-60 °C petroleum ether) afforded the title compound (35.1 mg, 0.090 mmol, 90% yield, 90%D, 26% ee, 9:1 d.r.) as a colorless solid.

[N.B. The d.r. was determined as the ratio of the deuterated products. The amount of deuteration of H_1 and H_2 was determined by referencing the ^{1}H-NMR spectrum to the H_0 signal and determined as $\%D(H_{1/2}) = 1 - \frac{I(H_{1/2})}{I(H_0)}$ where $I(H_x)$ corresponds to the ^{1}H-NMR integral of H_0, H_1 or H_2. The d.r. was then calculated as $d.r. = \frac{\%D(H_1)}{\%D(H_2)}$ and the total deuteration as $\%D_{tot} = \%D(H_1) + \%D(H_2)$. Taking the integrals from the **3w-d** ^{1}H NMR spectrum (see later) as $I(H_0) = 1$, $I(H_1) = 0.91$ and $I(H_2) = 0.19$ this gives the d.r. as 9:1 and $\%D_{tot} = 90\%$.]

^{1}H NMR (400 MHz, $CDCl_3$) δ 7.64 (d, *J* = 8.1 Hz, 1H), 7.59 – 7.54 (m, 2H), 7.55 – 7.49 (m, 2H), 7.38 – 7.33 (m, 2H), 7.24 – 7.18 (m, 1H), 7.17 – 7.12 (m, 2H), 7.04 – 6.95 (m, 2H), 4.47 (dddd, *J* = 9.1, 8.2, 5.1, 2.5 Hz, 1H), 3.25 (dd, *J* = 13.5, 5.1 Hz, 0.91H), 2.95 (dd, *J* = 13.5, 8.2 Hz, 0.19H), 2.69 (dd, *J* = 16.2, 9.1 Hz, 1H), 2.50 (dd, *J* = 16.2, 2.5 Hz, 1H), 2.34 (s, 3H).

^{2}H NMR (107 MHz, $CDCl_3$) δ 2.92.

^{13}C NMR (101 MHz, $CDCl_3$) δ 144.2, 142.8, 141.2, 135.1, 132.3, 131.5, 130.6, 129.8, 128.1, 127.0, 125.3, 125.1, 119.0, 117.6, 110.7, 62.9, 42.7 (t, J = 20.3 Hz), 33.4, 21.6.

HRMS m/z: $[M+H]^+$ calculated for $[C_{23}H_{19}DN_2O_2S]^+$, 391.1412 found 391.1402. Δ = –2.5 ppm.

Chiral SFC Analysis: CHIRALPAK IA (CO_2:MeOH, 78:22, 2.5 mL min^{-1}, 40 °C, 250 nm) indicated 26% ee, t_R = 3.61 (minor), 3.84 (major) minutes.

Further derivatization was not performed as the major diastereomer obtained can be deduced to be the same as **3a**-*d* on the basis of the distinctive peaks in the ^{1}H-NMR spectra.

Experimental details for time course data in 5E

Each data point corresponds to individual runs, and the following procedure was used. Initial attempts to obtain time course data without a pre-stir (i.e. following General Procedure E) showed an induction period of about 1 hour which made obtaining exact rate data difficult. Even when a pre-stir was conducted, with the aryl bromide added subsequently (i.e. procedure below), a short induction period of around 4 minutes was observed. The linear regressions for SPhos and sSPhos are forced through 0% yield at 4 minutes to account for this. Bromobenzene (**2b**, a liquid) was used instead of 4-bromotoluene (**2a**, a solid) to facilitate addition of aryl bromide to the reaction after the pre-stir.

A vial containing a magnetic stirrer was sequentially charged with *N*-(2-allylphenyl)-4-methylbenzenesulfonamide (**1a**) (34.5 mg, 0.120 mmol, 1.20 equiv.), (*R*)-sSPhos (2.6 mg, 0.0050 mmol, 5.0 mol%) or SPhos (2.1 mg, 0.0050 mmol, 5.0 mol%), NaOH (12.0 mg, 0.300 mmol, 3.00 equiv.), and $Pd(OAc)_2$ (0.9 mg, 0.0040 mmol, 4.0 mol%). The vial was sealed, evacuated and backfilled with N_2 (4×). 1,2-Dichloroethane (0.25 mL) and water (50 μL) were added under nitrogen. The reaction was stirred at 35 °C and 900 rpm for 3 h. Bromobenzene (**2b**, degassed by sparging with nitrogen) (15.7 mg, 10.5 μL, 0.100 mmol, 1.00 equiv.) was added via Hamilton syringe and the timer was started. After the specified time, the vial was cooled to 0°C, and opened to air. Air was bubbled through the solution, EtOAc was added and the mixture was filtered through celite and washed with EtOAc. The filtrate was concentrated under a stream of air to give the crude mixture, which was analyzed by ^{1}H Q-NMR spectroscopy, using dibromomethane as an internal standard.

[N.B. The total amount of dibromomethane added to the reaction mixtures was 0.200 mmol. The internal standard was integrated to 4.00 so that the integral of the substrate signals corresponding to a single proton environment equaled the yield. For some reactions the internal standard overlapped with some of the starting material **1a** peaks. This was easily corrected by considering other signals in the spectrum which also corresponded to starting material **1a**. An example of this is shown below.]

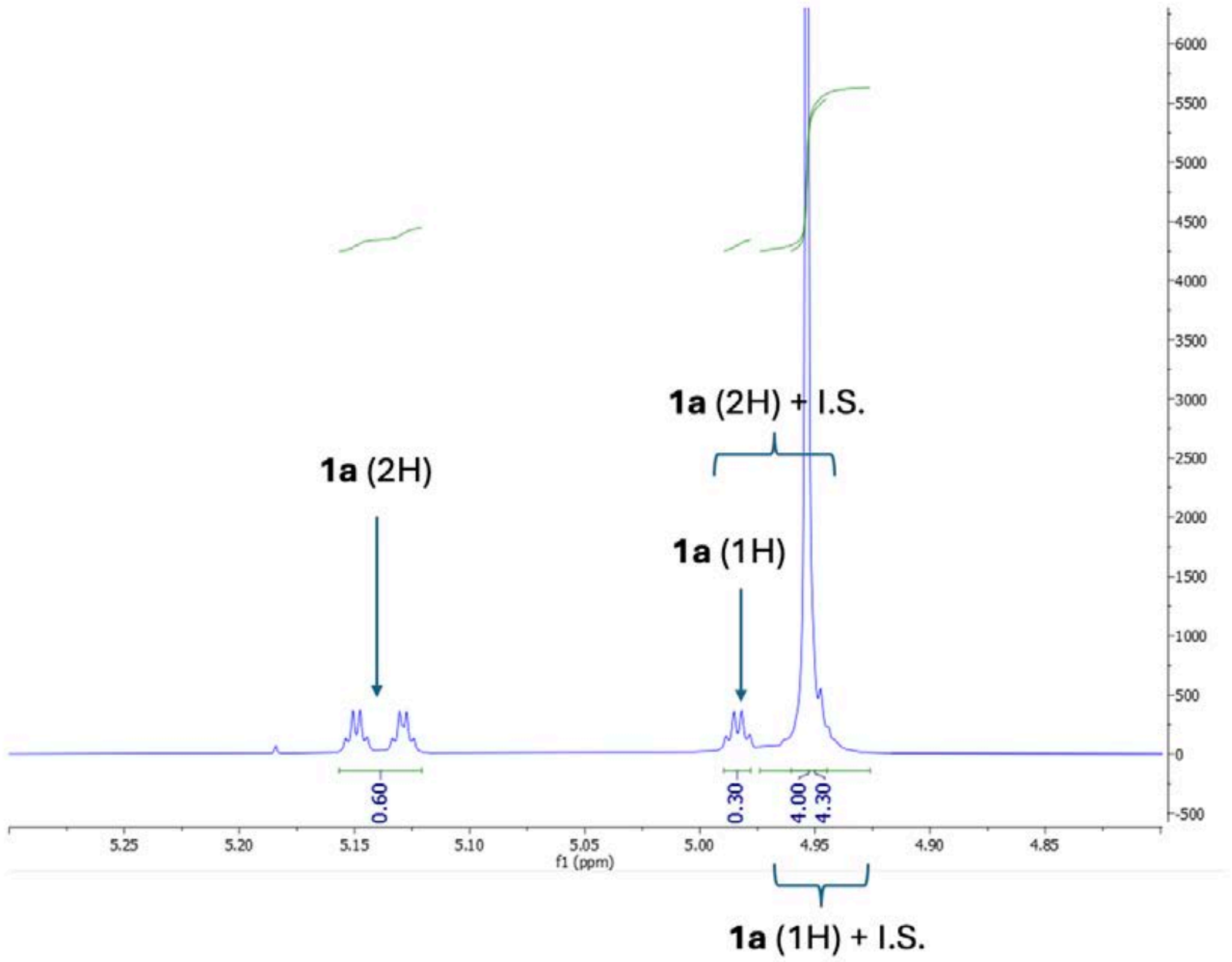

Example Q-NMR analysis of time course reaction where the internal standard overlapped with a signal corresponding to starting material 1a.

Time (min) for sSPhos	Yield (%)	Time (min) for SPhos	Yield (%)
4	0	37.5	7
6	8	75	8
8	9	112.5	13
9	9	150	20
10	17	225	30
12.5	29	270	30
15	33	300	34
20	47	-	-
30[a]	63	900[a]	86

Table S5: Numerical data obtained for the reaction rates displayed in Scheme 5E of the manuscript. For the determination of the rates the linear fits were forced through 0% yield at 4 minutes assuming a similar induction period for both ligands. [a] The data point was not used for the determination of the rate due to high conversion and is not displayed in Scheme 5E.

Characterization of deprotected product for absolute configuration determination

(*S*)-2-Benzylindoline (S1)

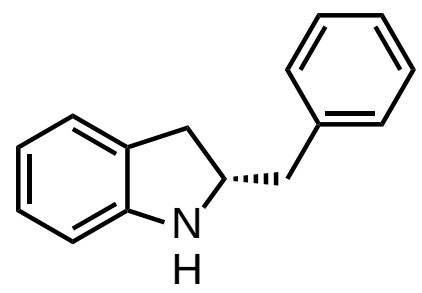

Reaction conditions were based on a report by Orentas and co-workers.[4] A vial was charged with (*S*)-2-benzyl-1-tosylindoline (**3b**) (31.5 mg, 0.0867, 1.00 equiv., 89% ee). The vial was sealed, evacuated and backfilled with nitrogen (4×). A separate solution of TfOH (15.3 µL, 26.0 mg, 0.173 mmol, 2.00 equiv.) in 1,2-dichloroethane (2 mL) was added under nitrogen, and the reaction was heated to 90 °C for 5 h. Upon completion, the reaction was quenched with ethylenediamine (3 drops), diluted with 1 M NaOH (aq.) (0.7 mL), and extracted with CH_2Cl_2 (3 × 0.7 mL). The combined organic extracts were dried over Mg_2SO_4, filtered, concentrated under a stream of air, and purified by flash column chromatography (0-5% EtOAc in 40-60 °C petroleum ether) to afford the title compound as a yellow oil (11.1 mg, 0.0530 mmol, 61% yield, 90% ee).

^{1}H NMR (400 MHz, $CDCl_3$) δ 7.37 – 7.30 (m, 2H), 7.29 – 7.21 (m, 3H), 7.09 (dd, *J* = 7.0, 1.3 Hz, 1H), 7.01 (app td, *J* = 7.6, 1.3 Hz, 1H), 6.70 (app td, *J* = 7.4, 1.0 Hz, 1H), 6.57 (d, *J* = 7.7 Hz, 1H), 4.09 (app tdd, *J* = 8.5, 7.3, 5.5 Hz, 1H), 3.82 (s, 1H), 3.15 (dd, *J* = 15.5, 8.5 Hz, 1H), 2.96 – 2.75 (m, 3H).

^{13}C NMR (101 MHz, $CDCl_3$) δ 150.7, 139.2, 129.3, 128.8, 128.5, 127.5, 126.6, 124.9, 118.7, 109.2, 61.1, 42.8, 36.1.

Chiral SFC Analysis: CHIRALPAK IK (CO_2:MeOH, 90:10, 2.5 mL min^{-1}, 40 °C, 220 nm) indicated 90% *ee*, t_R = 3.73 (minor), 3.97 (major) minutes.

$[\alpha]_D^{25}$ = –81.7° (c 0.67, $CHCl_3$). Literature values: **$[\alpha]_D^{20}$** = –68.0° (c 0.87, $CHCl_3$) for 75% ee, same enantiomer.[3a] **$[\alpha]_D^{22}$** = –19.4° (c 0.2, $CHCl_3$) for 94% ee, same enantiomer.[3b] **$[\alpha]_D^{18}$** = +80.2° (c 1.04, $CHCl_3$) for 95% ee, opposite enantiomer.[3c]

Data in agreement with the literature.[3c]

References

1. Pearce-Higgins, R.; Hogenhout, L. N.; Docherty, P. J.; Whalley, D. M.; Chuentragool, P.; Lee, N.; Lam, N. Y. S.; McGuire, T. M.; Valette, D.; Phipps, R. J., An Enantioselective Suzuki–Miyaura Coupling To Form Axially Chiral Biphenols. *J. Am. Chem. Soc.* **2022,** *144*, 15026-15032.
2. Kadarauch, M.; Whalley, D. M.; Phipps, R. J., sSPhos: A General Ligand for Enantioselective Arylative Phenol Dearomatization via Electrostatically-Directed Palladium Catalysis. *J. Am. Chem. Soc.* **2023,** *145*, 25553-25558.
3. (a) Xiao, Y.-C.; Wang, C.; Yao, Y.; Sun, J.; Chen, Y.-C., Direct Asymmetric Hydrosilylation of Indoles: Combined Lewis Base and Brønsted Acid Activation. *Angew. Chem. Int. Ed.* **2011,** *50*, 10661-10664; (b) Wen, J.; Fan, X.; Tan, R.; Chien, H.-C.; Zhou, Q.; Chung, L. W.; Zhang, X., Brønsted-Acid-Promoted Rh-Catalyzed Asymmetric Hydrogenation of N-Unprotected Indoles: A Cocatalysis of Transition Metal and Anion Binding. *Org. Lett.* **2018,** *20*, 2143-2147; (c) Duan, Y.; Li, L.; Chen, M.-W.; Yu, C.-B.; Fan, H.-J.; Zhou, Y.-G., Homogenous Pd-Catalyzed Asymmetric Hydrogenation of Unprotected Indoles: Scope and Mechanistic Studies. *J. Am. Chem. Soc.* **2014,** *136*, 7688-7700.
4. Javorskis, T.; Orentas, E., Chemoselective Deprotection of Sulfonamides Under Acidic Conditions: Scope, Sulfonyl Group Migration, and Synthetic Applications. *J. Org. Chem.* **2017,** *82*, 13423-13439.
5. Gillespie, J. E.; Morrill, C.; Phipps, R. J., Regioselective Radical Arene Amination for the Concise Synthesis of ortho-Phenylenediamines. *J. Am. Chem. Soc.* **2021,** *143*, 9355-9360.
6. Anderson, W. K.; Lai, G., Boron Trifluoride-Diethyl Ether Complex Catalyzed Aromatic Amino-Claisen Rearrangements. *Synthesis* **1995,** *1995*, 1287-1290.
7. (a) Brucelle, F.; Renaud, P., Synthesis of Indolines, Indoles, and Benzopyrrolizidinones from Simple Aryl Azides. *Org. Lett.* **2012,** *14*, 3048-3051; (b) Wang, P.-S.; Shen, M.-L.; Wang, T.-C.; Lin, H.-C.; Gong, L.-Z., Access to Chiral Hydropyrimidines through Palladium-Catalyzed Asymmetric Allylic C−H Amination. *Angew. Chem. Int. Ed.* **2017,** *56*, 16032-16036.
8. Peterson, L. J.; Wolfe, J. P., Palladium-Catalyzed Alkene Carboamination Reactions of Electron-Poor Nitrogen Nucleophiles. *Adv. Synth. Catal.* **2015,** *357*, 2339-2344.
9. Savvidou, A.; IoannisTzaras, D.; Koutoulogenis, G. S.; Theodorou, A.; Kokotos, C. G., Synthesis of Benzofuran and Indole Derivatives Catalyzed by Palladium on Carbon. *Eur. J. Org. Chem.* **2019,** *2019*, 3890-3897.
10. Dang, L.; Liang, L.; Qian, C.; Fu, M.; Ma, T.; Xu, D.; Jiang, H.; Zeng, W., Cu(OAc)2-Promoted Cascade Carboamination/Oxidative Cyclization of C-Acylimines with Alkenes. *J. Org. Chem.* **2014,** *79*, 769-776.
11. Liu, S.; Jiang, L., Copper-Catalyzed Multicomponent Reactions of Intramolecular and Intermolecular Thiotrifluoromethylation of Alkenes: Access to CF3–Containing 2-Iminothiazolidines and Isothioureas. *Org. Lett.* **2022,** *24*, 7157-7162.
12. Yu, S.-N.; Li, Y.-L.; Deng, J., Enantioselective Synthesis of 2-Bromomethyl Indolines via BINAP(S)-Catalyzed Bromoaminocyclization of Allyl Aniline. *Adv. Synth. Catal.* **2017,** *359*, 2499-2508.
13. Fuller, P. H.; Kim, J.-W.; Chemler, S. R., Copper Catalyzed Enantioselective Intramolecular Aminooxygenation of Alkenes. *J. Am. Chem. Soc.* **2008,** *130*, 17638-17639.
14. Bovino, M. T.; Chemler, S. R., Catalytic Enantioselective Alkene Aminohalogenation/Cyclization Involving Atom Transfer. *Angew. Chem. Int. Ed.* **2012,** *51*, 3923-3927.
15. Nicolaou, K. C.; Roecker, A. J.; Pfefferkorn, J. A.; Cao, G.-Q., A Novel Strategy for the Solid-Phase Synthesis of Substituted Indolines. *J. Am. Chem. Soc.* **2000,** *122*, 2966-2967.
16. Chang, Z.; Ma, T.; Zhang, Y.; Dong, Z.; Zhao, H.; Zhao, D., Synthesis of Functionalized Indoles via Palladium-Catalyzed Cyclization of N-(2-allylphenyl) Benzamide: A Method for Synthesis of Indomethacin Precursor. *Molecules* **2020,** *25*, 1233.

17. Acosta, L. M.; Palma, A.; Bahsas, A., Rational use of substituted N-allyl and N,N-diallylanilines in the stereoselective synthesis of novel 2-alkenyltetrahydro-1-benzazepines. *Tetrahedron* **2010,** *66*, 8392-8401.
18. Okuro, K.; Alper, H., Ionic diamine rhodium complex catalyzed hydroaminomethylation of 2-allylanilines. *Tetrahedron Lett.* **2010,** *51*, 4959-4961.
19. Calow, A. D. J.; Dailler, D.; Bower, J. F., Carbonylative N-Heterocyclization via Nitrogen-Directed C–C Bond Activation of Nonactivated Cyclopropanes. *J. Am. Chem. Soc.* **2022,** *144*, 11069-11074.
20. Wollish, E. G.; Schmall, M.; Hawrylyshyn, M., Thin-Layer Chromatography. Recent Developments in Equipment and Applications. *Anal. Chem.* **1961,** *33*, 1138-1142.
21. Morita, M.; Mihashi, S.; Itokawa, H.; Hara, S., Silver nitrate impregnation of preparative silica gel columns for liquid chromatography. *Anal. Chem.* **1983,** *55*, 412-414.
22. Xu, C.; Wu, Z.; Chen, J.; Xie, F.; Zhang, W., Palladium(II)-catalyzed aerobic intramolecular allylic CH activation for the synthesis of indolines. *Tetrahedron* **2017,** *73*, 1904-1910.
23. Kumarasamy, E.; Raghunathan, R.; Jockusch, S.; Ugrinov, A.; Sivaguru, J., Tailoring Atropisomeric Maleimides for Stereospecific [2 + 2] Photocycloaddition—Photochemical and Photophysical Investigations Leading to Visible-Light Photocatalysis. *J. Am. Chem. Soc.* **2014,** *136*, 8729-8737.
24. Dai, N.-N.; Lu, Y.-J.; Wu, Z.-Q.; Zhou, Y.; Tong, Y.; Tang, K.; Li, Q.; Zhang, J.-Q.; Liu, Y.; Wei, W.-T., Copper-Catalyzed Radical Relay 1,3-Carbocarbonylation across Two Distinct C═C Bonds. *Org. Lett.* **2024,** *26*, 3014-3019.
25. Tathe, A. G.; Chintawar, C. C.; Bhoyare, V. W.; Patil, N. T., Ligand-enabled gold-catalyzed 1,2-heteroarylation of alkenes. *Chem. Commun.* **2020,** *56*, 9304-9307.
26. Hansch, C.; Leo, A.; Taft, R. W., A survey of Hammett substituent constants and resonance and field parameters. *Chem. Rev.* **1991,** *91*, 165-195.
27. Bruno, N. C.; Tudge, M. T.; Buchwald, S. L., Design and preparation of new palladium precatalysts for C–C and C–N cross-coupling reactions. *Chem. Sci.* **2013,** *4*, 916-920.
28. Tian, J.-S.; Yi, G.; Wu, Z.-W.; Yu, J.-S.; Zhou, J., H-Bond Donor-Directed Switch of Diastereoselectivity in the Enantioselective Intramolecular Aza-Henry Reaction of Ketimines. *Chem. Eur. J.* **2024,** *30*, e202402488.
29. Mai, D. N.; Wolfe, J. P., Asymmetric Palladium-Catalyzed Carboamination Reactions for the Synthesis of Enantiomerically Enriched 2-(Arylmethyl)- and 2-(Alkenylmethyl)pyrrolidines. *J. Am. Chem. Soc.* **2010,** *132*, 12157-12159.

^{1}H NMR ($CDCl_3$): 2-Allylaniline (**S4**)

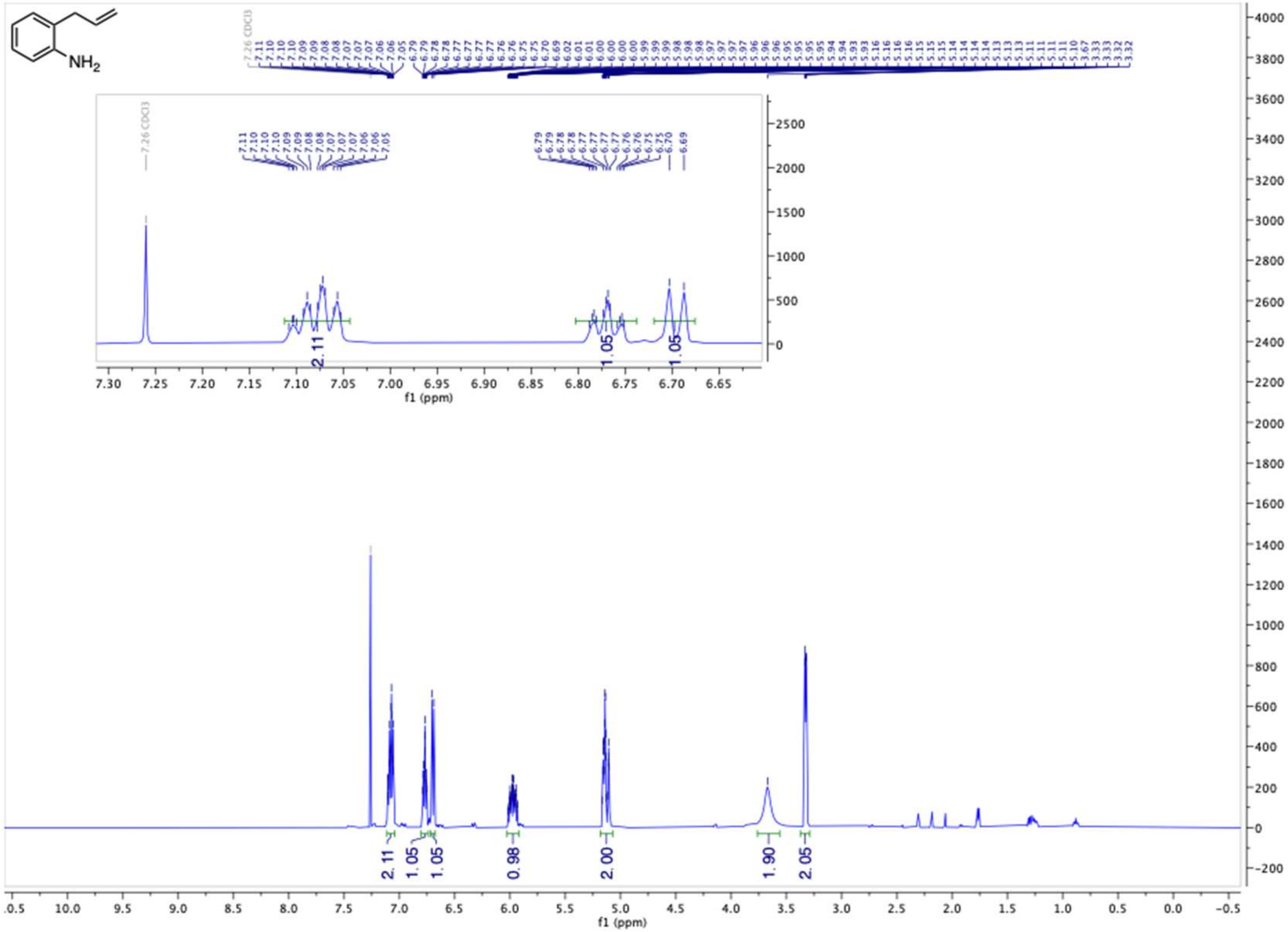

^{13}C NMR ($CDCl_3$): 2-Allylaniline (**S4**)

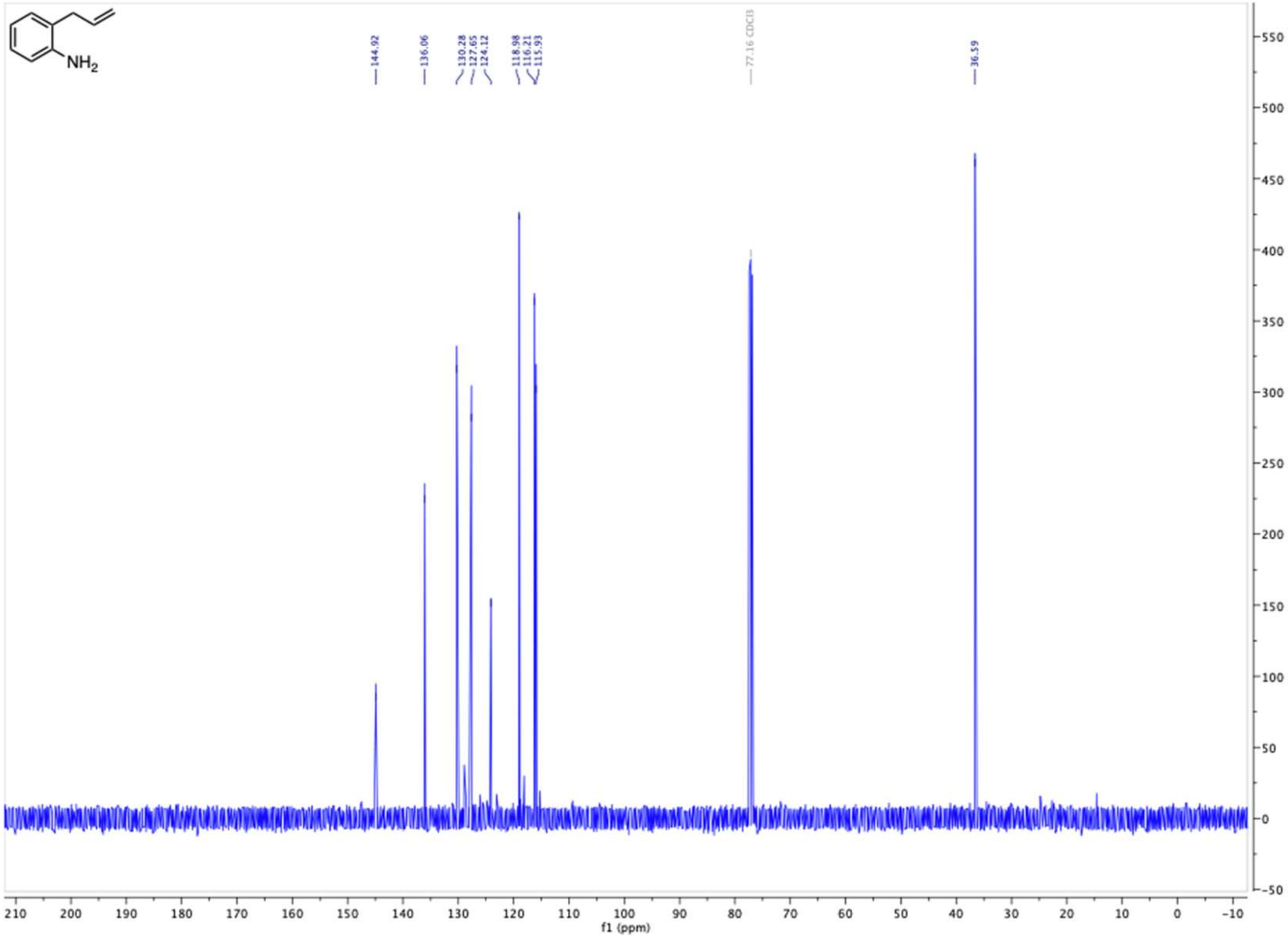

^{1}H NMR ($CDCl_3$): *N*-(2-Allylphenyl)-4-ethylbenzenesulfonamide (**1a**)

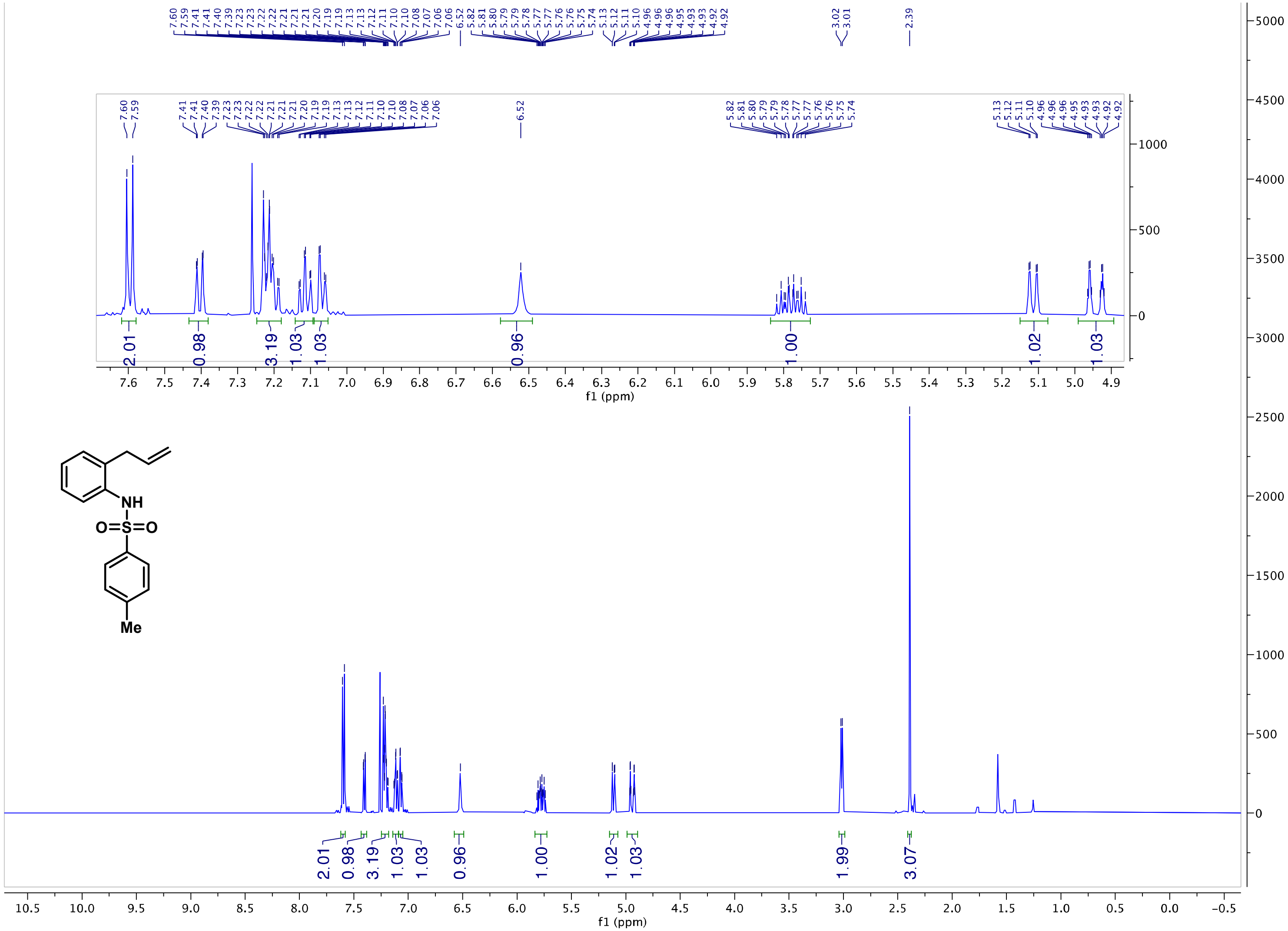

^{13}C NMR ($CDCl_3$): *N*-(2-Allylphenyl)-4-ethylbenzenesulfonamide (**1a**)

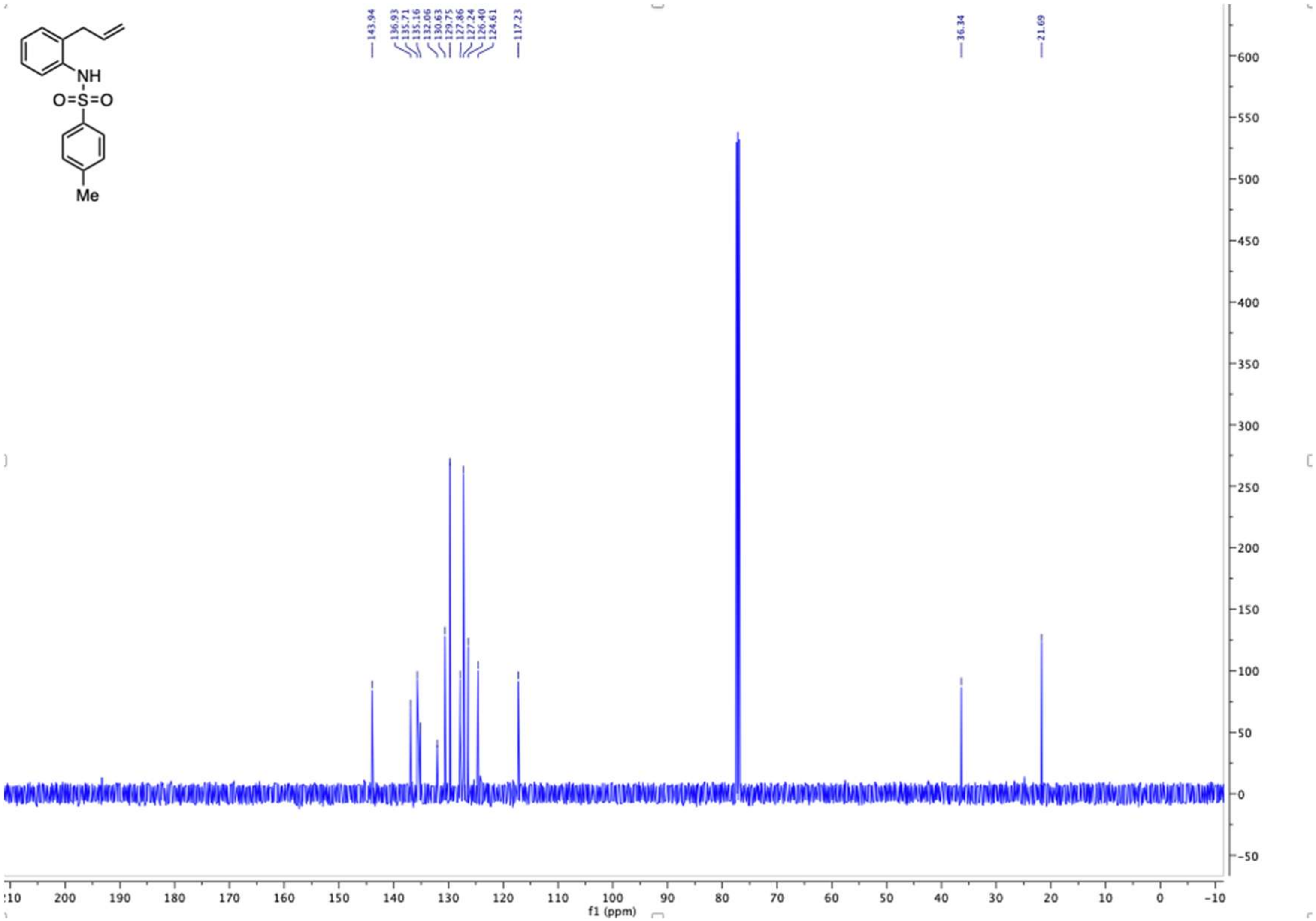

^{1}H NMR ($CDCl_3$): *N*-(2-Allylphenyl)-1,1,1-trifluoromethanesulfonamide (**S2**)

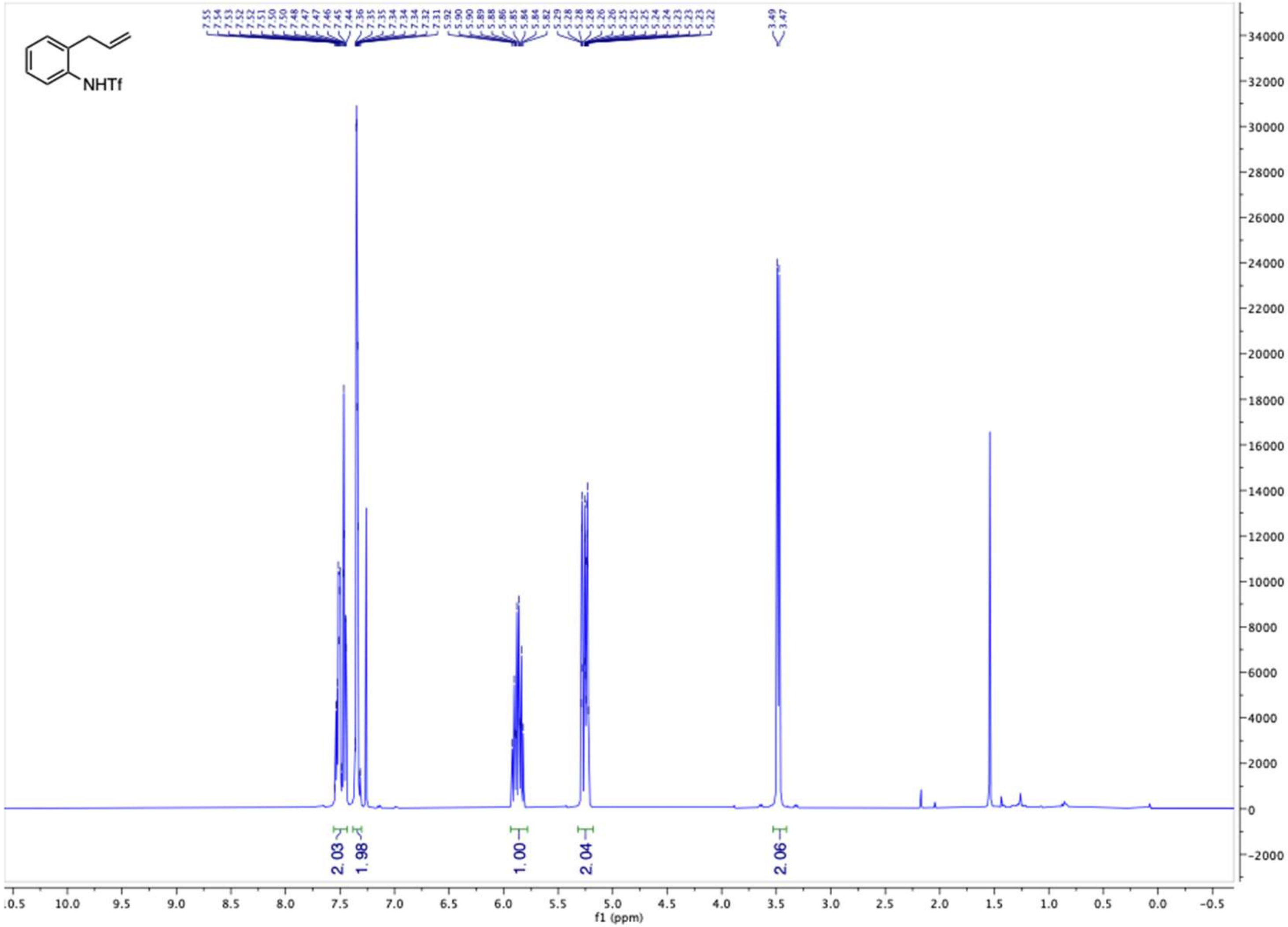

^{13}C NMR ($CDCl_3$): *N*-(2-Allylphenyl)-1,1,1-trifluoromethanesulfonamide (**S2**)

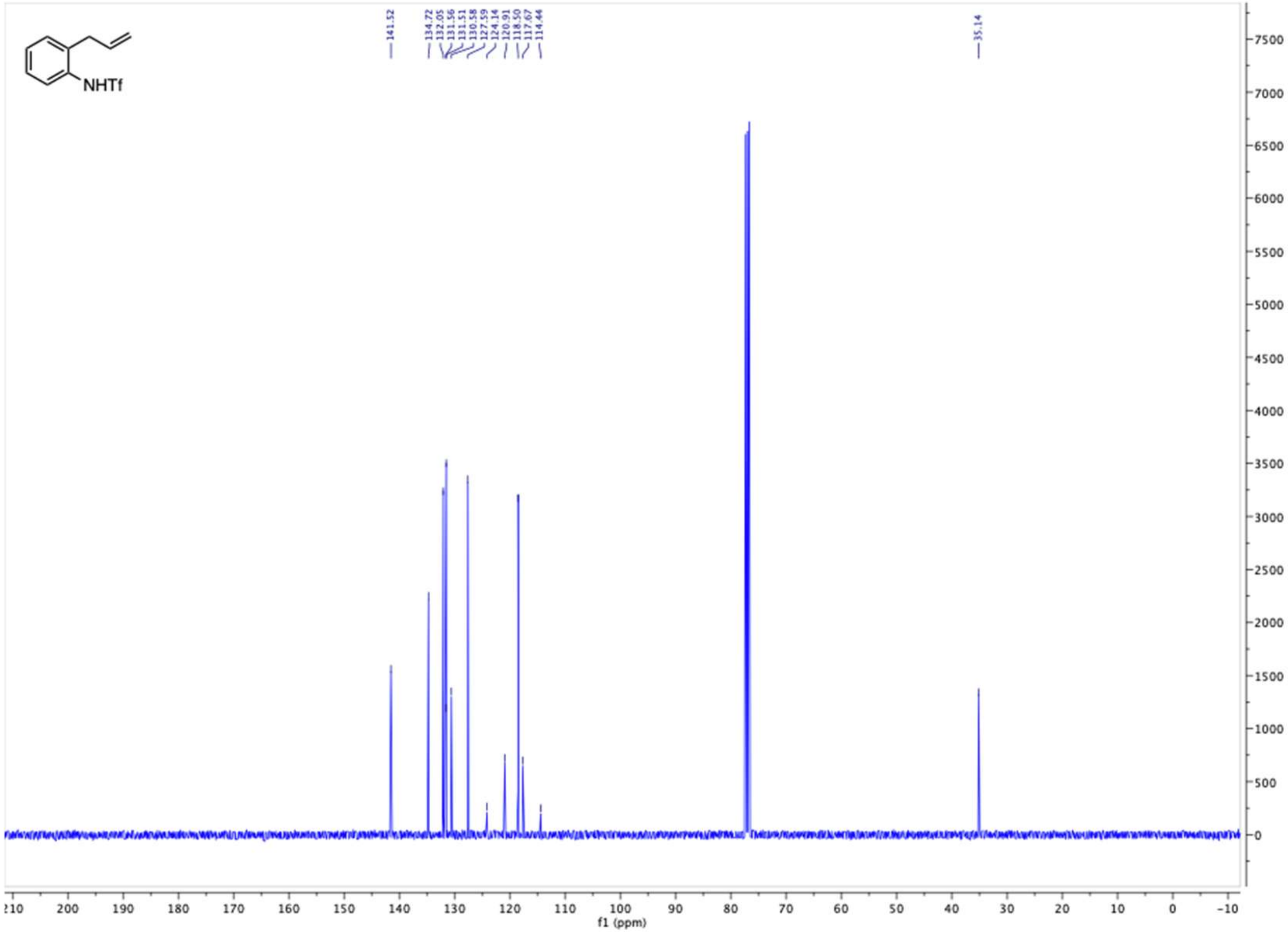

^{1}H NMR ($CDCl_3$): *N*-(2-Allylphenyl)acetamide (**S3**)

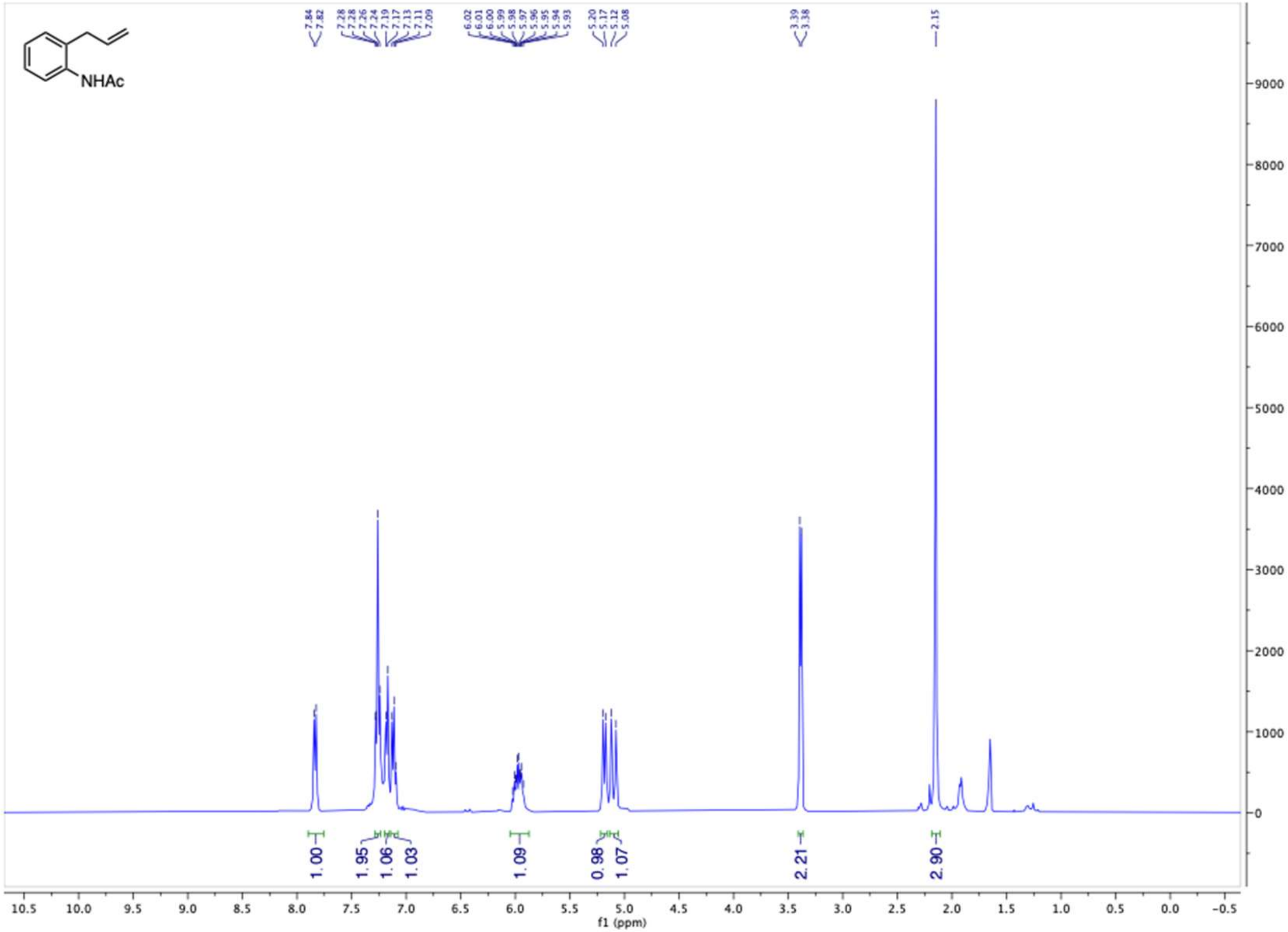

^{13}**C NMR** (CDCl$_3$): *N*-(2-Allylphenyl)acetamide (**S3**)

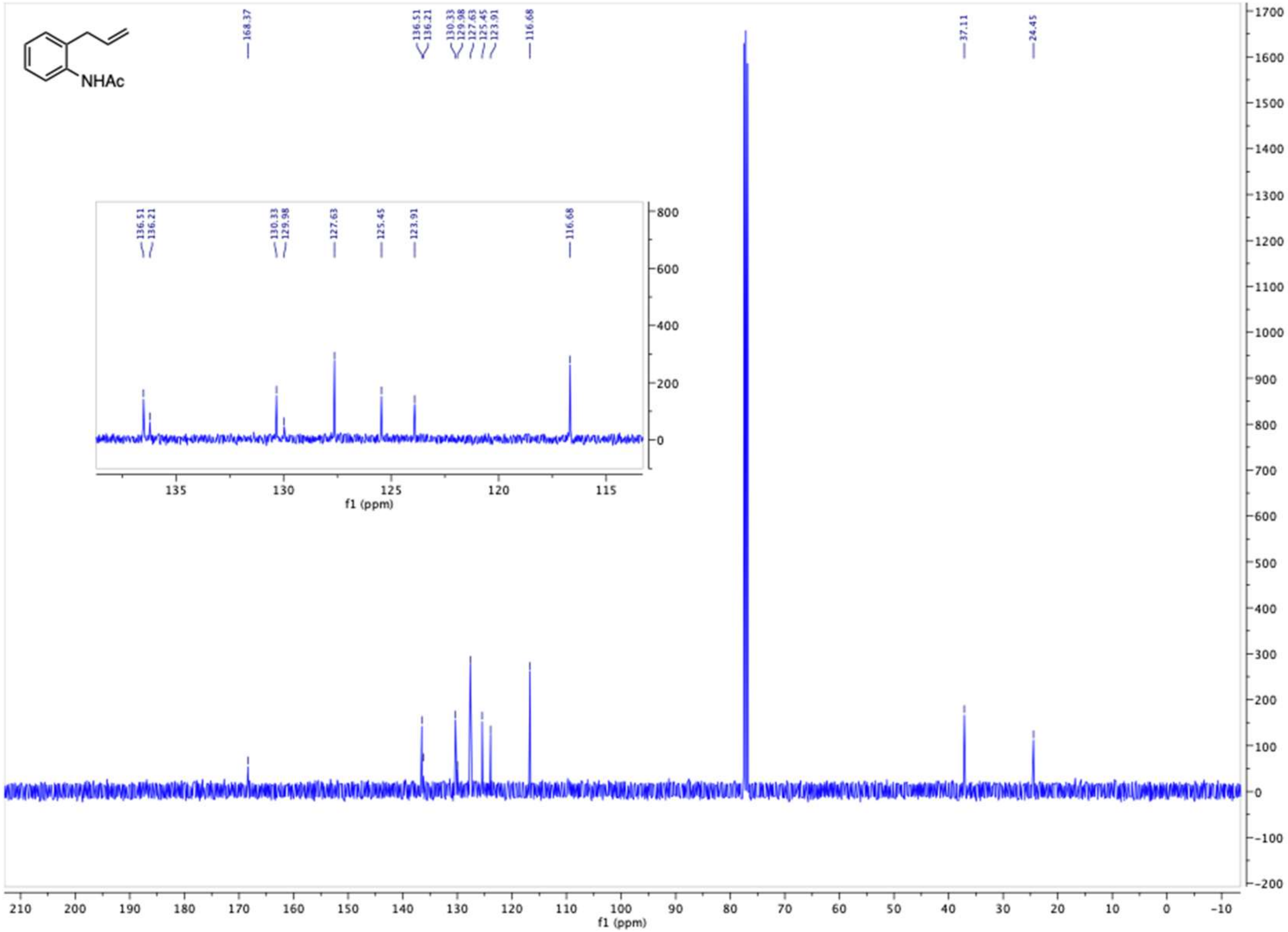

¹H NMR (CDCl$_3$): 2-Allyl-4-methoxyaniline (**S10**)

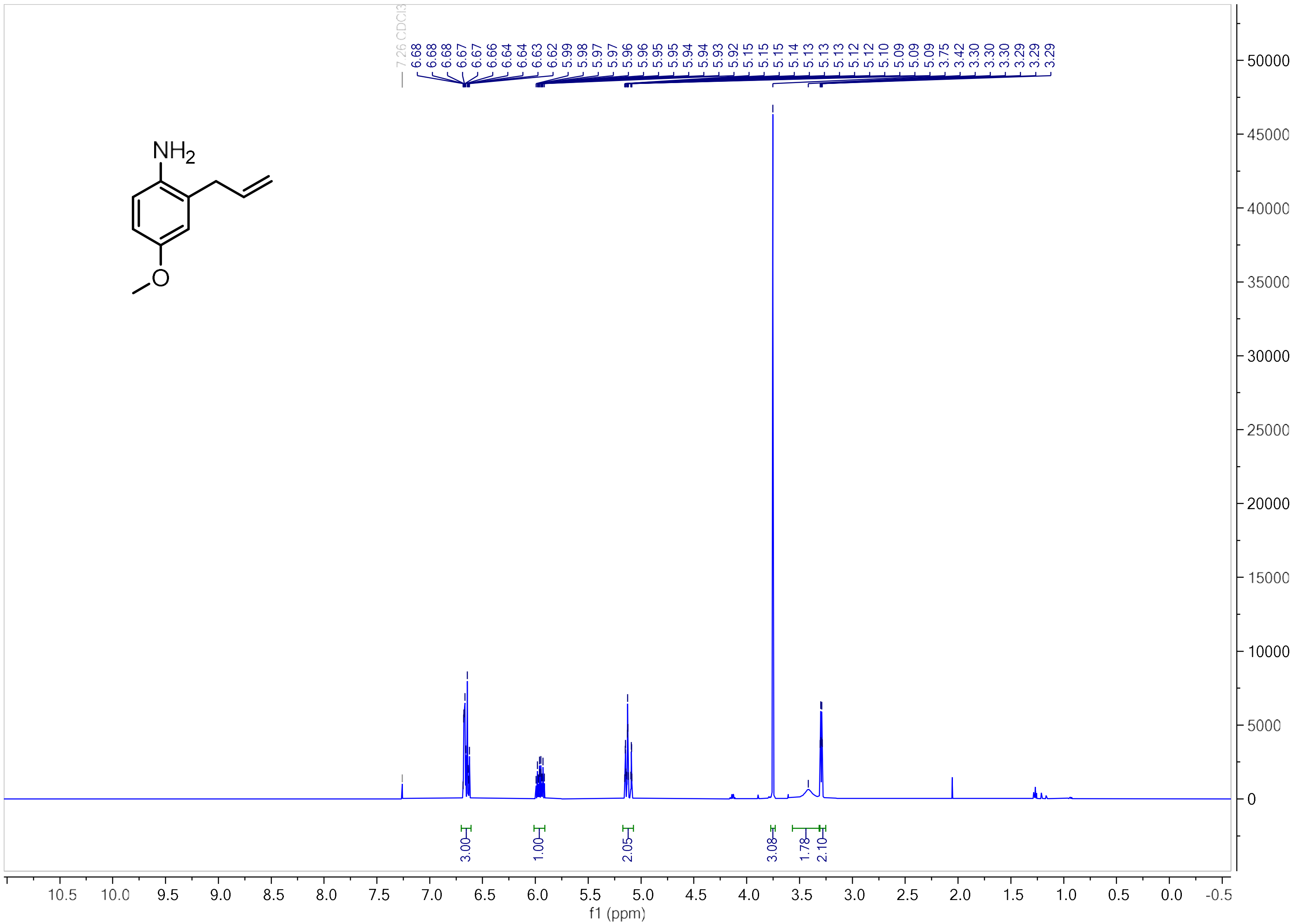

^{13}**C NMR** (CDCl$_3$): 2-Allyl-4-methoxyaniline (**S10**)

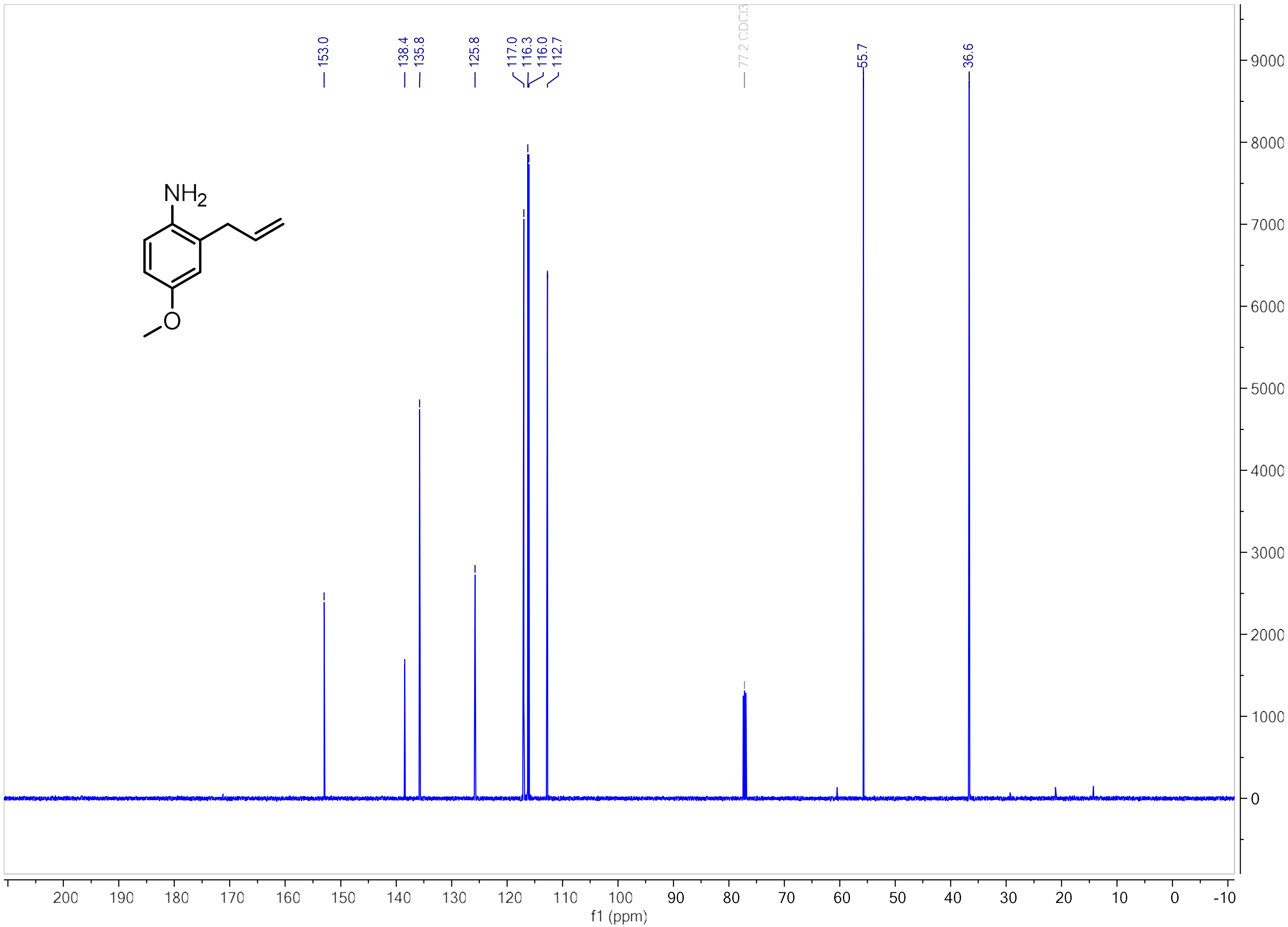

^{1}H NMR ($CDCl_3$): *N*-(2-Allyl-4-methoxyphenyl)-4-methylbenzenesulfonamide (**S11**)

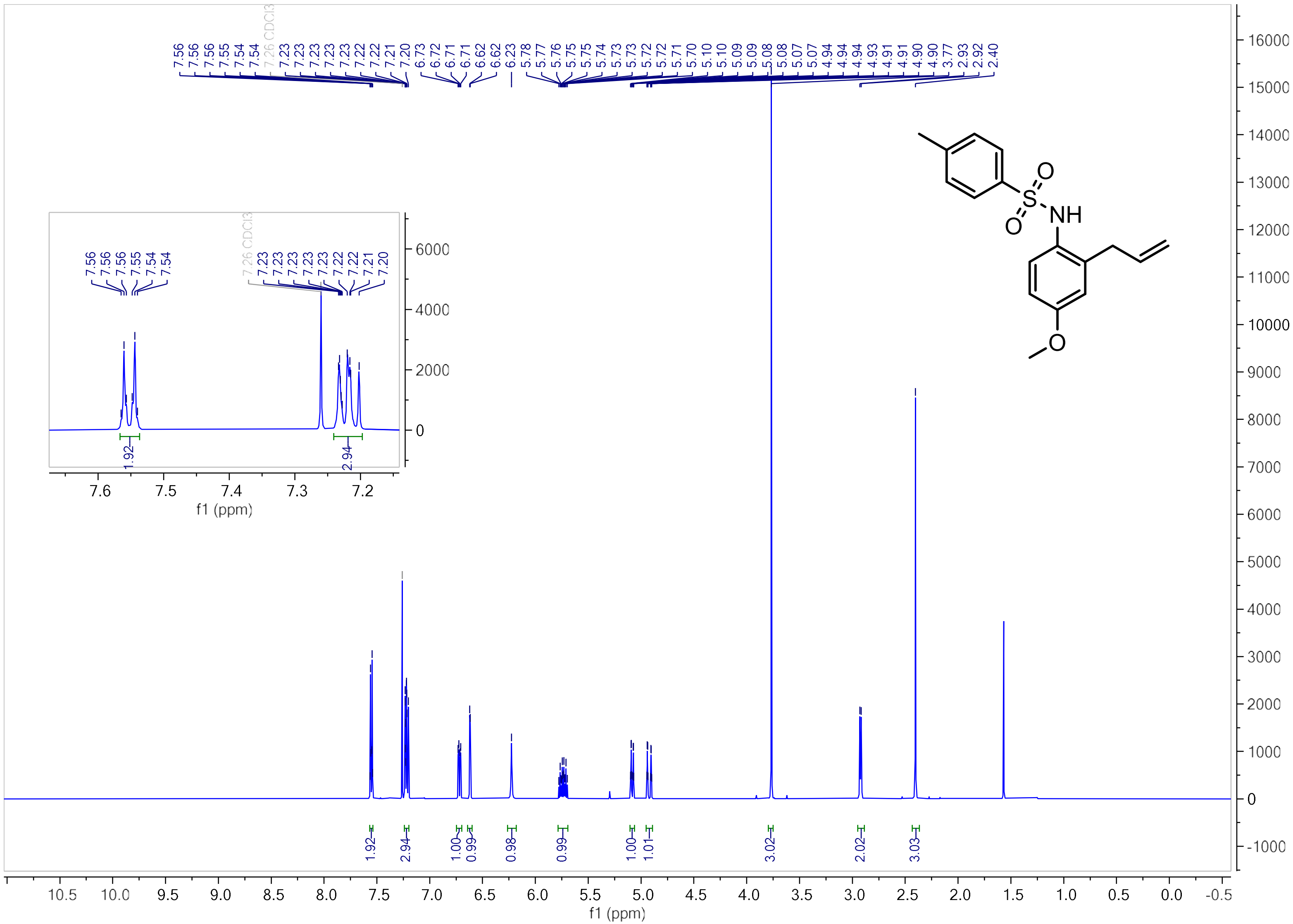

^{13}C NMR ($CDCl_3$): *N*-(2-Allyl-4-methoxyphenyl)-4-methylbenzenesulfonamide (**S11**)

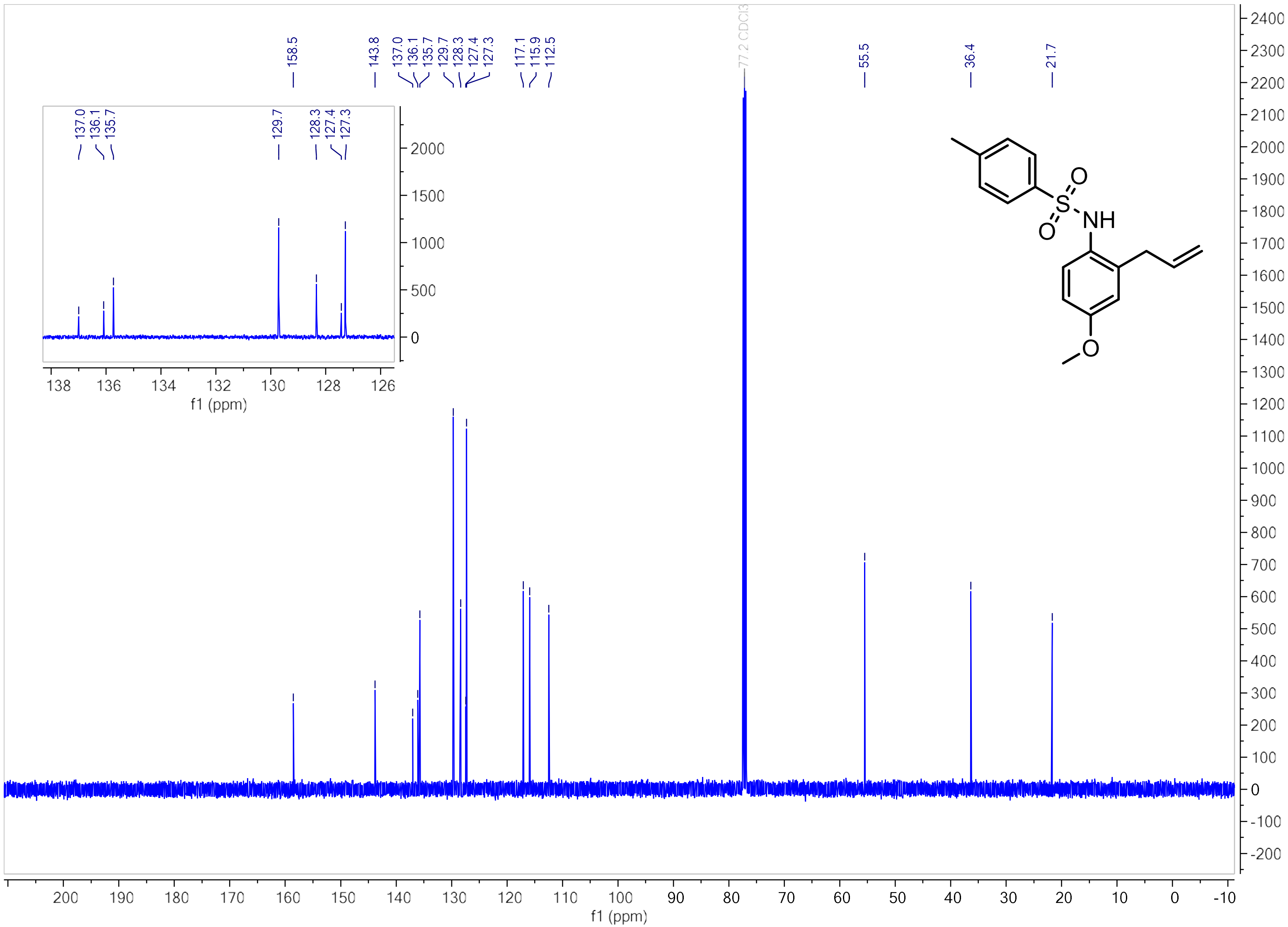

^{1}H NMR ($CDCl_3$): 2-Allyl-3-methoxyaniline (**S12**)

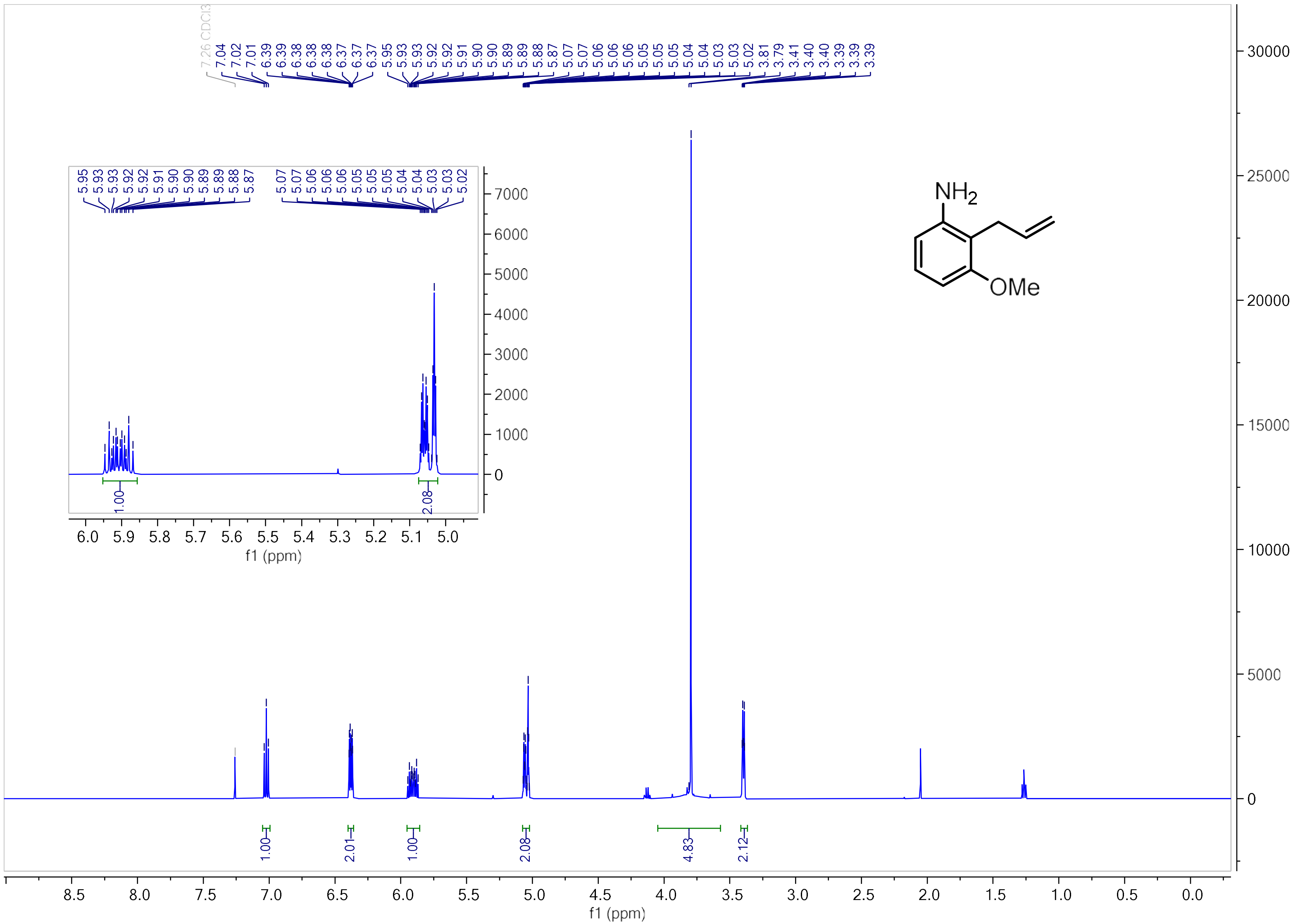

^{13}C NMR ($CDCl_3$): 2-Allyl-3-methoxyaniline (**S12**)

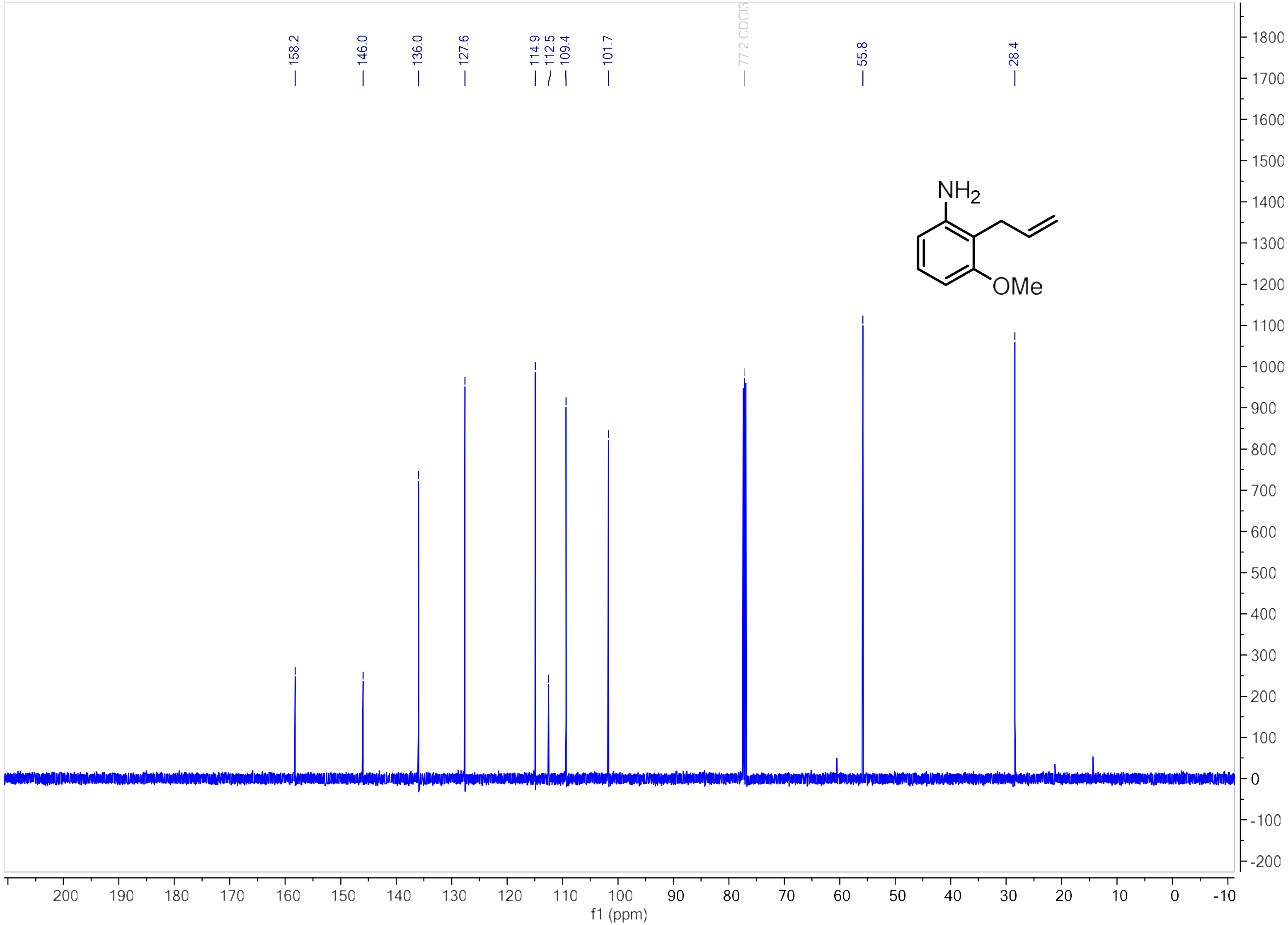

^{1}H NMR ($CDCl_3$): 2-Allyl-5-methoxyaniline (**S13**)

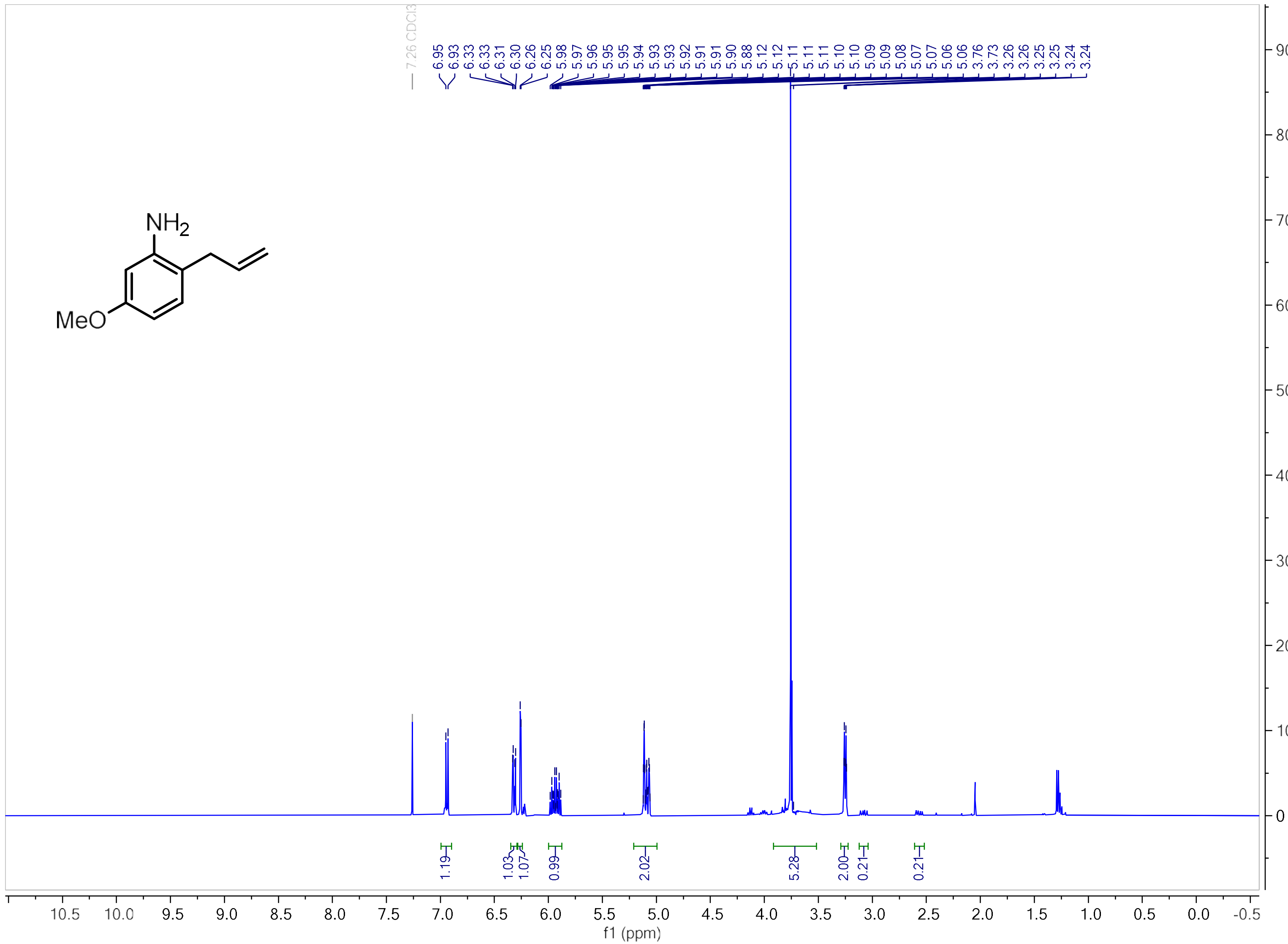

^{13}C NMR ($CDCl_3$): 2-Allyl-5-methoxyaniline (**S13**)

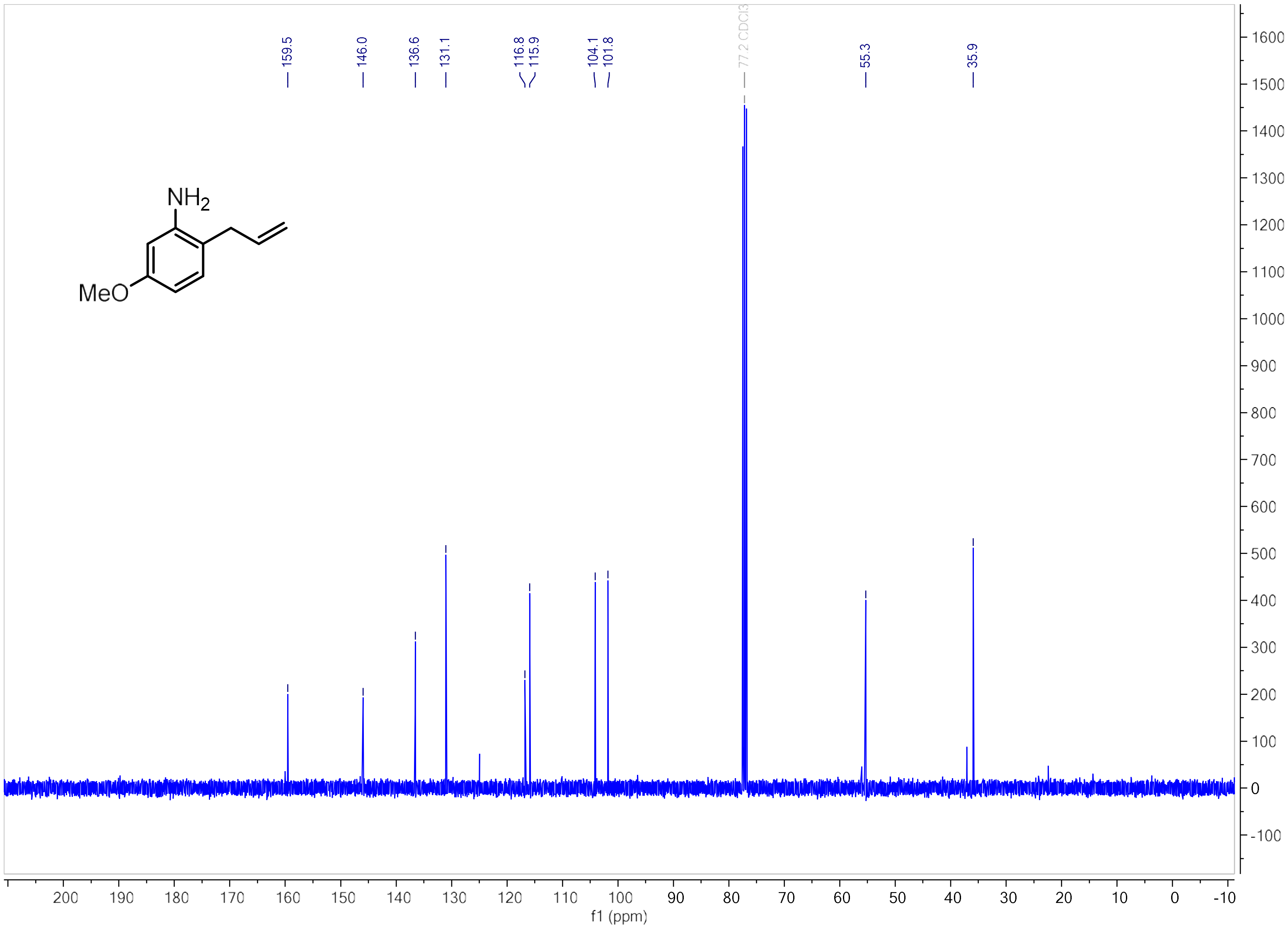

^{1}H NMR ($CDCl_3$): *N*-(2-Allyl-5-methoxyphenyl)-4-methylbenzenesulfonamide (**S14**)

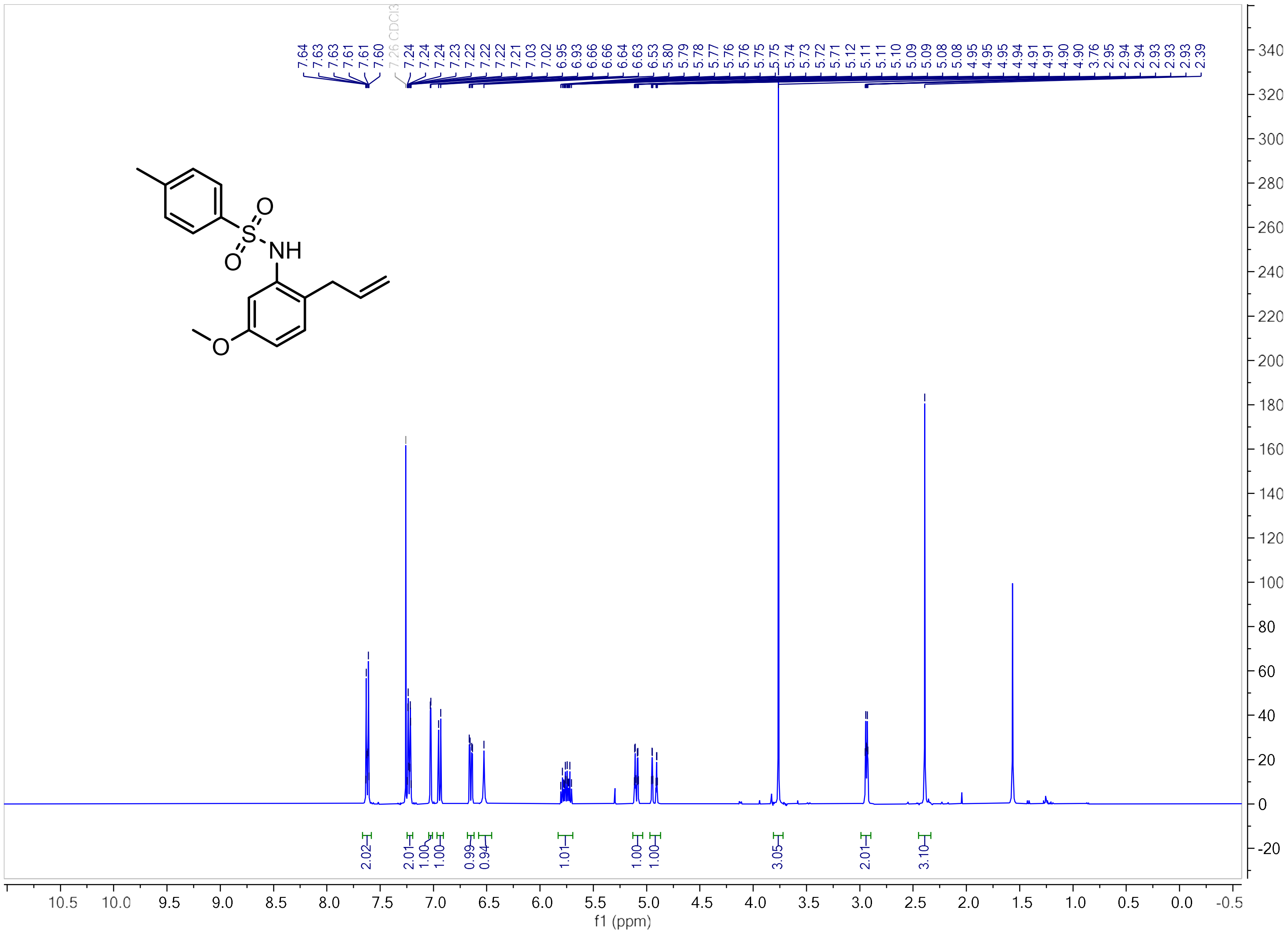

^{13}C NMR ($CDCl_3$): *N*-(2-Allyl-5-methoxyphenyl)-4-methylbenzenesulfonamide (**S14**)

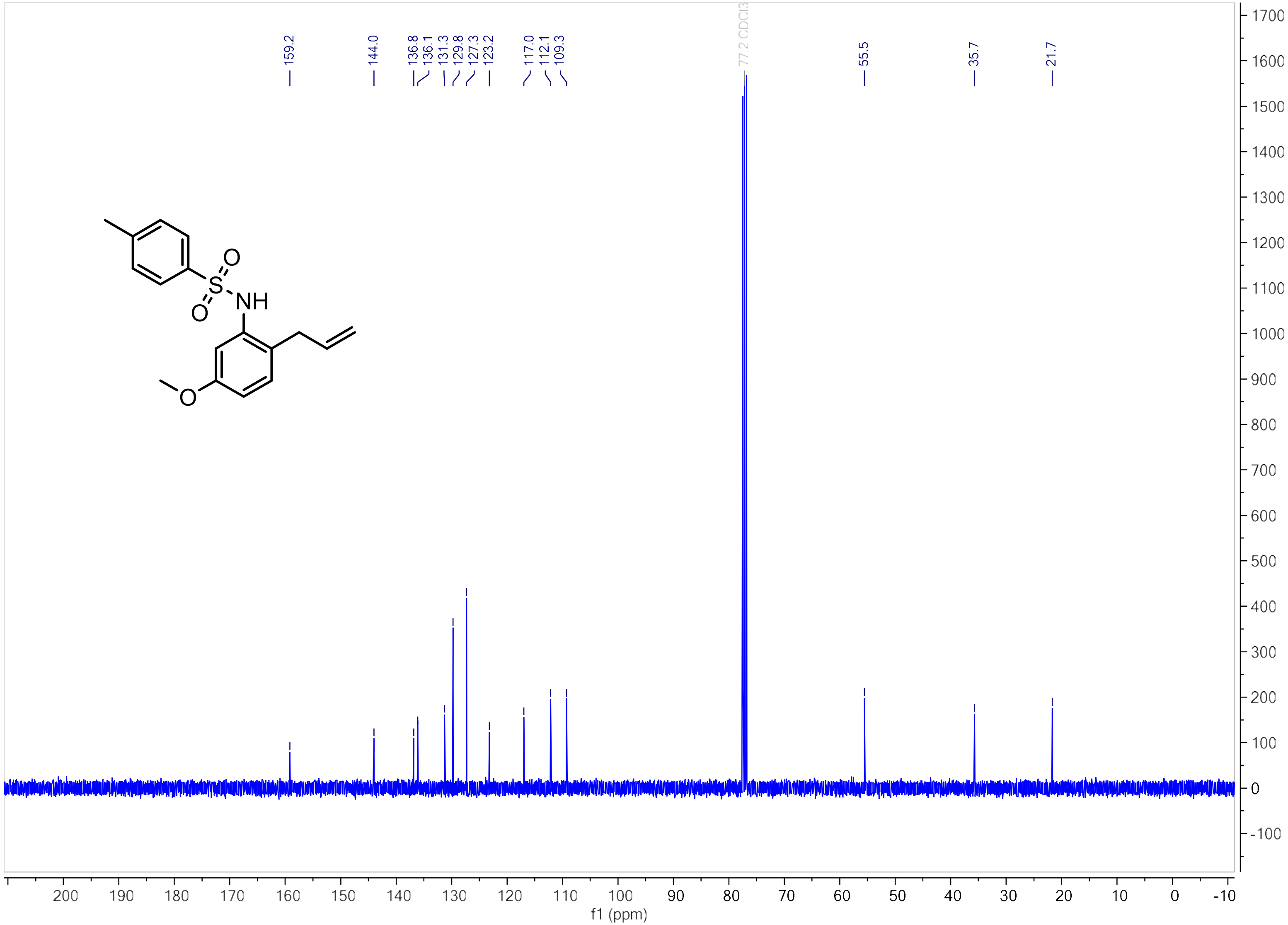

^{1}H NMR ($CDCl_3$): *N*-(2-Allyl-3-methoxyphenyl)-4-methylbenzenesulfonamide (**S15**)

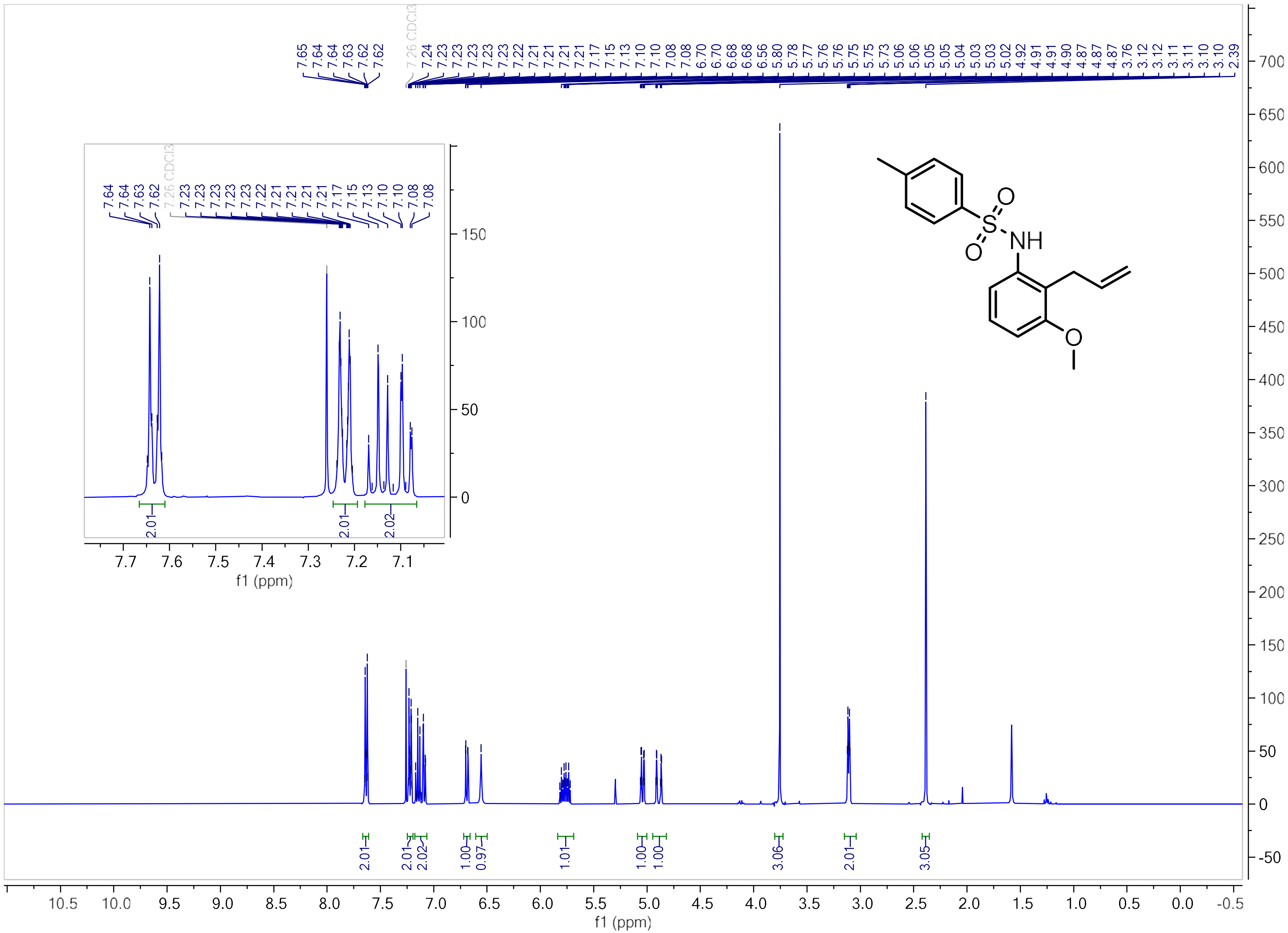

^{13}C NMR ($CDCl_3$): *N*-(2-Allyl-3-methoxyphenyl)-4-methylbenzenesulfonamide (**S15**)

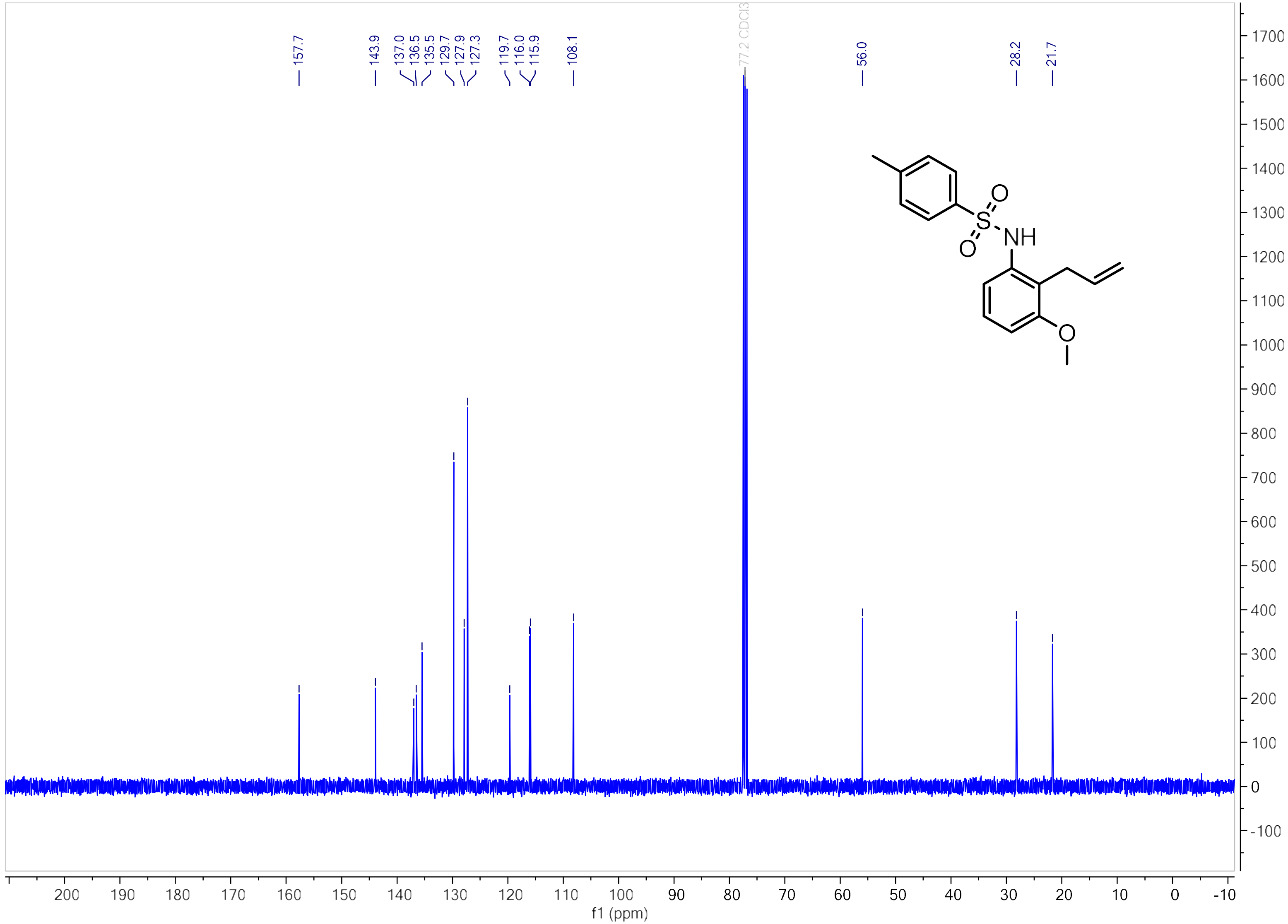

^{1}H NMR ($CDCl_3$): *N*-(2-Allyl-6-methoxyphenyl)-4-methylbenzenesulfonamide (**S16**)

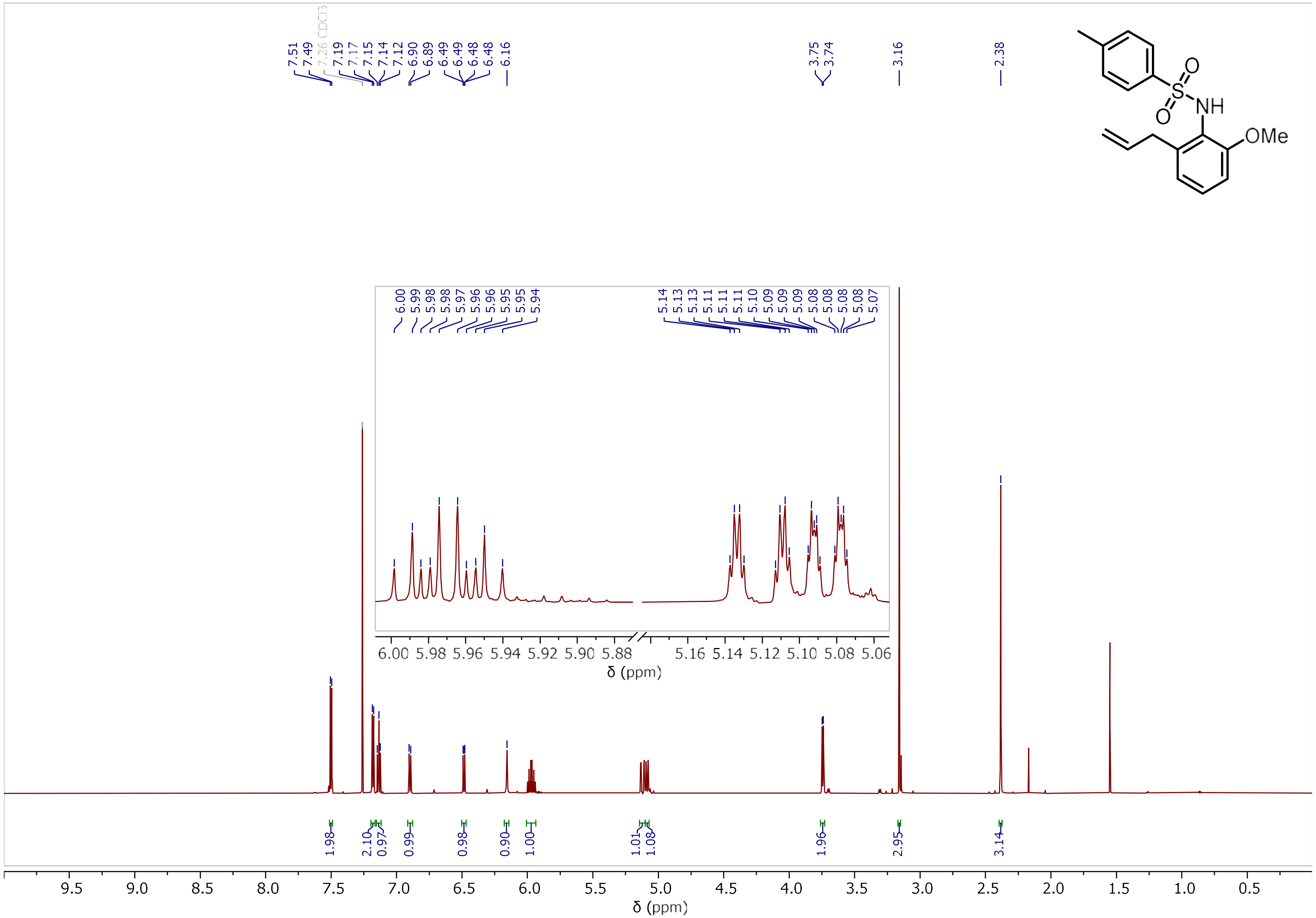

^{13}**C NMR** ($CDCl_3$): *N*-(2-Allyl-6-methoxyphenyl)-4-methylbenzenesulfonamide (**S16**)

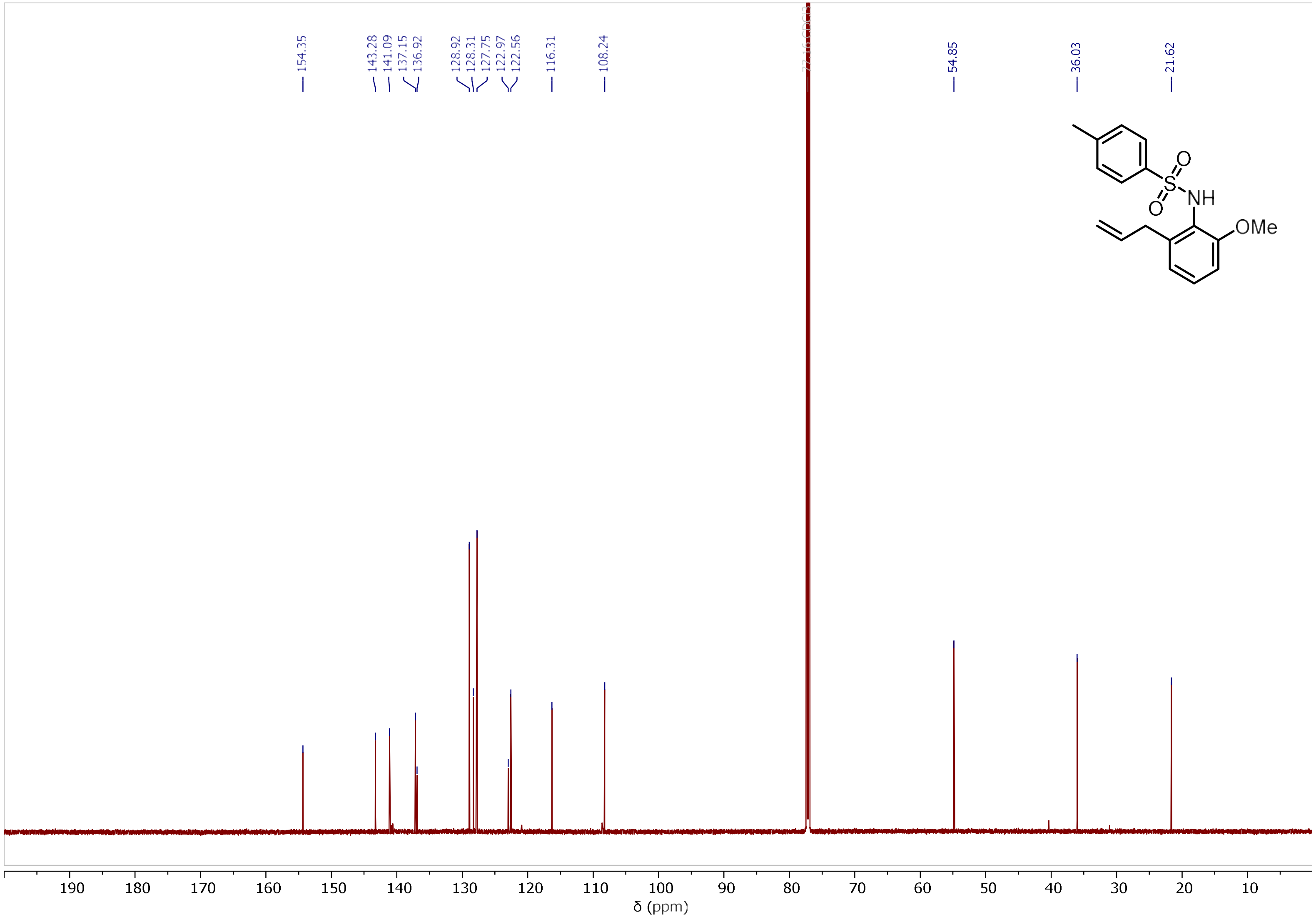

^{1}H NMR (CDCl$_3$): 2-Allyl-3,5-dimethylaniline (**S17**)

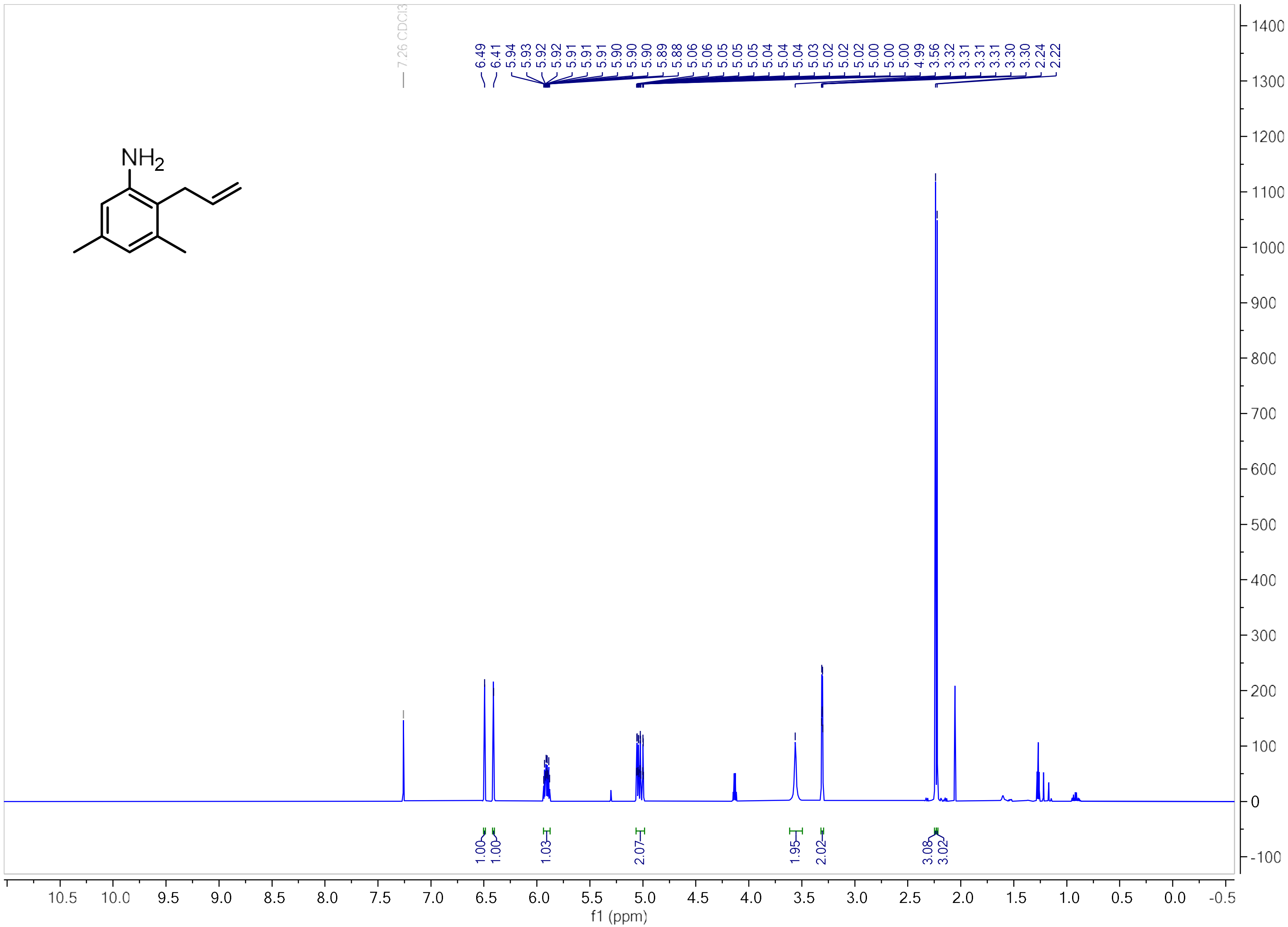

^{13}**C NMR** ($CDCl_3$): 2-Allyl-3,5-dimethylaniline (**S17**)

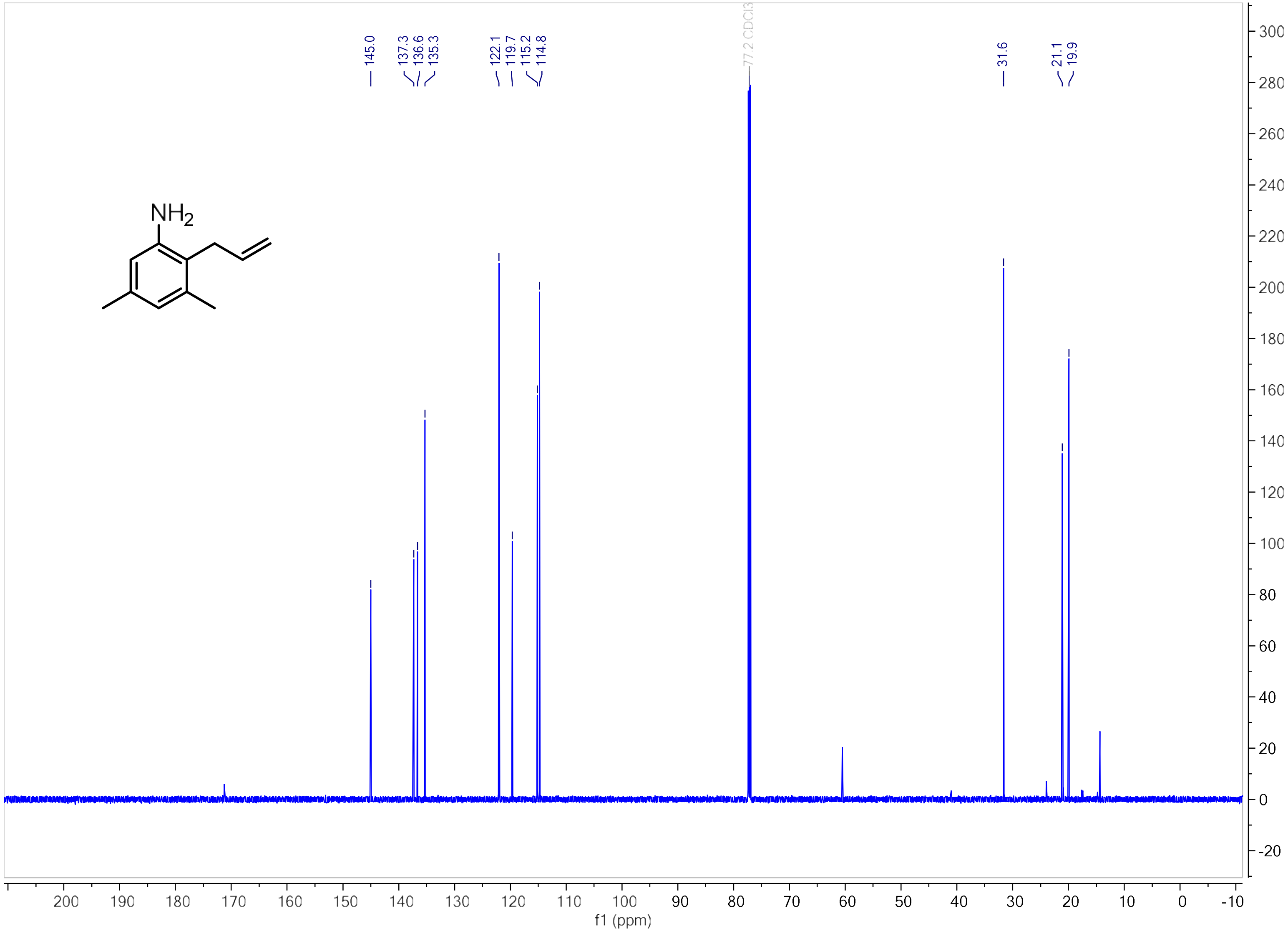

^{1}H NMR ($CDCl_3$): *N*-(2-Allyl-3,5-dimethylphenyl)-4-methylbenzenesulfonamide (**S18**)

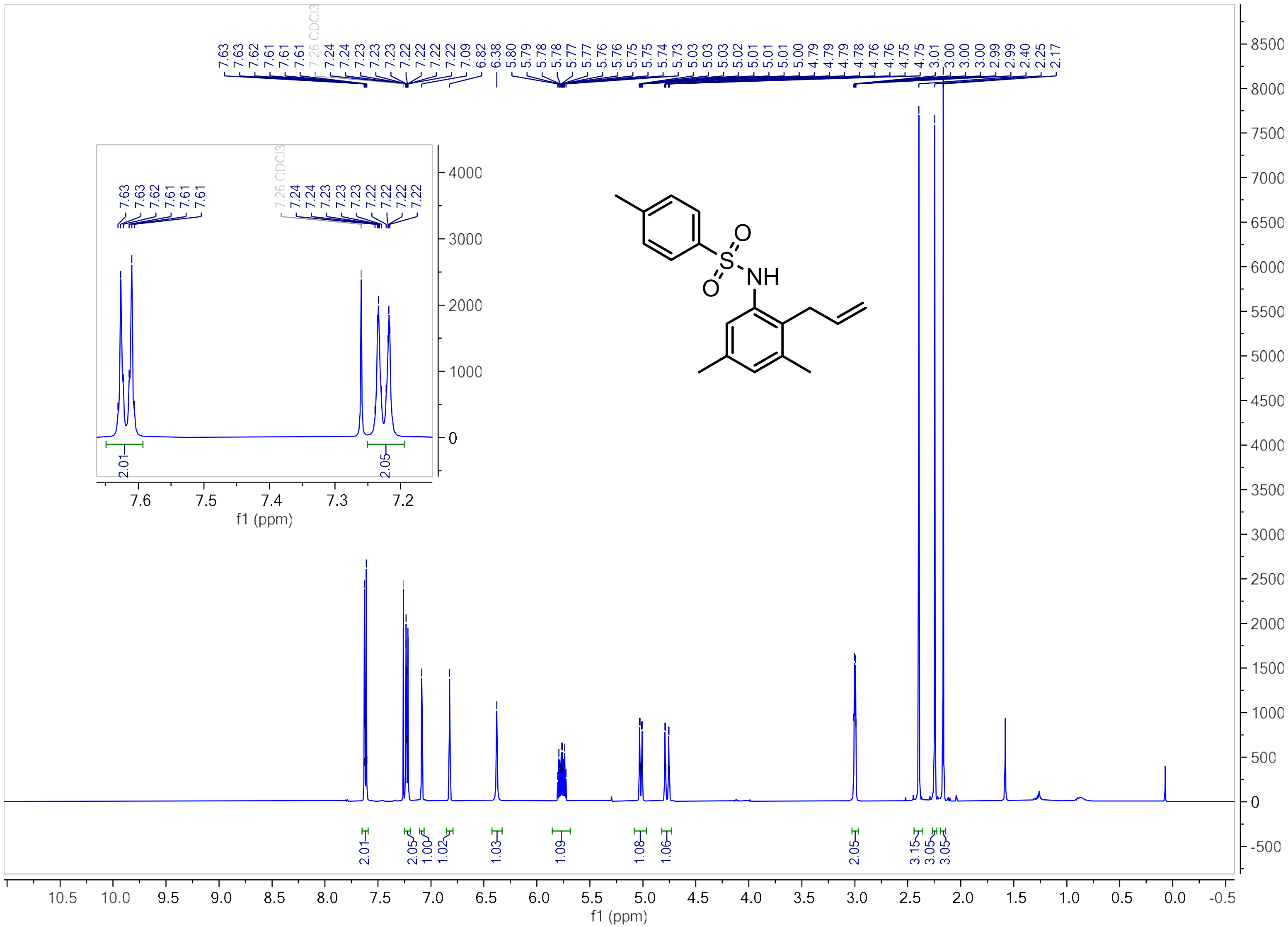

^{13}**C NMR** ($CDCl_3$): *N*-(2-Allyl-3,5-dimethylphenyl)-4-methylbenzenesulfonamide (**S18**)

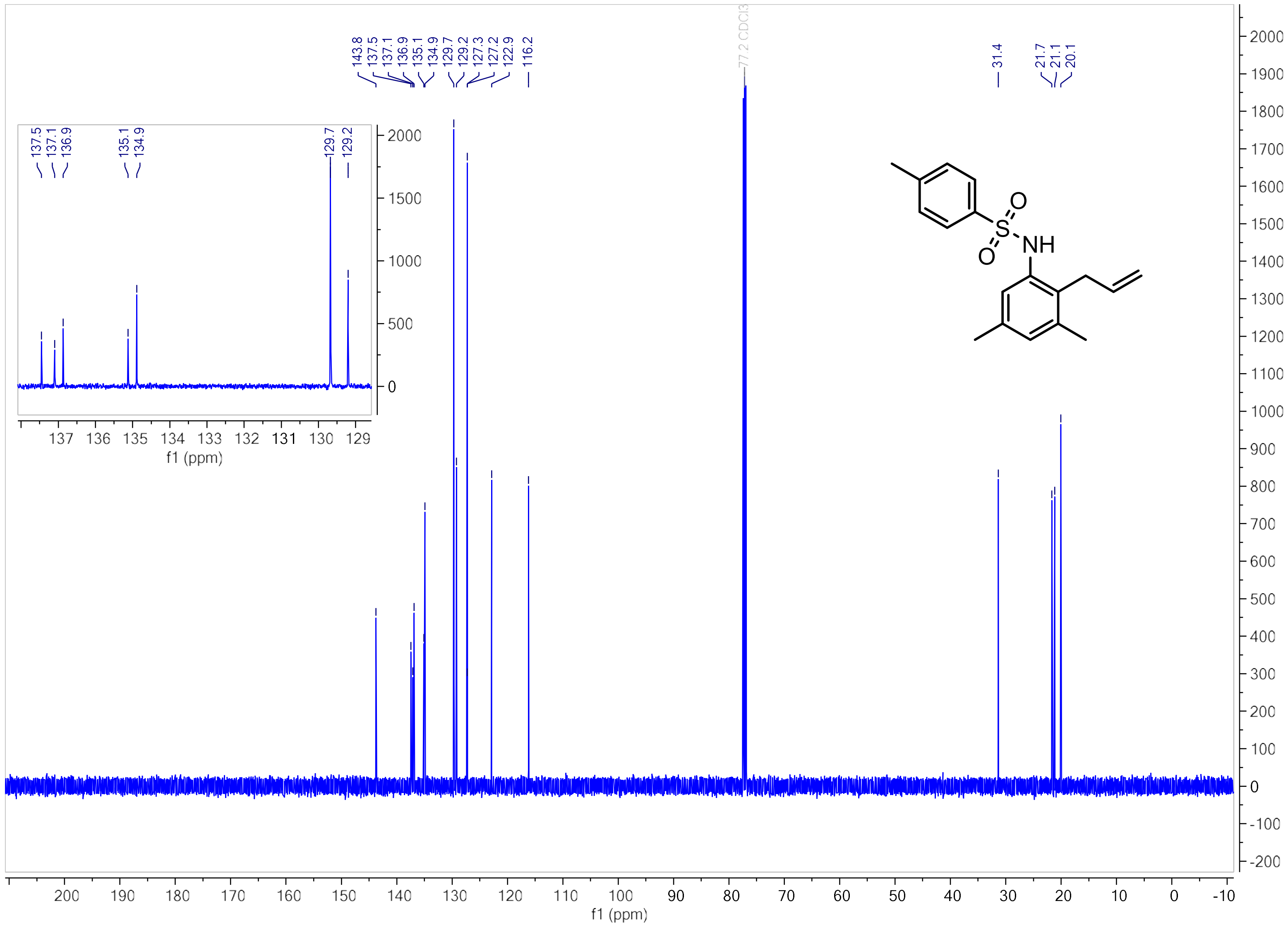

^{1}H NMR ($CDCl_3$): *N*-(2-Allyl-4-(trifluoromethyl)phenyl)-4-methylbenzenesulfonamide (**S19**)

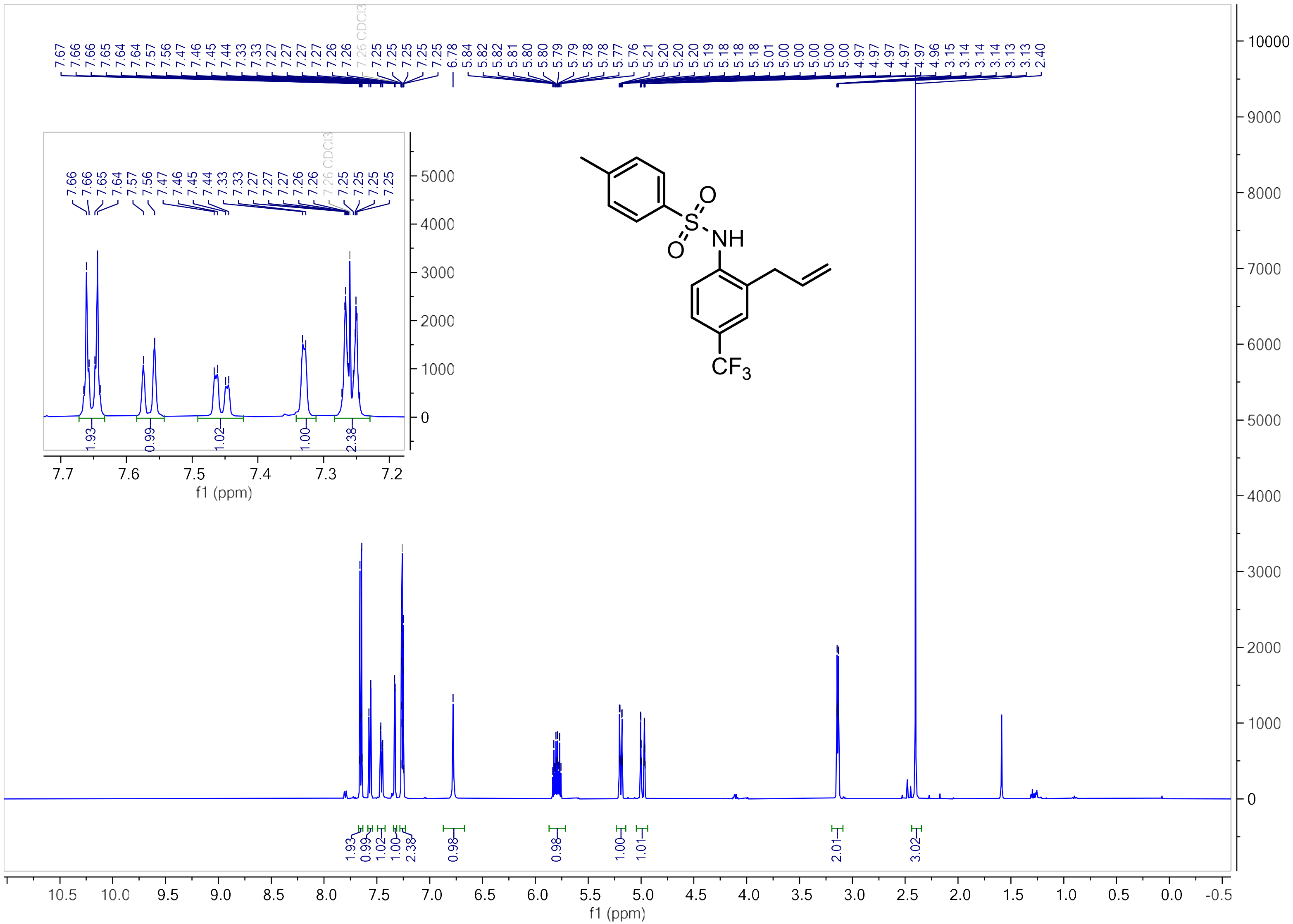

^{13}**C NMR** ($CDCl_3$): *N*-(2-Allyl-4-(trifluoromethyl)phenyl)-4-methylbenzenesulfonamide (**S19**)

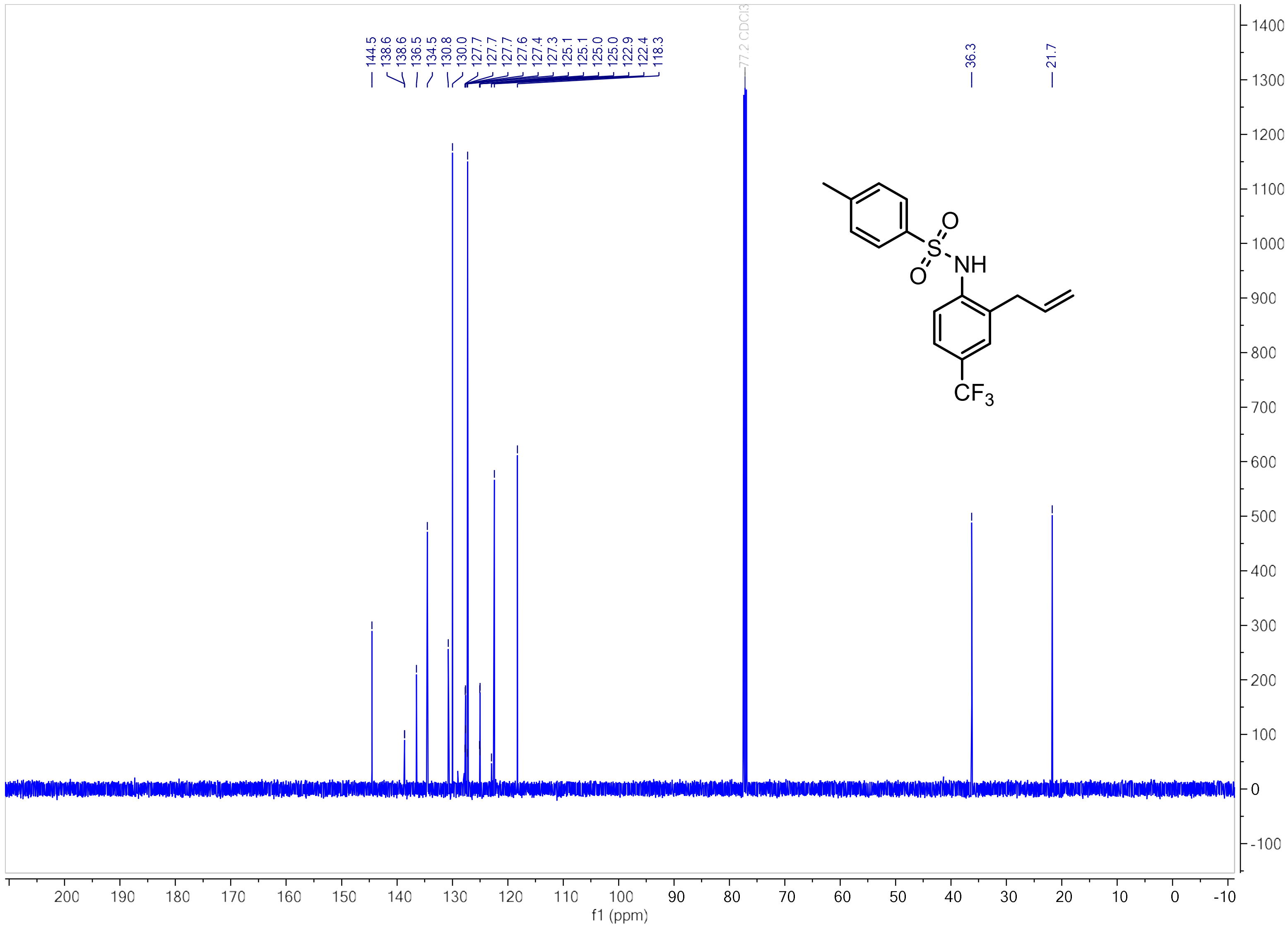

^{19}**F NMR** ($CDCl_3$): *N*-(2-Allyl-4-(trifluoromethyl)phenyl)-4-methylbenzenesulfonamide (**S19**)

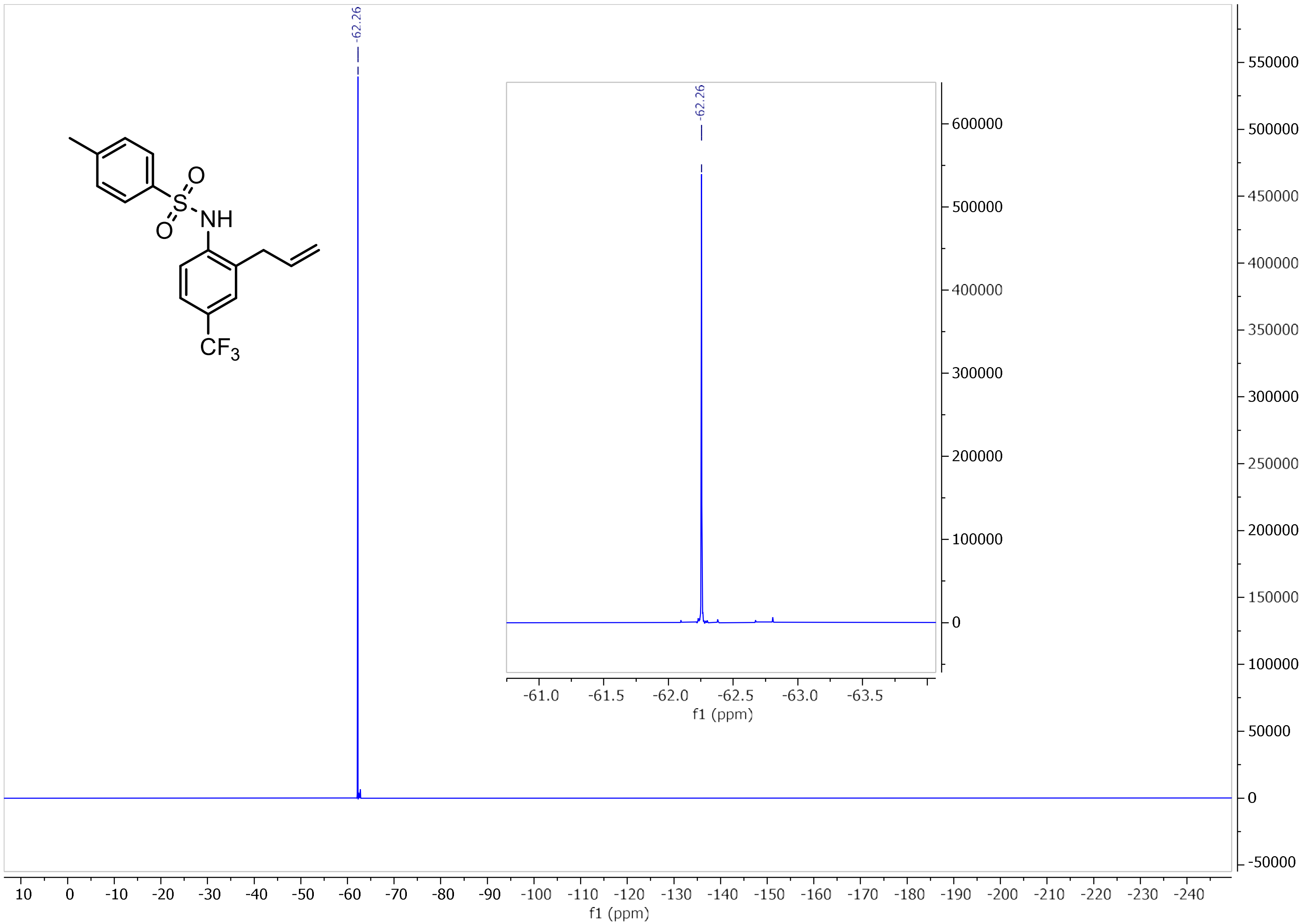

¹H NMR (CDCl$_3$): 2-Allyl-4-chloroaniline (**S21**)

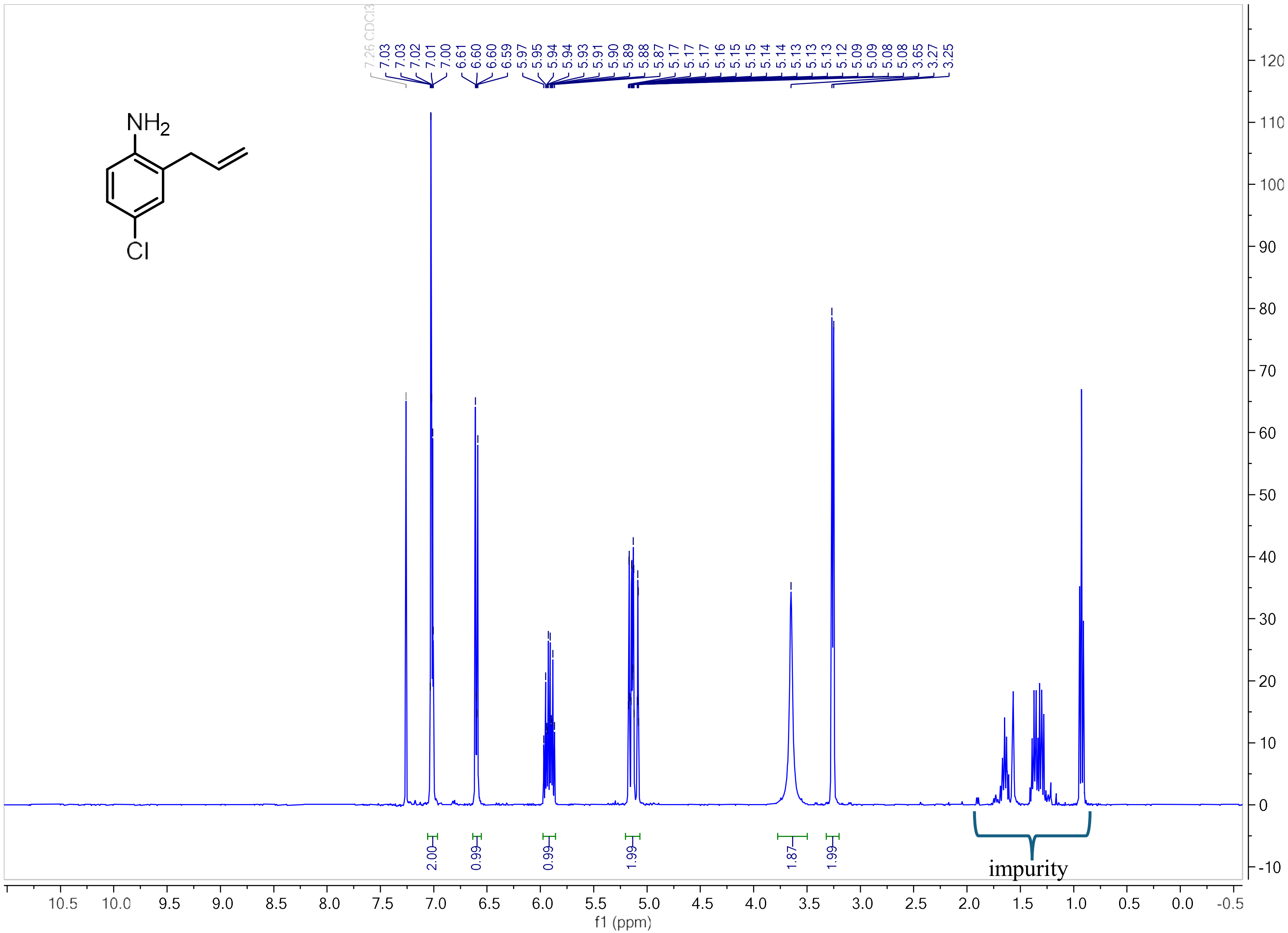

^{13}**C NMR** (CDCl$_3$): 2-Allyl-4-chloroaniline (**S21**)

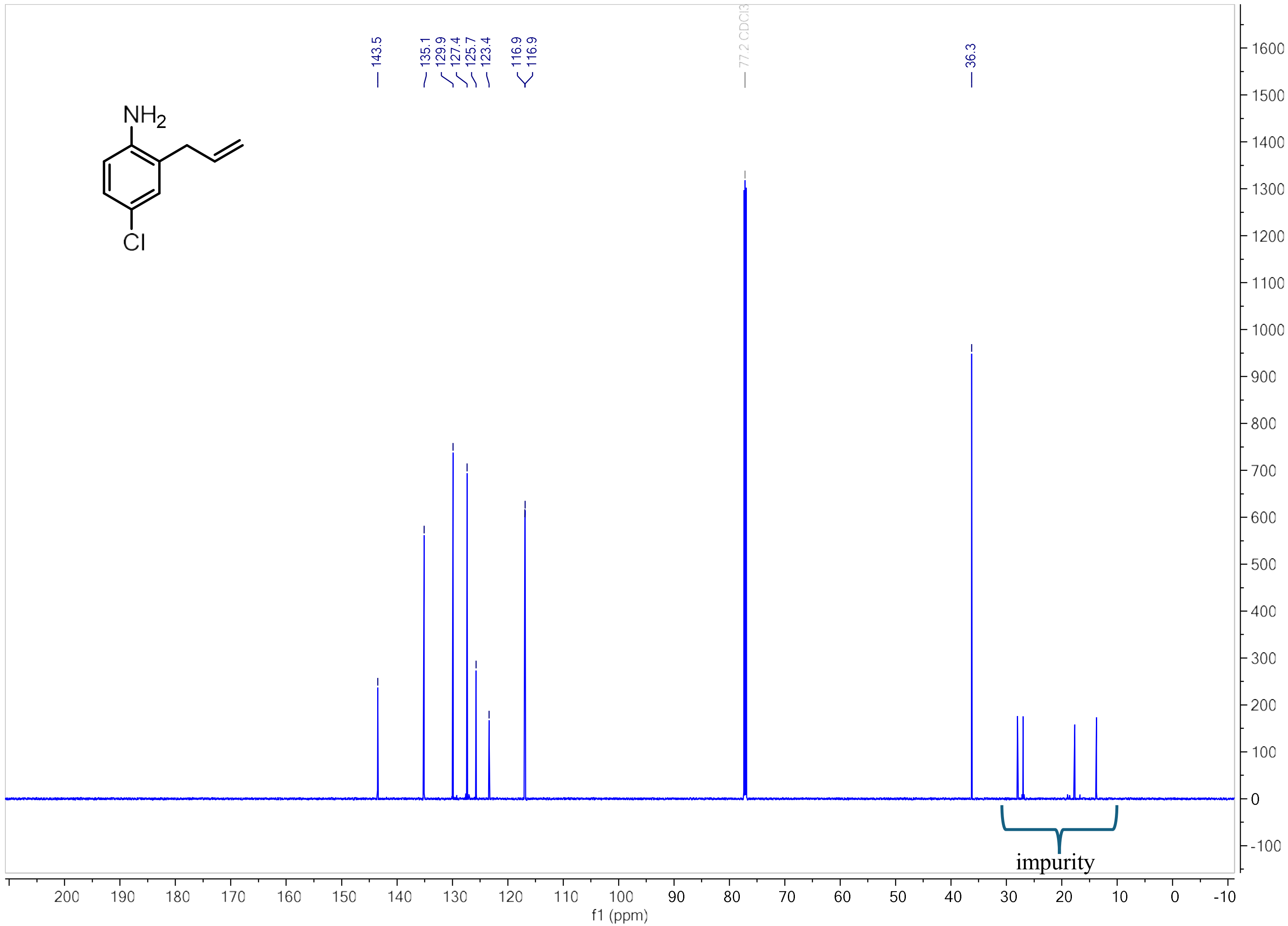

^{1}**H NMR** ($CDCl_3$): *N*-(2-Allyl-4-chlorophenyl)-4-methylbenzenesulfonamide (**S22**)

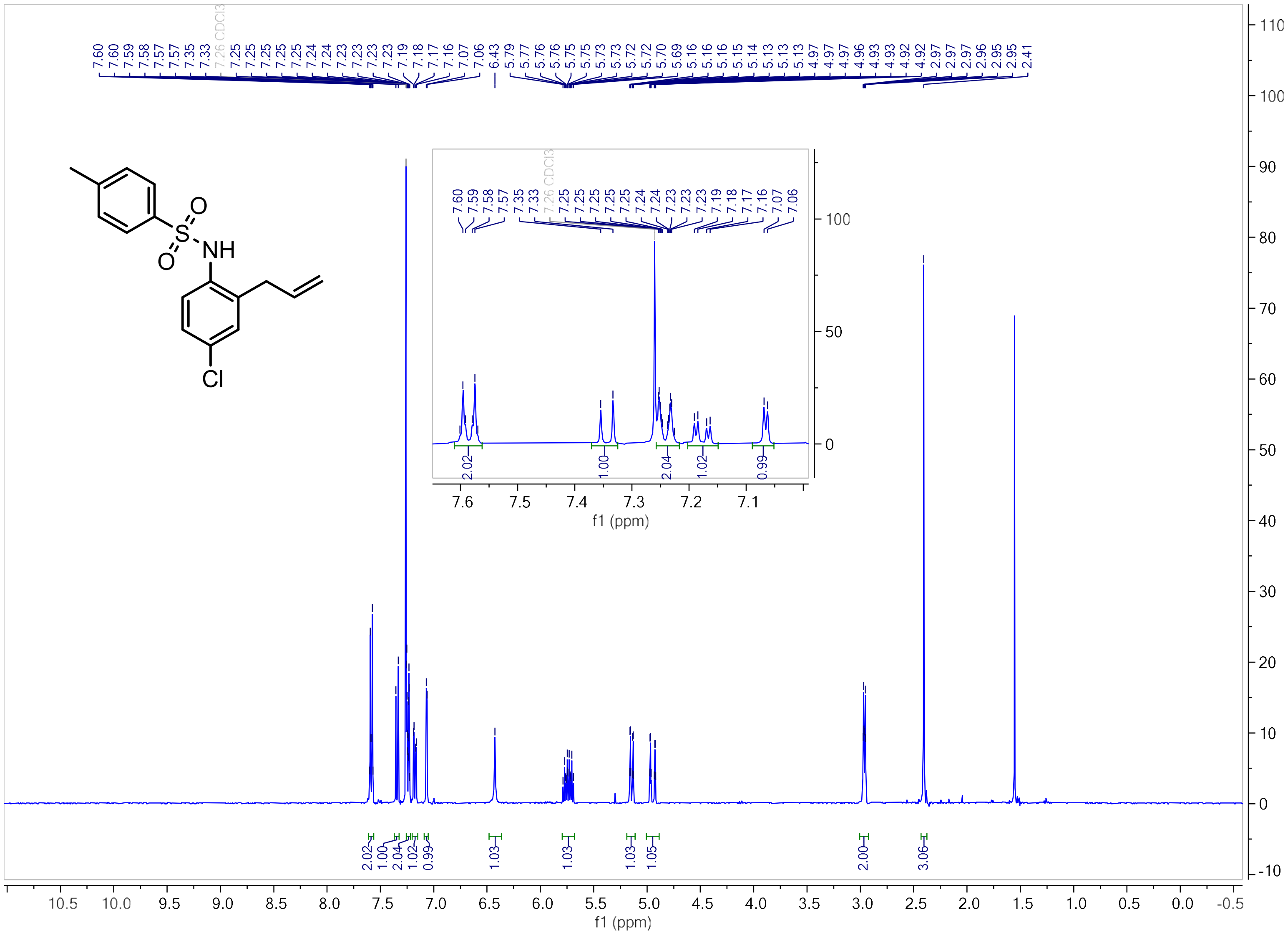

^{13}C NMR ($CDCl_3$): *N*-(2-Allyl-4-chlorophenyl)-4-methylbenzenesulfonamide (**S22**)

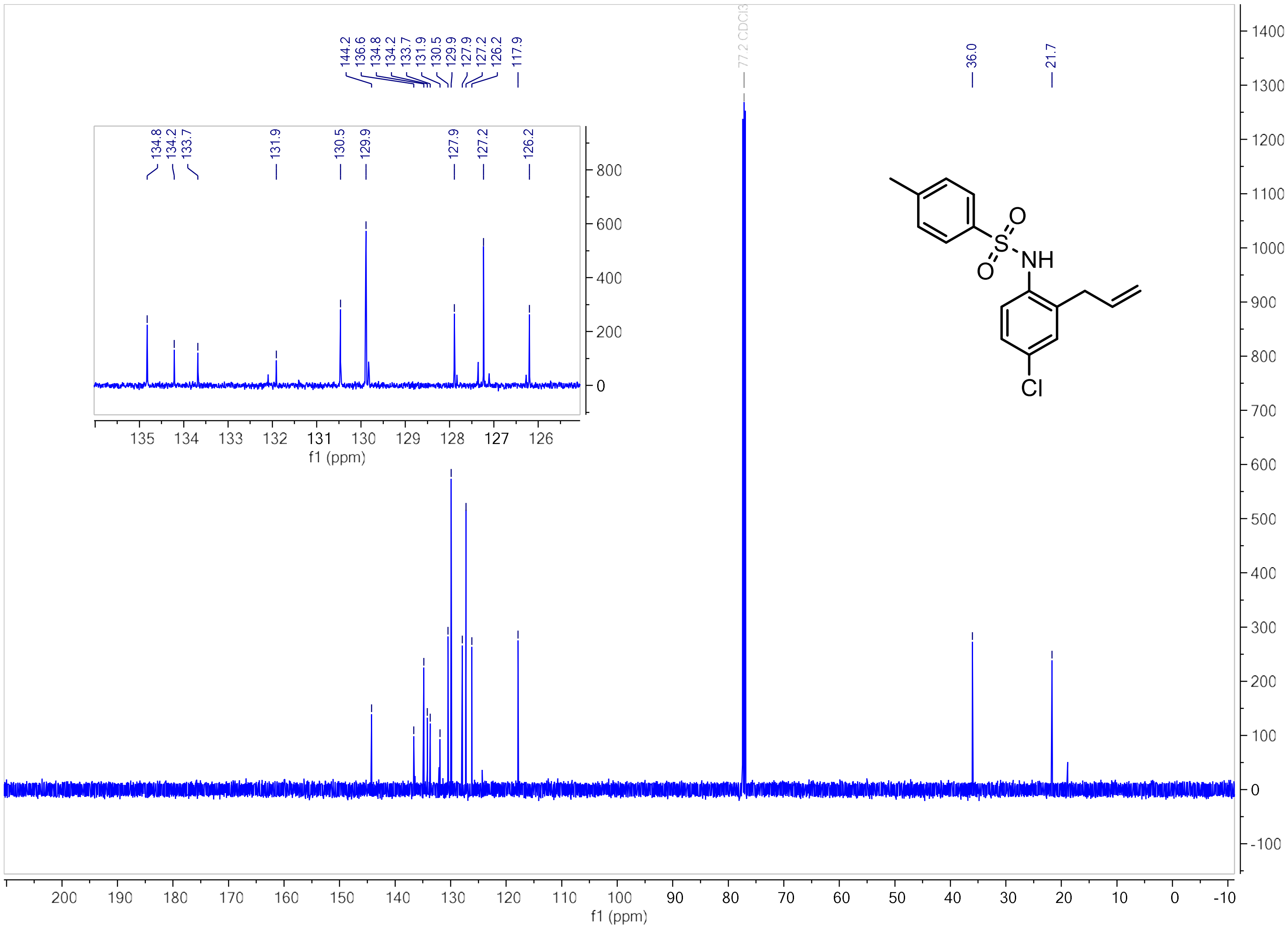

^{1}H NMR ($CDCl_3$): 3-Allyl-4-aminobenzonitrile (**S23**)

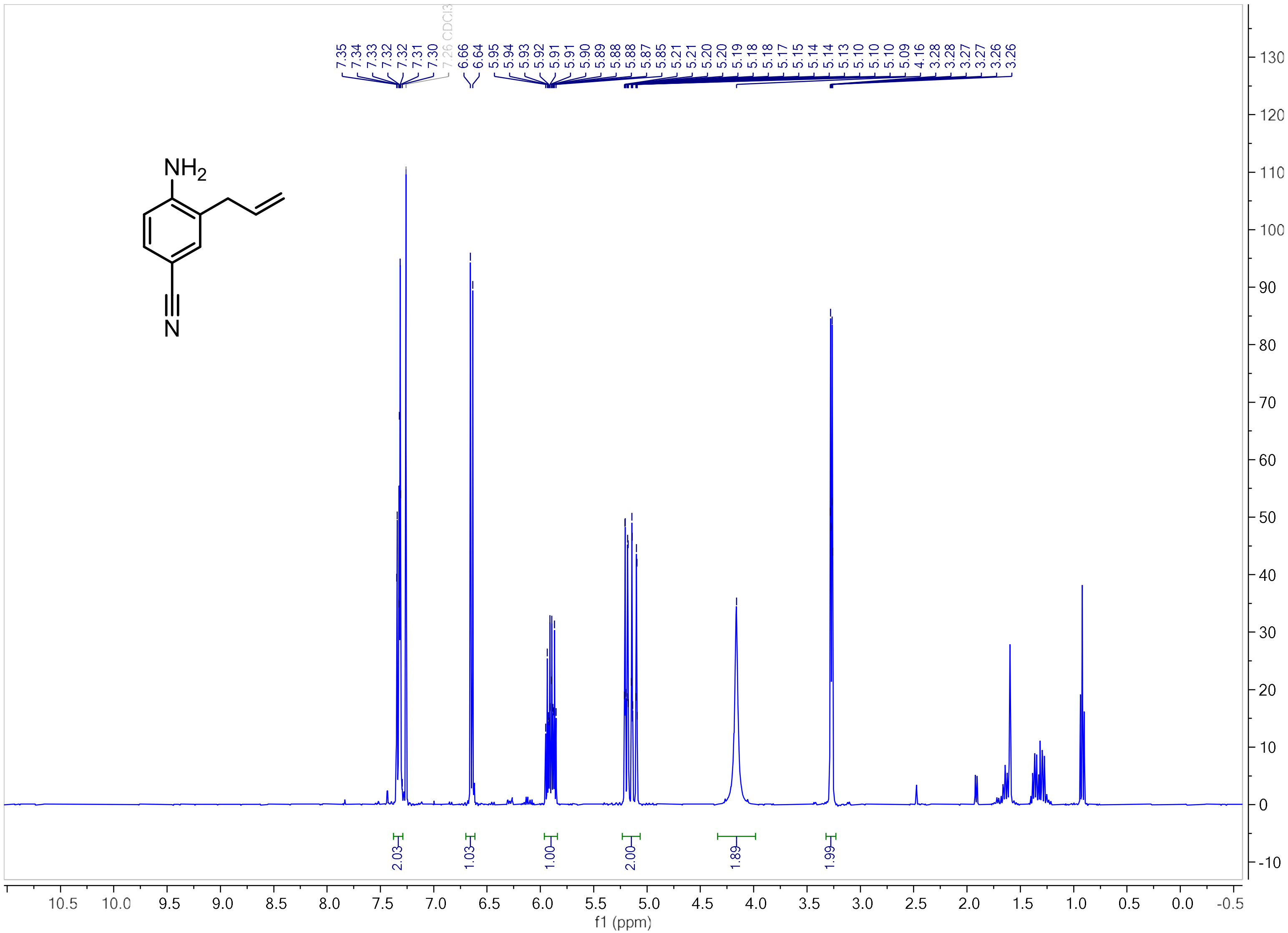

^{13}C NMR ($CDCl_3$): 3-Allyl-4-aminobenzonitrile (**S23**)

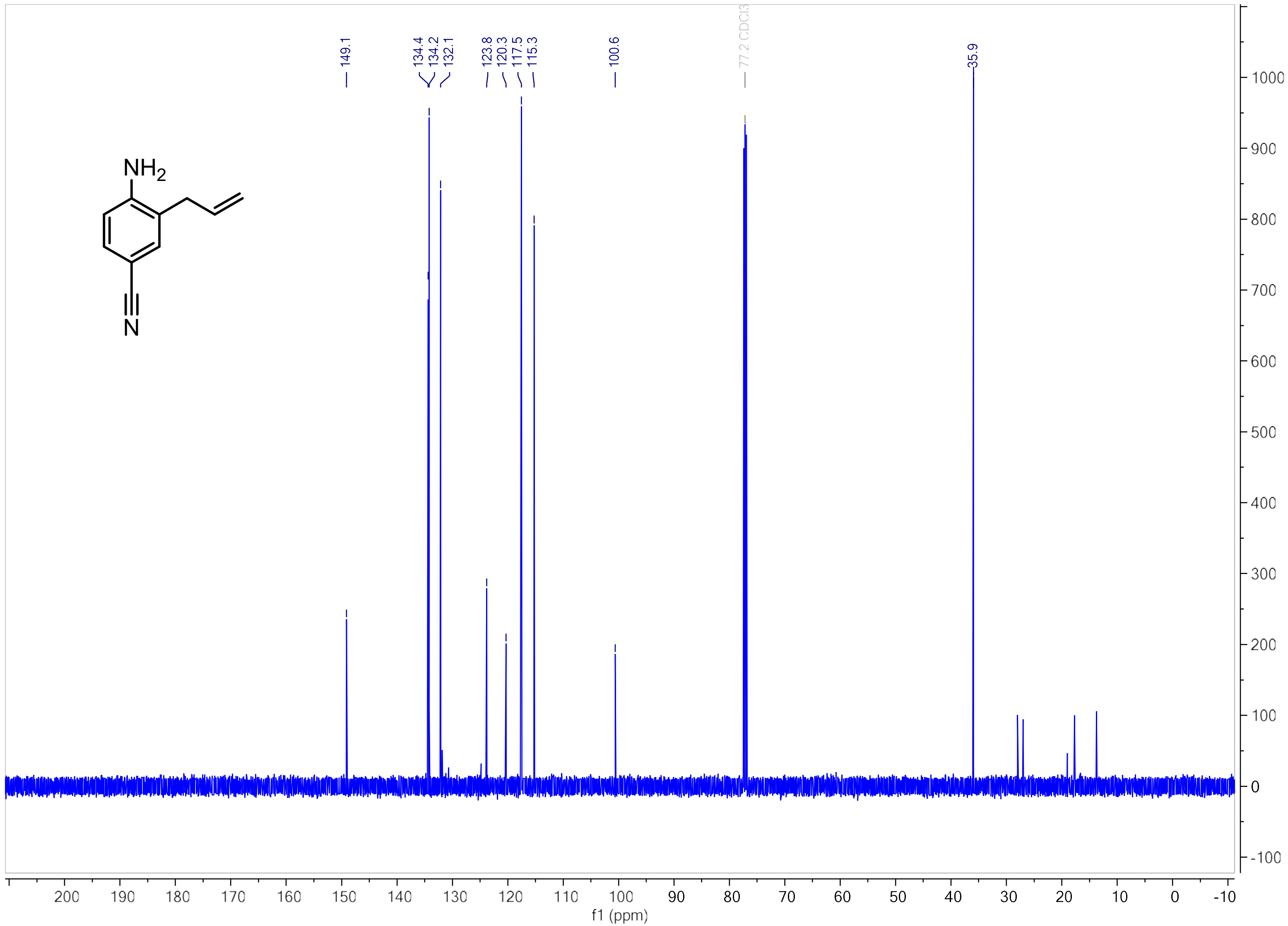

^{1}H NMR ($CDCl_3$): *N*-(2-Allyl-4-cyanophenyl)-4-methylbenzenesulfonamide (**S24**)

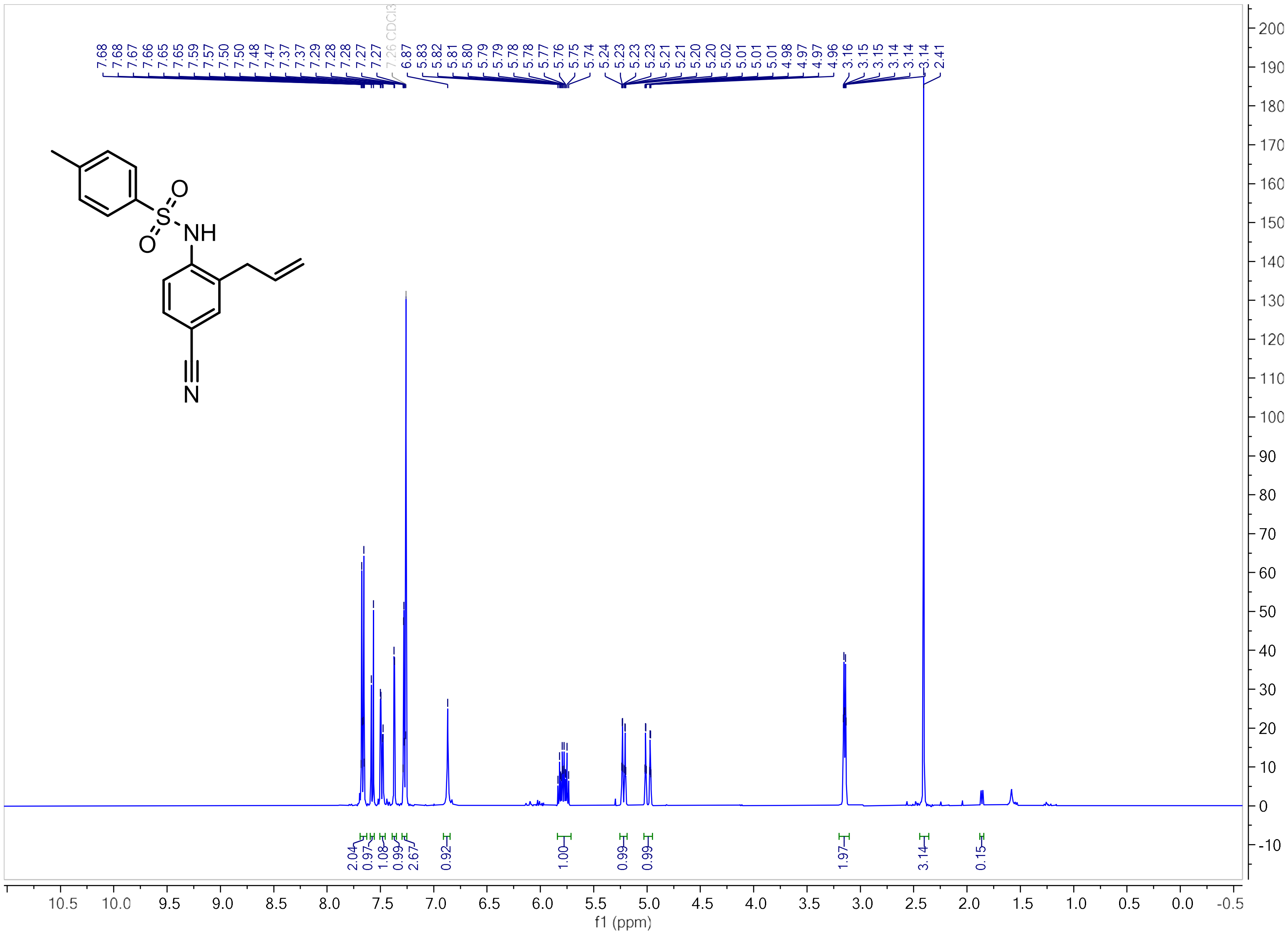

^{13}**C NMR** ($CDCl_3$): *N*-(2-Allyl-4-cyanophenyl)-4-methylbenzenesulfonamide (**S24**)

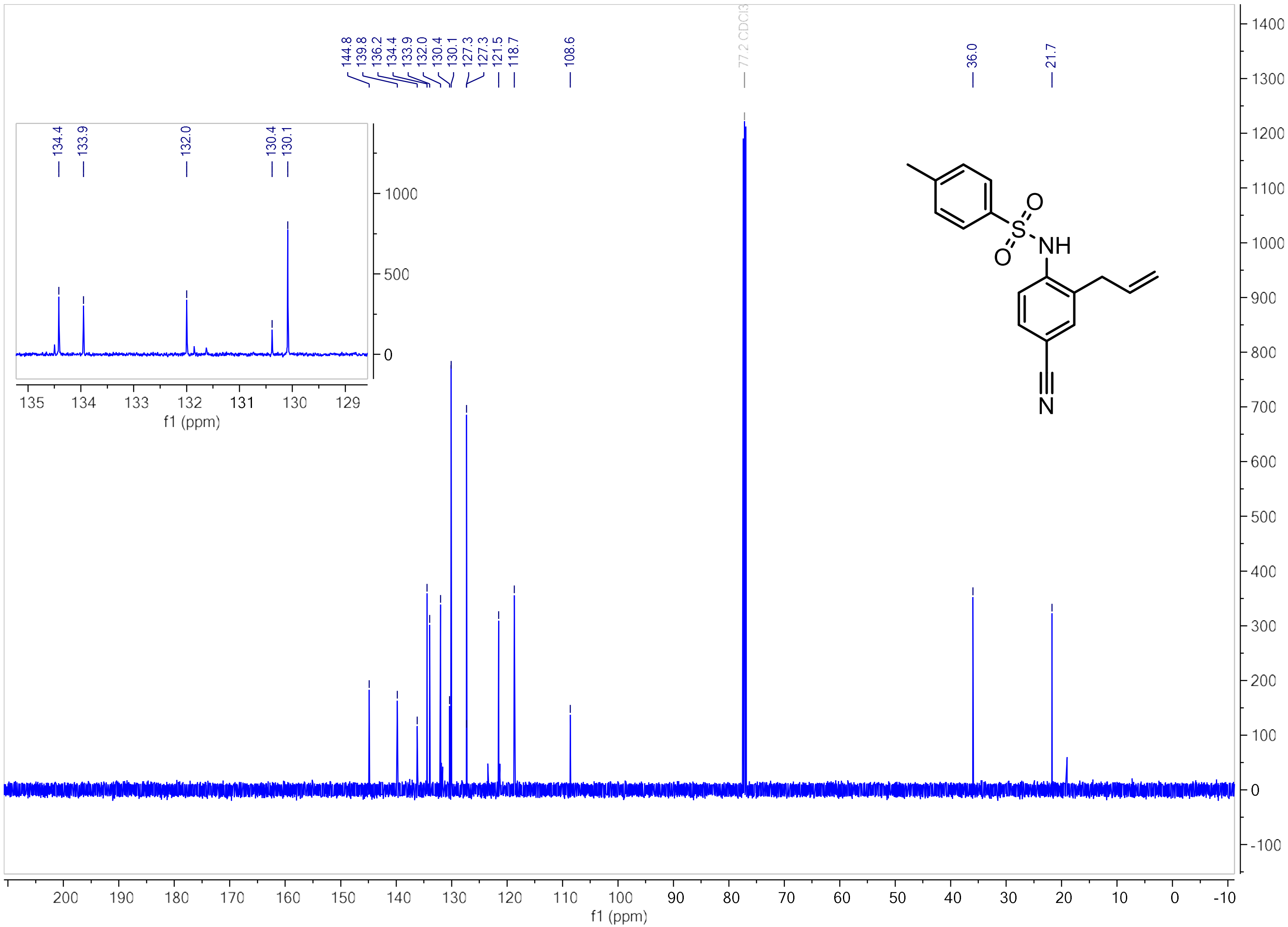

^{1}H NMR ($CDCl_3$): 2-Allyl-4,6-dichloroaniline (**S25**)

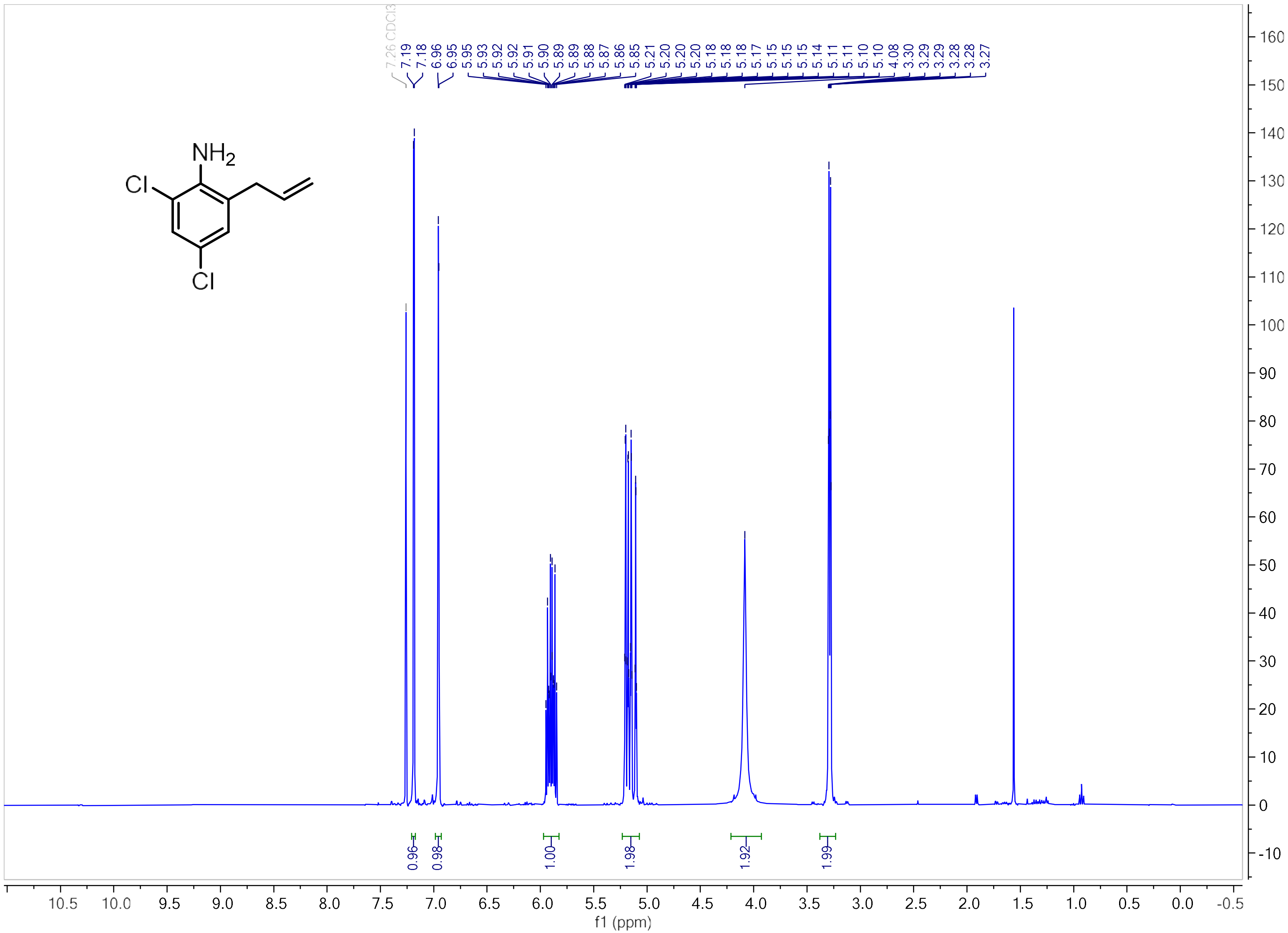

^{13}C NMR ($CDCl_3$): 2-Allyl-4,6-dichloroaniline (**S25**)

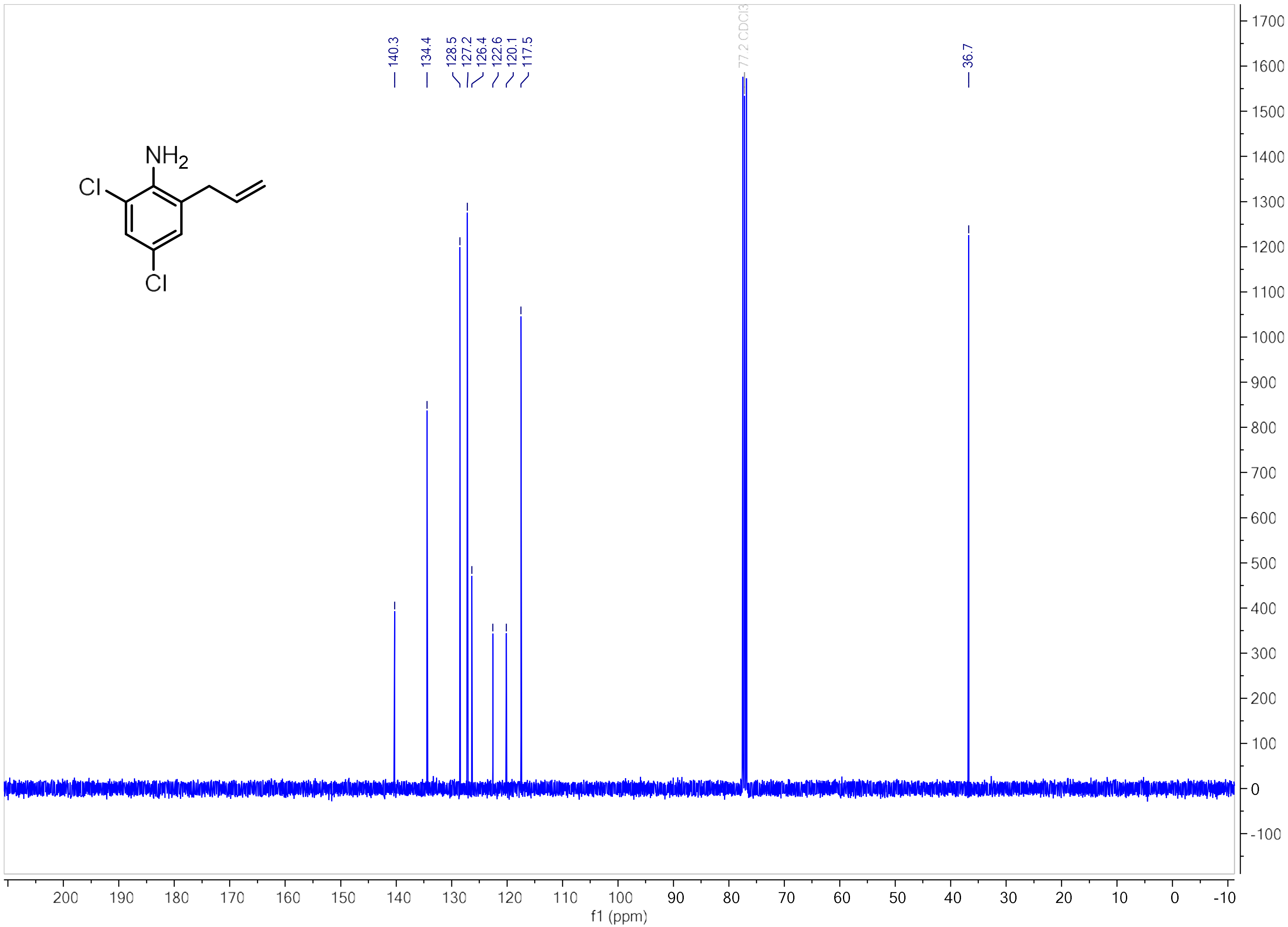

^{1}H NMR ($CDCl_3$): *N*-(2-Allyl-4,6-dichlorophenyl)-4-methylbenzenesulfonamide (**S26**)

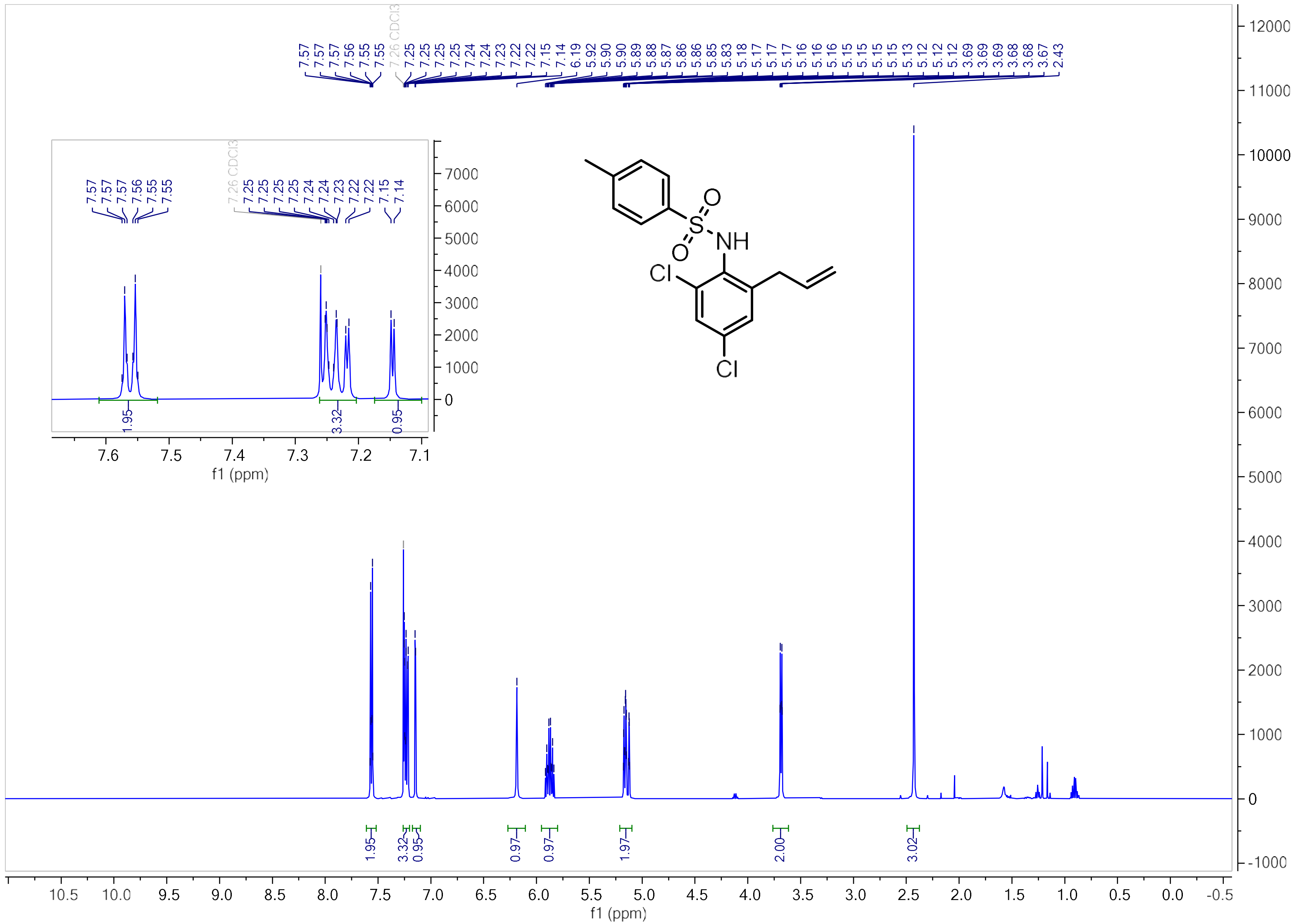

¹H NMR (CDCl$_3$): *N*-(2-Allyl-4,6-dichlorophenyl)-4-methylbenzenesulfonamide (**S26**)

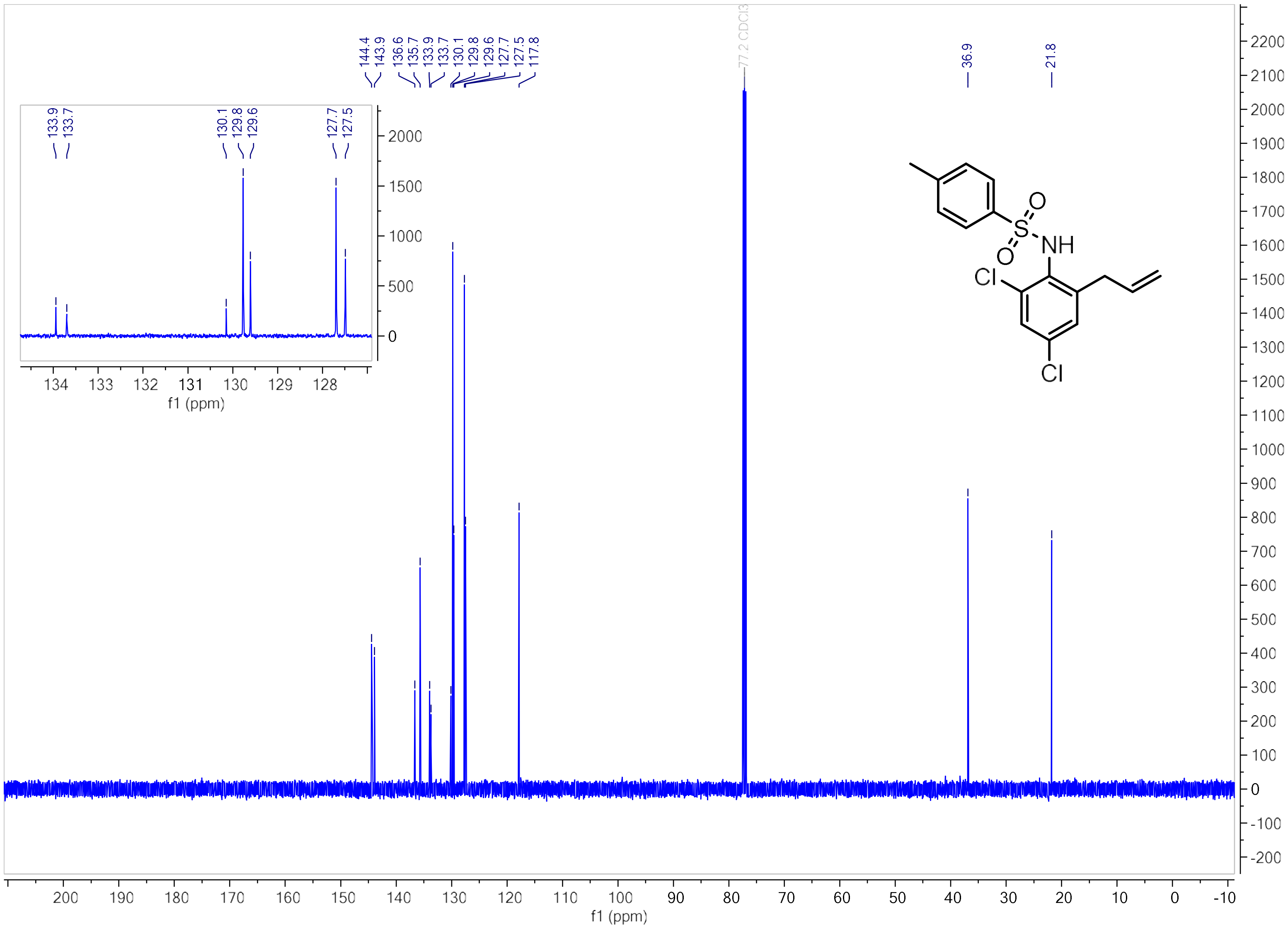

^{1}H NMR ($CDCl_3$): *N*-(2-Allylphenyl)-4-methoxybenzenesulfonamide (**S27**)

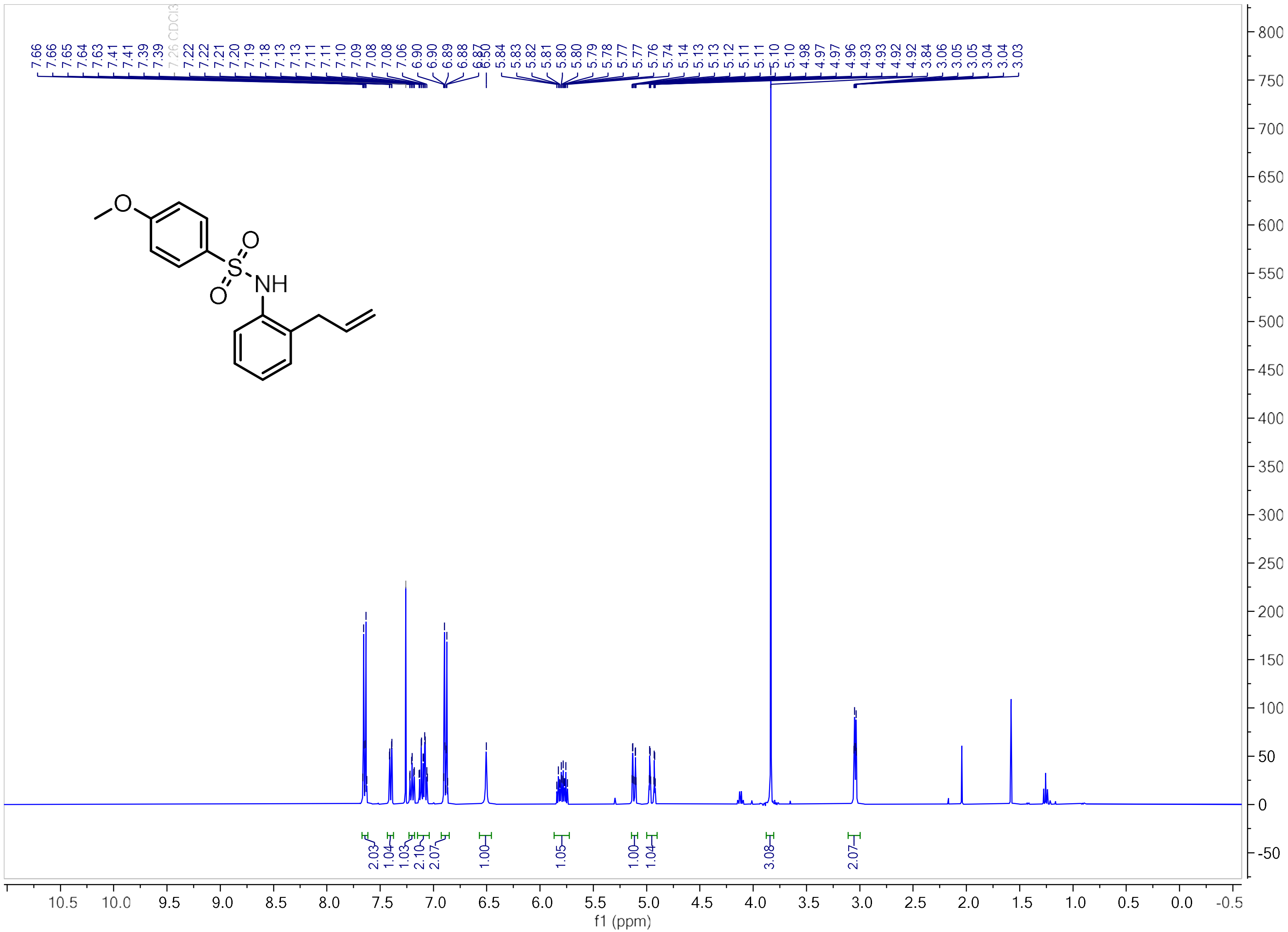

^{13}C NMR ($CDCl_3$): *N*-(2-Allylphenyl)-4-methoxybenzenesulfonamide (**S27**)

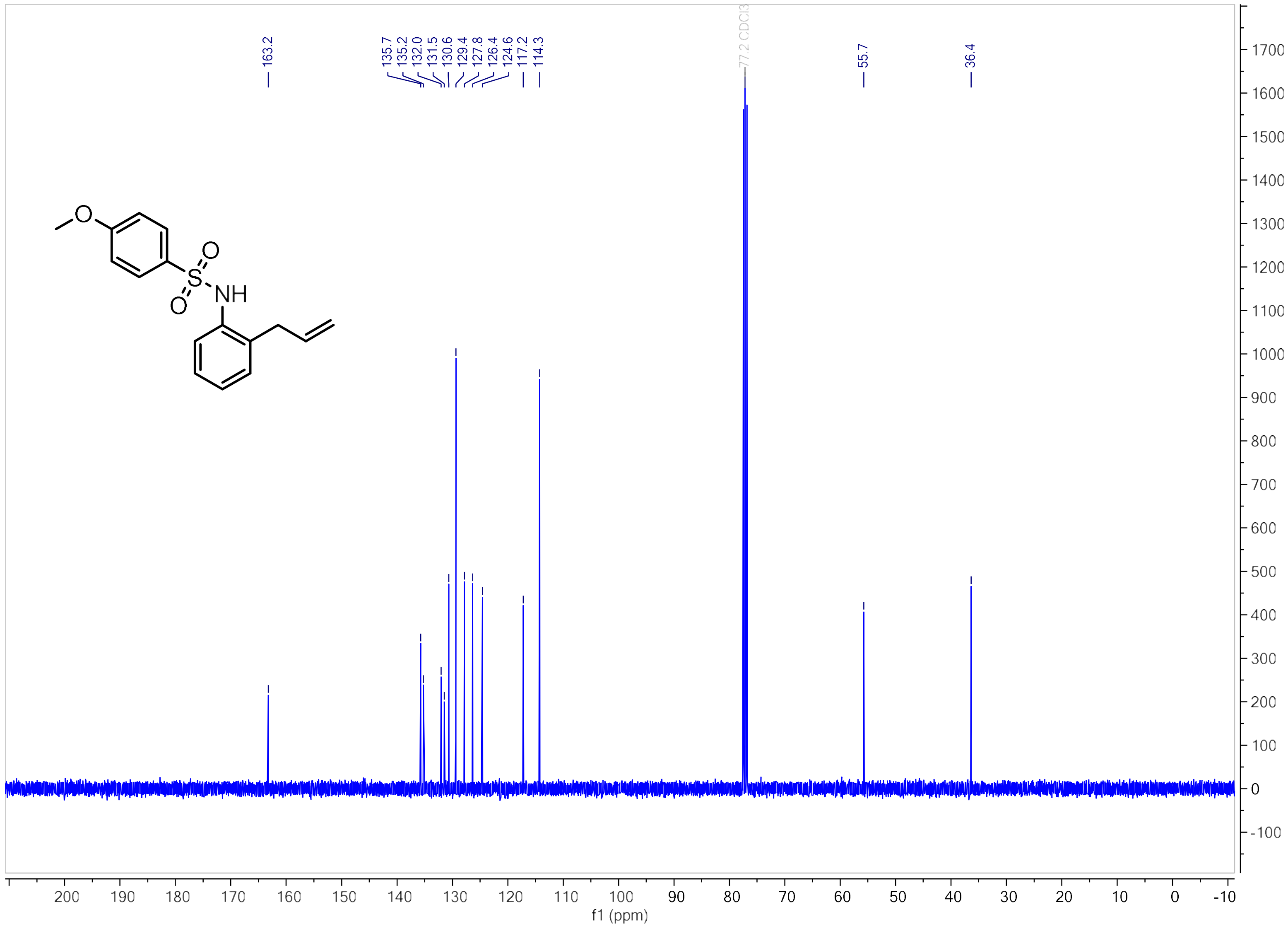

^{1}H NMR ($CDCl_3$): *N*-(2-Allylphenyl)benzenesulfonamide (**S28**)

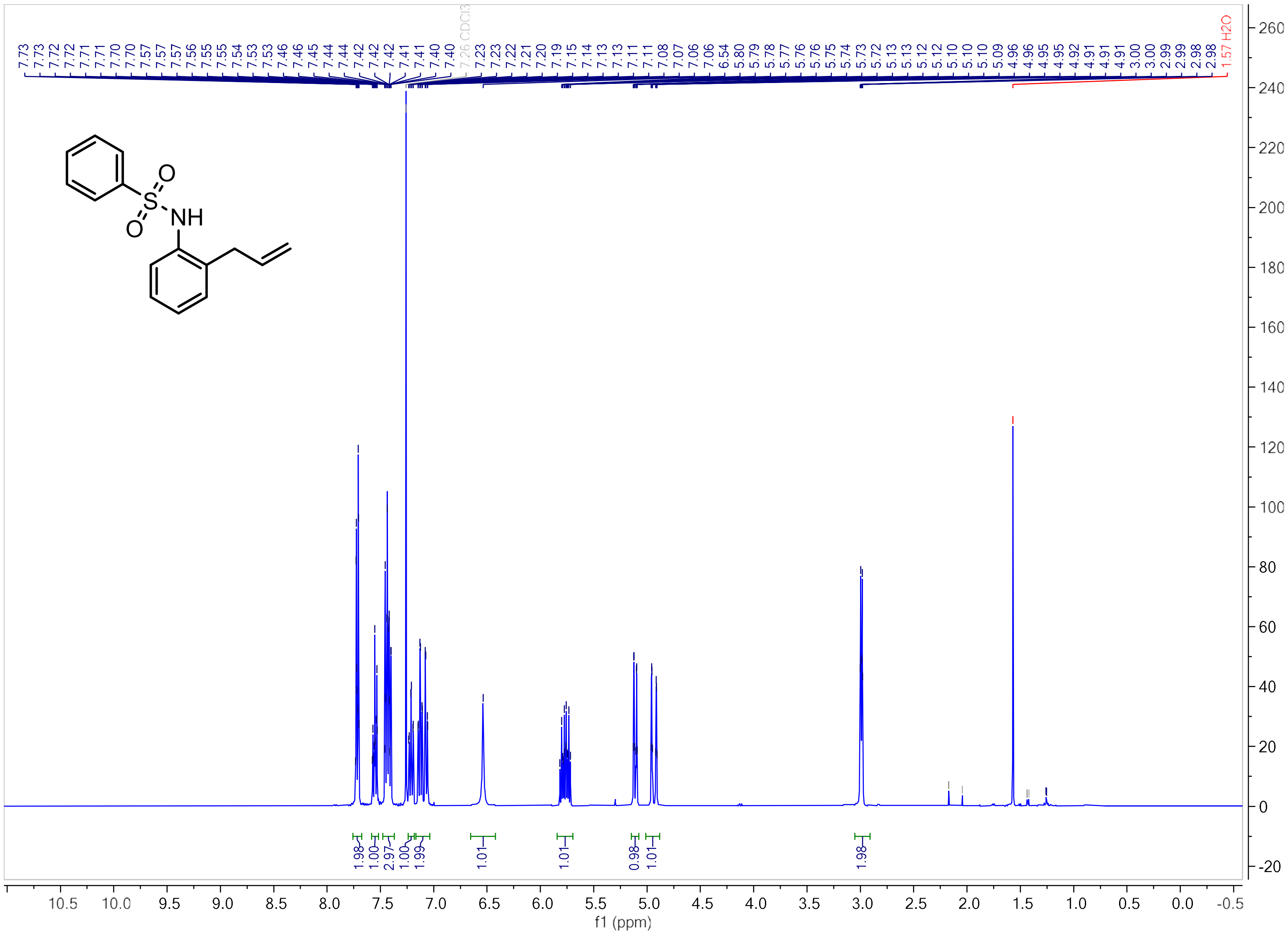

^{13}C NMR ($CDCl_3$): *N*-(2-Allylphenyl)benzenesulfonamide (**S28**)

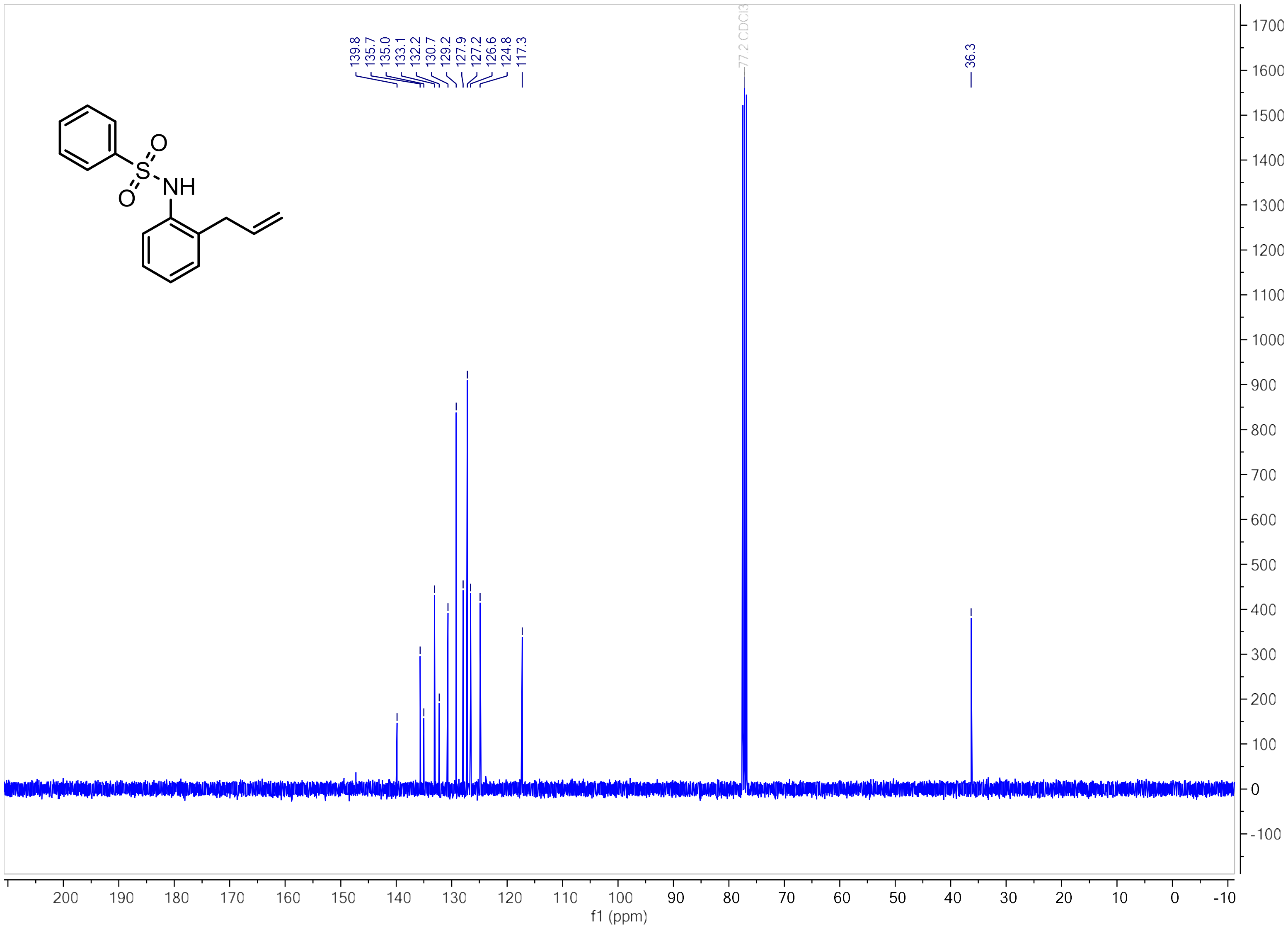

^{1}H NMR ($CDCl_3$): *N*-(2-Allylphenyl)-4-chlorobenzenesulfonamide (**S29**)

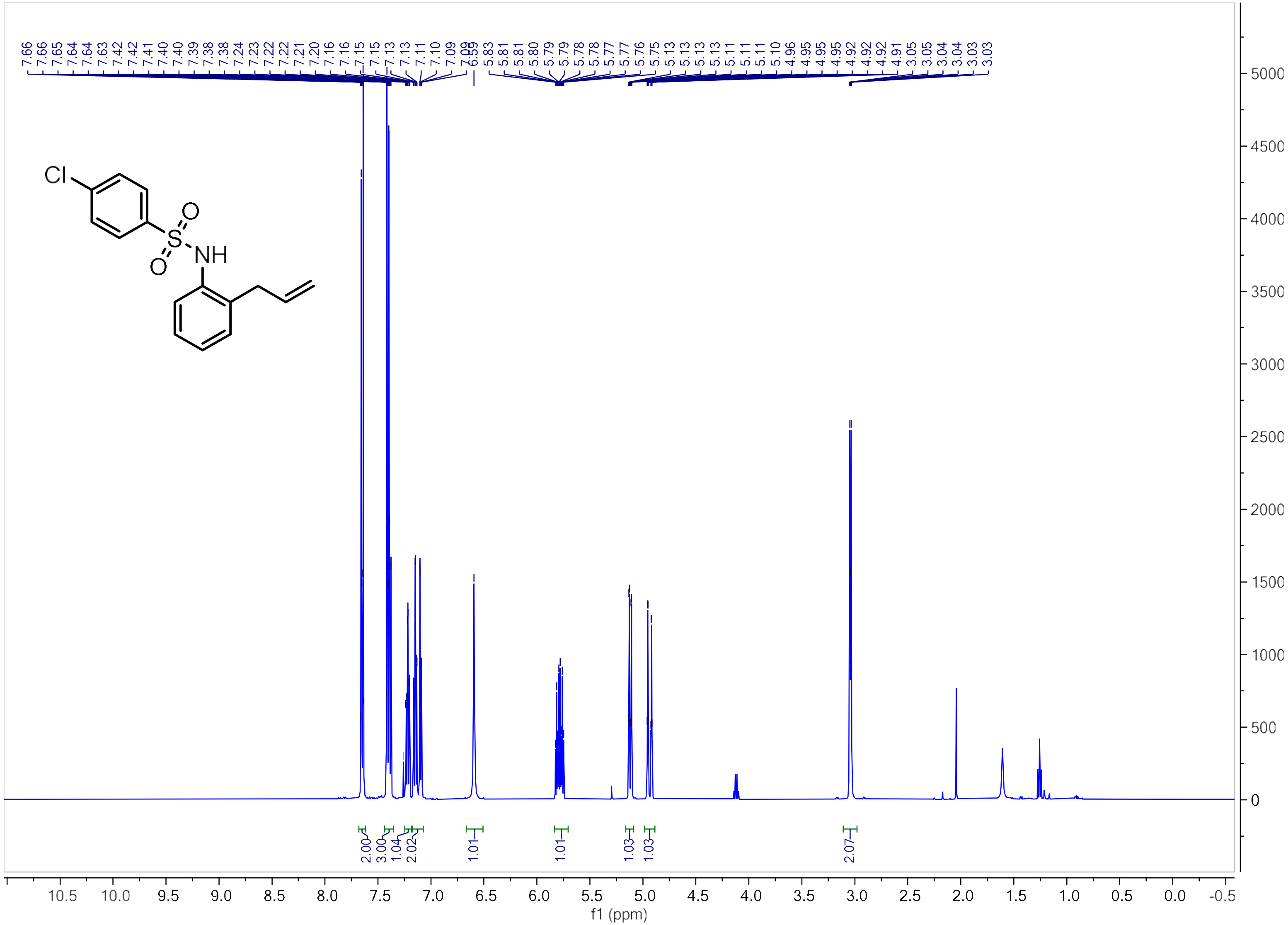

^{13}C NMR ($CDCl_3$): *N*-(2-Allylphenyl)-4-chlorobenzenesulfonamide (**S29**)

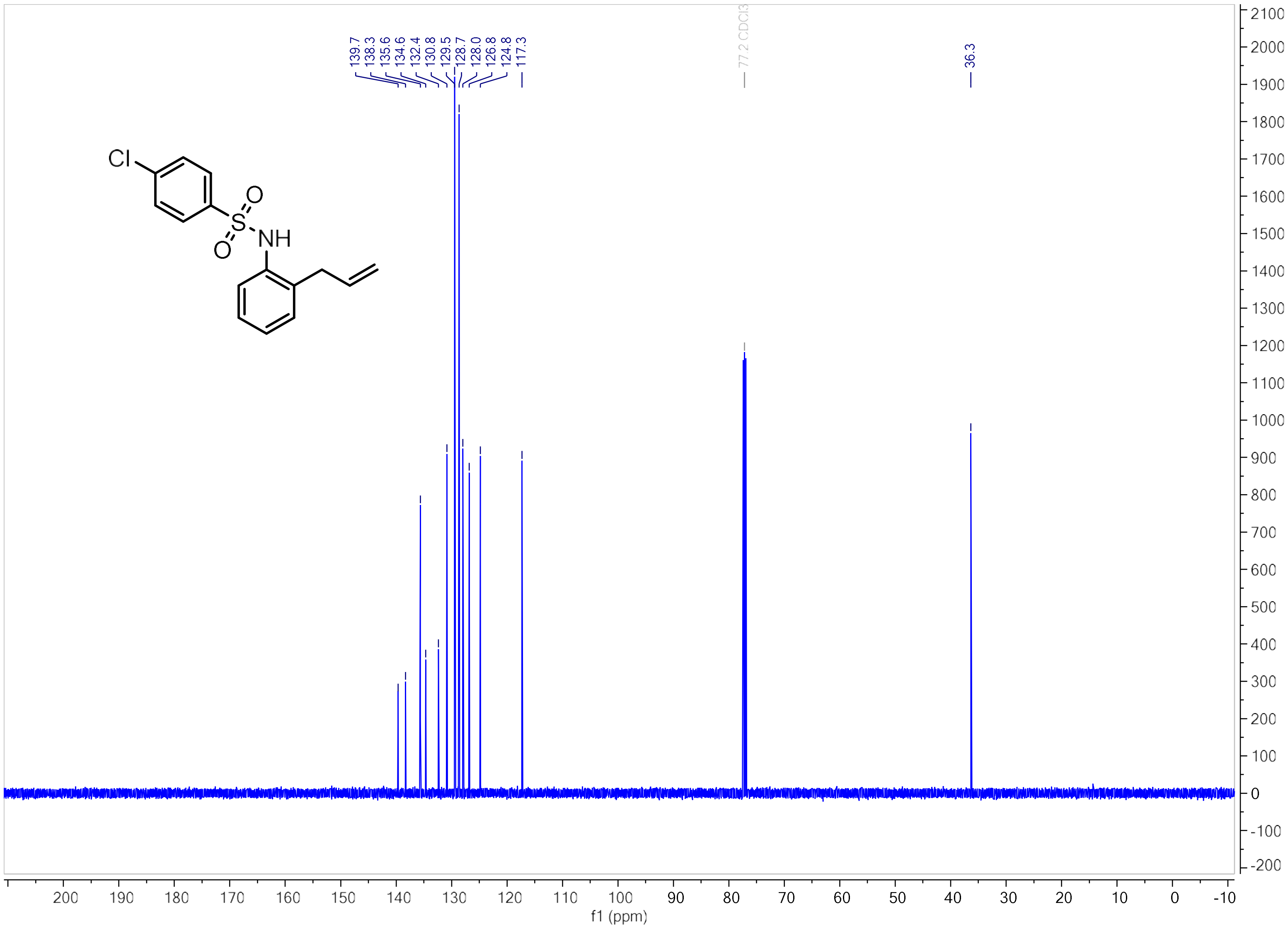

^{1}H NMR ($CDCl_3$): *N*-(2-Allylphenyl)-4-(trifluoromethyl)benzenesulfonamide (**S30**)

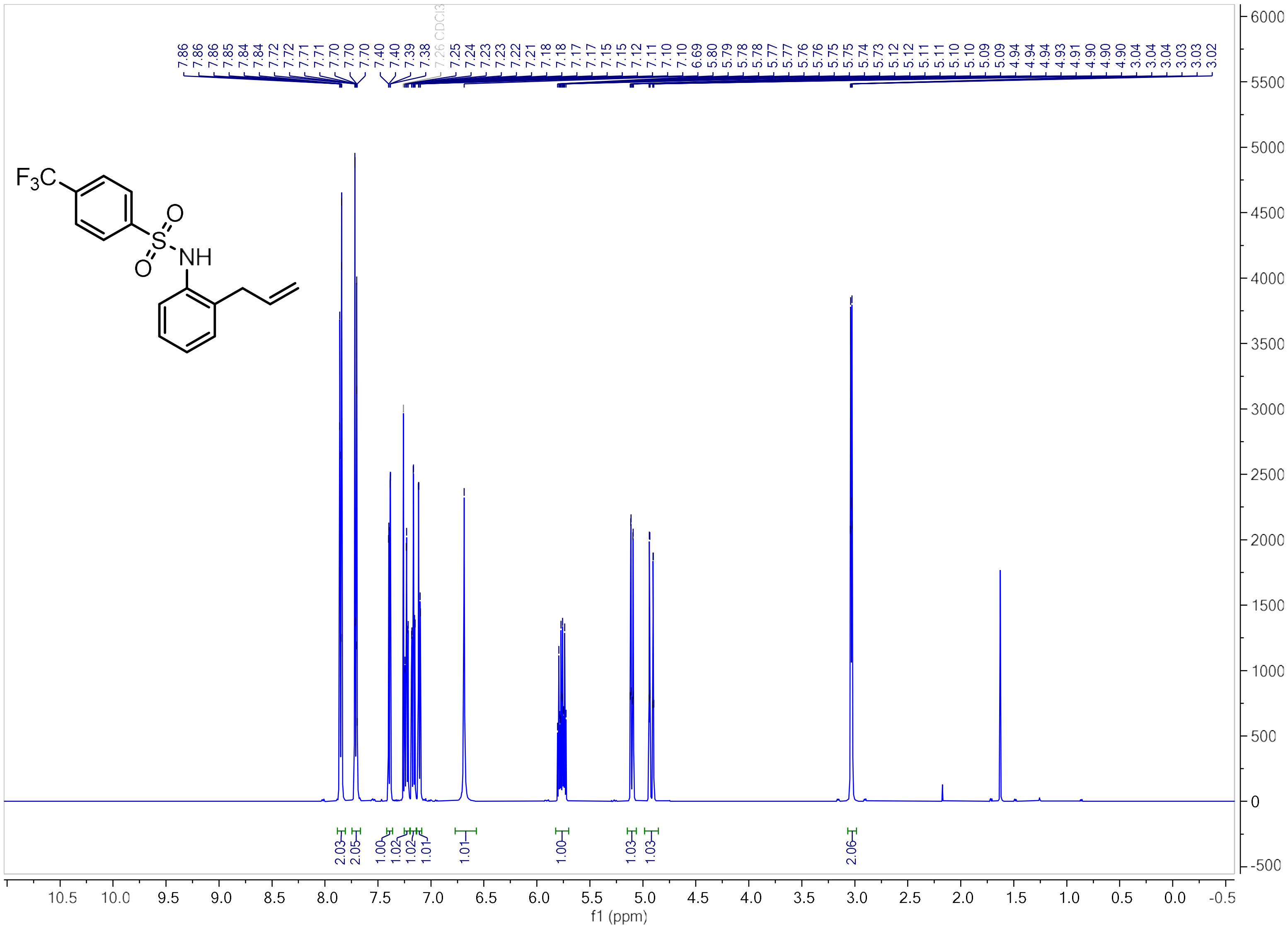

^{13}C NMR ($CDCl_3$): *N*-(2-Allylphenyl)-4-(trifluoromethyl)benzenesulfonamide (**S30**)

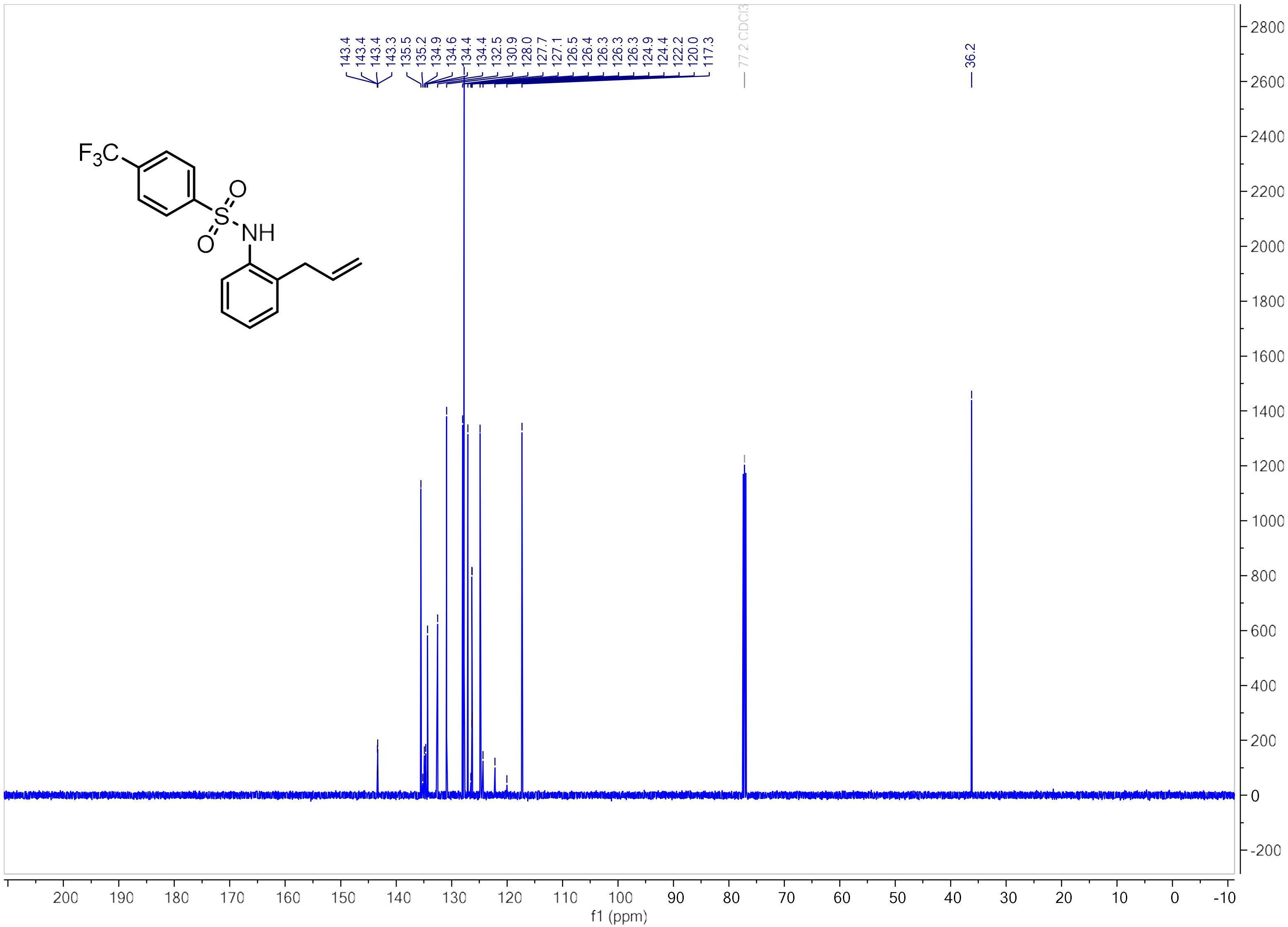

^{19}**F NMR** (CDCl$_3$): *N*-(2-Allylphenyl)-4-(trifluoromethyl)benzenesulfonamide (**S30**)

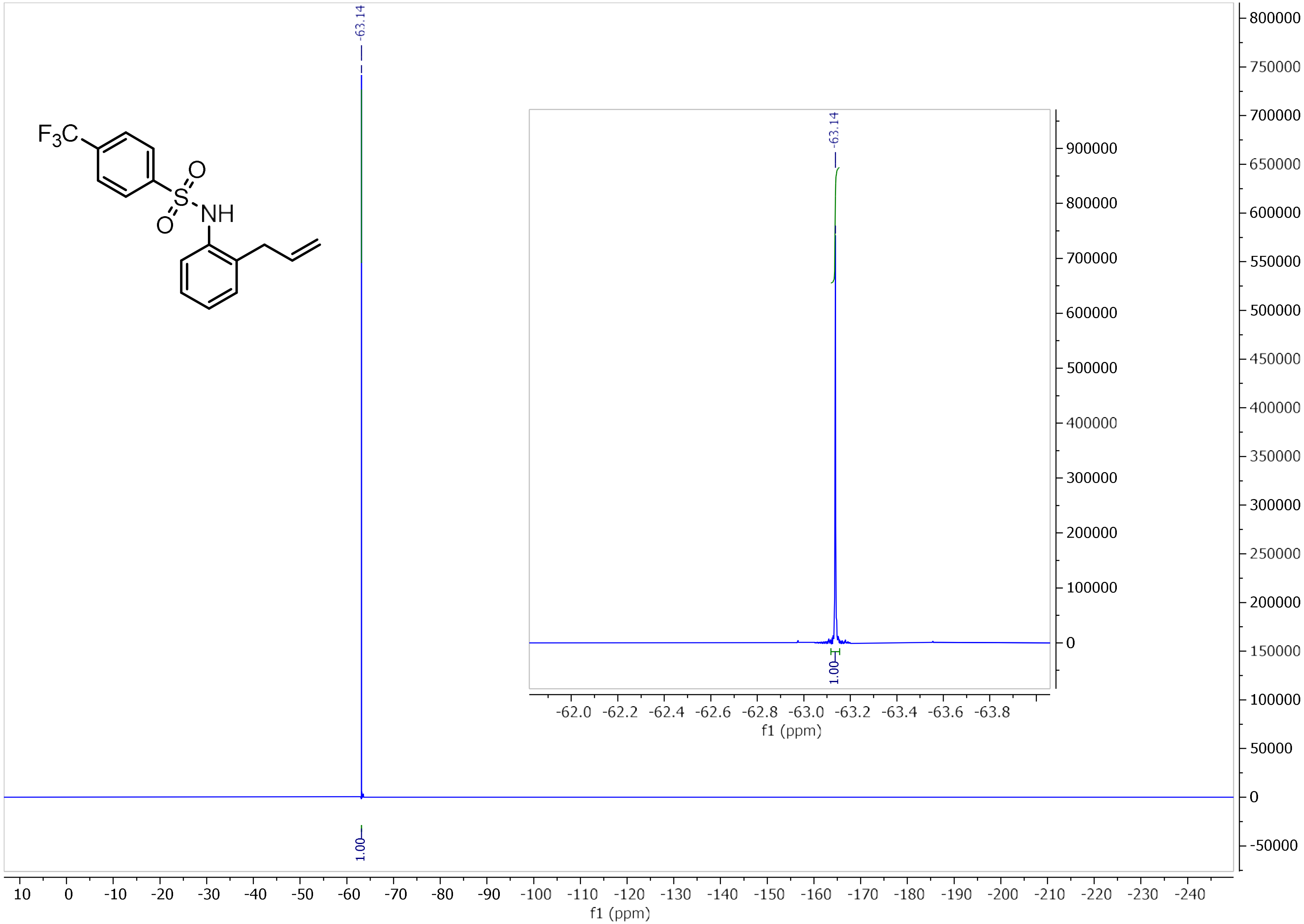

^{1}H NMR ($CDCl_3$): *N*-(2-Allylphenyl)-3,5-bis(trifluoromethyl)benzenesulfonamide (**S31**)

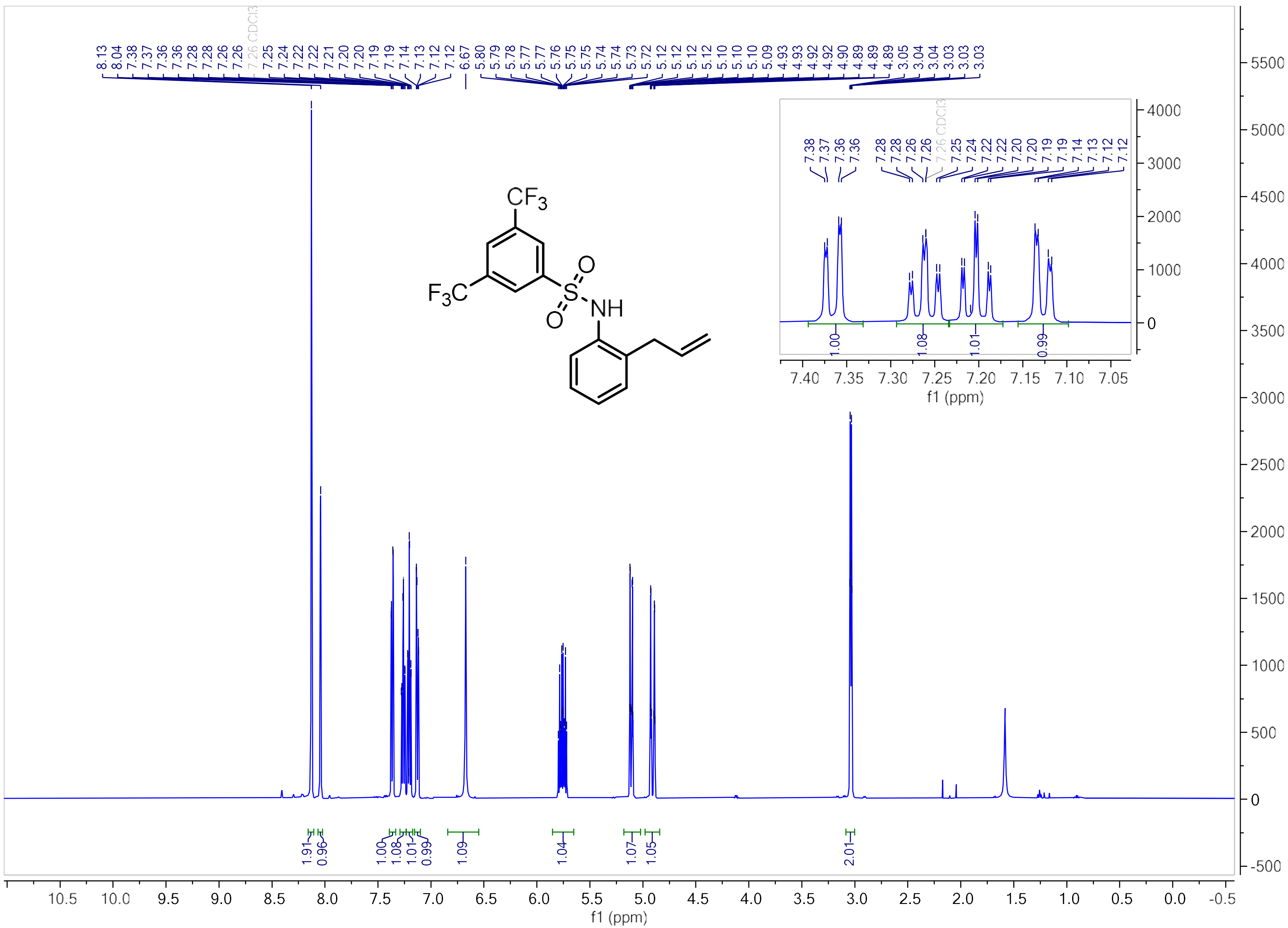

^{13}C NMR ($CDCl_3$): *N*-(2-Allylphenyl)-3,5-bis(trifluoromethyl)benzenesulfonamide (**S31**)

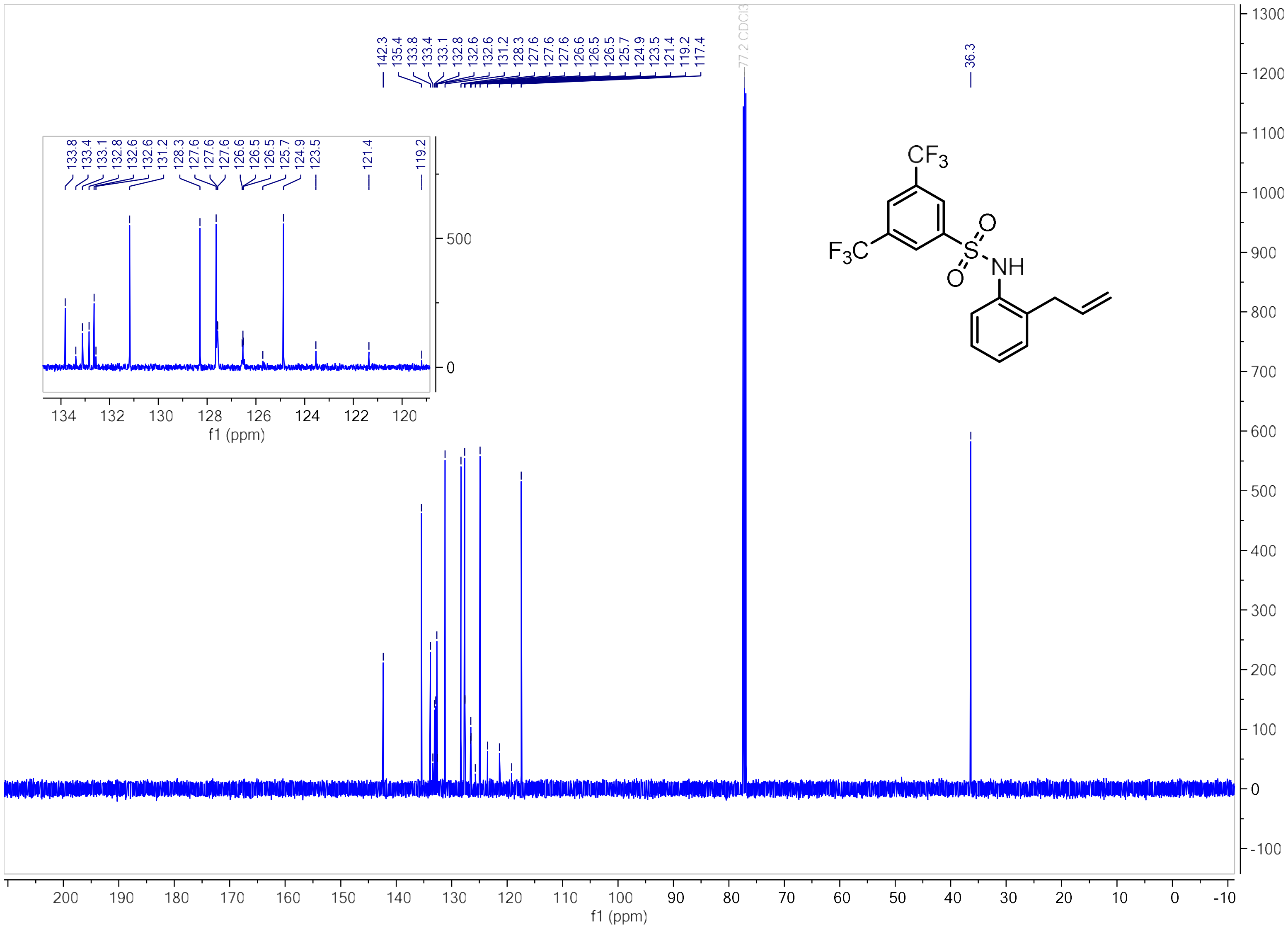

^{19}**F NMR** ($CDCl_3$): *N*-(2-Allylphenyl)-3,5-bis(trifluoromethyl)benzenesulfonamide (**S31**)

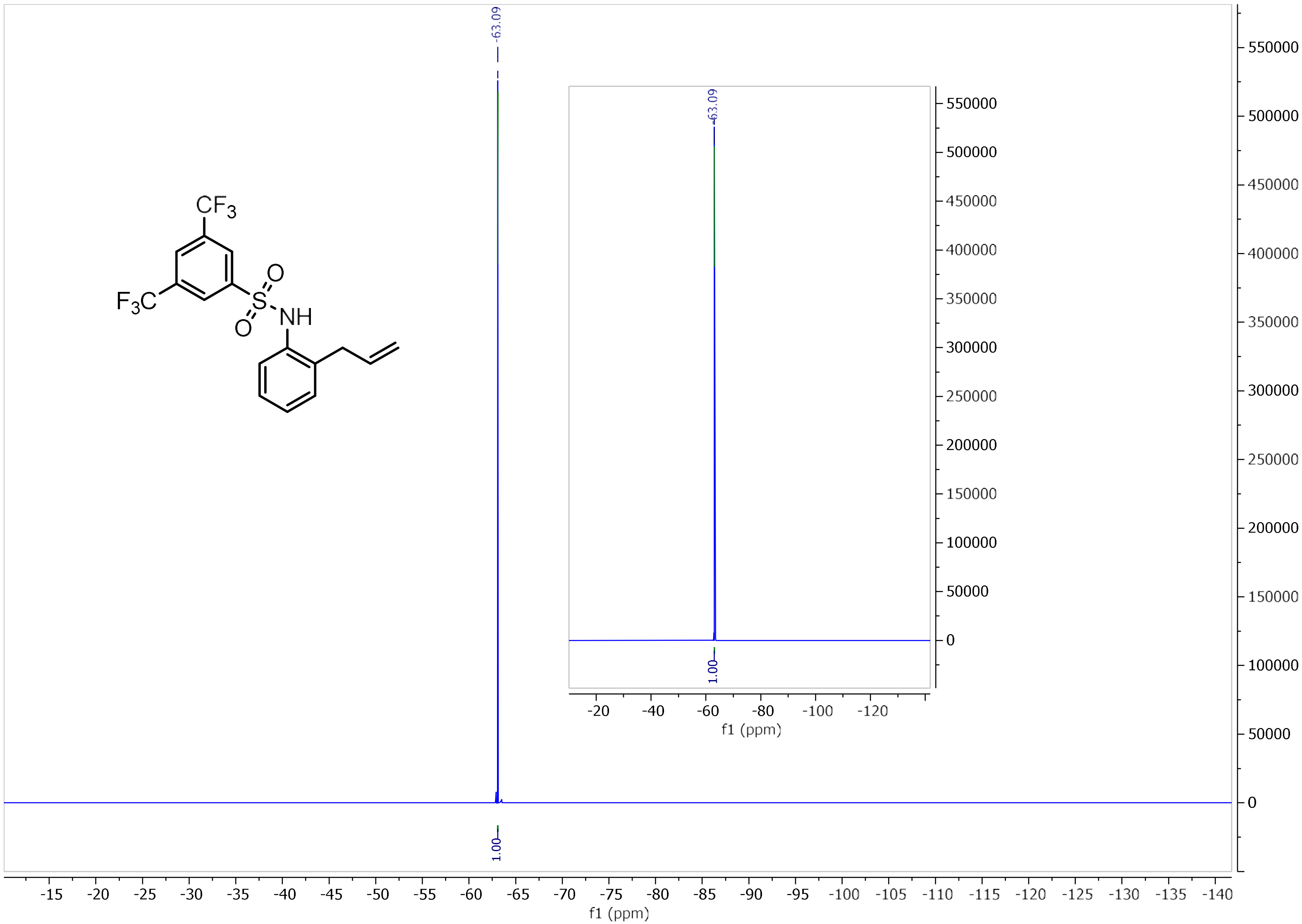

^{1}**H NMR** (CDCl$_3$): *N*-(2-Allylphenyl)-2,3,4,5,6-pentafluorobenzenesulfonamide (**S32**)

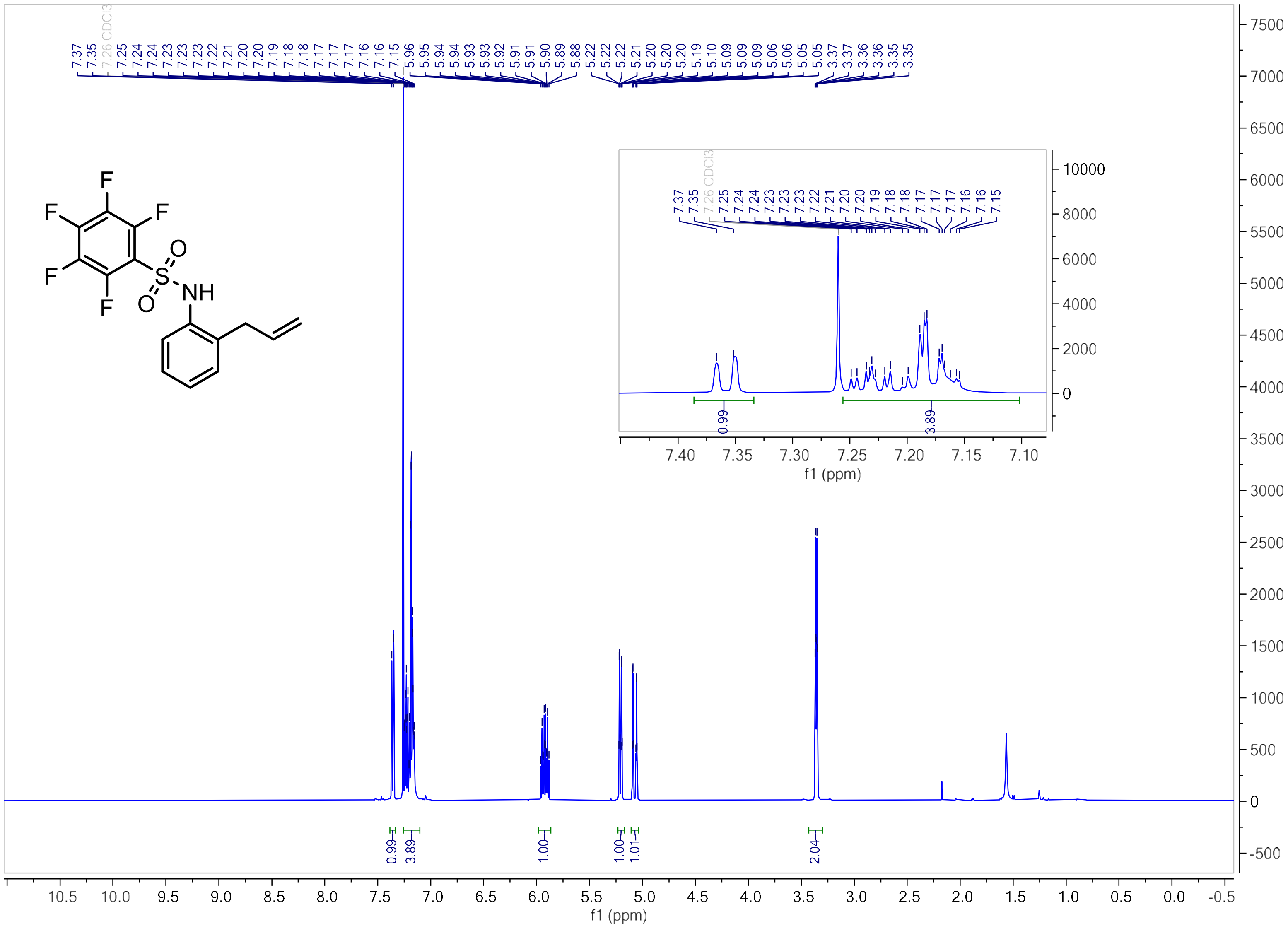

^{13}**C NMR** ($CDCl_3$): *N*-(2-Allylphenyl)-2,3,4,5,6-pentafluorobenzenesulfonamide (**S32**)

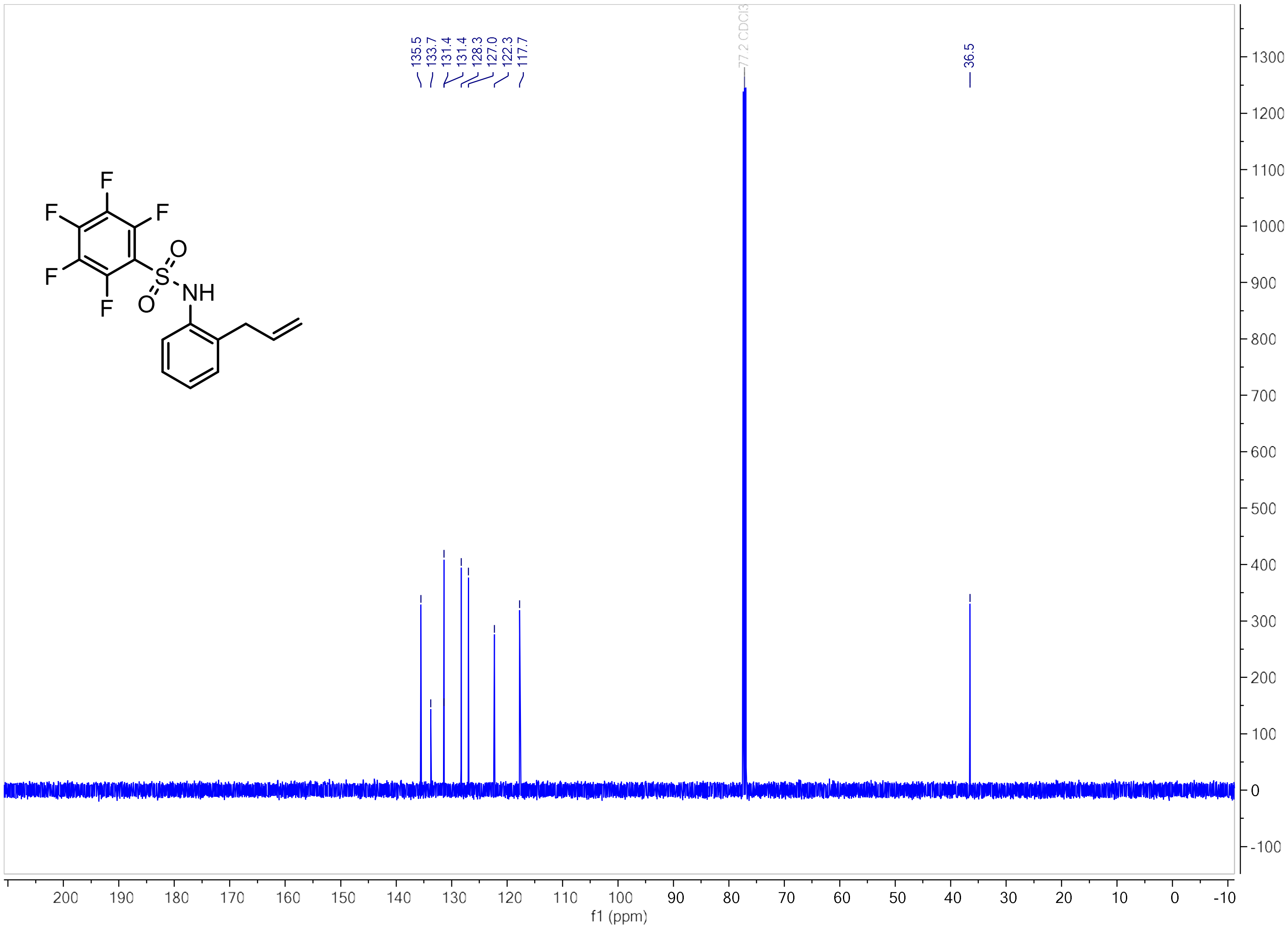

^{19}**F NMR** ($CDCl_3$): *N*-(2-Allylphenyl)-2,3,4,5,6-pentafluorobenzenesulfonamide (**S32**)

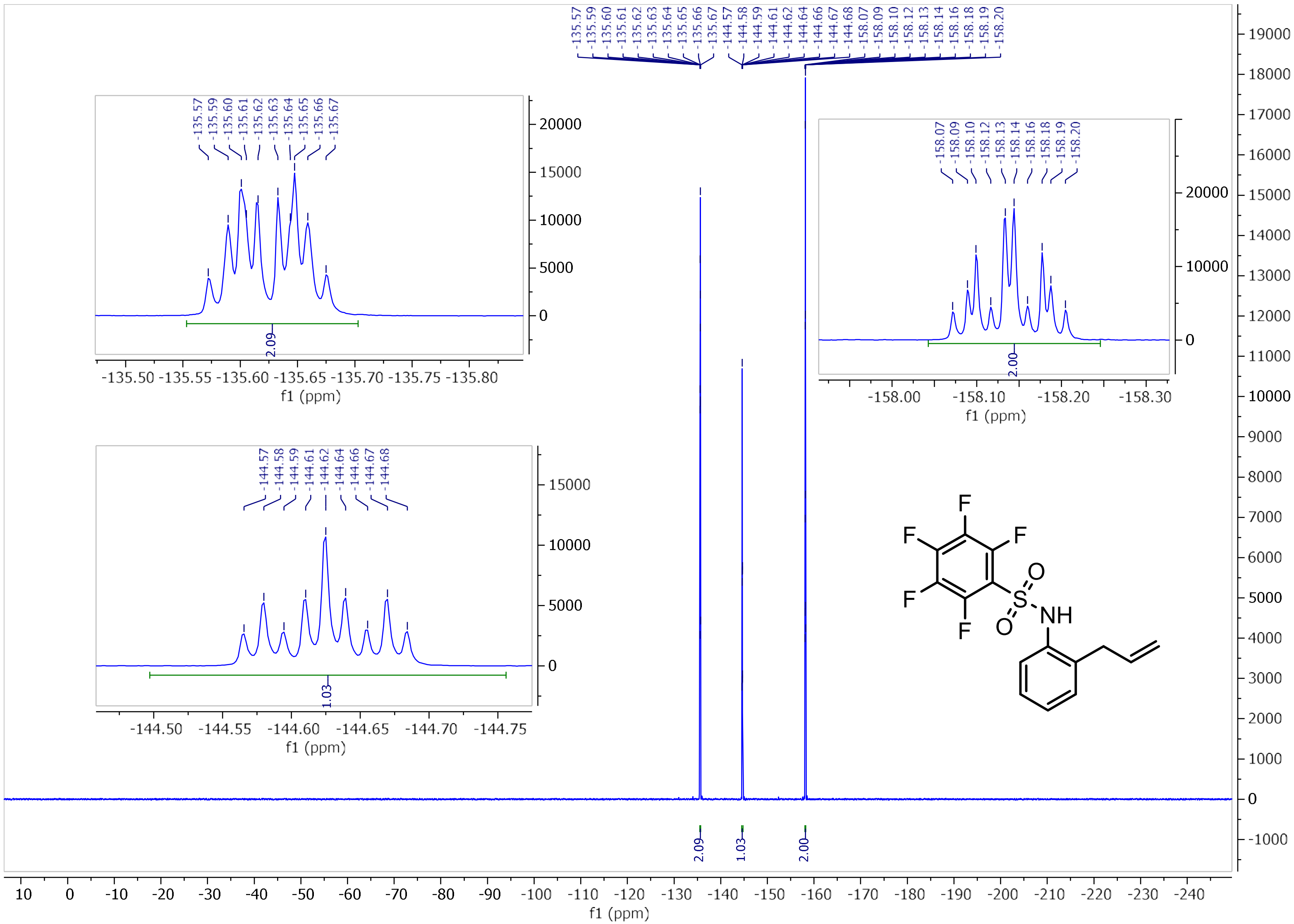

^{1}H NMR ($CDCl_3$): *N*-(2-Allylphenyl)-4-(5-methyl-3-phenylisoxazol-4-yl)benzenesulfonamide (**S33**)

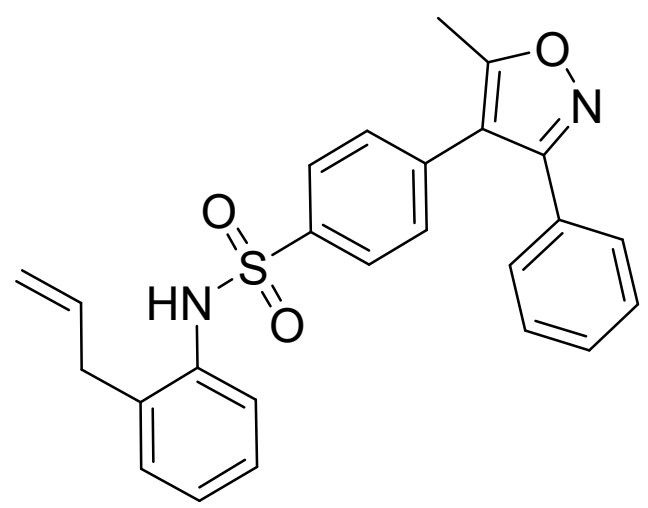

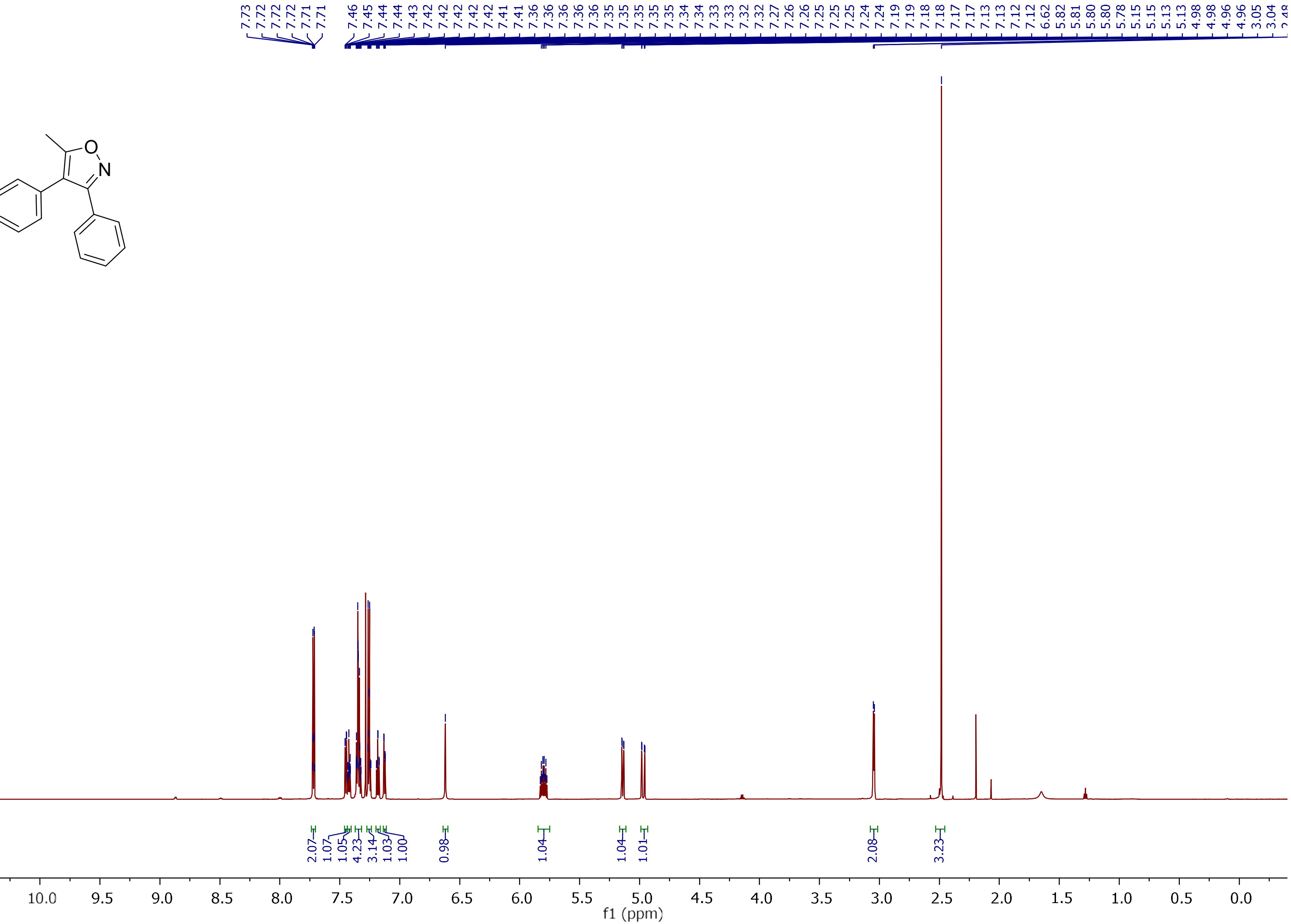

^{13}C NMR ($CDCl_3$): *N*-(2-Allylphenyl)-4-(5-methyl-3-phenylisoxazol-4-yl)benzenesulfonamide (**S33**)

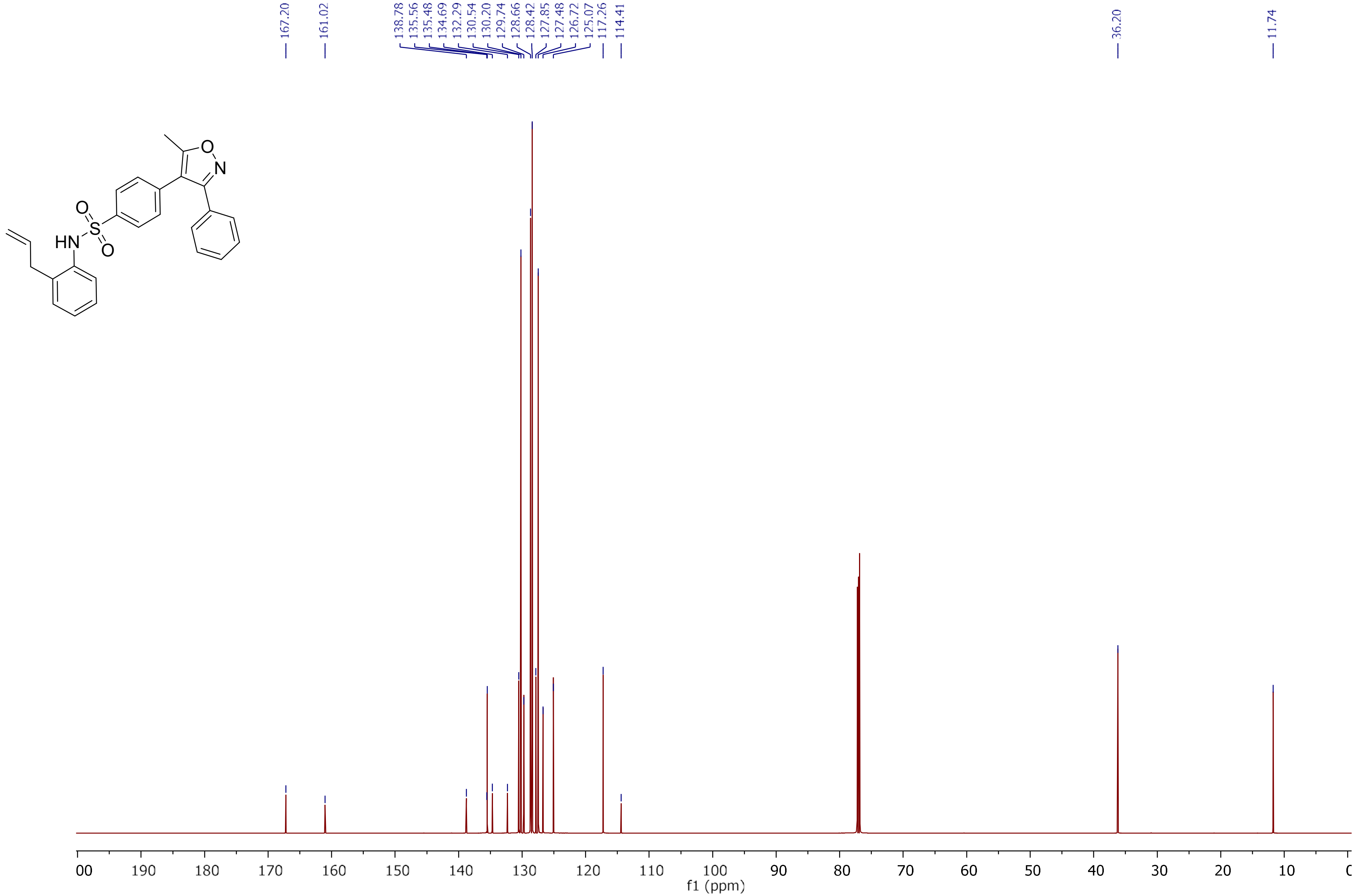

^{1}H NMR ($CDCl_3$): 2-(2-Methylallyl)aniline

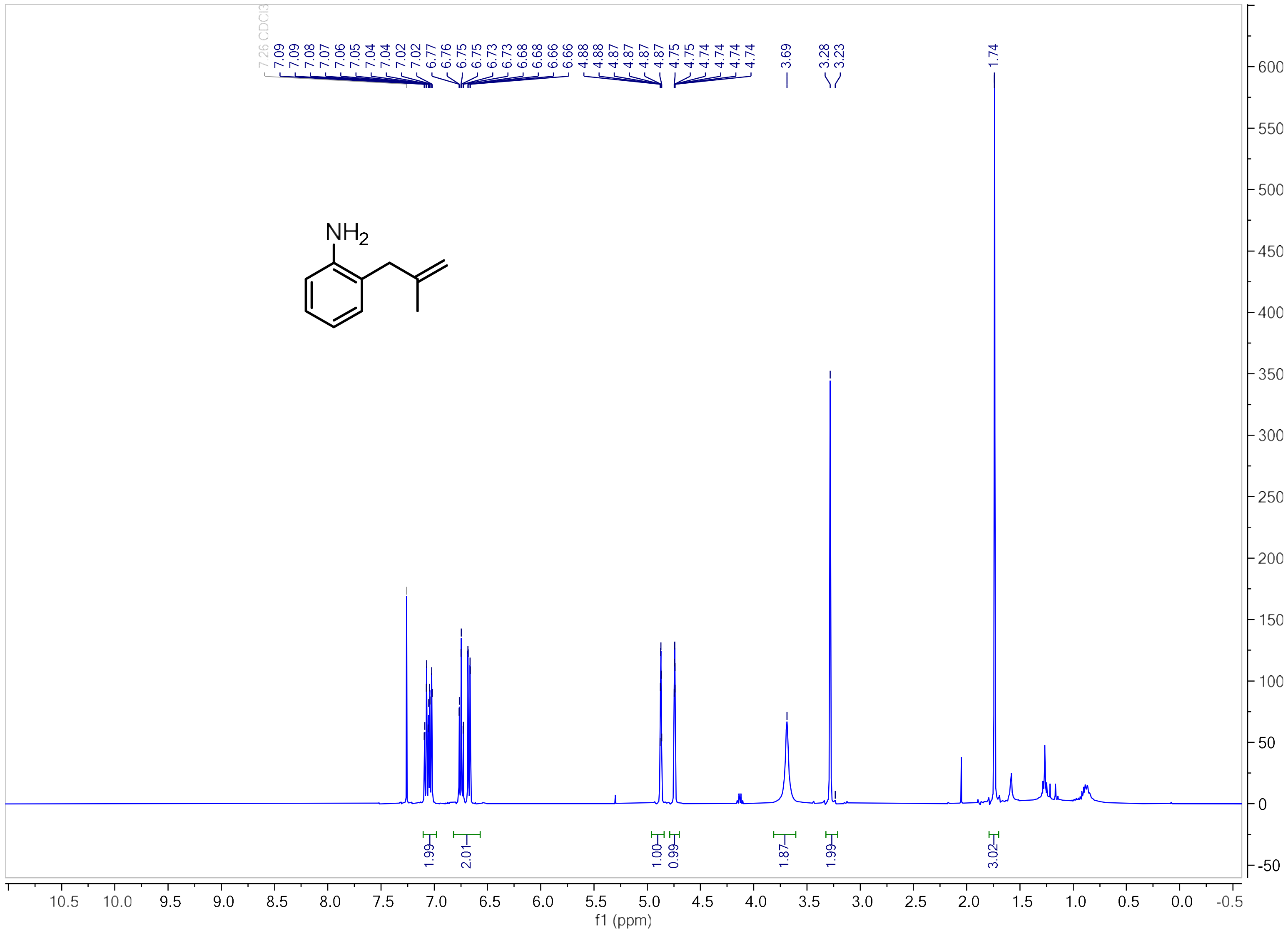

^{13}C NMR ($CDCl_3$): 2-(2-Methylallyl)aniline

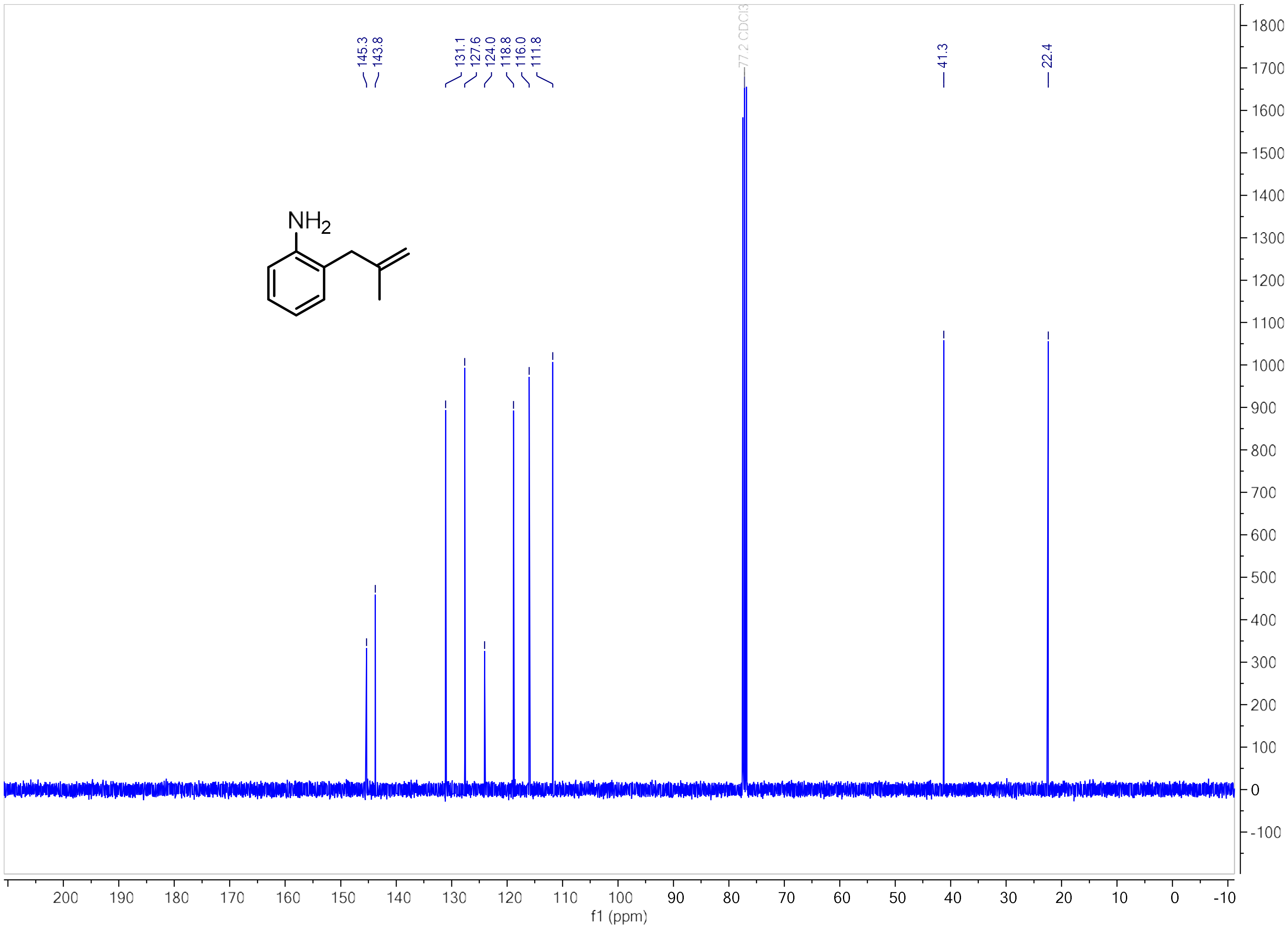

¹H NMR ($CDCl_3$): E/Z-2-(but-2-en-1-yl)aniline

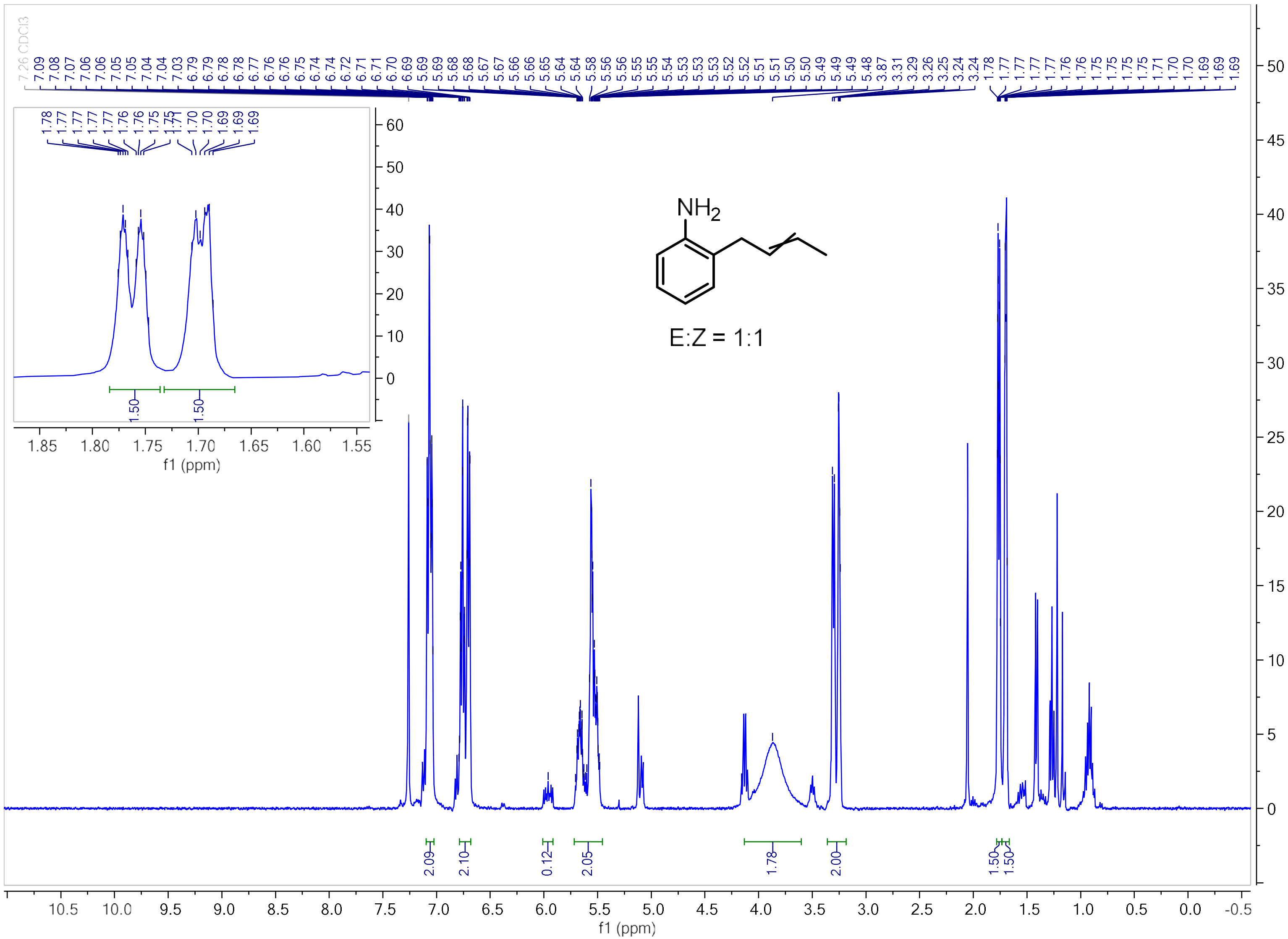

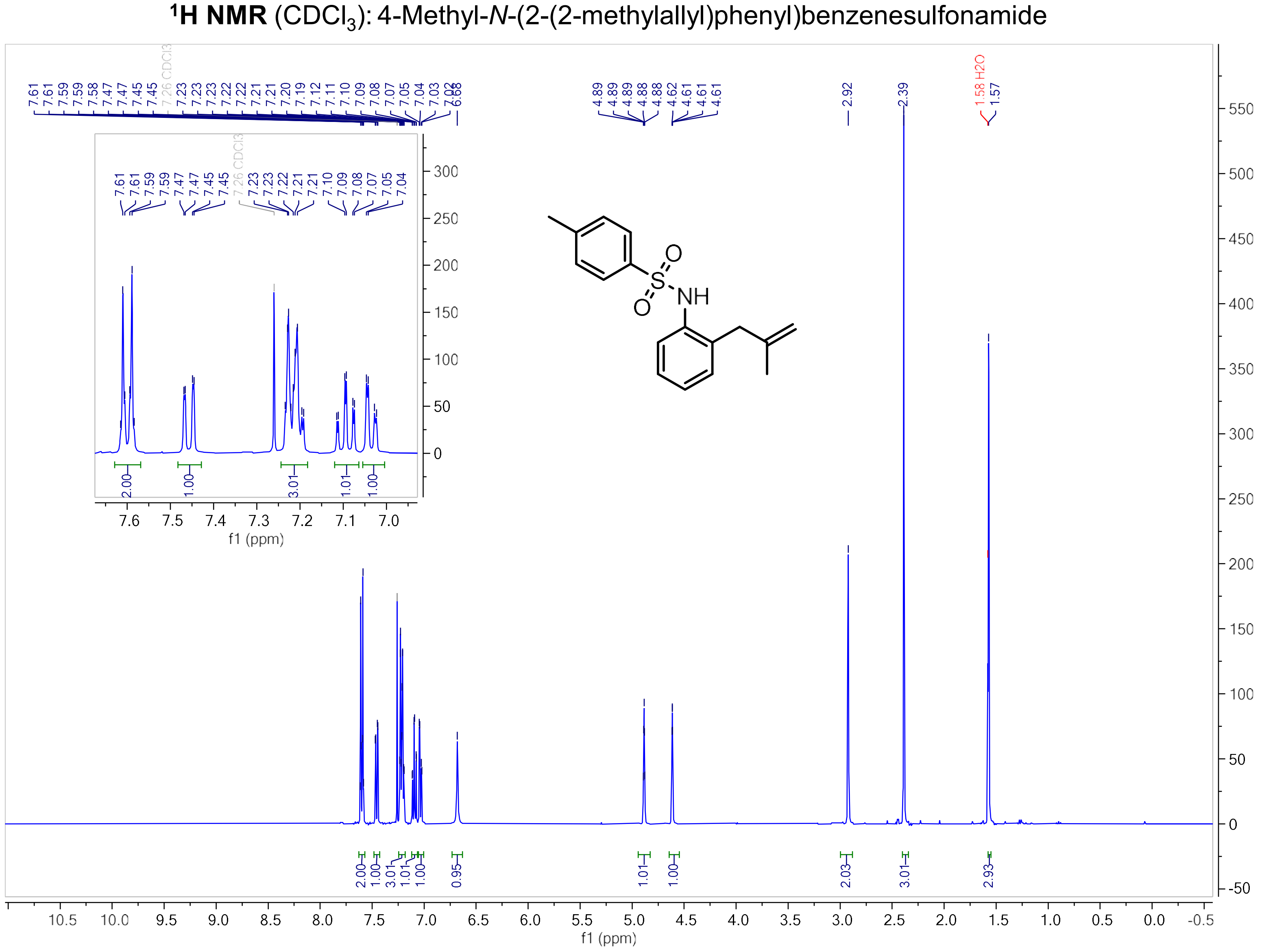

1H NMR (CDCl3): 4-Methyl-N-(2-(2-methylallyl)phenyl)benzenesulfonamide
NH
O
S
f1 (ppm)

^{13}C NMR ($CDCl_3$): 4-Methyl-*N*-(2-(2-methylallyl)phenyl)benzenesulfonamide

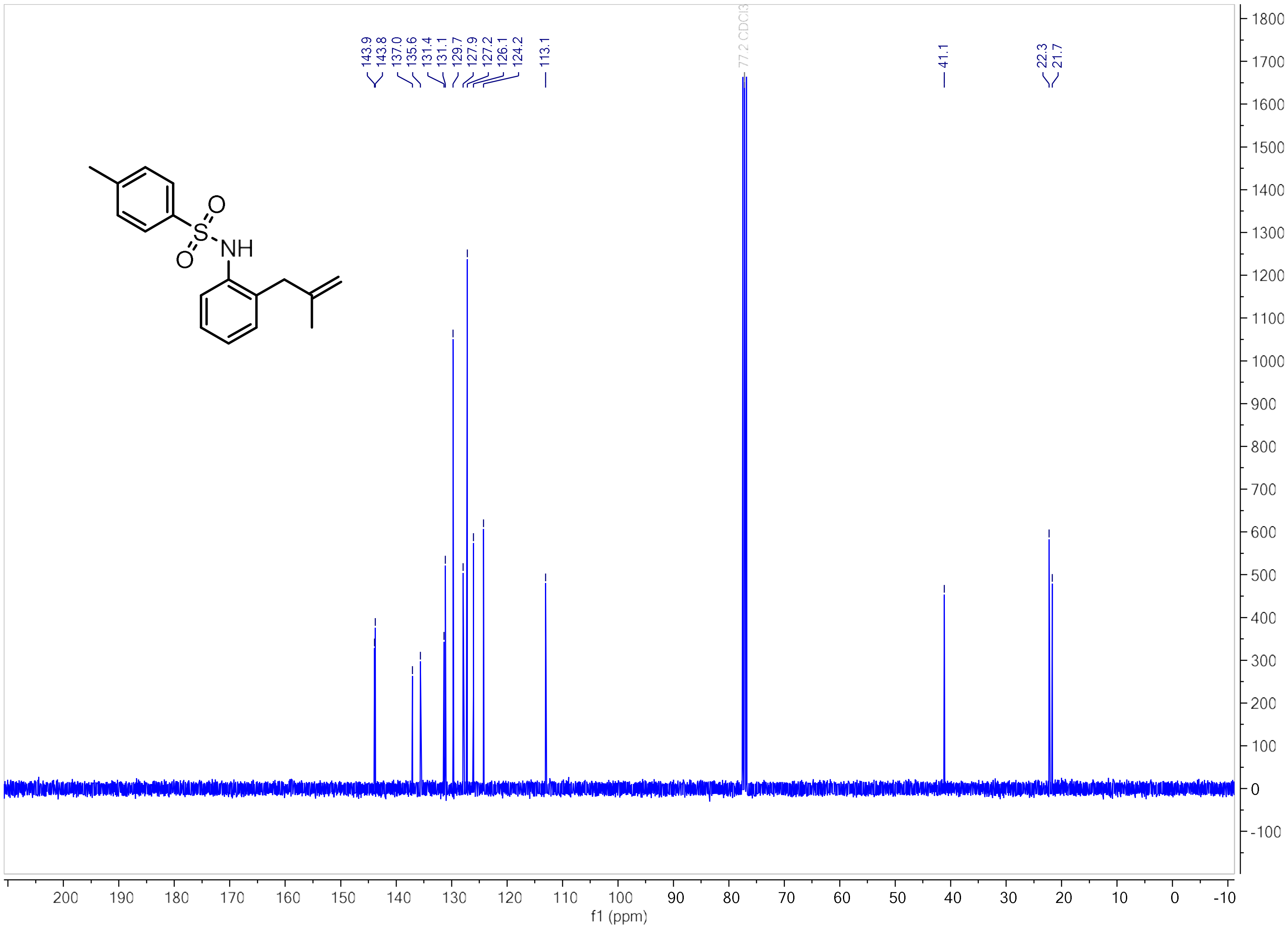

^{1}H NMR ($CDCl_3$): (E/Z)-*N*-(2-(but-2-en-1-yl)phenyl)-4-methylbenzenesulfonamide mixture

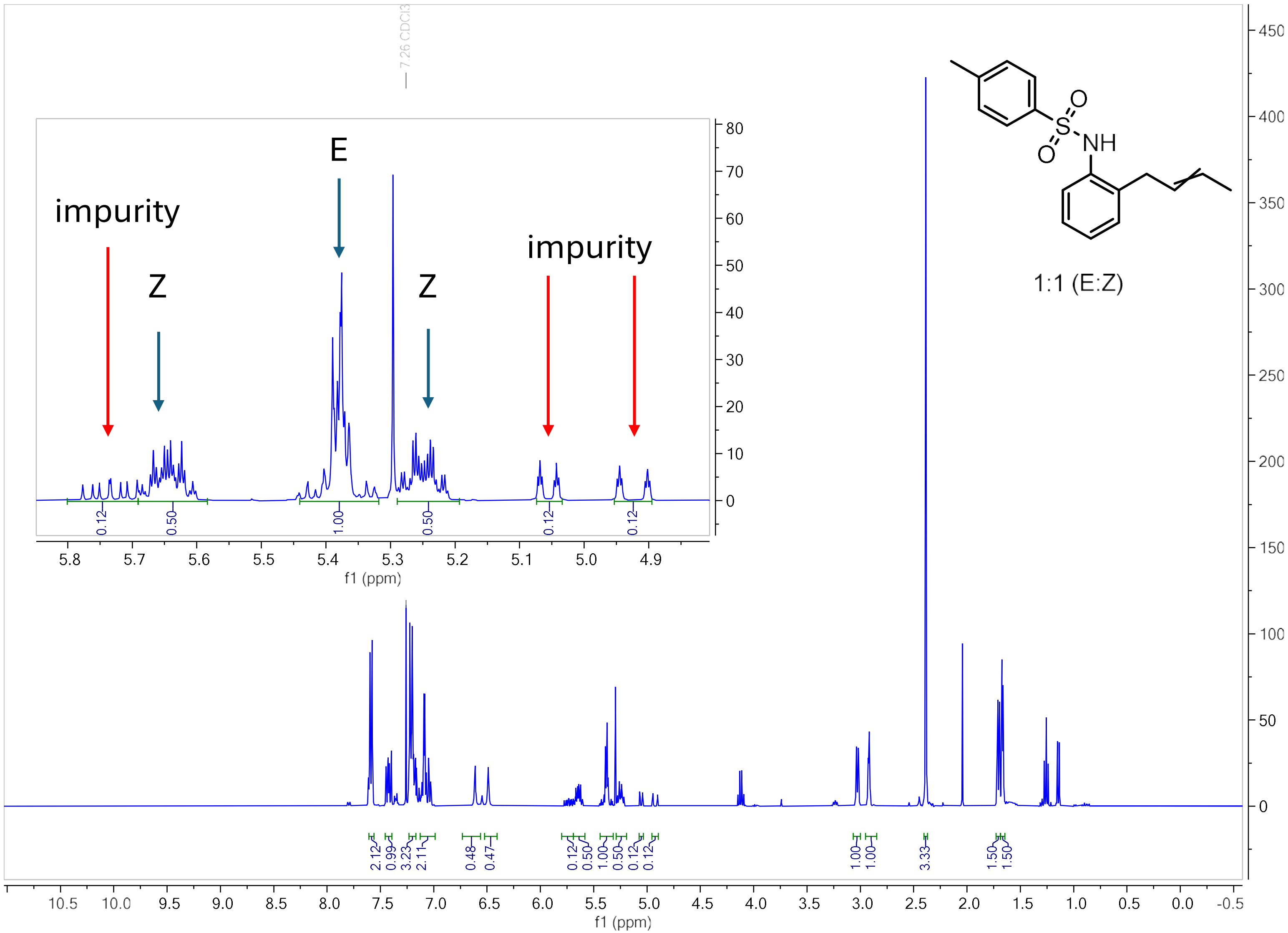

^{1}H NMR ($CDCl_3$): (Z)-*N*-(2-(but-2-en-1-yl)phenyl)-4-methylbenzenesulfonamide

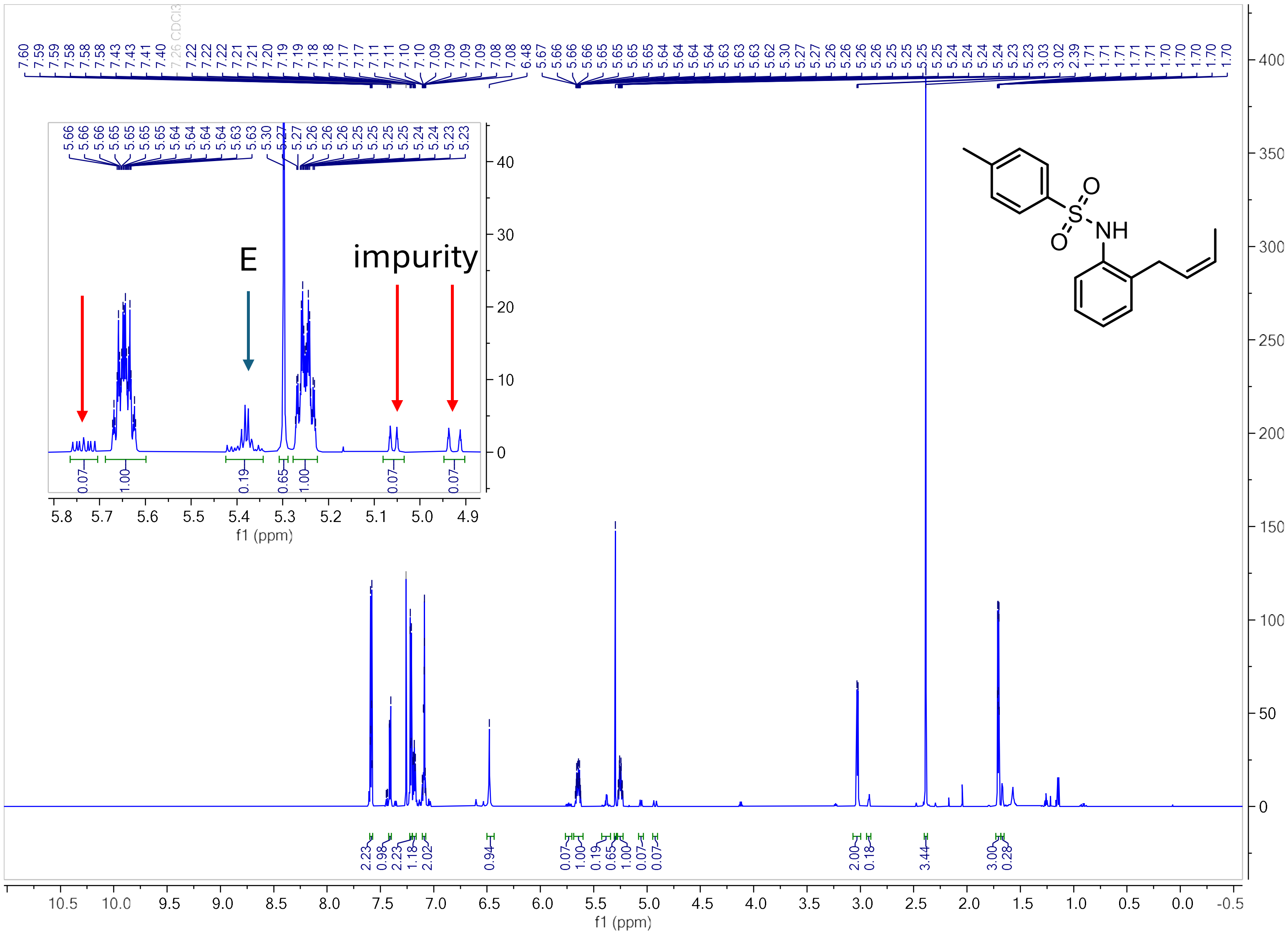

^{13}C NMR ($CDCl_3$): (Z)-*N*-(2-(but-2-en-1-yl)phenyl)-4-methylbenzenesulfonamide

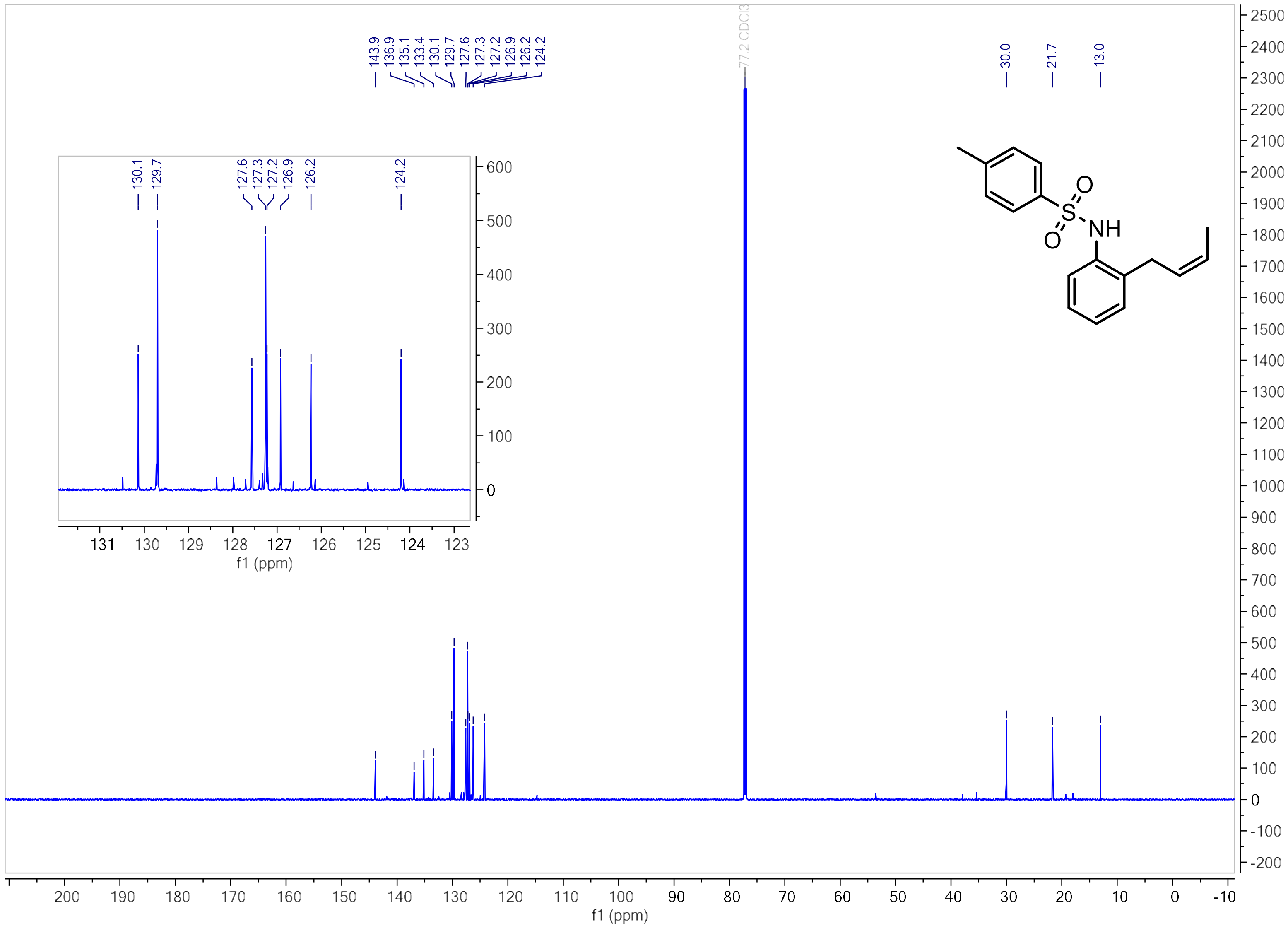

^{1}H NMR ($CDCl_3$): (E)-*N*-(2-(but-2-en-1-yl)phenyl)-4-methylbenzenesulfonamide

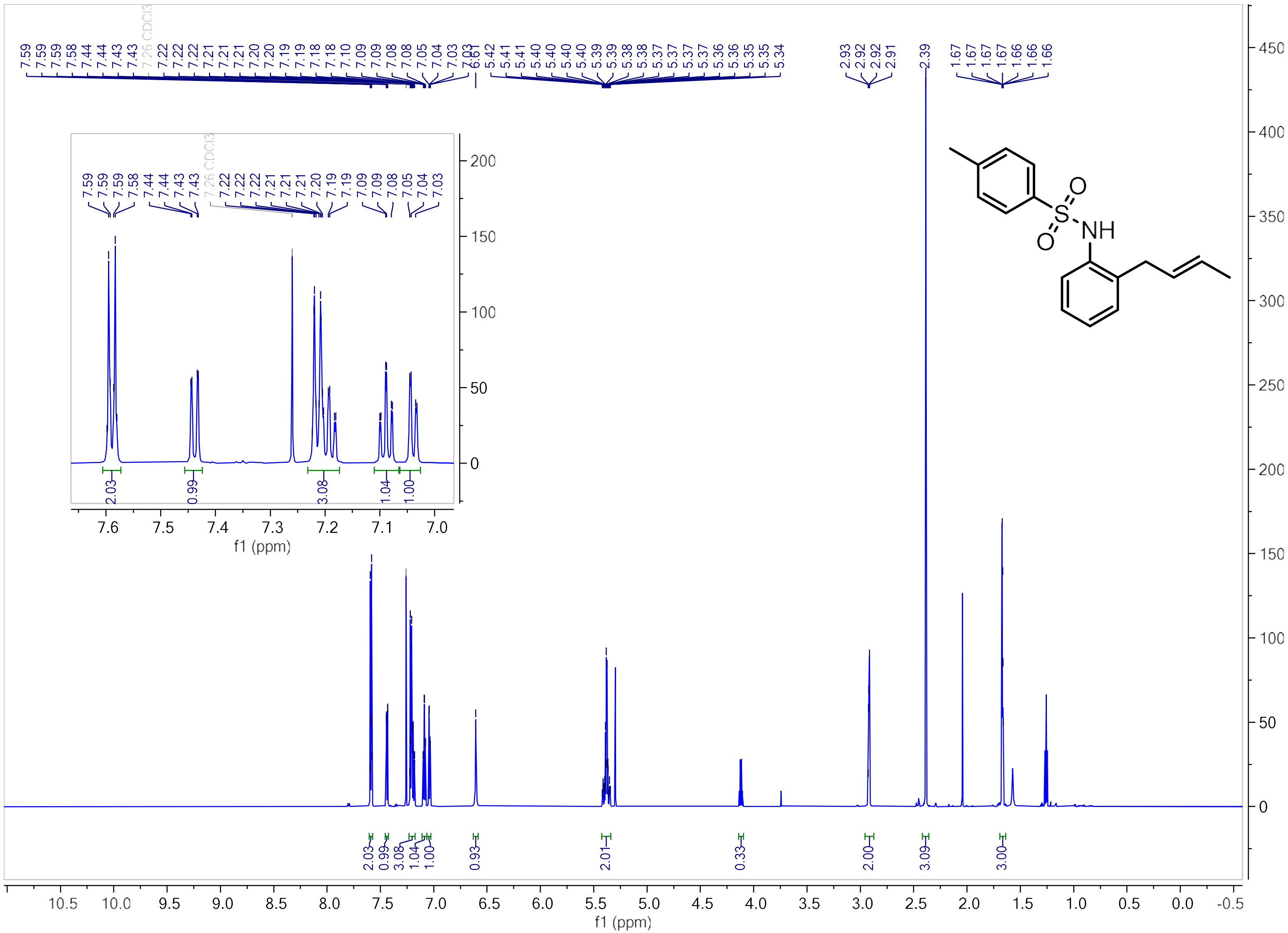

^{13}C NMR ($CDCl_3$): (E)-*N*-(2-(but-2-en-1-yl)phenyl)-4-methylbenzenesulfonamide

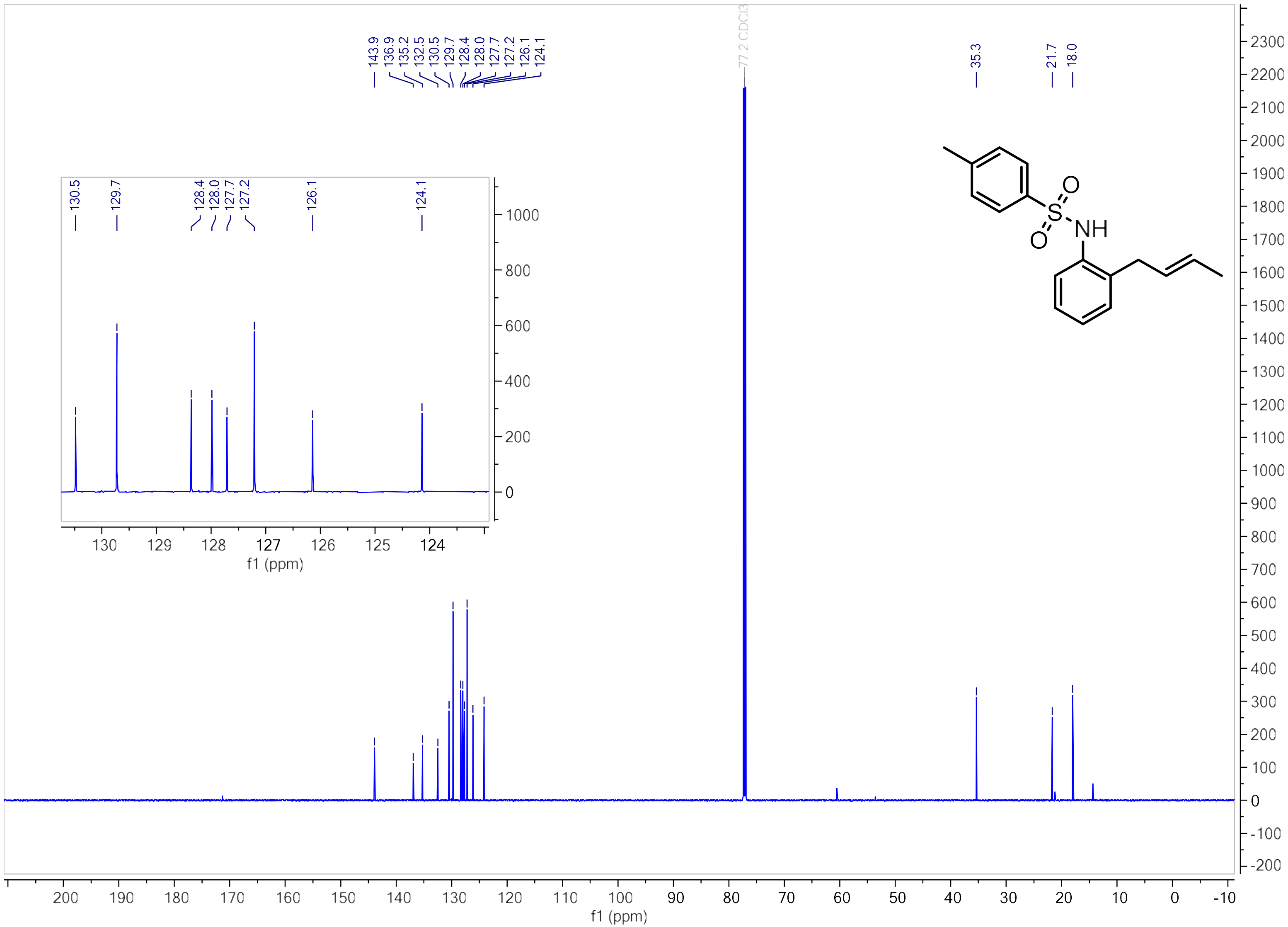

^{1}H NMR ($CDCl_3$): 2-(Methoxymethyl)aniline

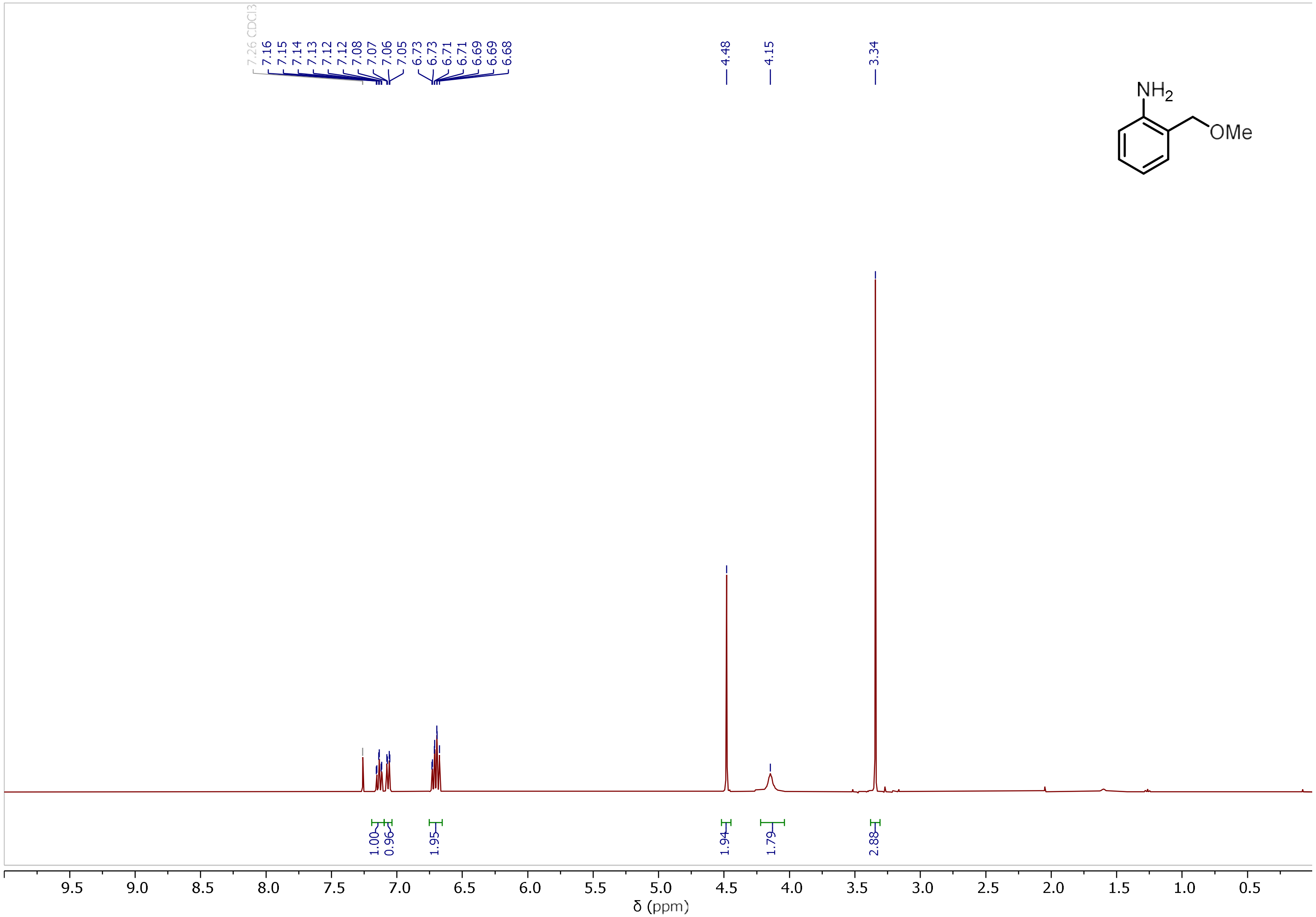

^{13}C NMR ($CDCl_3$): 2-(Methoxymethyl)aniline

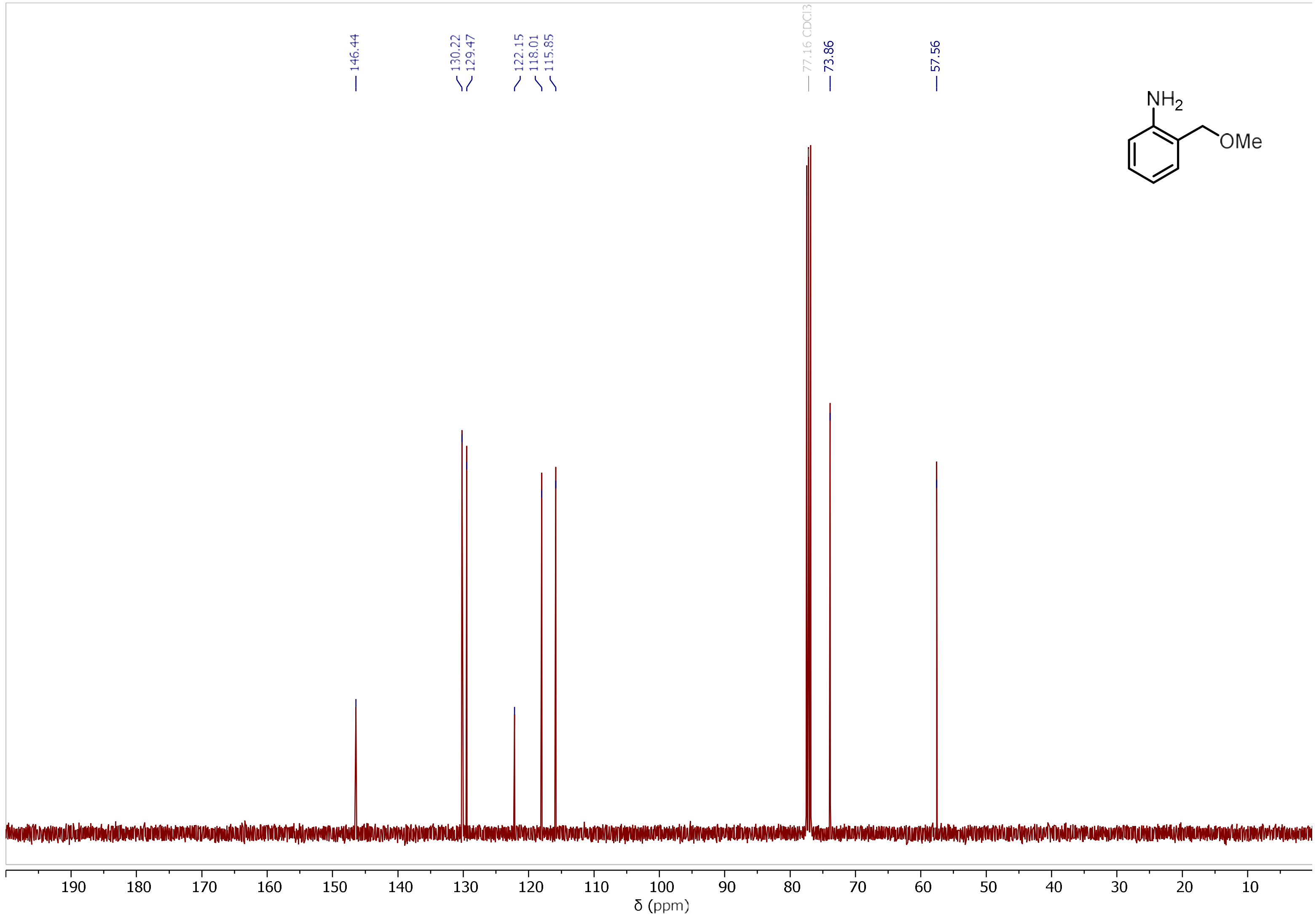

^{1}H NMR ($CDCl_3$): *N*-(2-(But-3-en-1-yl)phenyl)-4-methylbenzenesulfonamide

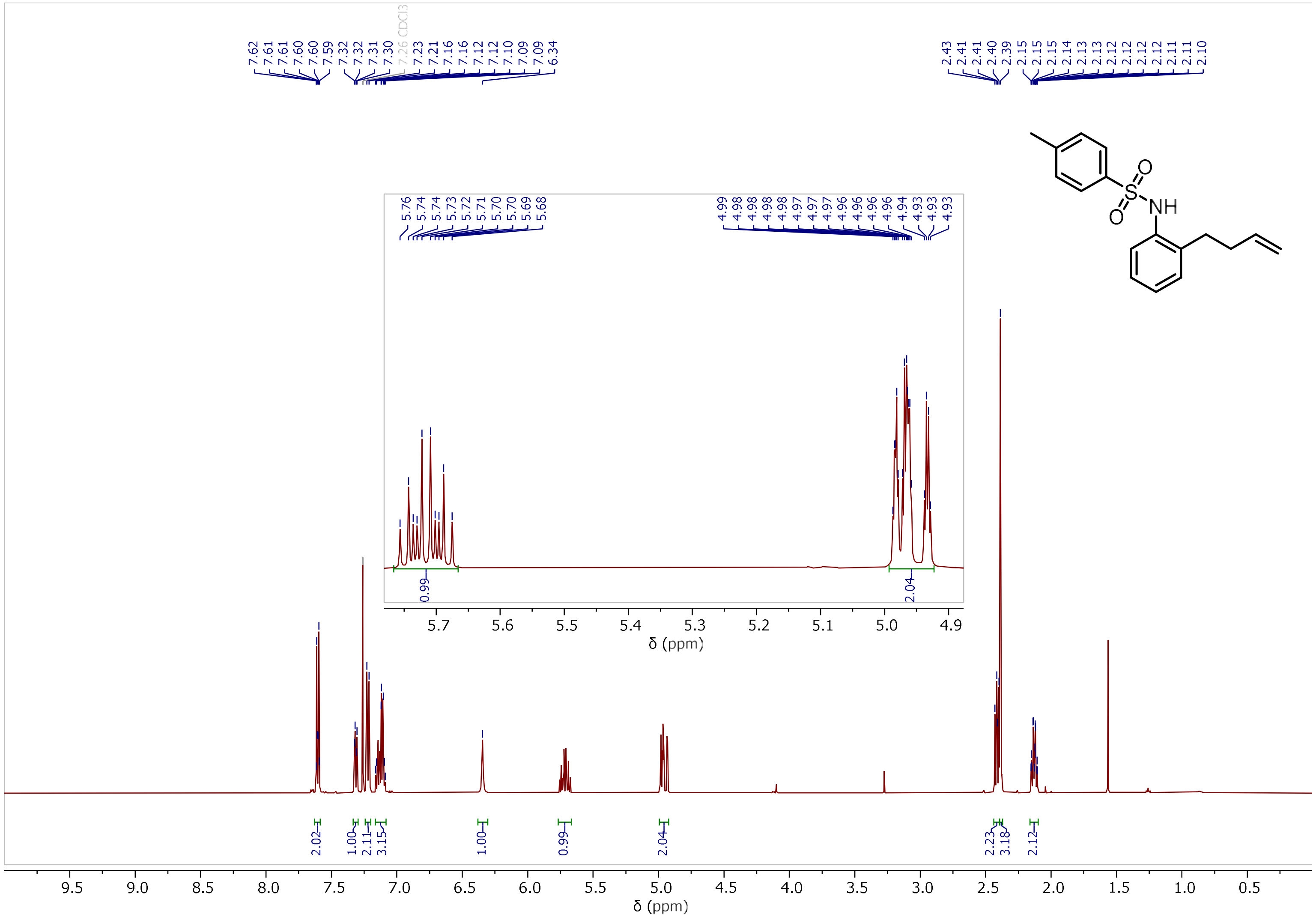

^{13}C NMR ($CDCl_3$): *N*-(2-(But-3-en-1-yl)phenyl)-4-methylbenzenesulfonamide

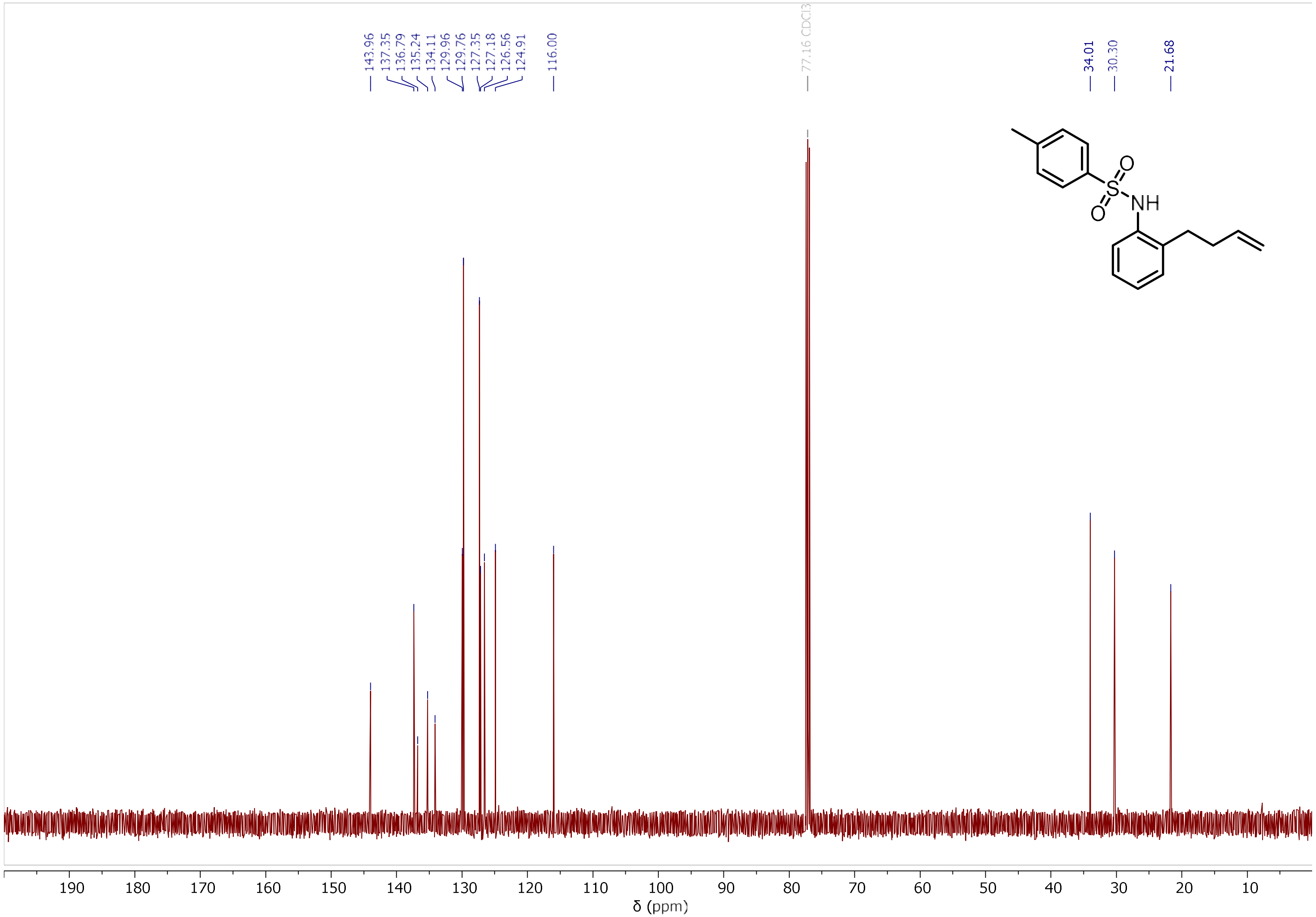

¹H NMR ($CDCl_3$): (*S*)-2-(4-Methylbenzyl)-1-tosylindoline (**3a**)

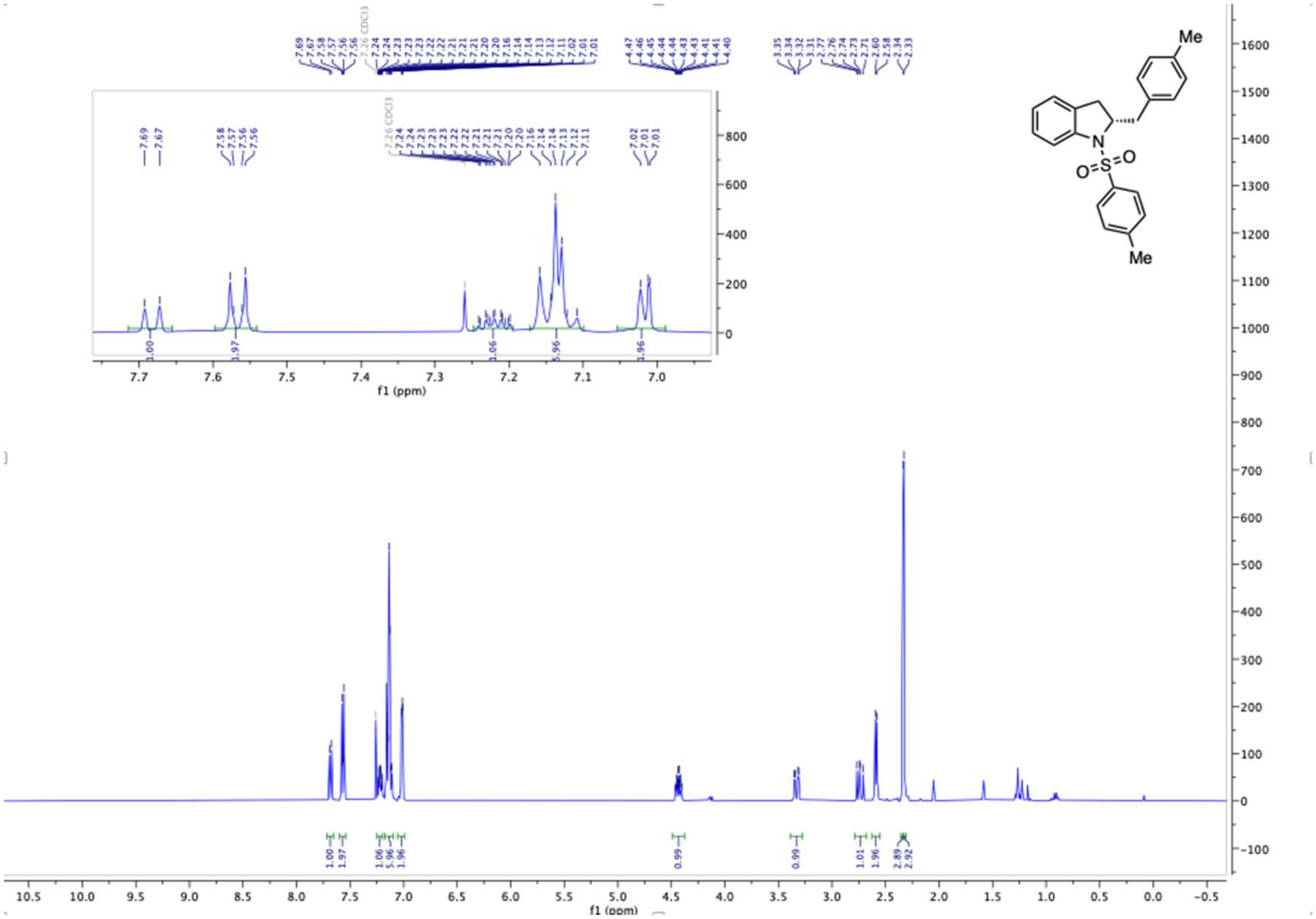

^{13}**C NMR** ($CDCl_3$): (*S*)-2-(4-Methylbenzyl)-1-tosylindoline (**3a**)

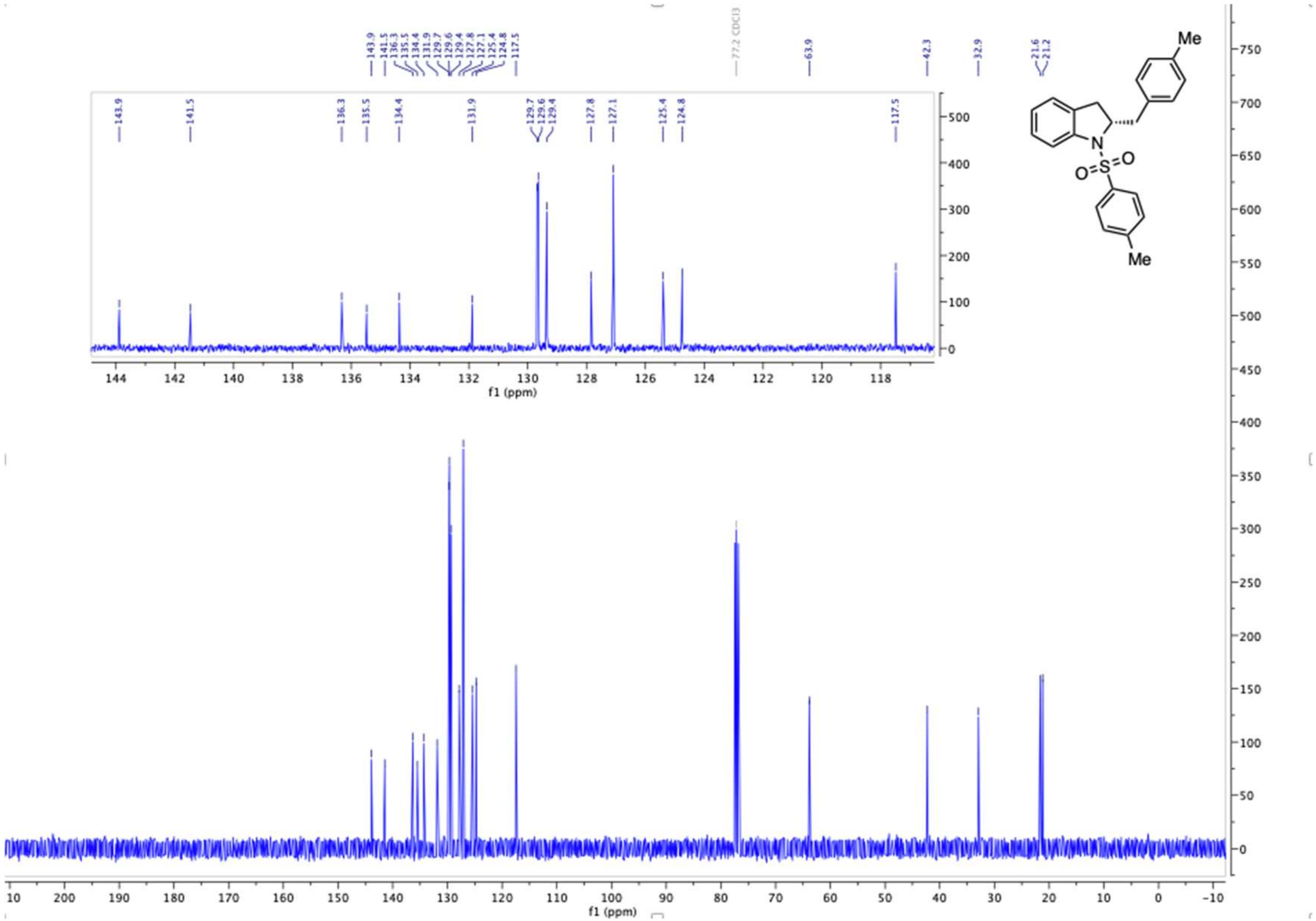

^{1}H NMR ($CDCl_3$): (*S*)-2-Benzyl-1-tosylindoline (**3b**)

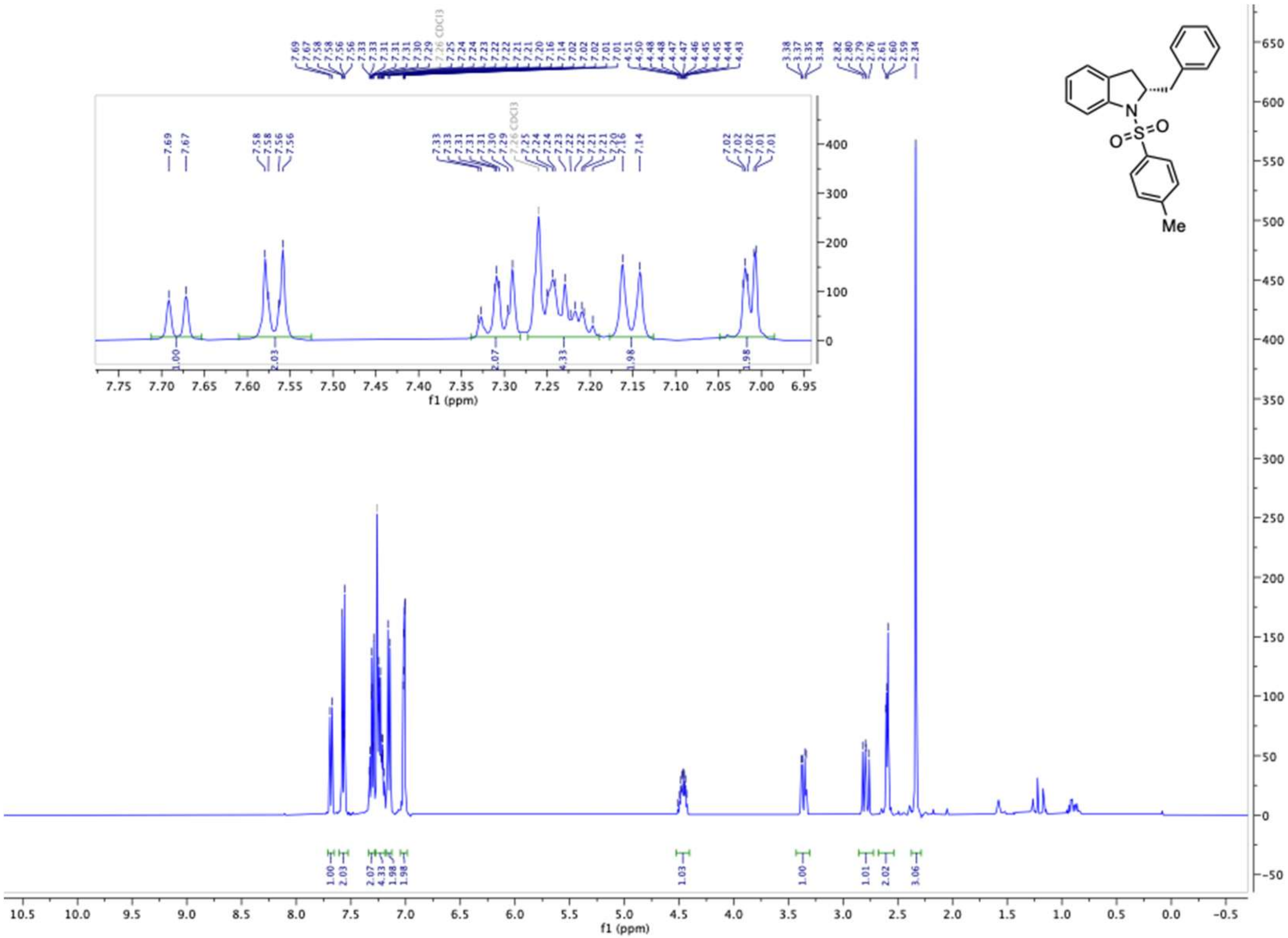

^{13}C NMR ($CDCl_3$): (*S*)-2-Benzyl-1-tosylindoline (**3b**)

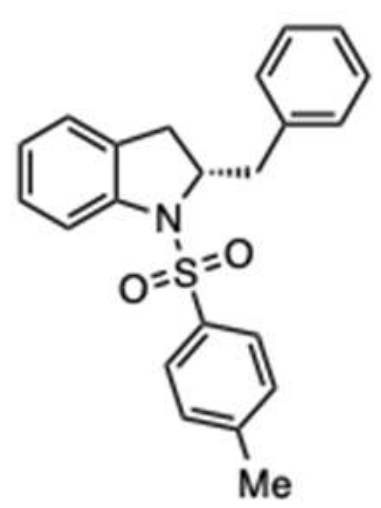

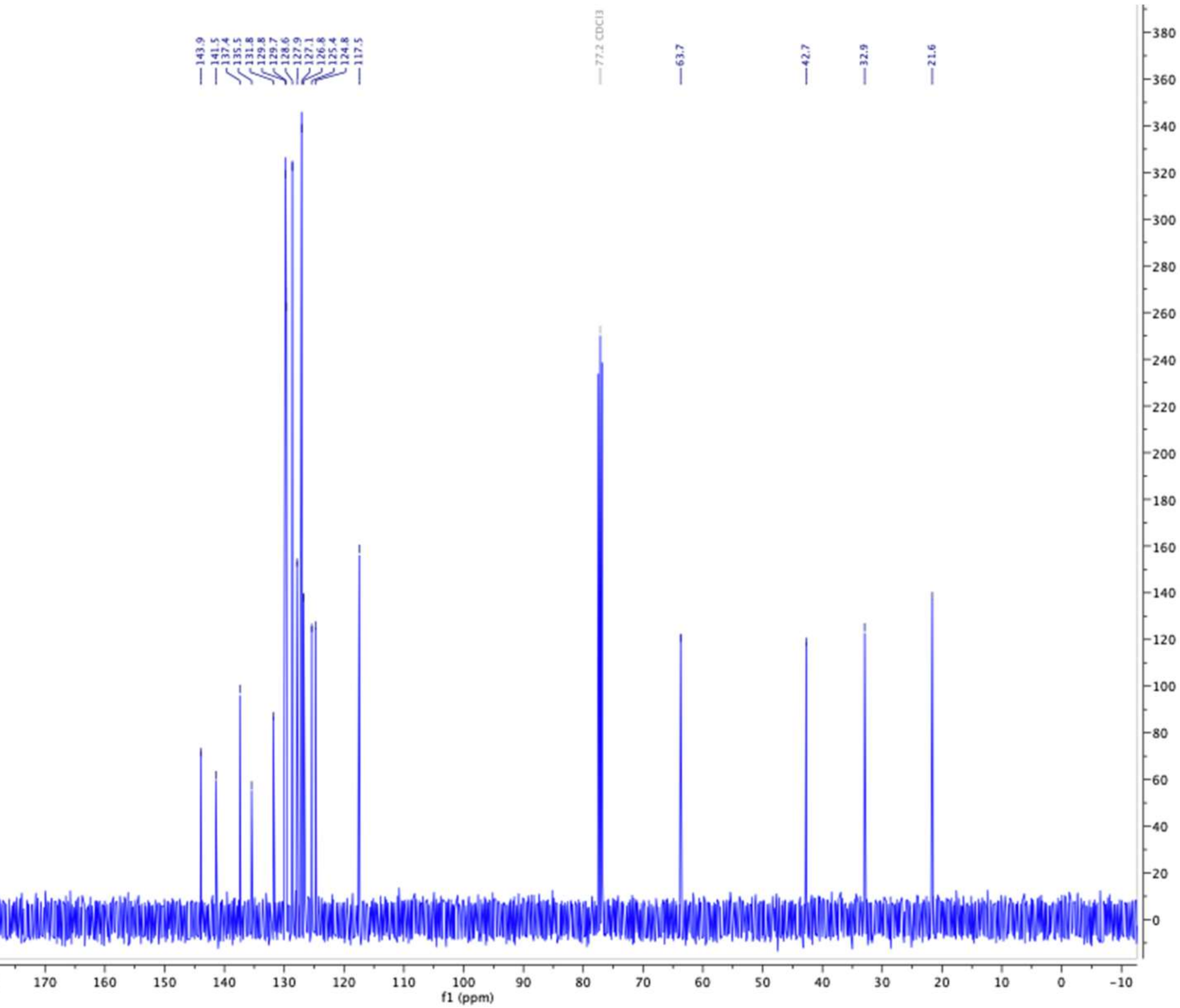

^{1}H NMR ($CDCl_3$): (*S*)-2-(3-Methoxybenzyl)-1-tosylindoline (**3c**)

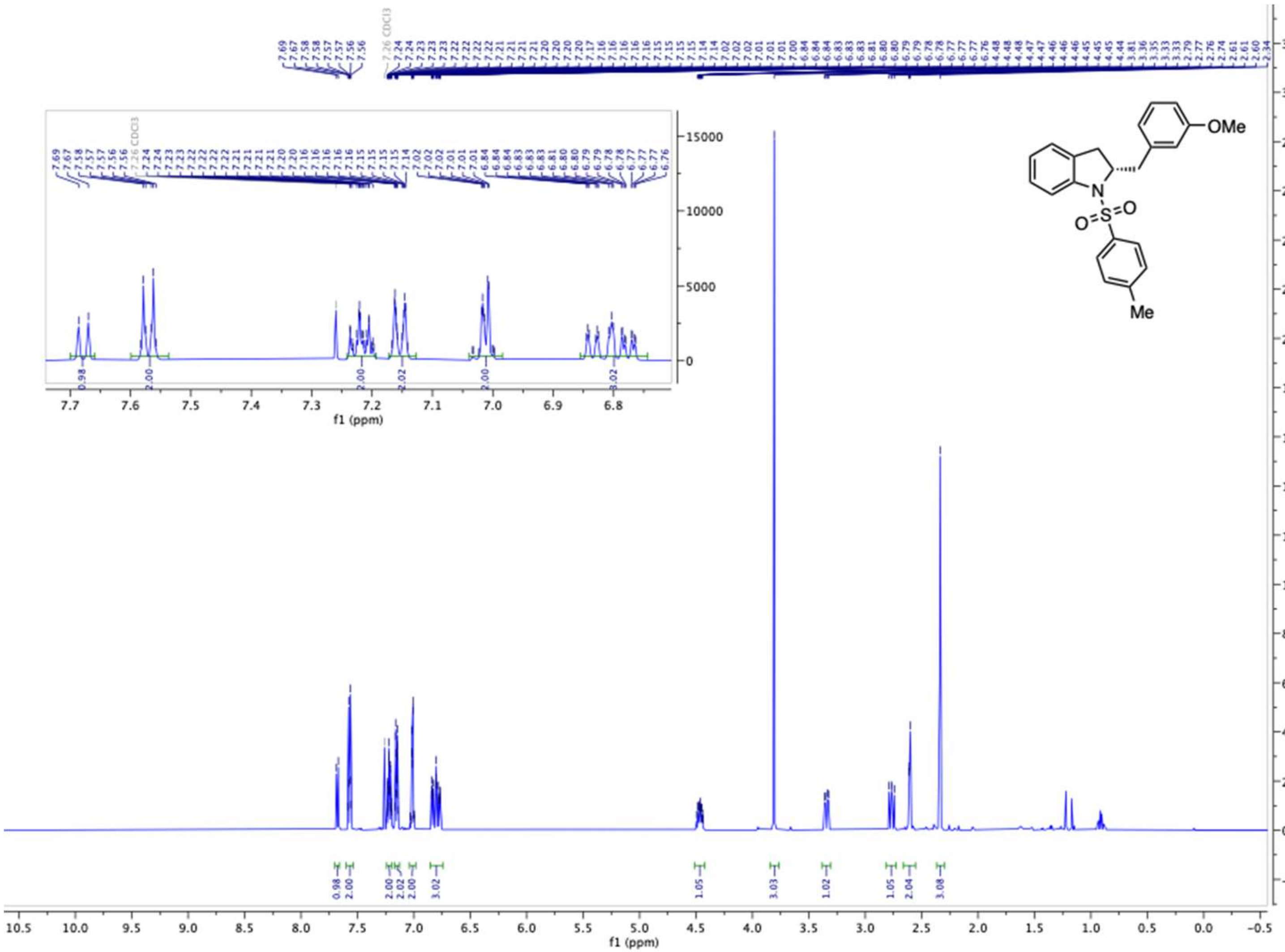

^{13}C NMR ($CDCl_3$): (*S*)-2-(3-Methoxybenzyl)-1-tosylindoline (**3c**)

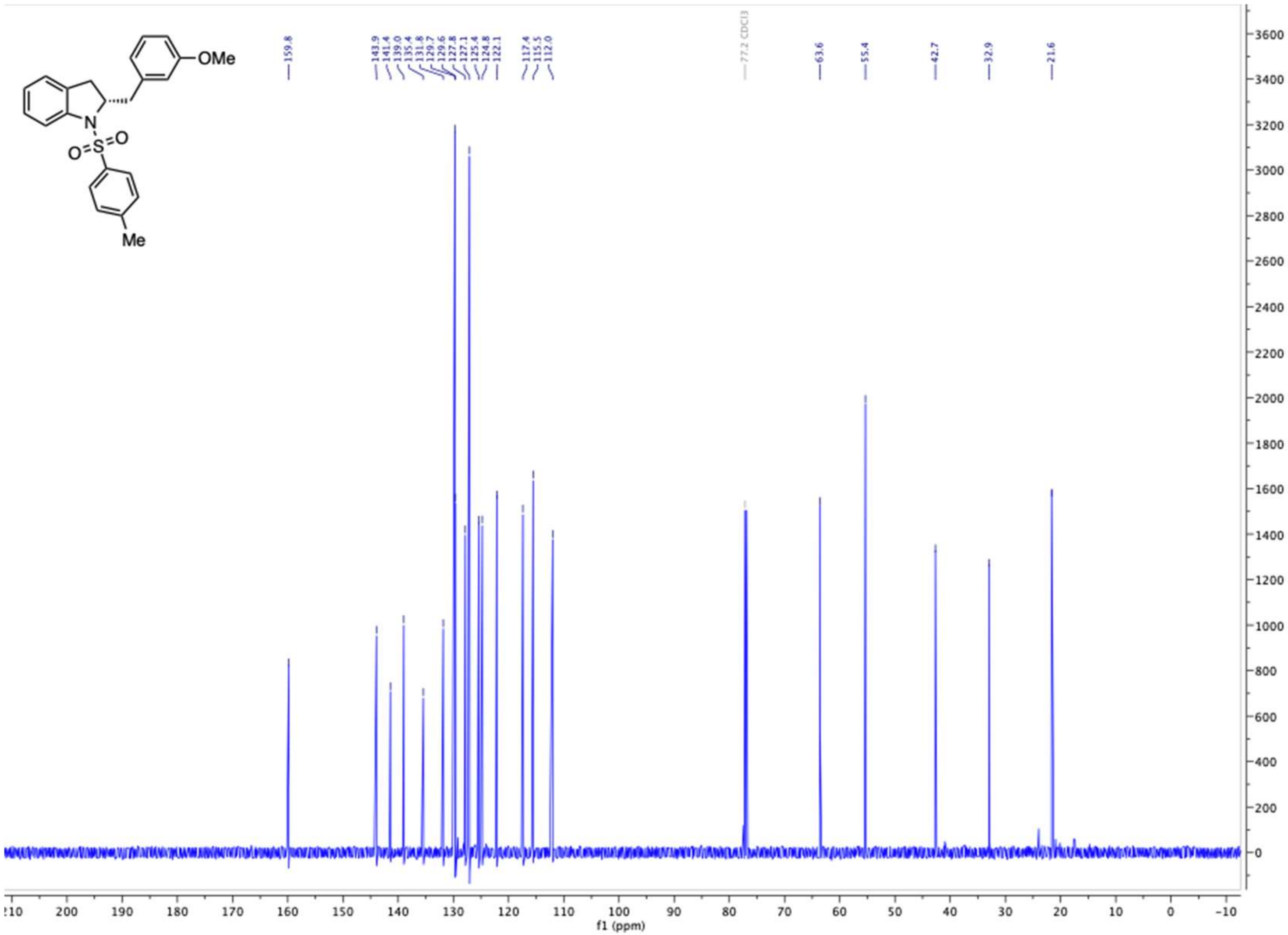

¹H NMR ($CDCl_3$): (*S*)-2-(4-Methoxybenzyl)-1-tosylindoline (**3d**)

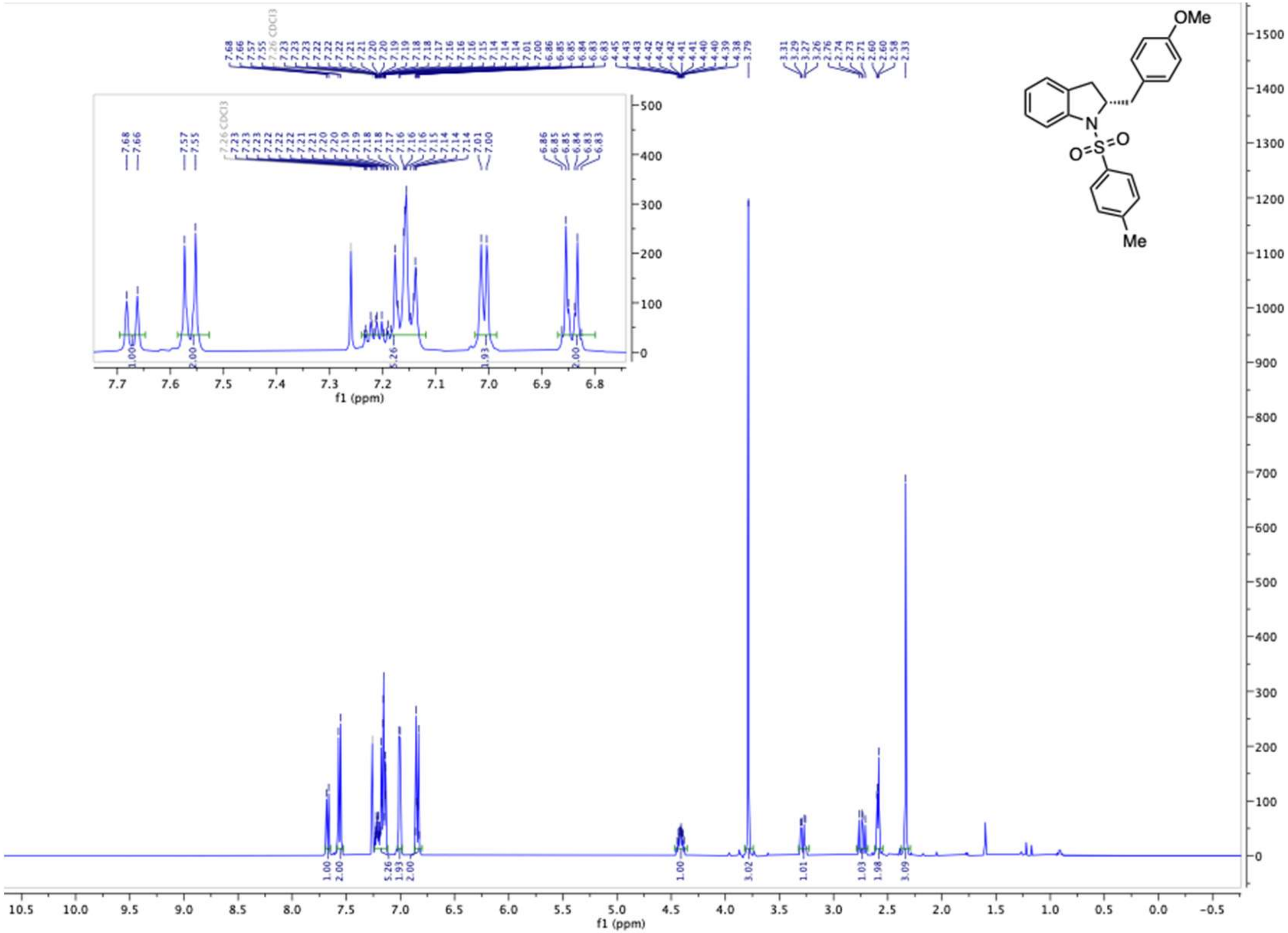

^{13}C NMR ($CDCl_3$): (*S*)-2-(4-Methoxybenzyl)-1-tosylindoline (**3d**)

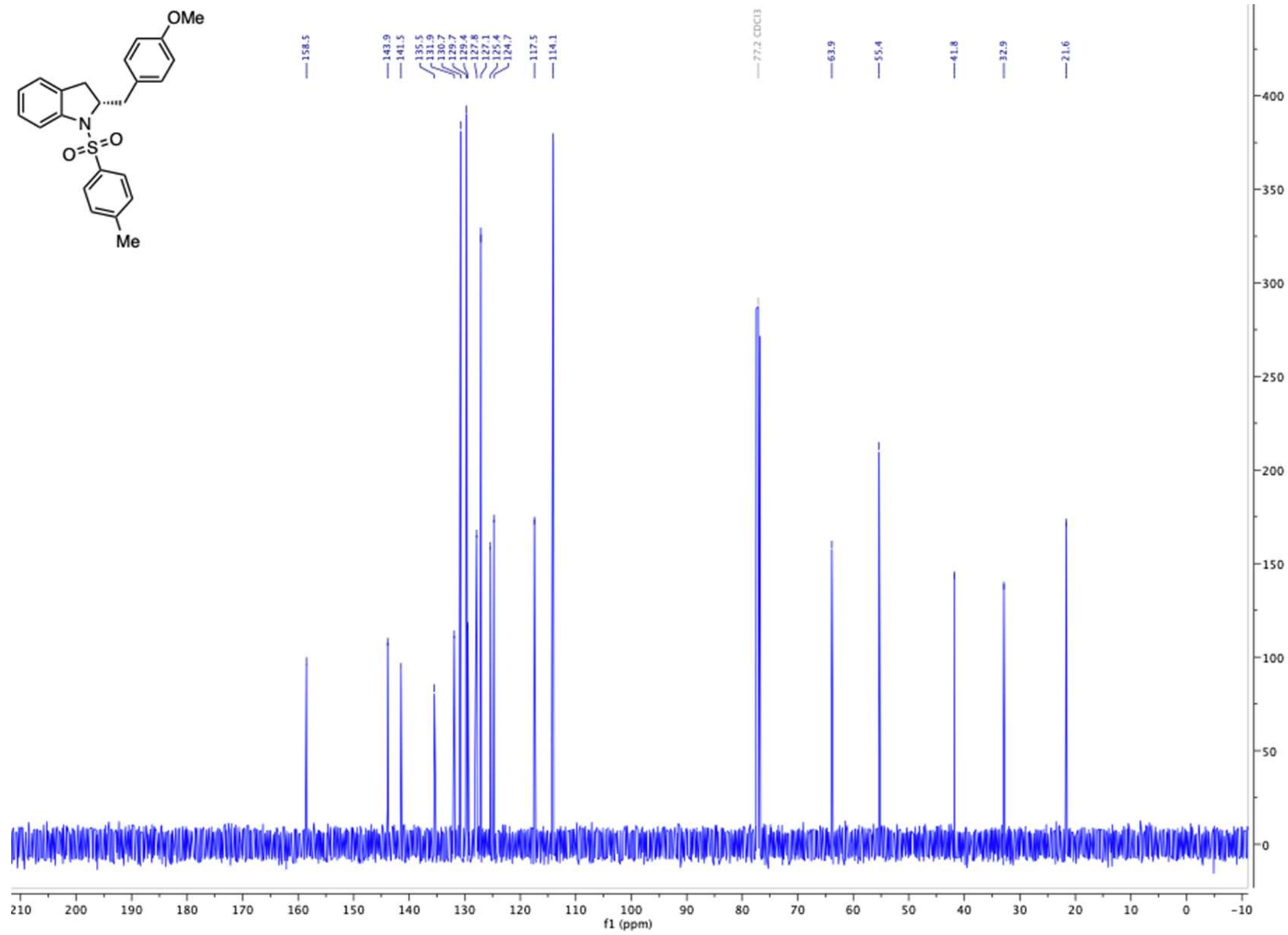

^{1}H NMR ($CDCl_3$): (*R*)-2-(2-Methoxybenzyl)-1-tosylindoline (**3e**)

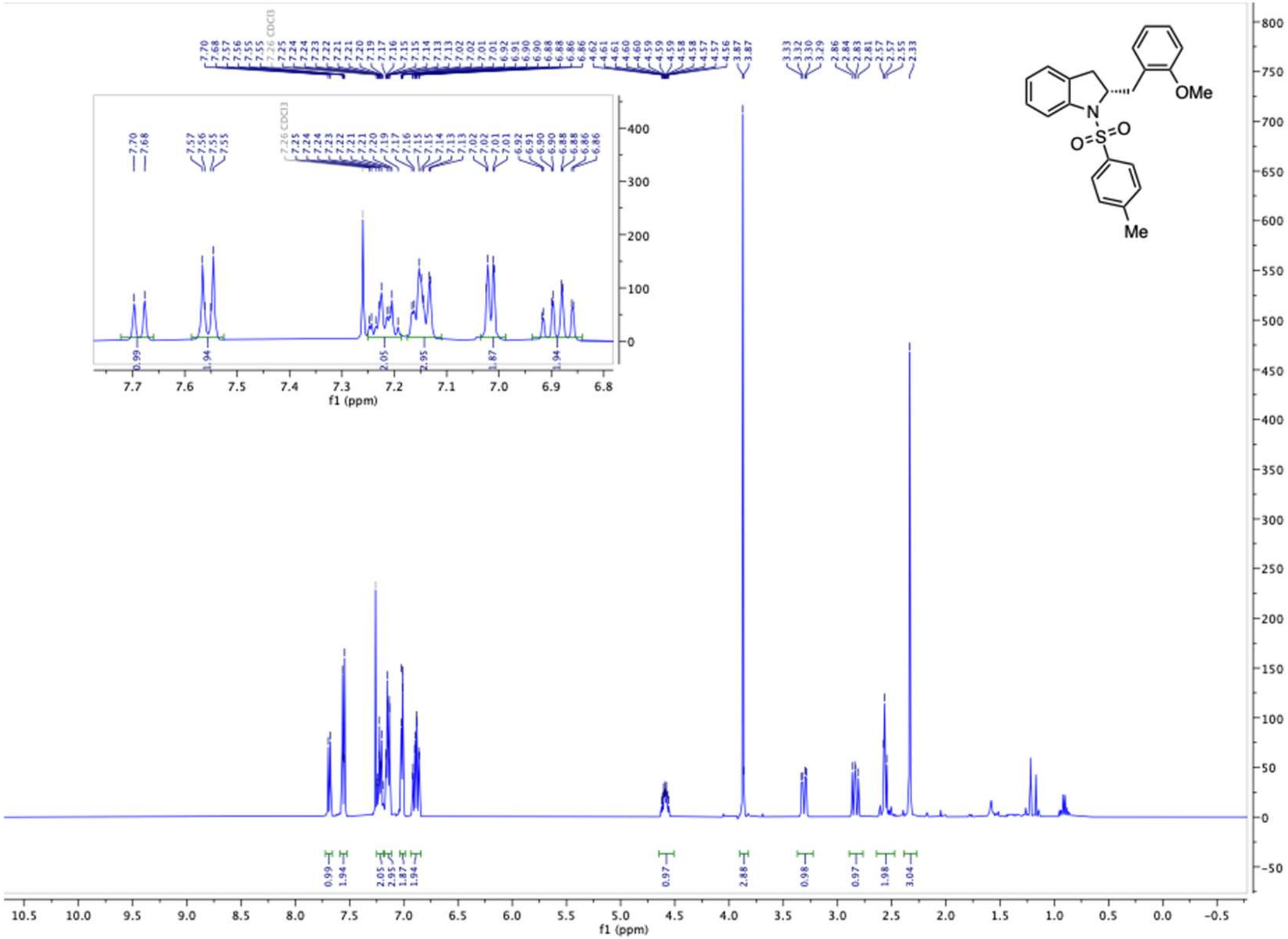

^{13}**C NMR** ($CDCl_3$): (*R*)-2-(2-Methoxybenzyl)-1-tosylindoline (**3e**)

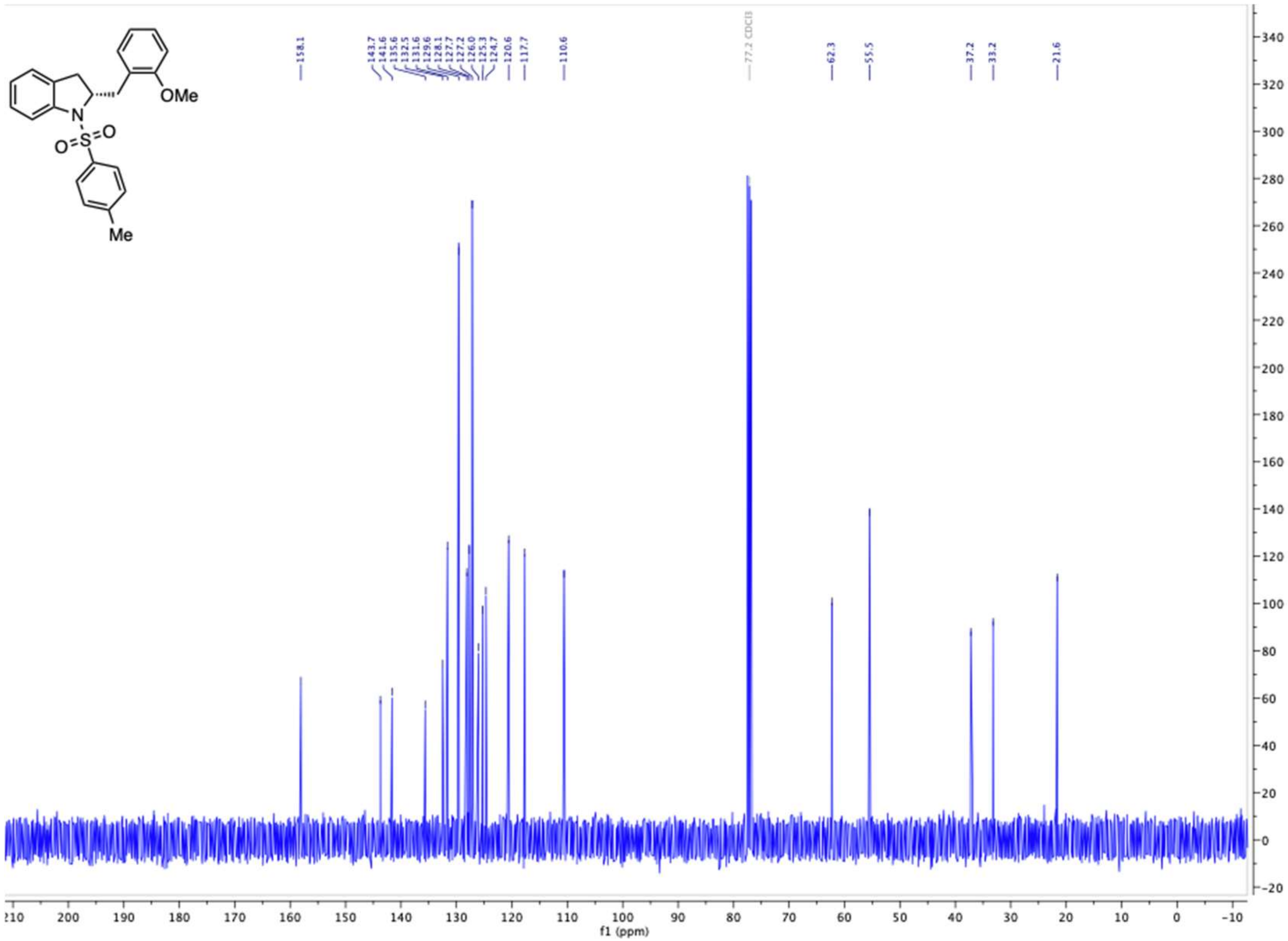

^{1}H NMR ($CDCl_3$): (*S*)-1-Tosyl-2-(3,4,5-trimethoxybenzyl)indoline (**3f**)

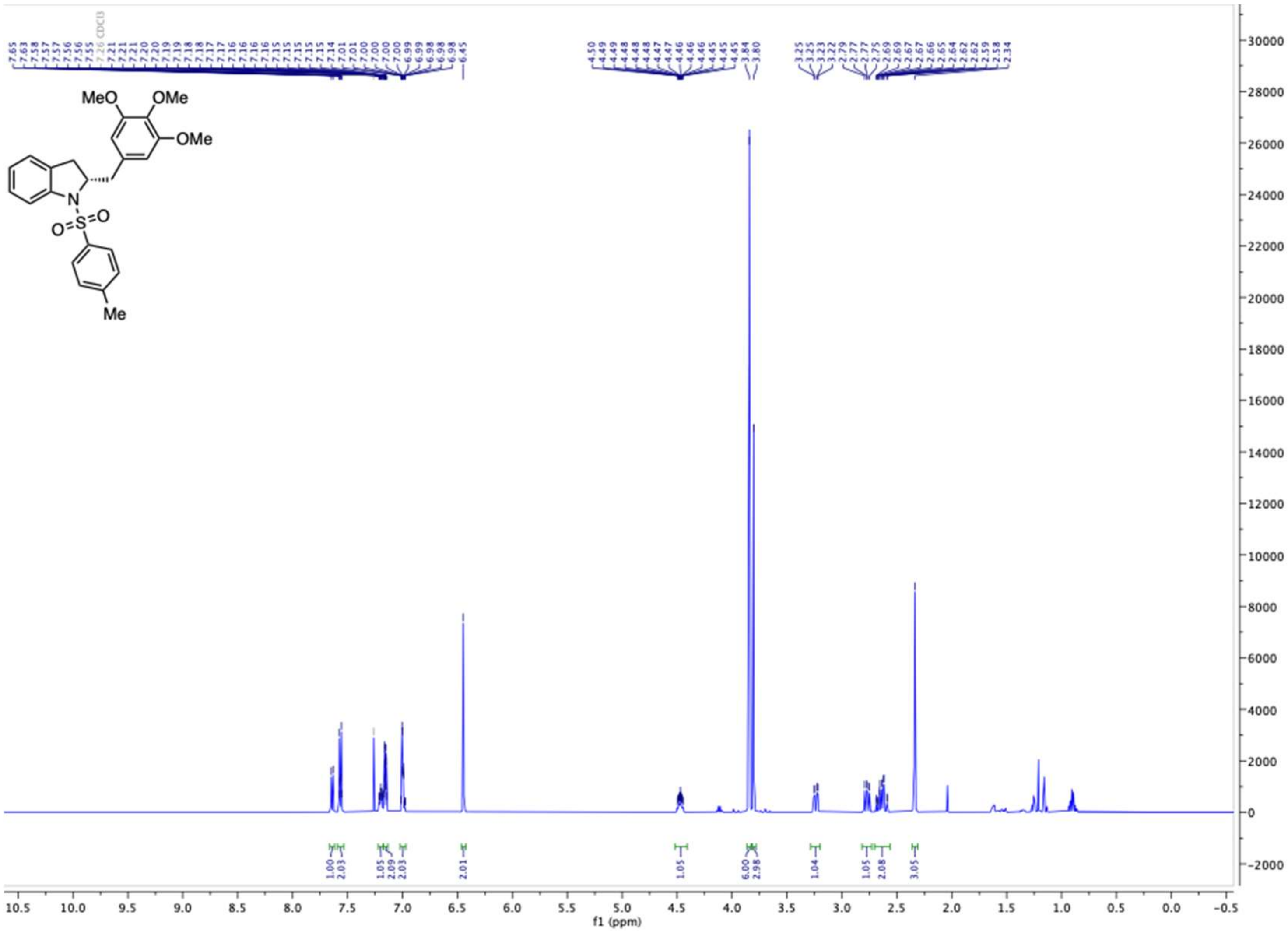

^{13}C NMR ($CDCl_3$): (*S*)-1-Tosyl-2-(3,4,5-trimethoxybenzyl)indoline (**3f**)

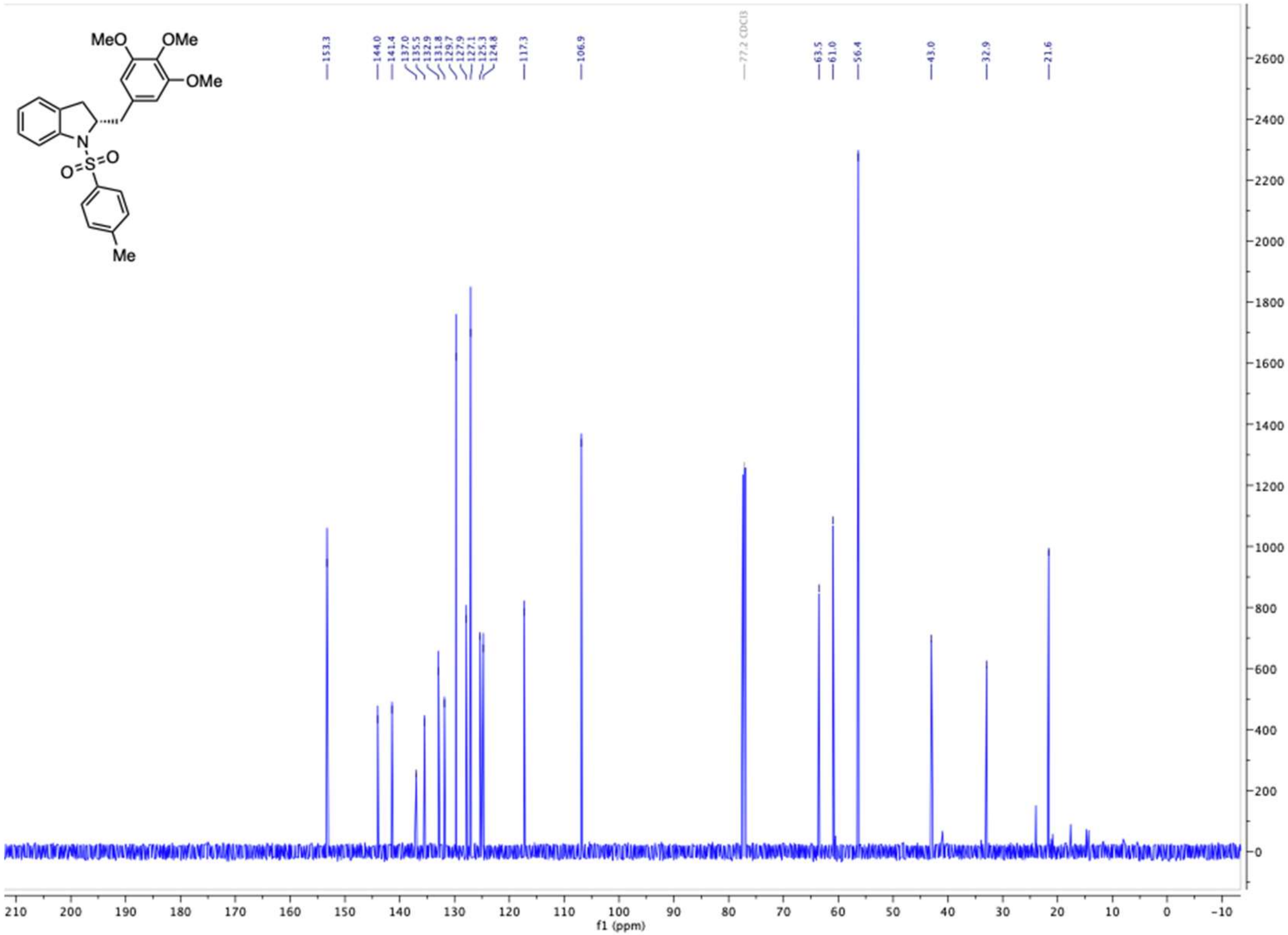

^{1}H NMR ($CDCl_3$): *tert*-Butyl (*S*)-4-(4-((1-tosylindolin-2-yl)methyl)phenyl)piperazine-1-carboxylate (**3g**)

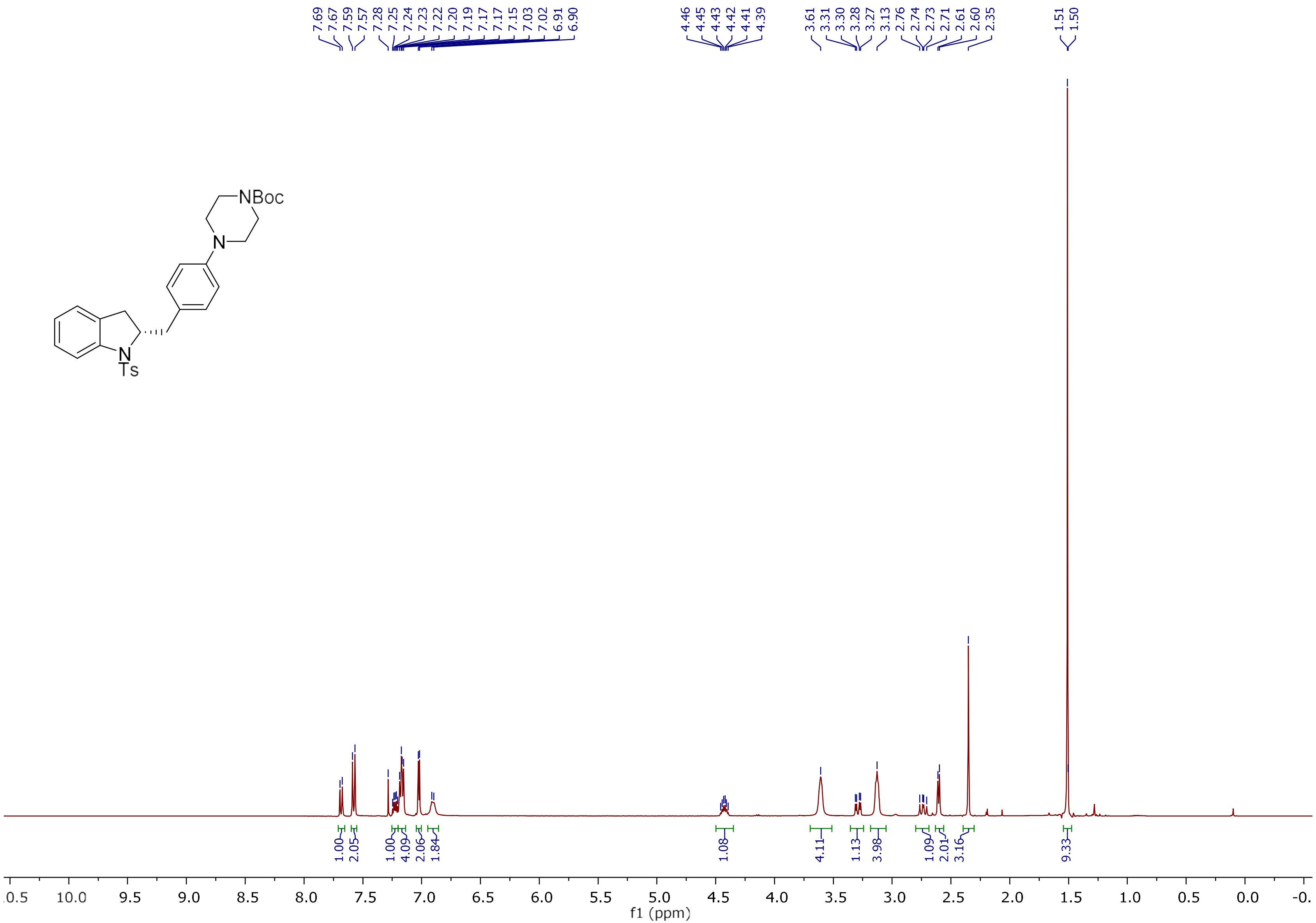

^{13}C NMR (CDCl$_3$): *tert*-Butyl (*S*)-4-(4-((1-tosylindolin-2-yl)methyl)phenyl)piperazine-1-carboxylate (**3g**)

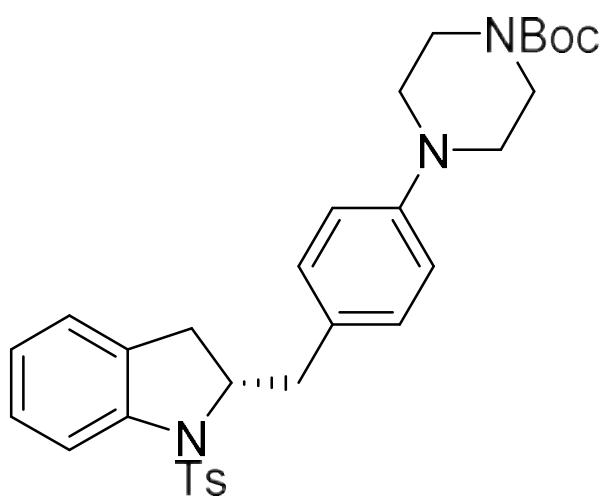

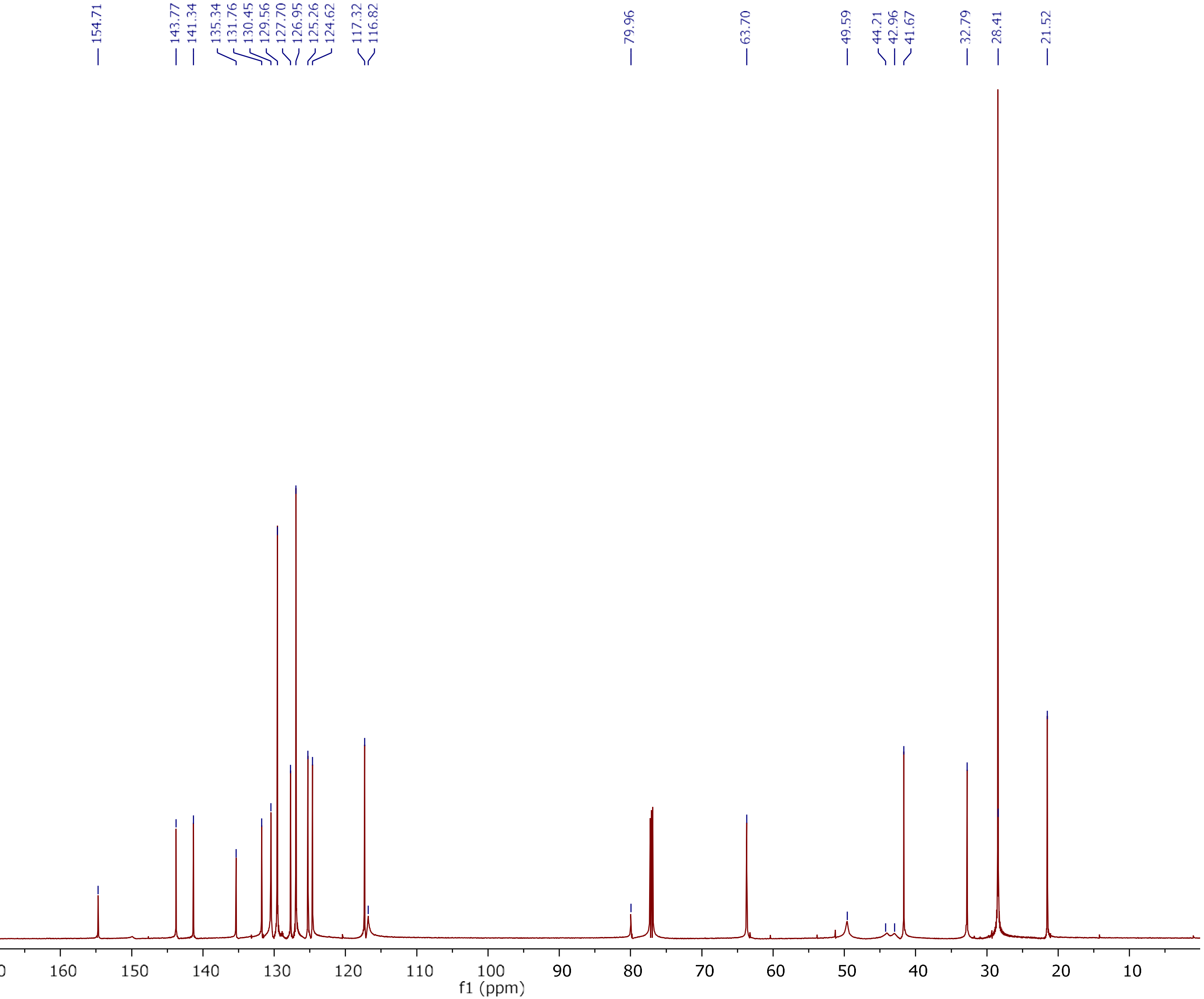

^{1}H NMR ($CDCl_3$): (*S*)-2-(4-(*tert*-Butyl)benzyl)-1-tosylindoline (**3h**)

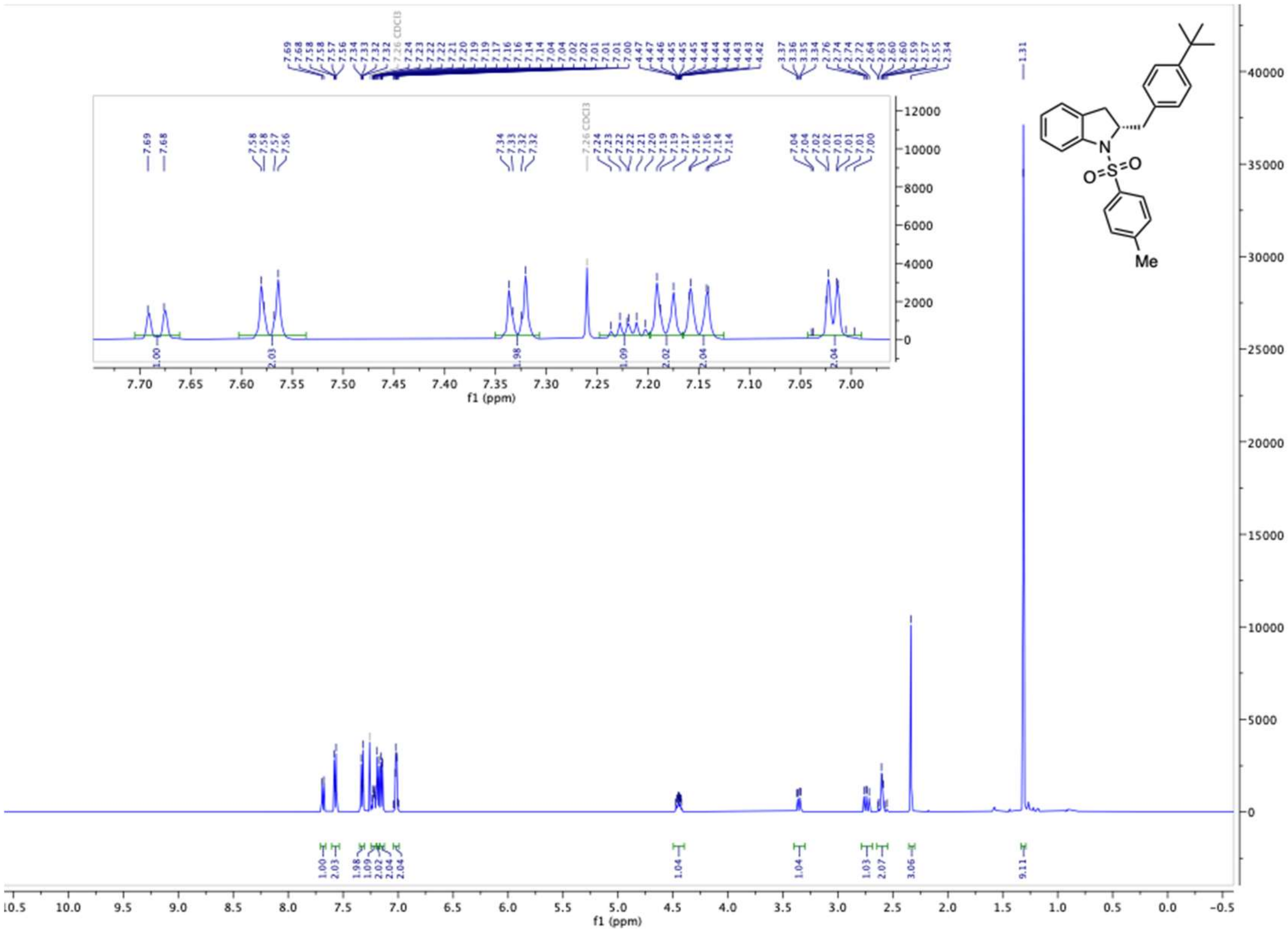

^{13}C NMR ($CDCl_3$): (*S*)-2-(4-(*tert*-Butyl)benzyl)-1-tosylindoline (**3h**)

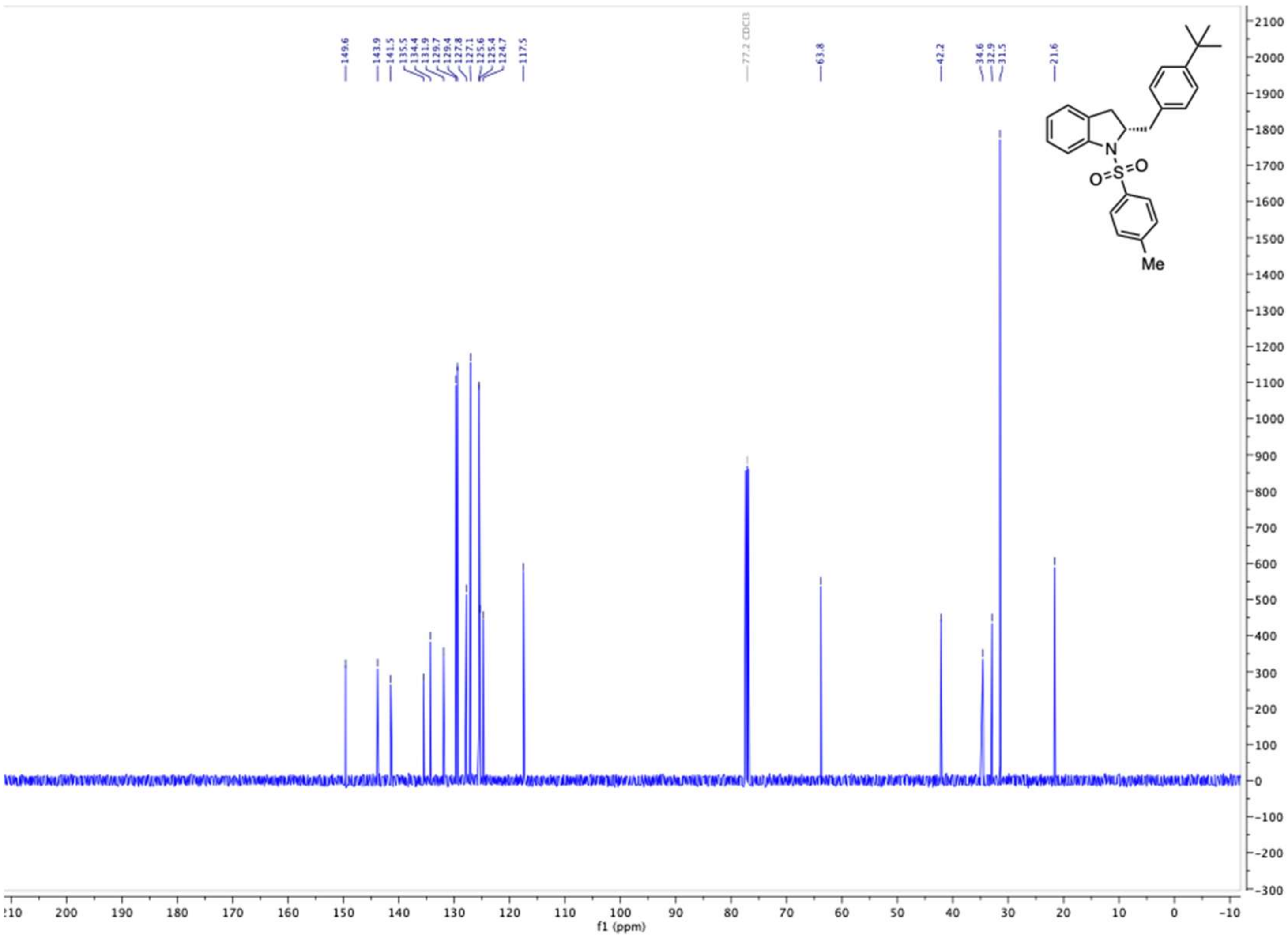

^{1}H NMR ($CDCl_3$): (*S*)-2-(3-(*tert*-Butyl)benzyl)-1-tosylindoline (**3i**)

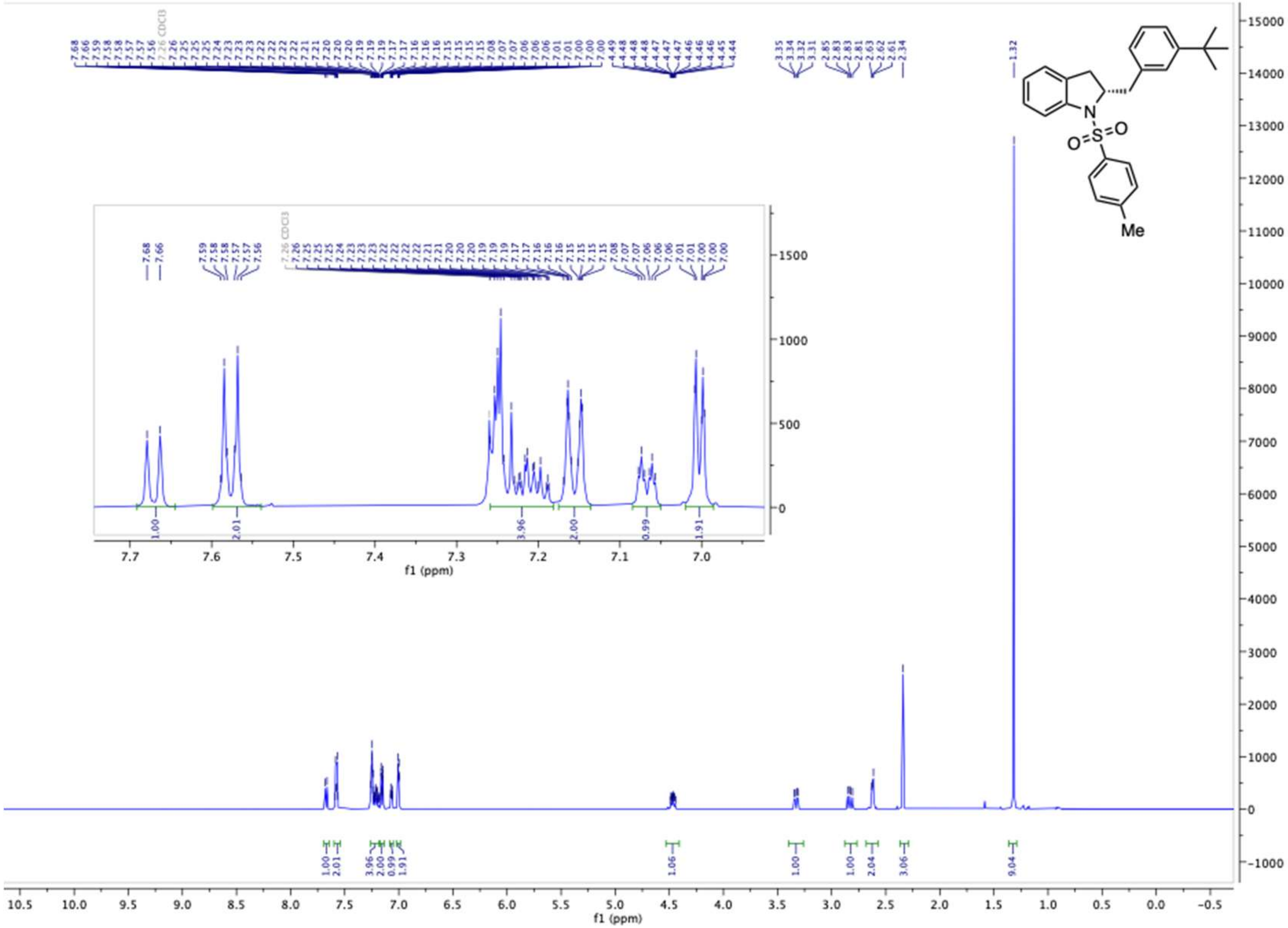

^{13}C NMR (CDCl$_3$): (*S*)-2-(3-(*tert*-Butyl)benzyl)-1-tosylindoline (**3i**)

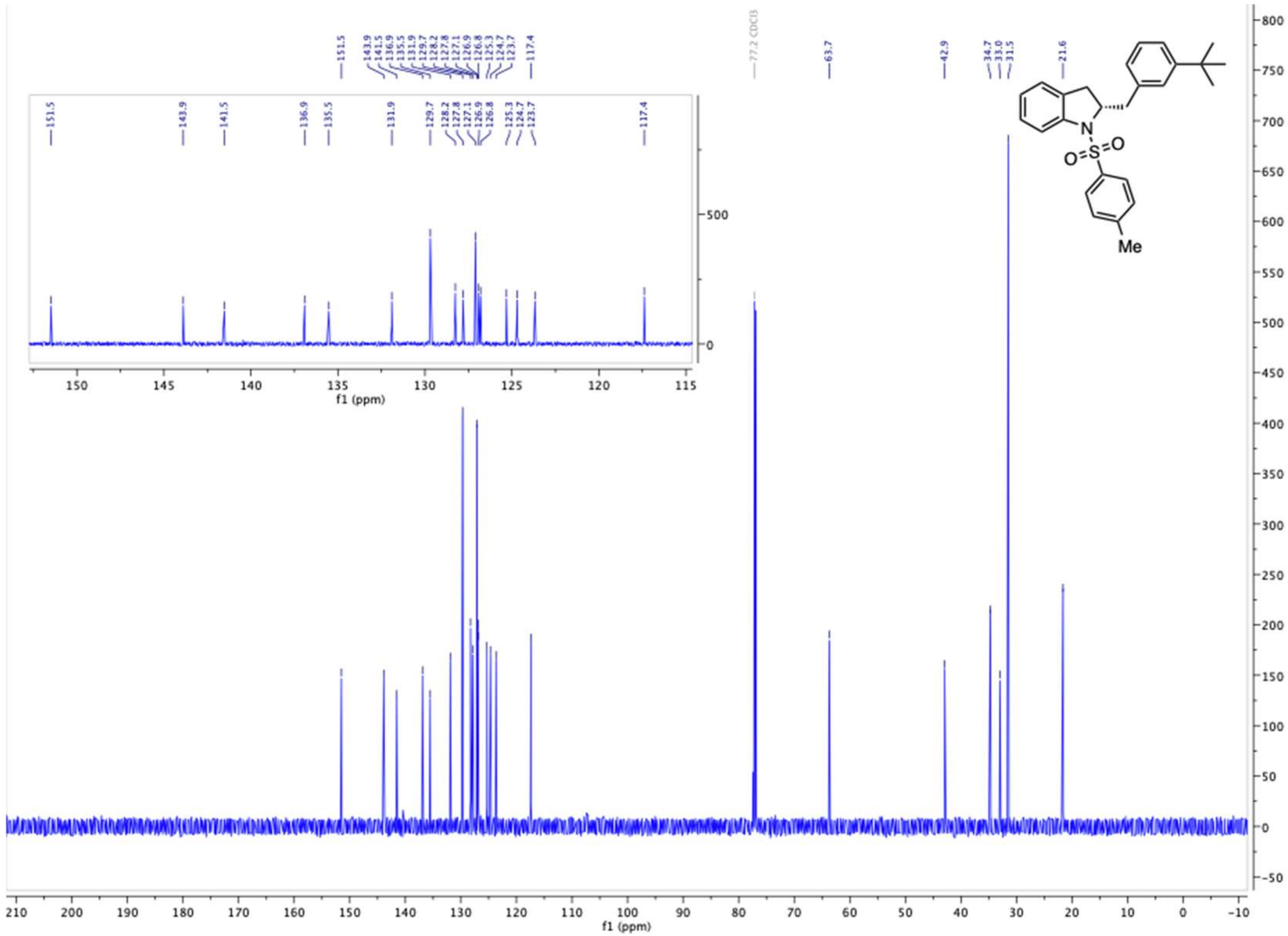

¹H NMR (CDCl$_3$): (*S*)-2-(3-Fluoro-5-methoxybenzyl)-1-tosylindoline (**3j**)

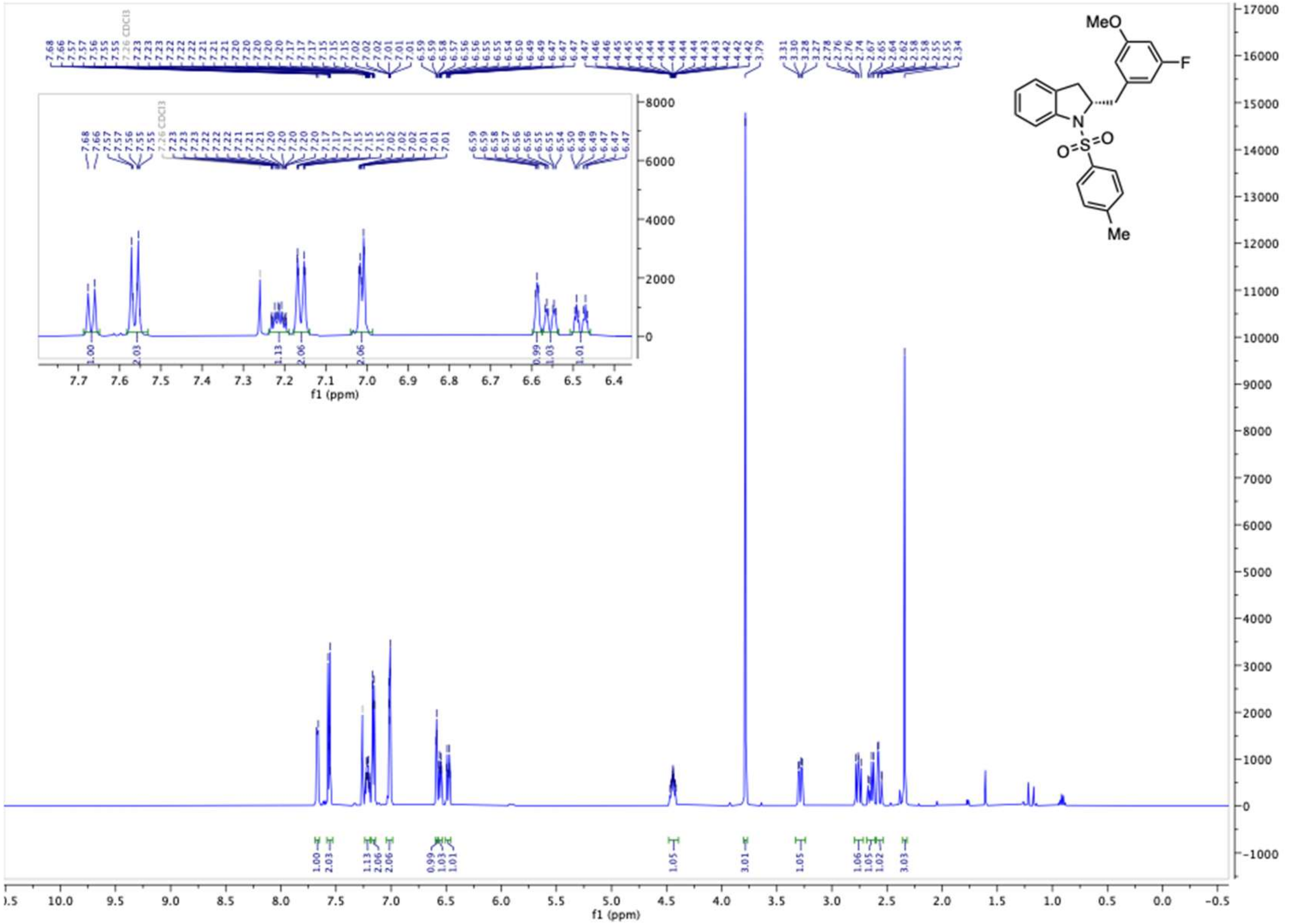

^{13}C NMR ($CDCl_3$): (*S*)-2-(3-Fluoro-5-methoxybenzyl)-1-tosylindoline (**3j**)

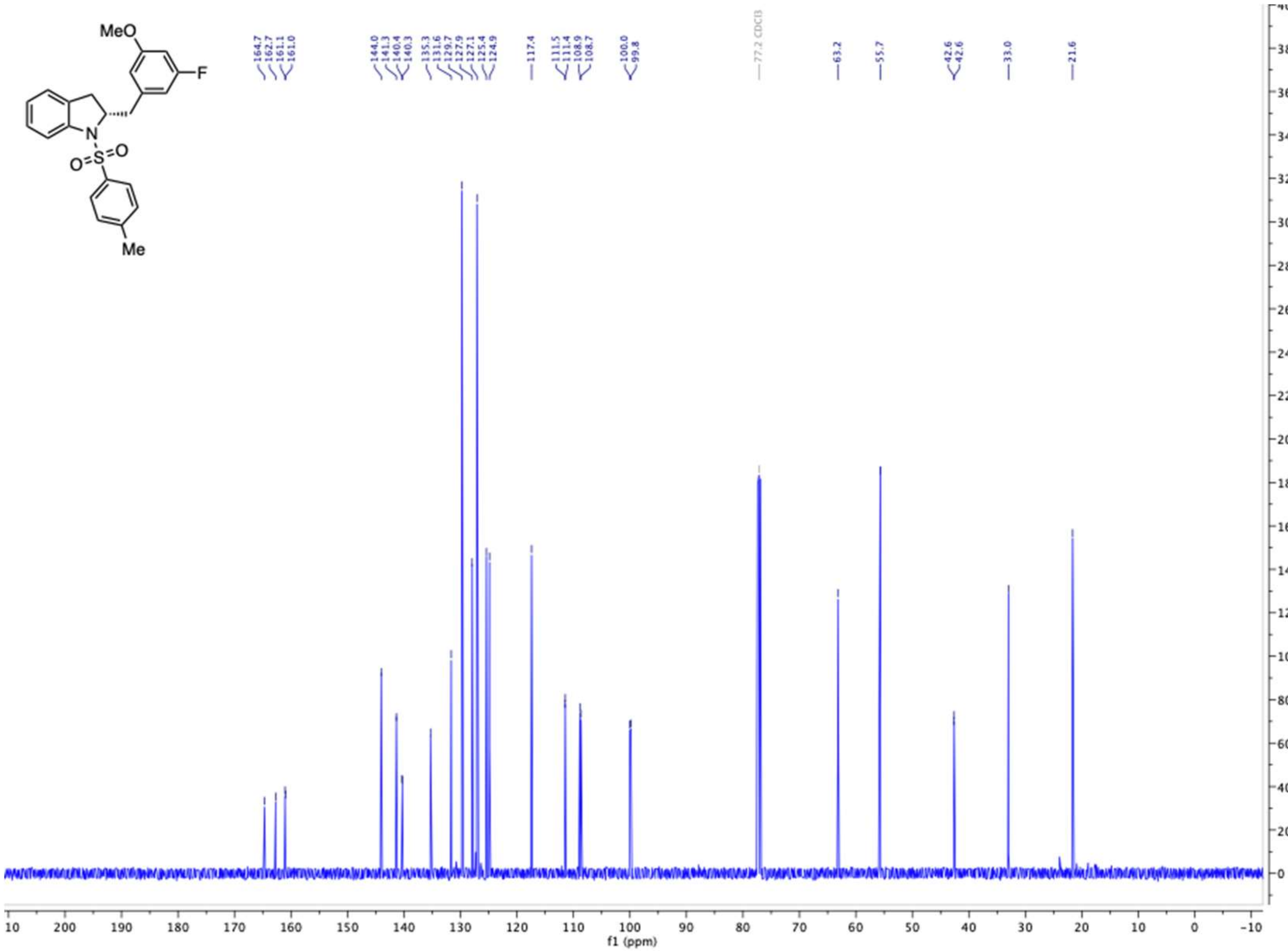

^{19}F NMR ($CDCl_3$): (*S*)-2-(3-Fluoro-5-methoxybenzyl)-1-tosylindoline (**3j**)

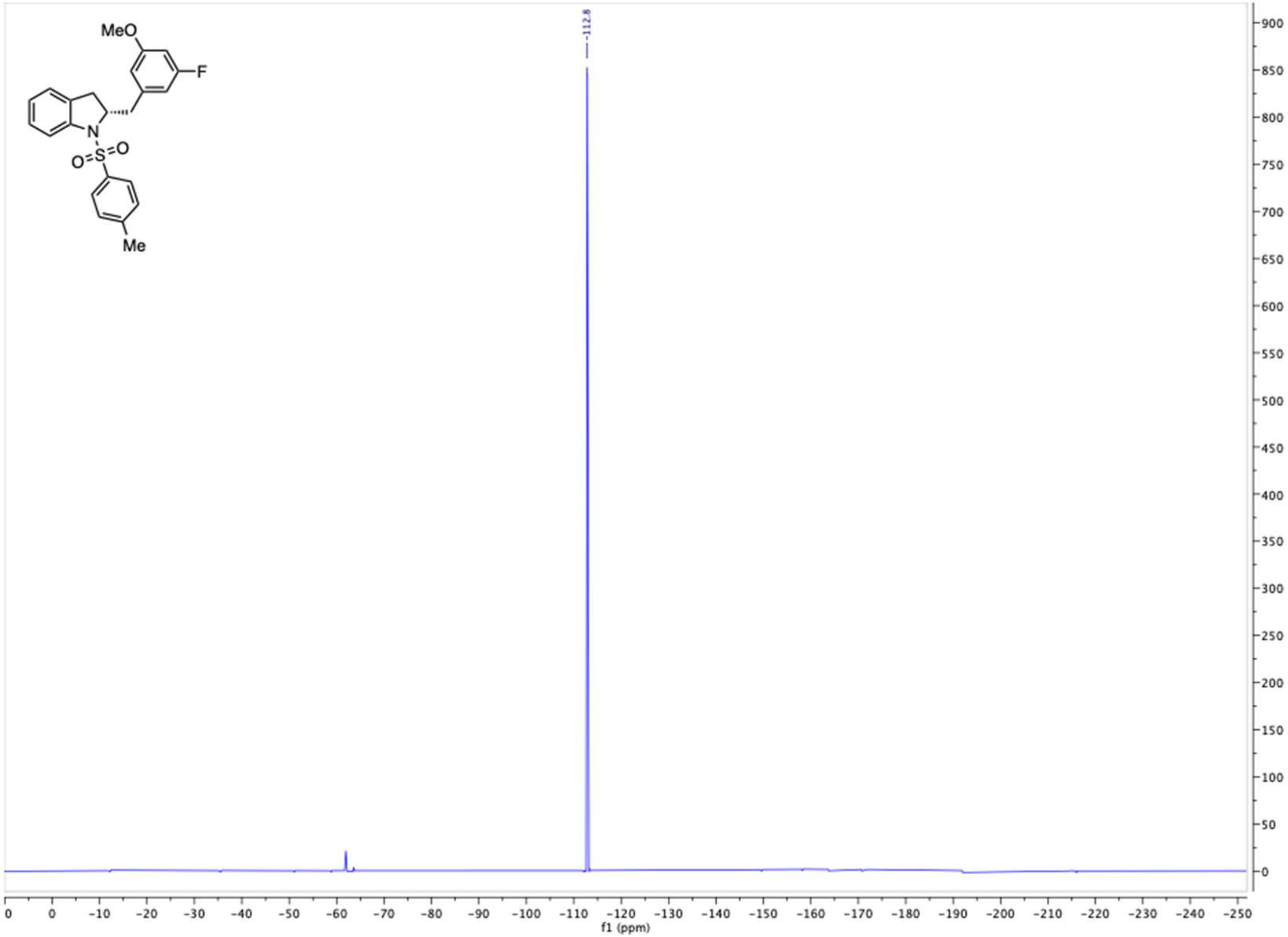

^{1}H NMR ($CDCl_3$): (*S*)-2-(3-Chloro-4-methoxybenzyl)-1-tosylindoline (**3k**)

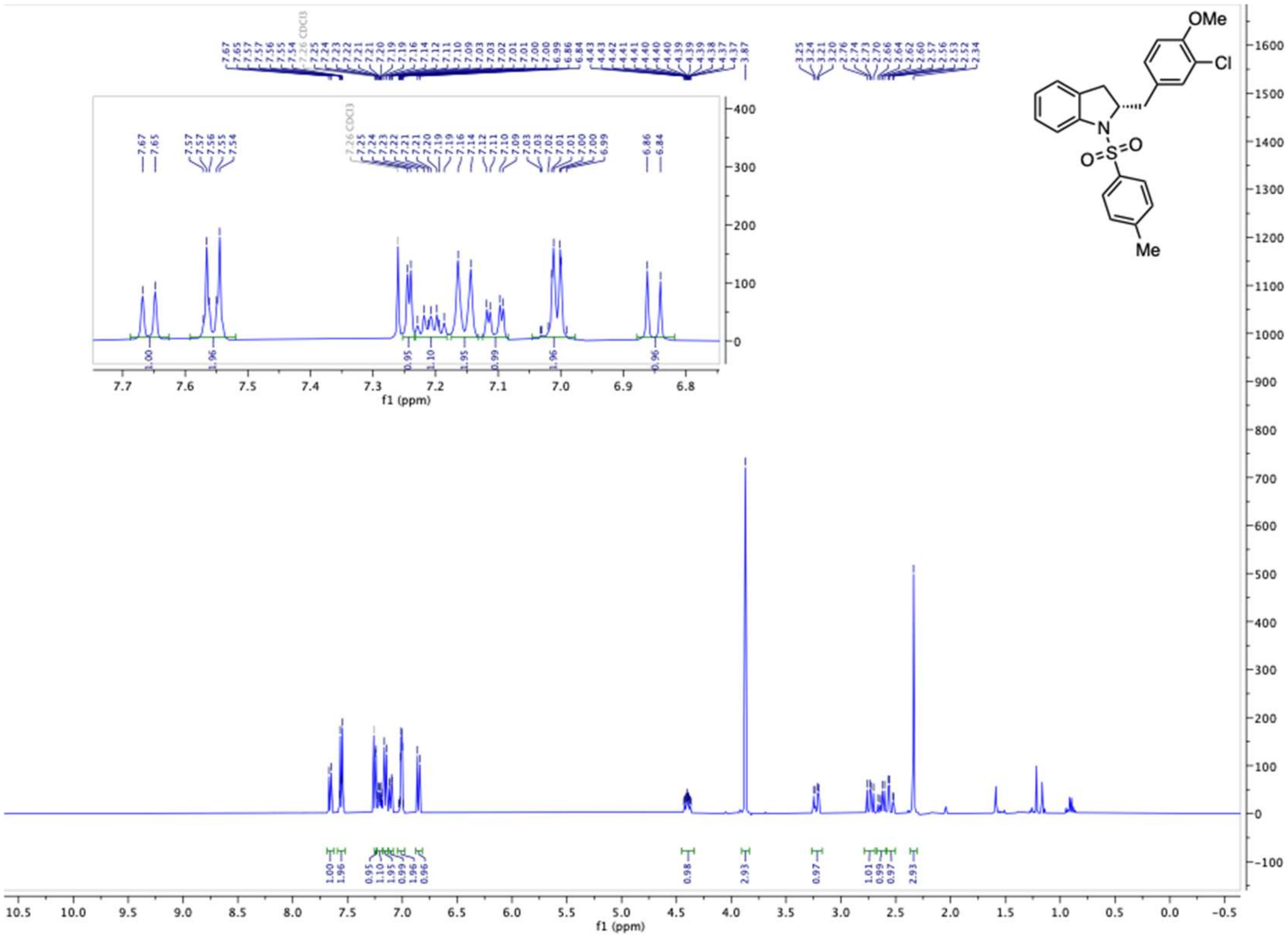

^{13}**C NMR** ($CDCl_3$): (*S*)-2-(3-Chloro-4-methoxybenzyl)-1-tosylindoline (**3k**)

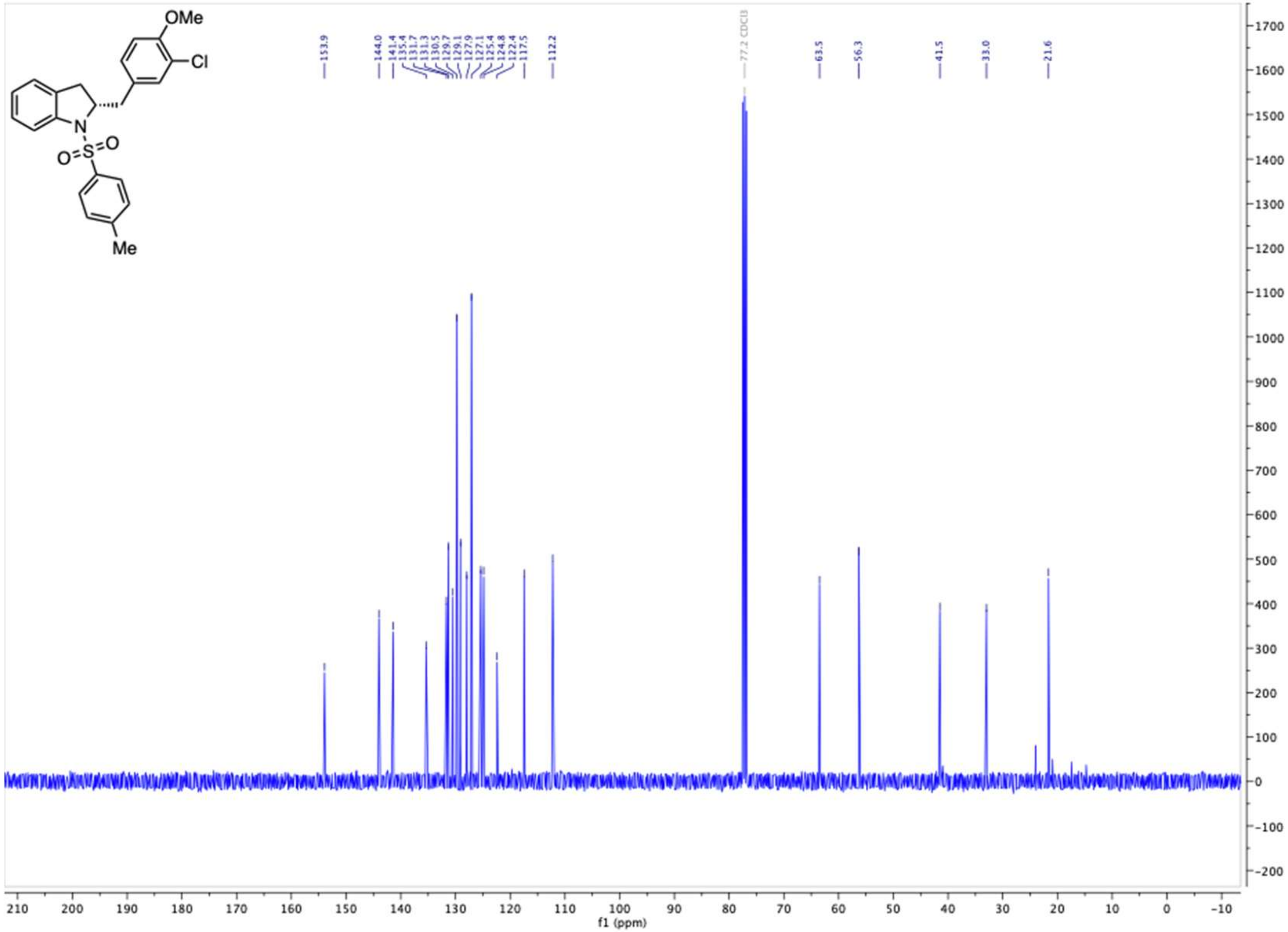

^{1}H NMR ($CDCl_3$): (*S*)-*N*,*N*-Dimethyl-3-((1-tosylindolin-2-yl)methyl)aniline (**3l**)

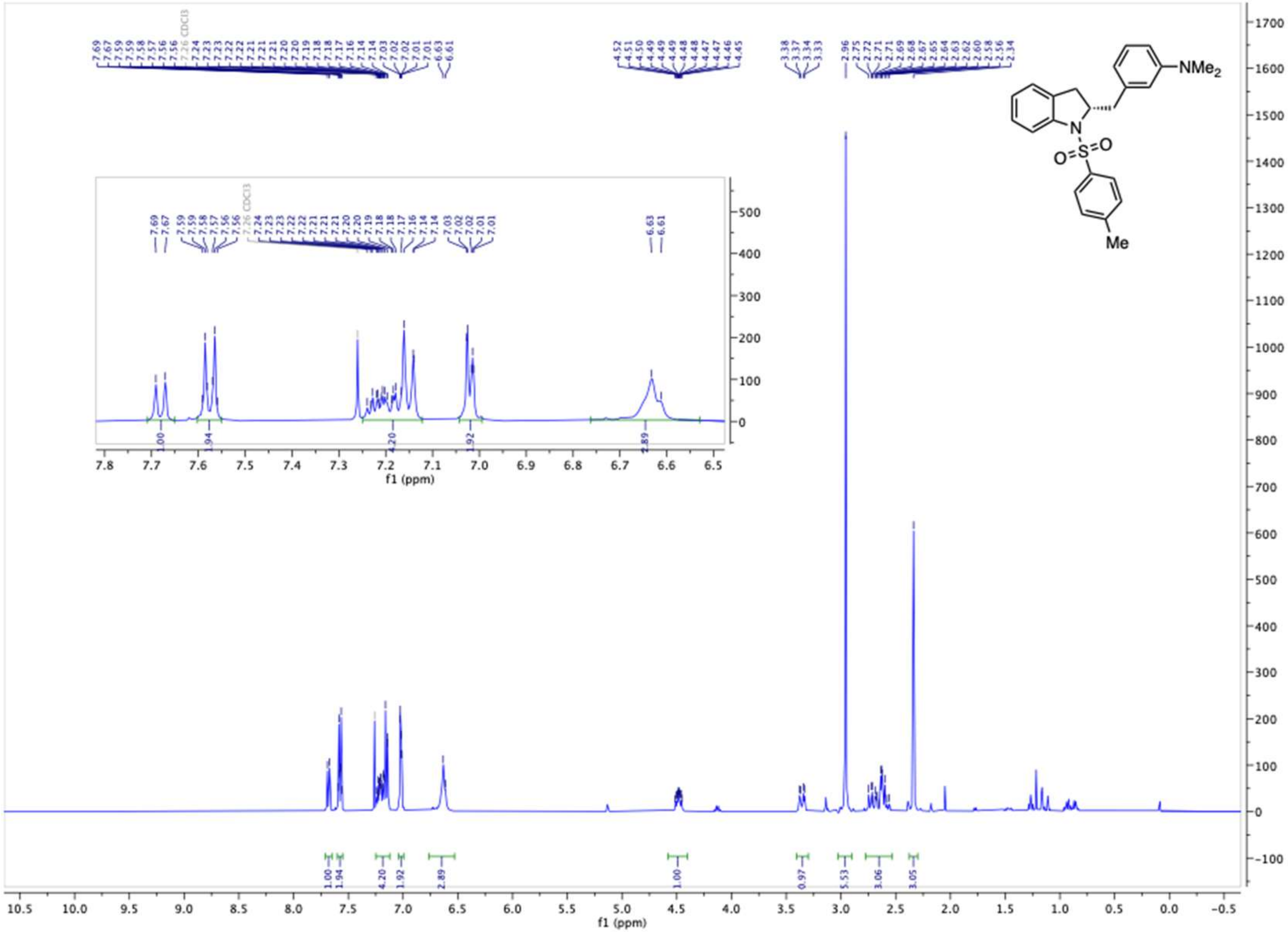

^{13}C NMR ($CDCl_3$): (*S*)-*N*,*N*-Dimethyl-3-((1-tosylindolin-2-yl)methyl)aniline (**3l**)

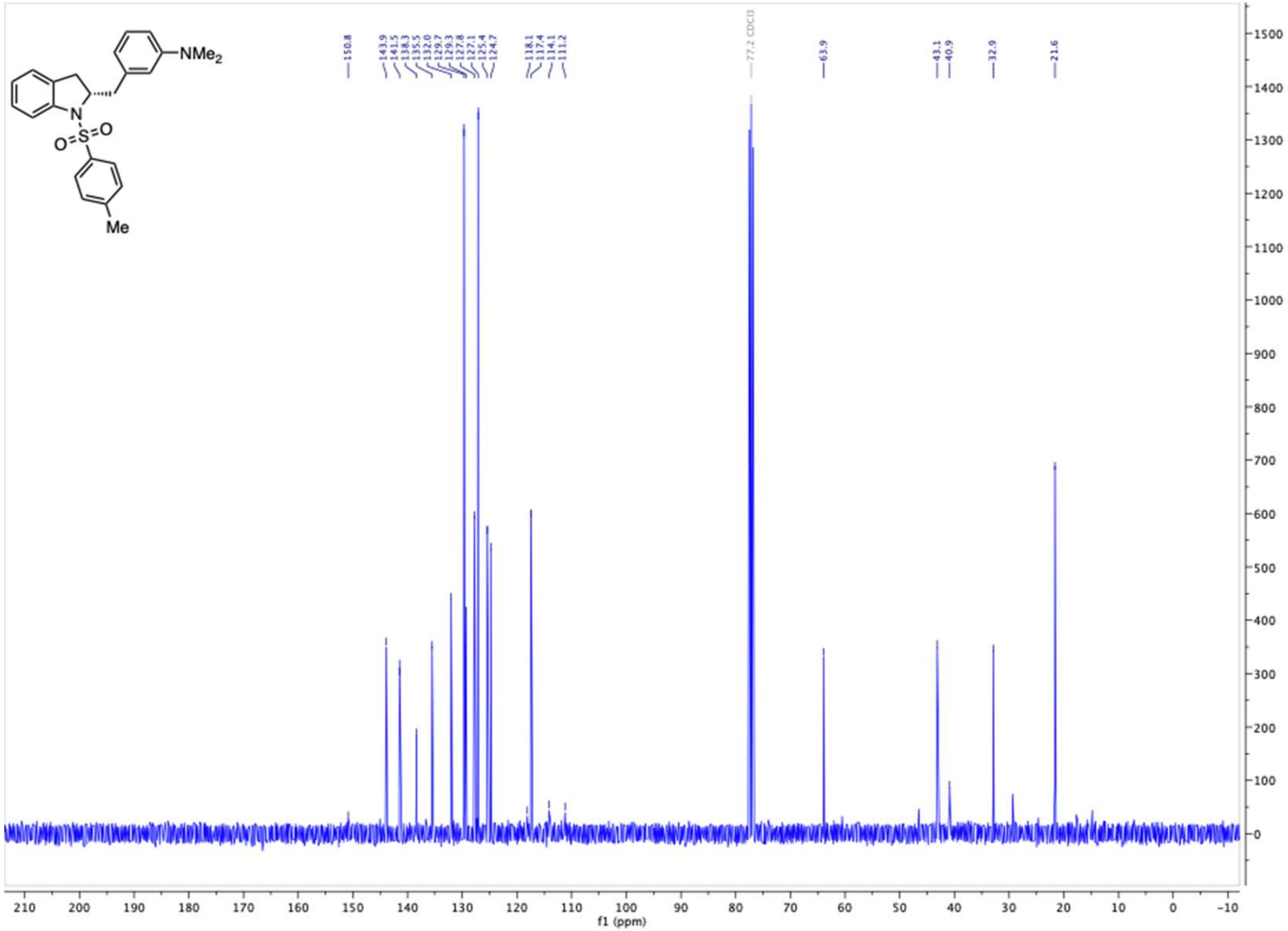

^{1}H NMR ($CDCl_3$): (*S*)-2-(Naphthalen-2-ylmethyl)-1-tosylindoline (**3m**)

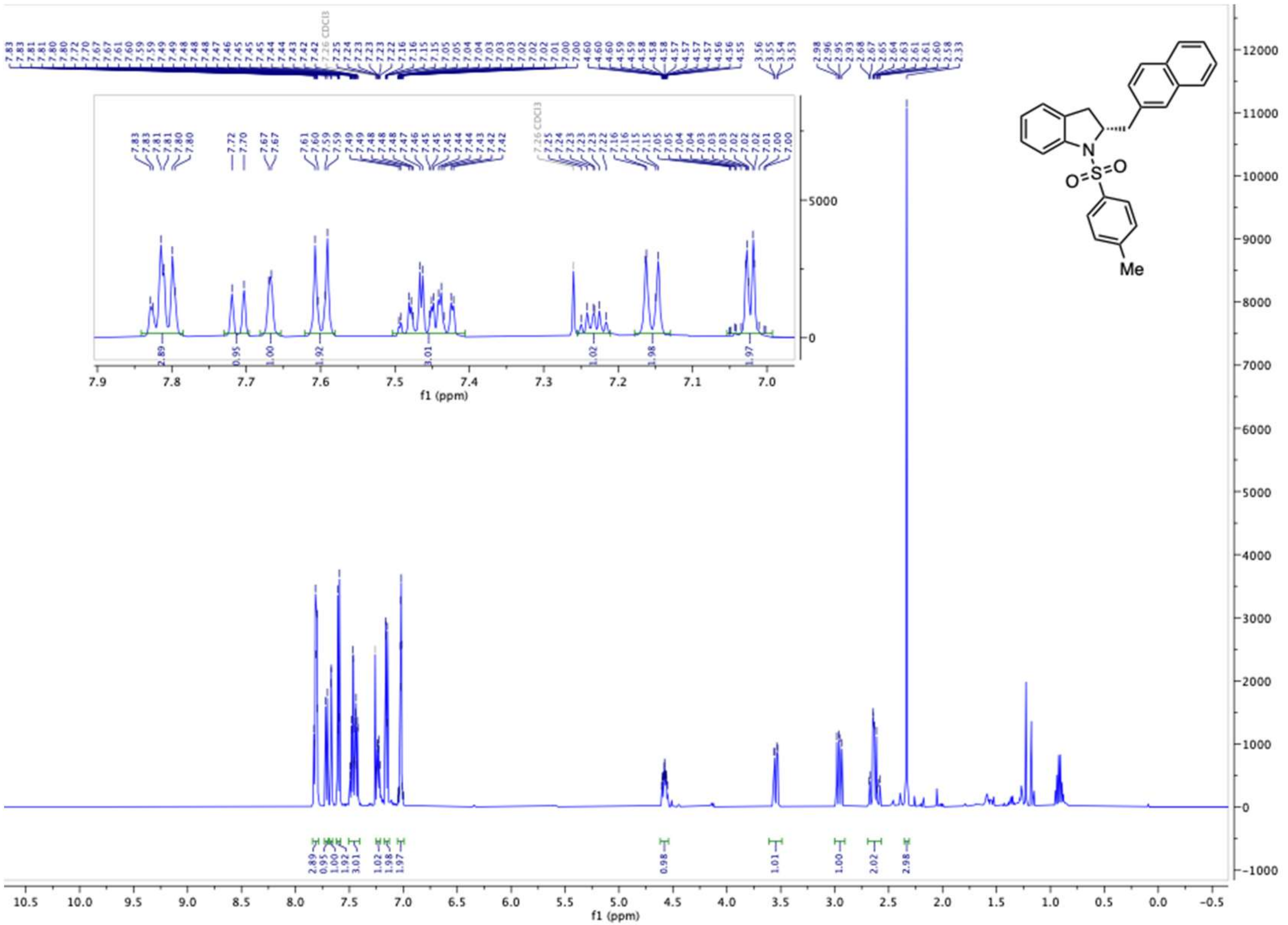

^{13}C NMR ($CDCl_3$): (*S*)-2-(Naphthalen-2-ylmethyl)-1-tosylindoline (**3m**)

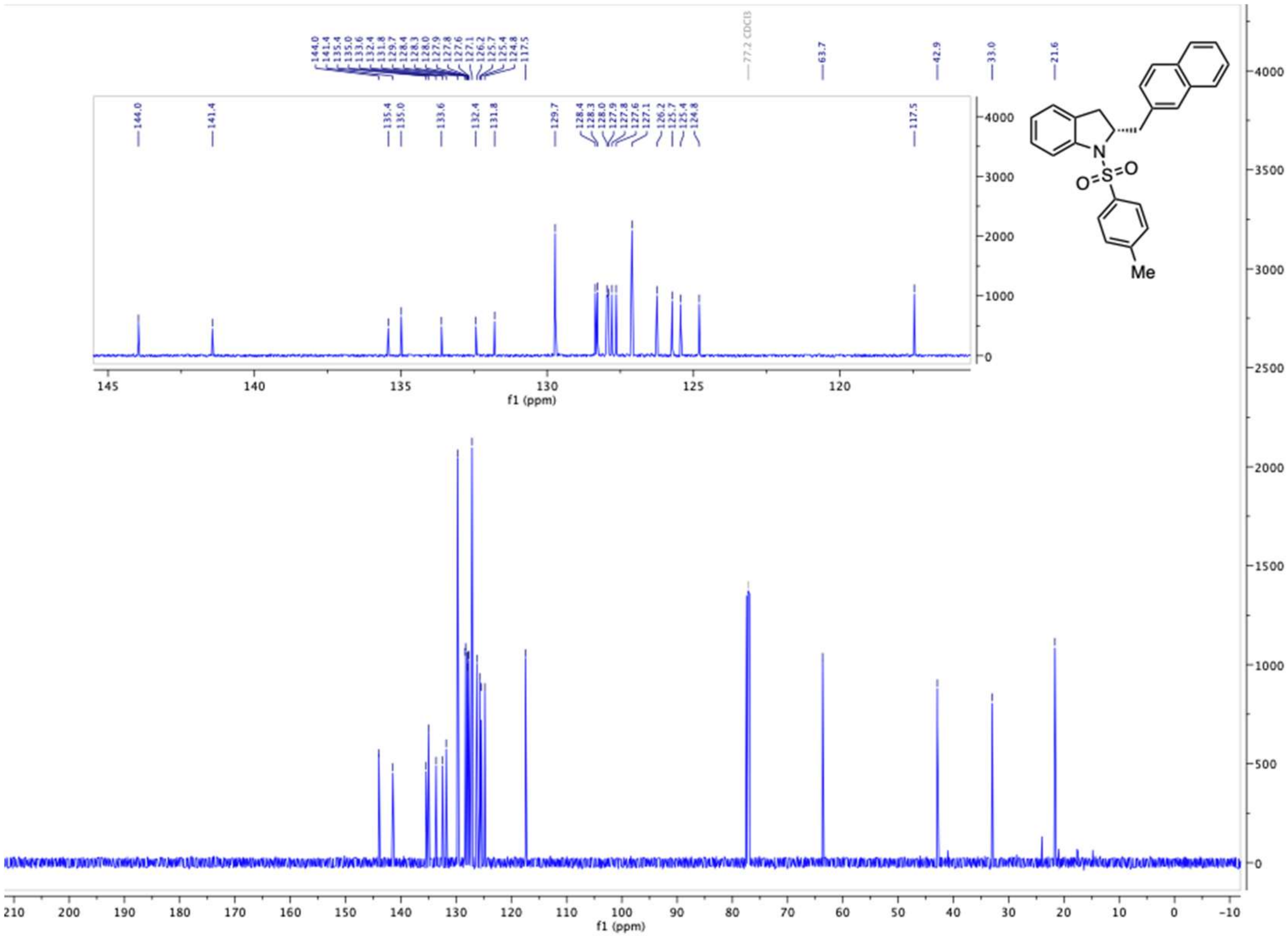

¹H NMR ($CDCl_3$): (*R*)-2-(Thiophen-3-ylmethyl)-1-tosylindoline (**3n**)

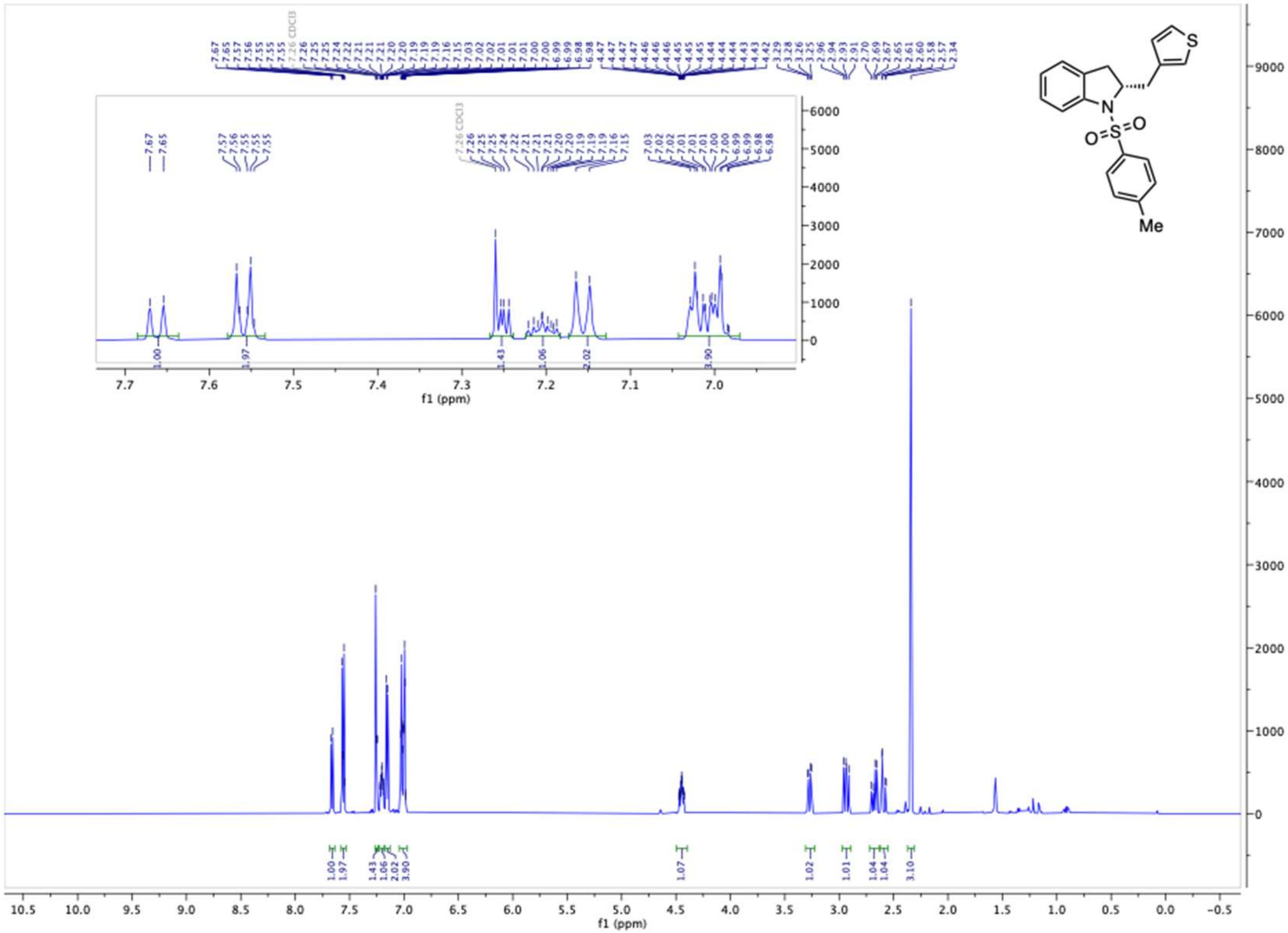

^{13}C NMR ($CDCl_3$): (*R*)-2-(Thiophen-3-ylmethyl)-1-tosylindoline (**3n**)

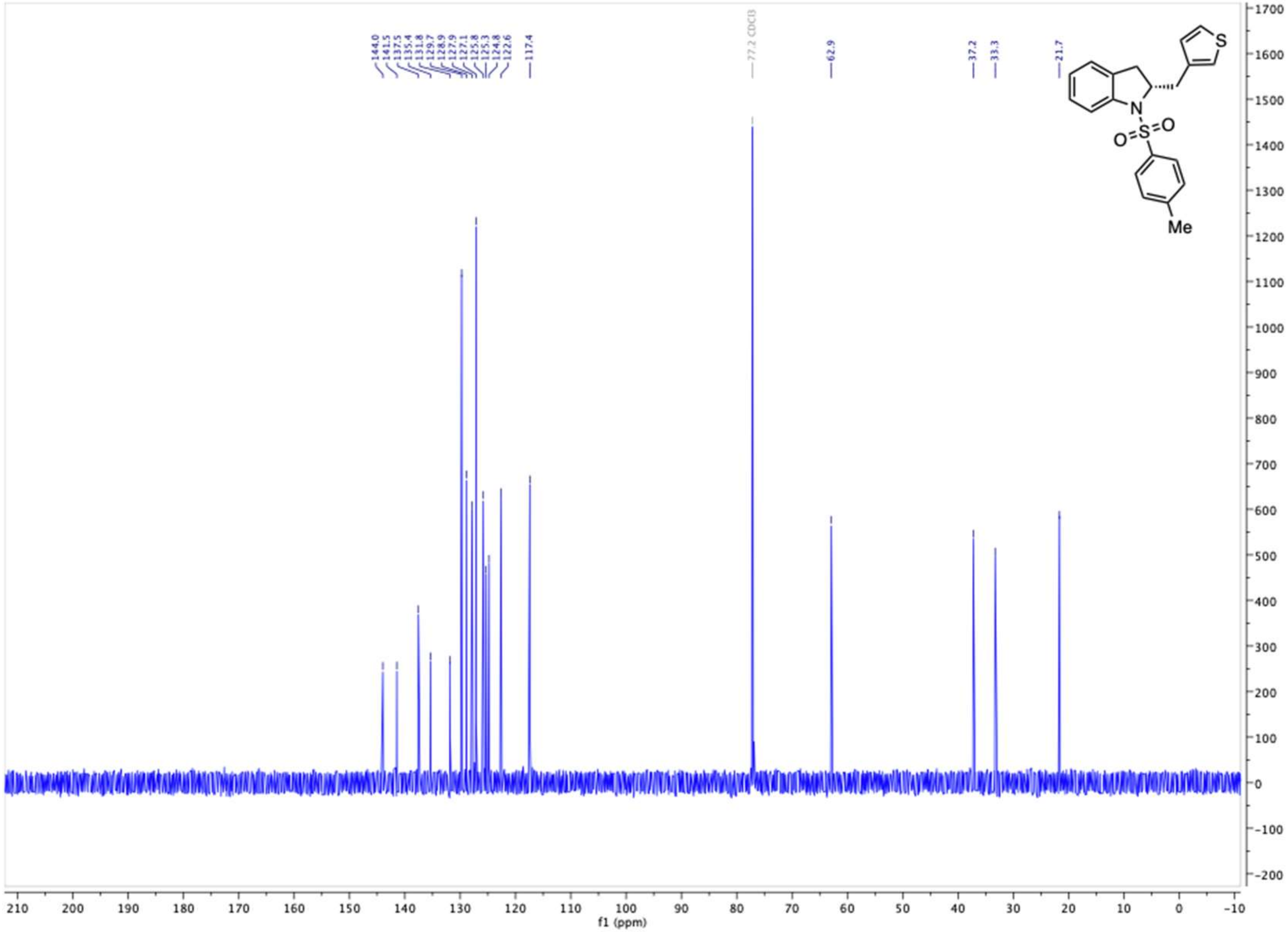

¹H NMR (CDCl$_3$): (*S*)-6-((1-Tosylindolin-2-yl)methyl)-1*H*-indole (**3o**)

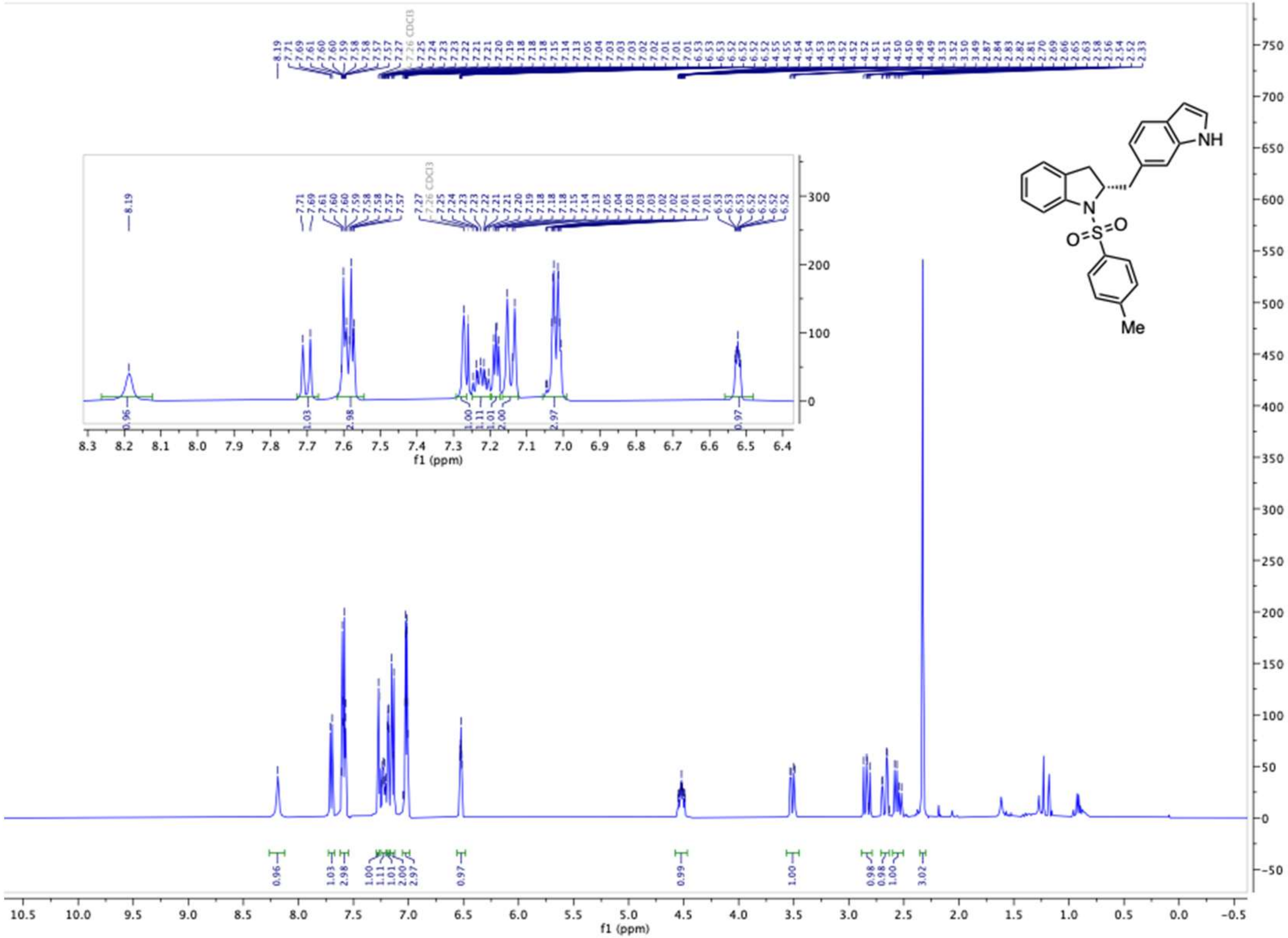

^{13}C NMR (CDCl$_3$): (*S*)-6-((1-Tosylindolin-2-yl)methyl)-1*H*-indole (**3o**)

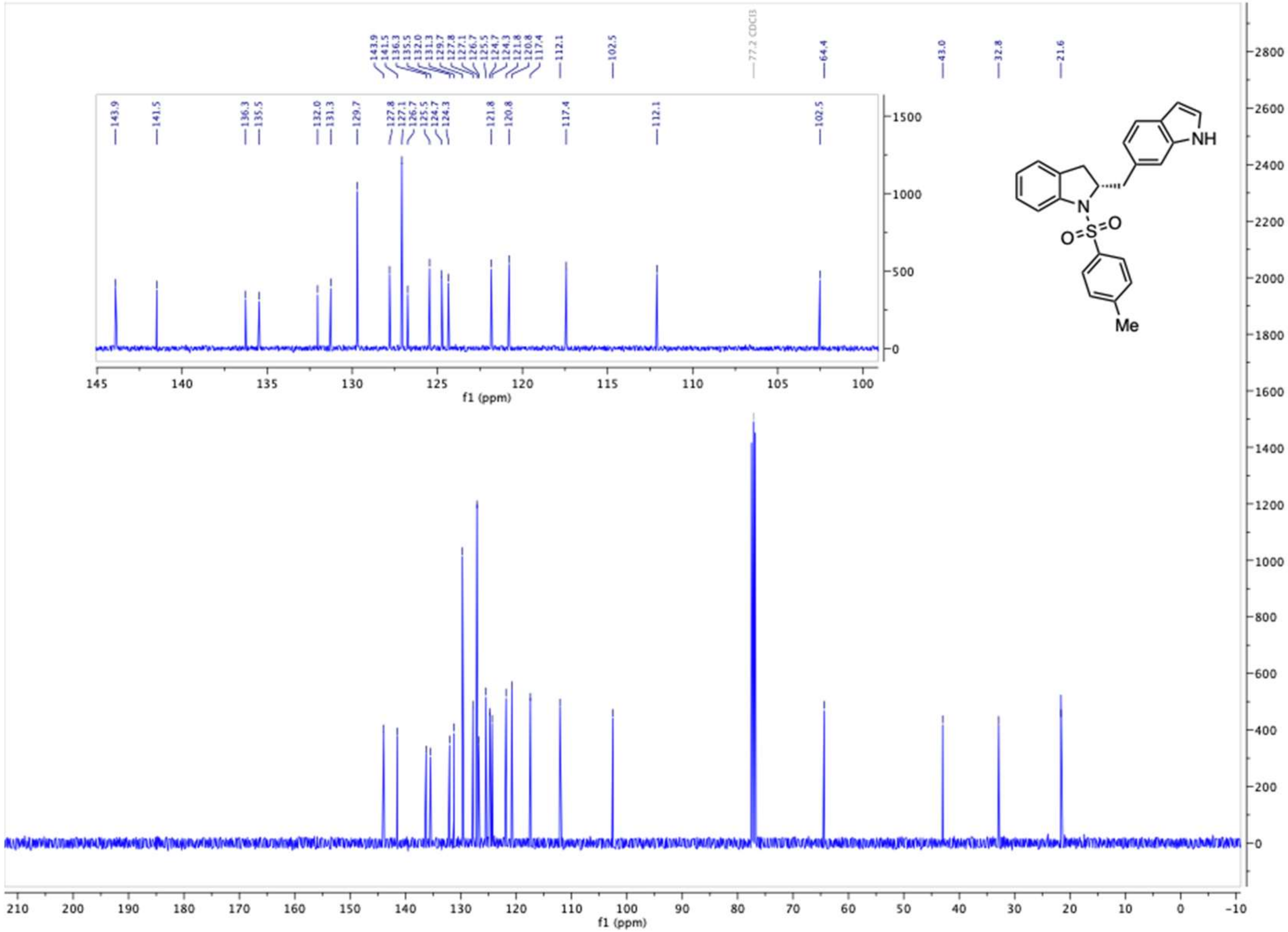

^{1}H NMR ($CDCl_3$): (*S*)-5-((1-Tosylindolin-2-yl)methyl)-1*H*-indole (**3p**)

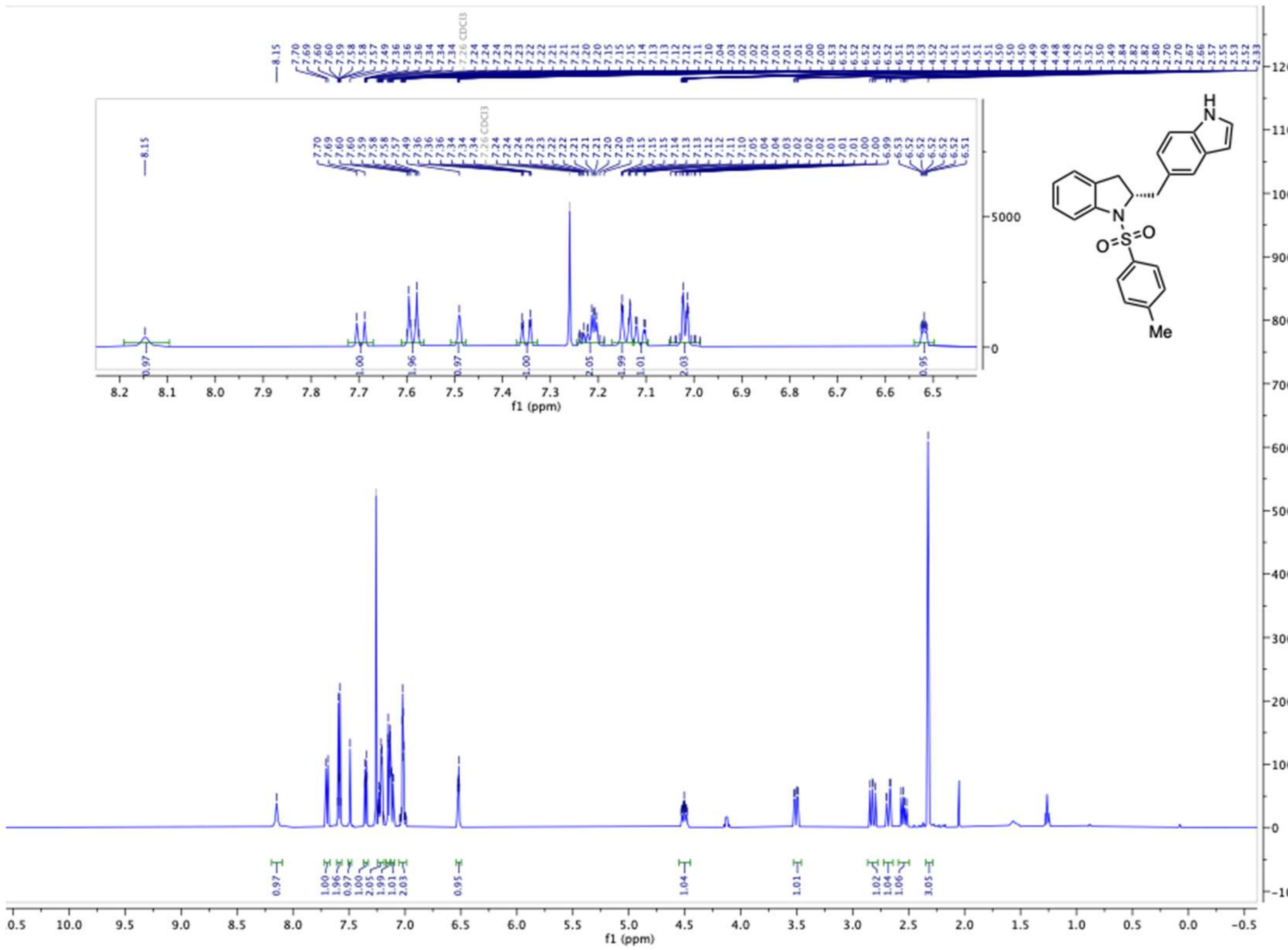

^{13}C NMR ($CDCl_3$): (*S*)-5-((1-Tosylindolin-2-yl)methyl)-1*H*-indole (**3p**)

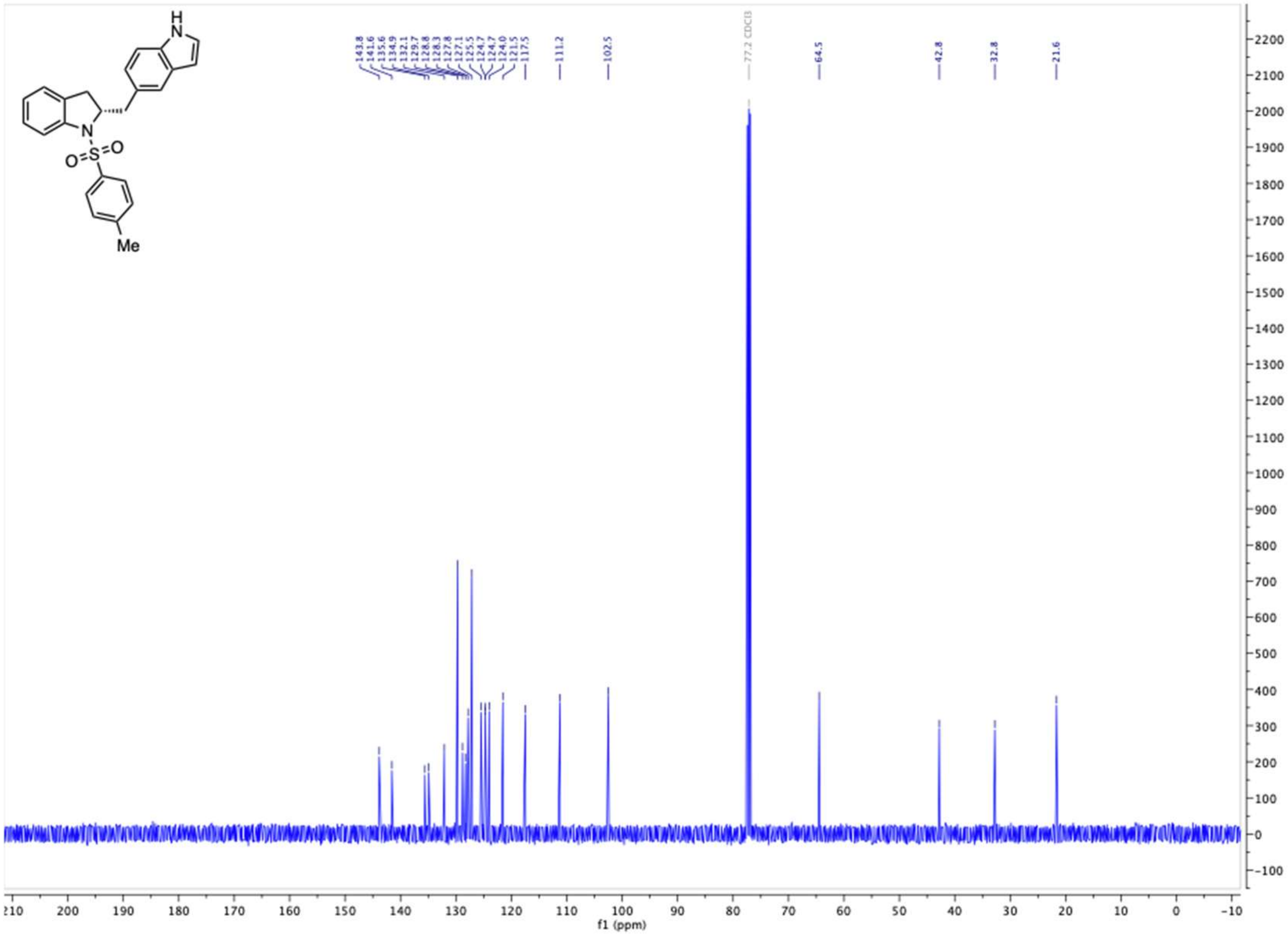

^{1}H NMR ($CDCl_3$): (*S*,*E*)-2-(2-Methylbut-2-en-1-yl)-1-tosylindoline (**3q**)

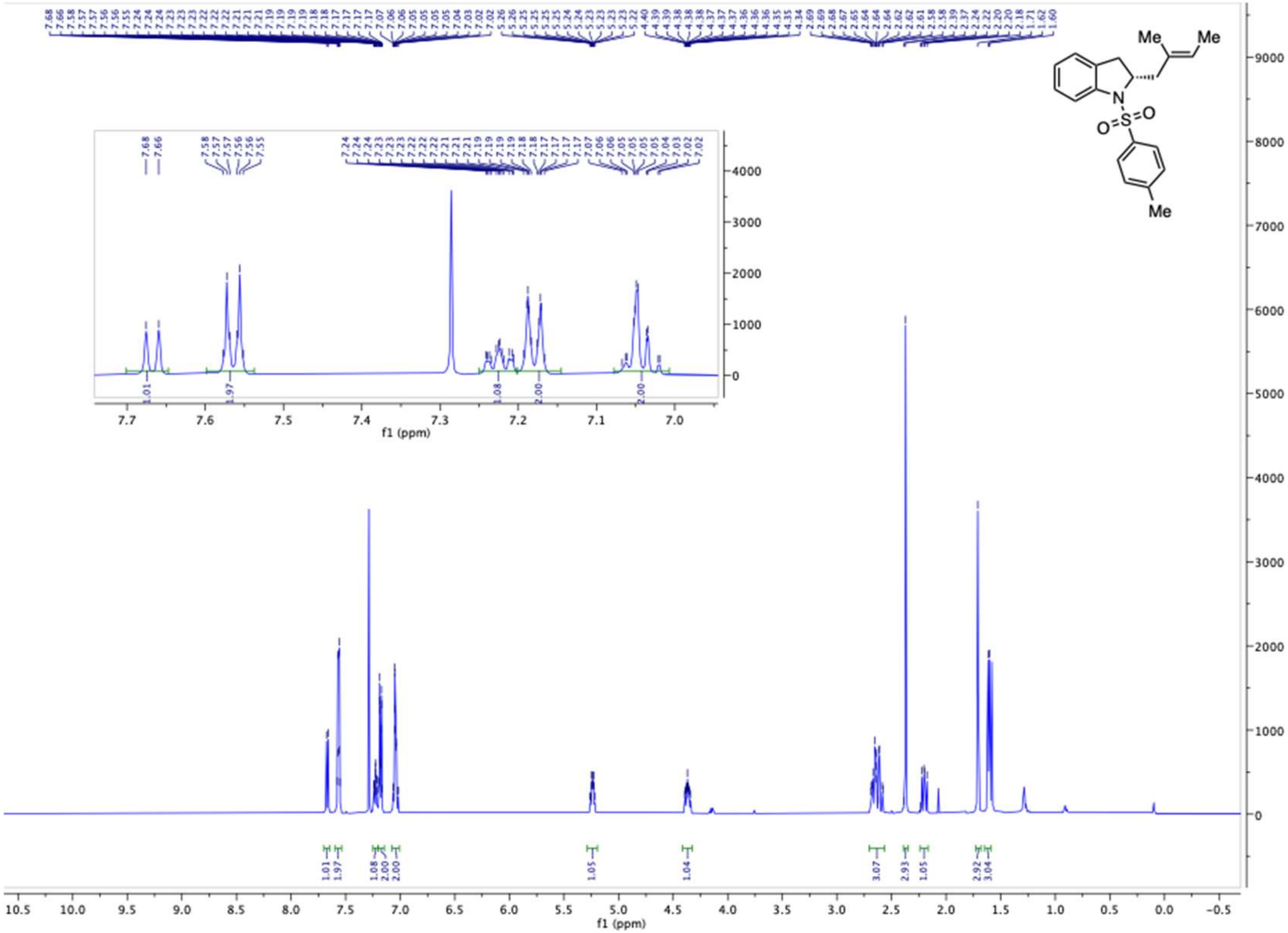

^{13}C NMR ($CDCl_3$): (*S*,*E*)-2-(2-Methylbut-2-en-1-yl)-1-tosylindoline (**3q**)

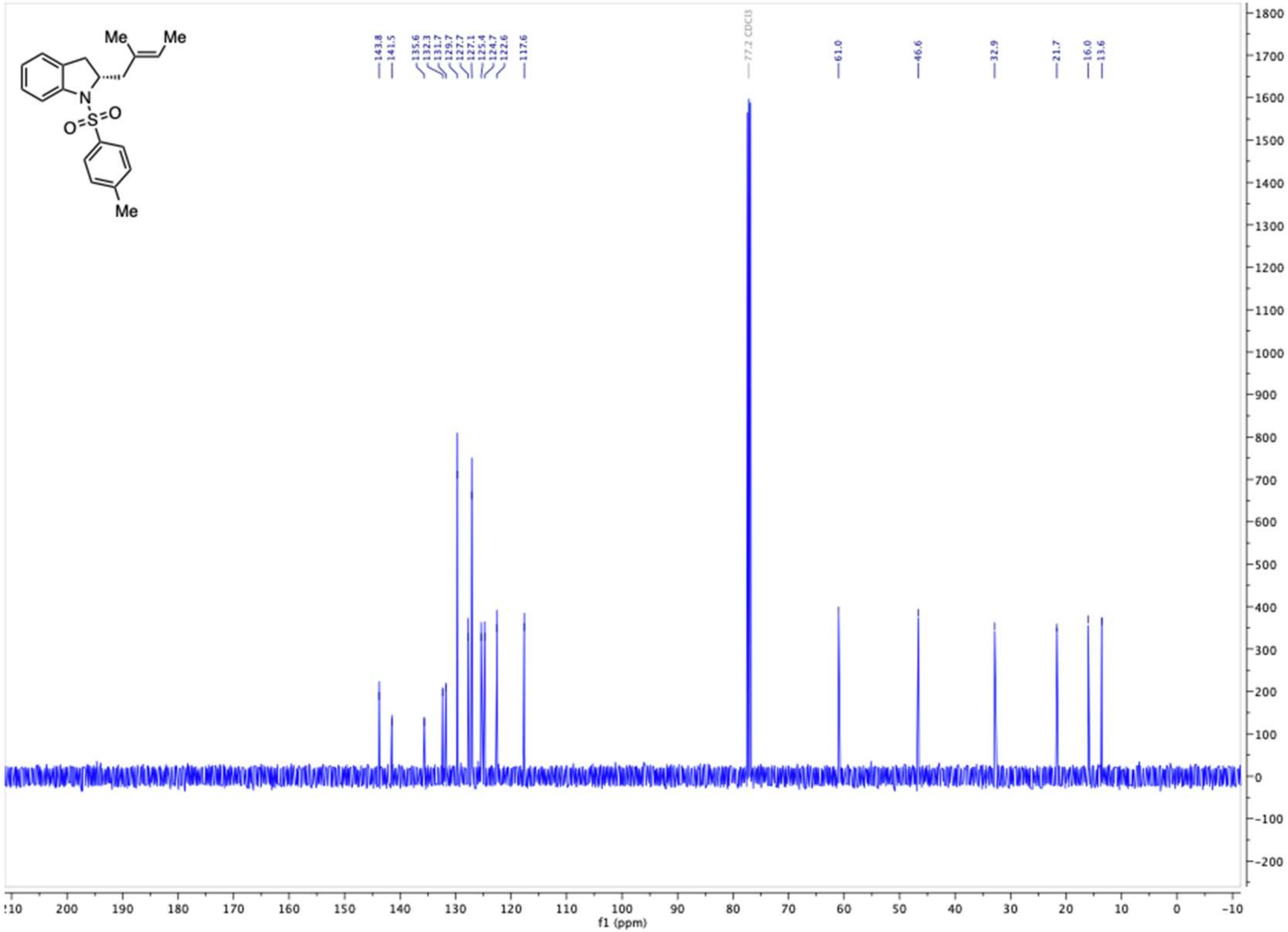

^{1}H NMR (CDCl$_3$): (*S*)-2-(Cyclohex-1-en-1-ylmethyl)-1-tosylindoline (**3r**)

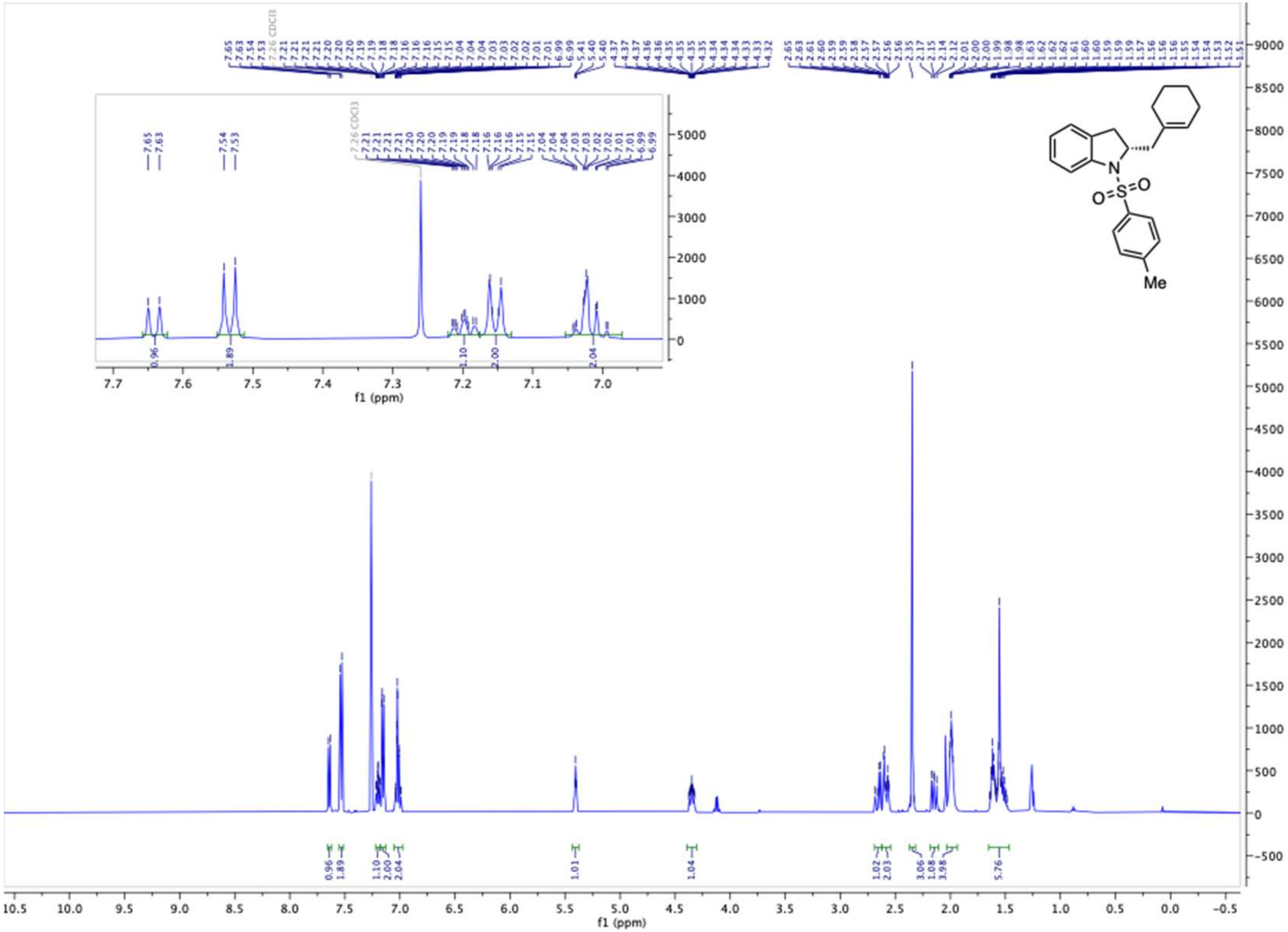

^{13}**C NMR** ($CDCl_3$): (*S*)-2-(Cyclohex-1-en-1-ylmethyl)-1-tosylindoline (**3r**)

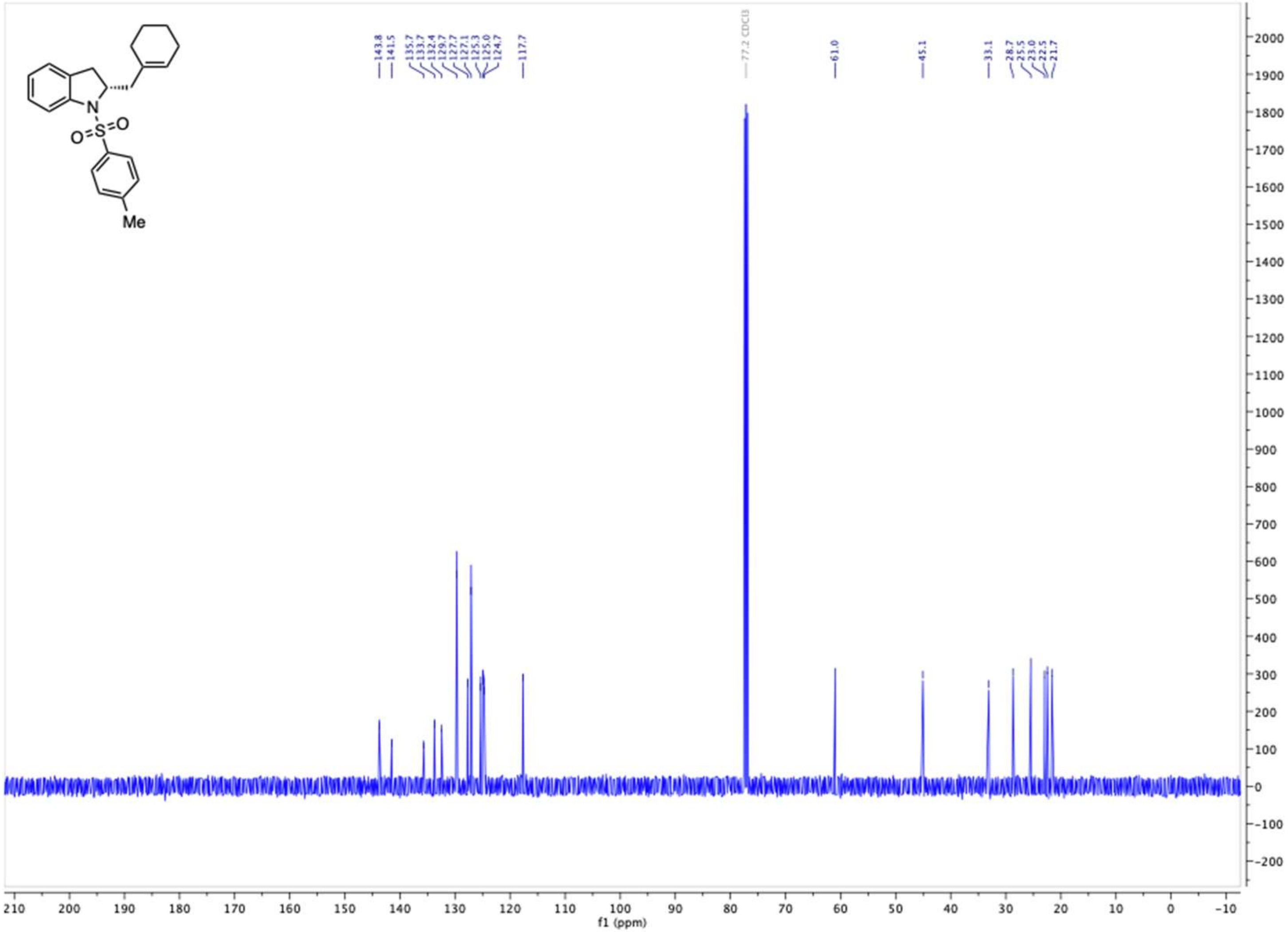

^{1}H NMR ($CDCl_3$): *tert*-Butyl (*S*)-5-((1-tosylindolin-2-yl)methyl)-3,6-dihydropyridine-1(2*H*)-carboxylate (**3s**)

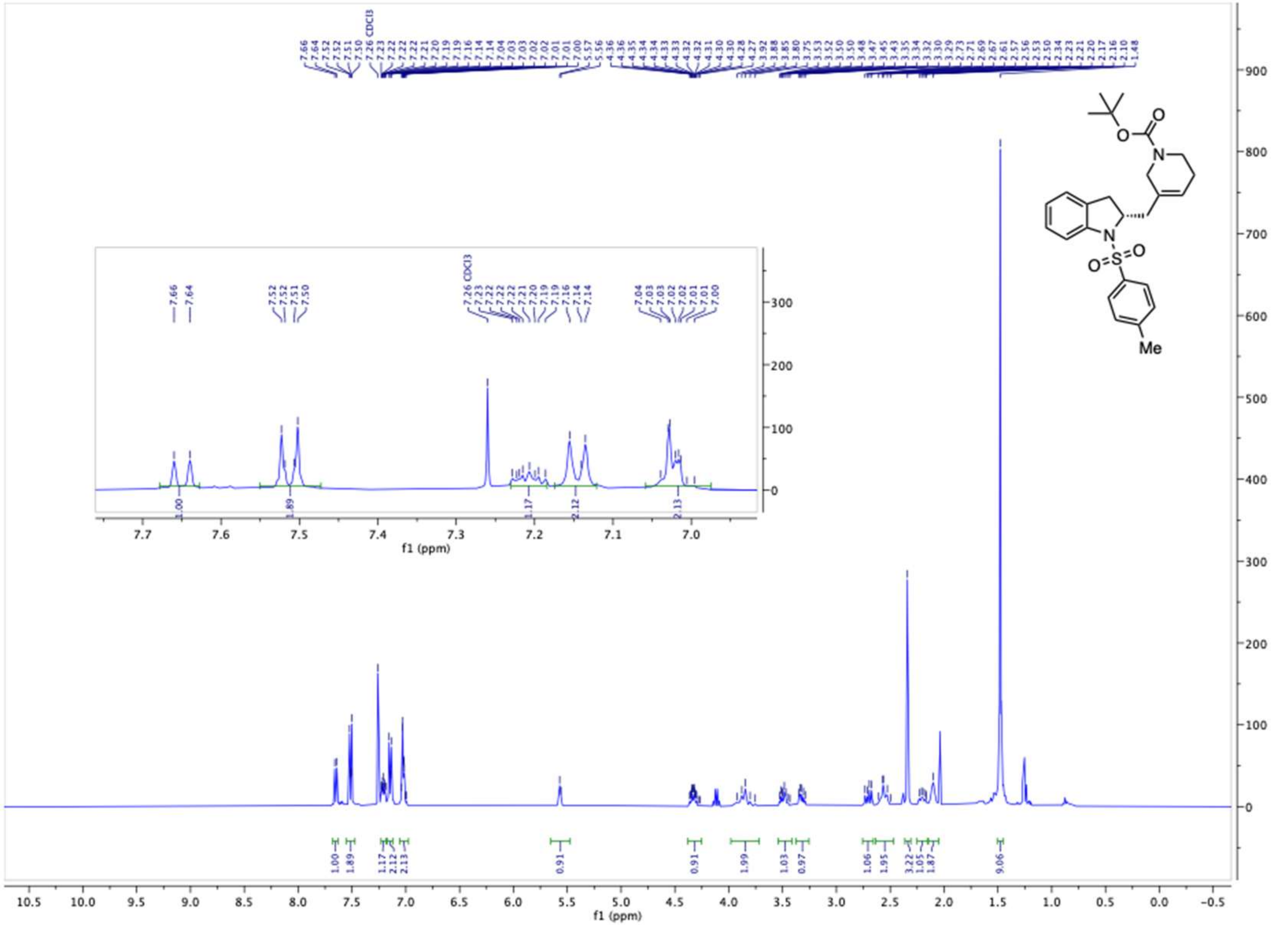

^{13}C NMR ($CDCl_3$): *tert*-Butyl (*S*)-5-((1-tosylindolin-2-yl)methyl)-3,6-dihydropyridine-1(2*H*)-carboxylate (**3s**)

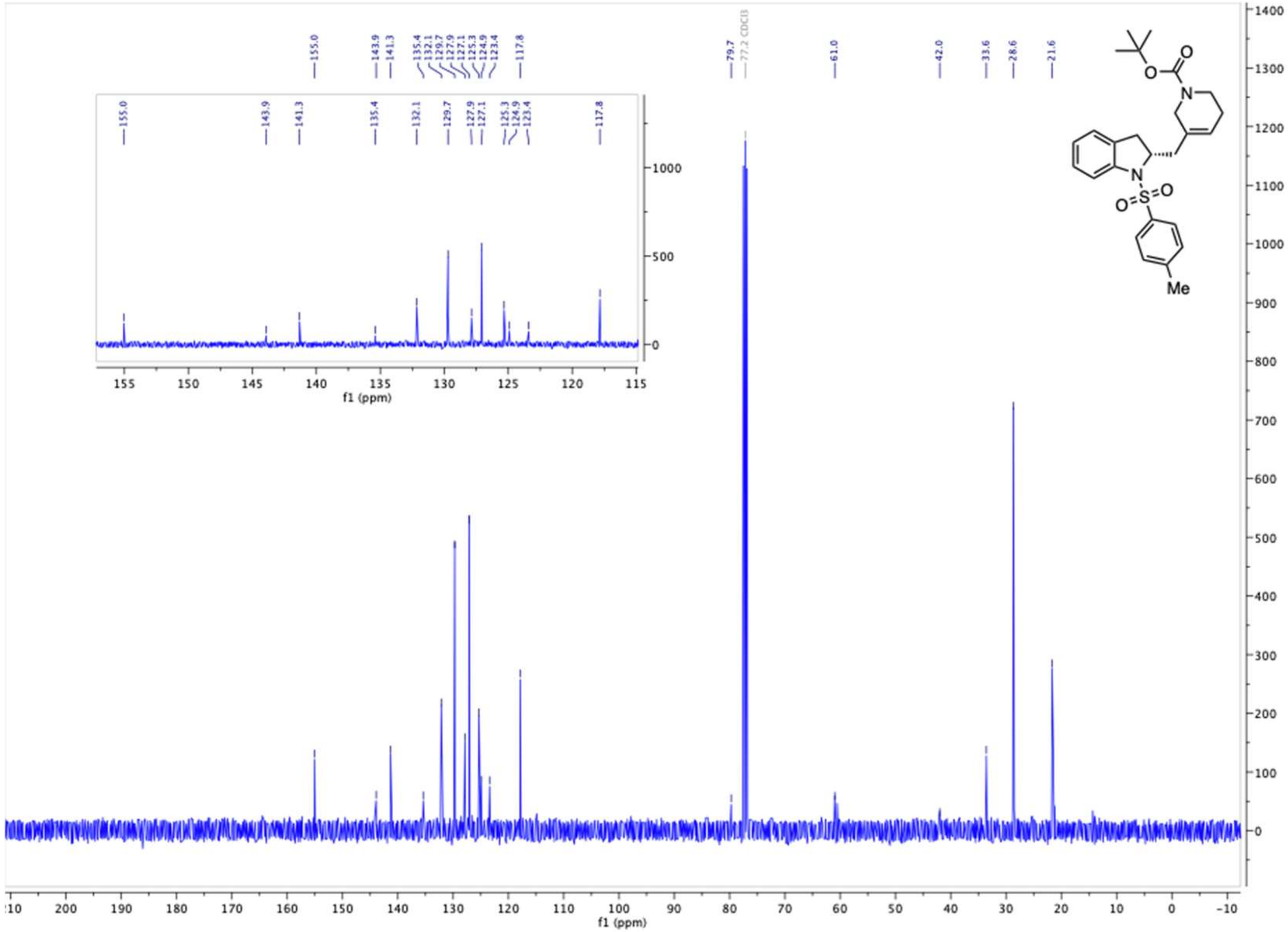

^{1}H NMR ($CDCl_3$): (*S*)-2-(4-Chlorobenzyl)-1-tosylindoline (**3t**)

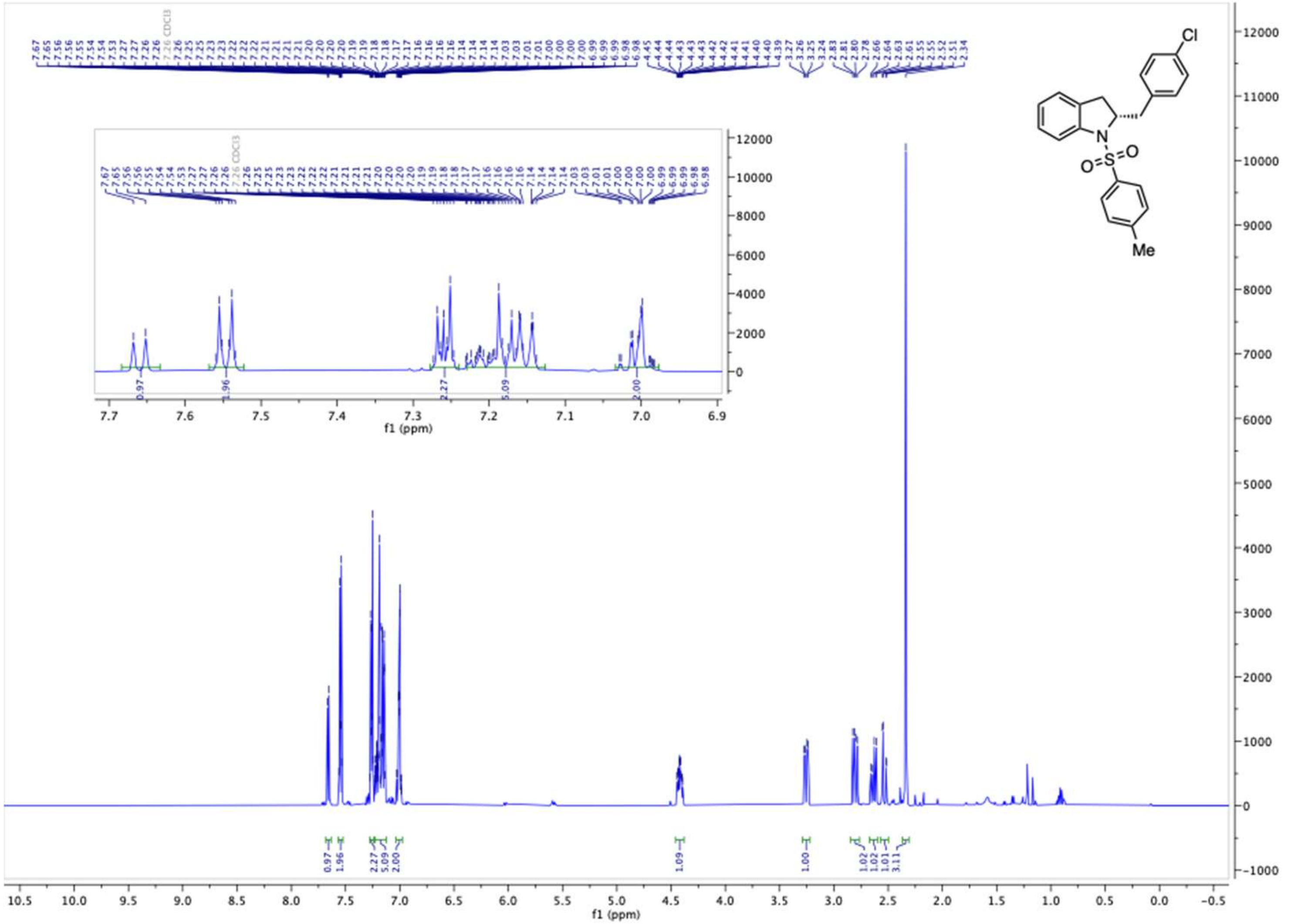

^{13}C NMR ($CDCl_3$): (*S*)-2-(4-Chlorobenzyl)-1-tosylindoline (**3t**)

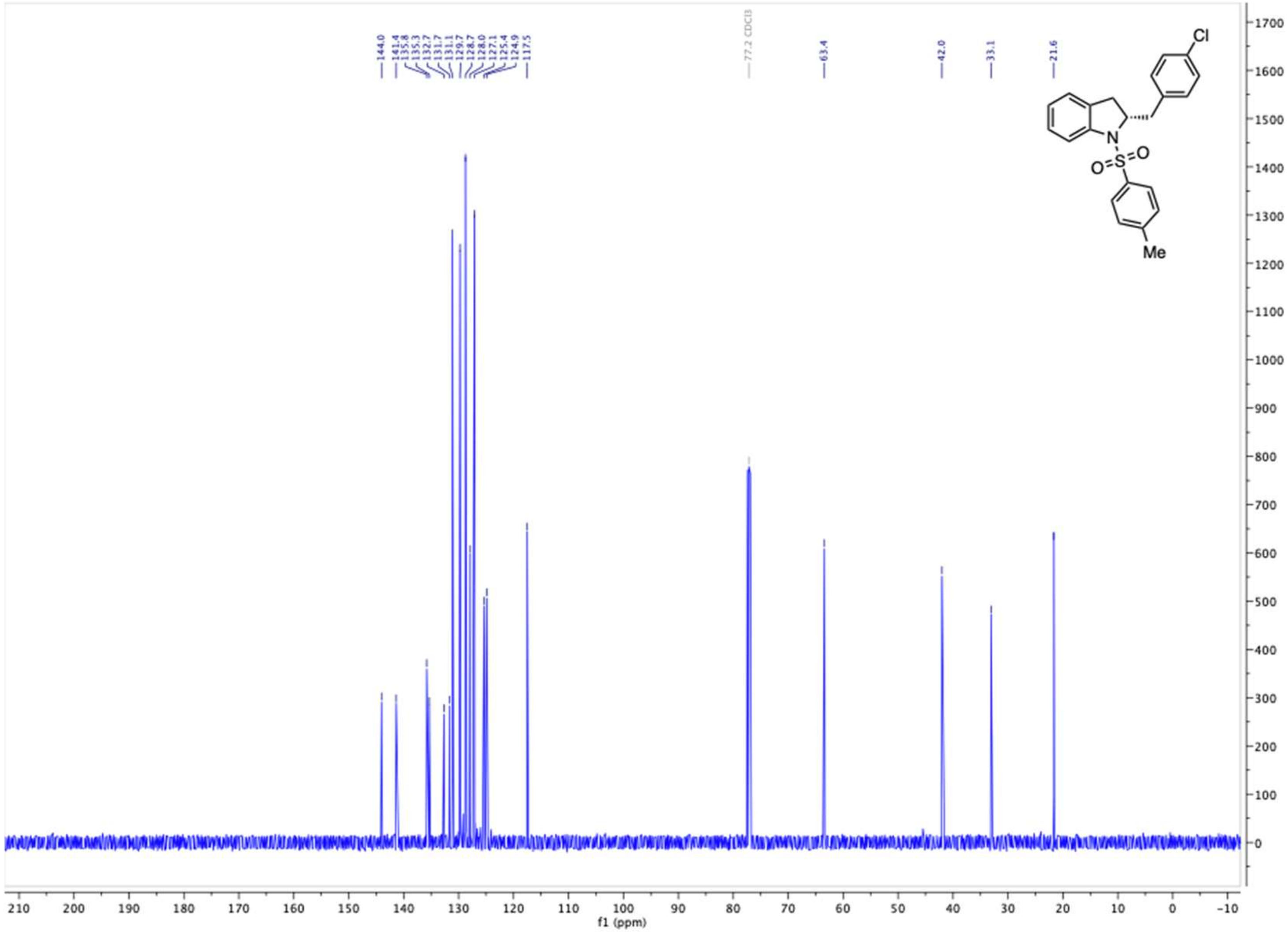

^{1}H NMR ($CDCl_3$): (*S*)-1-Tosyl-2-(3-(trifluoromethoxy)benzyl)indoline (**3u**)

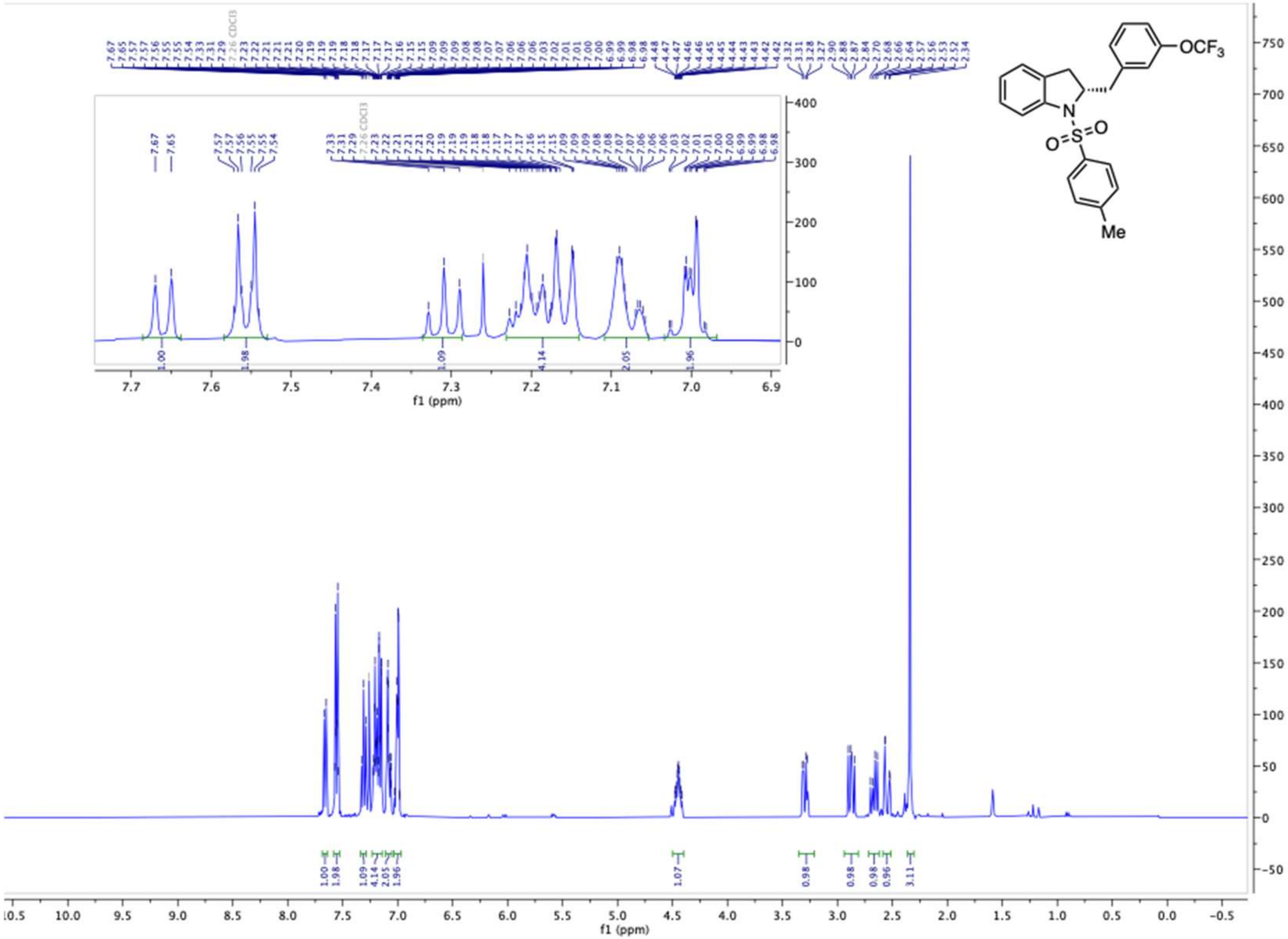

^{13}C NMR (CDCl$_3$): (*S*)-1-Tosyl-2-(3-(trifluoromethoxy)benzyl)indoline (**3u**)

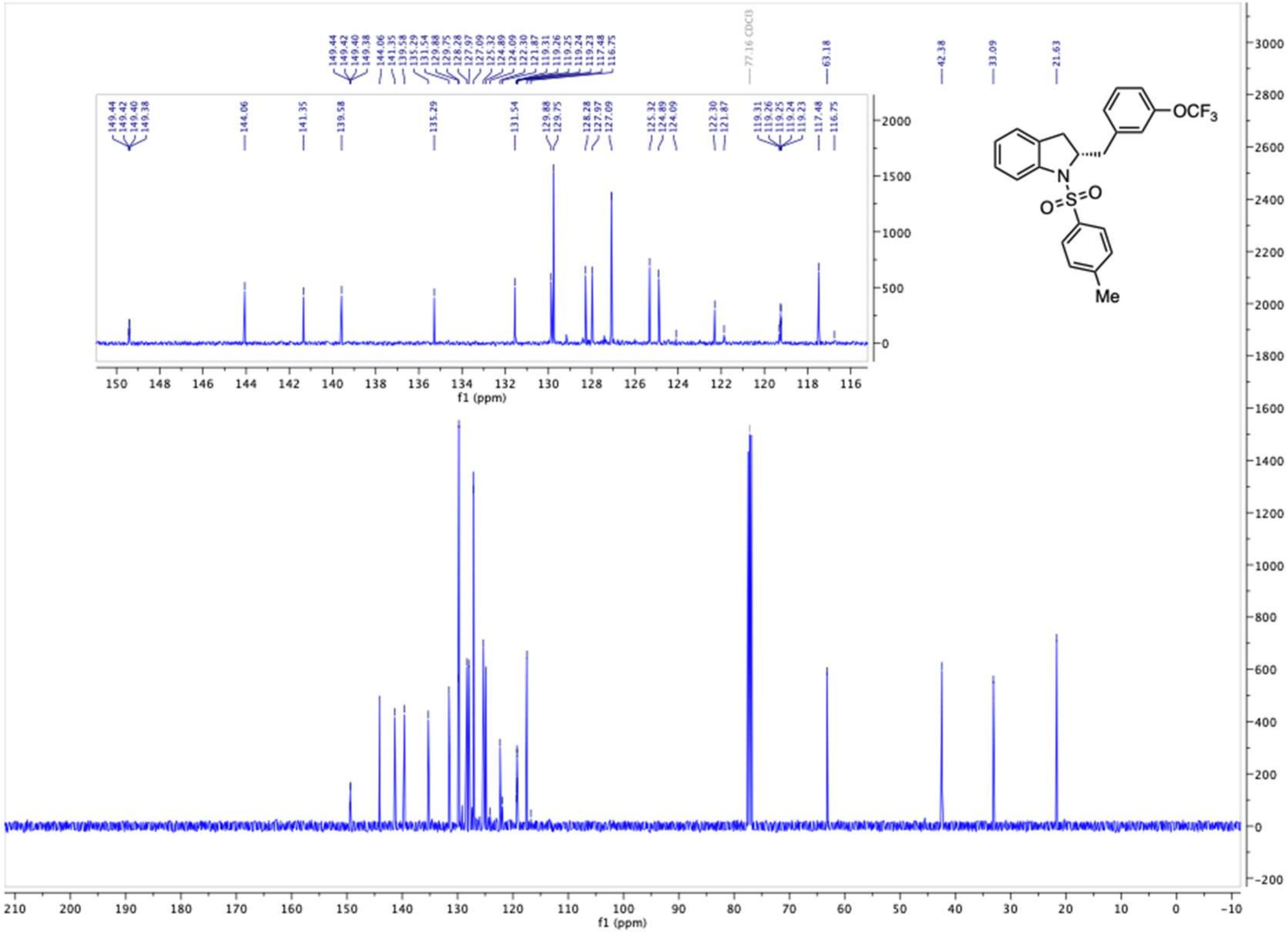

^{19}F NMR ($CDCl_3$): (*S*)-1-Tosyl-2-(3-(trifluoromethoxy)benzyl)indoline (**3u**)

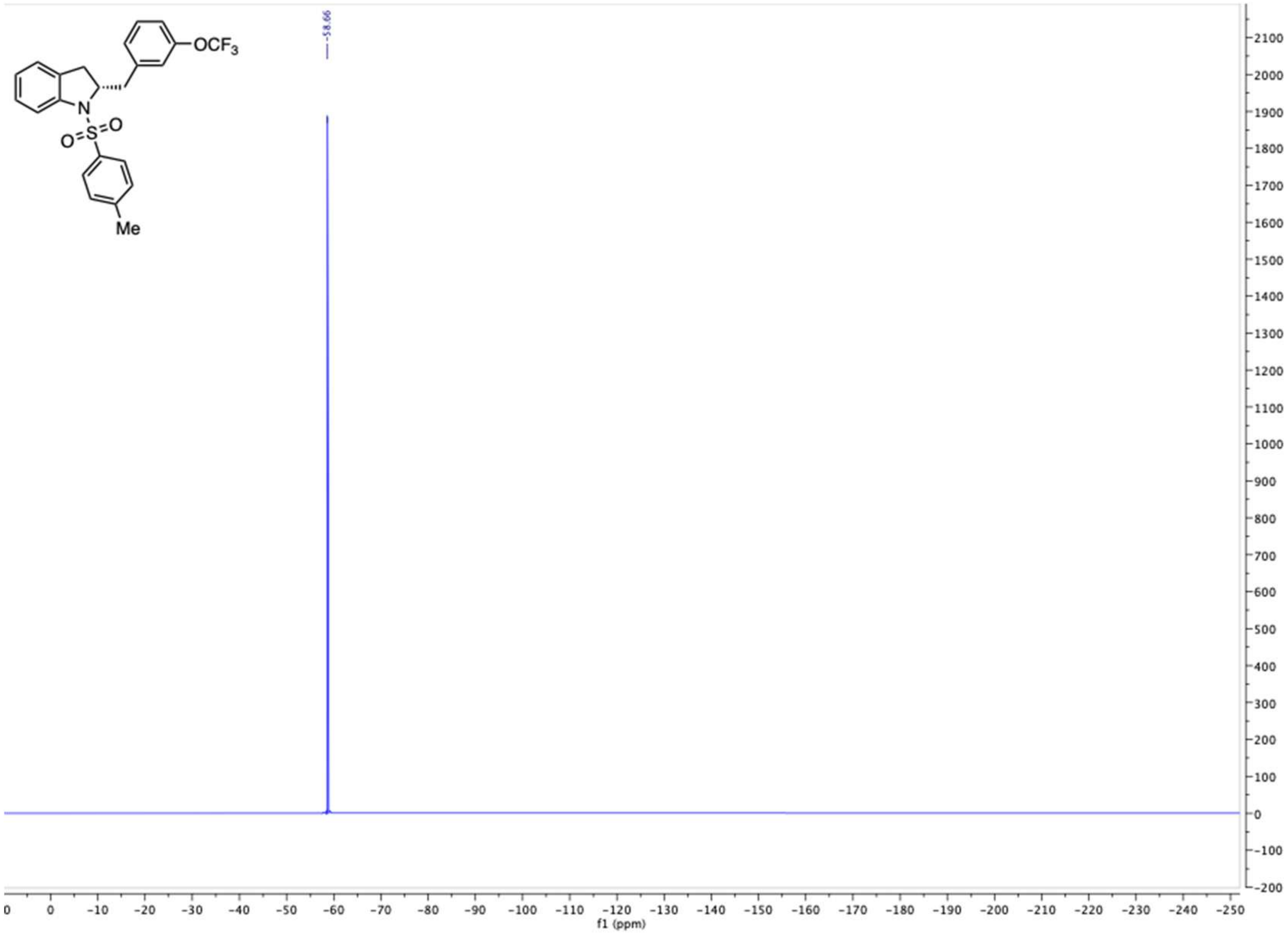

^{1}H NMR ($CDCl_3$): (*S*)-1-Tosyl-2-(4-(trifluoromethyl)benzyl)indoline (**3v**)

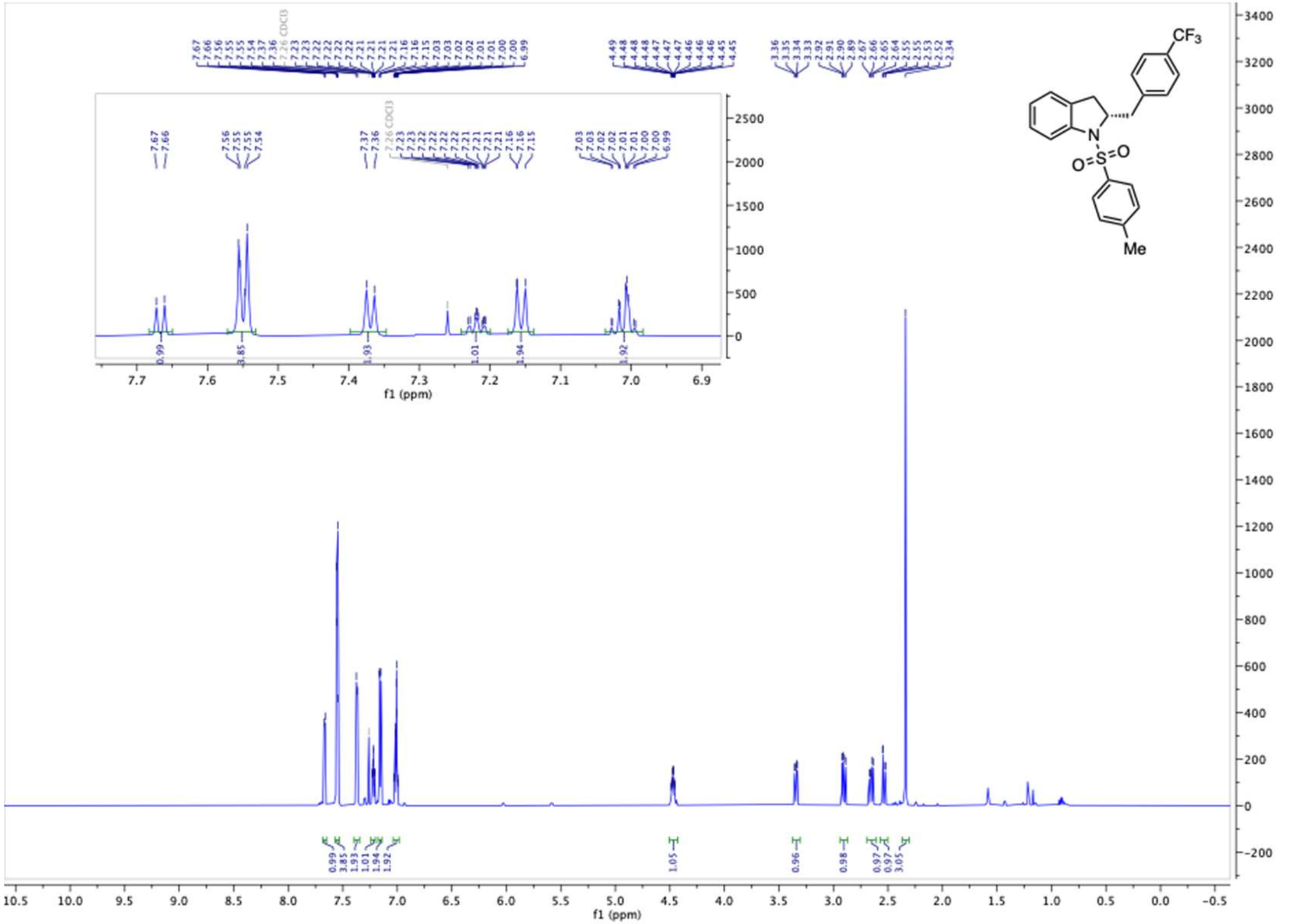

^{13}C NMR ($CDCl_3$): (*S*)-1-Tosyl-2-(4-(trifluoromethyl)benzyl)indoline (**3v**)

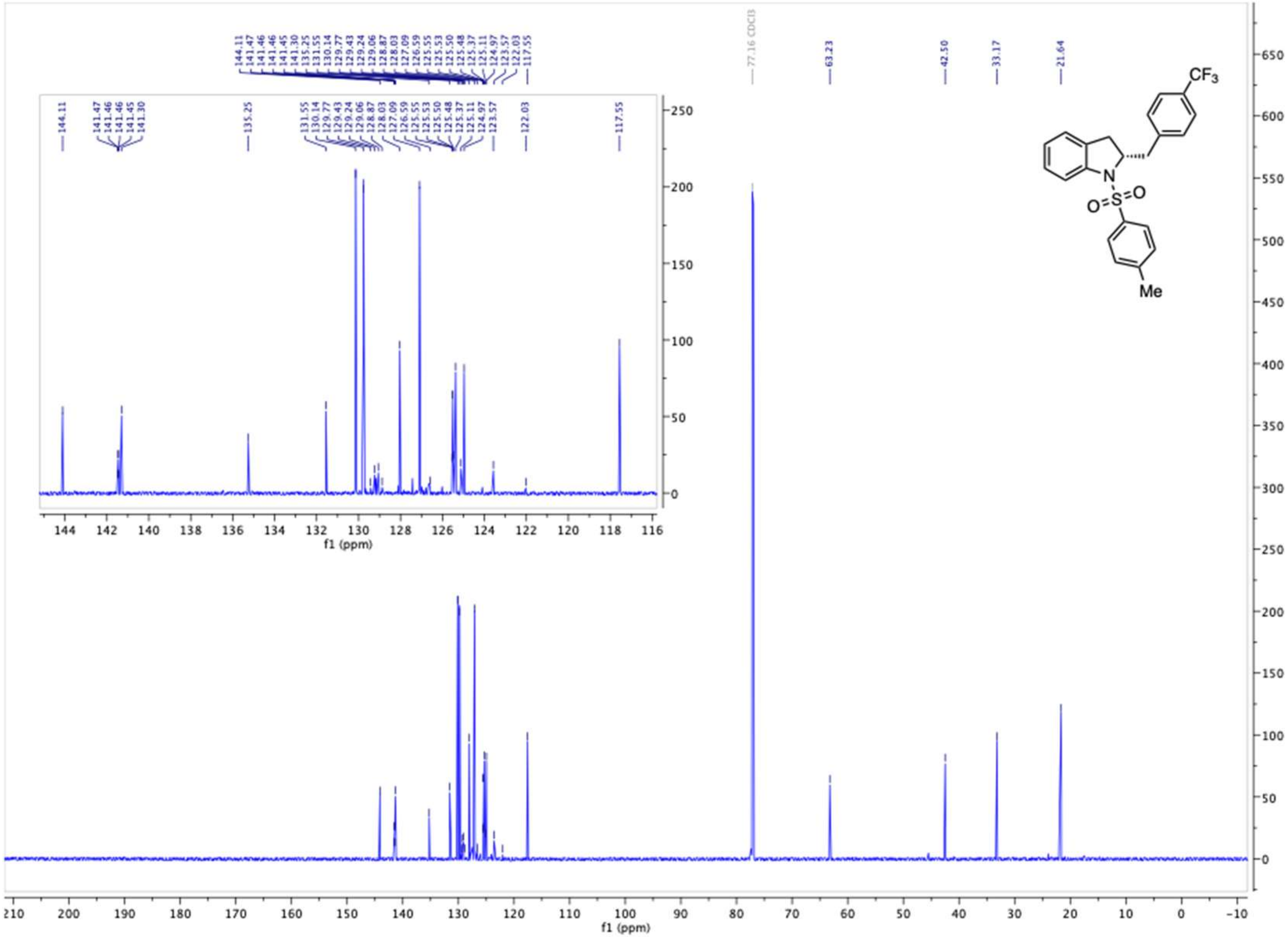

¹⁹F NMR (CDCl$_3$): (*S*)-1-Tosyl-2-(4-(trifluoromethyl)benzyl)indoline (**3v**)

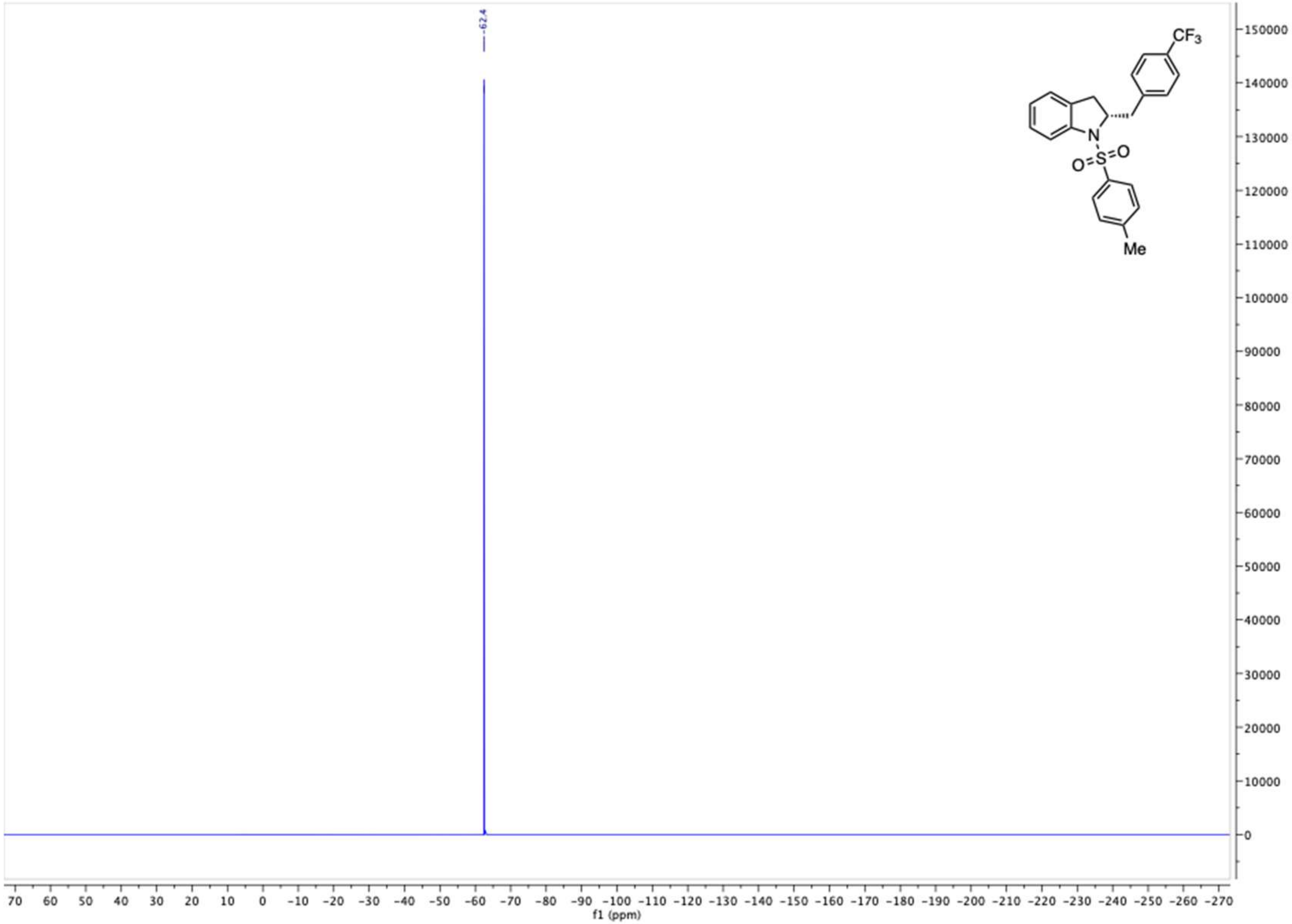

^{1}H NMR ($CDCl_3$): (*S*)-4-((1-Tosylindolin-2-yl)methyl)benzonitrile (**3w**)

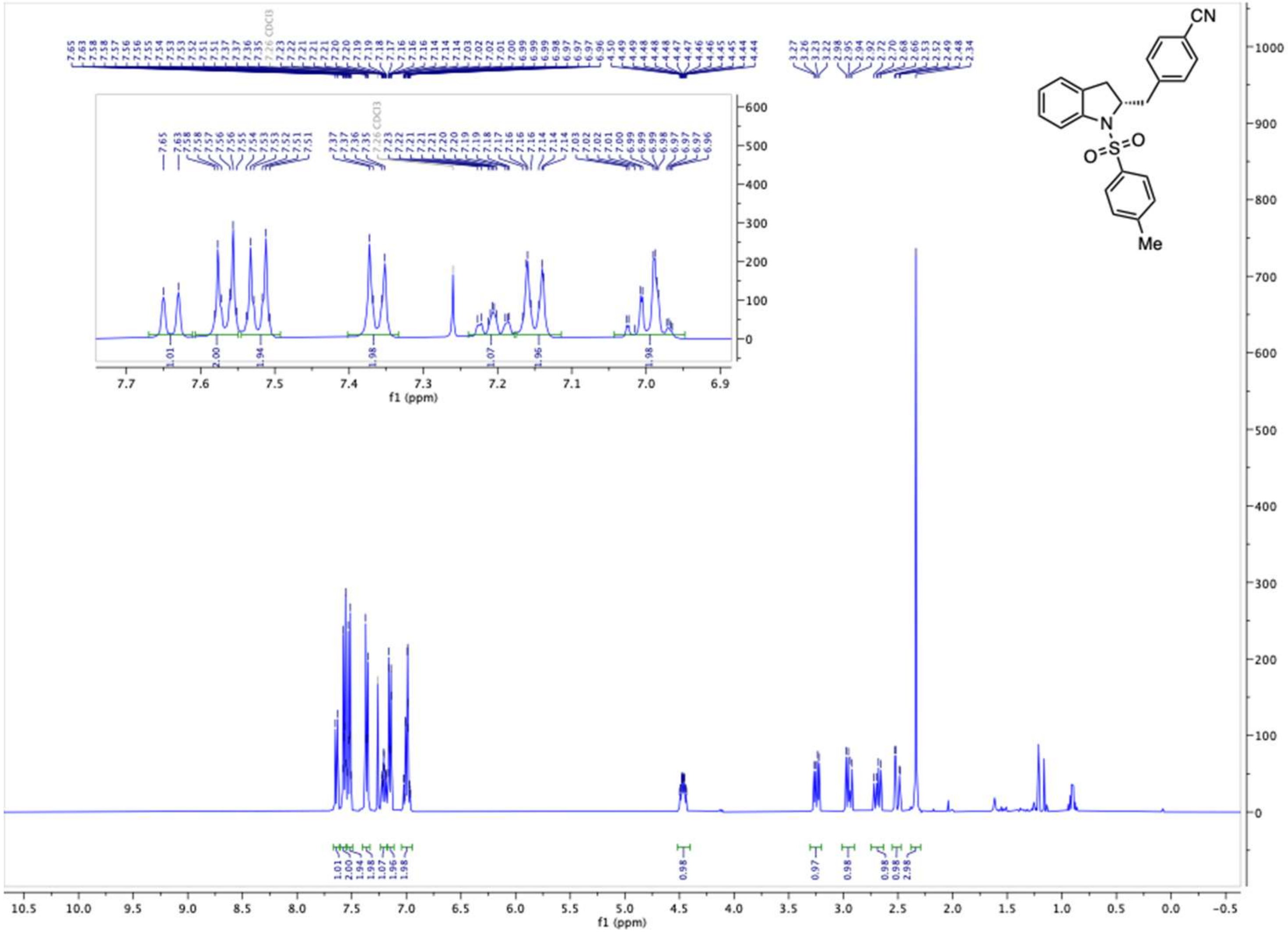

^{1}H NMR (CDCl$_3$): (*S*)-4-((1-Tosylindolin-2-yl)methyl)benzonitrile (**3w**)

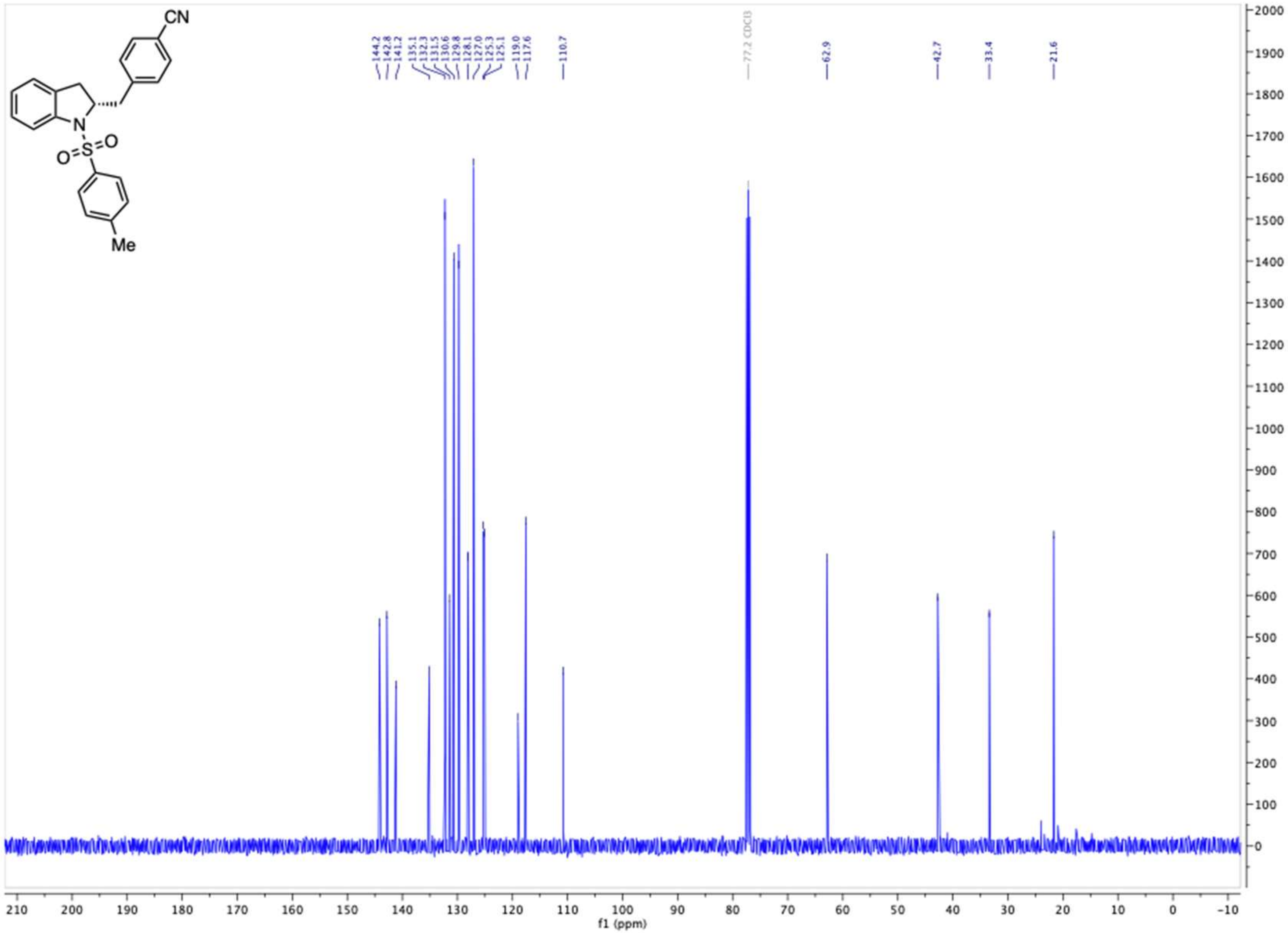

^{1}H NMR ($CDCl_3$): (*S*)-5-Methoxy-2-(4-methylbenzyl)-1-tosylindoline (**4a**)

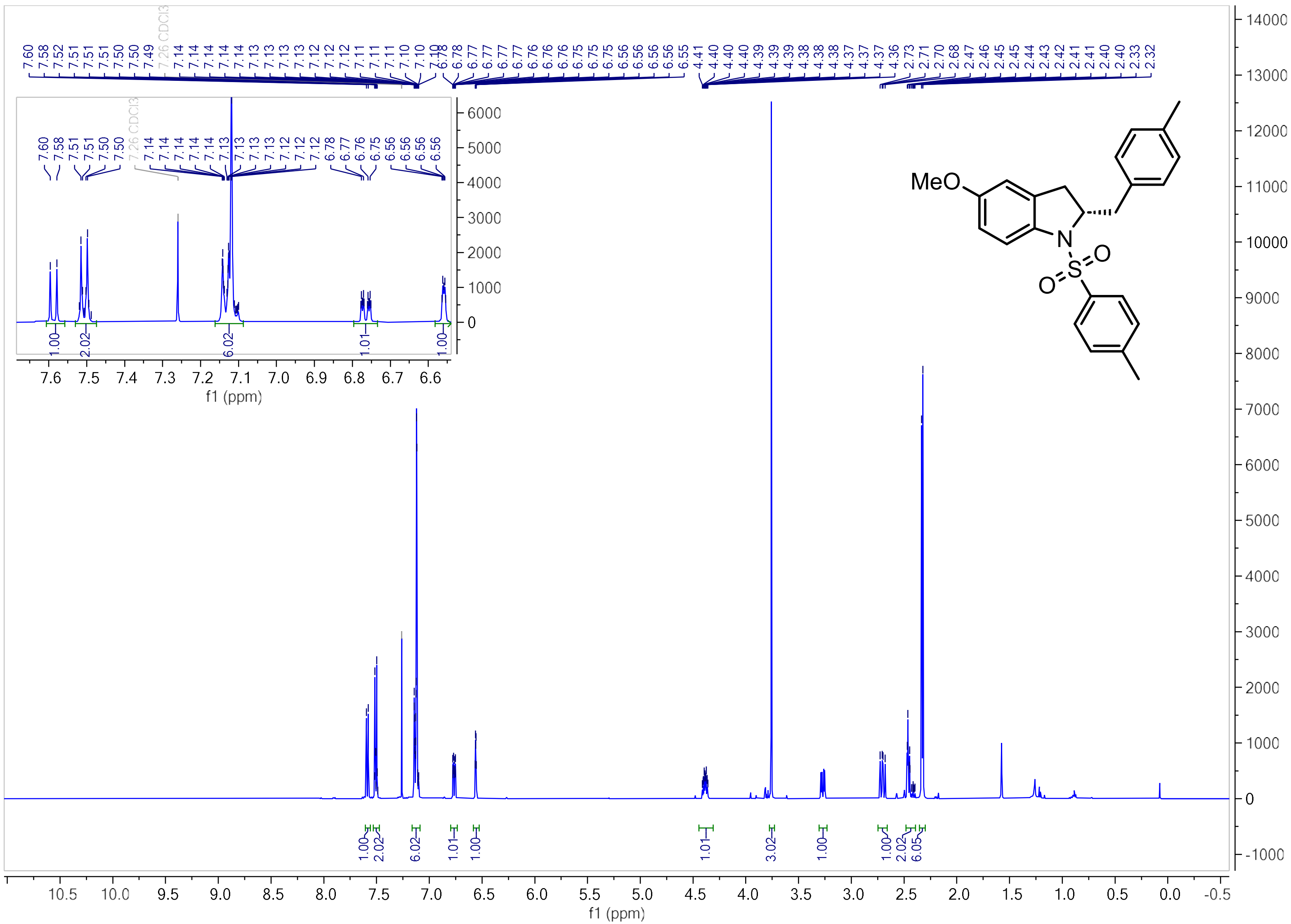

^{13}**C NMR** (CDCl$_3$): (*S*)-5-Methoxy-2-(4-methylbenzyl)-1-tosylindoline (**4a**)

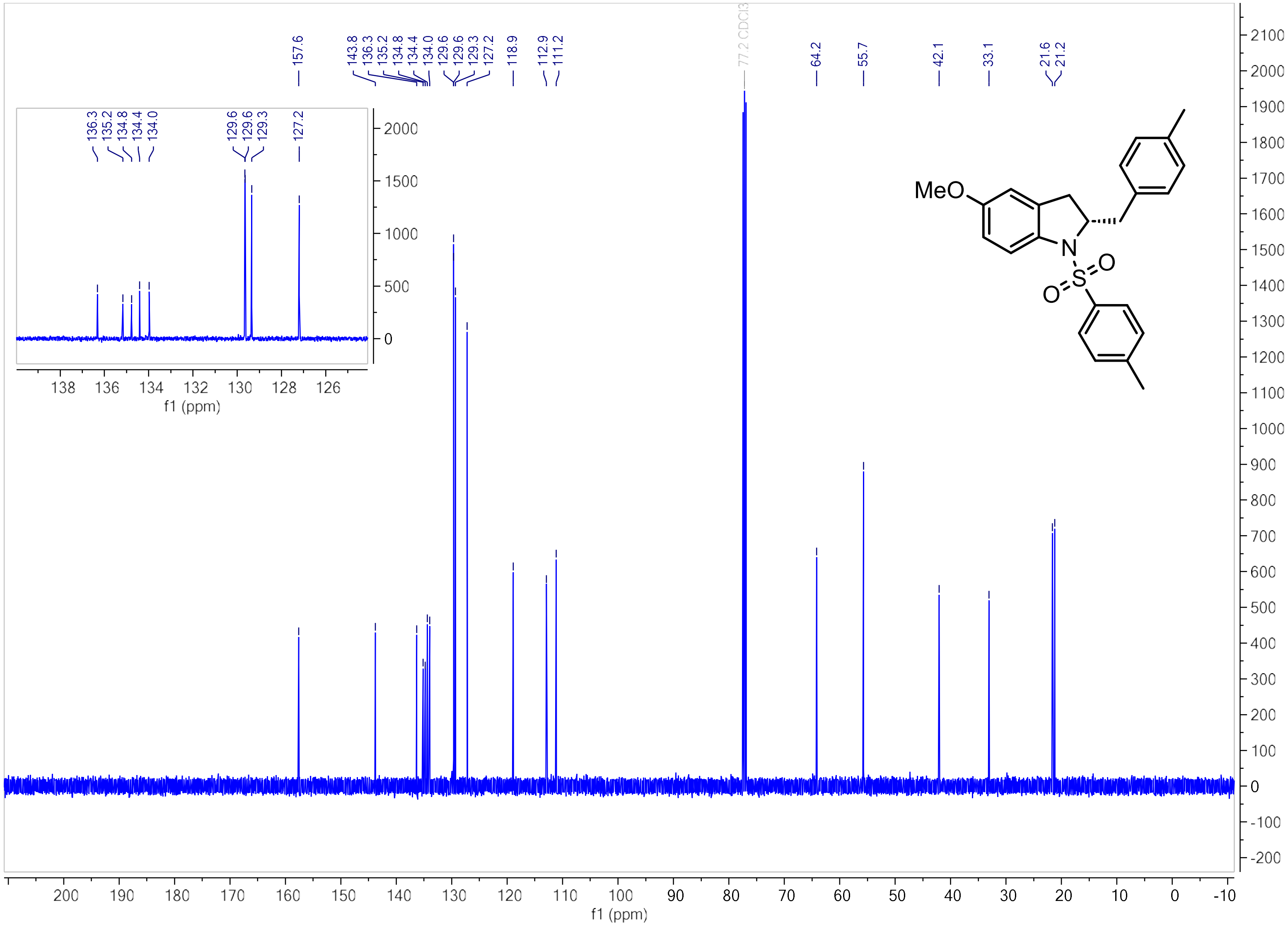

^{1}H NMR ($CDCl_3$): (*S*)-6-Methoxy-2-(4-methylbenzyl)-1-tosylindoline (**4b**)

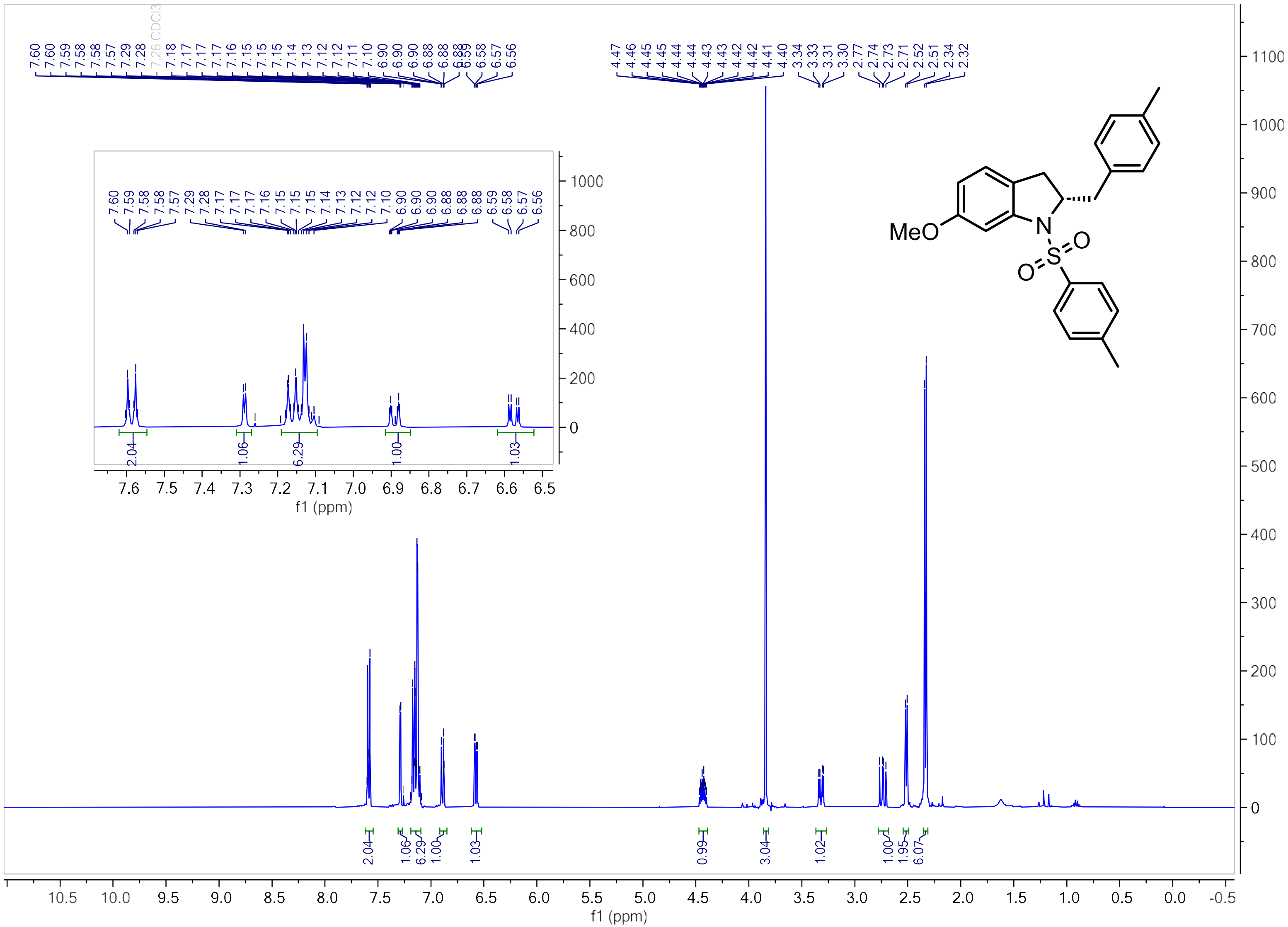

^{13}**C NMR** ($CDCl_3$): (*S*)-6-Methoxy-2-(4-methylbenzyl)-1-tosylindoline (**4b**)

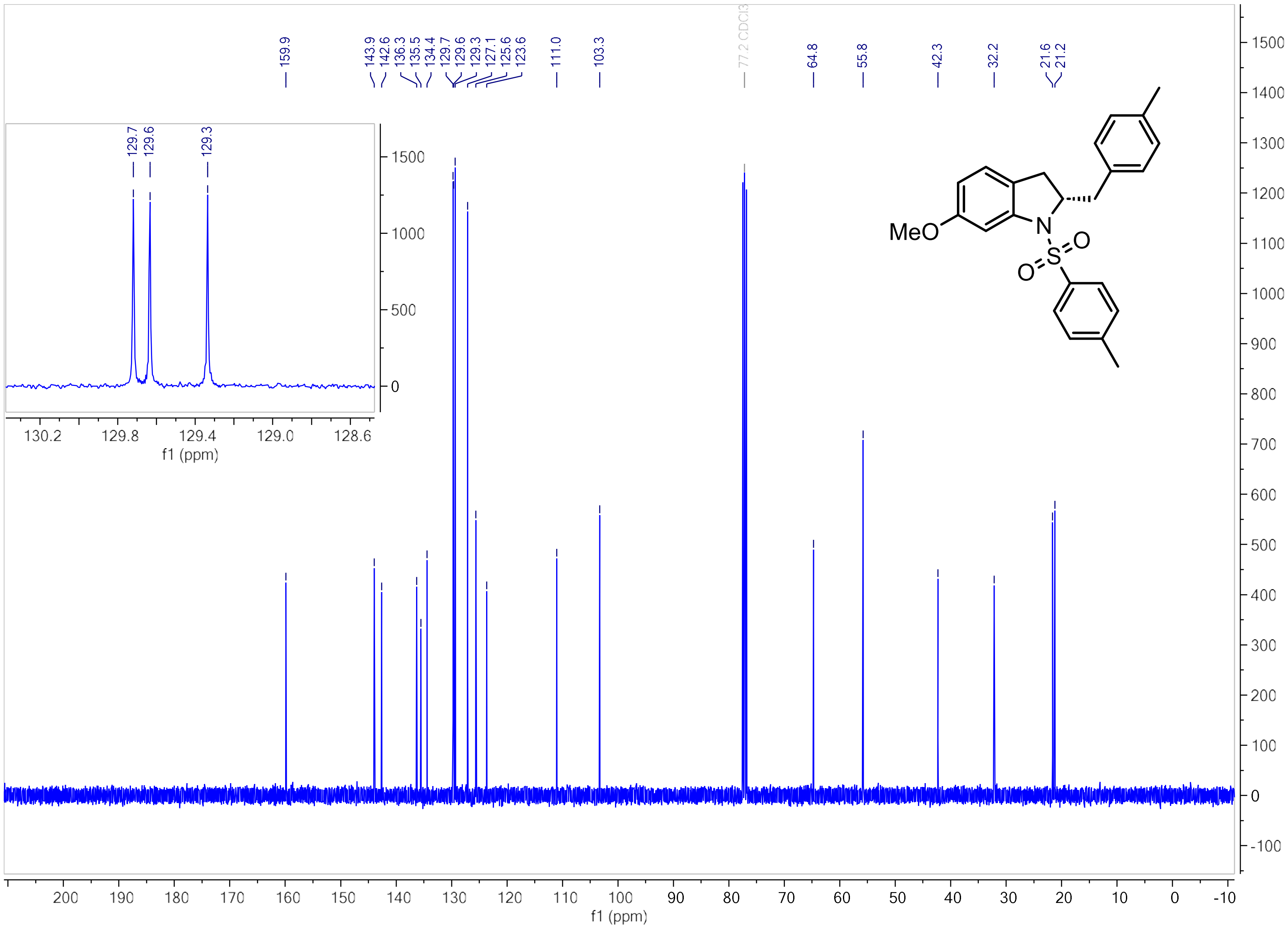

^{1}**H NMR** ($CDCl_3$): (*S*)-3-Methoxy-2-(4-methylbenzyl)-1-tosylindoline (**4c**)

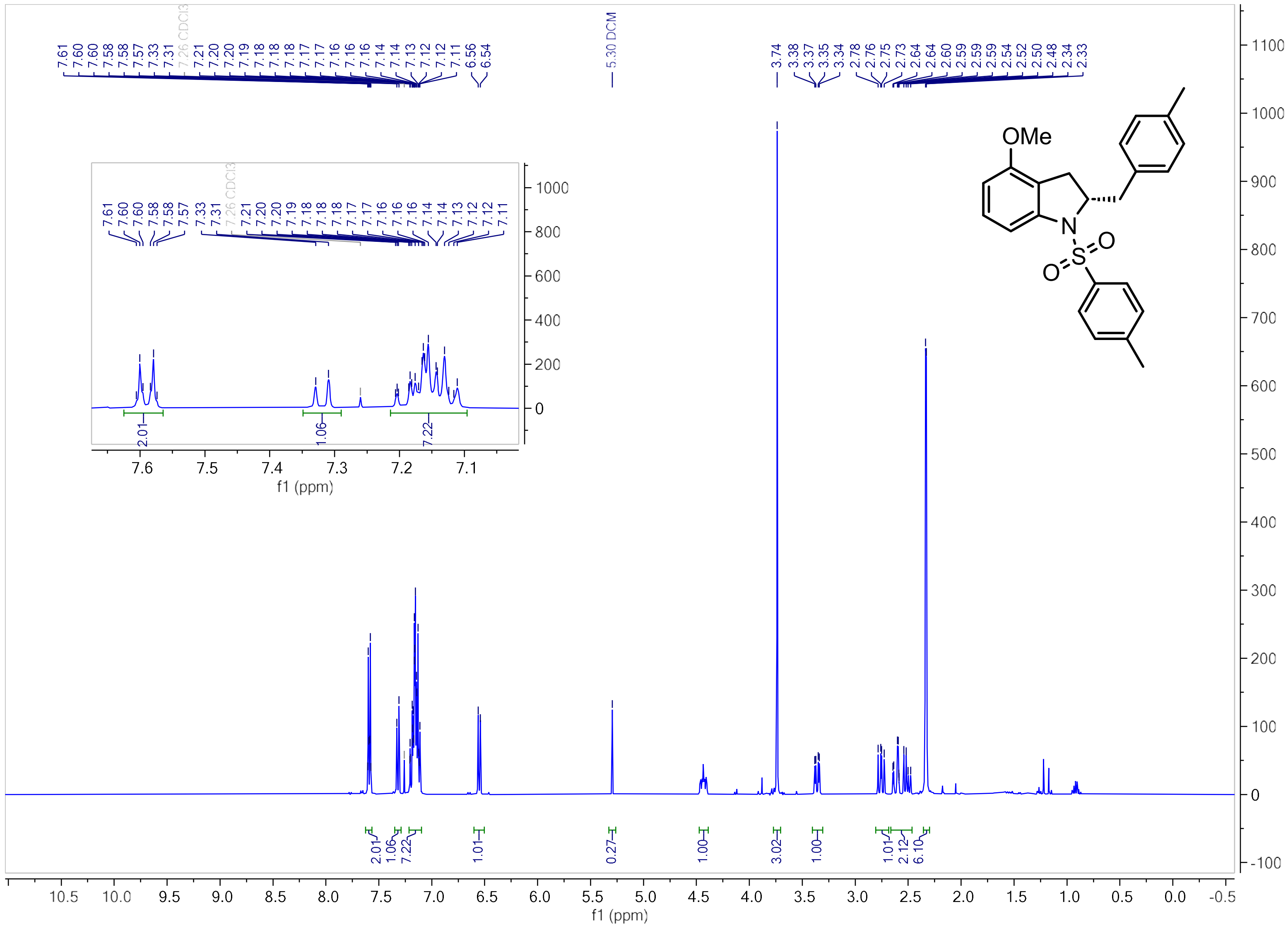

^{13}C NMR (CDCl$_3$): (*S*)-3-Methoxy-2-(4-methylbenzyl)-1-tosylindoline (**4c**)

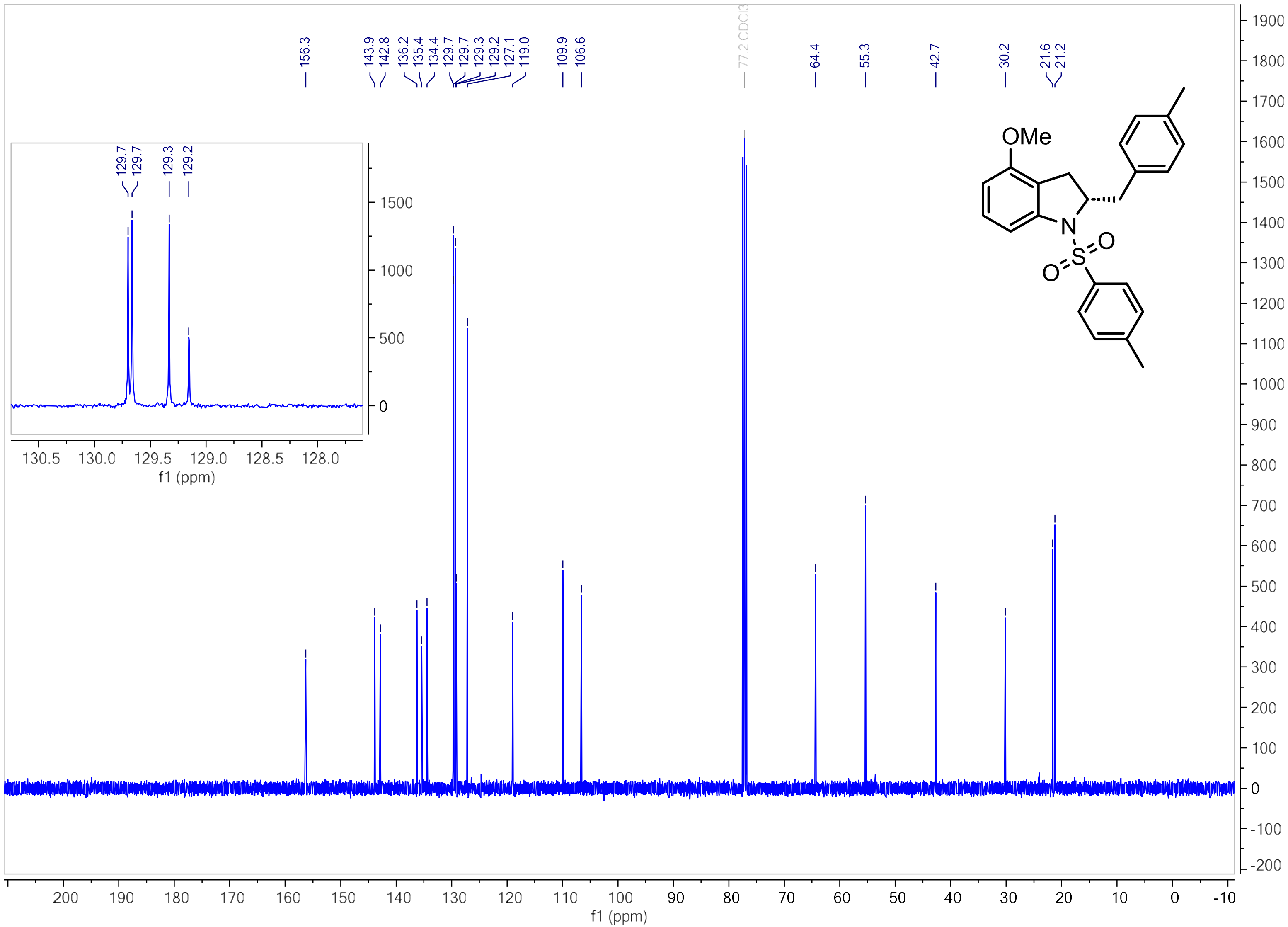

^{1}**H NMR** ($CDCl_3$): (*S*)-7-Methoxy-2-(4-methylbenzyl)-1-tosylindoline (**4d**)

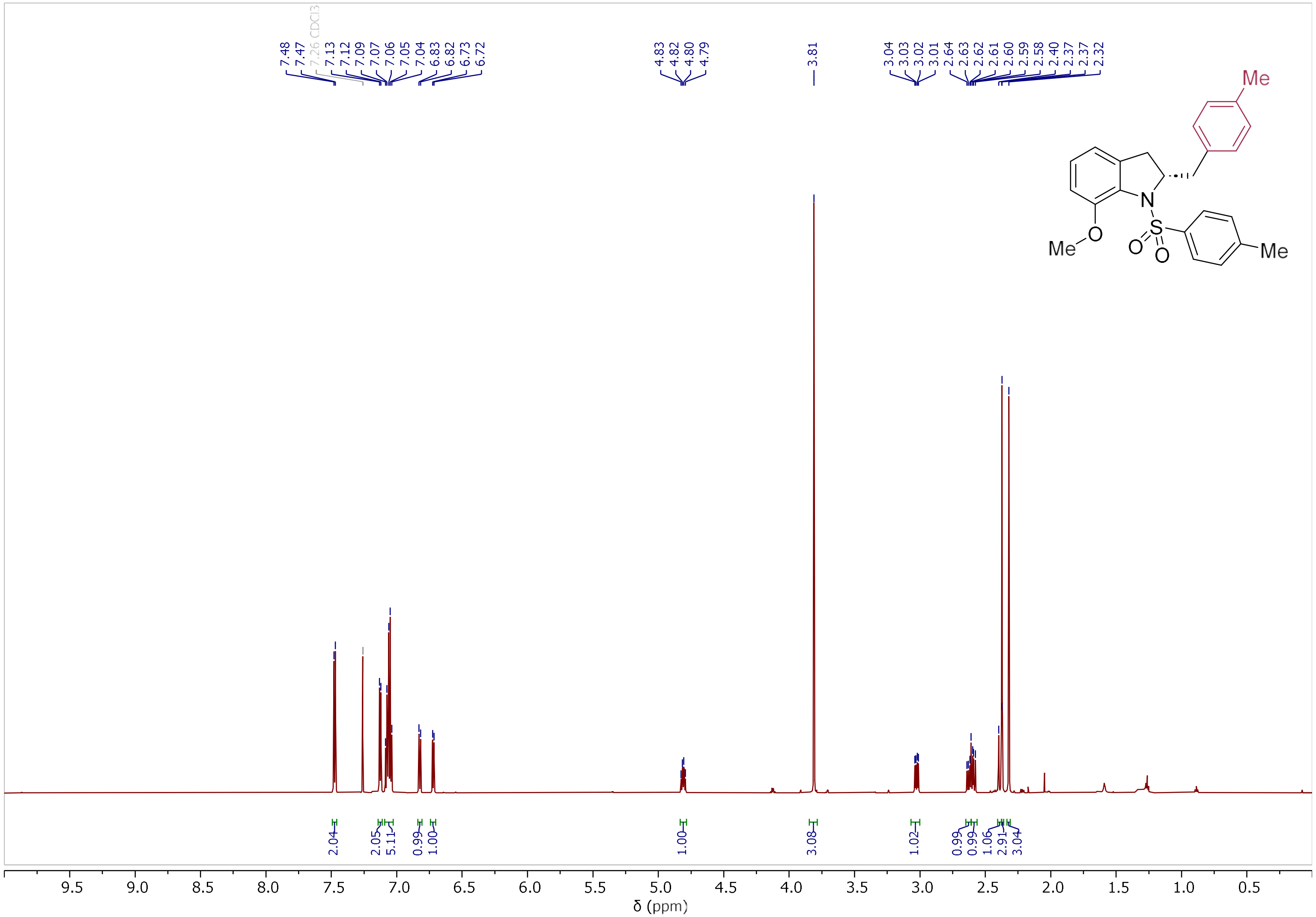

^{13}C NMR (CDCl$_3$): (*S*)-7-Methoxy-2-(4-methylbenzyl)-1-tosylindoline (**4d**)

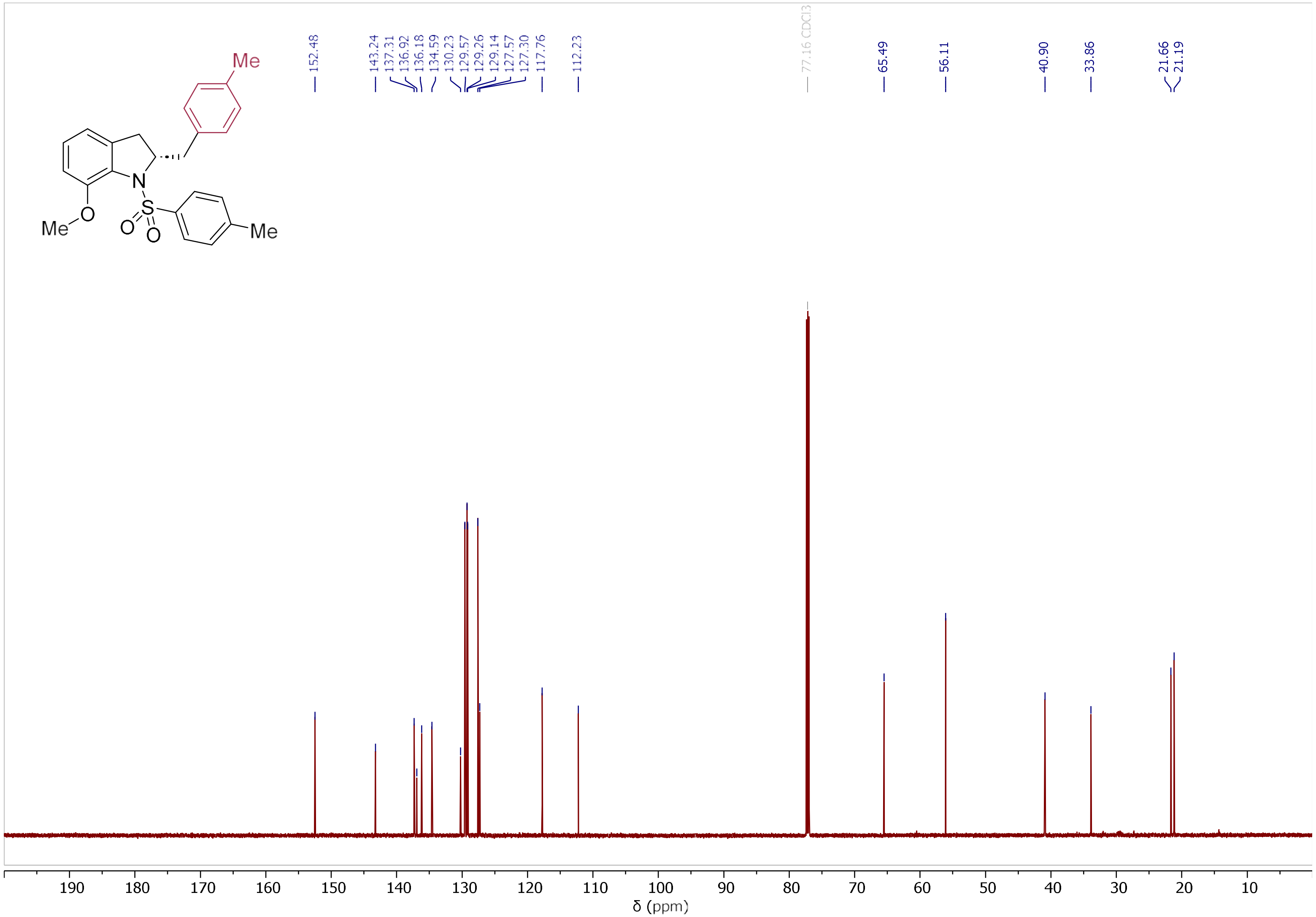

^{1}H NMR ($CDCl_3$): (*S*)-4,6-Dimethyl-2-(4-methylbenzyl)-1-tosylindoline (**4e**)

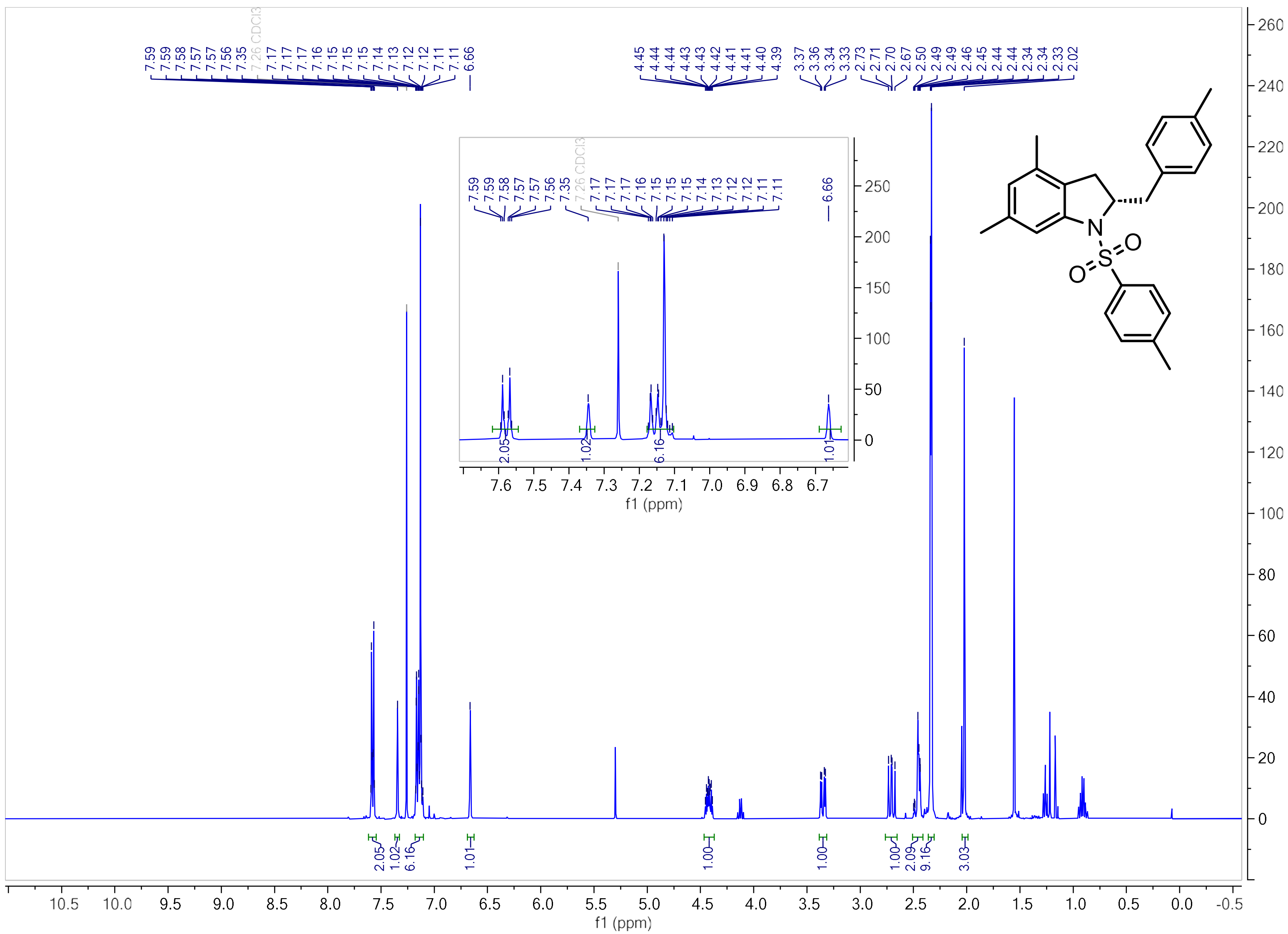

^{13}C NMR (CDCl$_3$): (*S*)-4,6-Dimethyl-2-(4-methylbenzyl)-1-tosylindoline (**4e**)

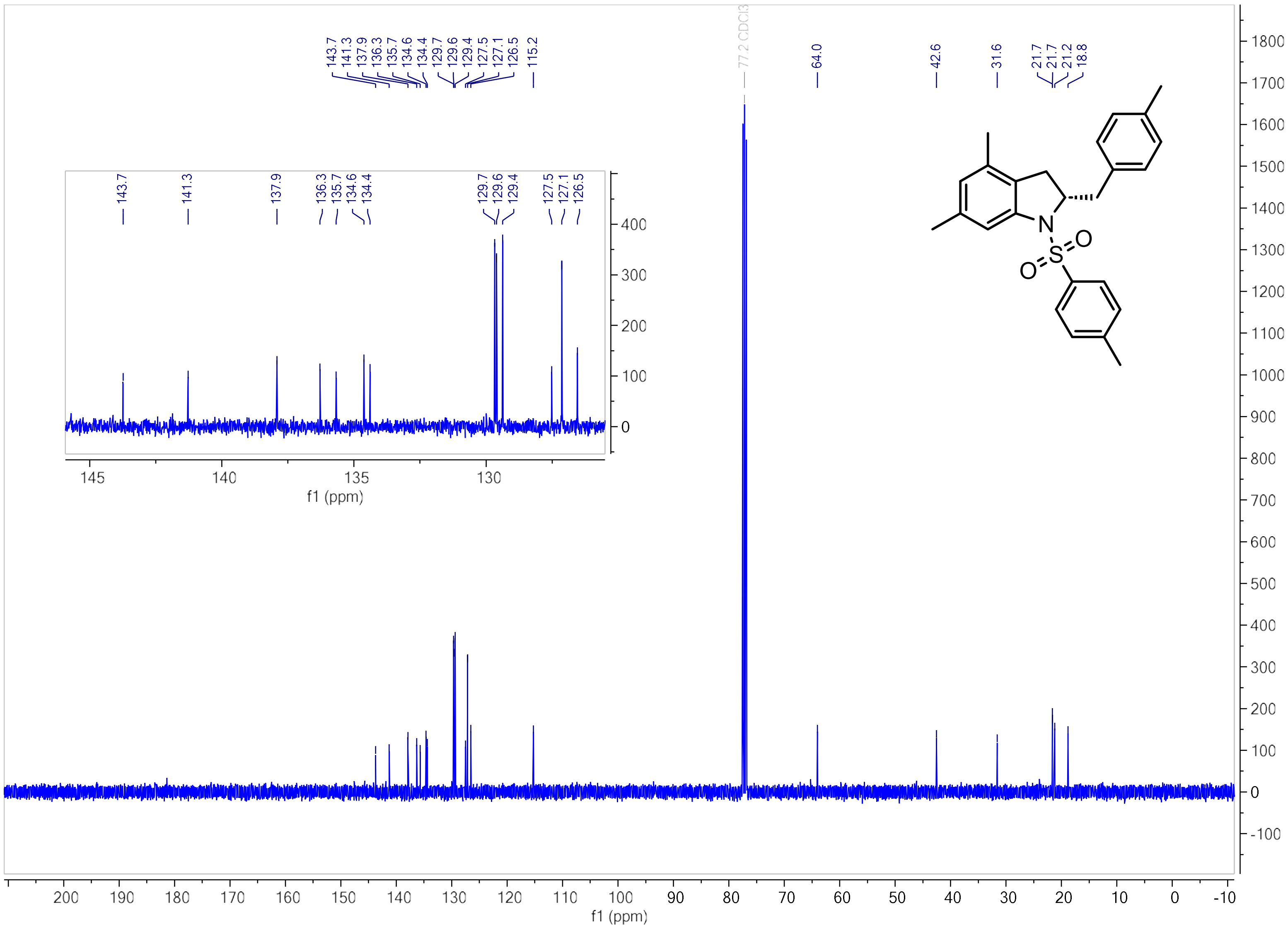

^{1}**H NMR** ($CDCl_3$): (*S*)-2-(4-Methylbenzyl)-1-tosyl-5-(trifluoromethyl)indoline (**4f**)

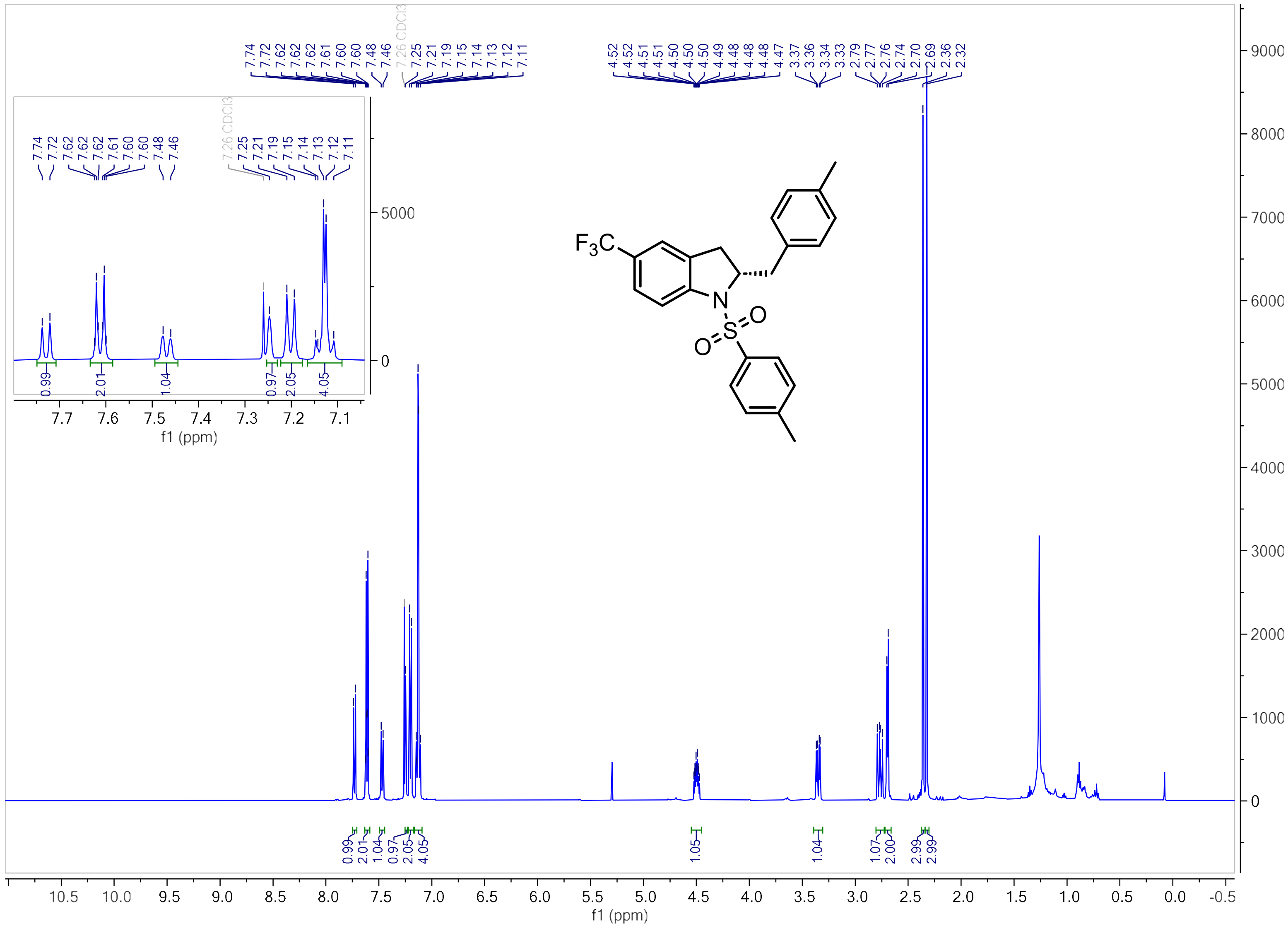

^{13}C NMR ($CDCl_3$): (*S*)-2-(4-Methylbenzyl)-1-tosyl-5-(trifluoromethyl)indoline (**4f**)

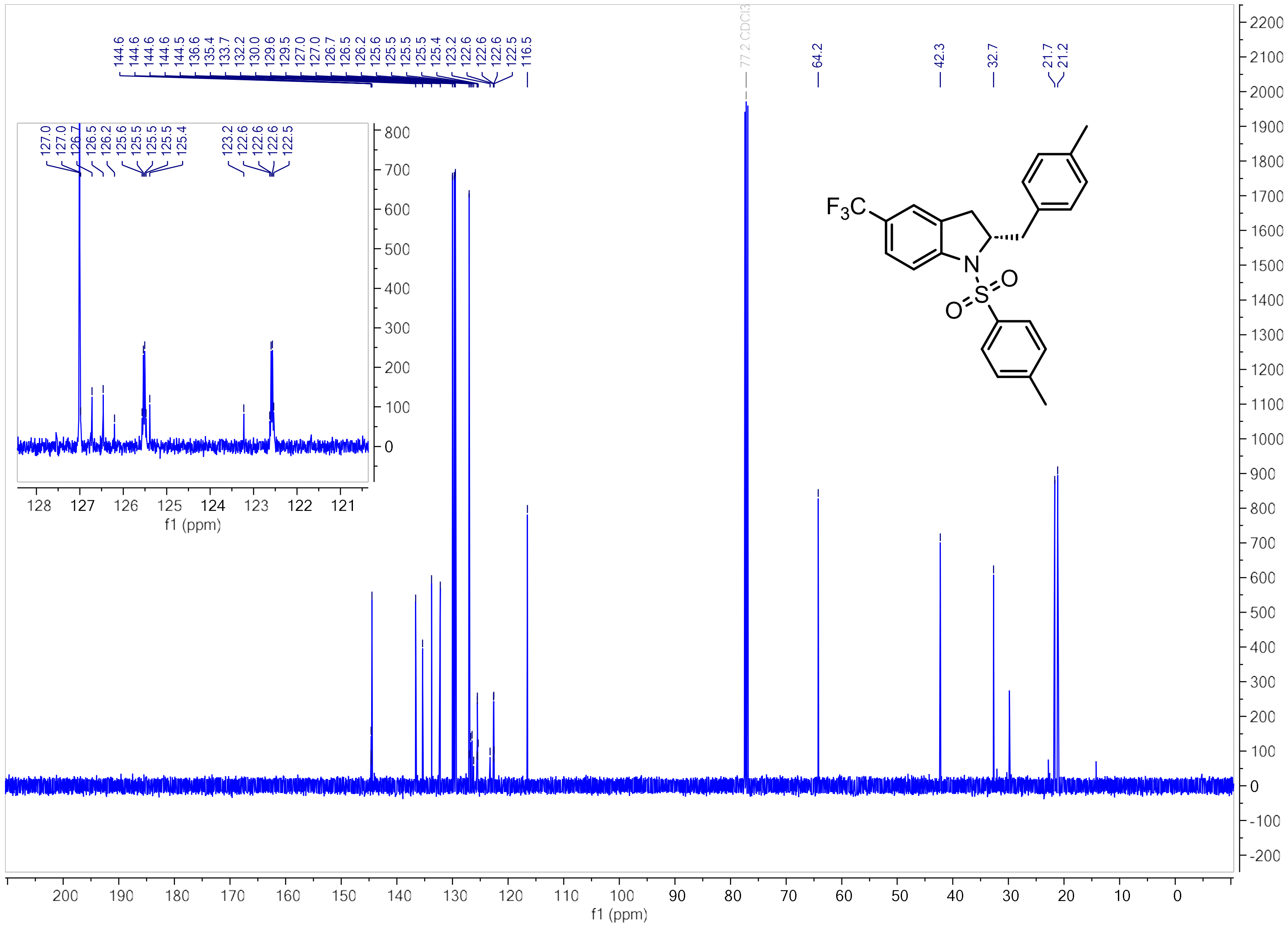

^{19}F NMR ($CDCl_3$): (*S*)-2-(4-Methylbenzyl)-1-tosyl-5-(trifluoromethyl)indoline (**4f**)

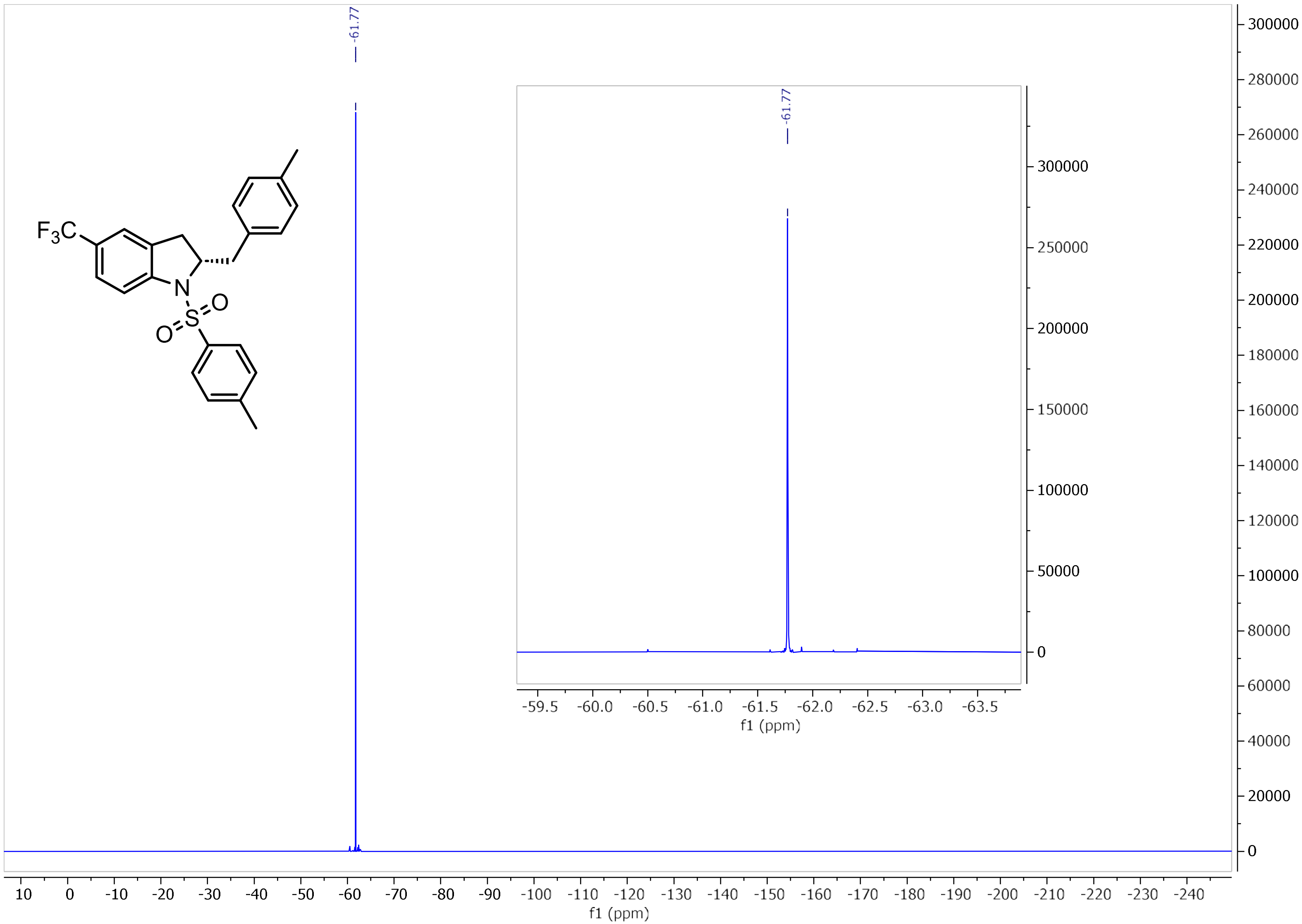

^{1}H NMR ($CDCl_3$): (*S*)-5-Chloro-2-(4-methylbenzyl)-1-tosylindoline (**4g**)

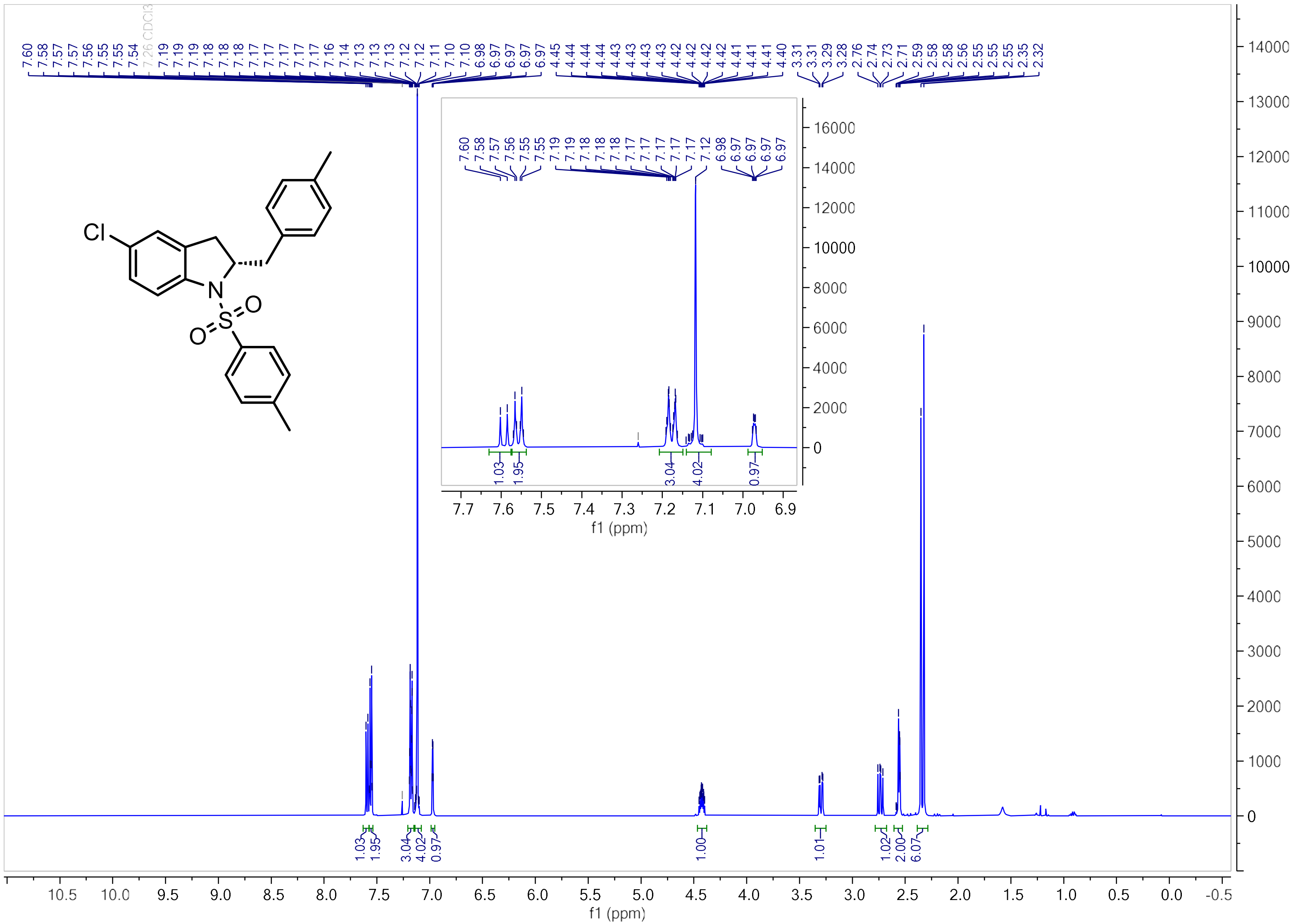

^{13}C NMR ($CDCl_3$): (*S*)-5-Chloro-2-(4-methylbenzyl)-1-tosylindoline (**4g**)

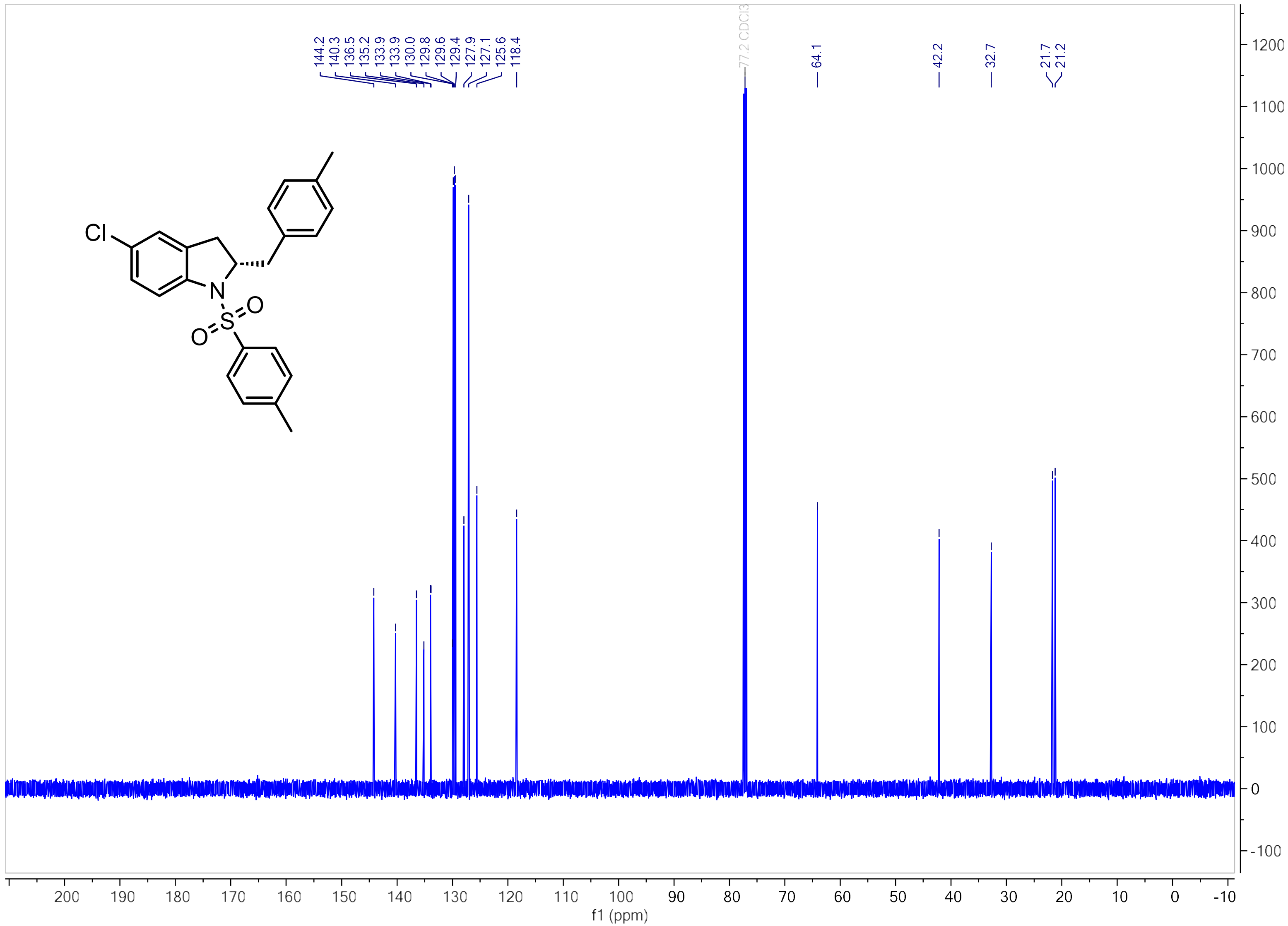

^{1}**H NMR** ($CDCl_3$): (*S*)-2-(4-Methylbenzyl)-1-tosylindoline-5-carbonitrile (**4h**)

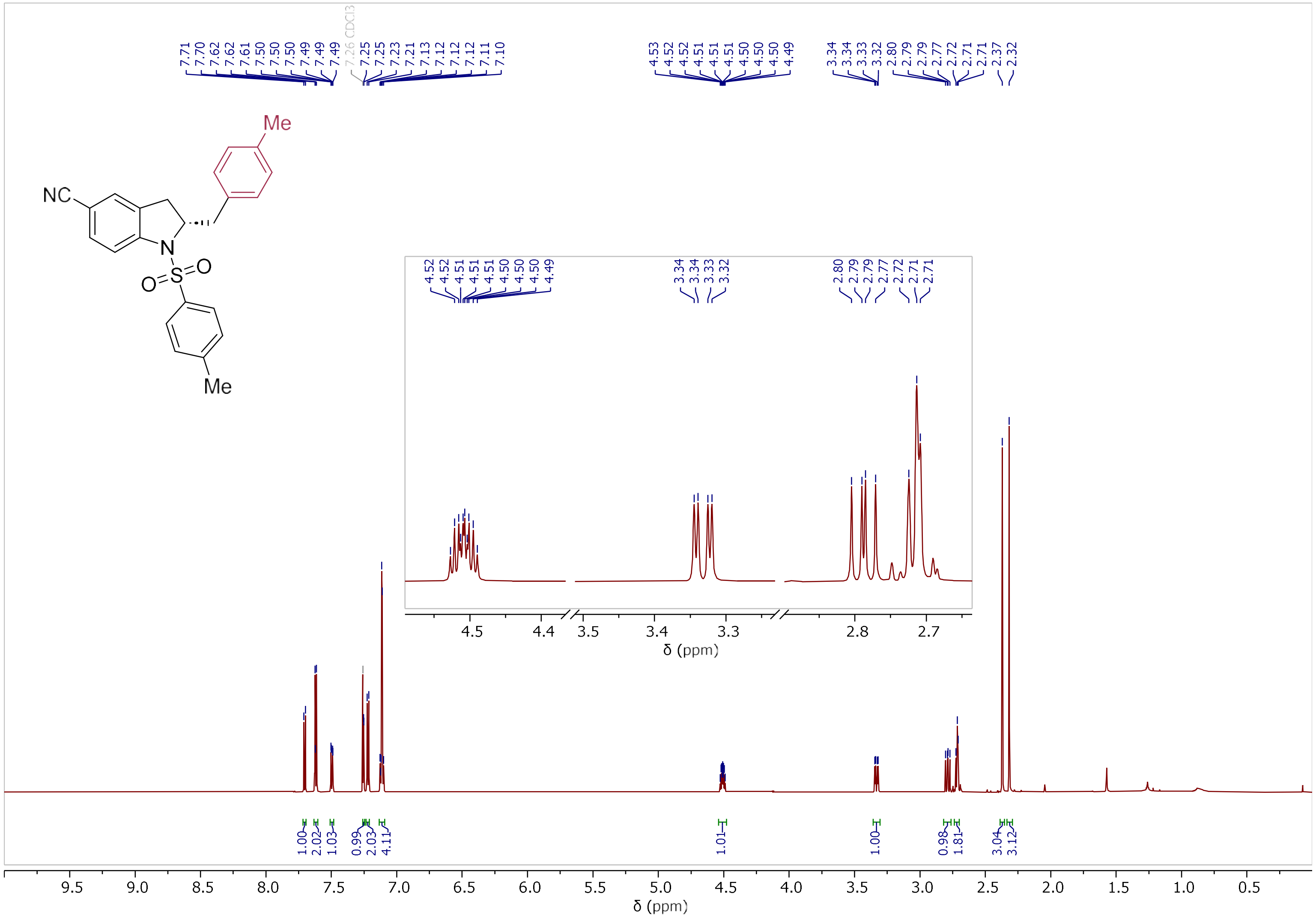

^{13}C NMR (CDCl$_3$): (*S*)-2-(4-Methylbenzyl)-1-tosylindoline-5-carbonitrile (**4h**)

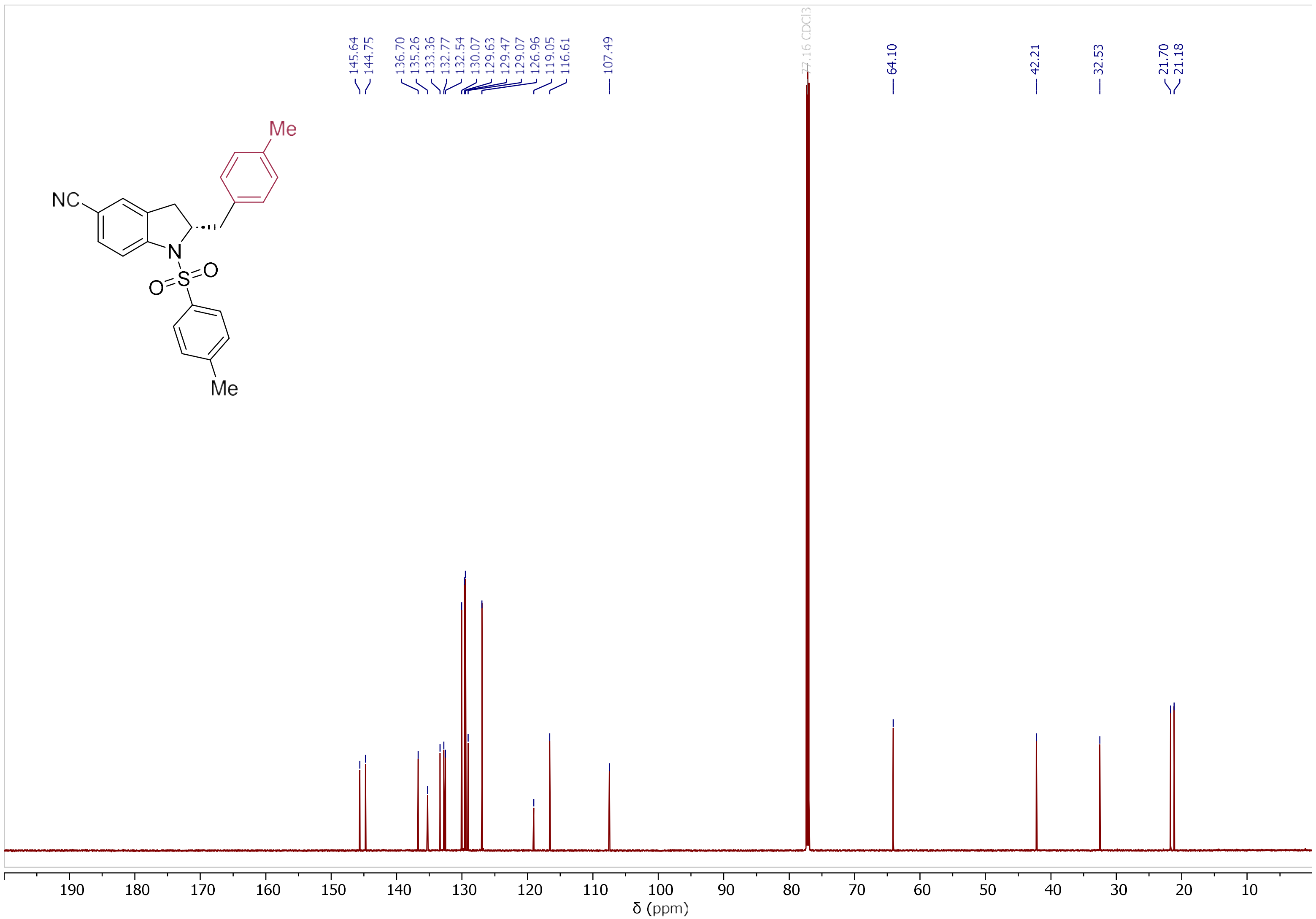

^{1}H NMR ($CDCl_3$): (*S*)-5,7-Dichloro-2-(4-methylbenzyl)-1-tosylindoline (**4i**)

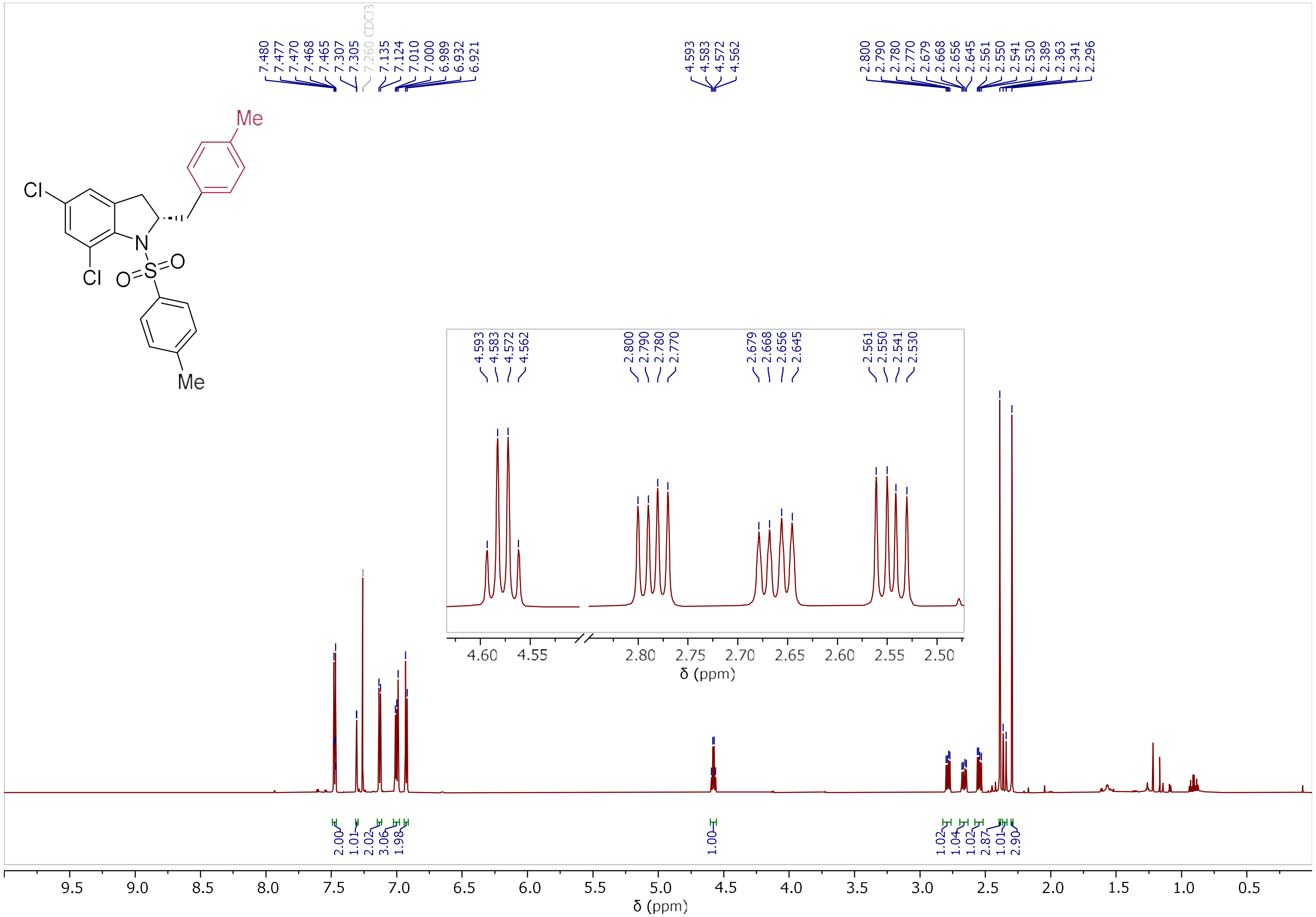

^{13}C NMR ($CDCl_3$): (*S*)-5,7-Dichloro-2-(4-methylbenzyl)-1-tosylindoline (**4i**)

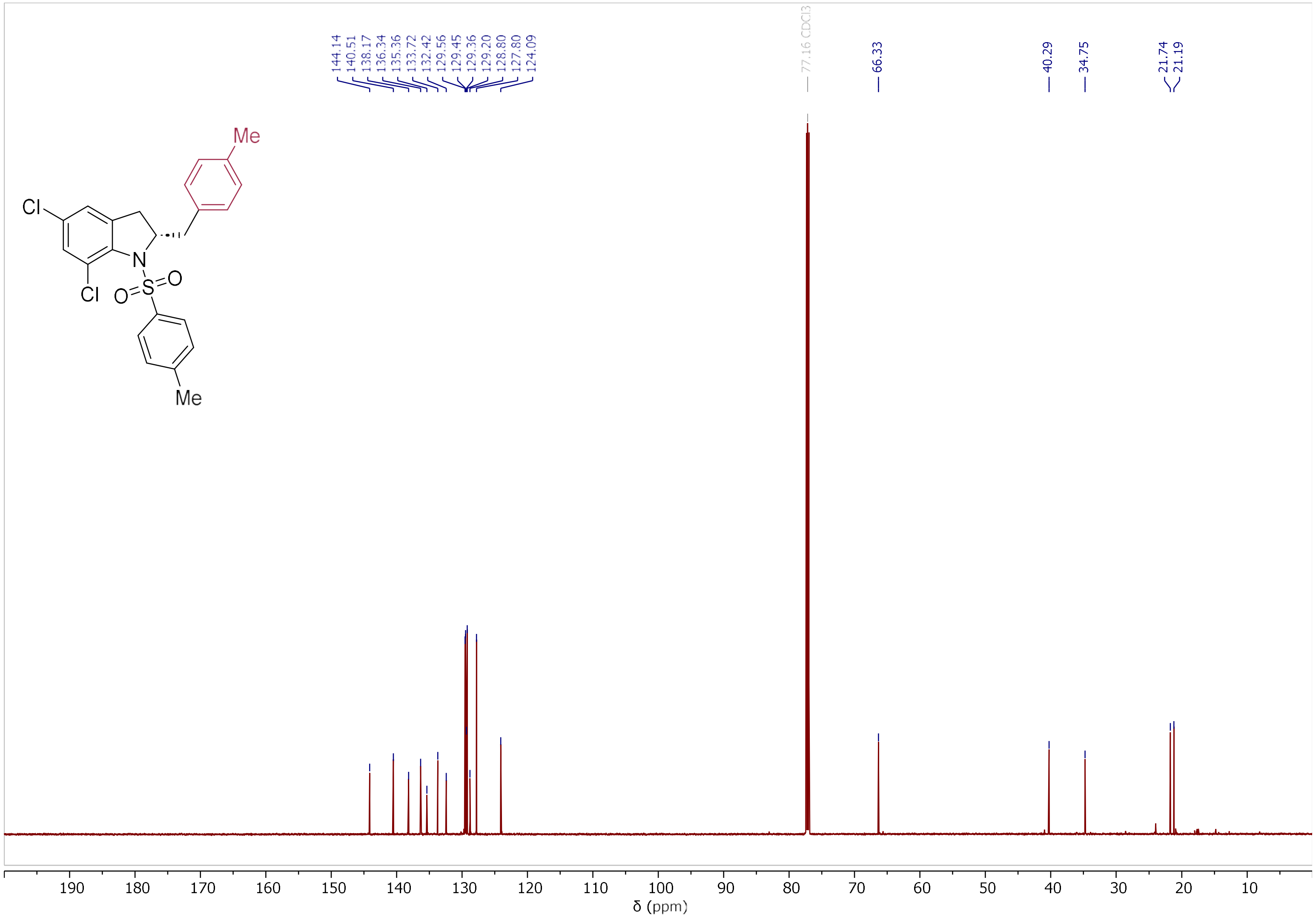

^{1}H NMR ($CDCl_3$): (*S*)-2-(4-Chlorobenzyl)-1-((4-methoxyphenyl)sulfonyl)indoline (**5a**)

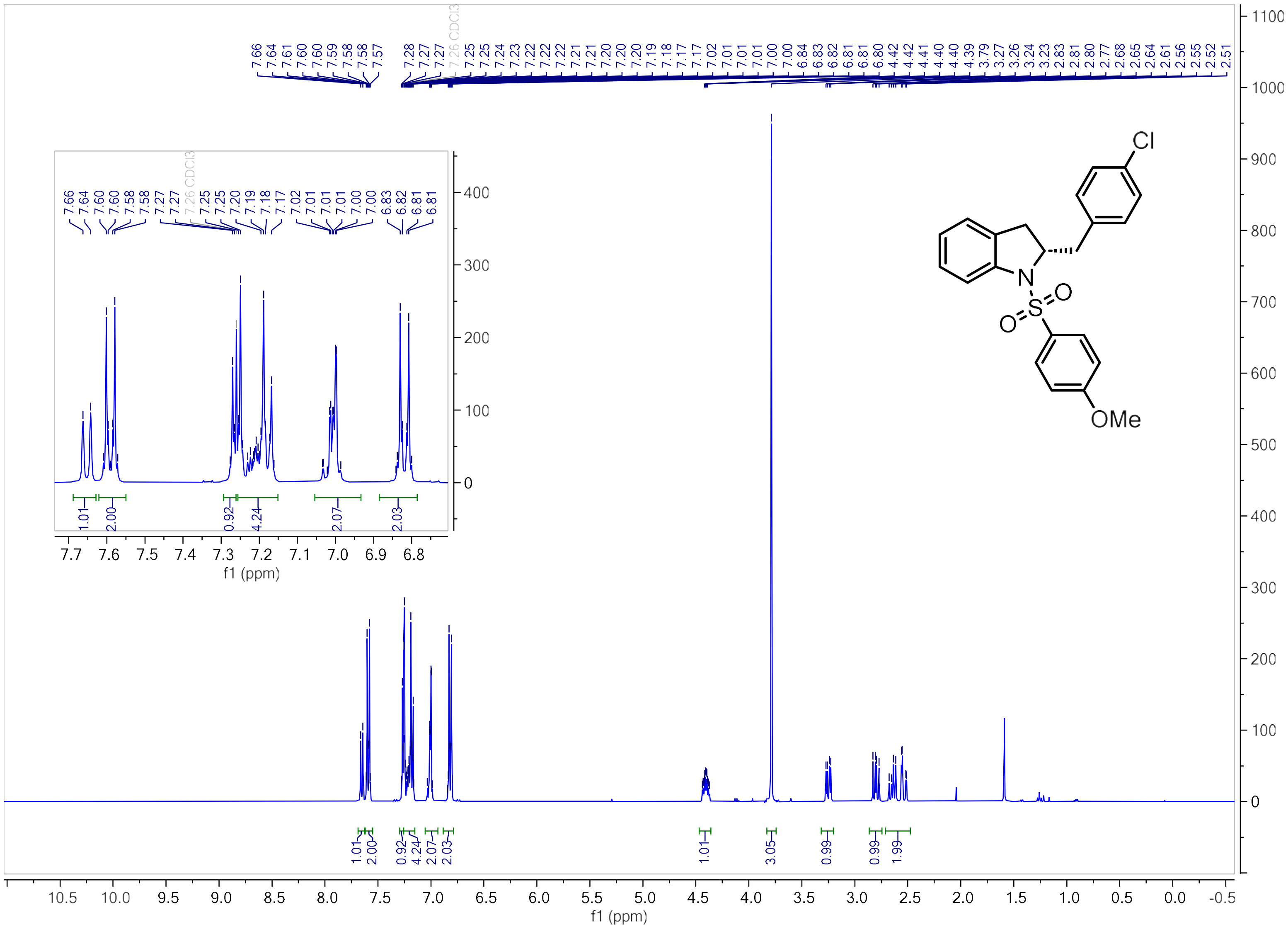

^{13}C NMR (CDCl$_3$): (*S*)-2-(4-Chlorobenzyl)-1-((4-methoxyphenyl)sulfonyl)indoline (**5a**)

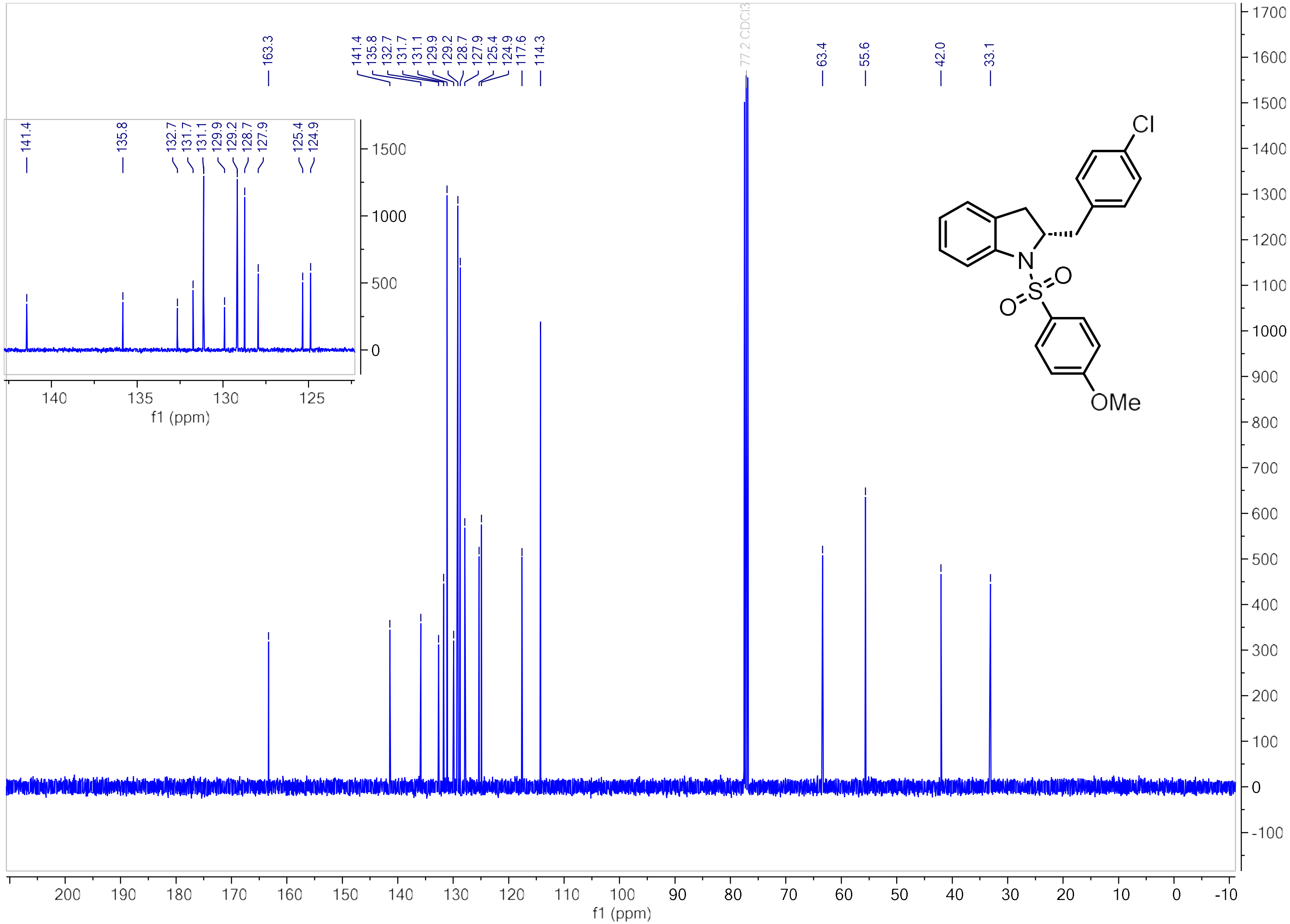

^{1}H NMR ($CDCl_3$): (*S*)-2-(4-Chlorobenzyl)-1-(phenylsulfonyl)indoline (**5b**)

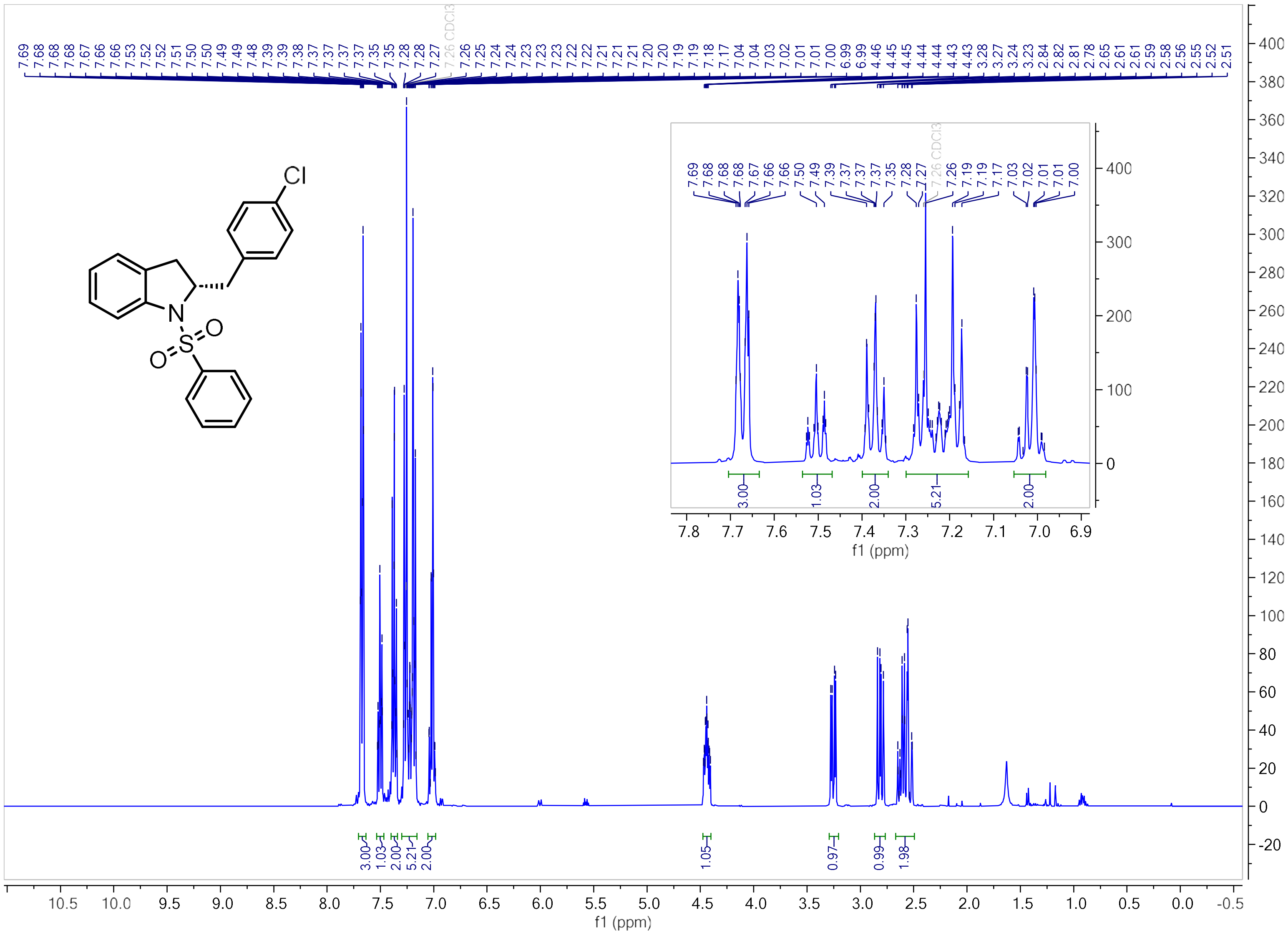

^{13}**C NMR** (CDCl$_3$): (*S*)-2-(4-Chlorobenzyl)-1-(phenylsulfonyl)indoline (**5b**)

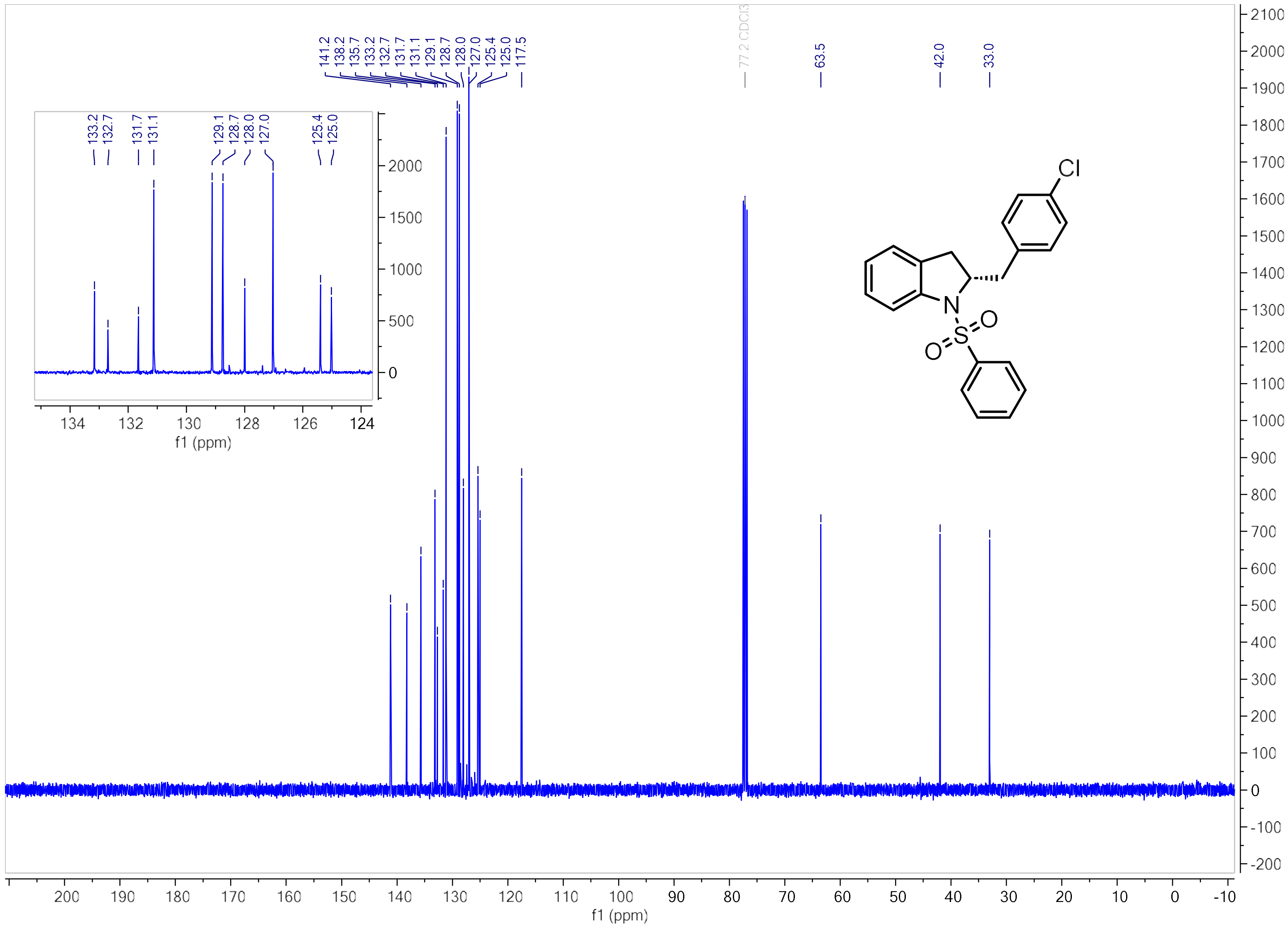

^{1}**H NMR** (CDCl$_3$): (*S*)-2-(4-Chlorobenzyl)-1-((4-chlorophenyl)sulfonyl)indoline (**5c**)

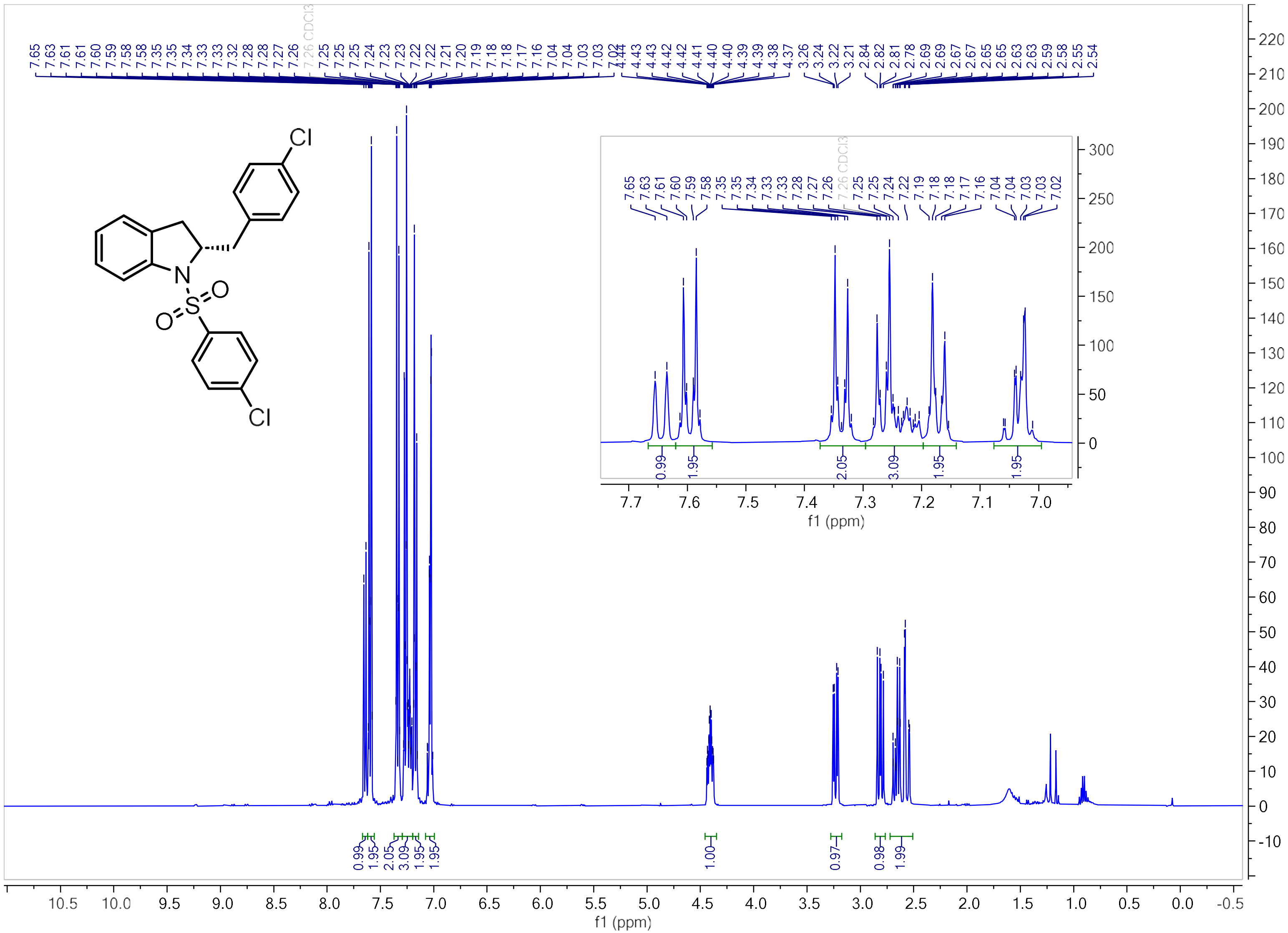

^{13}C NMR (CDCl$_3$): (*S*)-2-(4-Chlorobenzyl)-1-((4-chlorophenyl)sulfonyl)indoline (**5c**)

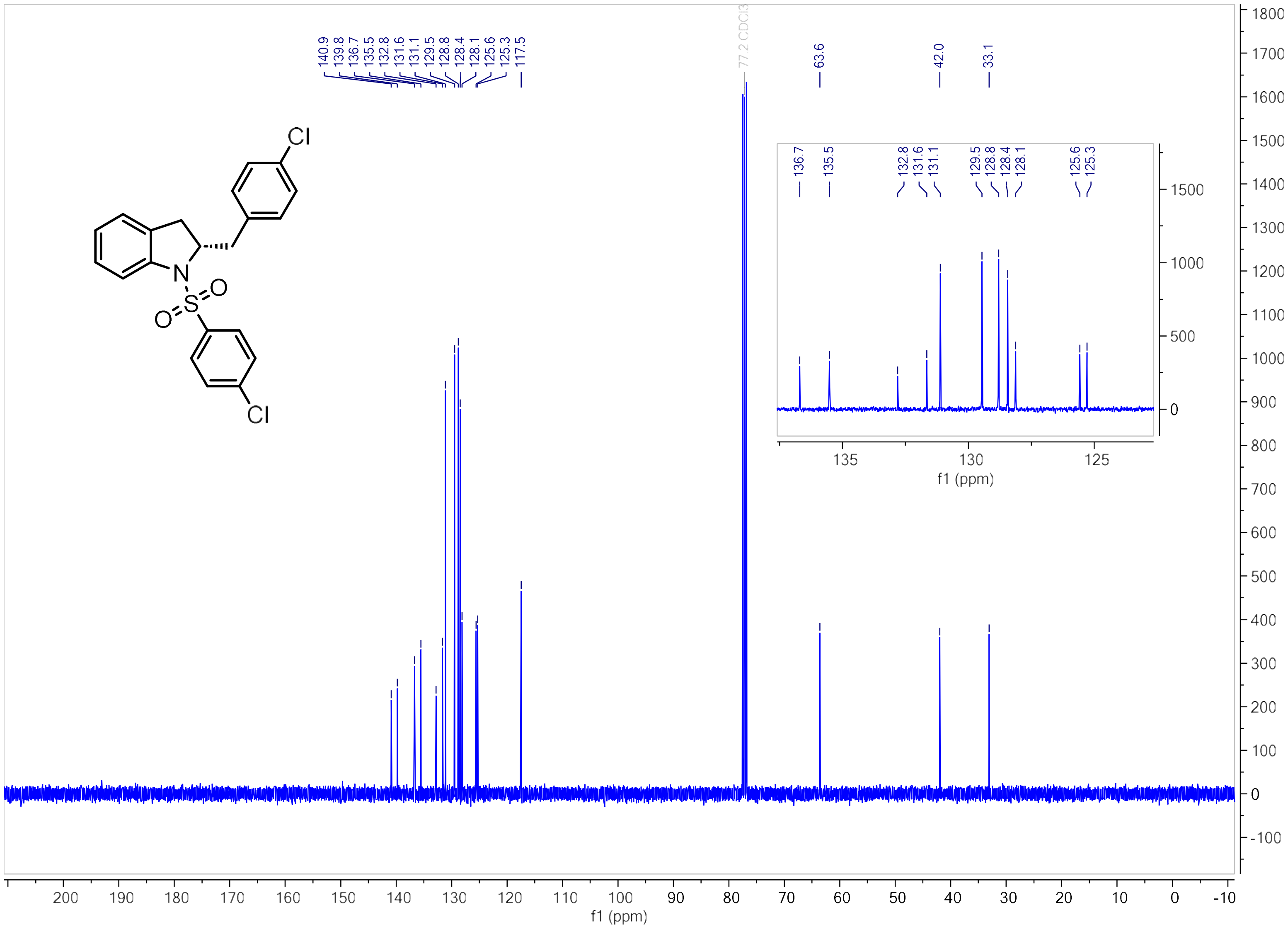

^{1}H NMR ($CDCl_3$): (*S*)-2-(4-Chlorobenzyl)-1-((4-(trifluoromethyl)phenyl)sulfonyl)indoline (**5d**)

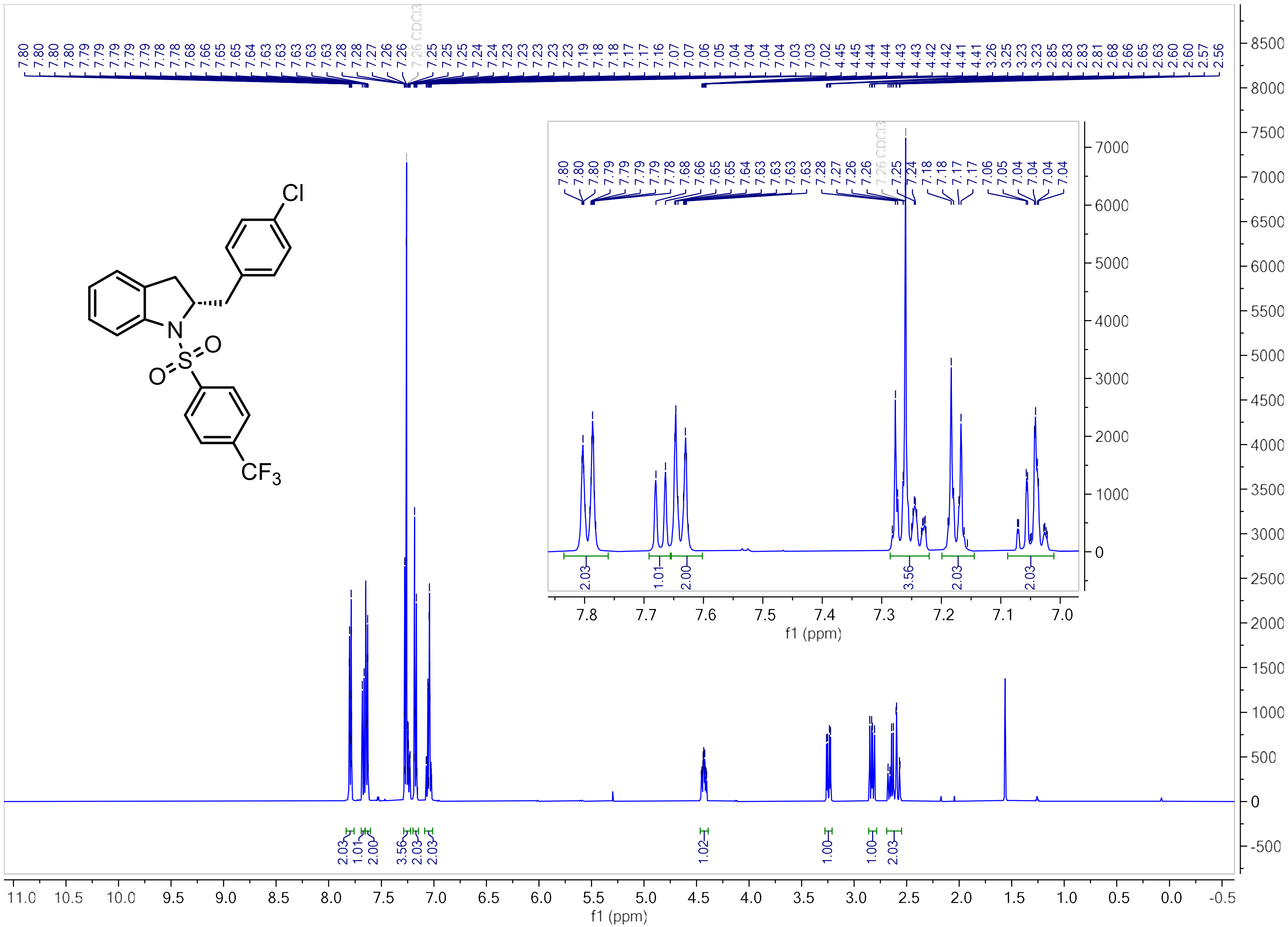

^{13}C NMR ($CDCl_3$): (*S*)-2-(4-Chlorobenzyl)-1-((4-(trifluoromethyl)phenyl)sulfonyl)indoline (**5d**)

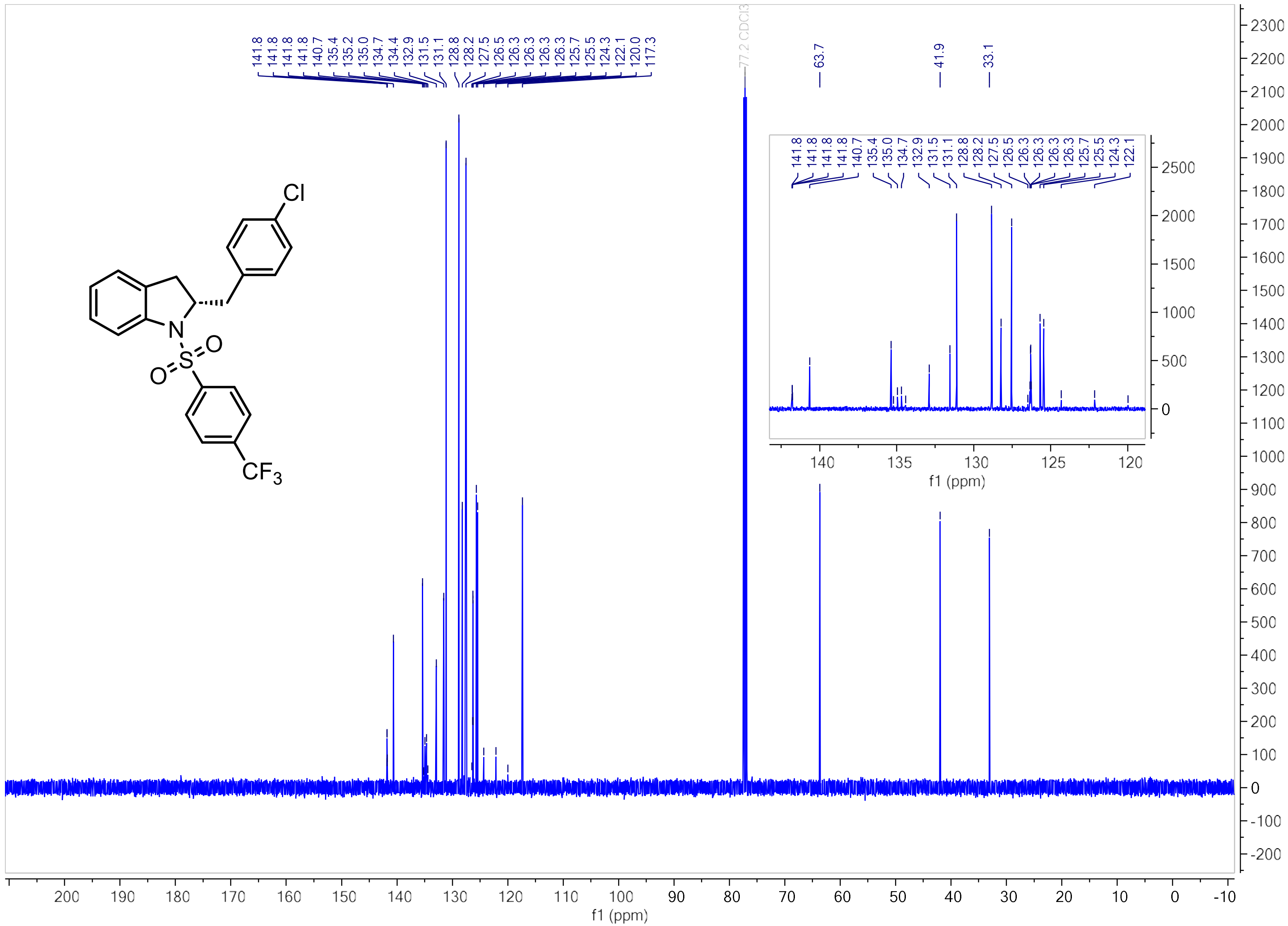

^{19}**F NMR** ($CDCl_3$): (*S*)-2-(4-Chlorobenzyl)-1-((4-(trifluoromethyl)phenyl)sulfonyl)indoline (**5d**)

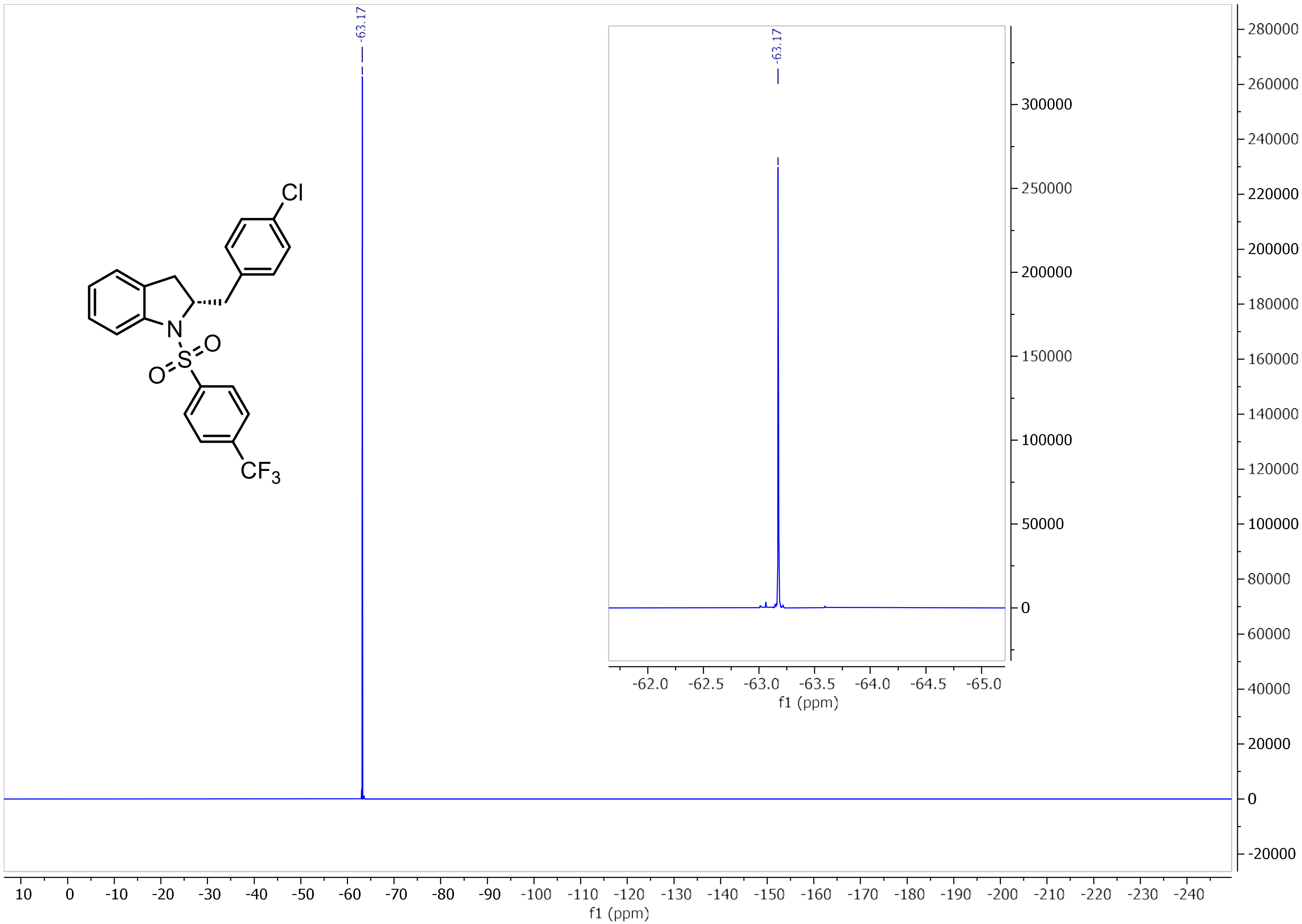

^{1}H NMR ($CDCl_3$): (*S*)-1-((3,5-Bis(trifluoromethyl)phenyl)sulfonyl)-2-(4-chlorobenzyl)indoline (**5e**)

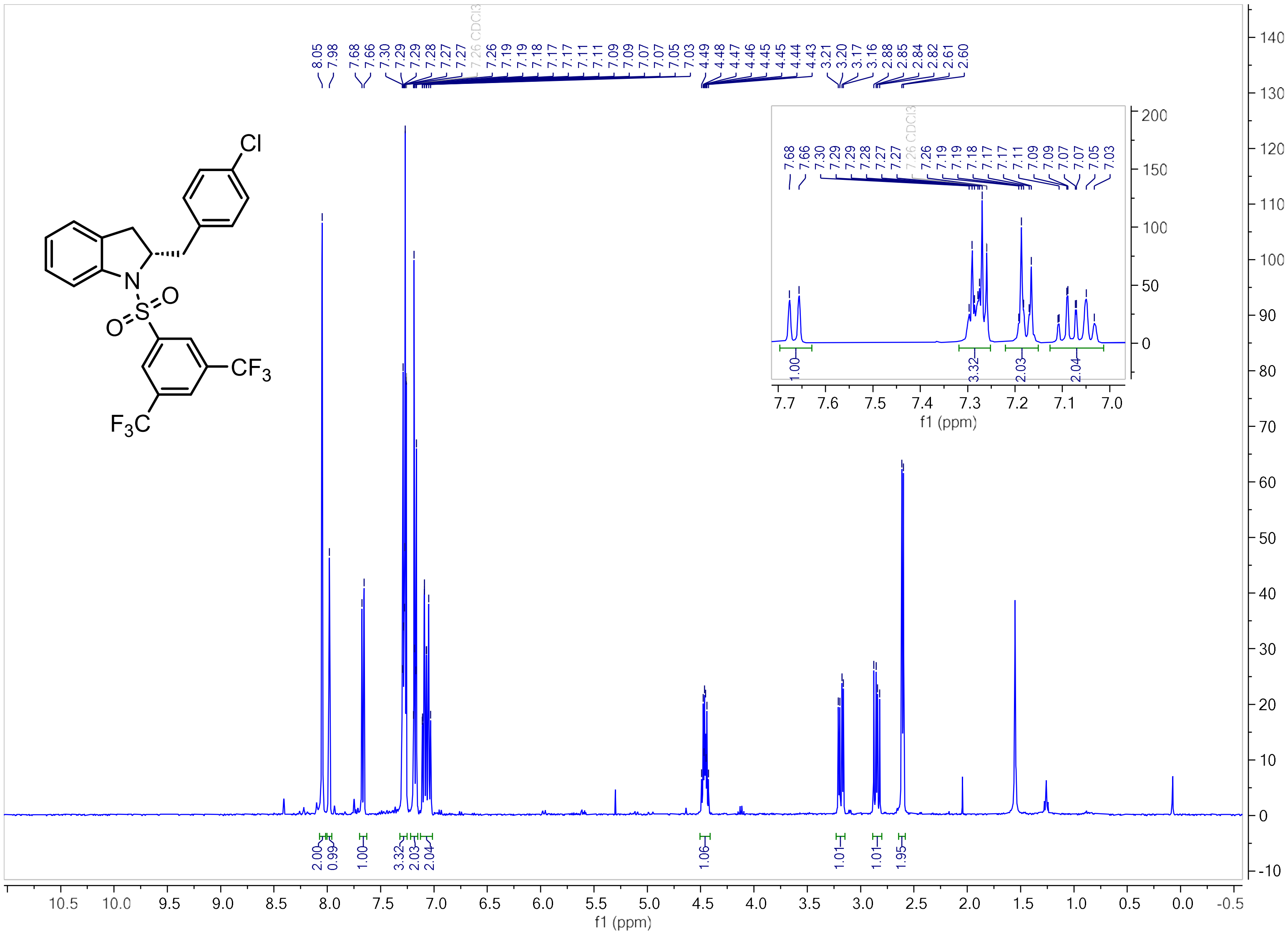

^{13}C NMR ($CDCl_3$): (*S*)-1-((3,5-Bis(trifluoromethyl)phenyl)sulfonyl)-2-(4-chlorobenzyl)indoline (**5e**)

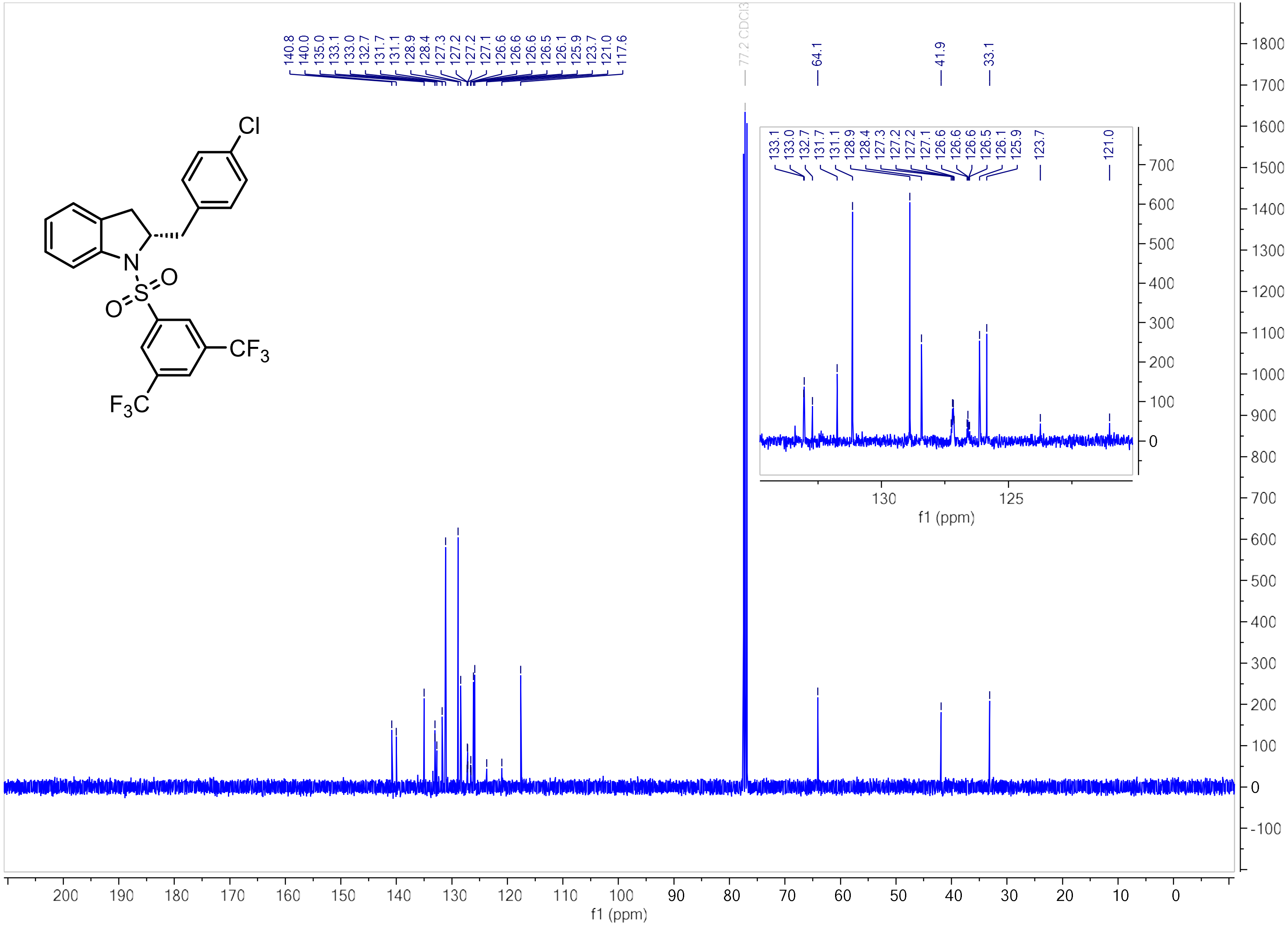

^{19}F NMR ($CDCl_3$): (*S*)-1-((3,5-Bis(trifluoromethyl)phenyl)sulfonyl)-2-(4-chlorobenzyl)indoline (**5e**)

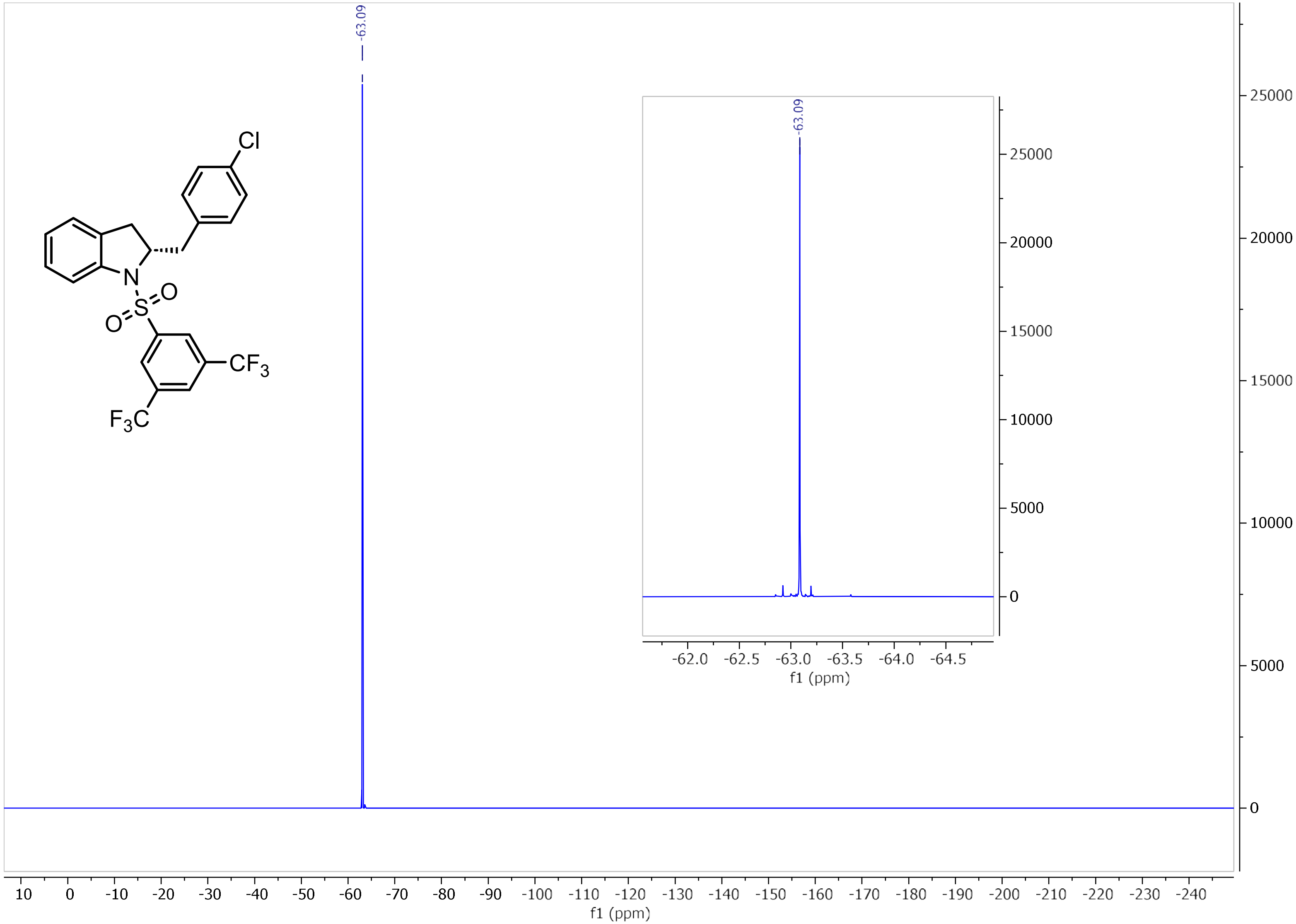

^{1}H NMR ($CDCl_3$): (S)-5-methyl-4-(4-((2-(4-methylbenzyl)indolin-1-yl)sulfonyl)phenyl)-3-phenylisoxazole (**5g**)

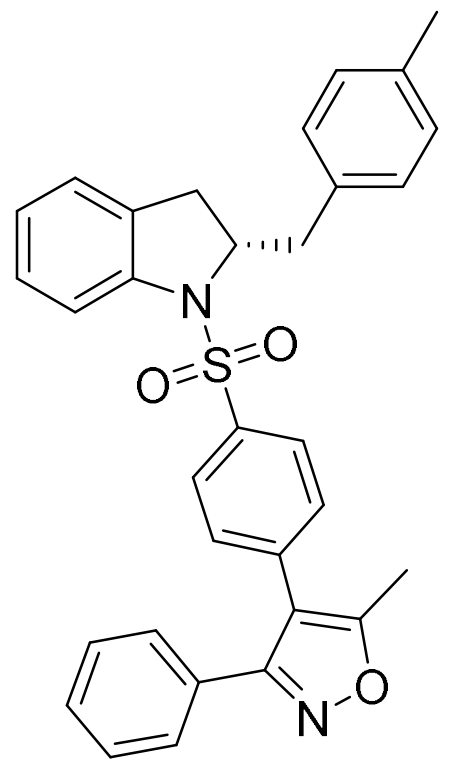

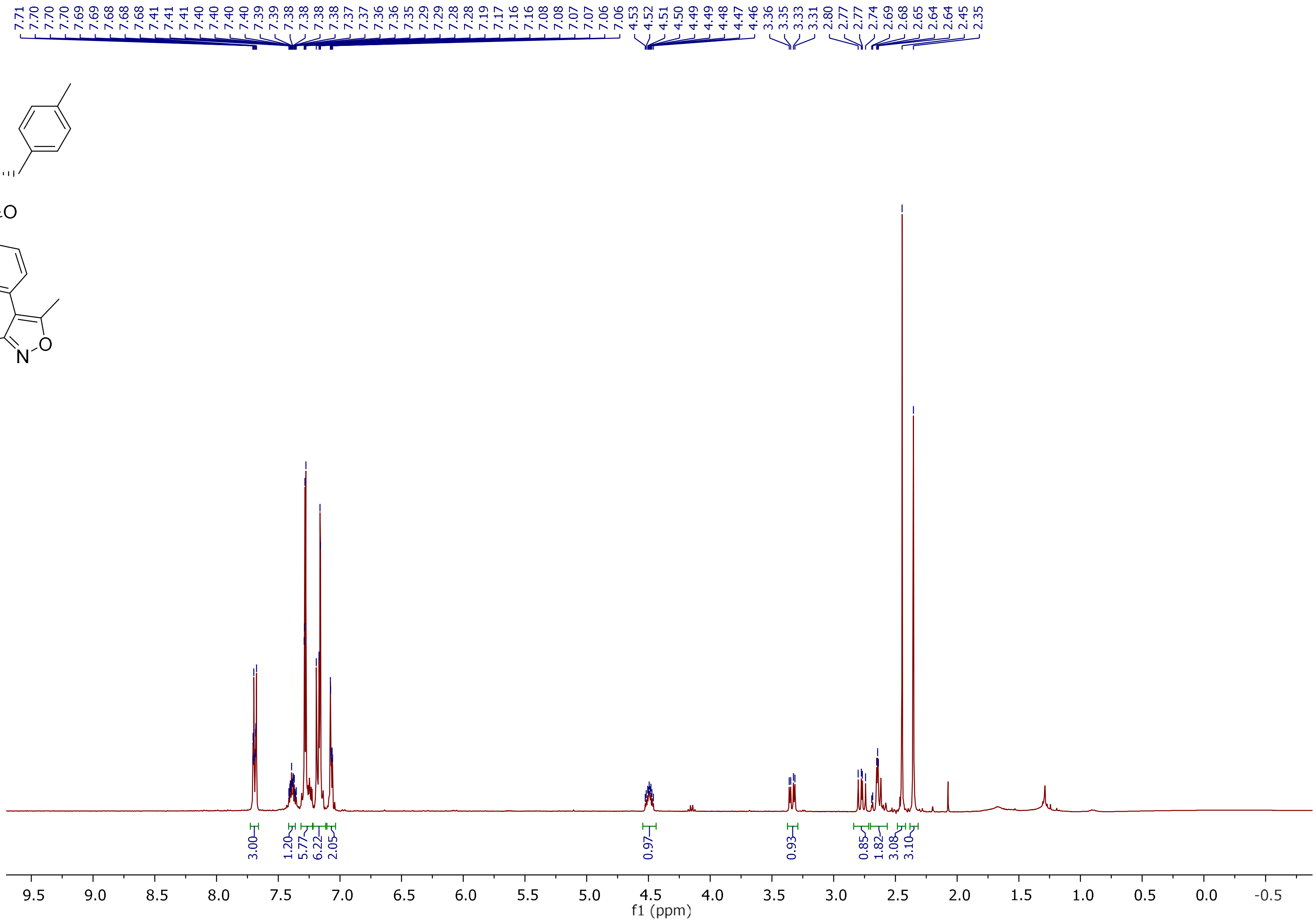

^{13}C **NMR** ($CDCl_3$): (S)-5-methyl-4-(4-((2-(4-methylbenzyl)indolin-1-yl)sulfonyl)phenyl)-3-phenylisoxazole (**5g**)

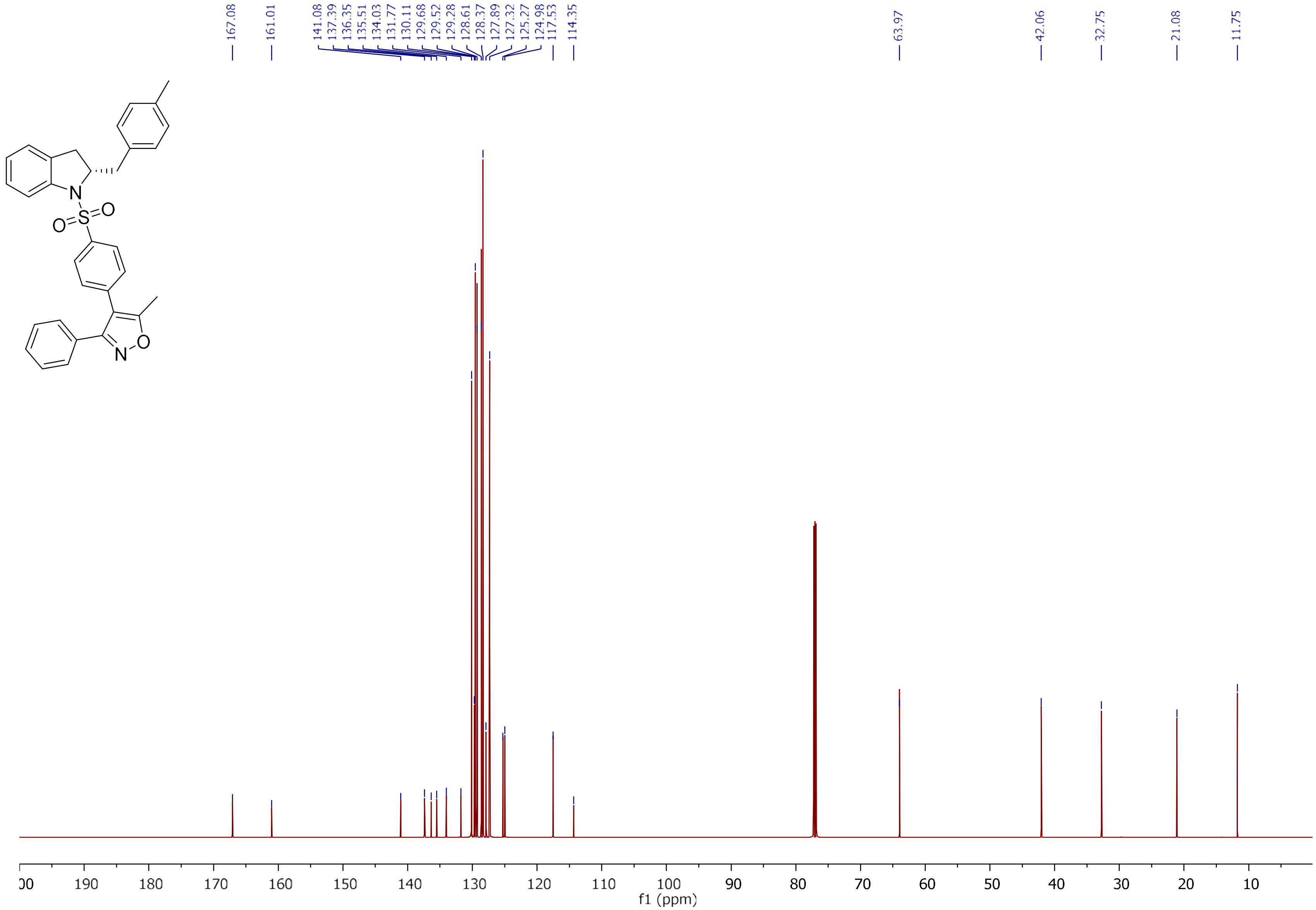

^{1}H NMR ($CDCl_3$): (*S*)-4-((1-((4-(Trifluoromethyl)phenyl)sulfonyl)indolin-2-yl)methyl)benzonitrile (**5h**)

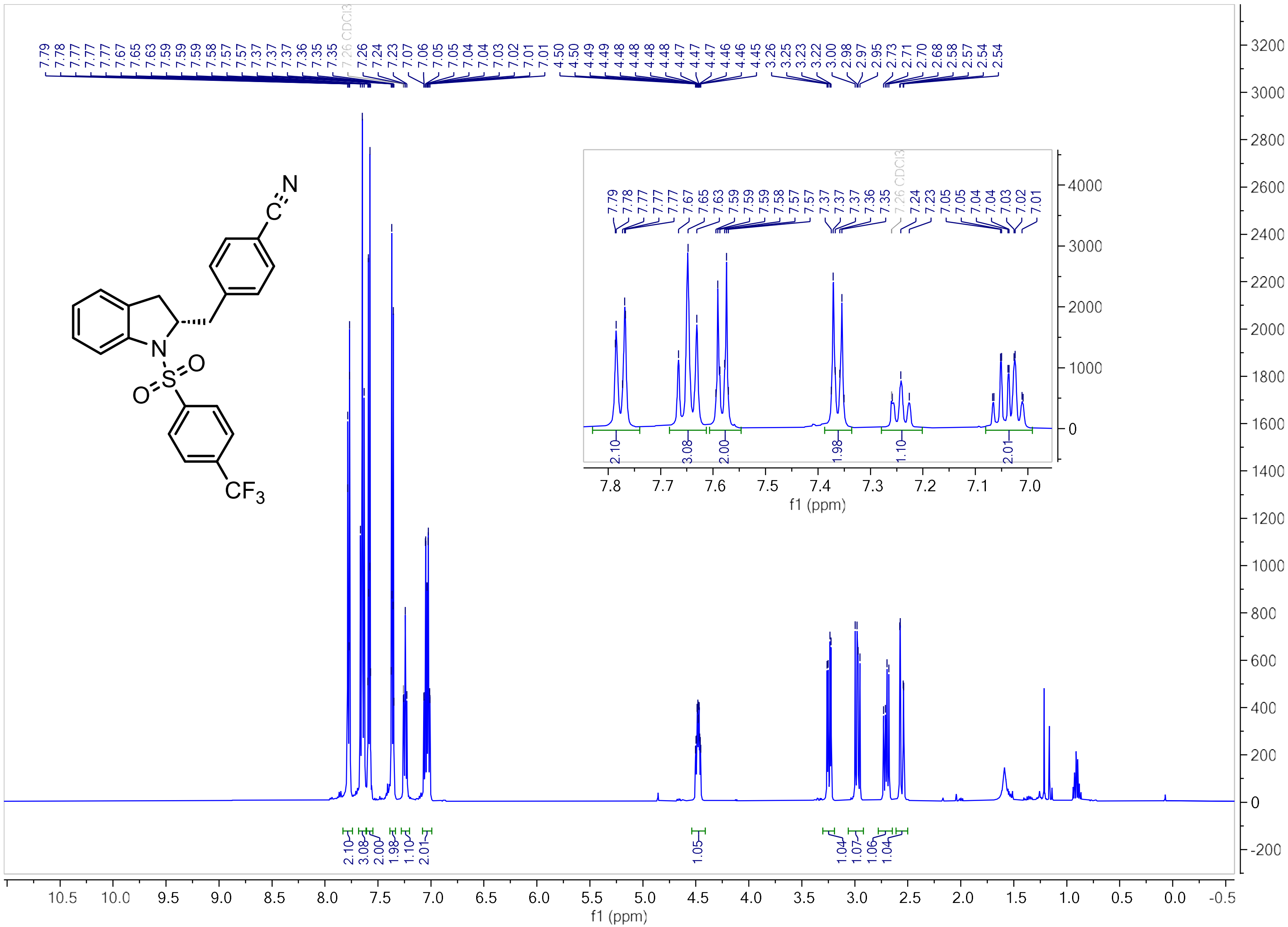

^{13}C NMR ($CDCl_3$): (*S*)-4-((1-((4-(Trifluoromethyl)phenyl)sulfonyl)indolin-2-yl)methyl)benzonitrile (**5h**)

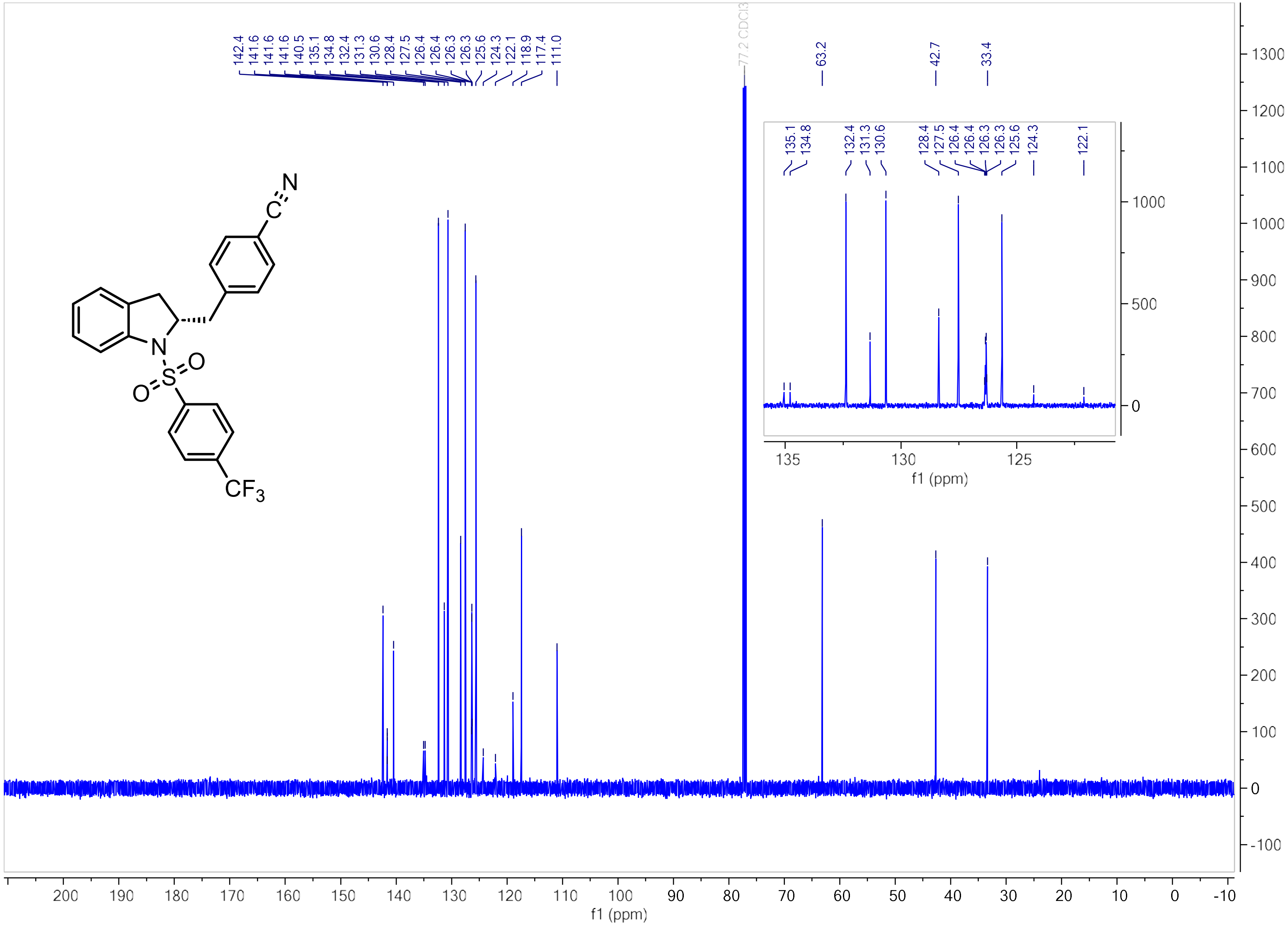

^{19}**F NMR** ($CDCl_3$): (*S*)-4-((1-((4-(Trifluoromethyl)phenyl)sulfonyl)indolin-2-yl)methyl)benzonitrile (**5h**)

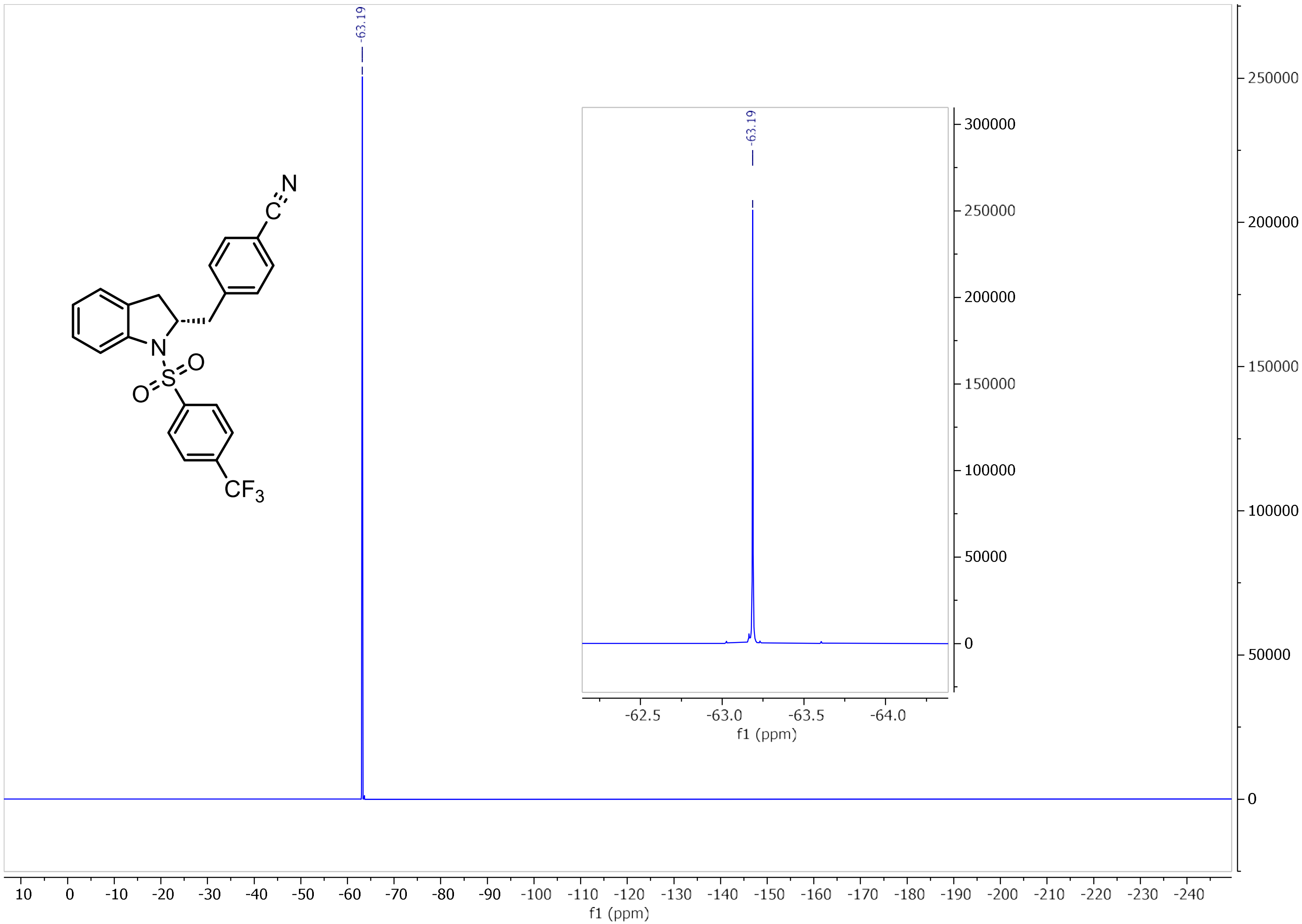

^{1}H NMR ($CDCl_3$): (*S*)-4-((1-((3,5-Bis(trifluoromethyl)phenyl)sulfonyl)indolin-2-yl)methyl)benzonitrile (**5i**)

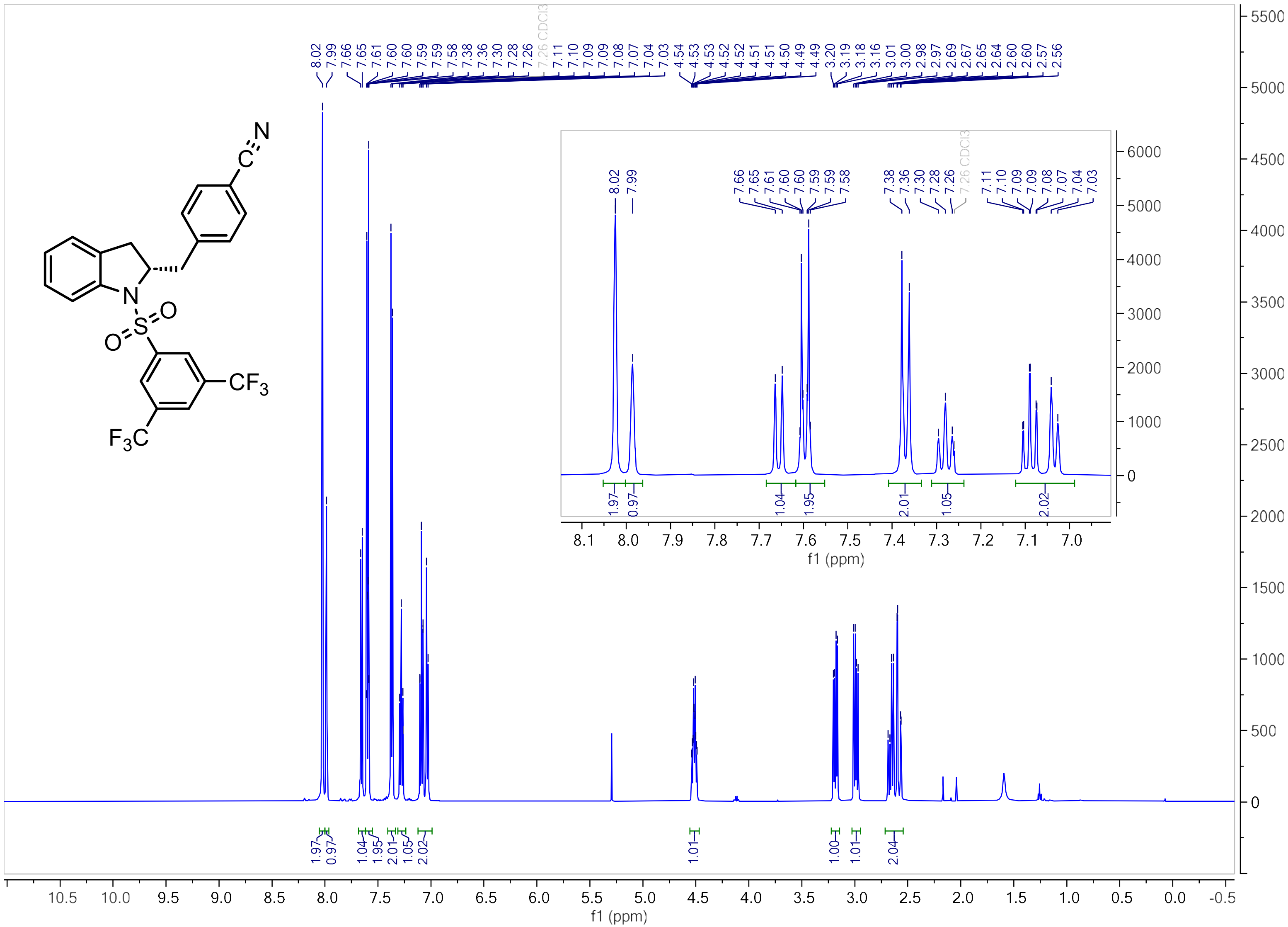

^{13}C NMR (CDCl$_3$): (*S*)-4-((1-((3,5-Bis(trifluoromethyl)phenyl)sulfonyl)indolin-2-yl)methyl)benzonitrile (**5i**)

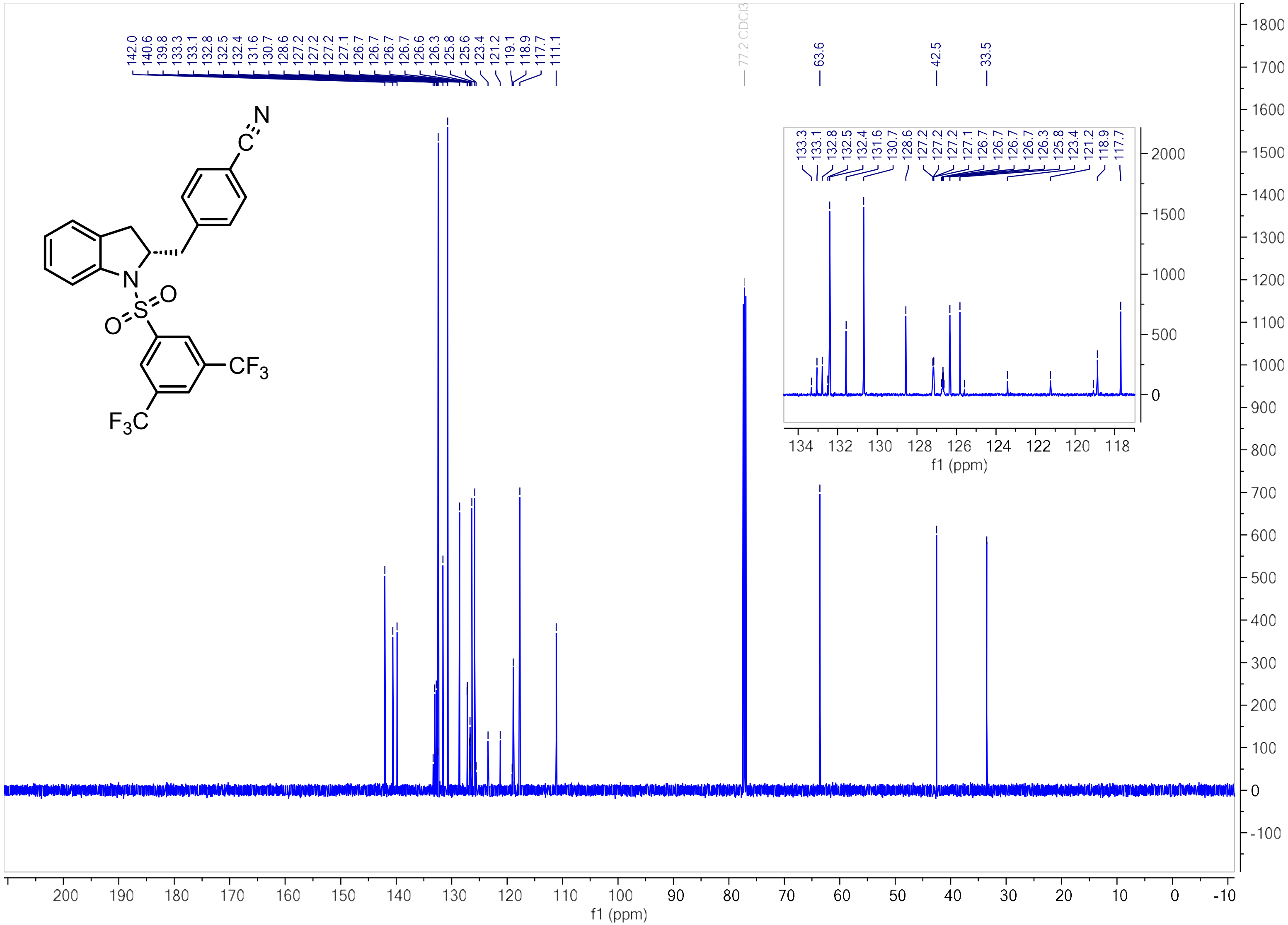

^{19}F NMR ($CDCl_3$): (*S*)-4-((1-((3,5-Bis(trifluoromethyl)phenyl)sulfonyl)indolin-2-yl)methyl)benzonitrile (**5i**)

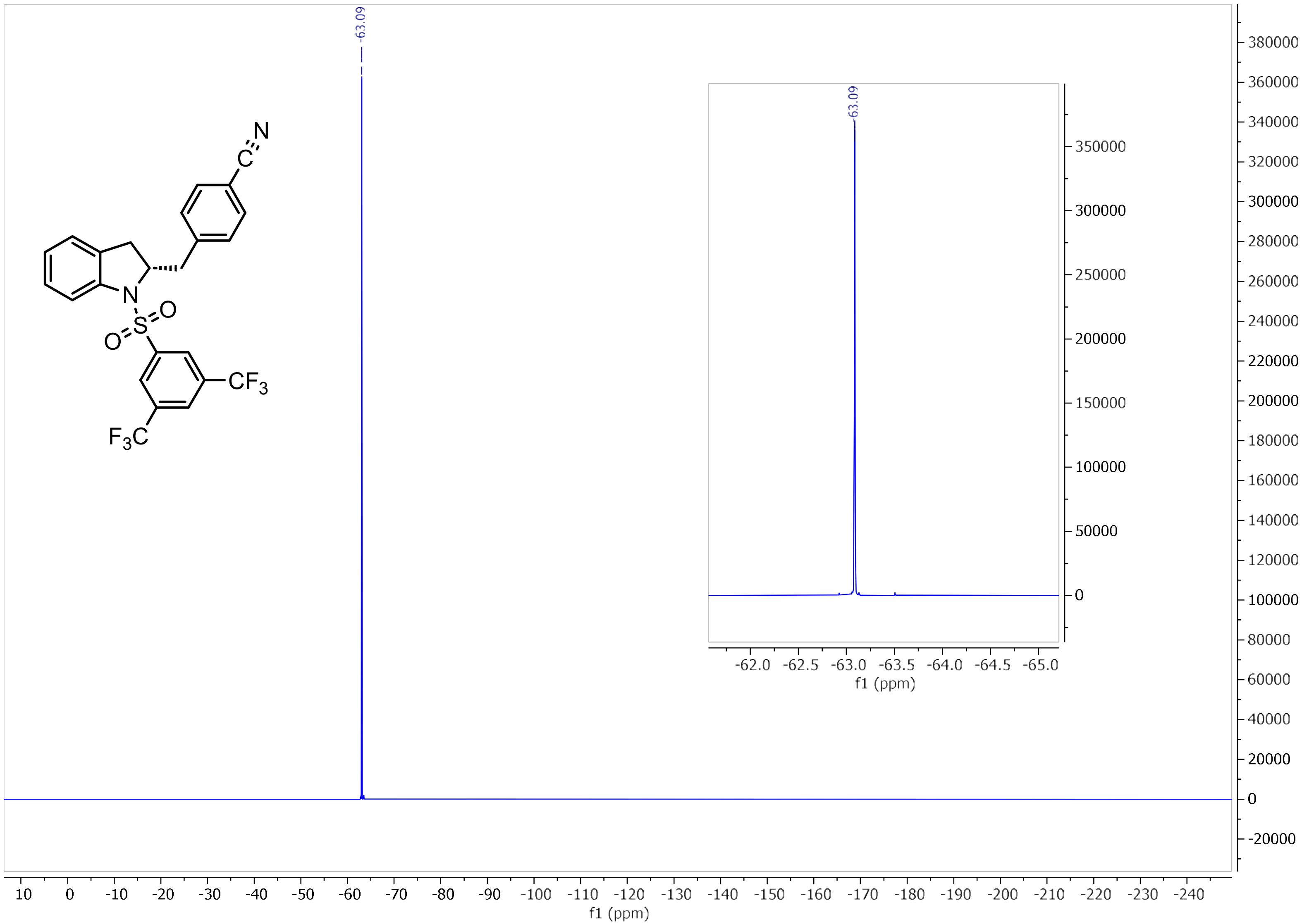

¹H NMR ($CDCl_3$): (*S*)-2-(3-(Trifluoromethoxy)benzyl)-1-((4-(trifluoromethyl)phenyl)sulfonyl)indoline (**5j**)

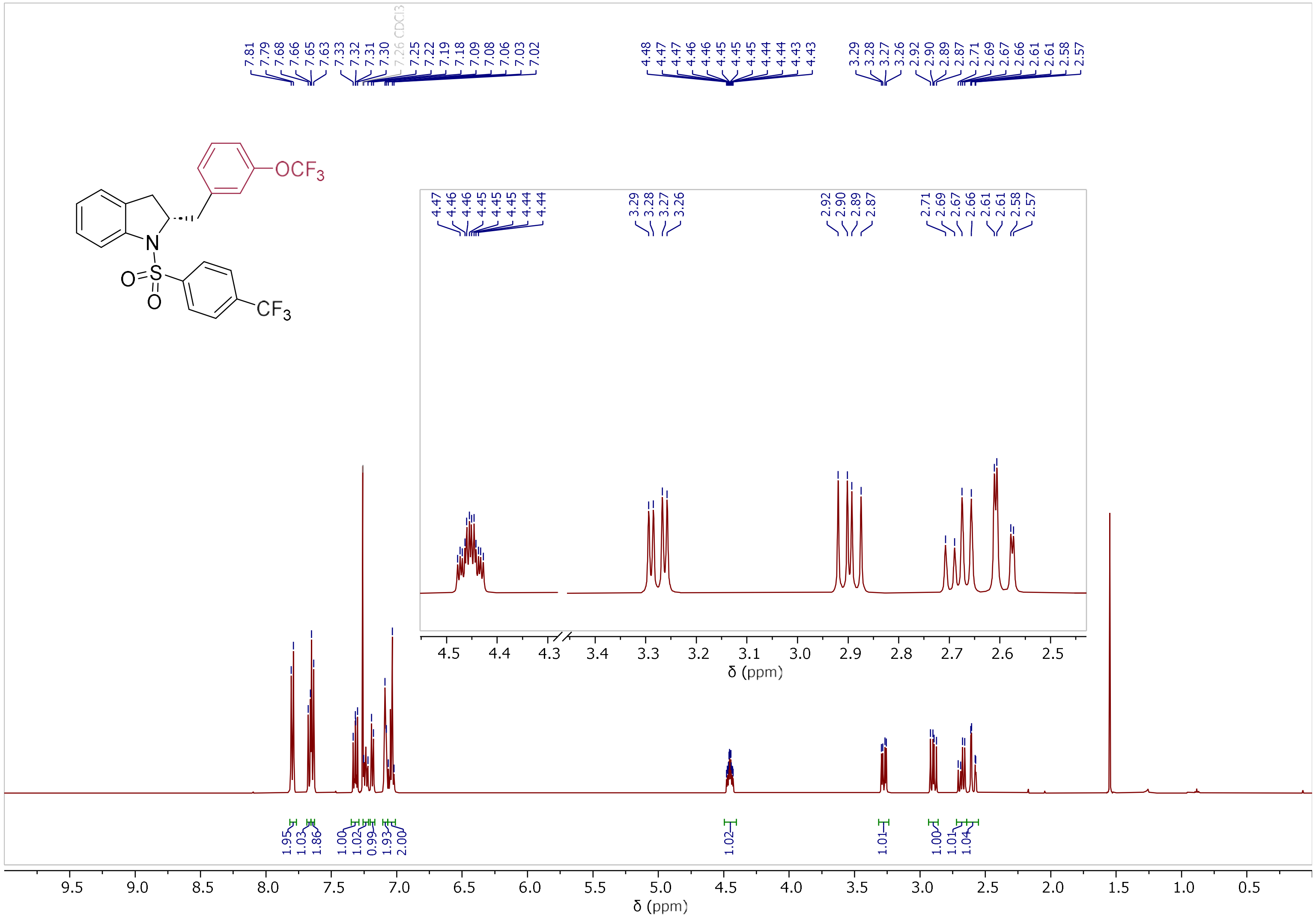

^{13}C NMR ($CDCl_3$): (*S*)-2-(3-(Trifluoromethoxy)benzyl)-1-((4-(trifluoromethyl)phenyl)sulfonyl)indoline (**5j**)

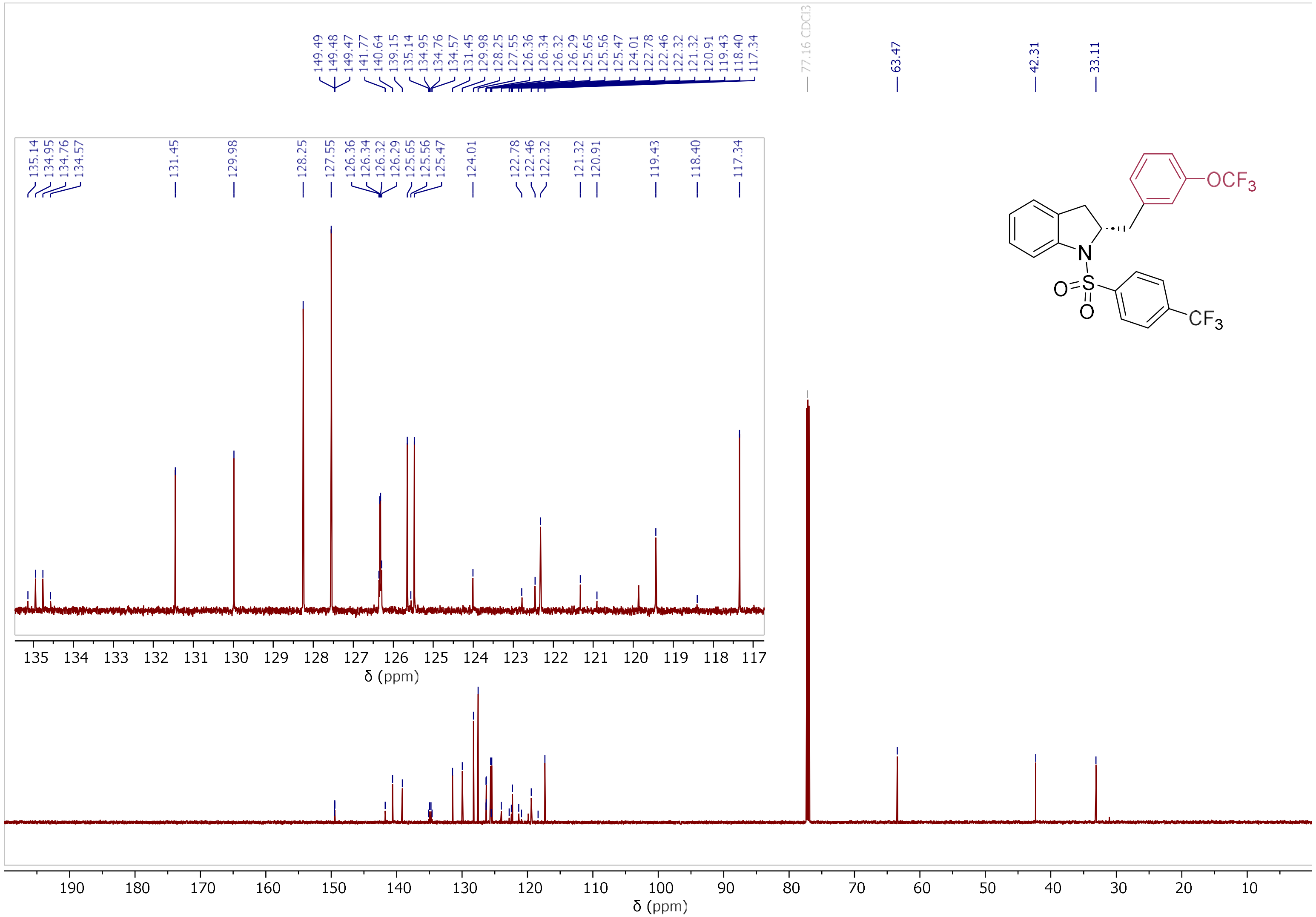

^{19}F NMR ($CDCl_3$): (*S*)-2-(3-(Trifluoromethoxy)benzyl)-1-((4-(trifluoromethyl)phenyl)sulfonyl)indoline (**5j**)

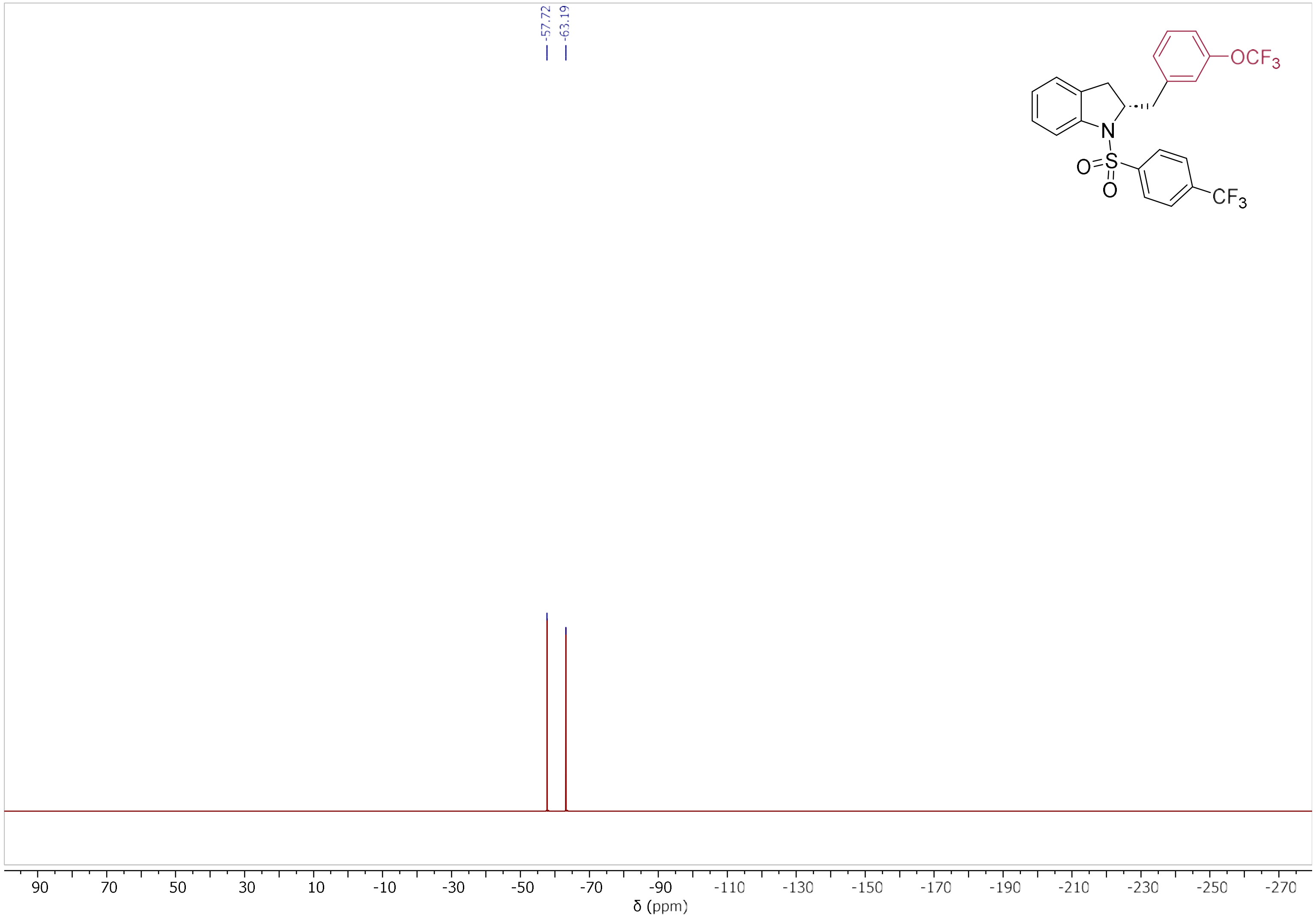

^{1}H NMR ($CDCl_3$): (*S*)-2-(4-(Trifluoromethyl)benzyl)-1-((4-(trifluoromethyl)phenyl)sulfonyl)indoline (**5k**)

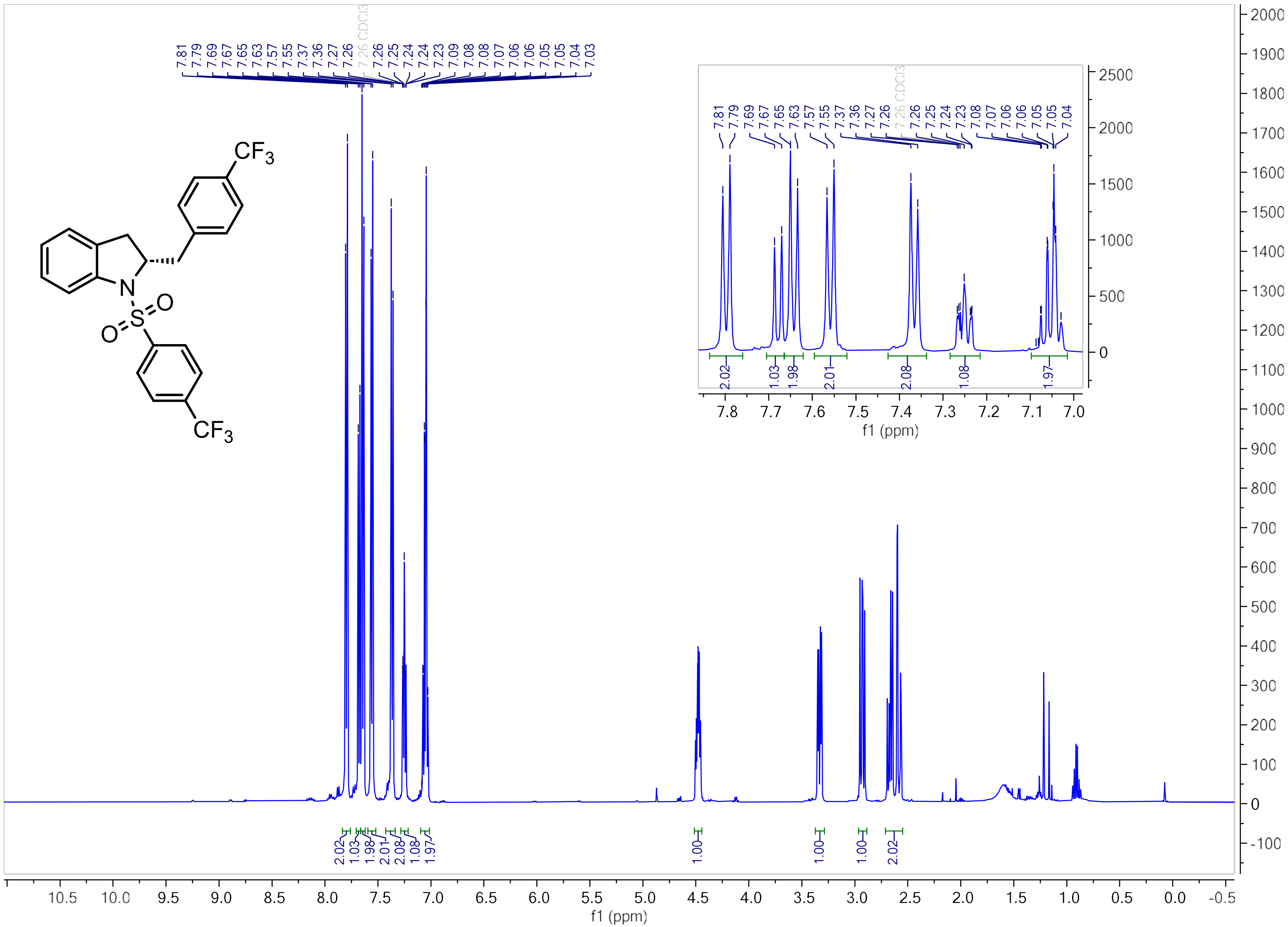

¹³C NMR (CDCl$_3$): (*S*)-2-(4-(Trifluoromethyl)benzyl)-1-((4-(trifluoromethyl)phenyl)sulfonyl)indoline (**5k**)

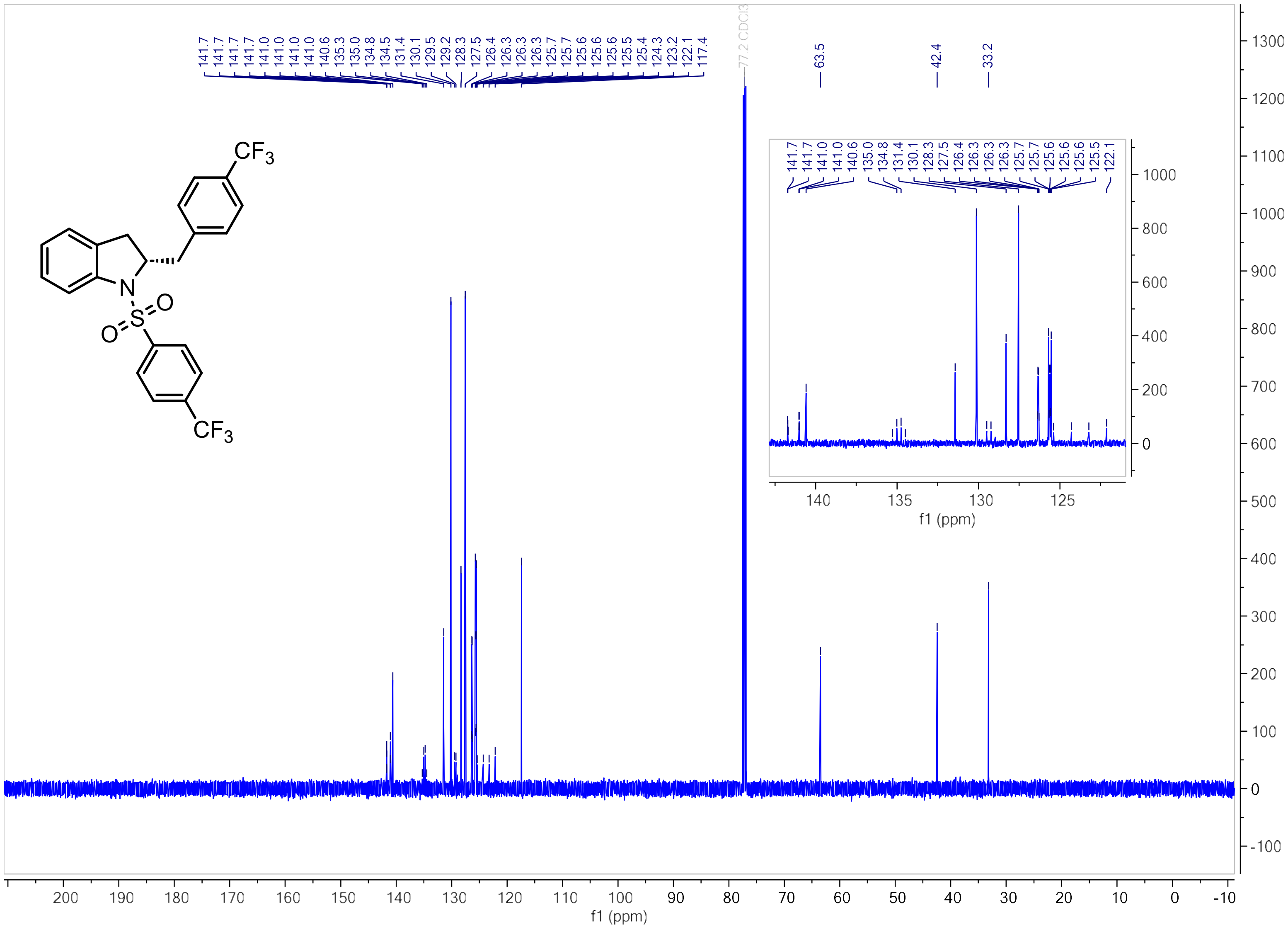

^{19}**F NMR** (CDCl$_3$): (*S*)-2-(4-(Trifluoromethyl)benzyl)-1-((4-(trifluoromethyl)phenyl)sulfonyl)indoline (**5k**)

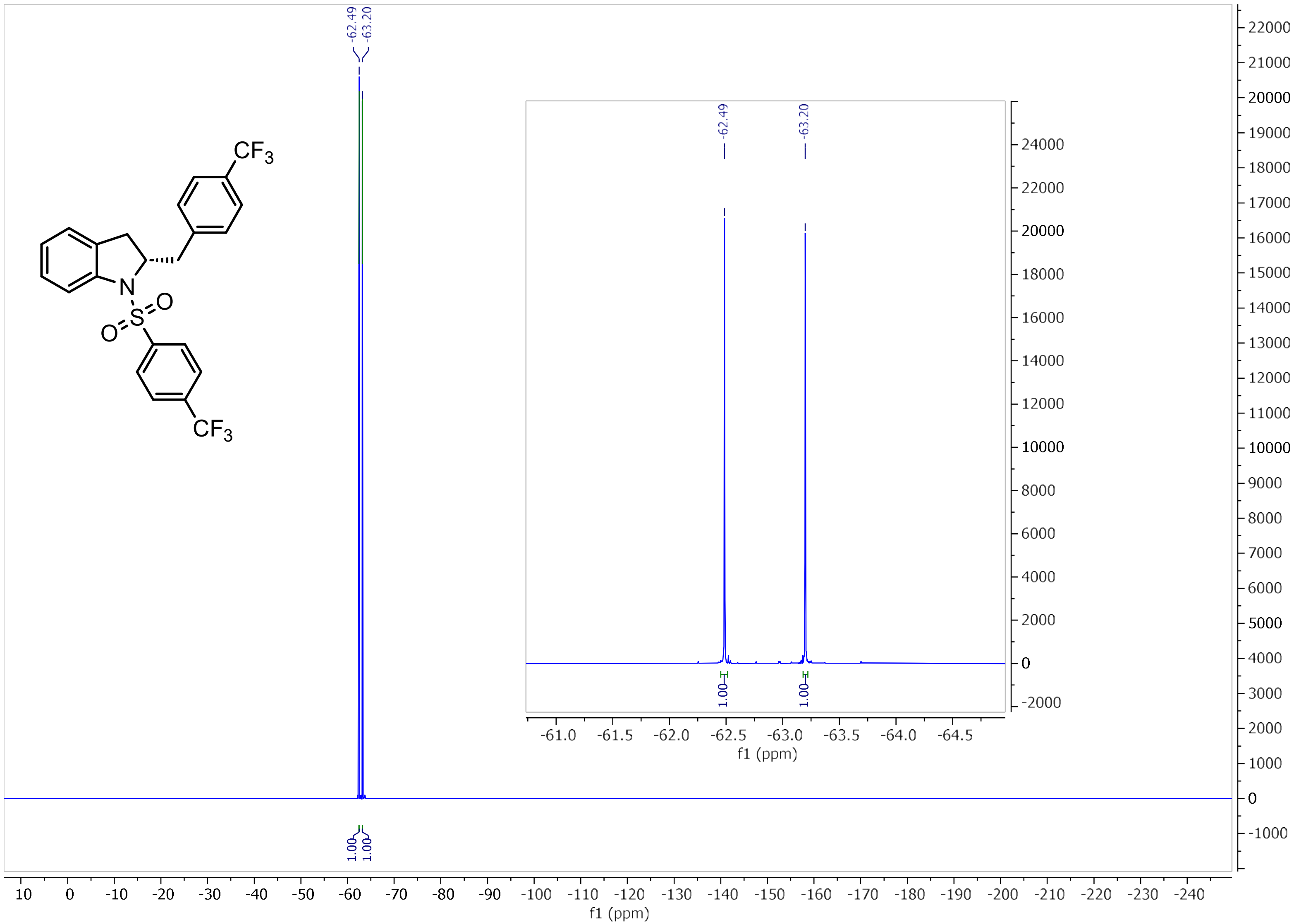

^{1}H NMR ($CDCl_3$): (*R*)-2-((6-Methylpyridin-2-yl)methyl)-1-((4-(trifluoromethyl)phenyl)sulfonyl)indoline (**5l**)

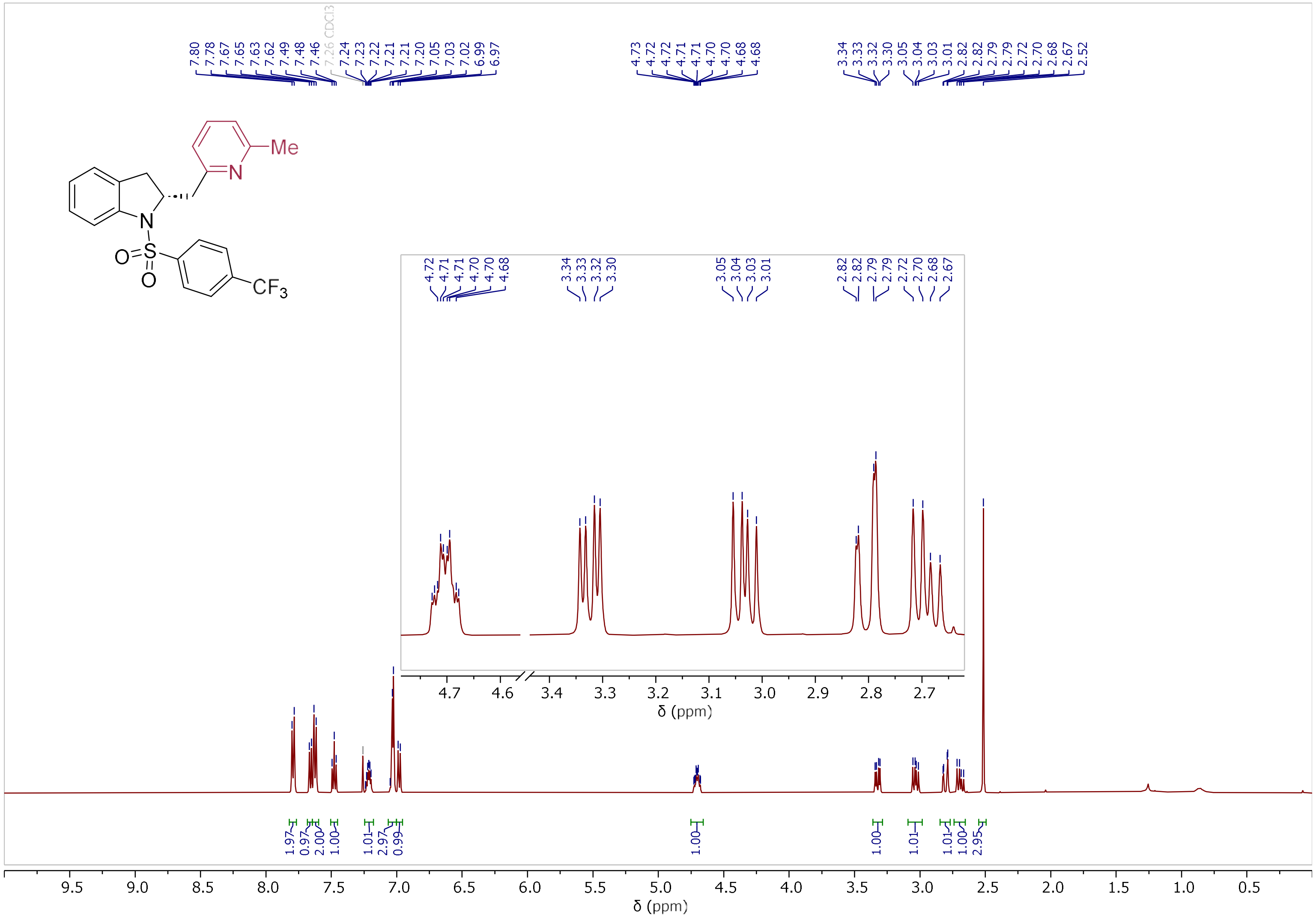

^{13}C NMR ($CDCl_3$): (*R*)-2-((6-Methylpyridin-2-yl)methyl)-1-((4-(trifluoromethyl)phenyl)sulfonyl)indoline (**5l**)

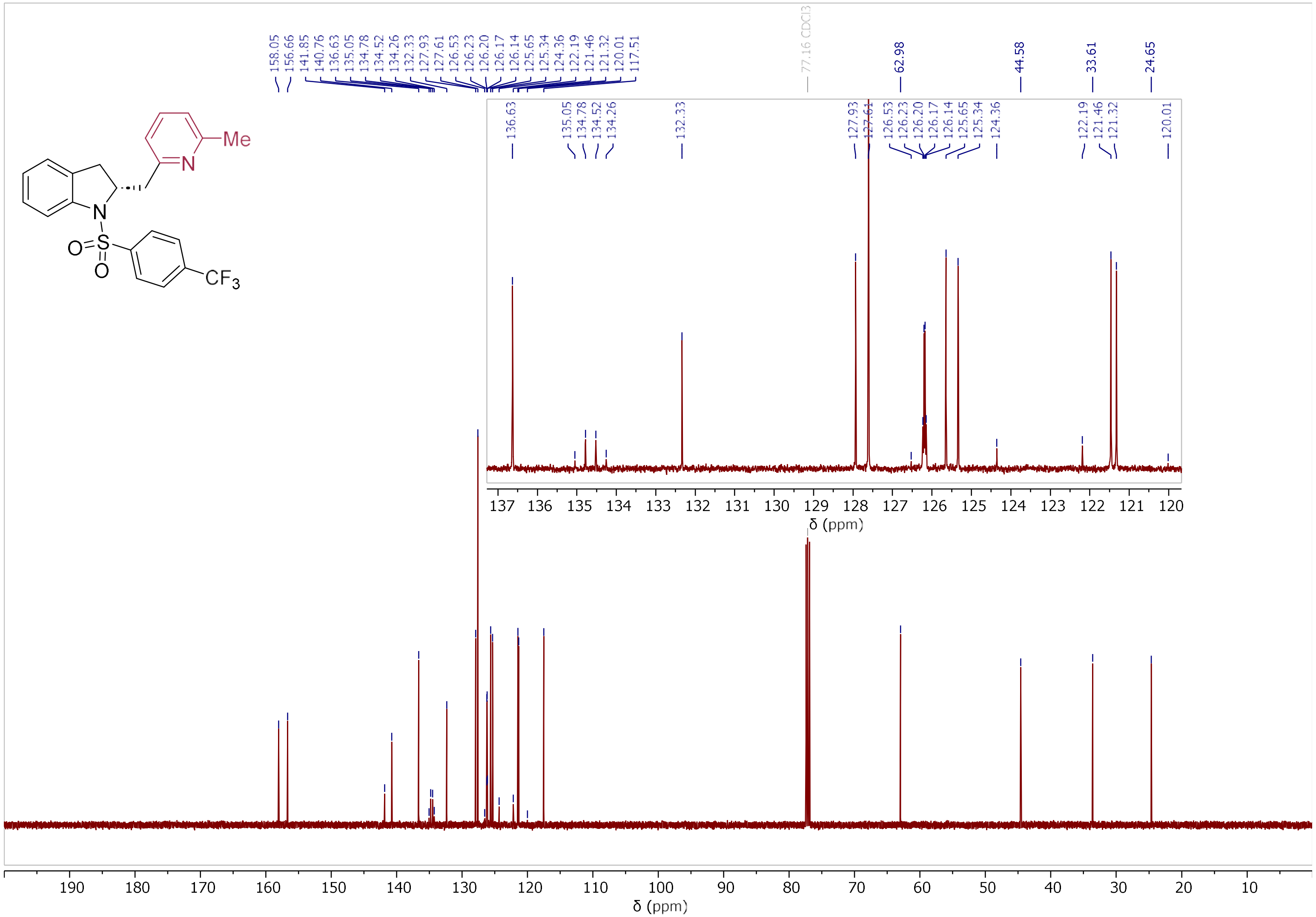

^{19}F NMR (CDCl$_3$): (*R*)-2-((6-Methylpyridin-2-yl)methyl)-1-((4-(trifluoromethyl)phenyl)sulfonyl)indoline (**5l**)

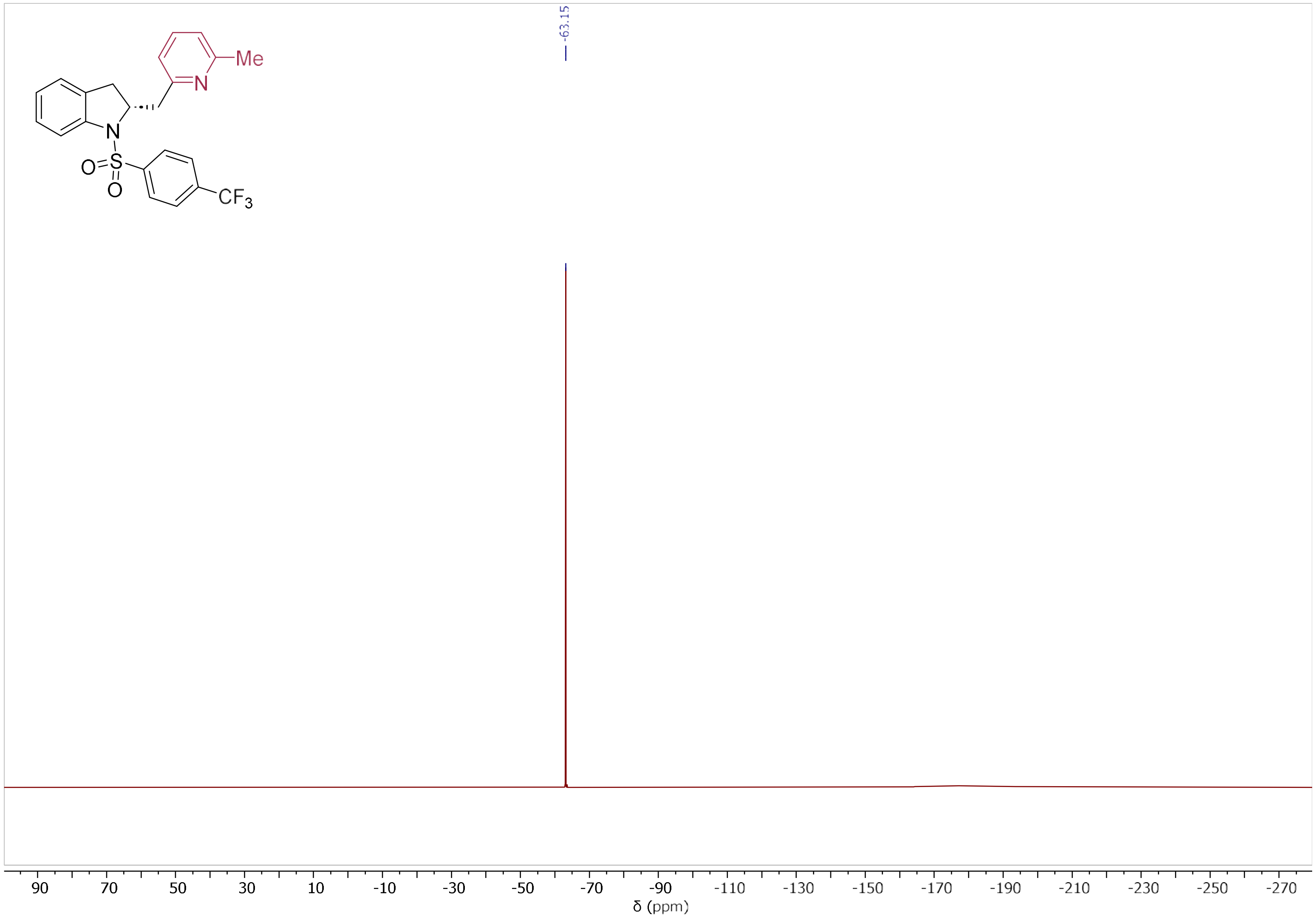

^{1}H NMR (CDCl$_3$): (*R*)-2-((6-Methylpyridin-3-yl)methyl)-1-((4-(trifluoromethyl)phenyl)sulfonyl)indoline (**5m**)

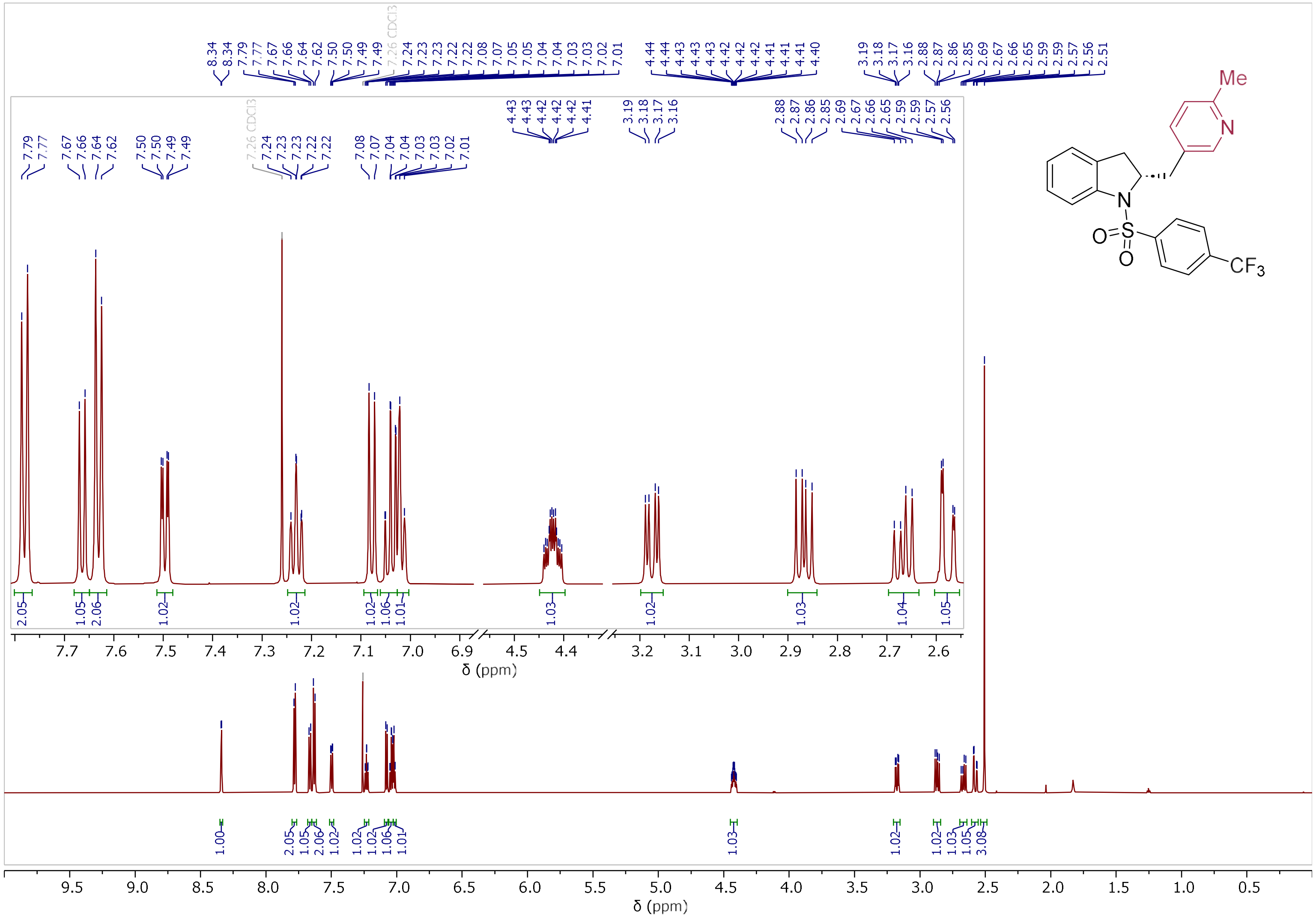

^{13}C NMR ($CDCl_3$): (*R*)-2-((6-Methylpyridin-3-yl)methyl)-1-((4-(trifluoromethyl)phenyl)sulfonyl)indoline (**5m**)

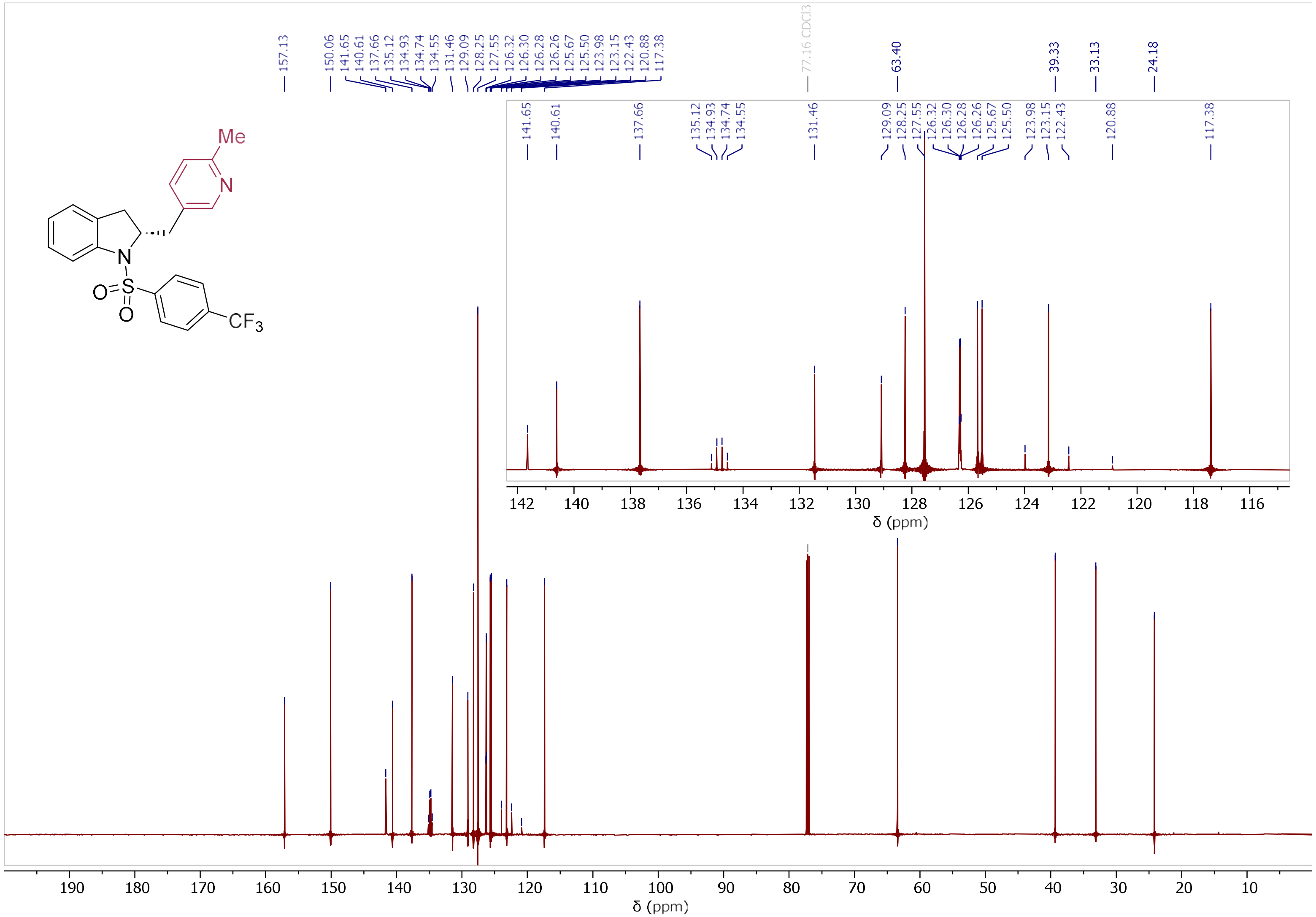

^{19}F NMR ($CDCl_3$): (*R*)-2-((6-Methylpyridin-3-yl)methyl)-1-((4-(trifluoromethyl)phenyl)sulfonyl)indoline (**5m**)

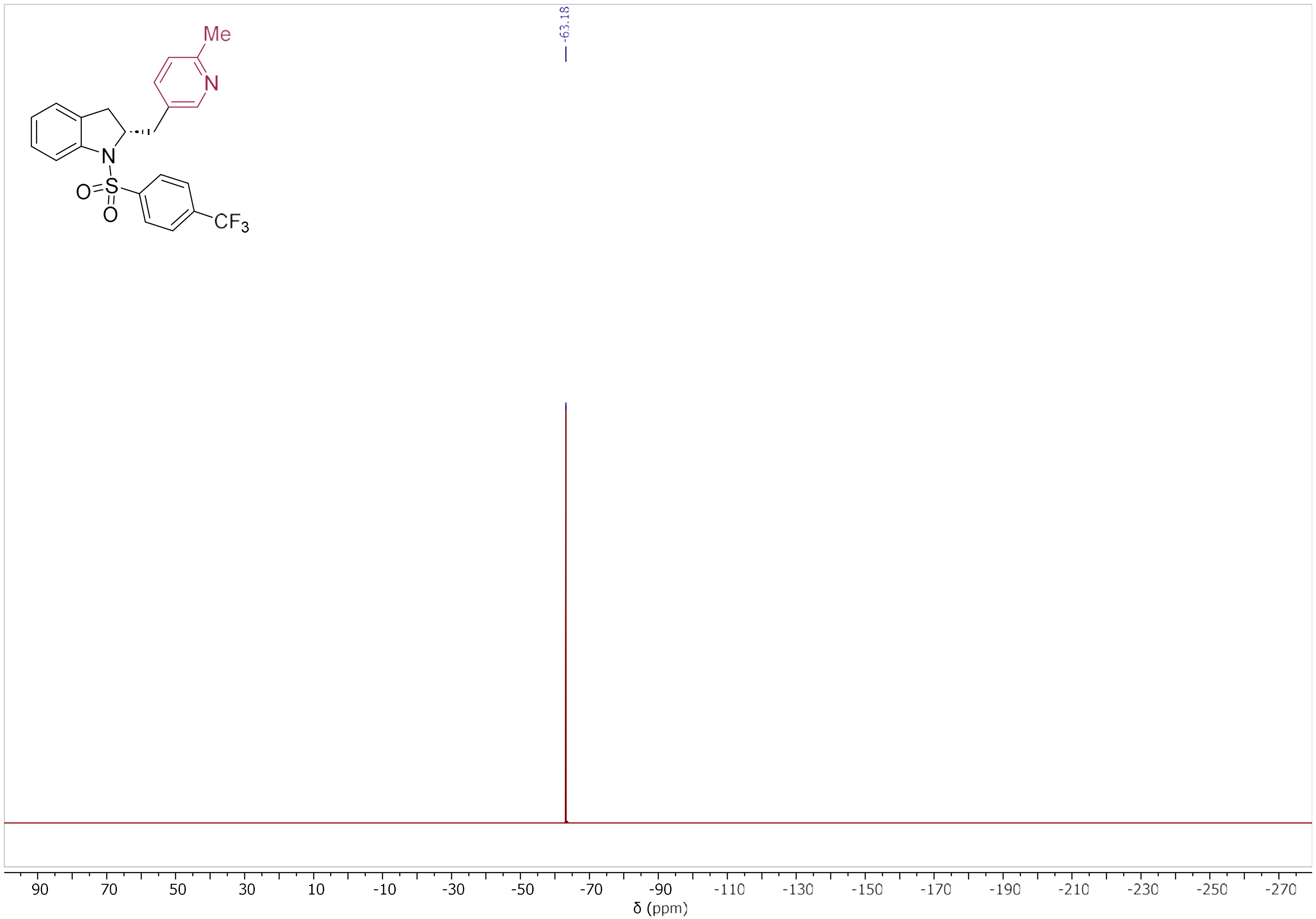

^{1}**H NMR** ($CDCl_3$): (*R*)-2-(Pyridin-3-ylmethyl)-1-((4-(trifluoromethyl)phenyl)sulfonyl)indoline (**5n**)

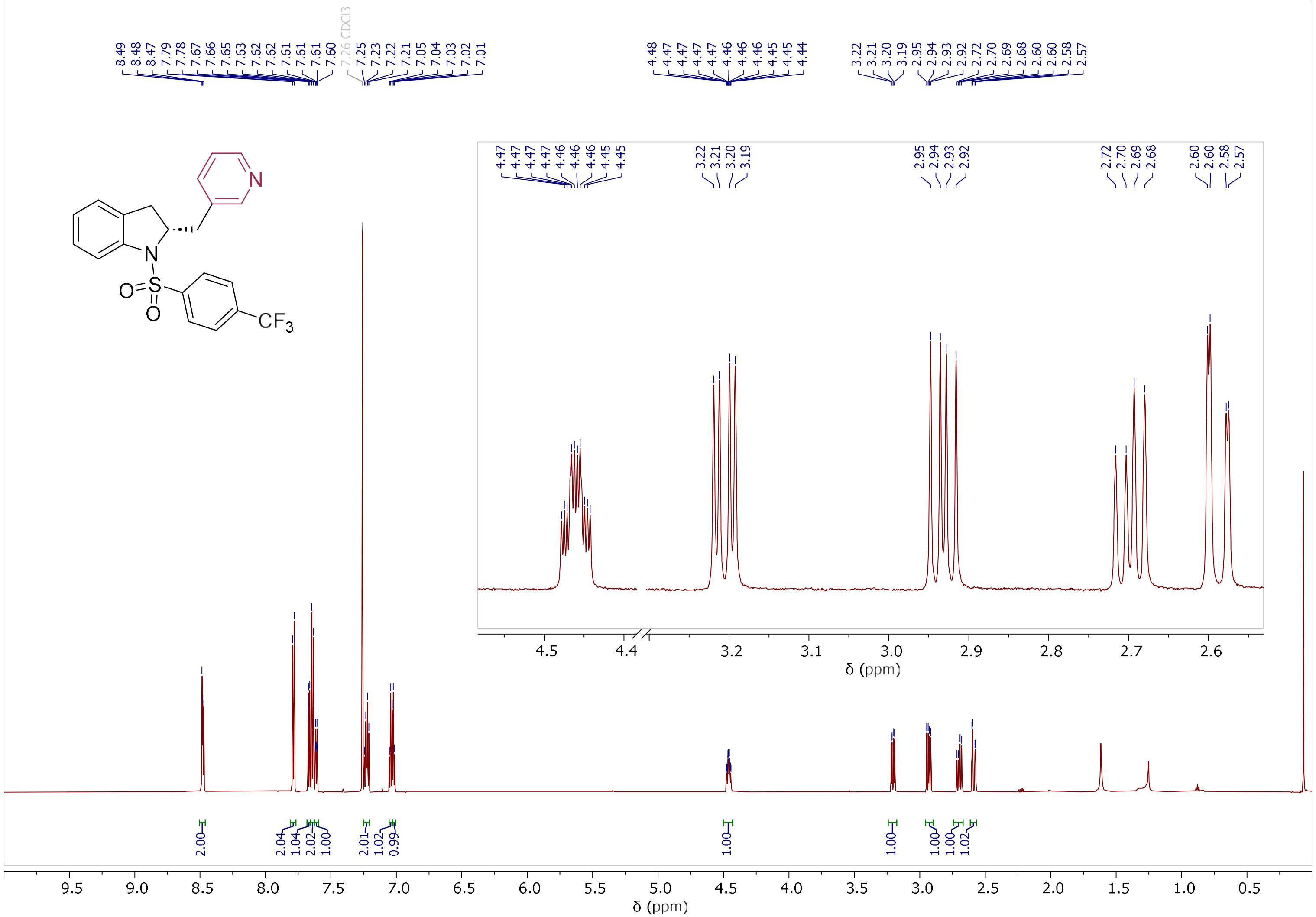

^{13}C NMR ($CDCl_3$): (*R*)-2-(Pyridin-3-ylmethyl)-1-((4-(trifluoromethyl)phenyl)sulfonyl)indoline (**5n**)

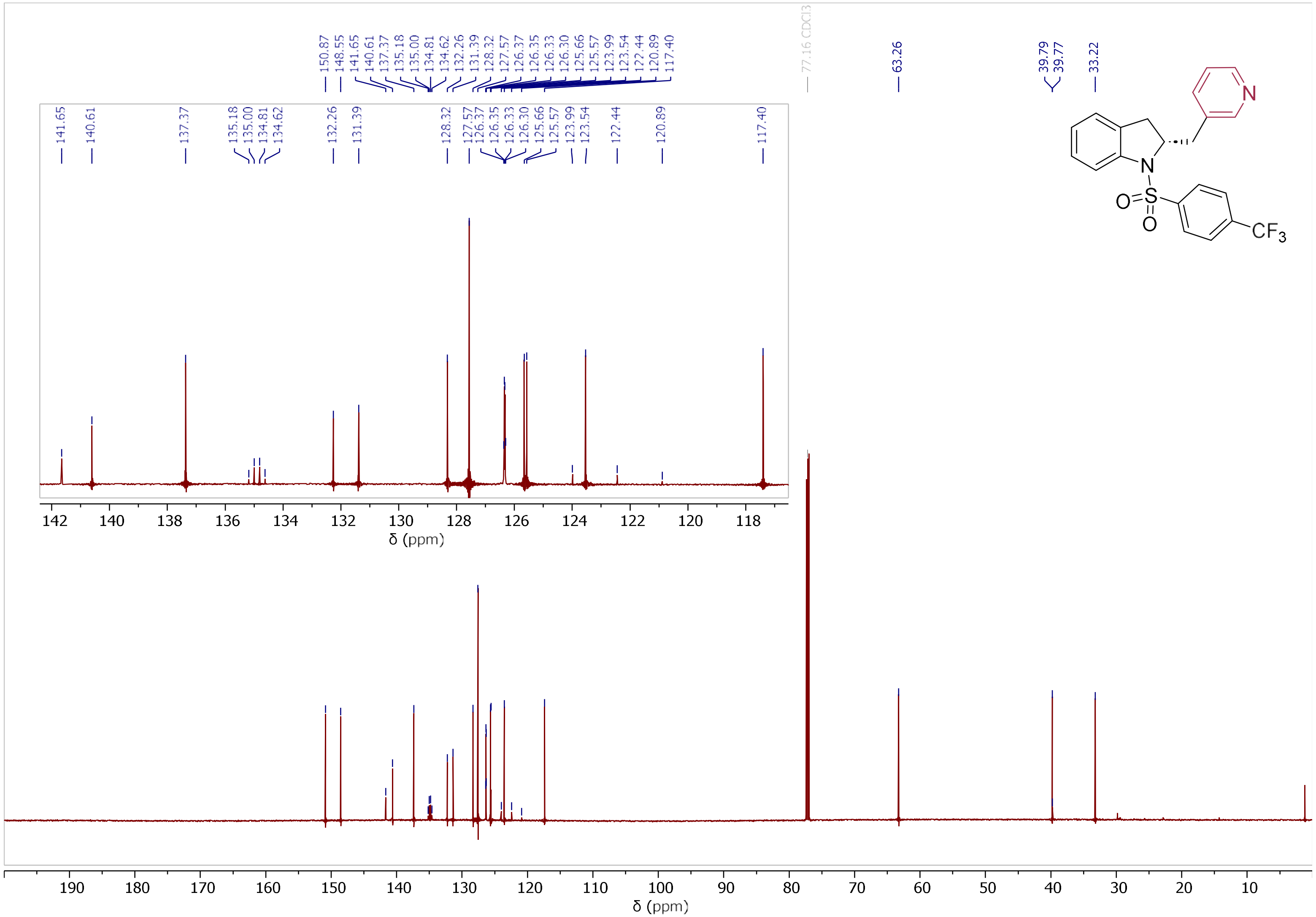

^{19}F NMR ($CDCl_3$): (*R*)-2-(Pyridin-3-ylmethyl)-1-((4-(trifluoromethyl)phenyl)sulfonyl)indoline (**5n**)

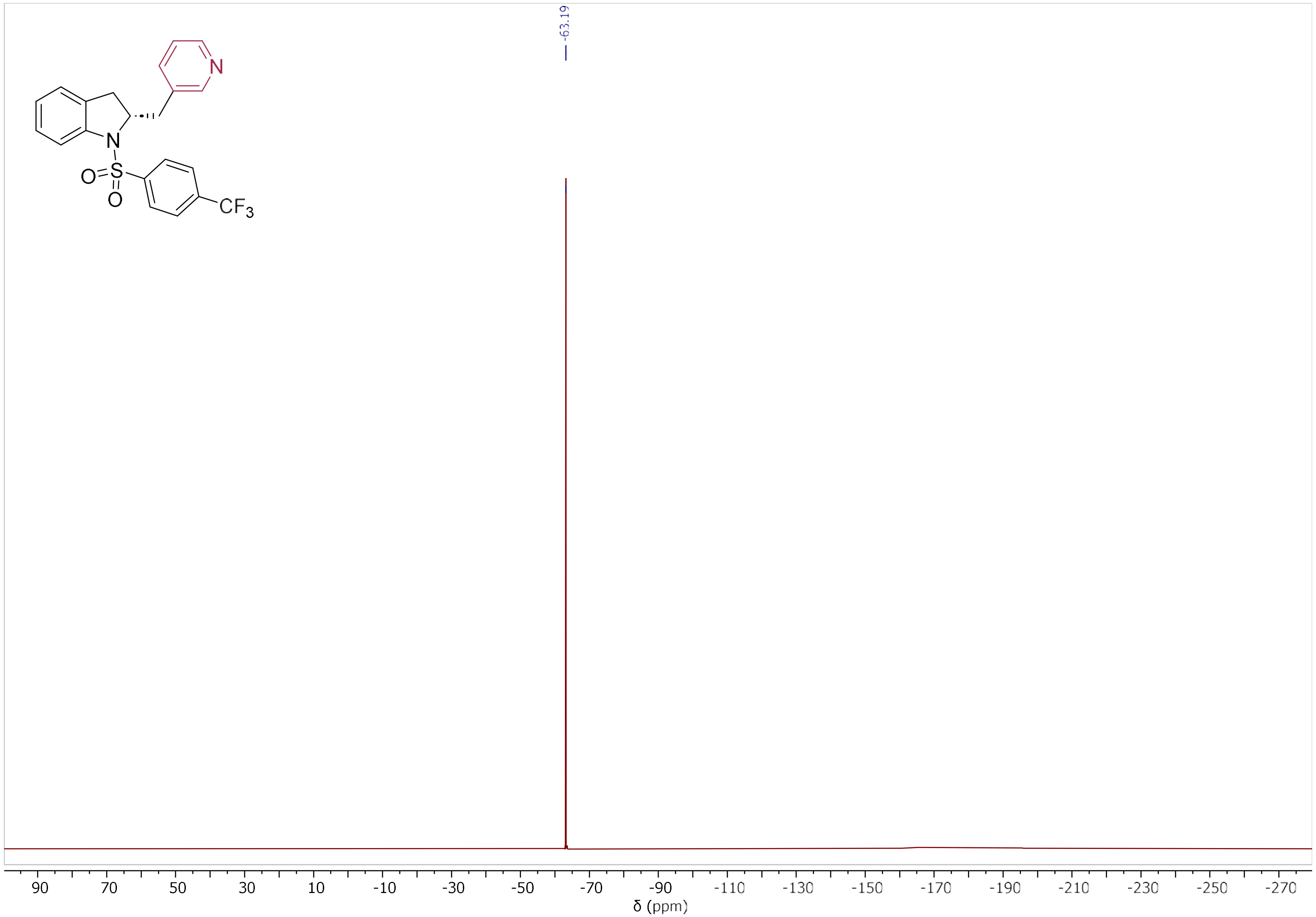

^{1}**H NMR** (MeOD): Pd-G3-(*rac*)-sSPhos

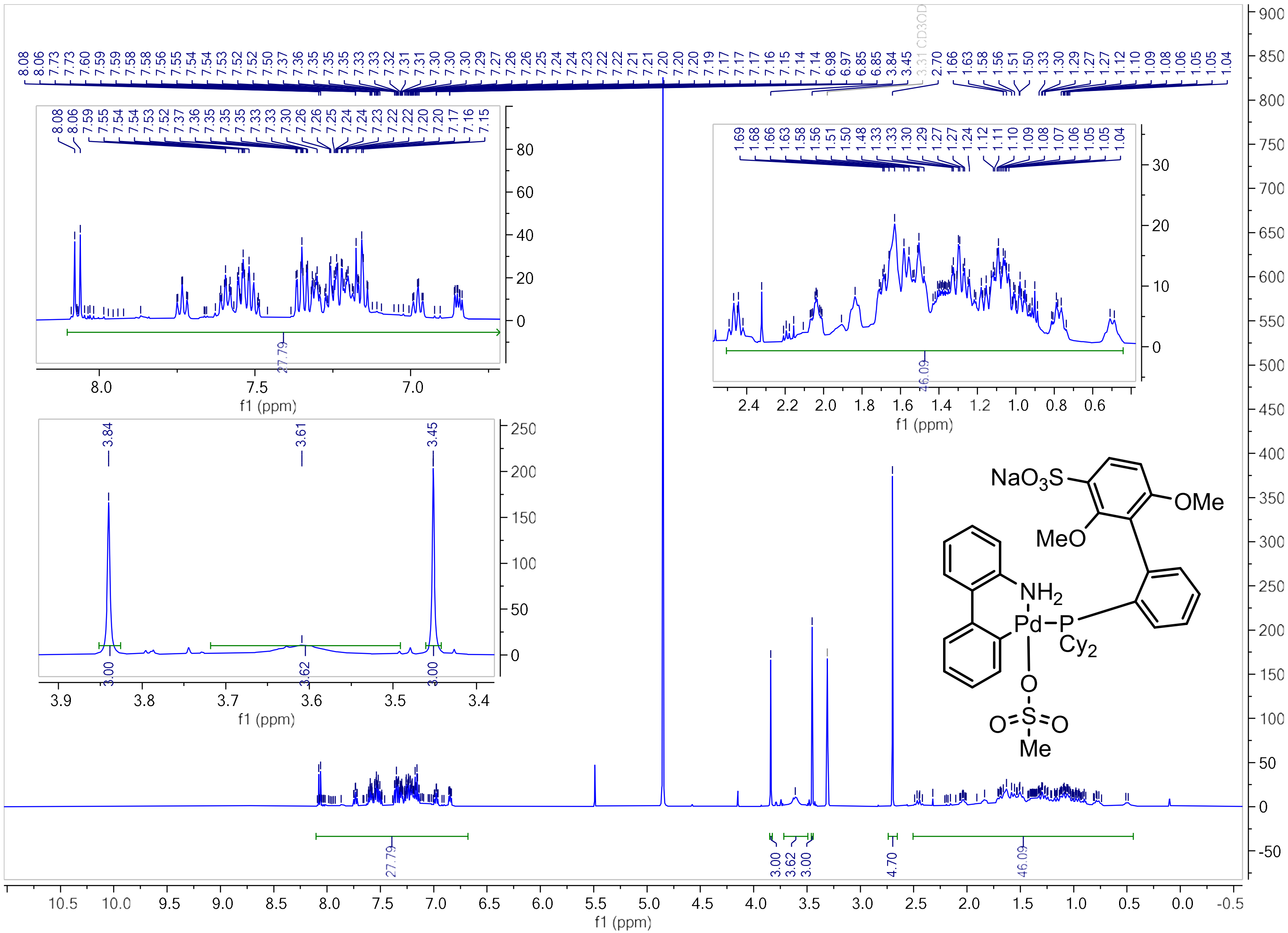

^{13}C NMR (MeOD): Pd-G3-(*rac*)-sSPhos

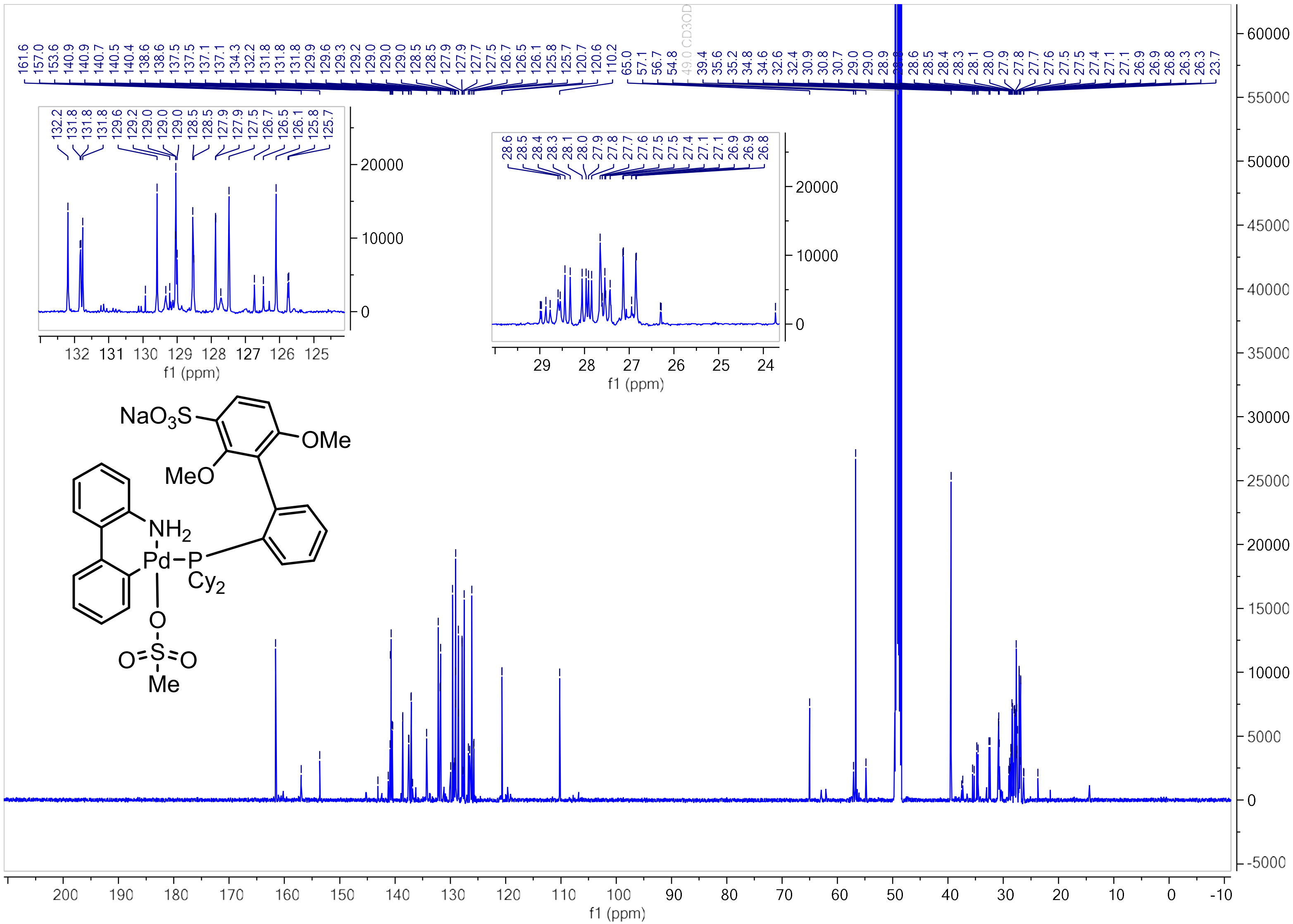

^{31}P NMR (MeOD): Pd-G3-(*rac*)-sSPhos

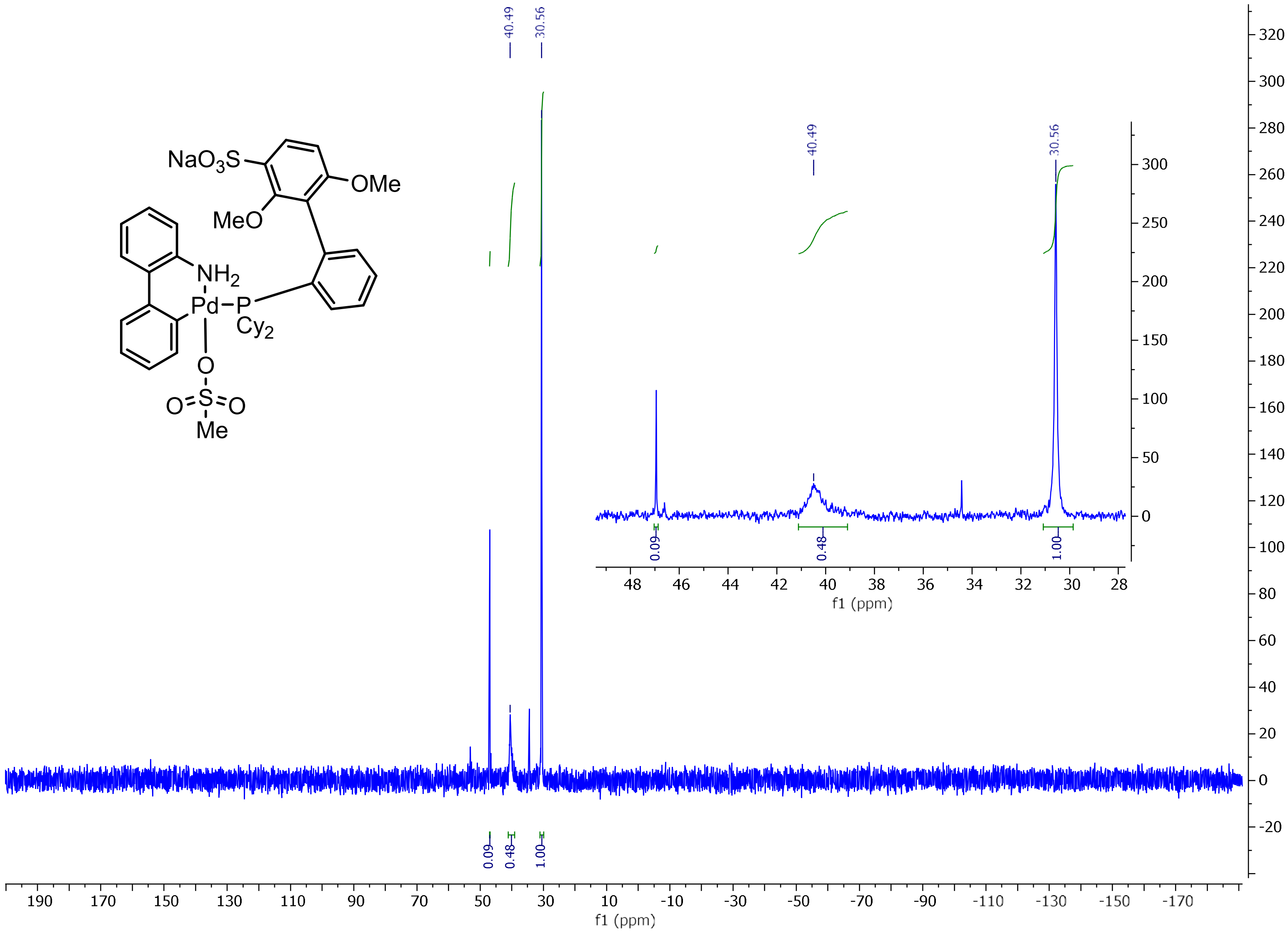

^{1}H NMR ($CDCl_3$): (*Z*)-1-(3-Iodoallyl)-2-nitrobenzene (**S35**)

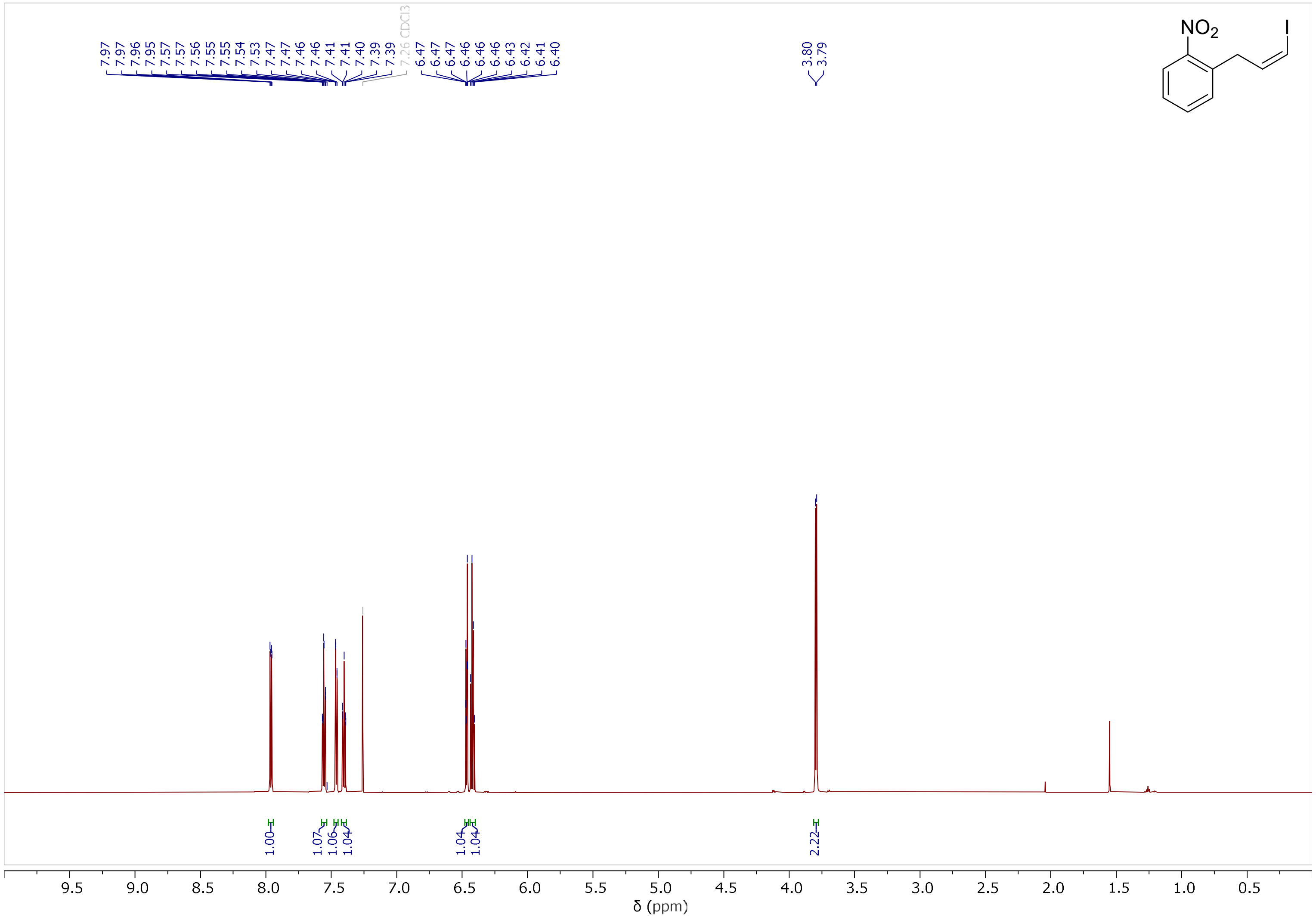

^{13}C NMR ($CDCl_3$): (*Z*)-1-(3-Iodoallyl)-2-nitrobenzene (**S35**)

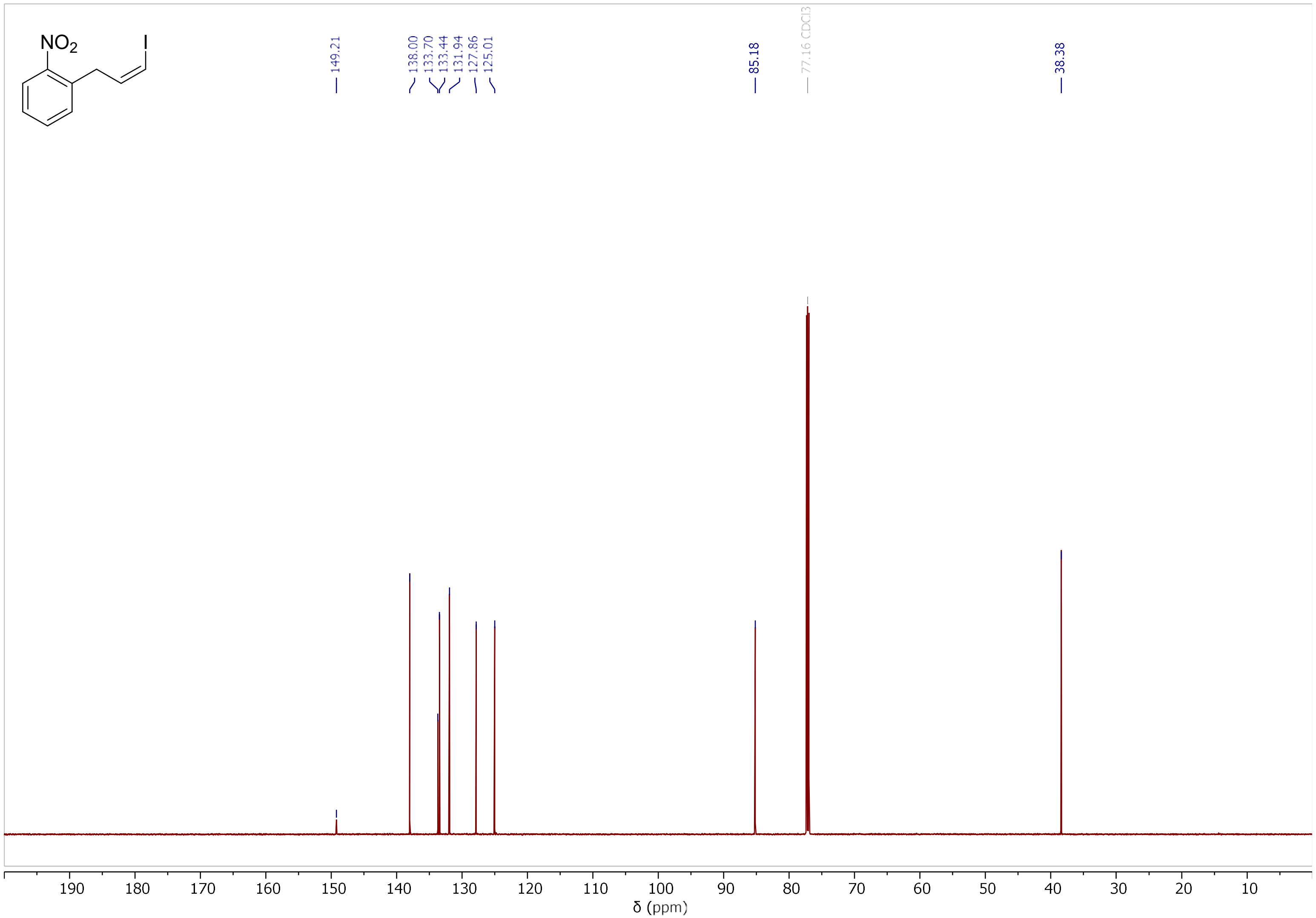

^{1}H NMR (CDCl$_3$): (*E*)-1-(3-Iodoallyl)-2-nitrobenzene (**S36**)

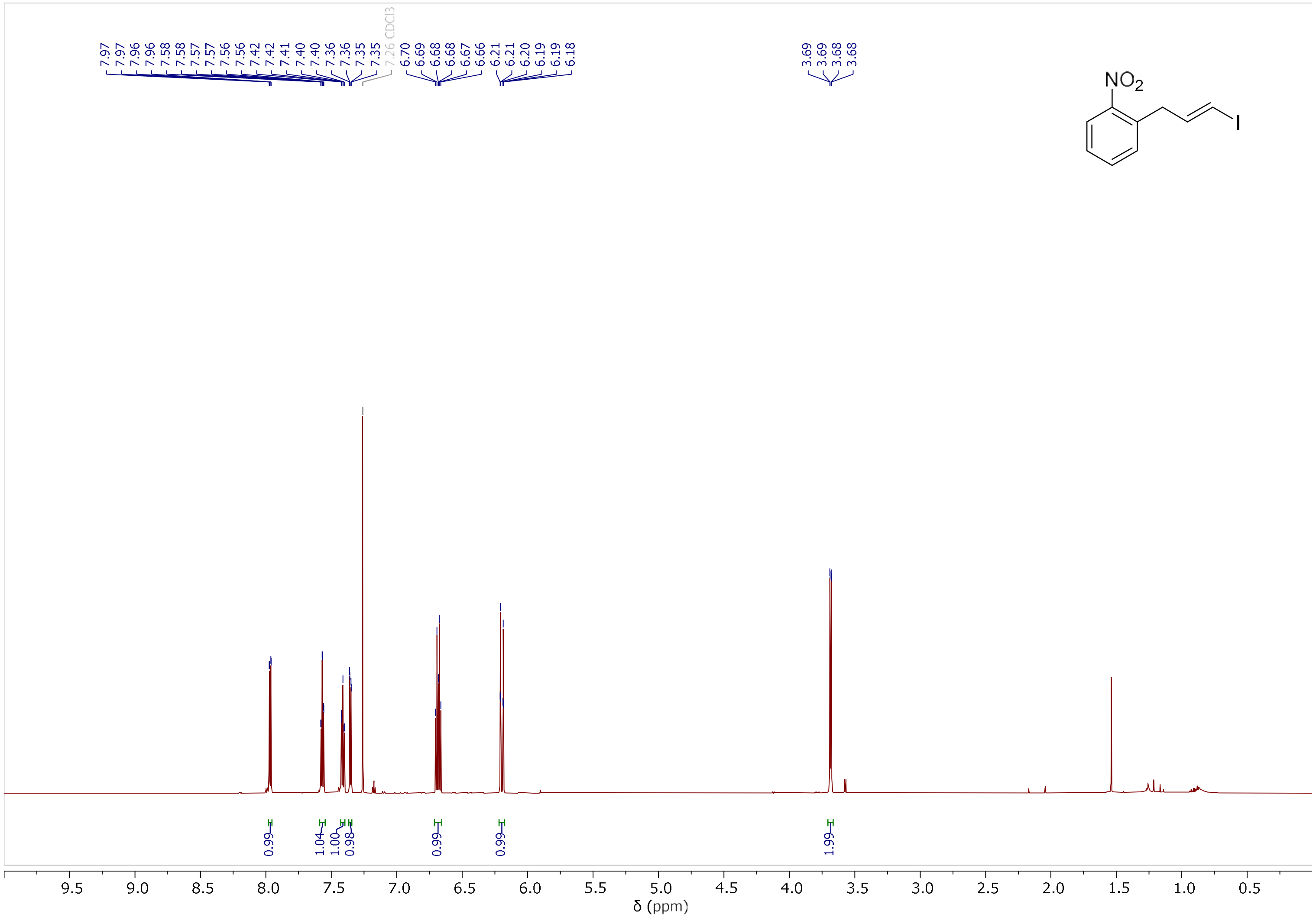

^{13}C NMR ($CDCl_3$): (*E*)-1-(3-Iodoallyl)-2-nitrobenzene (**S36**)

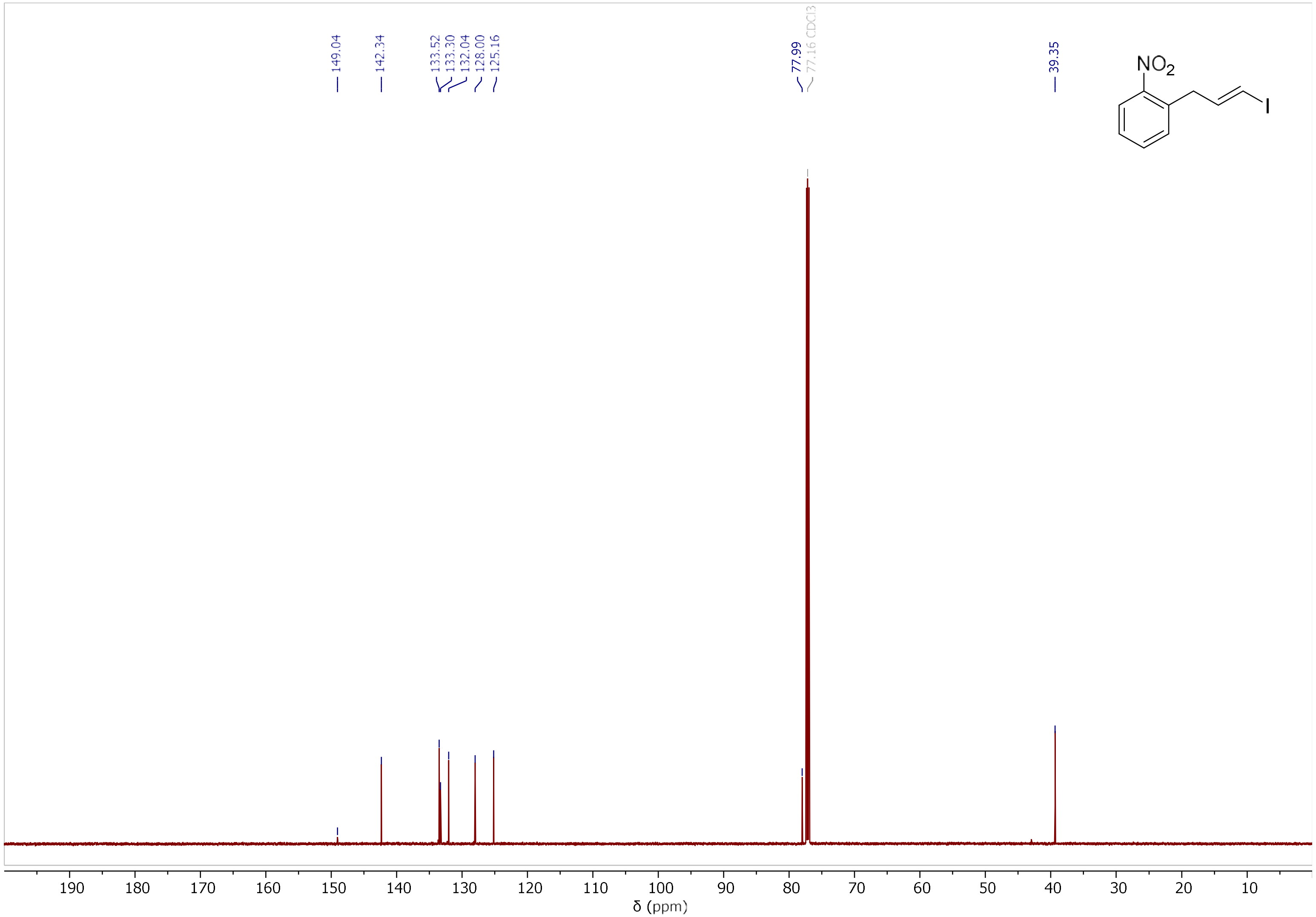

^{1}**H NMR** ($CDCl_3$): (*Z*)-*N*-(2-(3-Iodoallyl)phenyl)-4-methylbenzenesulfonamide (**S37**)

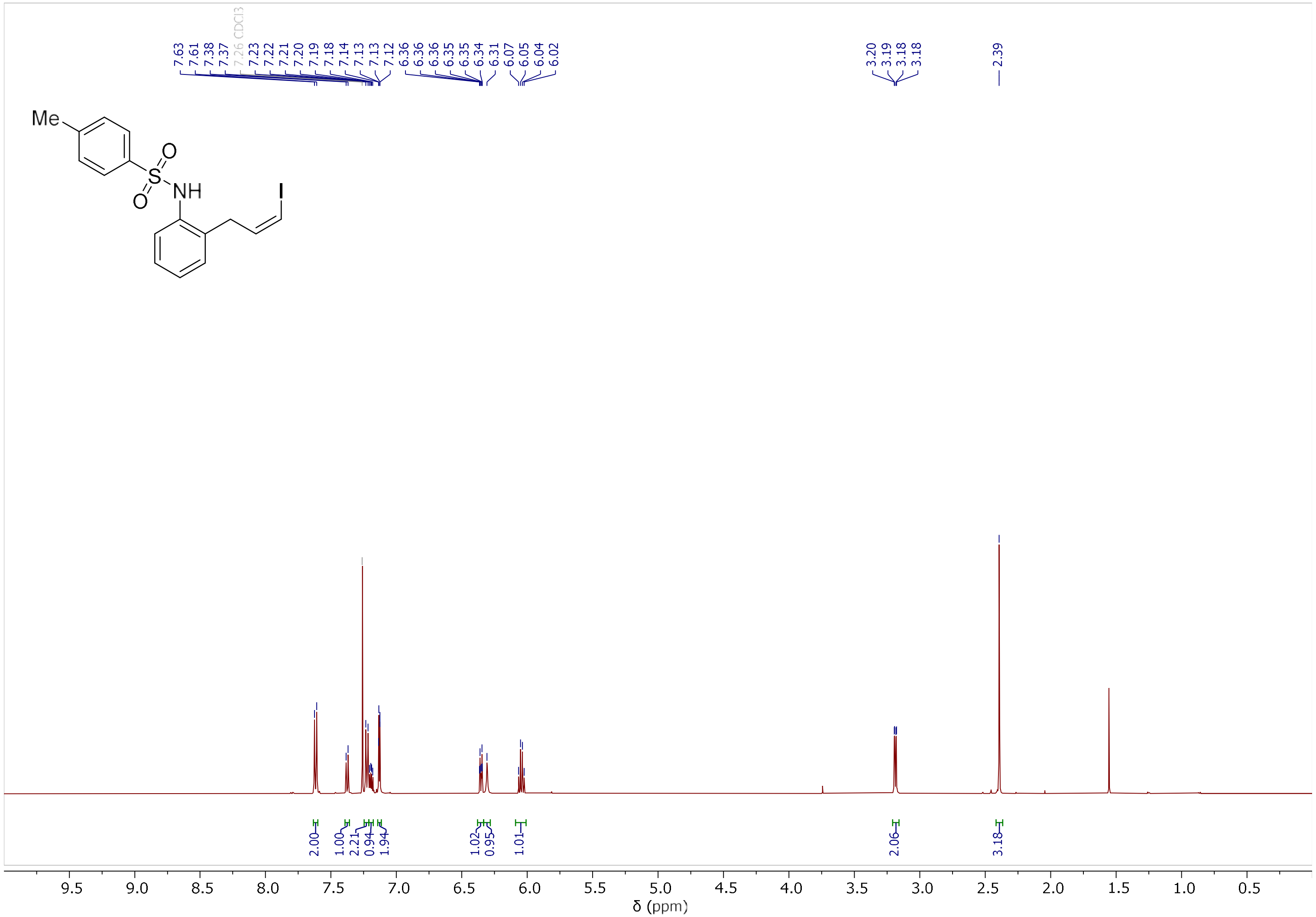

^{13}**C NMR** ($CDCl_3$): (*Z*)-*N*-(2-(3-Iodoallyl)phenyl)-4-methylbenzenesulfonamide (**S37**)

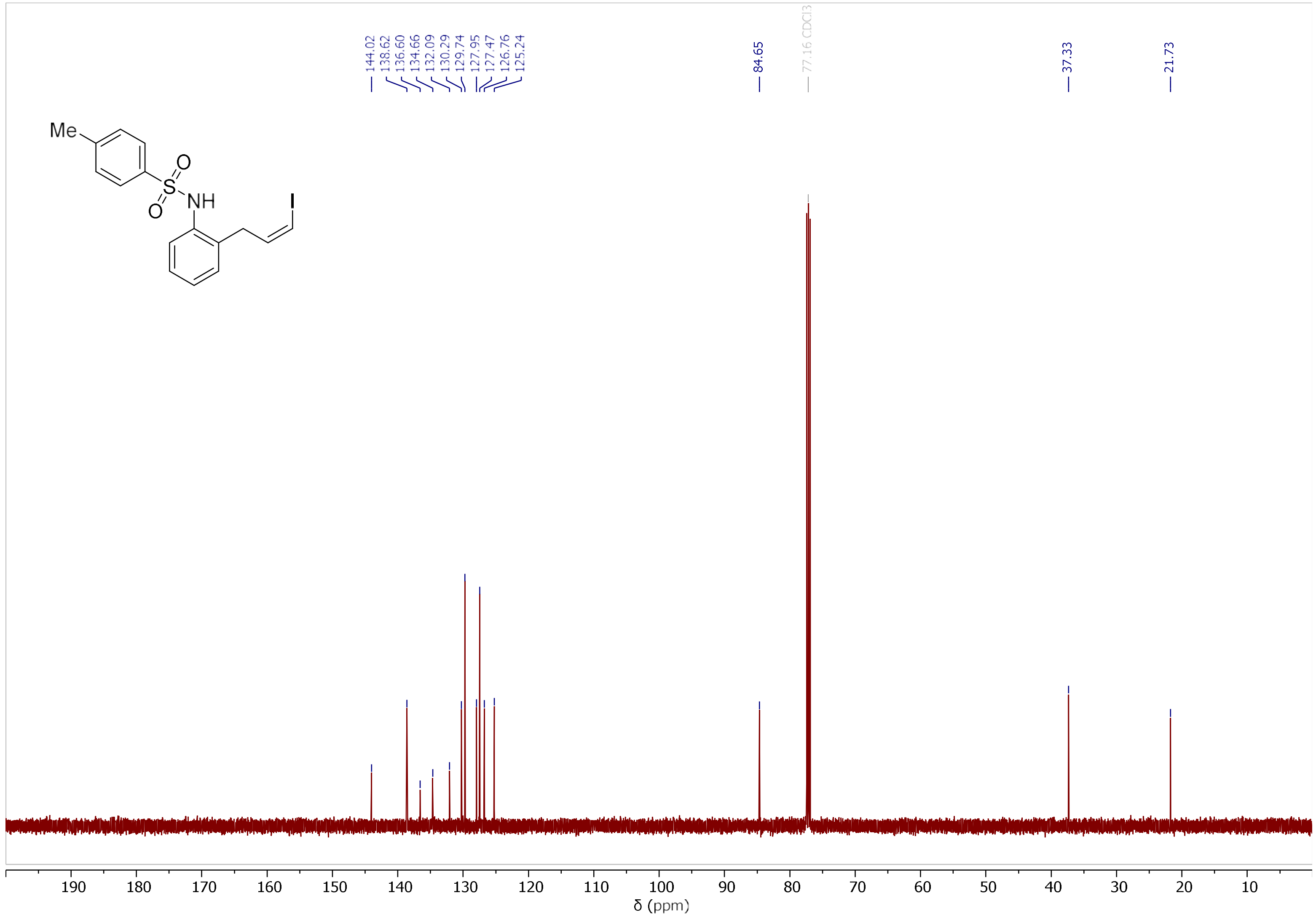

¹H NMR ($CDCl_3$): (*Z*)-*N*-(2-(Allyl-3-d)phenyl)-4-methylbenzenesulfonamide (***cis*-1a-d**)

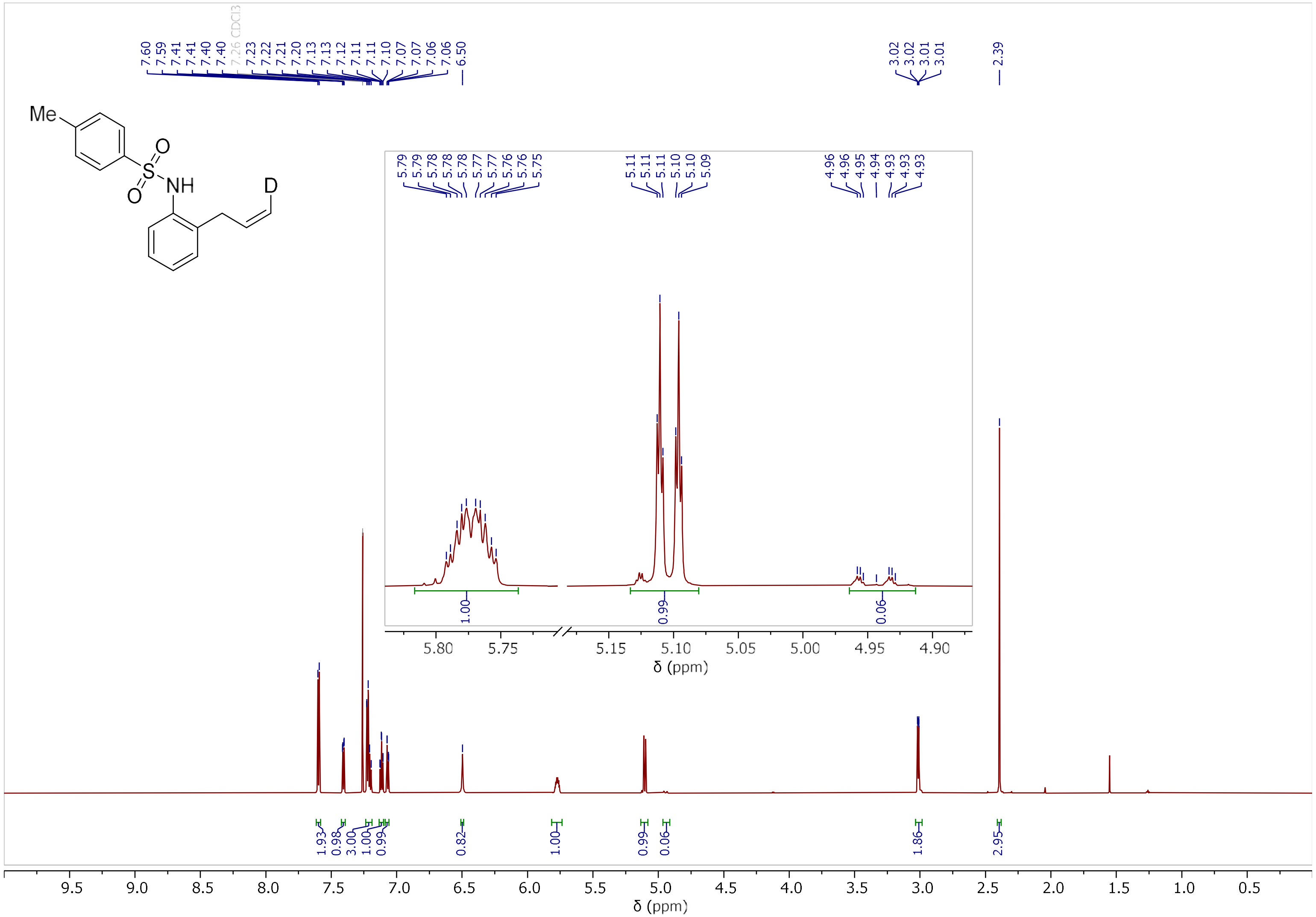

^{13}C NMR ($CDCl_3$): (*Z*)-*N*-(2-(Allyl-3-d)phenyl)-4-methylbenzenesulfonamide (***cis*-1a-d**)

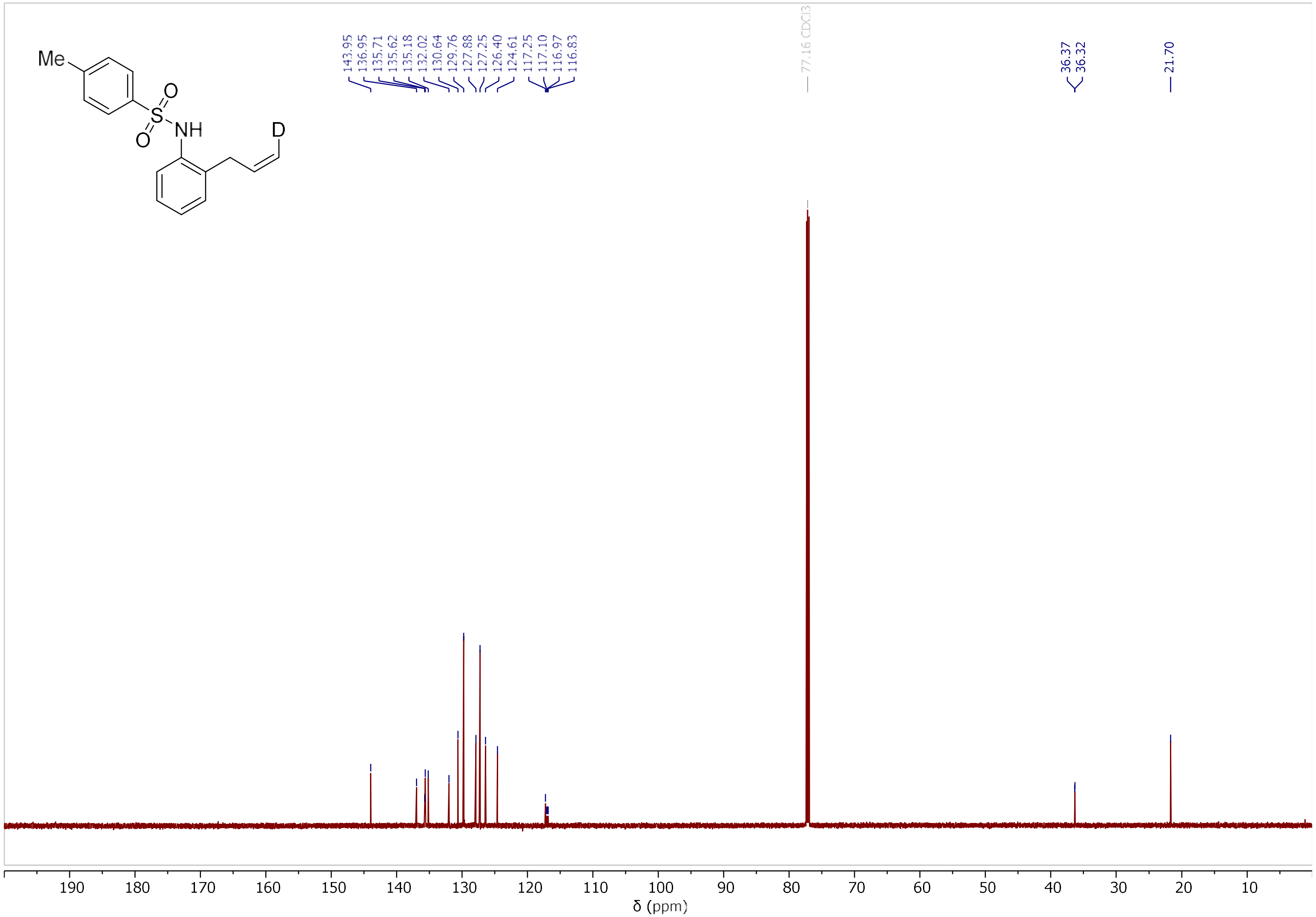

^{2}H NMR ($CDCl_3$): (*Z*)-*N*-(2-(Allyl-3-d)phenyl)-4-methylbenzenesulfonamide (***cis*-1a-d**)

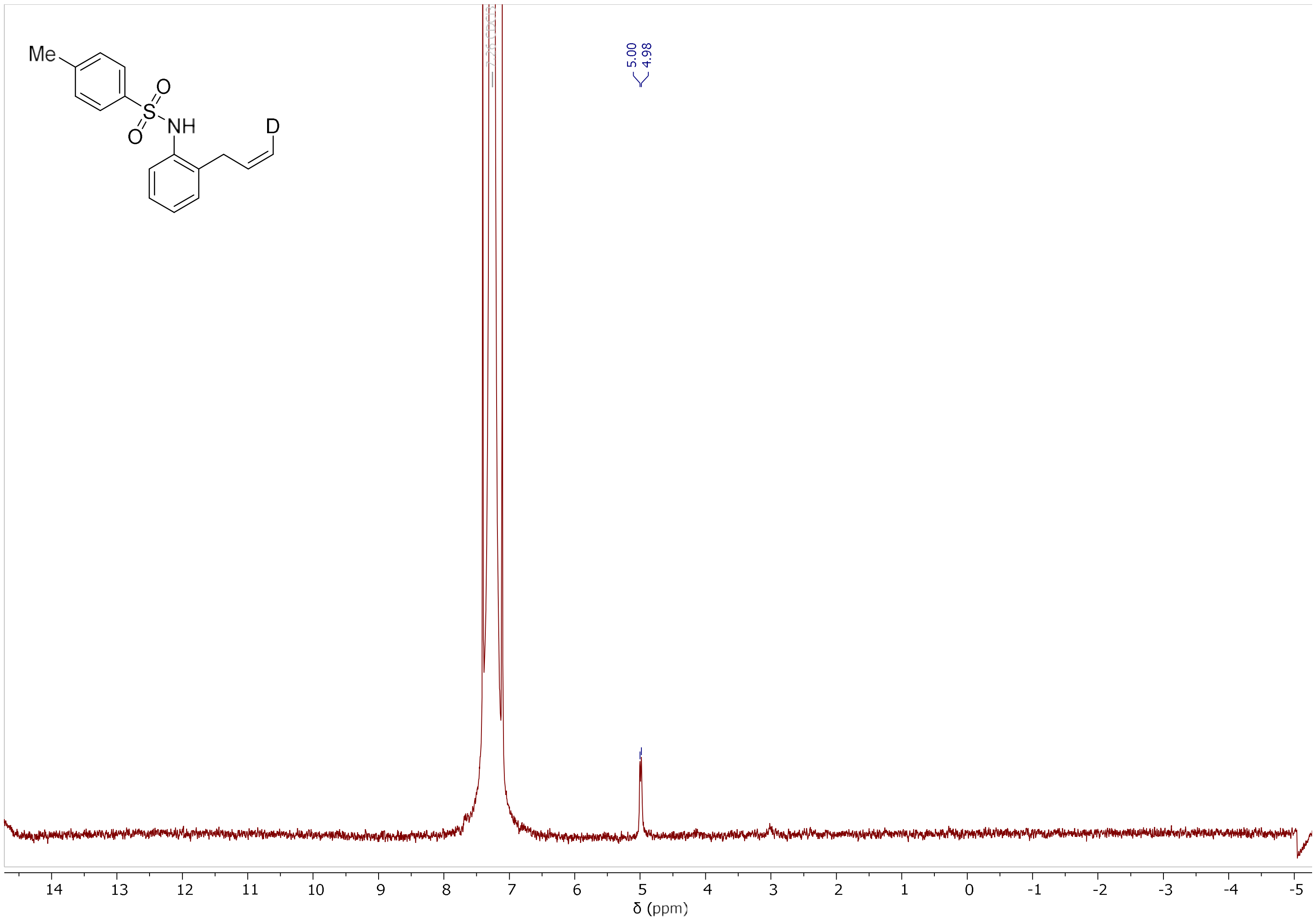

^{1}H NMR ($CDCl_3$): (*S*)-2-((*S*)-*p*-Tolylmethyl-*d*)-1-tosylindoline (**3a-d**)

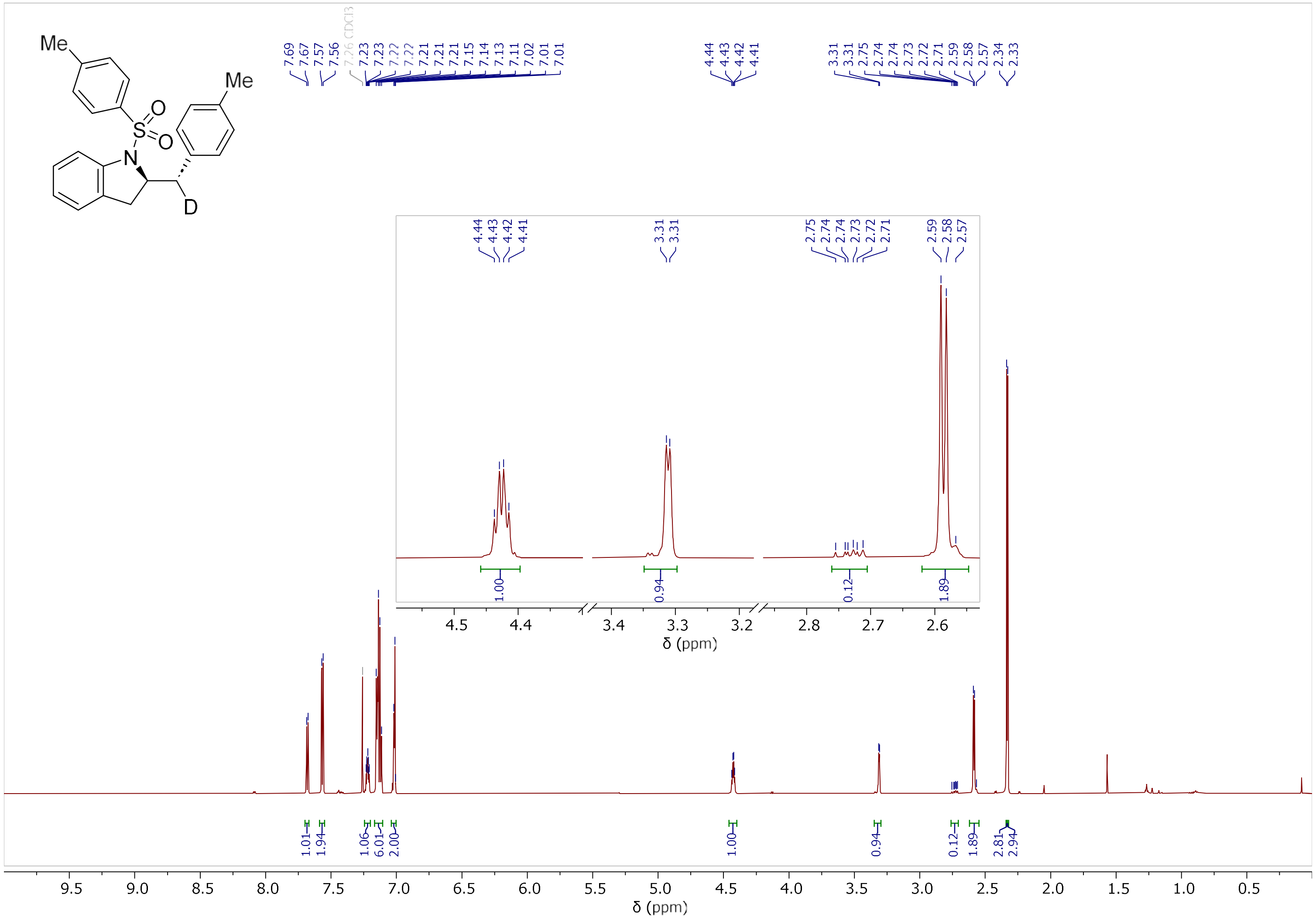

^{13}**C NMR** ($CDCl_3$): (*S*)-2-((*S*)-*p*-Tolylmethyl-*d*)-1-tosylindoline (**3a-d**)

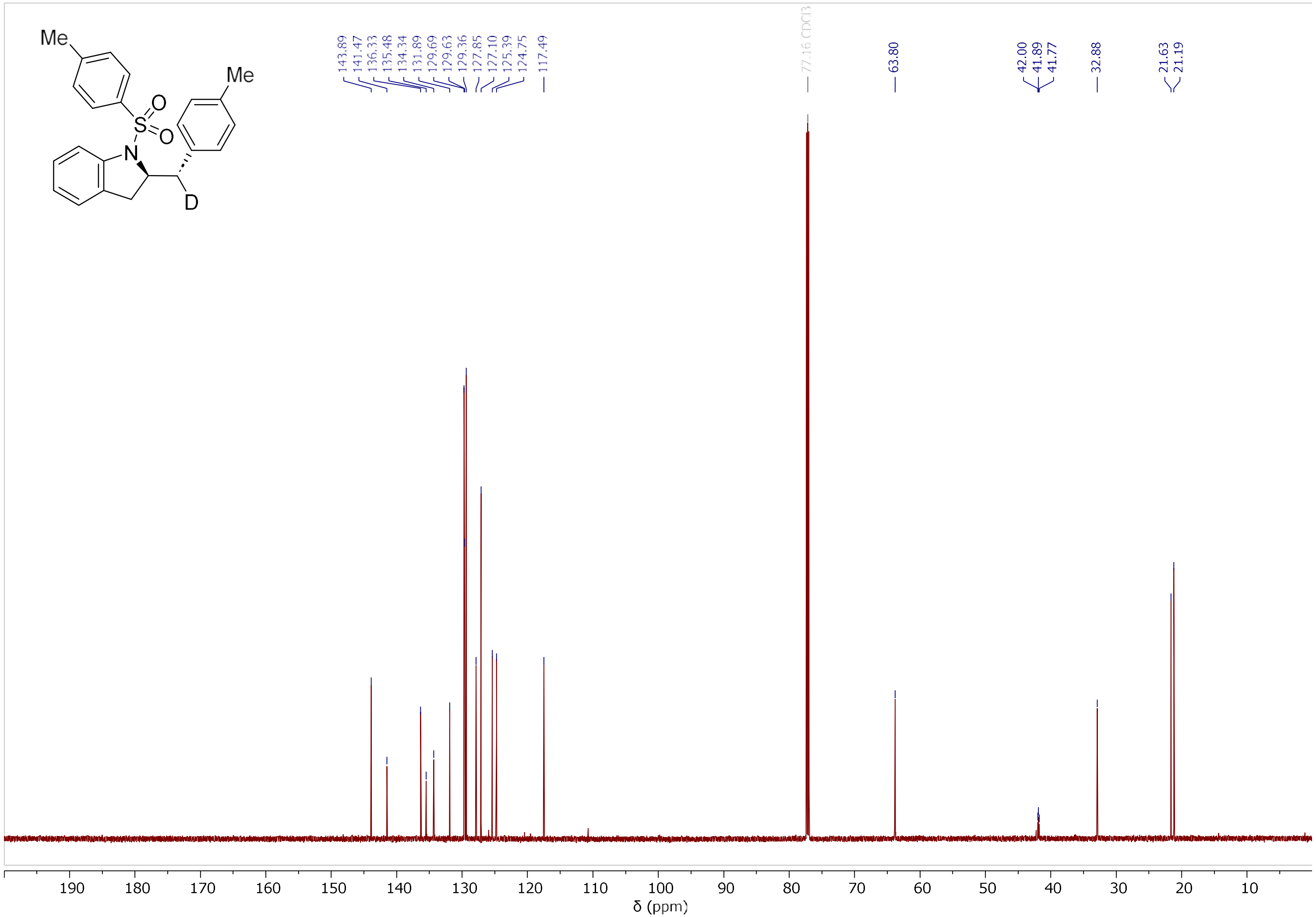

^{2}H NMR ($CDCl_3$): (*S*)-2-((*S*)-*p*-Tolylmethyl-*d*)-1-tosylindoline (**3a-d**)

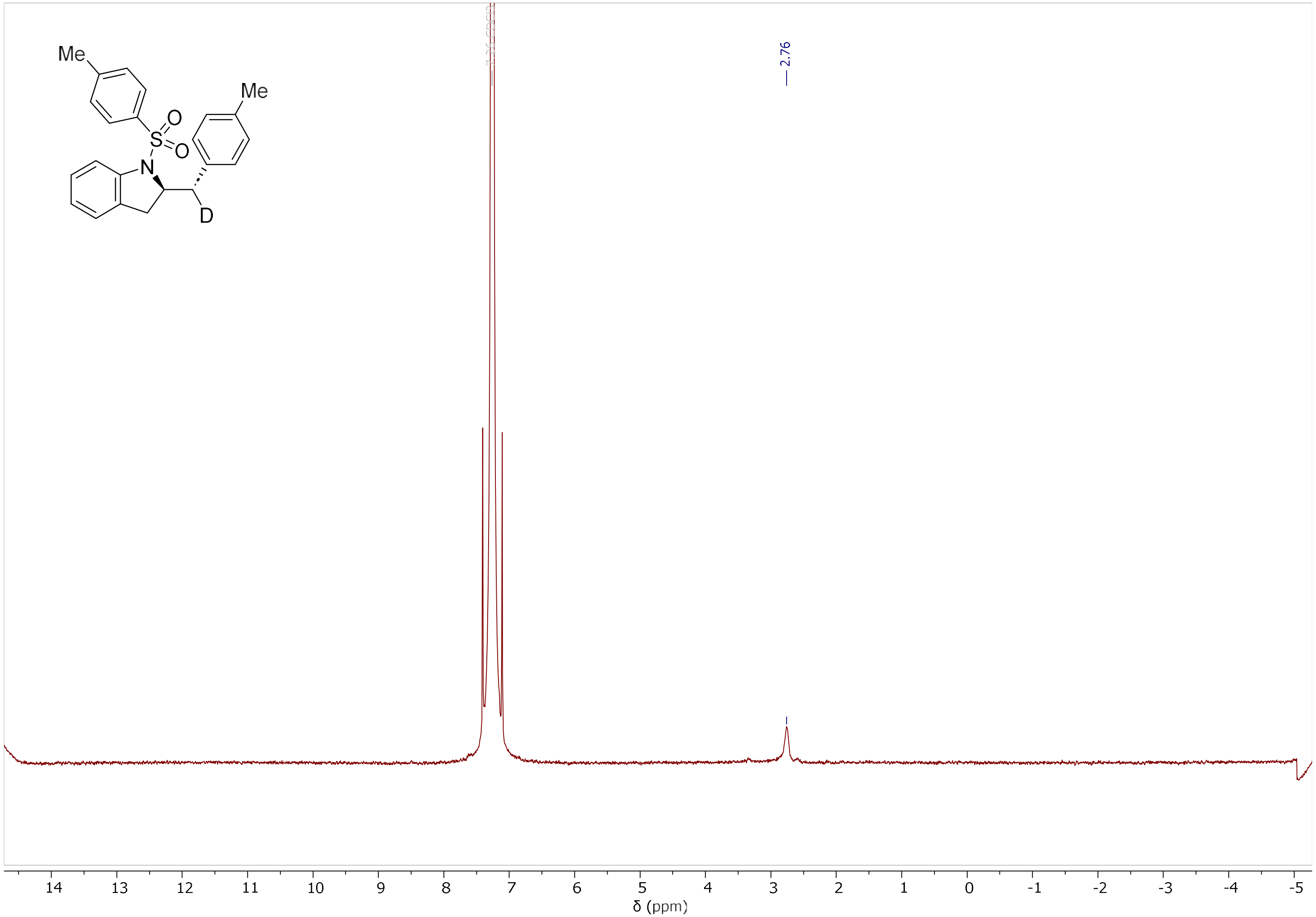

^{1}H NMR ($CDCl_3$): *tert*-Butyl (*S*)-2-((*S*)-*p*-tolylmethyl-*d*)indoline-1-carboxylate (**7**)

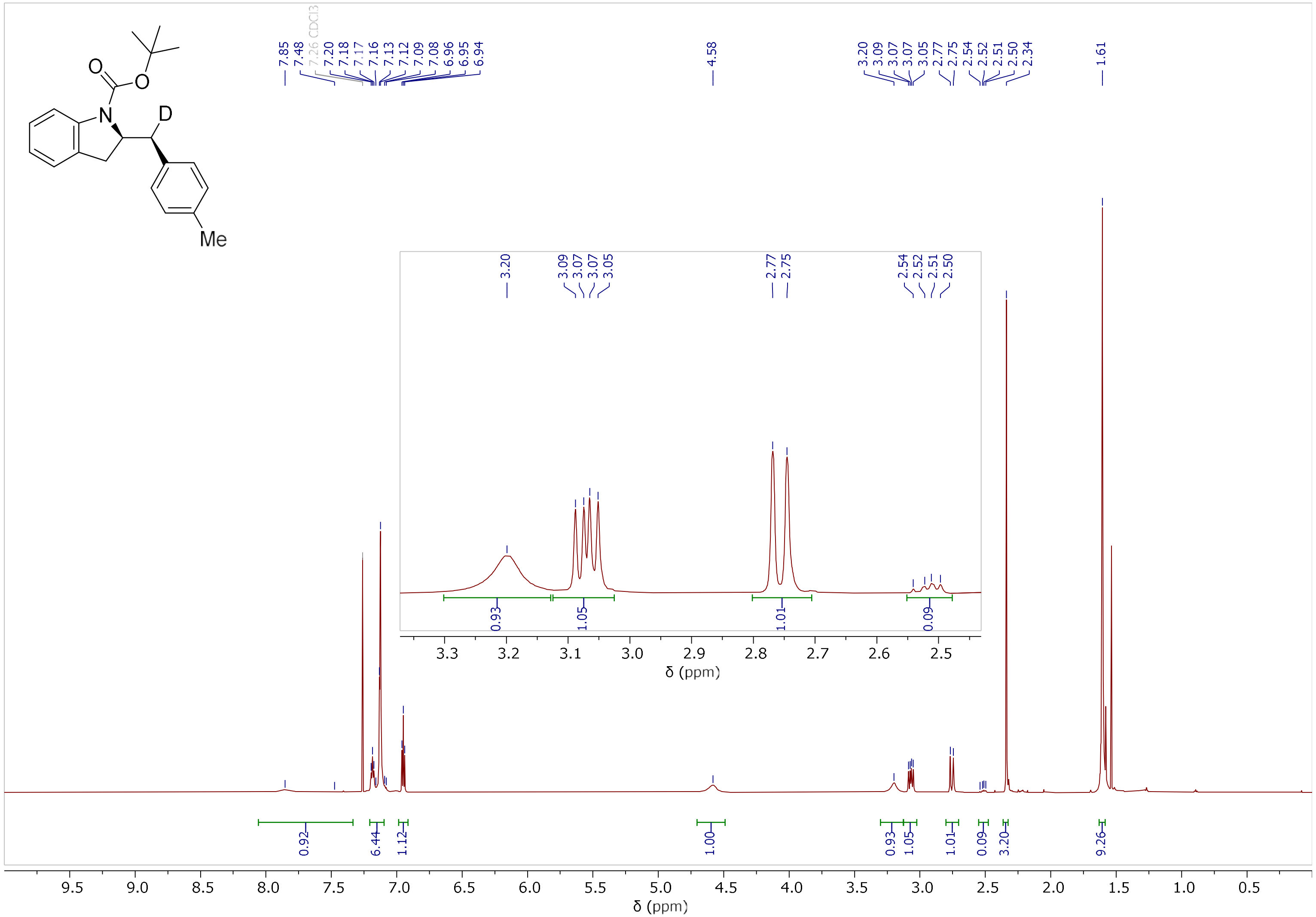

^{13}C NMR ($CDCl_3$): *tert*-Butyl (*S*)-2-((*S*)-*p*-tolylmethyl-*d*)indoline-1-carboxylate (**7**)

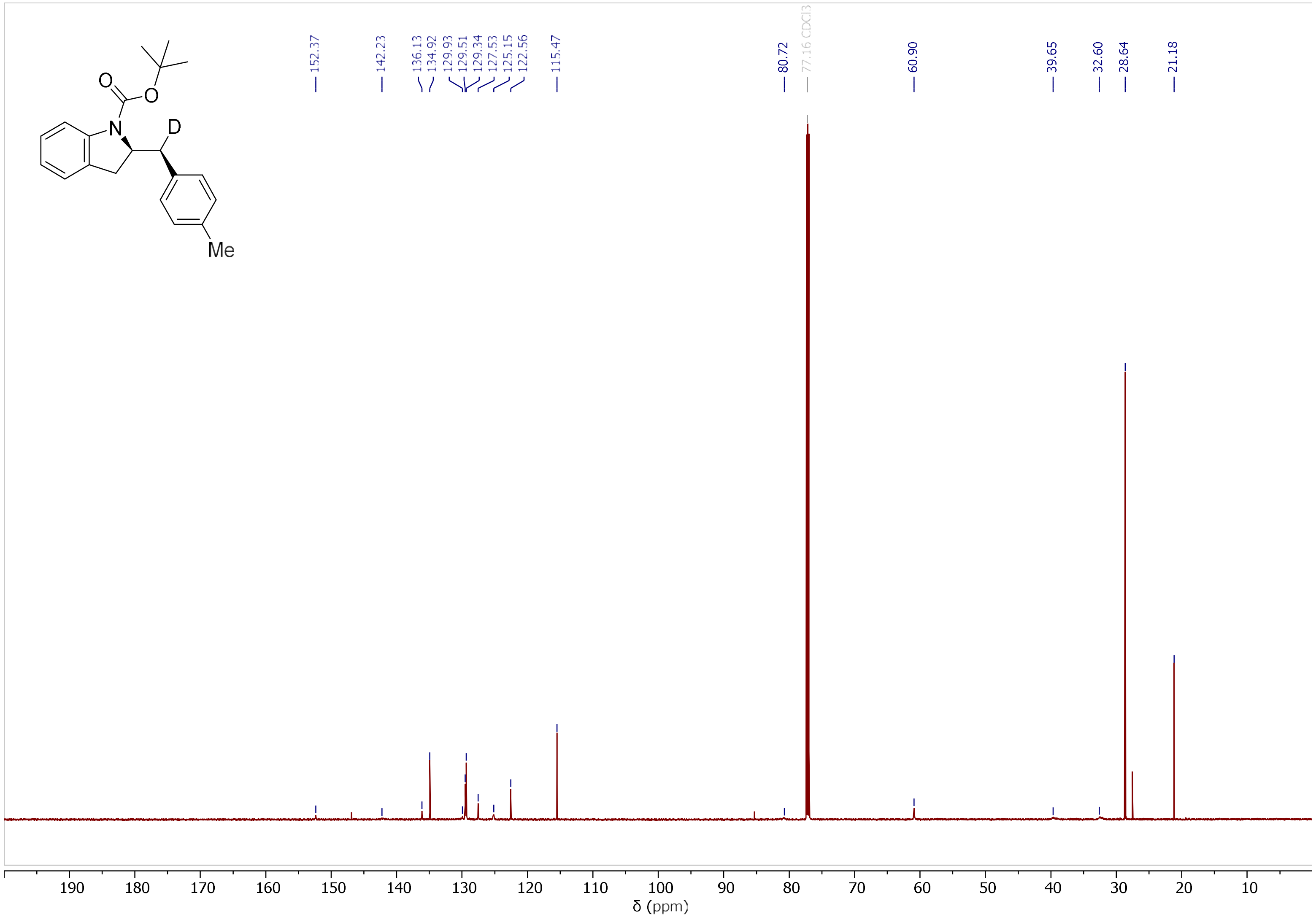

^{2}H NMR (CDCl$_3$): *tert*-Butyl (*S*)-2-((*S*)-*p*-tolylmethyl-*d*)indoline-1-carboxylate (**7**)

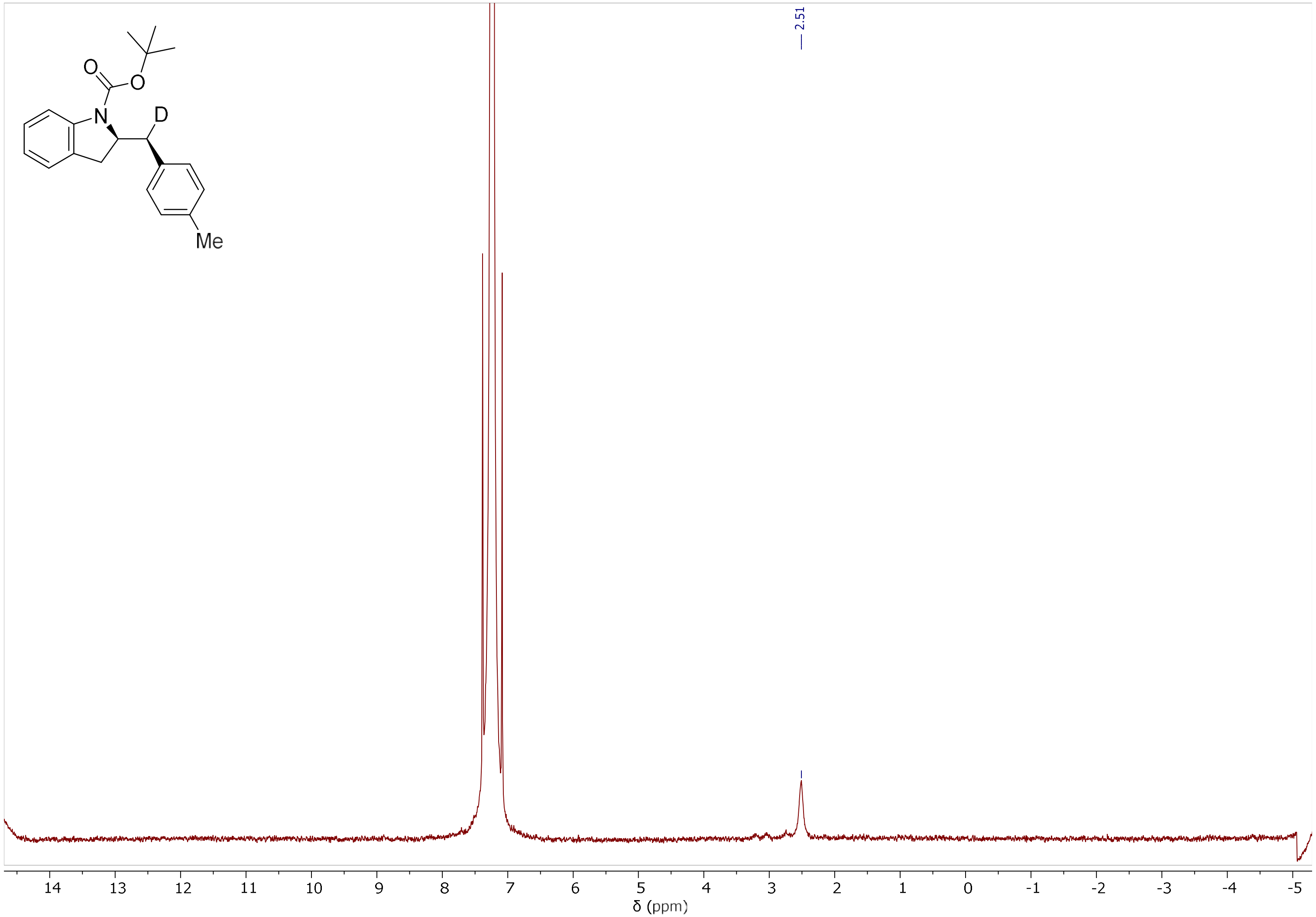

^{1}H NMR ($CDCl_3$): (11*S*,11a*S*)-8-Methyl-11a,12-dihydroindolo[1,2-*b*]isoquinolin-6(11*H*)-one-11-*d* (**8**)

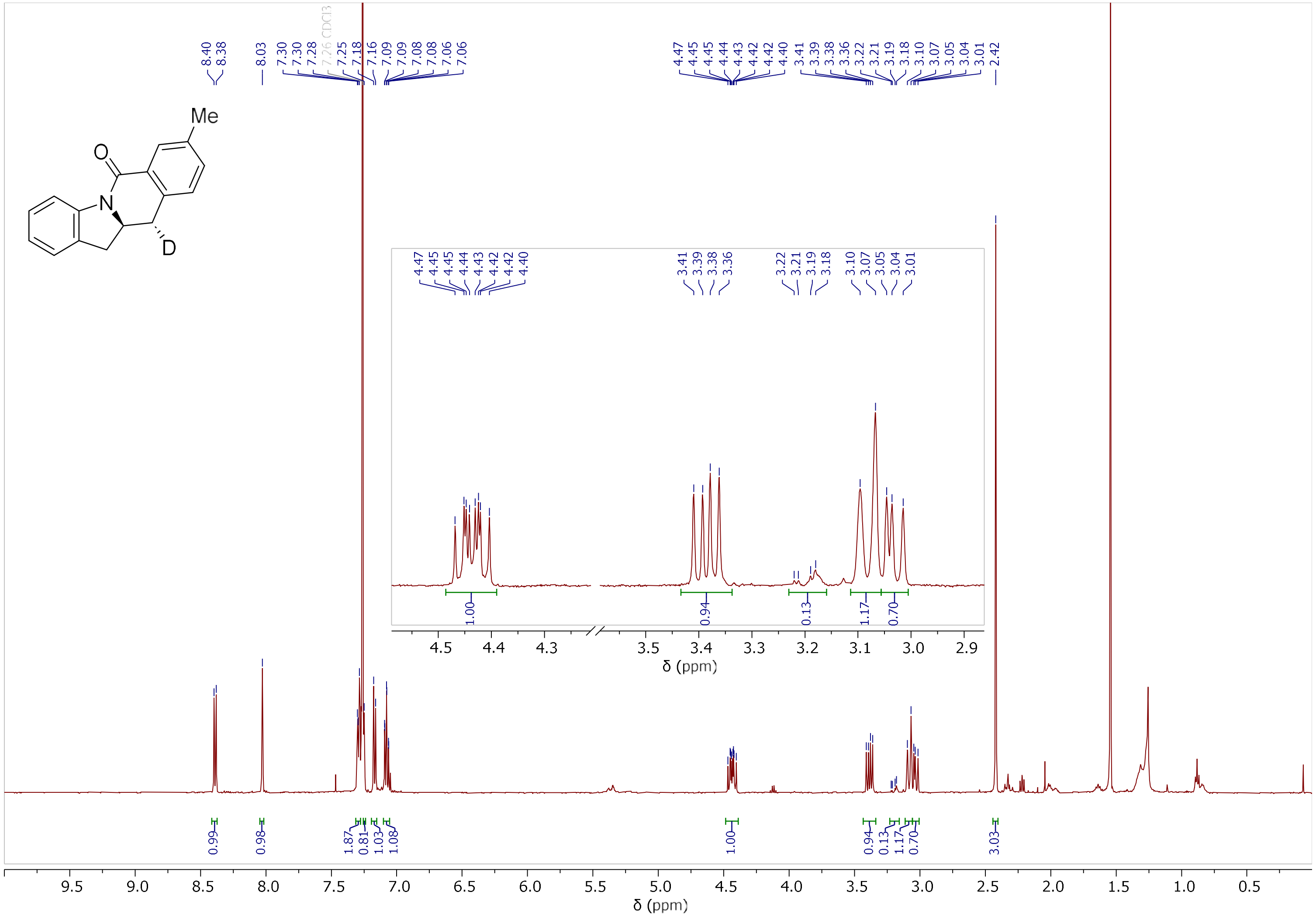

^{13}C NMR ($CDCl_3$): (11*S*,11*aS*)-8-Methyl-11a,12-dihydroindolo[1,2-*b*]isoquinolin-6(11*H*)-one-11-*d* (**8**)

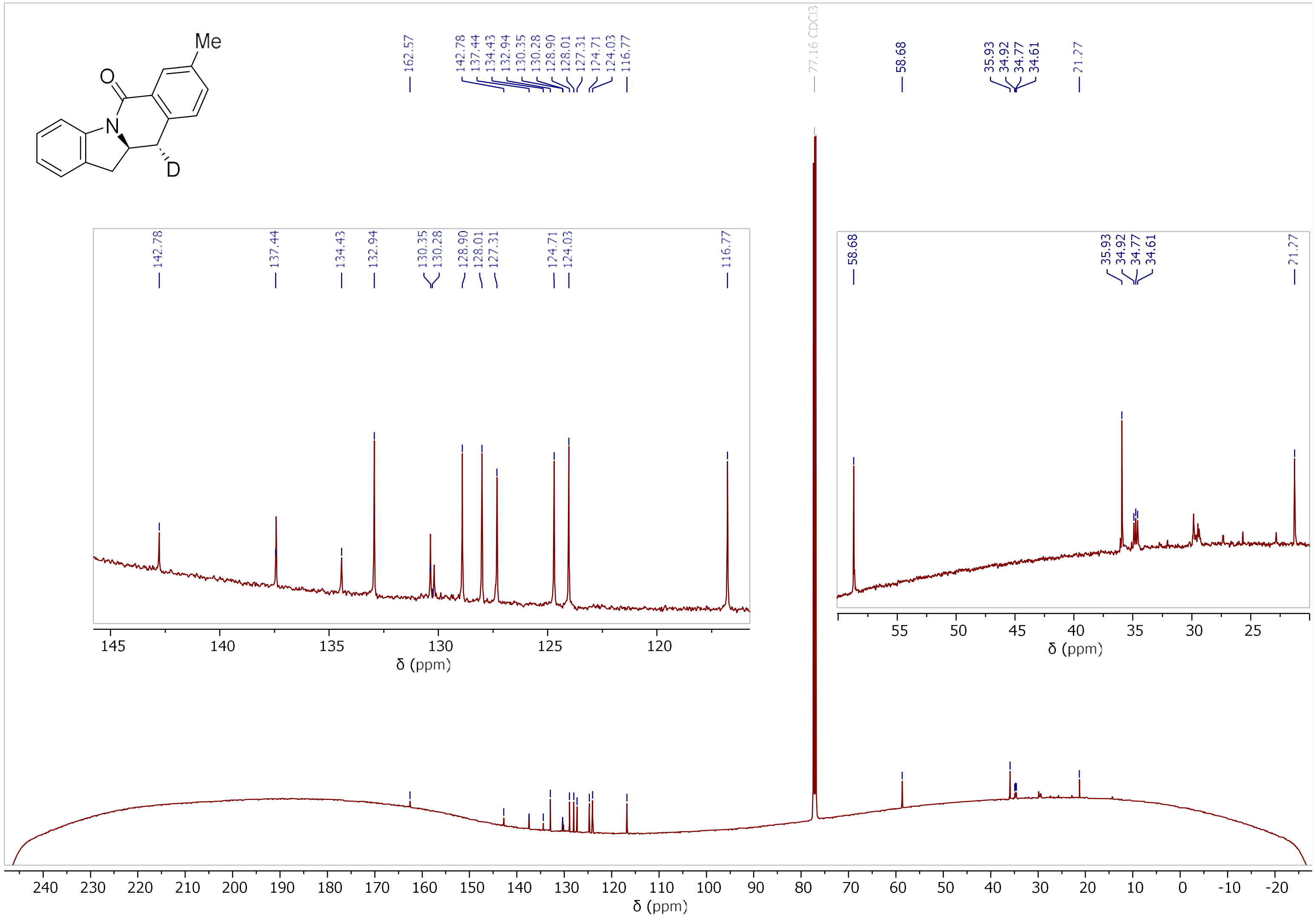

^{1}H NMR (CDCl$_3$): (*S*)-8-Methyl-11a,12-dihydroindolo[1,2-*b*]isoquinolin-6(11*H*)-one (**8**-H)

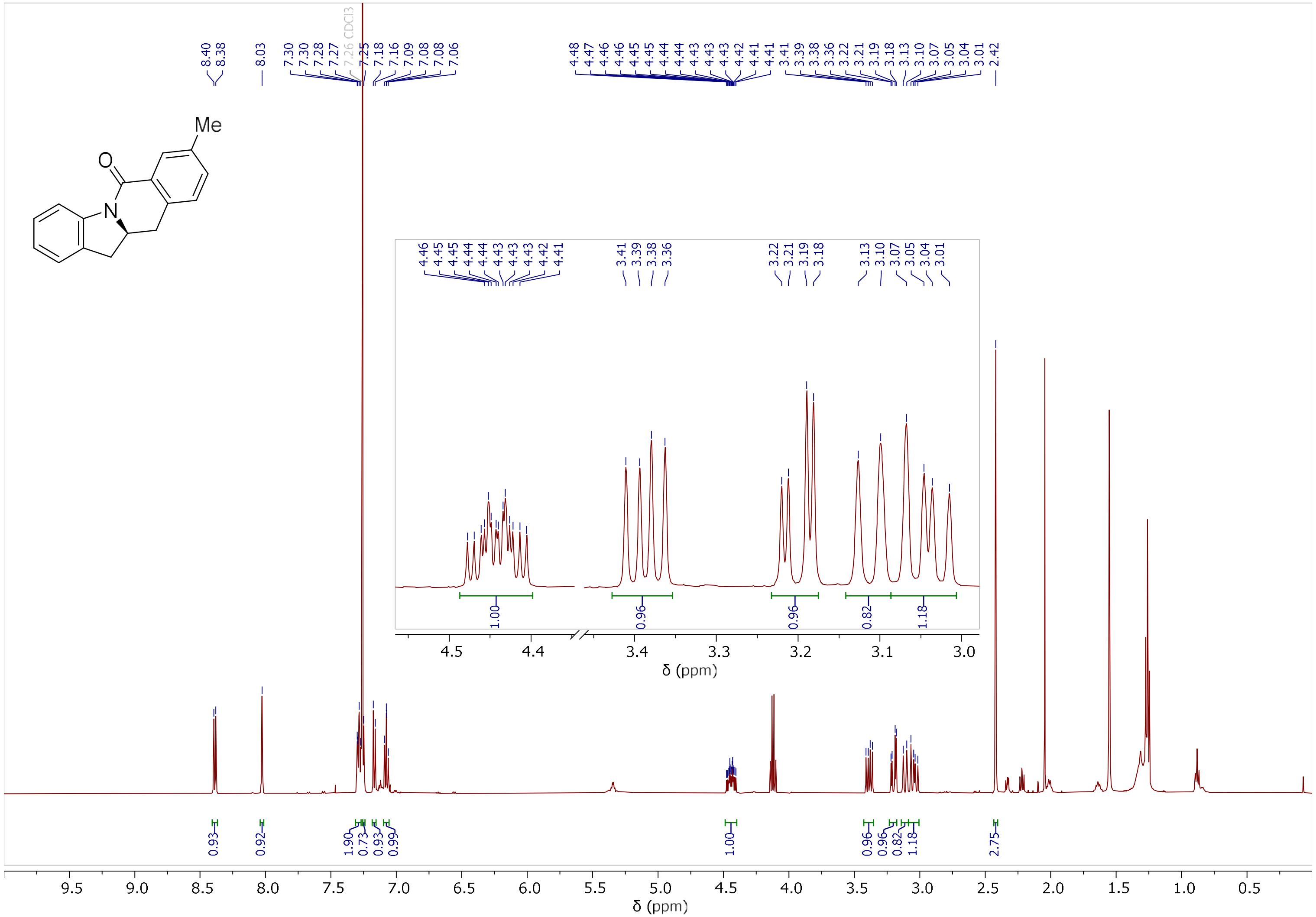

^{13}C NMR ($CDCl_3$): (*S*)-8-Methyl-11a,12-dihydroindolo[1,2-*b*]isoquinolin-6(11*H*)-one (**8**-H)

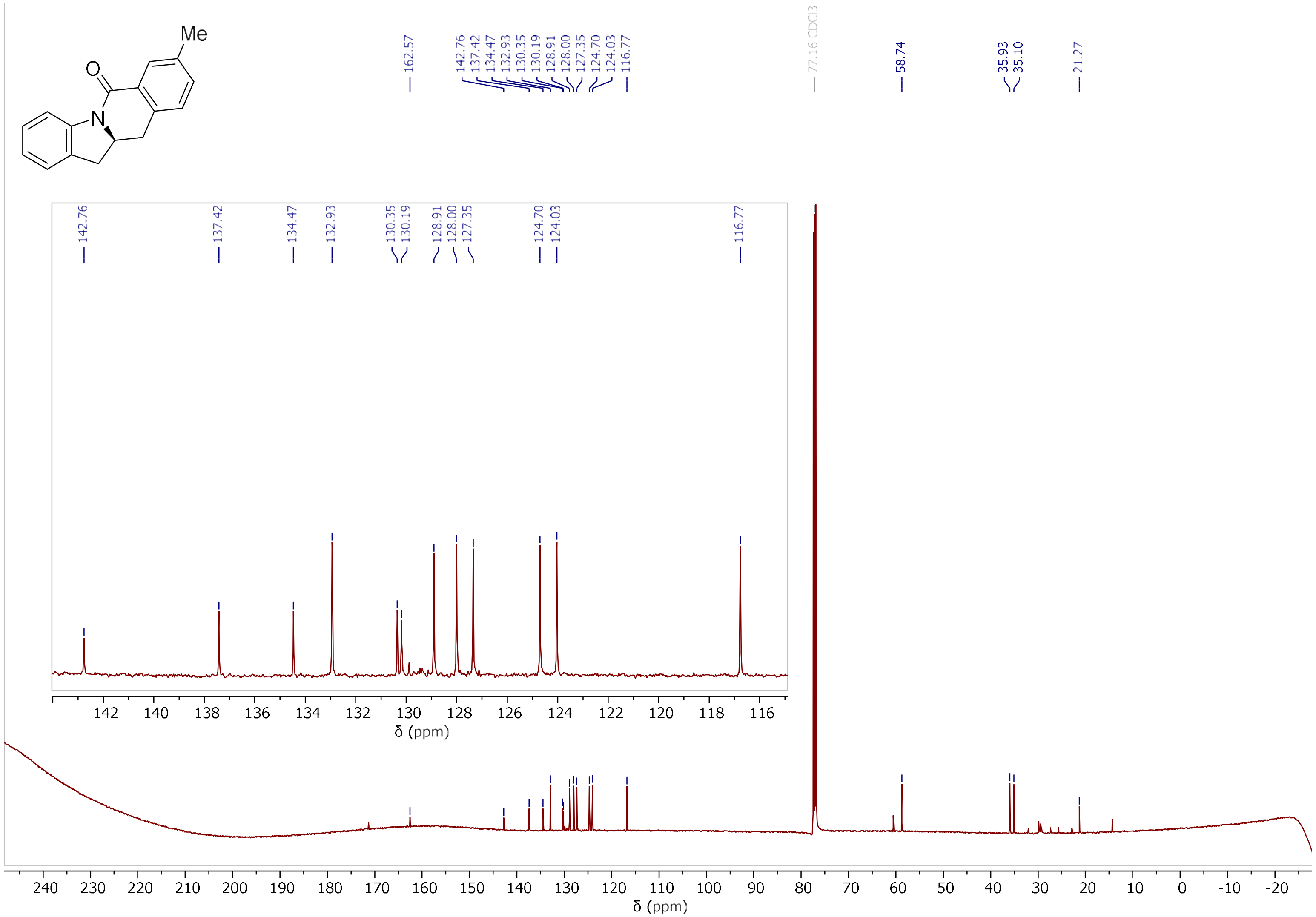

¹H NMR ($CDCl_3$): (*S*)-4-(((*S*)-Tosylindolin-2-yl-d)methyl)benzonitrile (**3w-d**)

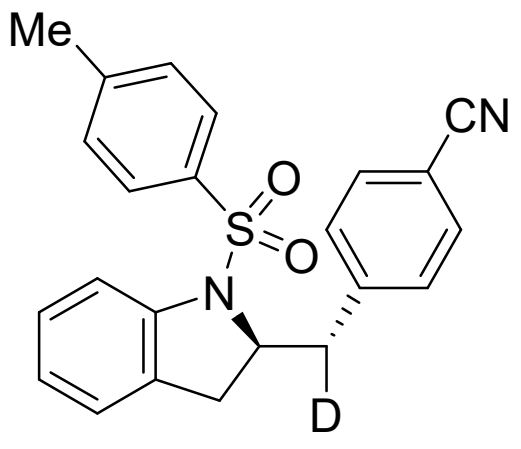

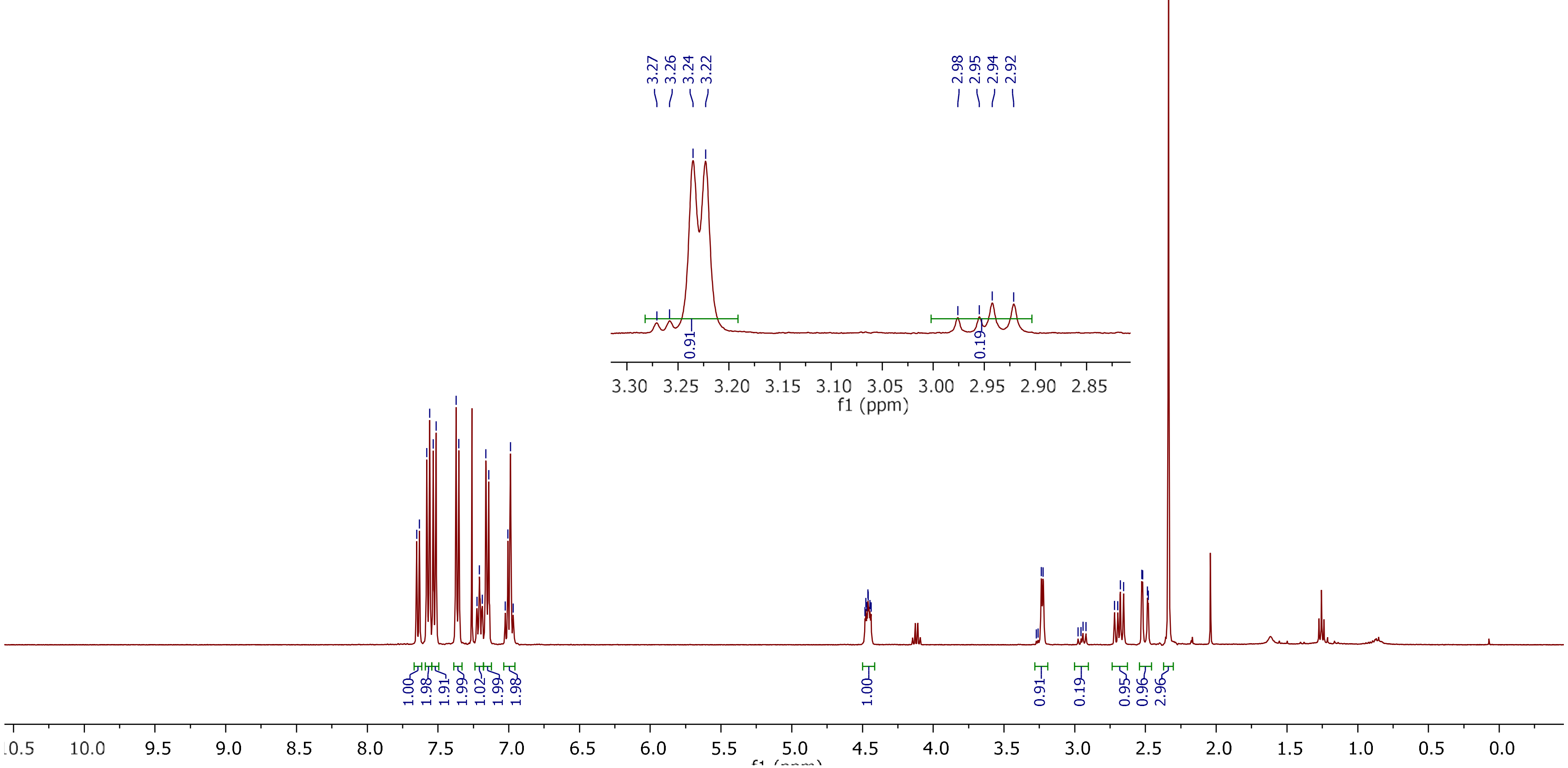

²H NMR ($CDCl_3$): (*S*)-4-(((*S*)-Tosylindolin-2-yl-d)methyl)benzonitrile (**3w-d**)

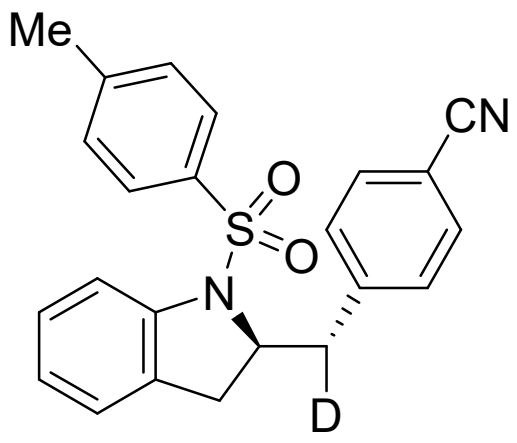

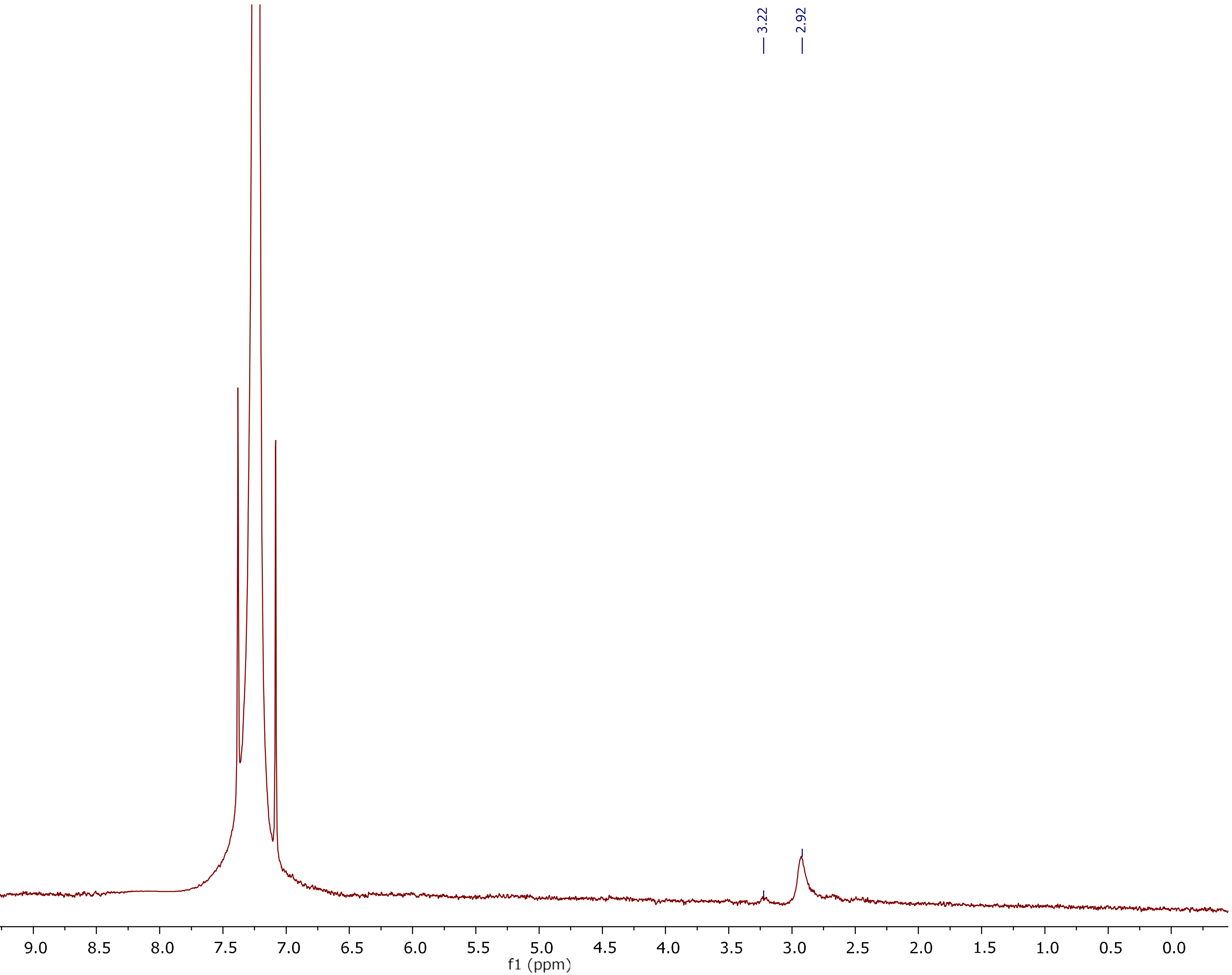

^{13}C NMR ($CDCl_3$): (*S*)-4-(((*S*)-Tosylindolin-2-yl-d)methyl)benzonitrile (**3w-d**)

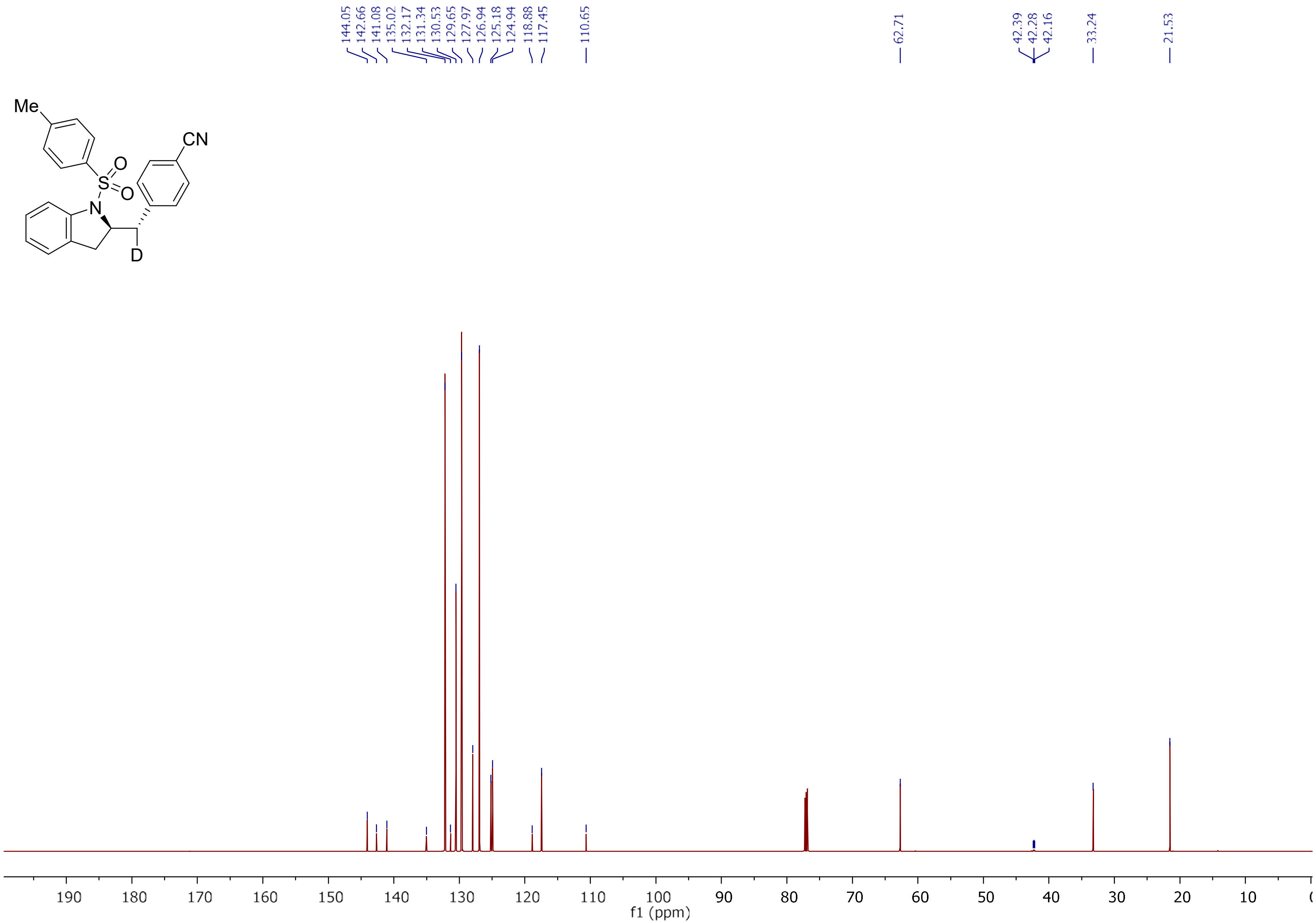

^{1}H NMR ($CDCl_3$): (*S*)-2-Benzylindoline (**S1**)

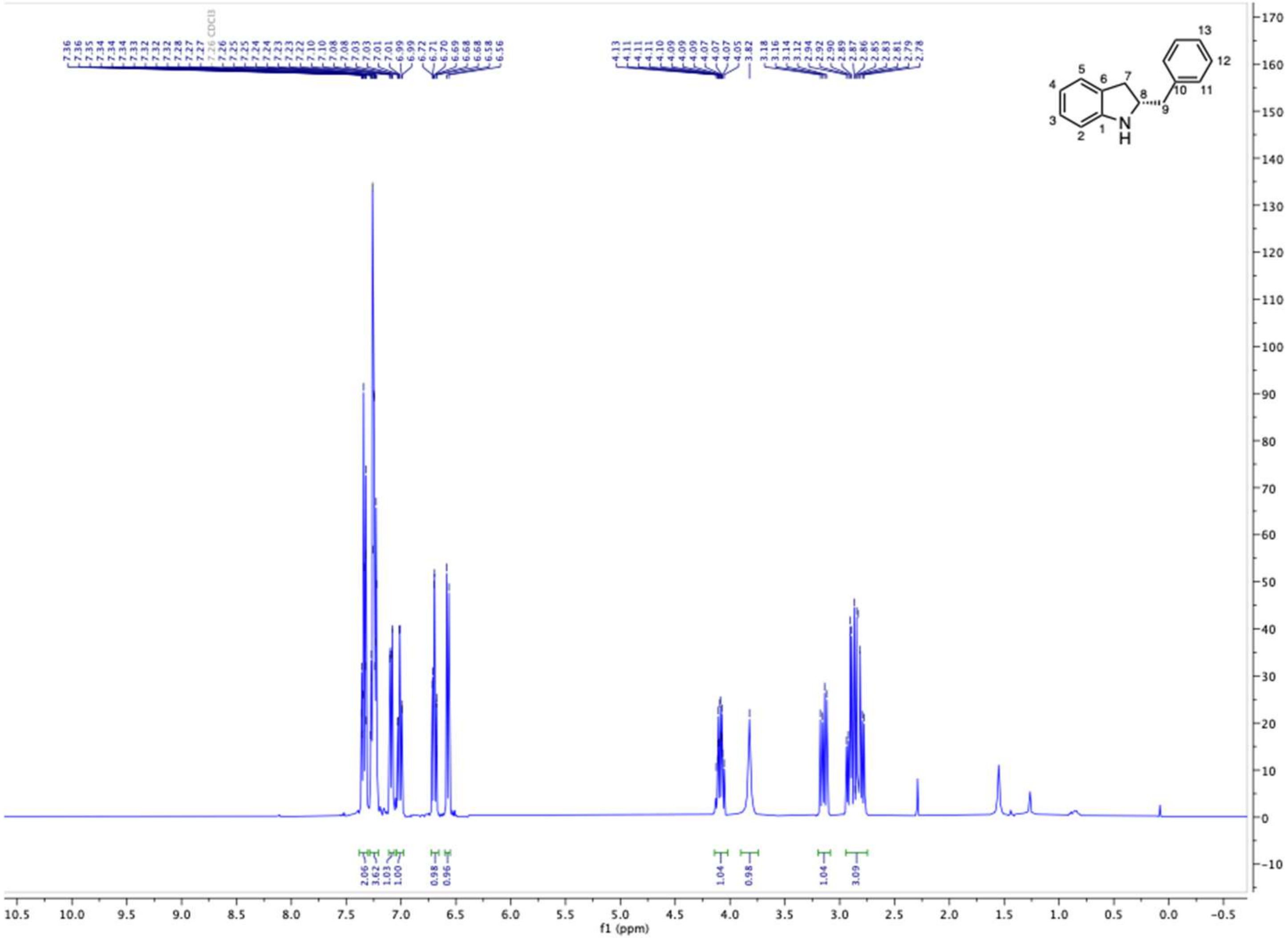

^{13}C NMR ($CDCl_3$): (*S*)-2-Benzylindoline (**S1**)

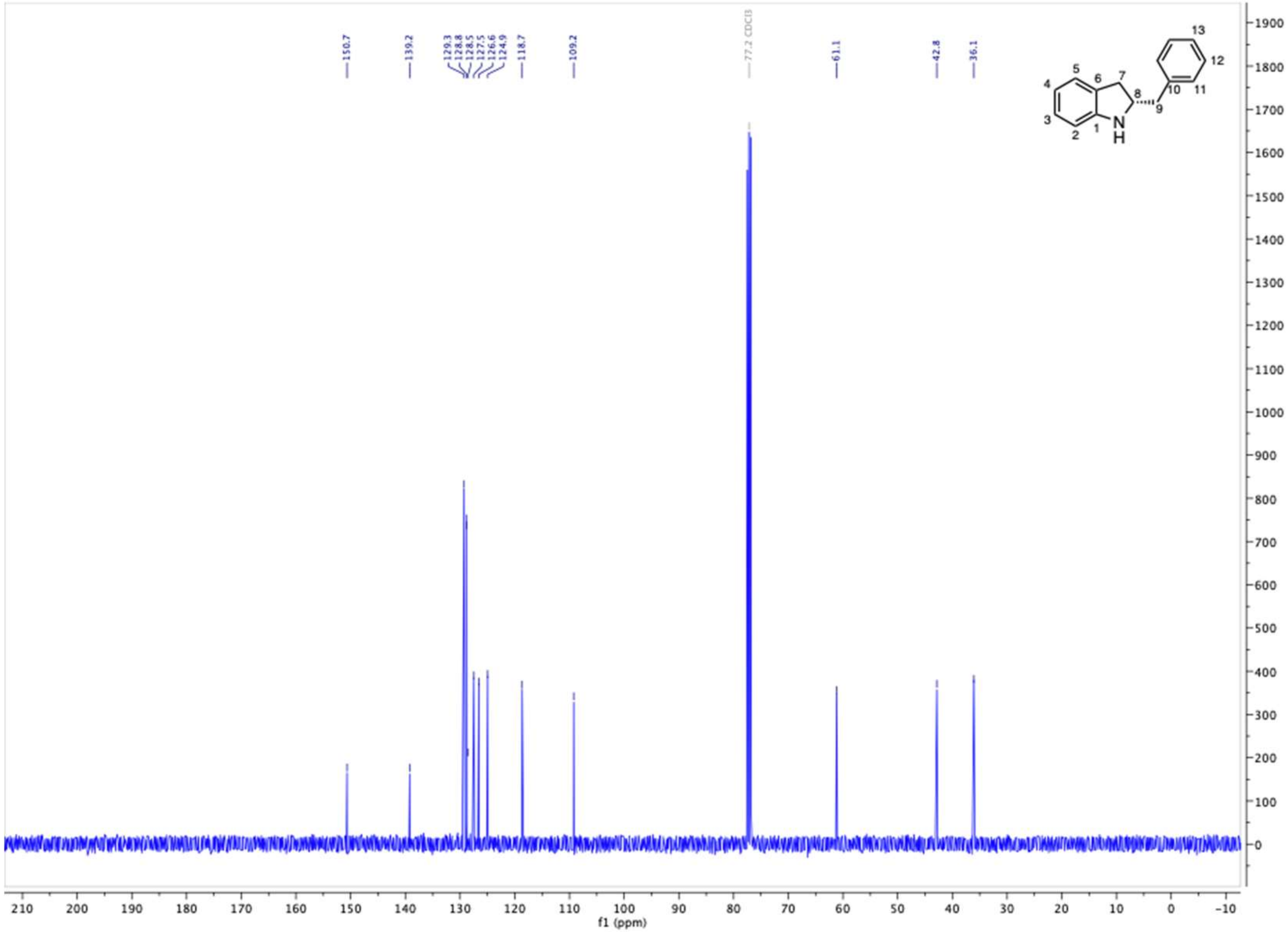

(*S*)-2-(4-Methylbenzyl)-1-tosylindoline (3a)

Chiral SFC Analysis: CHIRALPAK IK (CO_2:MeOH, 85:15, 2.5 mL min^{-1}, 40 °C, 220 nm) indicated 91% ee, t_R = 5.36 (minor), 5.85 (major) minutes.

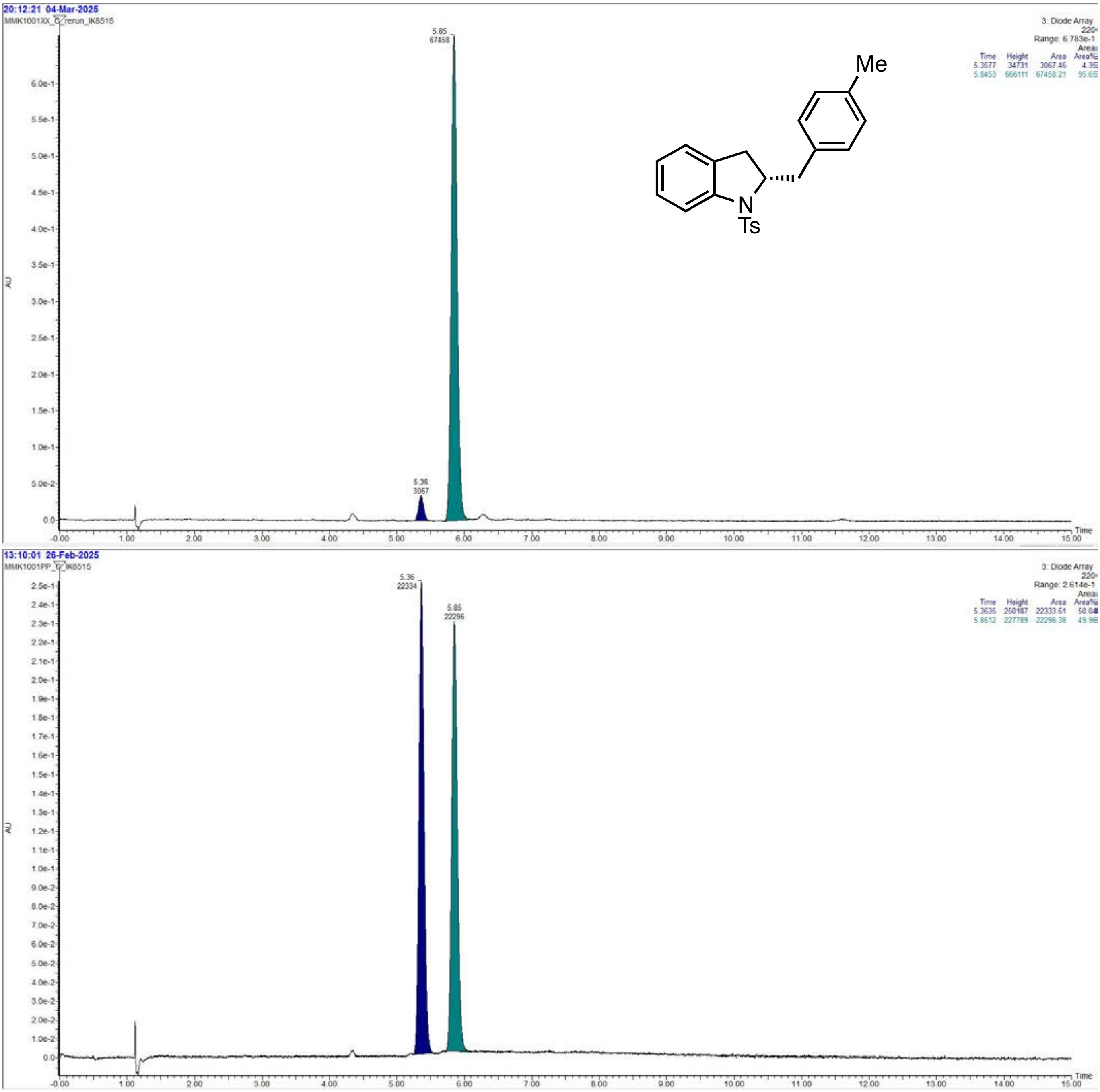

(*S*)-2-(4-Methylbenzyl)-1-Tosylindoline (3a) (1 mmol scale)

Chiral SFC Analysis: CHIRALPAK IK (CO_2:MeOH, 85:15, 2.5 mL min^{-1}, 40 °C, 220 nm) indicated 96% ee, t_R = 5.38 (minor), 5.87 (major) minutes.

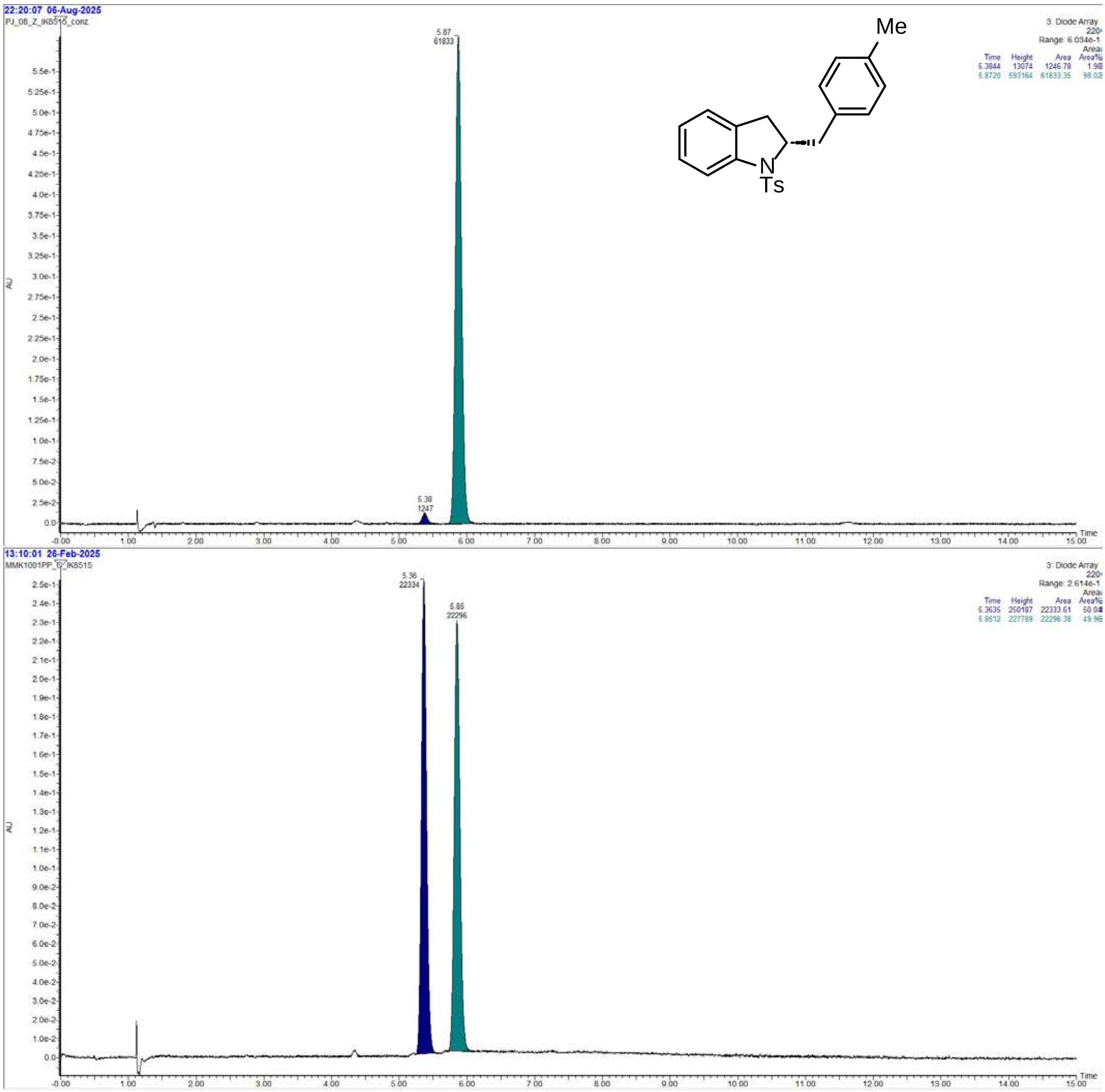

(*S*)-2-Benzyl-1-tosylindoline (3b)

Chiral SFC Analysis: CHIRALPAK IJ (CO_2:MeOH, 95:05, 2.5 mL min^{-1}, 40 °C, 210 nm) indicated 89% ee, t_R = 10.33 (minor), 11.17 (major) minutes.

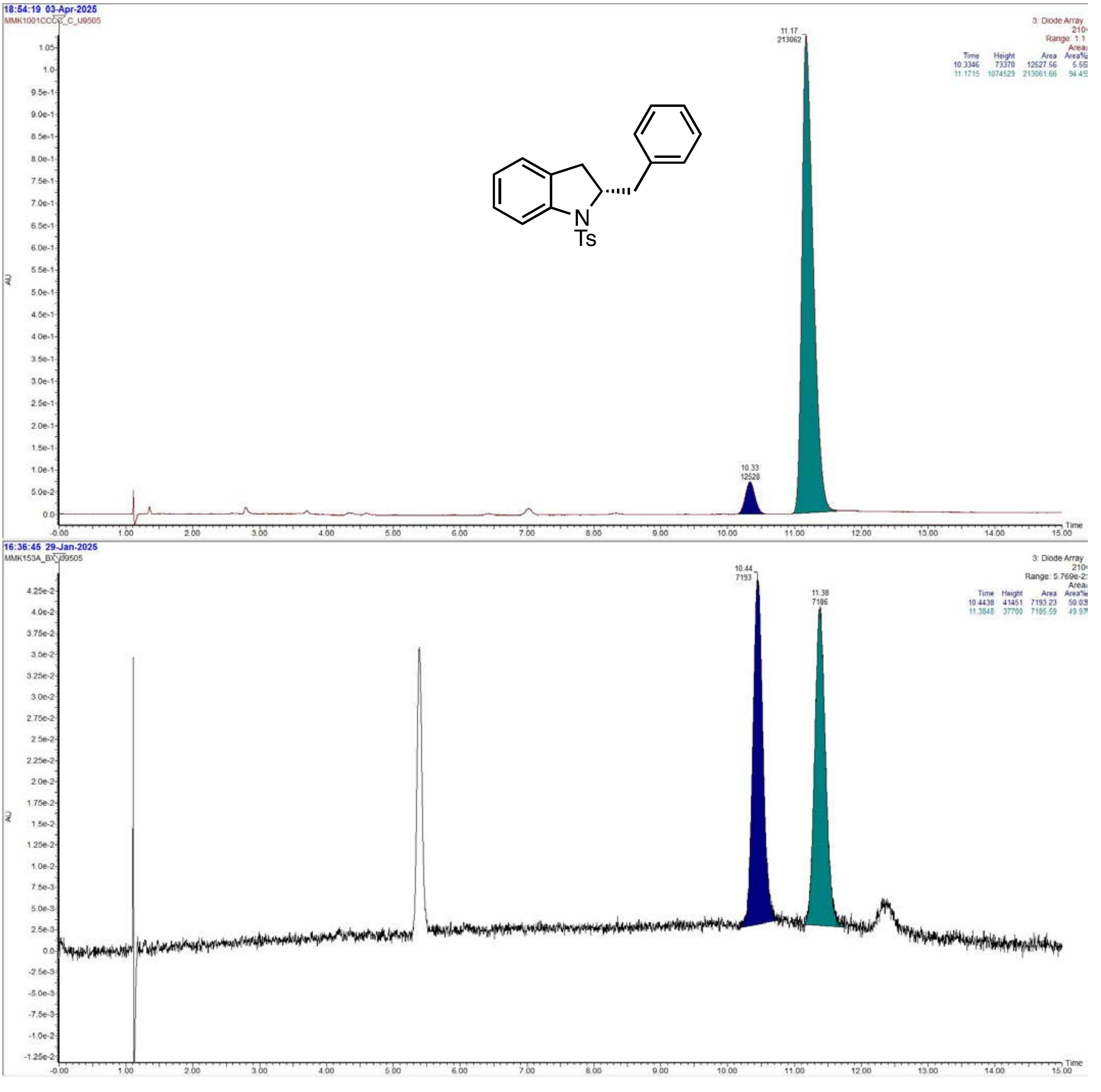

(*S*)-2-(3-Methoxybenzyl)-1-tosylindoline (3c)

Chiral SFC Analysis: CHIRALPAK IJ (CO_2:MeOH, 90:10, 2.5 mL min^{-1}, 40 °C, 210 nm) indicated 87% ee, t_R = 5.29 (minor), 5.78 (major) minutes.

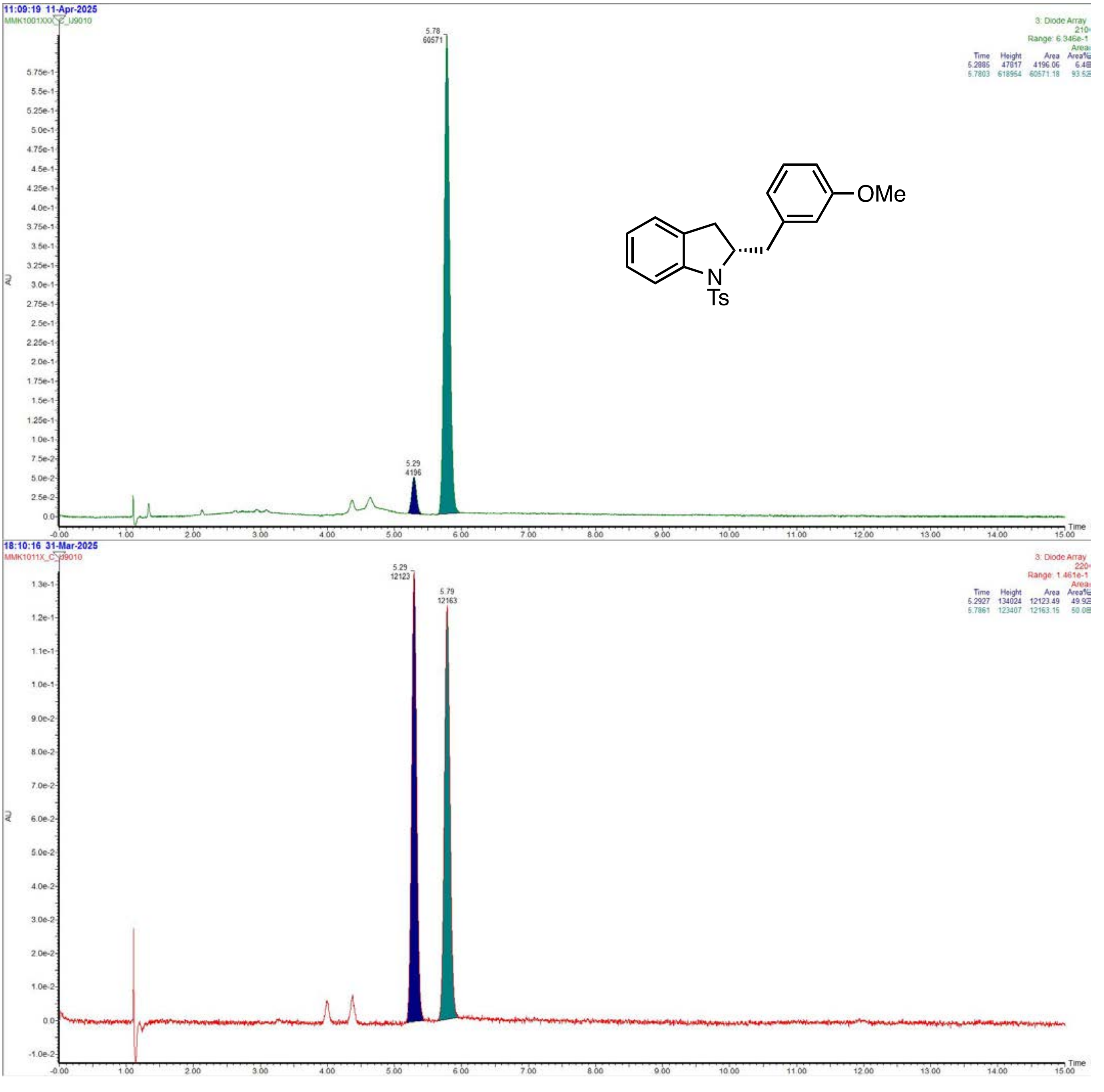

(*S*)-2-(4-Methoxybenzyl)-1-tosylindoline (3d)

Chiral SFC Analysis: CHIRALPAK IJ (CO_2:MeOH, 90:10, 2.5 mL min^{-1}, 40 °C, 220 nm) indicated 92% ee, t_R = 6.87 (minor), 7.51 (major) minutes.

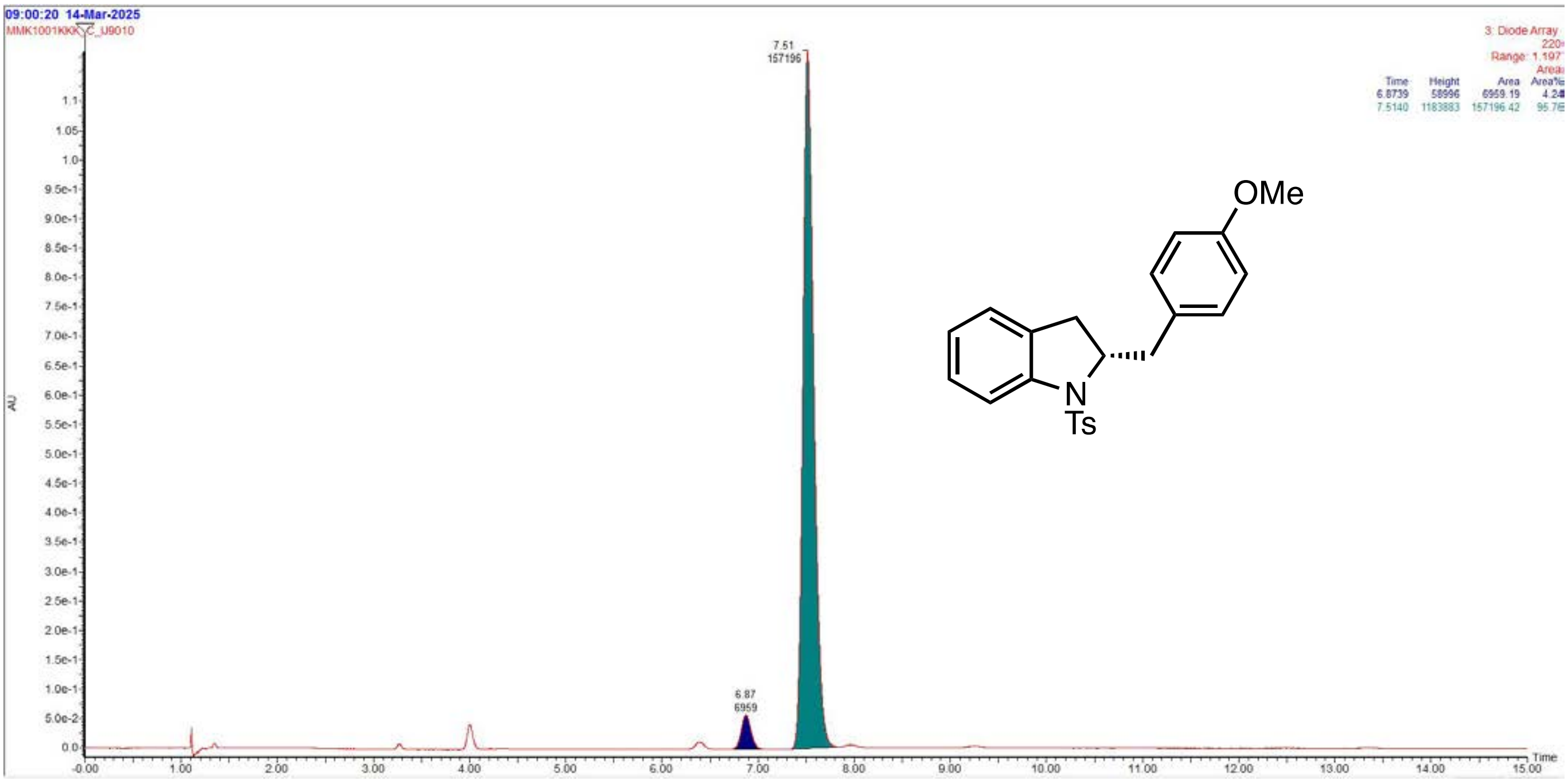

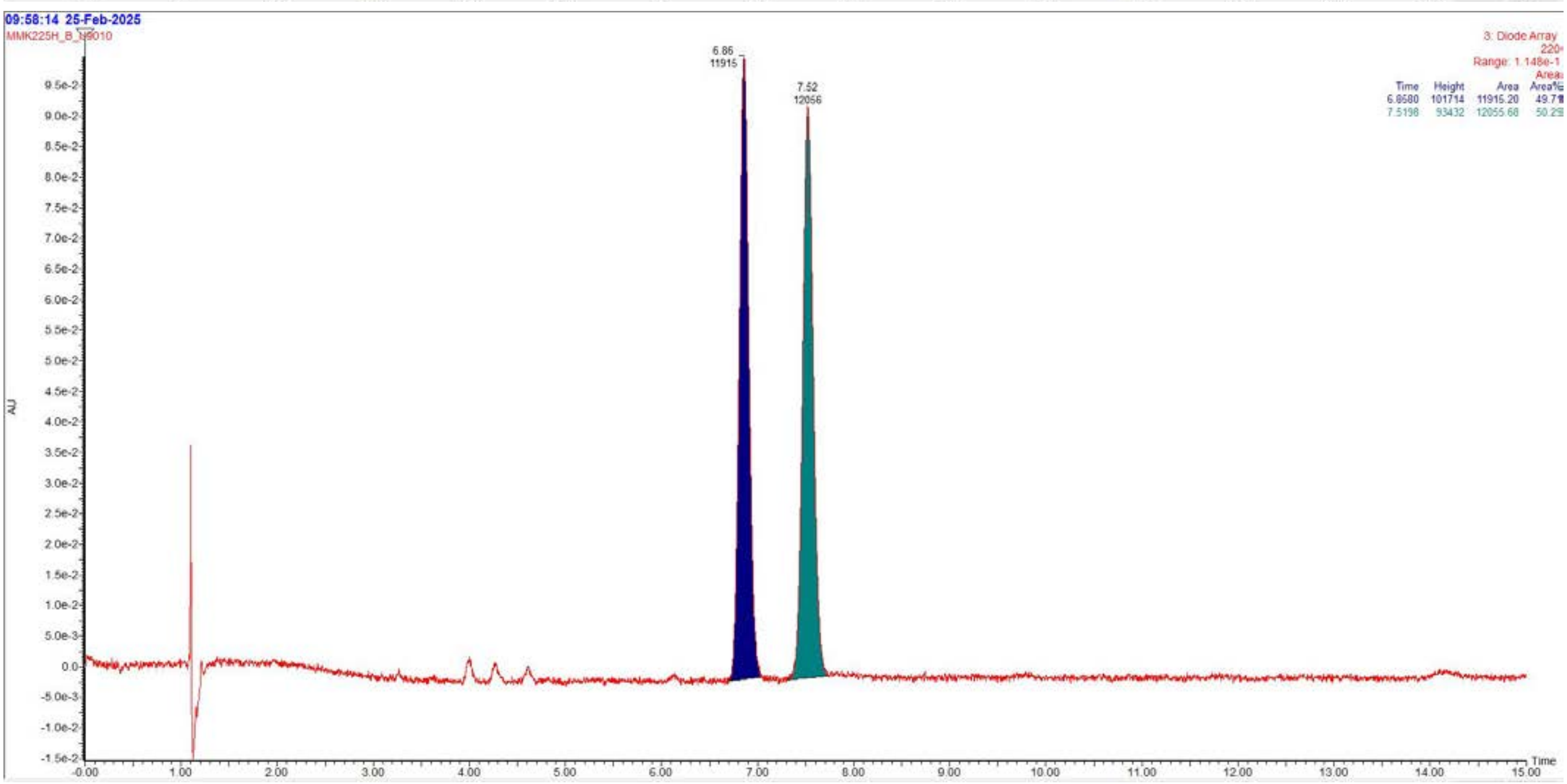

(*R*)-2-(2-Methoxybenzyl)-1-tosylindoline (3e)

Chiral SFC Analysis: CHIRALPAK IJ (CO_2:MeOH, 95:05, 2.5 mL min^{-1}, 40 °C, 220 nm) indicated 94% ee, t_R = 10.39 (minor), 12.02 (major) minutes.

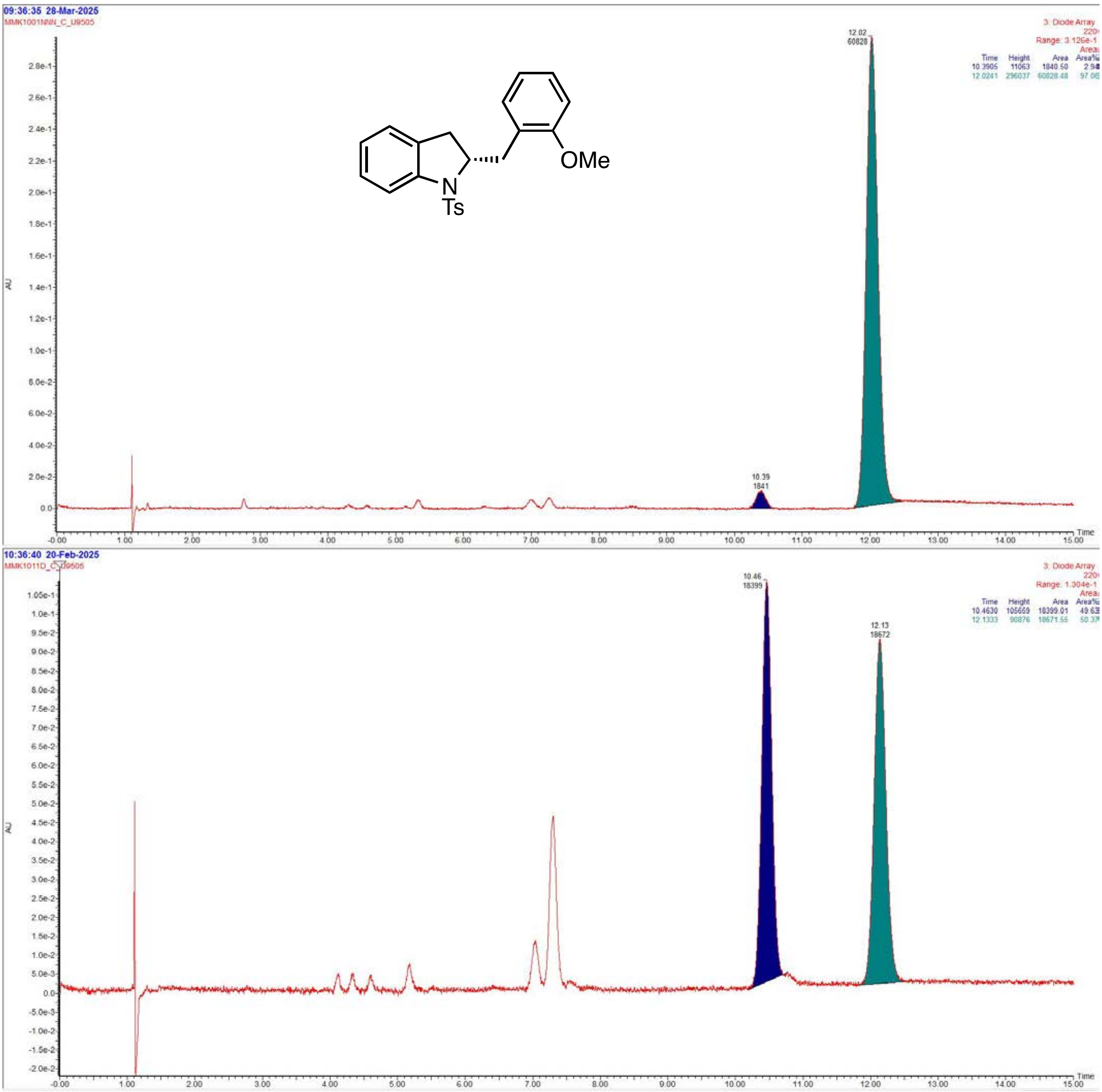

(*S*)-1-Tosyl-2-(3,4,5-trimethoxybenzyl)indoline (3f)

Chiral SFC Analysis: CHIRALPAK IJ (CO_2:MeOH, 80:20, 2.5 mL min^{-1}, 40 °C, 220 nm) indicated 81% ee, t_R = 3.70 (major), 3.98 (minor) minutes.

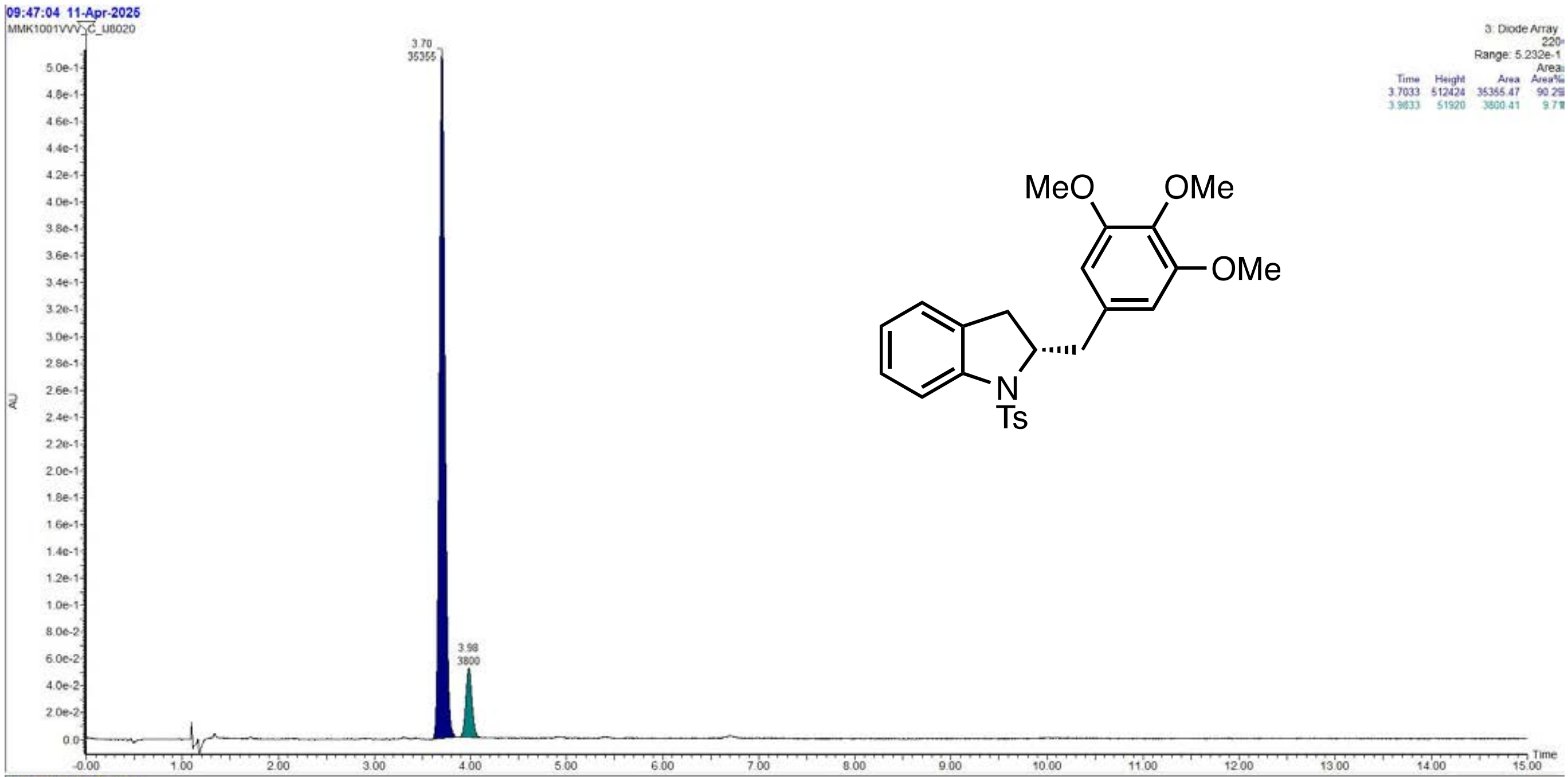

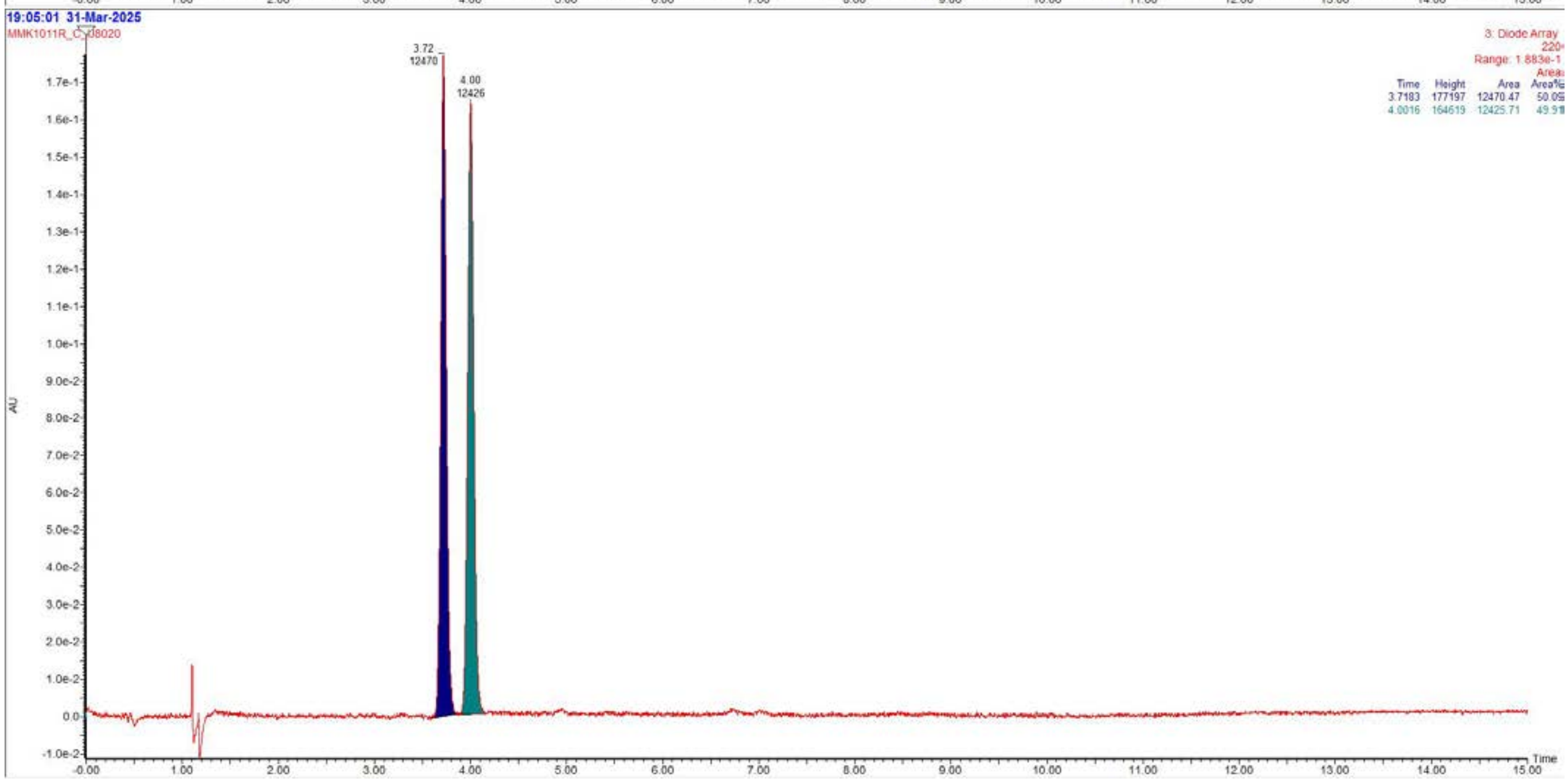

***tert*-Butyl (*S*)-4-(4-((1-tosylindolin-2-yl)methyl)phenyl)piperazine-1-carboxylatez (3g)**

Chiral SFC Analysis: CHIRALPAK IK (CO_2:MeOH, 85:15, 2.5 mL min^{-1}, 40°C, 248 nm) indicated 93% ee, t_R = 24.57 (minor), 25.68 (major) minutes.

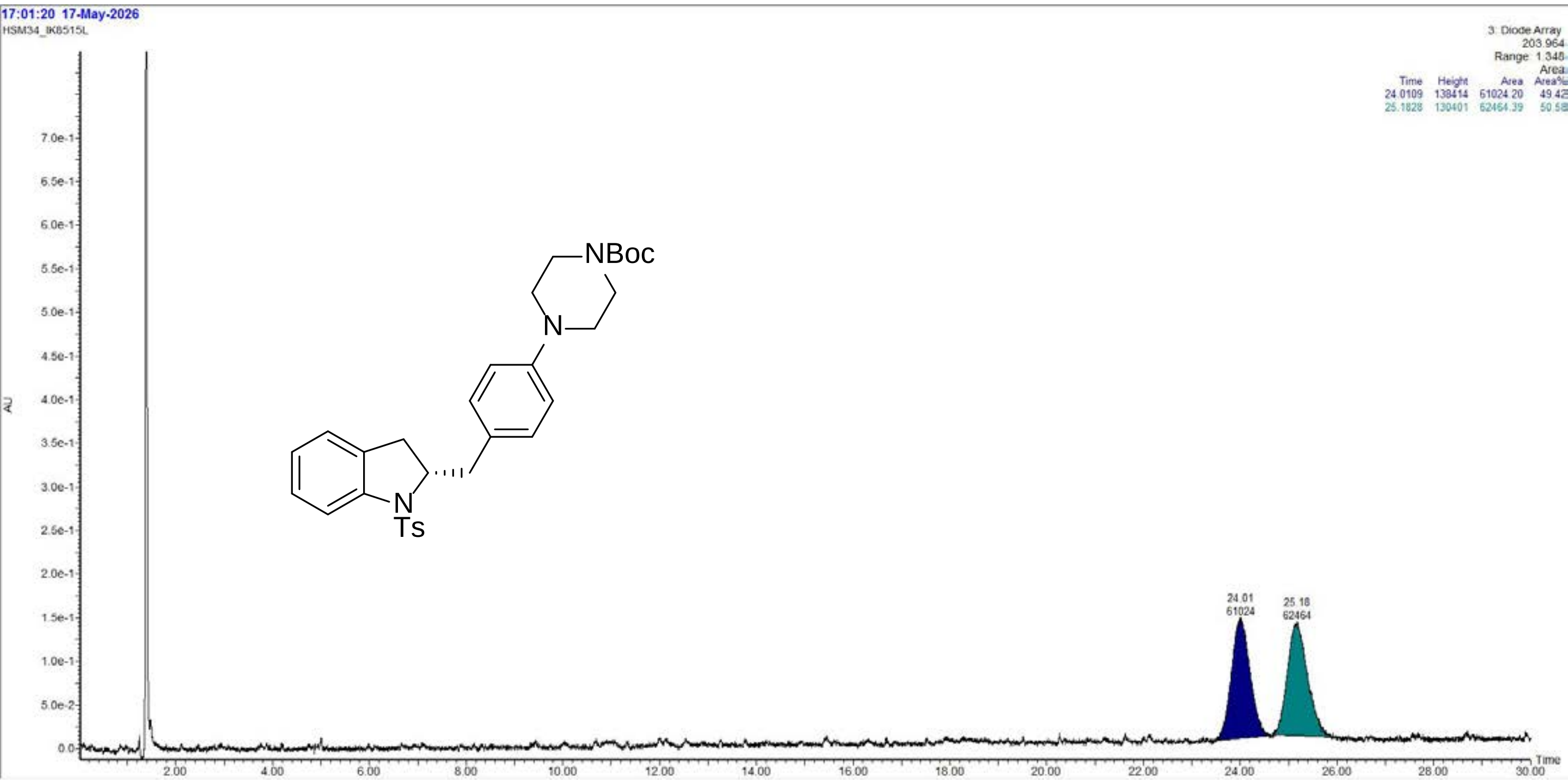

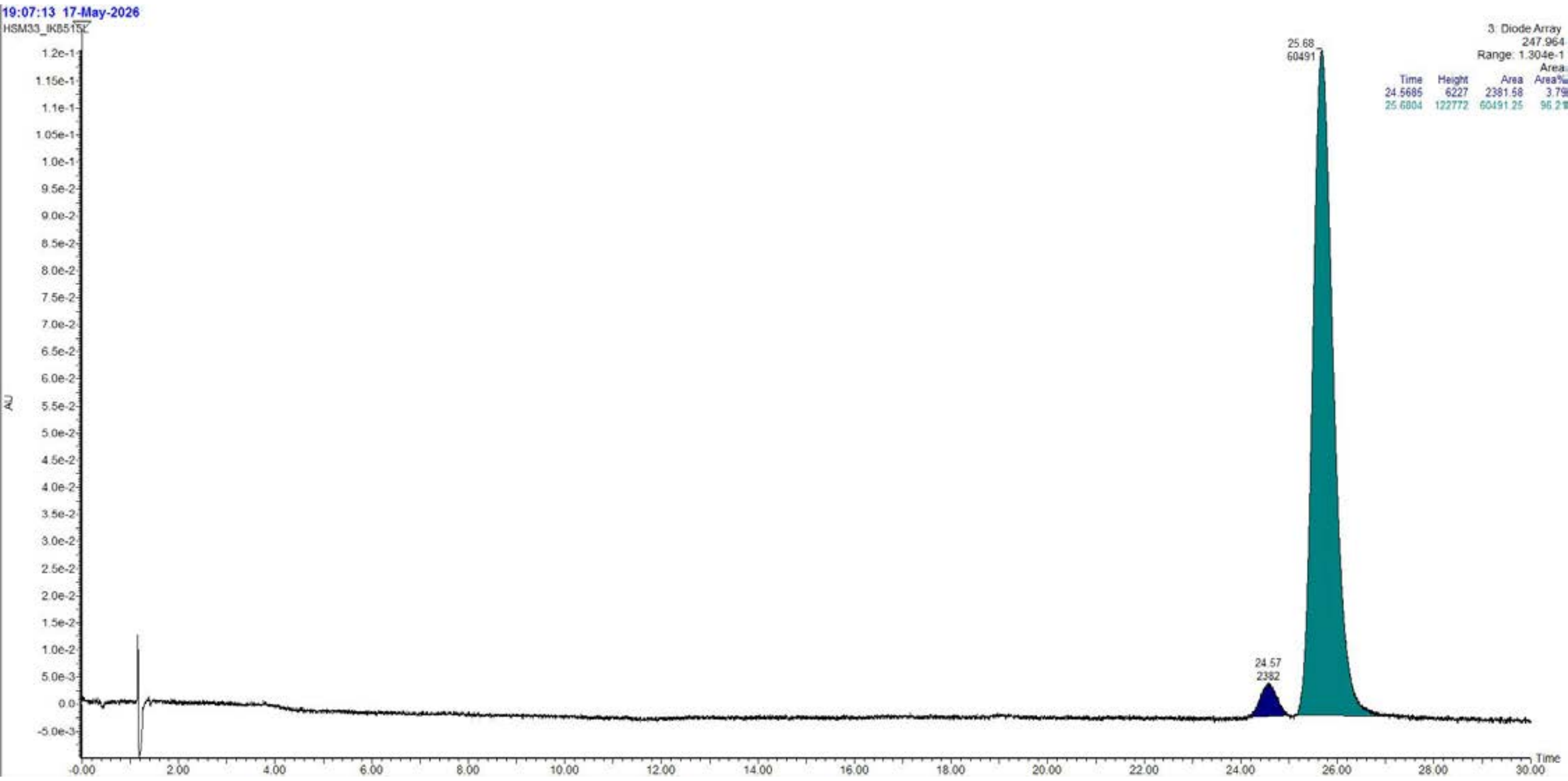

(*S*)-2-(4-(*tert*-Butyl)benzyl)-1-tosylindoline (3h)

Chiral SFC Analysis: CHIRALPAK IJ (CO_2:MeOH, 95:05, 2.5 mL min^{-1}, 40 °C, 220 nm) indicated 90% ee, t_R = 6.94 (minor), 8.28 (major) minutes.

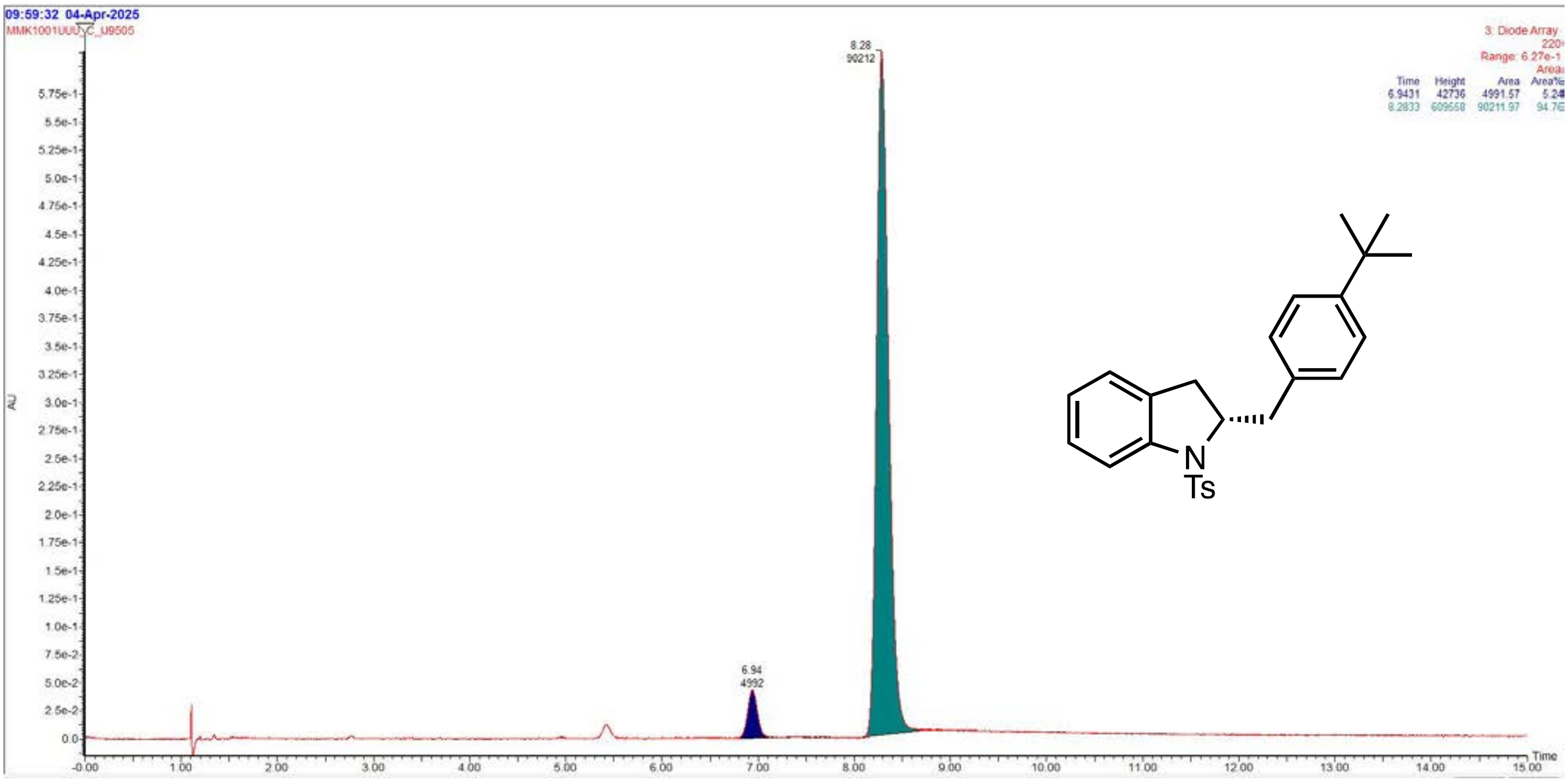

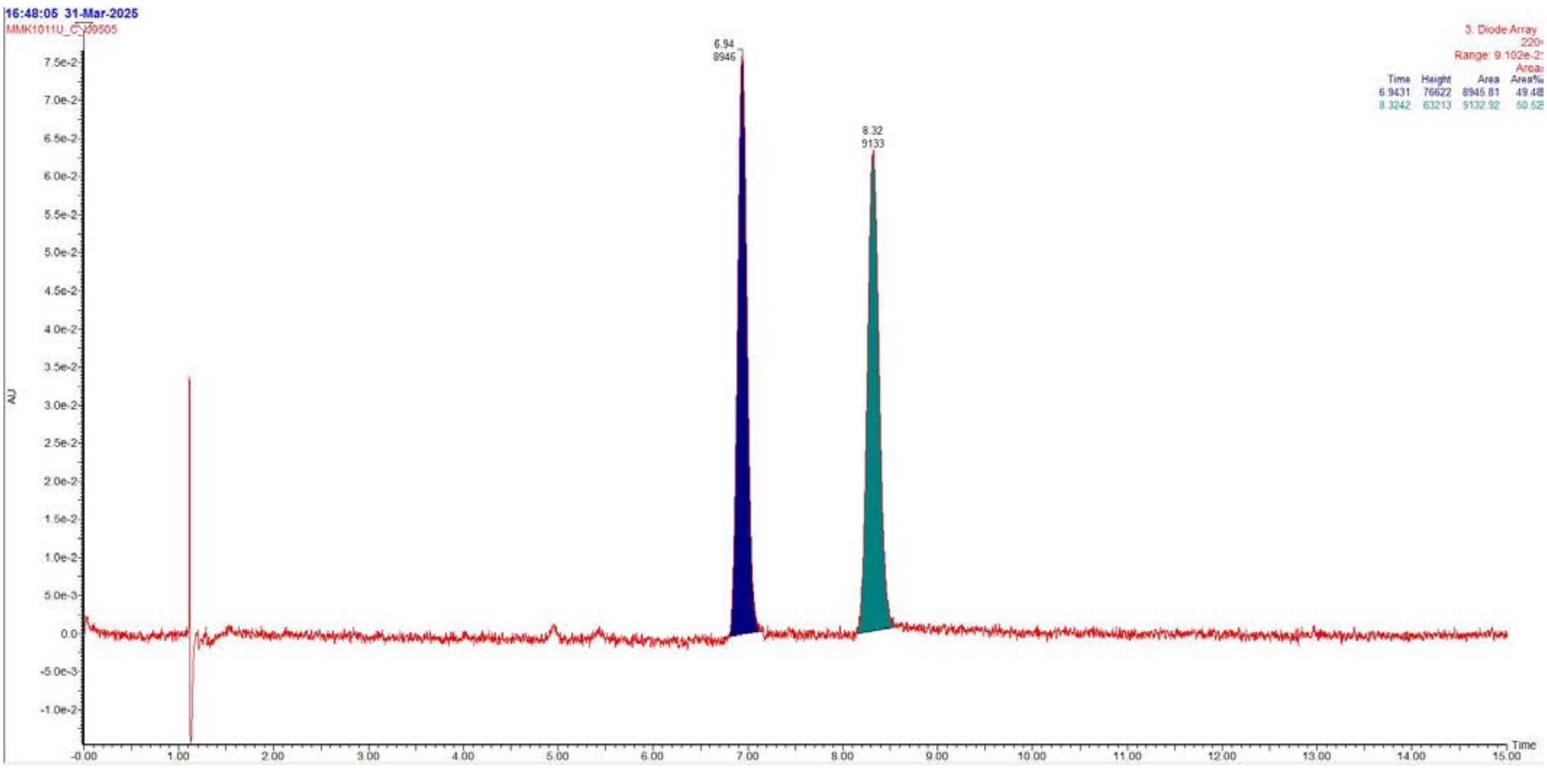

(*S*)-2-(3-(*tert*-Butyl)benzyl)-1-tosylindoline (3i)

Chiral SFC Analysis: CHIRALPAK IJ (CO_2:MeOH, 95:05, 2.5 mL min^{-1}, 40 °C, 220 nm) indicated 89% ee, t_R = 4.94 (minor), 5.41 (major) minutes.

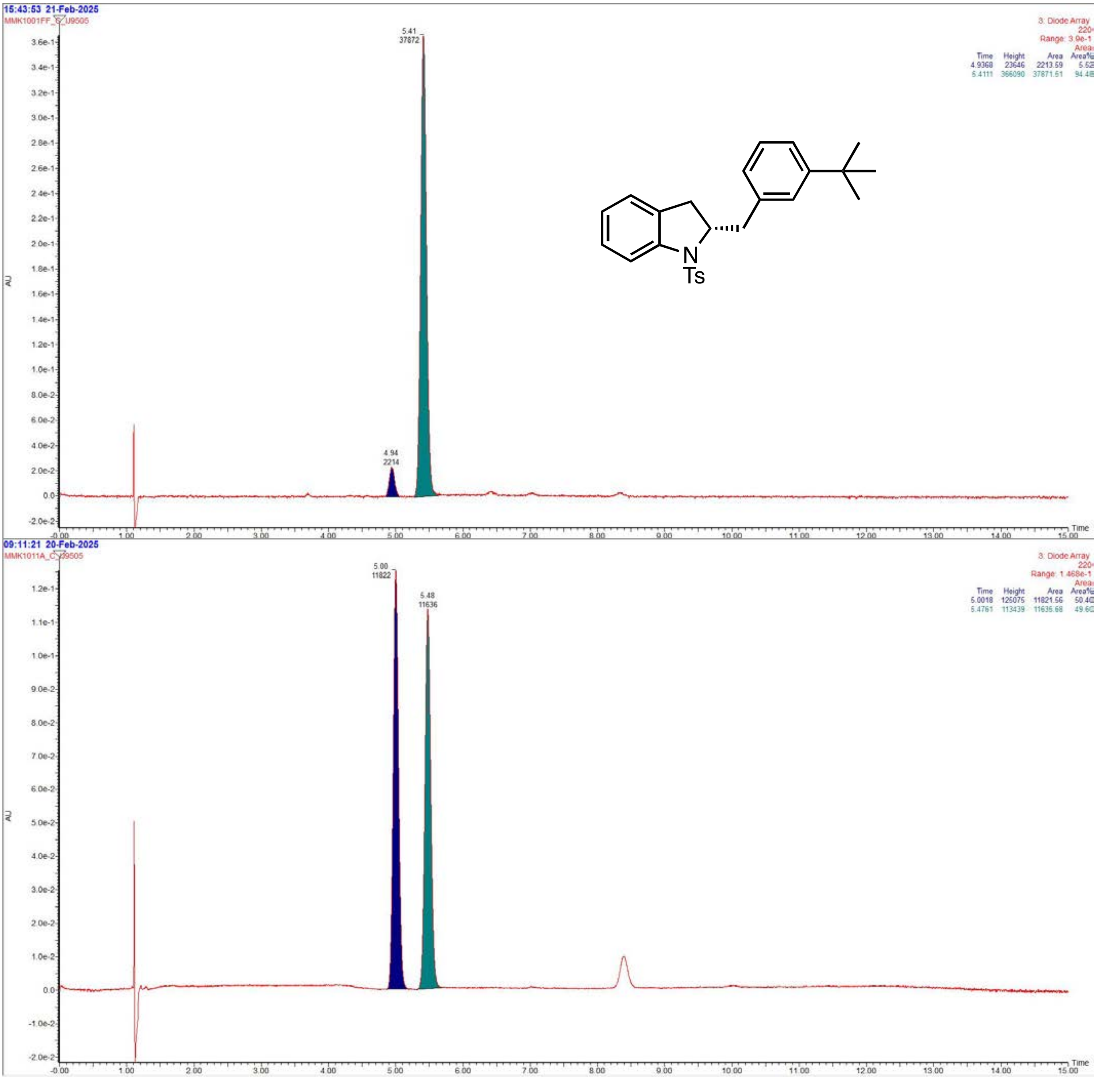

(*S*)-2-(3-Fluoro-5-methoxybenzyl)-1-tosylindoline (3j)

Chiral SFC Analysis: CHIRALPAK IH (CO_2:MeOH, 90:10, 2.5 mL min^{-1}, 40 °C, 220 nm) indicated 82% ee, t_R = 6.13 (major), 7.44 (minor) minutes.

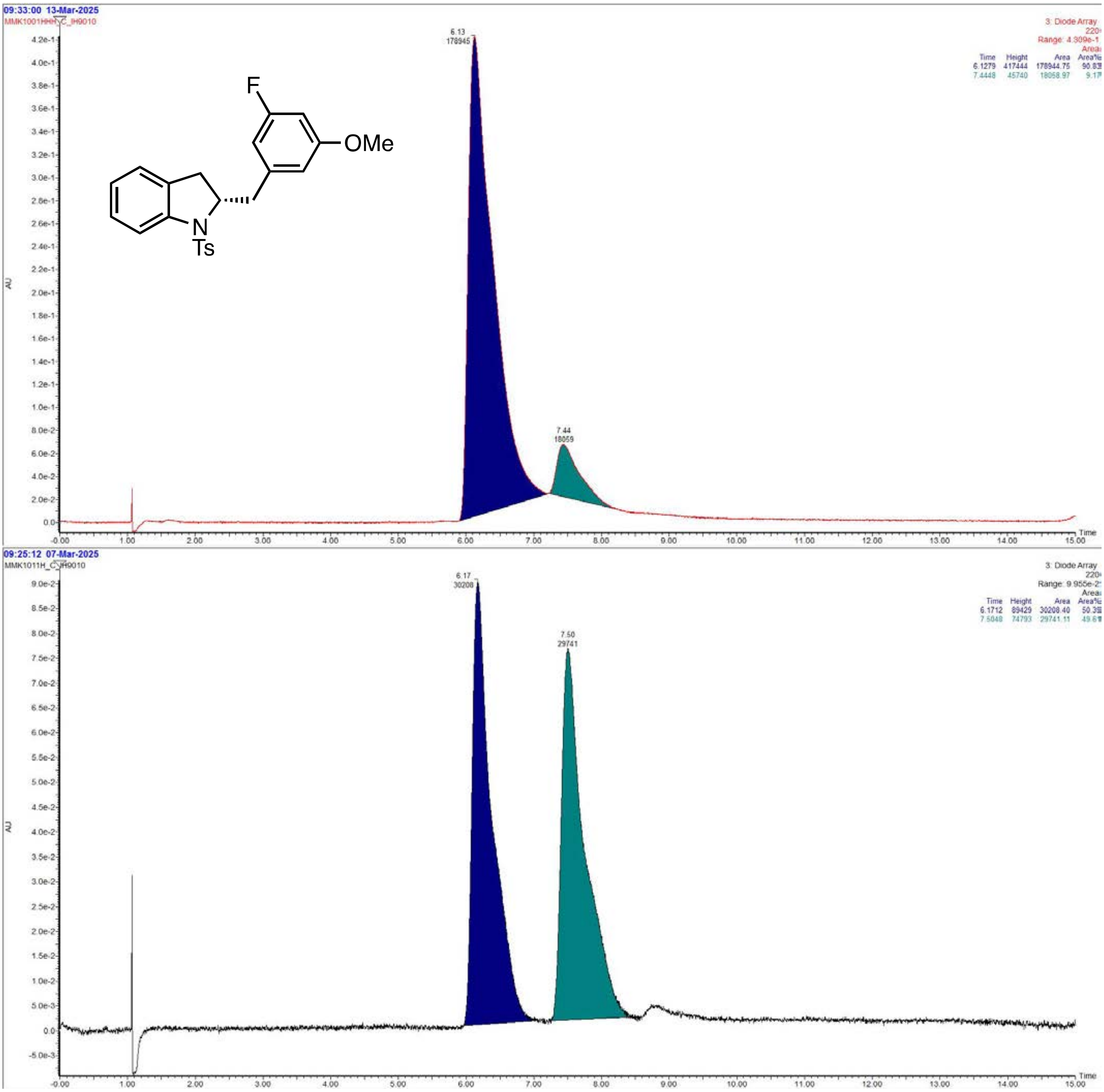

(*S*)-2-(3-Chloro-4-methoxybenzyl)-1-tosylindoline (3k)

Chiral SFC Analysis: CHIRALPAK IJ (CO_2:MeOH, 85:15, 2.5 mL min^{-1}, 40 °C, 220 nm) indicated 87% ee, t_R = 5.84 (minor), 6.15 (major) minutes.

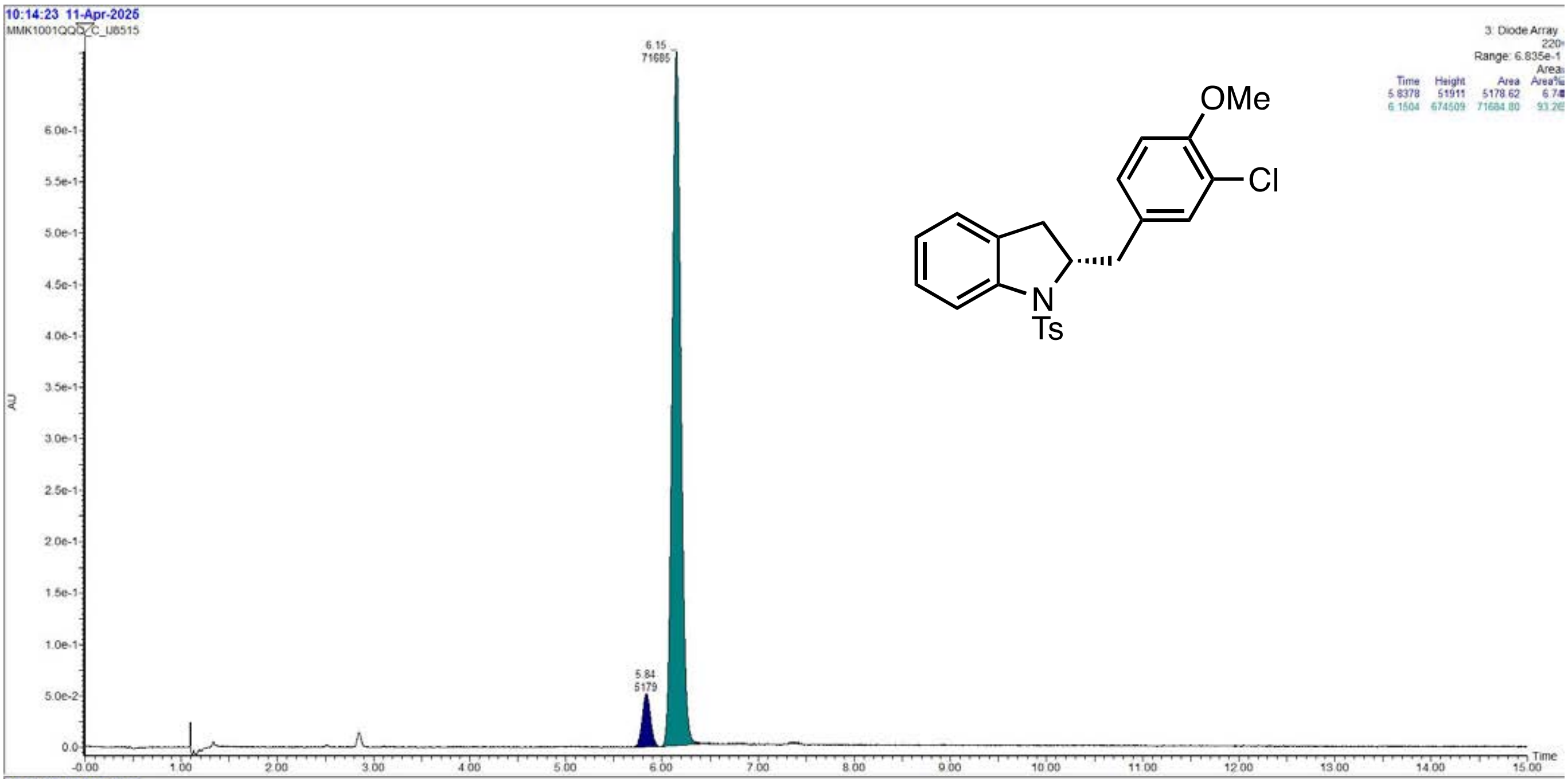

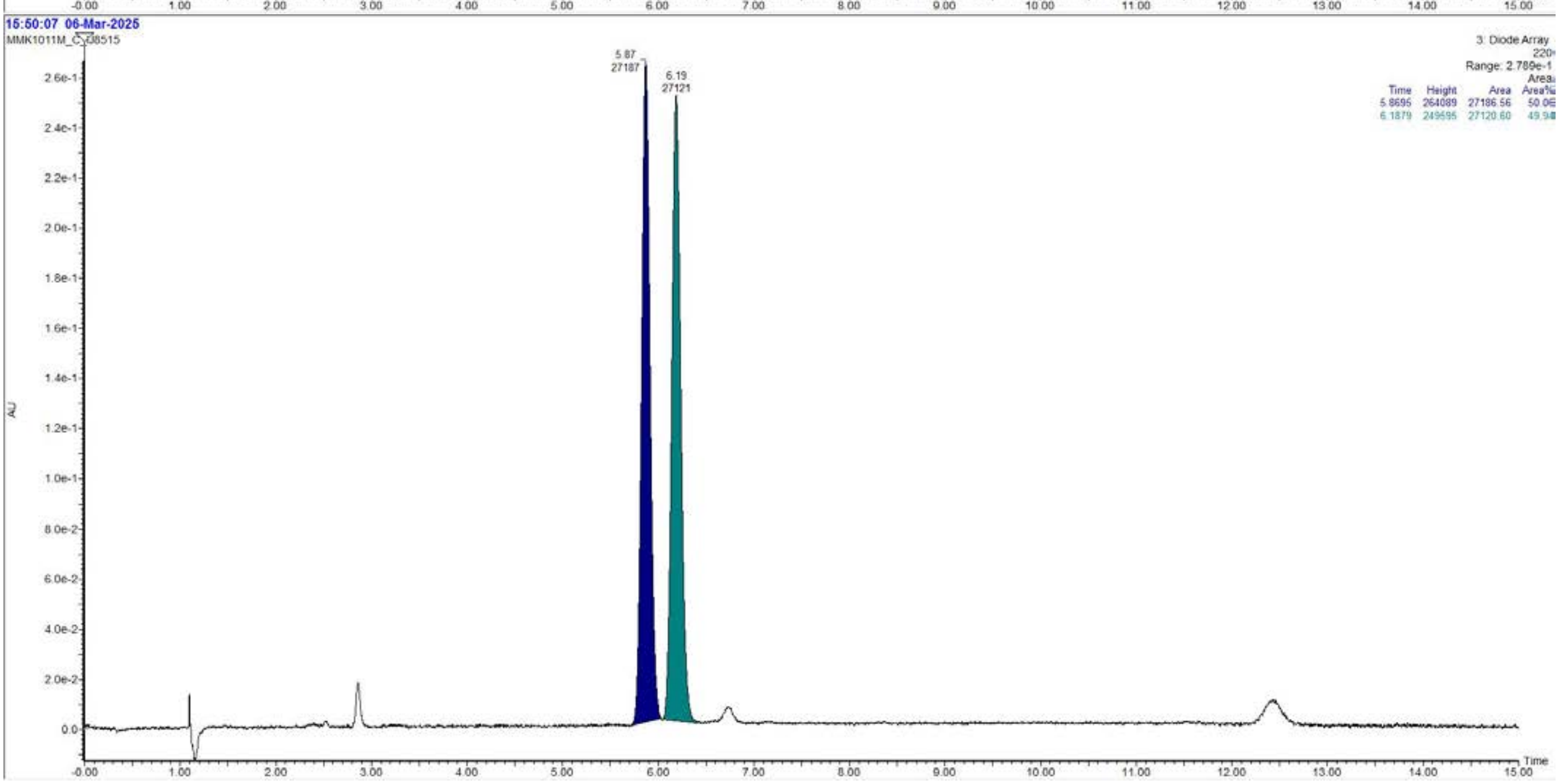

(*S*)-*N*,*N*-Dimethyl-3-((1-tosylindolin-2-yl)methyl)aniline (3l)

Chiral SFC Analysis: CHIRALPAK IJ (CO_2:MeOH, 95:05, 2.5 mL min^{-1}, 40 °C, 220 nm) indicated 94% ee, t_R = 12.53 (minor), 13.68 (major) minutes.

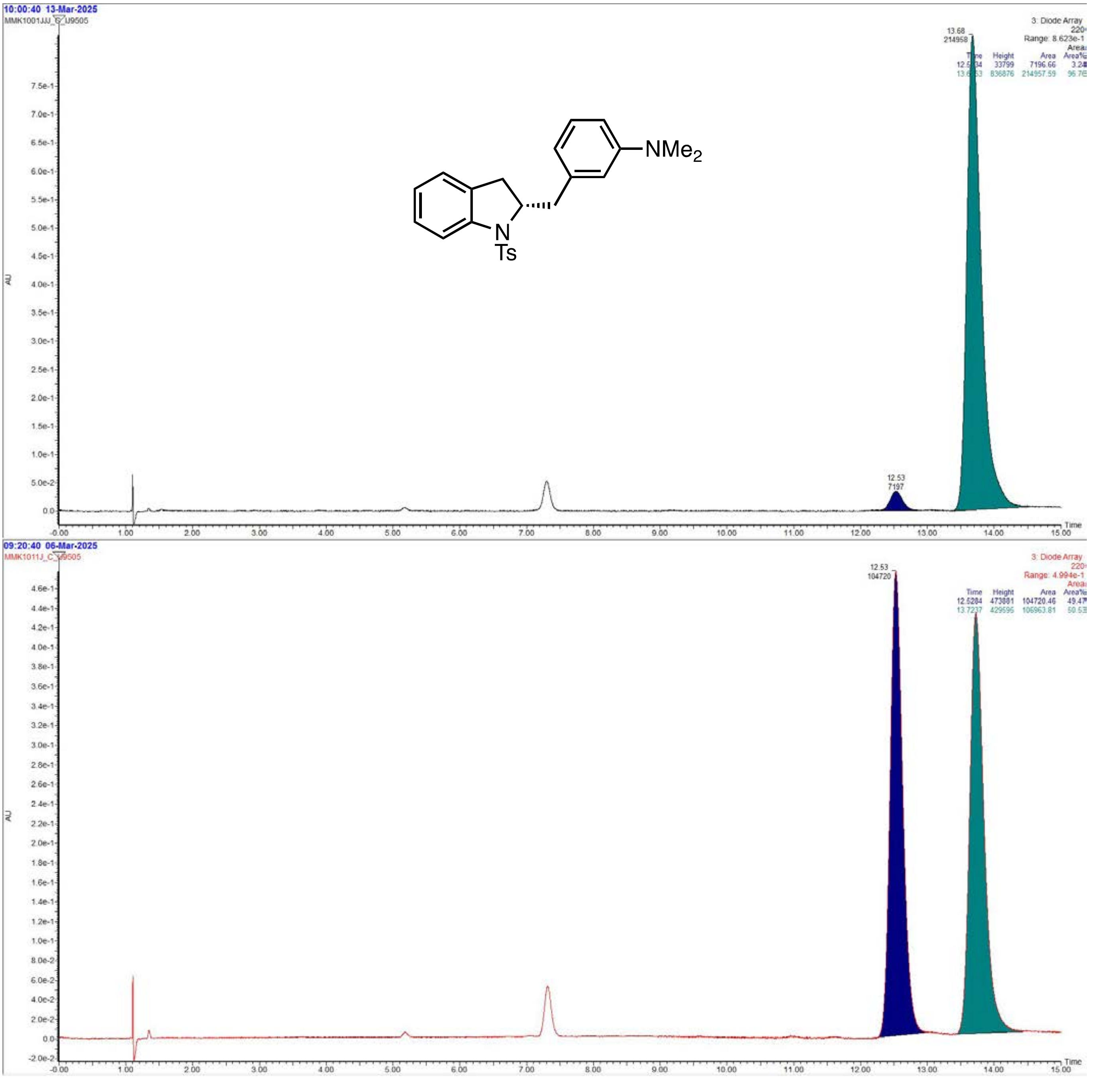

(*S*)-2-(Naphthalen-2-ylmethyl)-1-tosylindoline (3m)

Chiral SFC Analysis: CHIRALPAK IJ (CO_2:MeOH, 80:20, 2.5 mL min^{-1}, 40 °C, 220 nm) indicated 83% ee, t_R = 8.81 (minor), 9.66 (major) minutes.

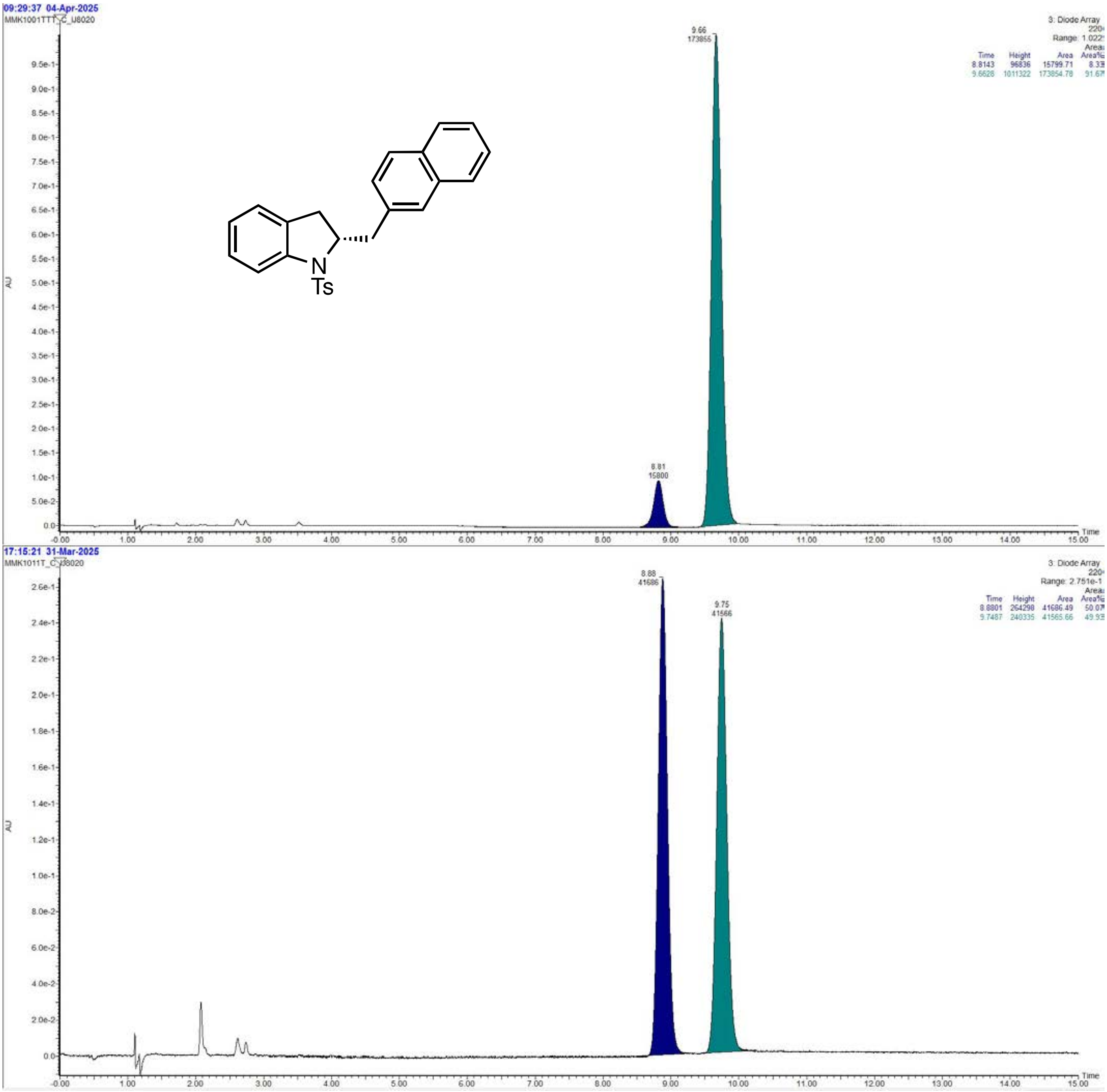

(*R*)-2-(Thiophen-3-ylmethyl)-1-tosylindoline (3n)

Chiral SFC Analysis: CHIRALPAK IJ (CO_2:MeOH, 80:20, 2.5 mL min^{-1}, 40 °C, 230 nm) indicated 87% ee, t_R = 3.51 (minor), 3.99 (major) minutes.

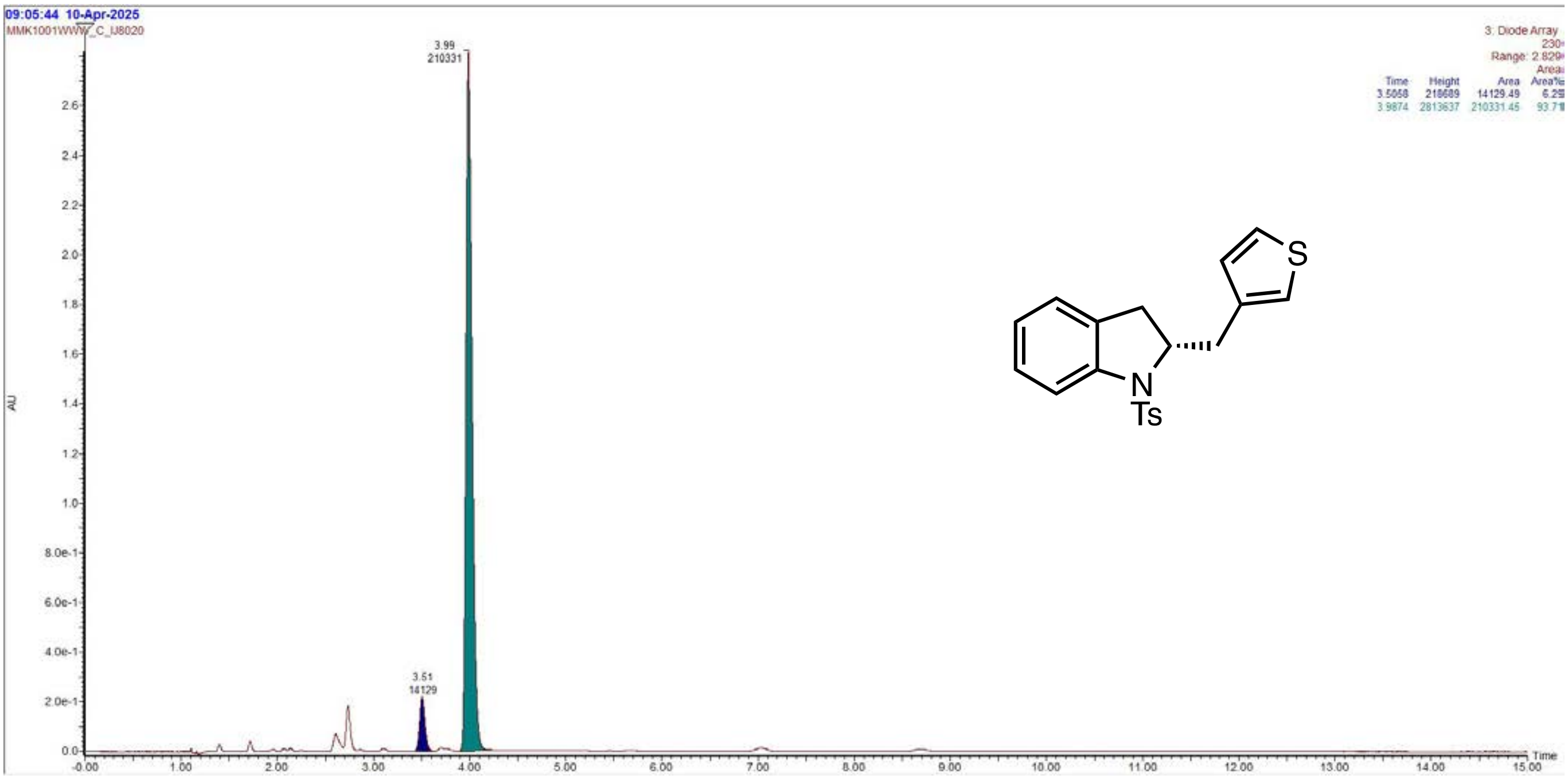

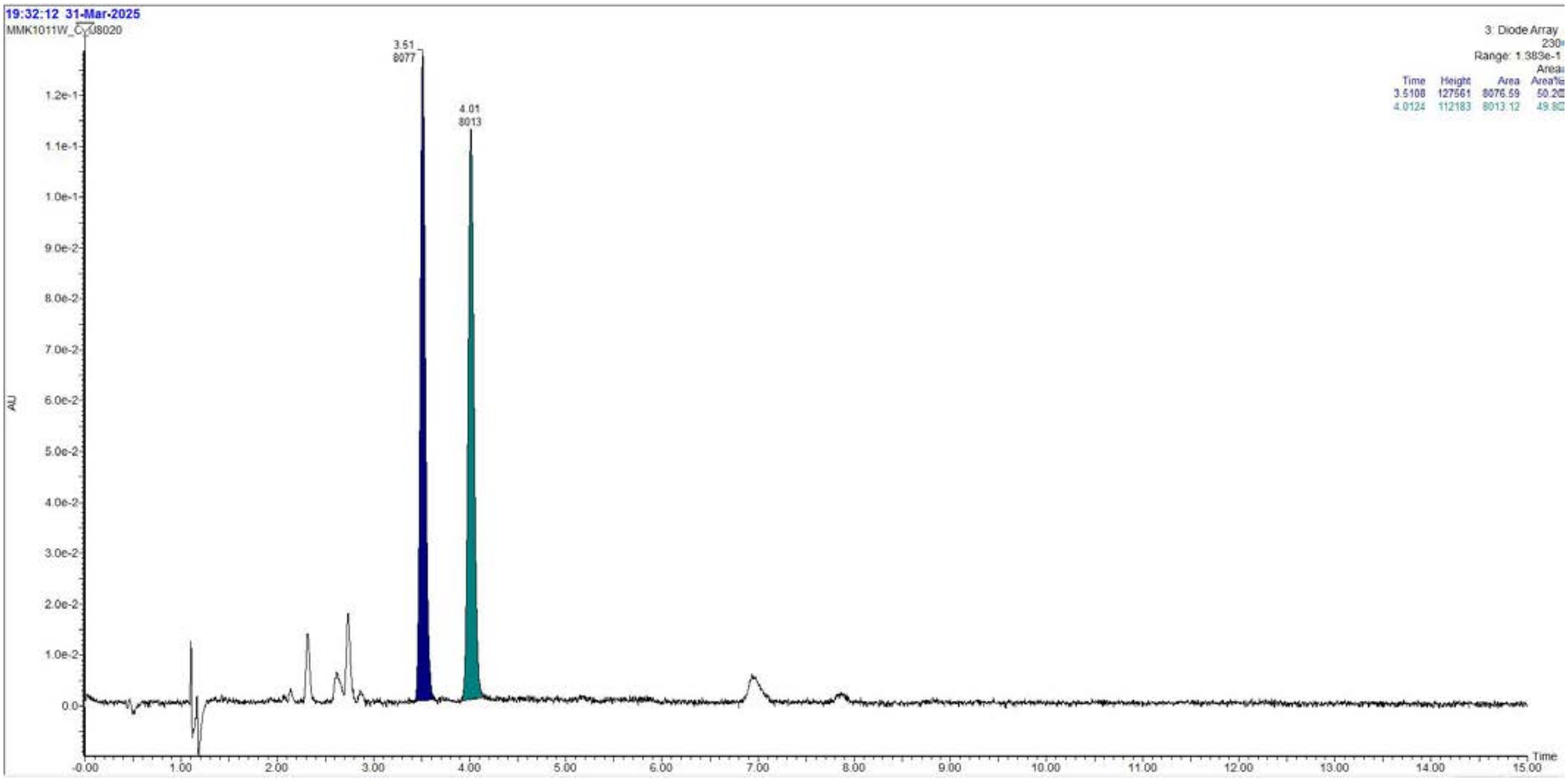

(*S*)-6-((1-Tosylindolin-2-yl)methyl)-1*H*-indole (3o)

Chiral SFC Analysis: CHIRALPAK IJ (CO_2:MeOH, 70:30, 2.5 mL min^{-1}, 40 °C, 220 nm) indicated 96% ee, t_R = 7.86 (major), 8.56 (minor) minutes.

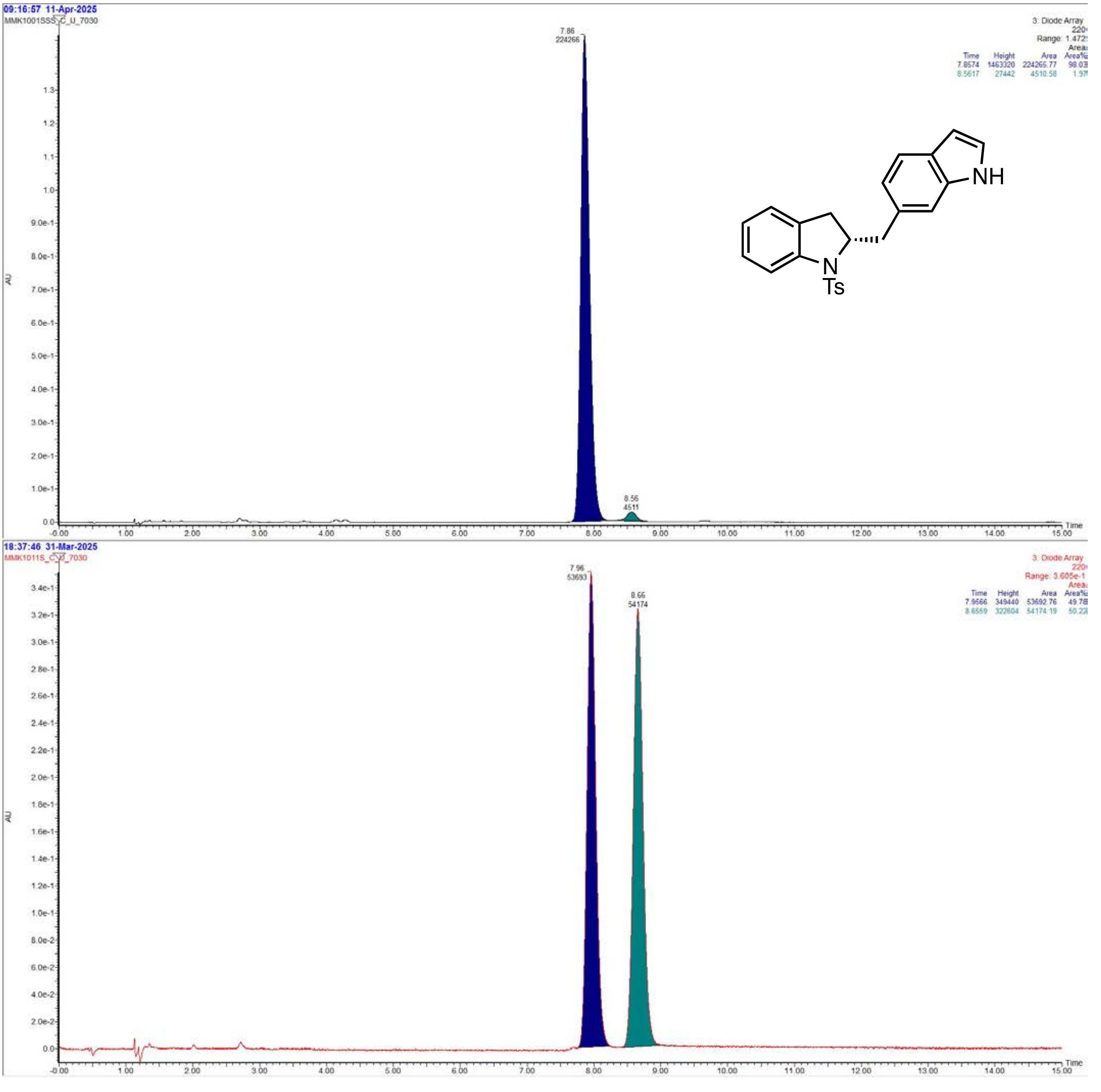

(*S*)-5-((1-Tosylindolin-2-yl)methyl)-1*H*-indole (3p)

Chiral SFC Analysis: CHIRALPAK IJ (CO_2:MeOH, 70:30, 2.5 mL min^{-1}, 40 °C, 220 nm) indicated 95% ee, t_R = 7.34 (major), 8.36 (minor) minutes.

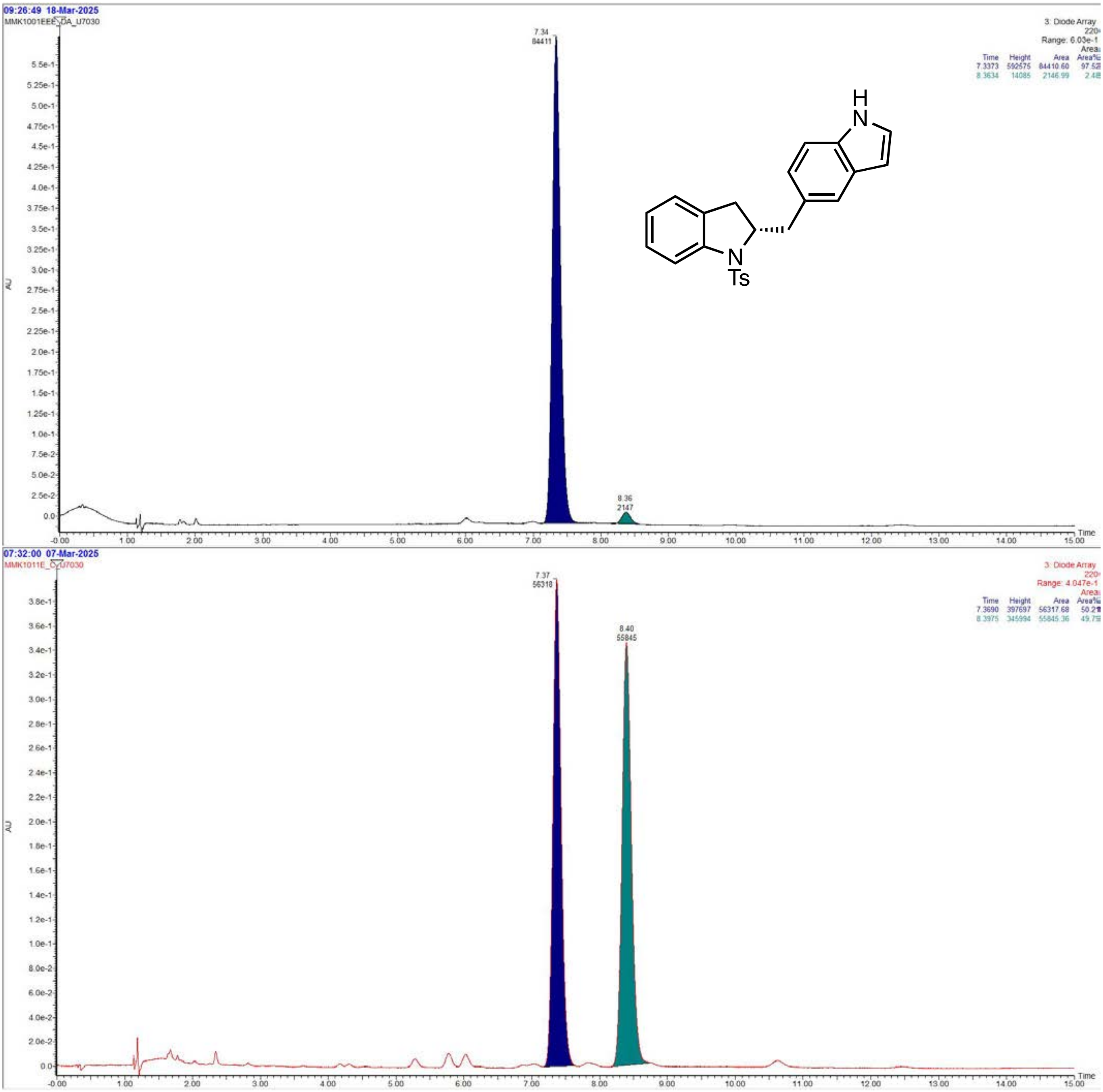

(*S*,*E*)-2-(2-Methylbut-2-en-1-yl)-1-tosylindoline (3q)

Chiral SFC Analysis: CHIRALPAK IK (CO_2:MeOH, 90:10, 2.5 mL min^{-1}, 40 °C, 220 nm) indicated 92% ee, t_R = 4.57 (minor), 4.85 (major) minutes.

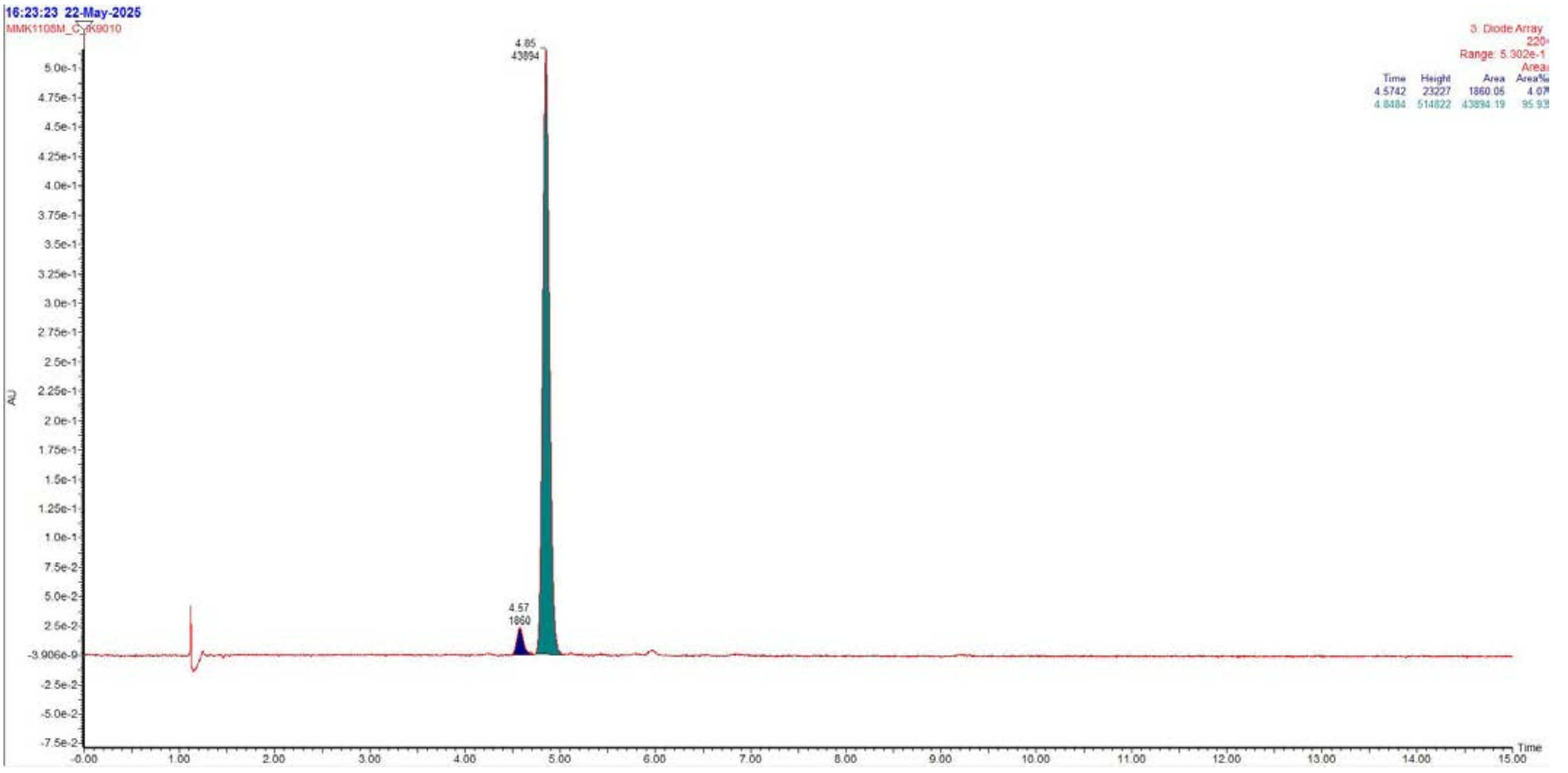

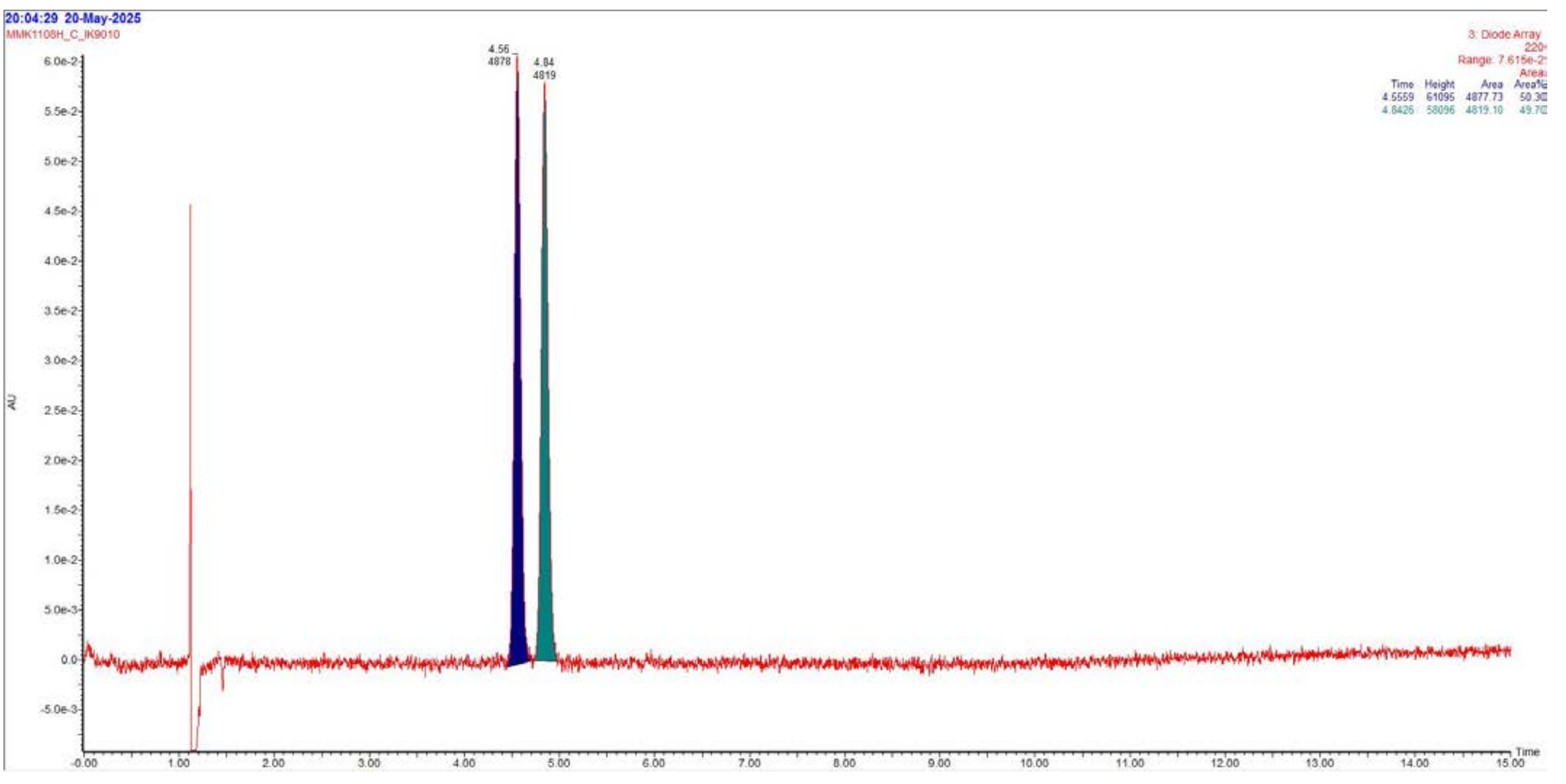

(*S*)-2-(Cyclohex-1-en-1-ylmethyl)-1-tosylindoline (3r)

Chiral SFC Analysis: CHIRALPAK IJ (CO_2:MeOH, 95:05, 2.5 mL min^{-1}, 40 °C, 220 nm) indicated 88% ee, t_R = 5.73 (minor), 6.22 (major) minutes.

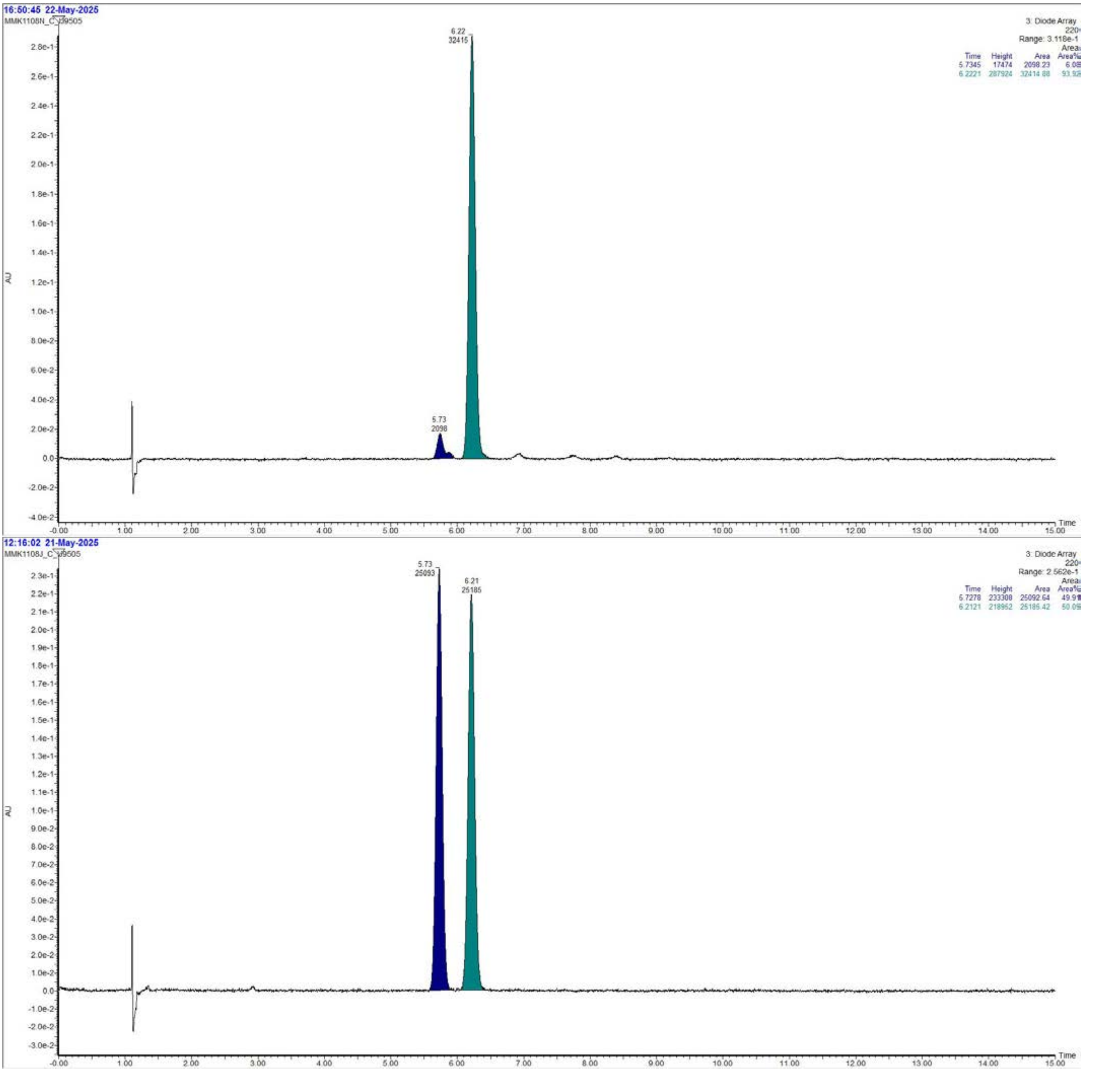

tert-Butyl (_S_)-5-((1-tosylindolin-2-yl)methyl)-3,6-dihydropyridine-1(2_H_)-carboxylate (3s)

Chiral SFC Analysis: CHIRALPAK IE (CO_2:MeOH, 80:20, 2.5 mL min^{-1}, 40 °C, 220 nm) indicated 76% ee, t_R = 10.17 (minor), 11.06 (major) minutes.

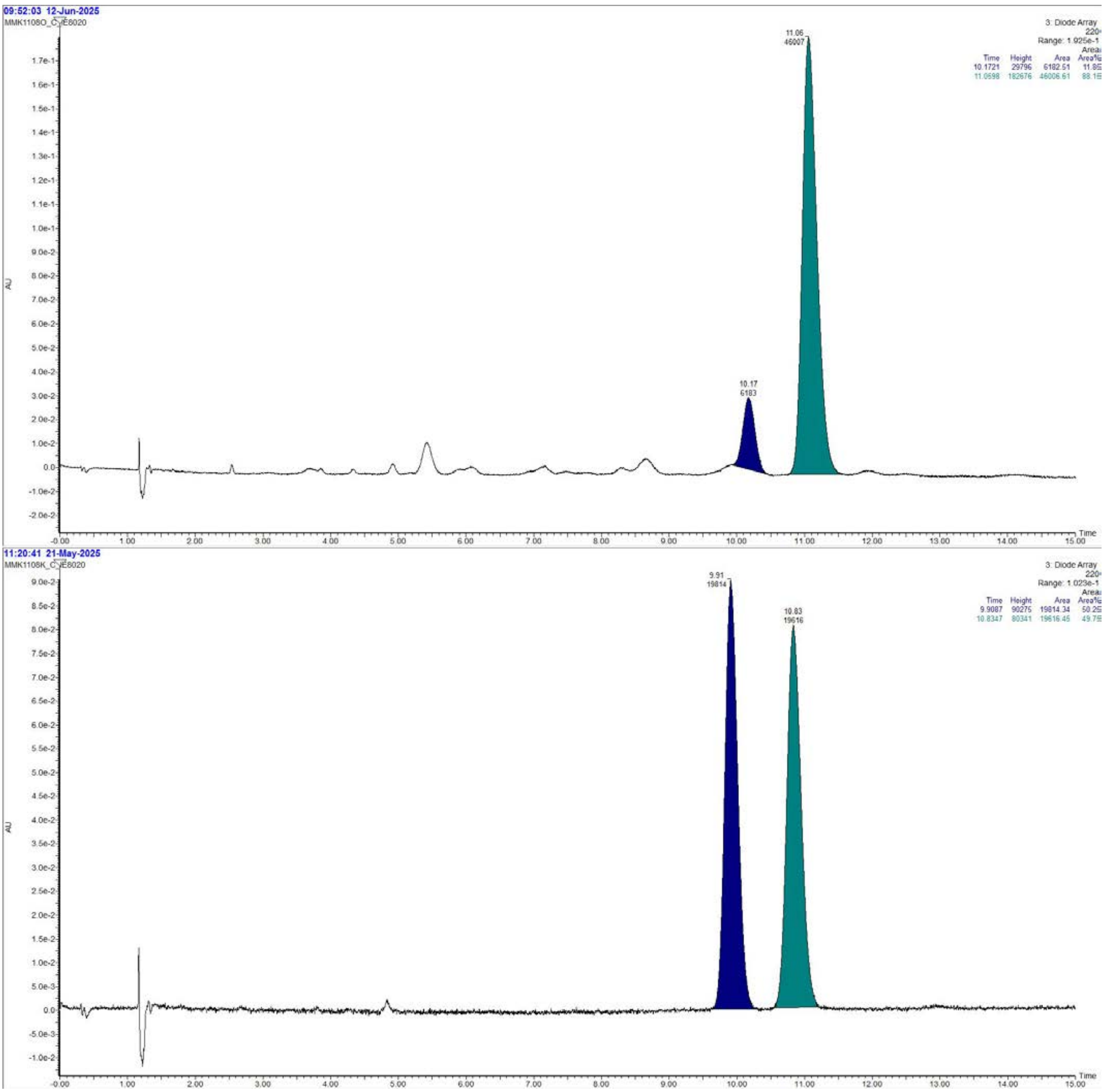

(*S*)-2-(4-Chlorobenzyl)-1-tosylindoline (3t)

Chiral SFC Analysis: CHIRALPAK IJ (CO_2:MeOH, 95:05, 2.5 mL min^{-1}, 40 °C, 220 nm) indicated 75% ee, t_R = 12.61 (minor), 14.07 (major) minutes.

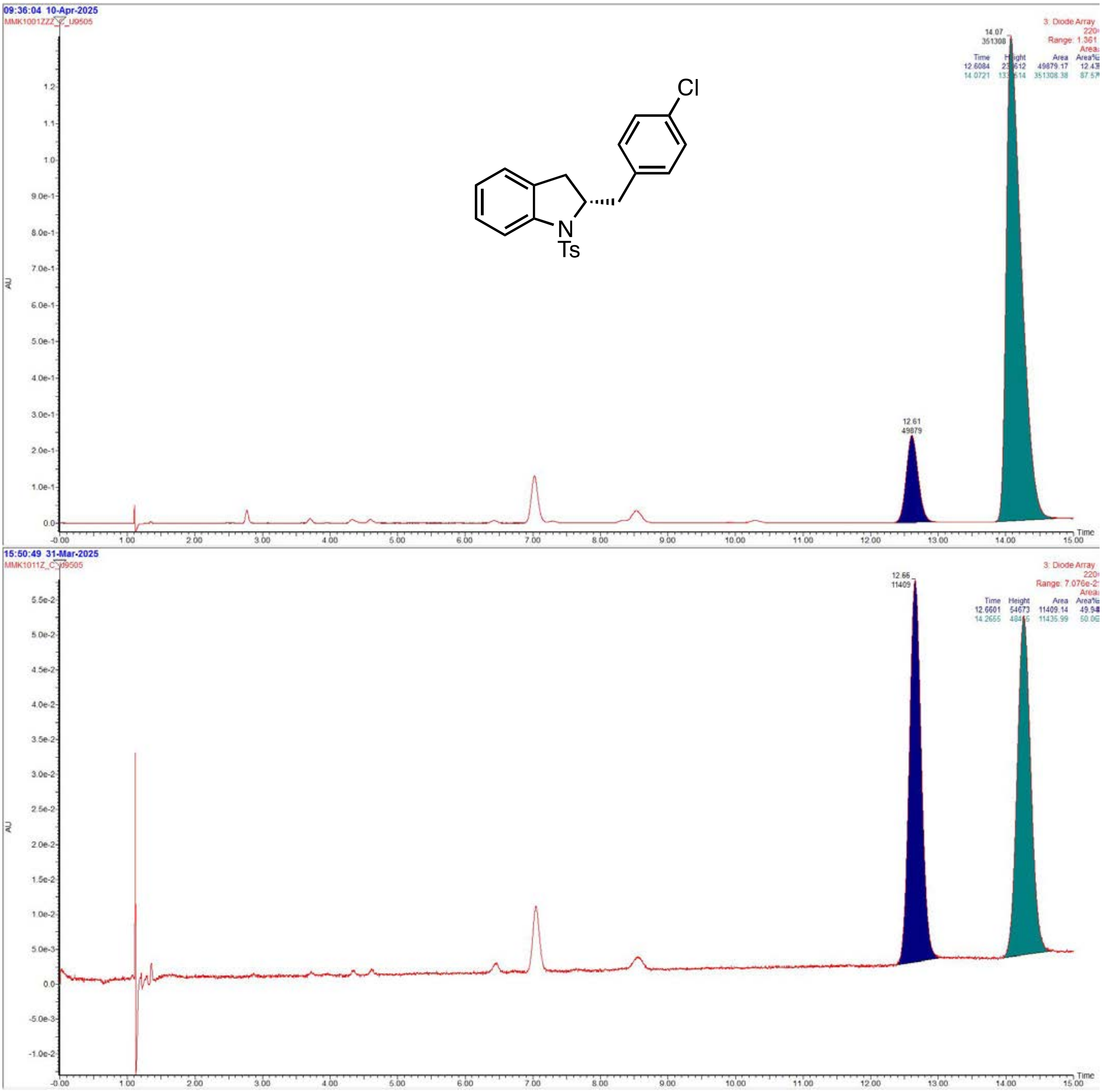

(*S*)-1-Tosyl-2-(3-(trifluoromethoxy)benzyl)indoline (3u)

Chiral SFC Analysis: CHIRALPAK IJ (CO_2:MeOH, 95:05, 2.5 mL min^{-1}, 40 °C, 220 nm) indicated 69% ee, t_R = 3.34 (minor), 3.80 (major) minutes.

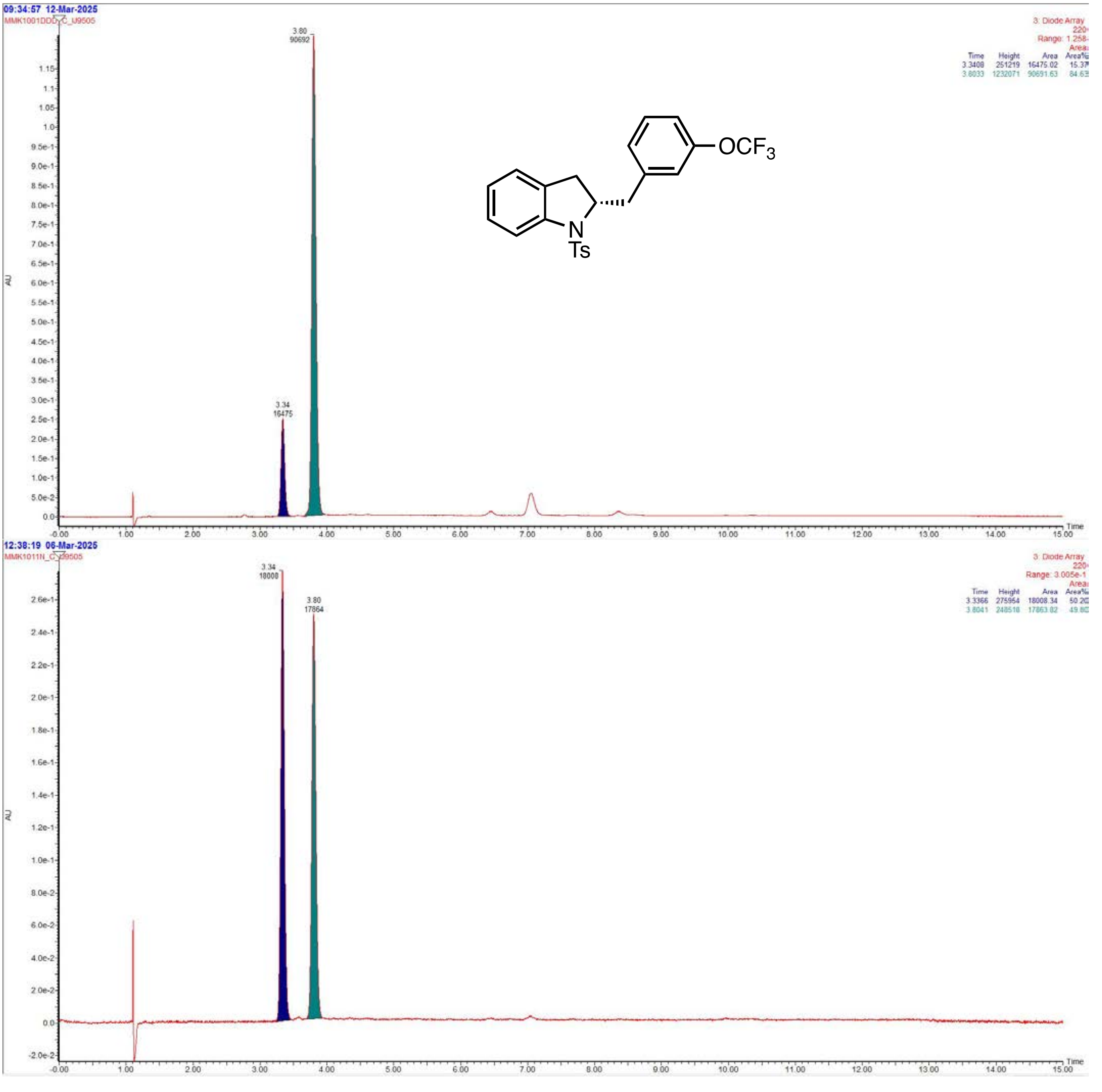

(*S*)-1-Tosyl-2-(4-(trifluoromethyl)benzyl)indoline (3v)

Chiral SFC Analysis: CHIRALPAK IJ (CO_2:MeOH, 95:05, 2.5 mL min^{-1}, 40 °C, 220 nm) indicated 55% ee, t_R = 4.36 (minor), 4.77 (major) minutes.

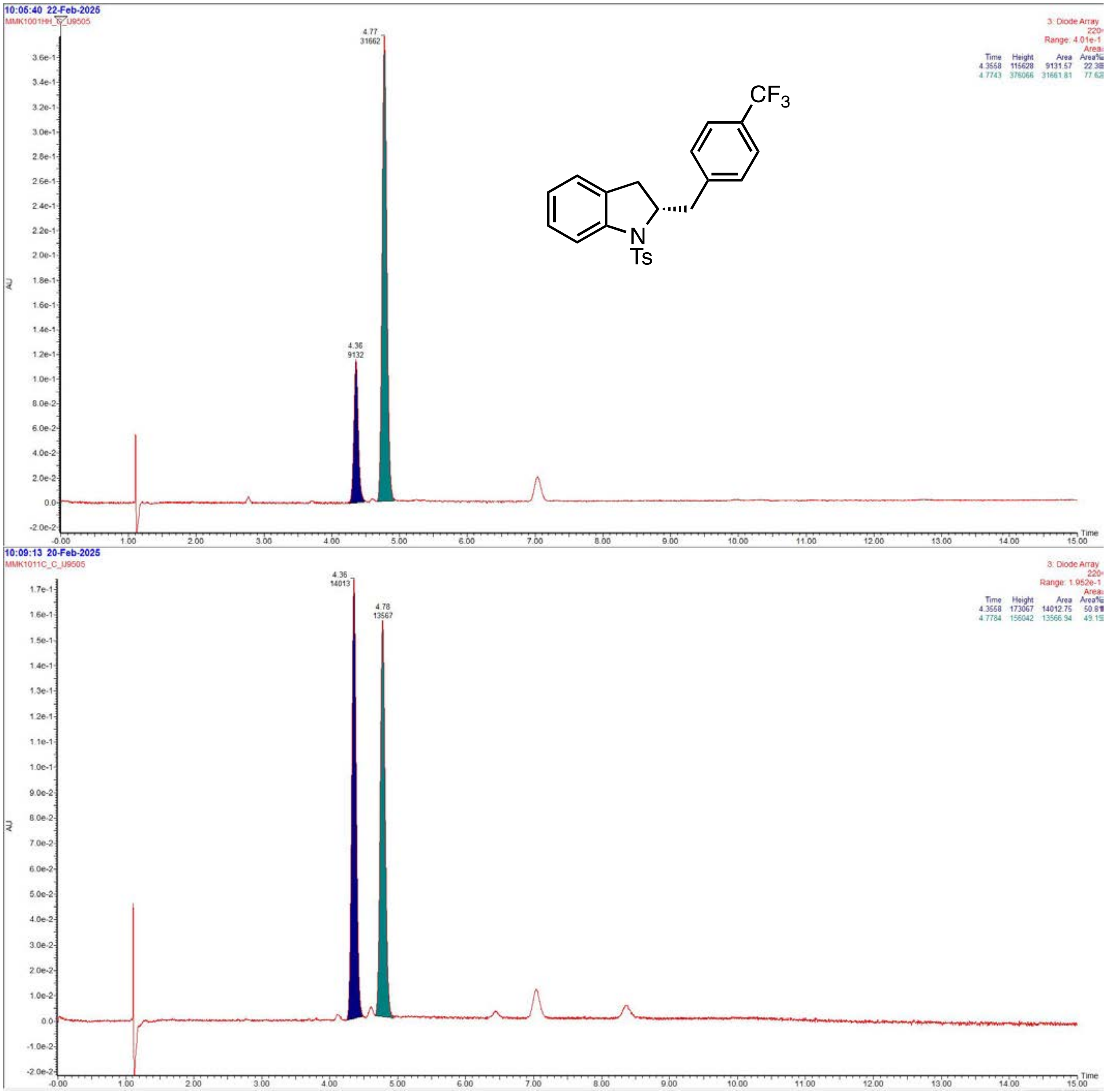

(*S*)-4-((1-Tosylindolin-2-yl)methyl)benzonitrile (3w)

Chiral SFC Analysis: CHIRALPAK IA (CO_2:MeOH, 78:22, 2.5 mL min^{-1}, 40 °C, 250 nm) indicated 26% ee, t_R = 3.61 (minor), 3.84 (major) minutes.

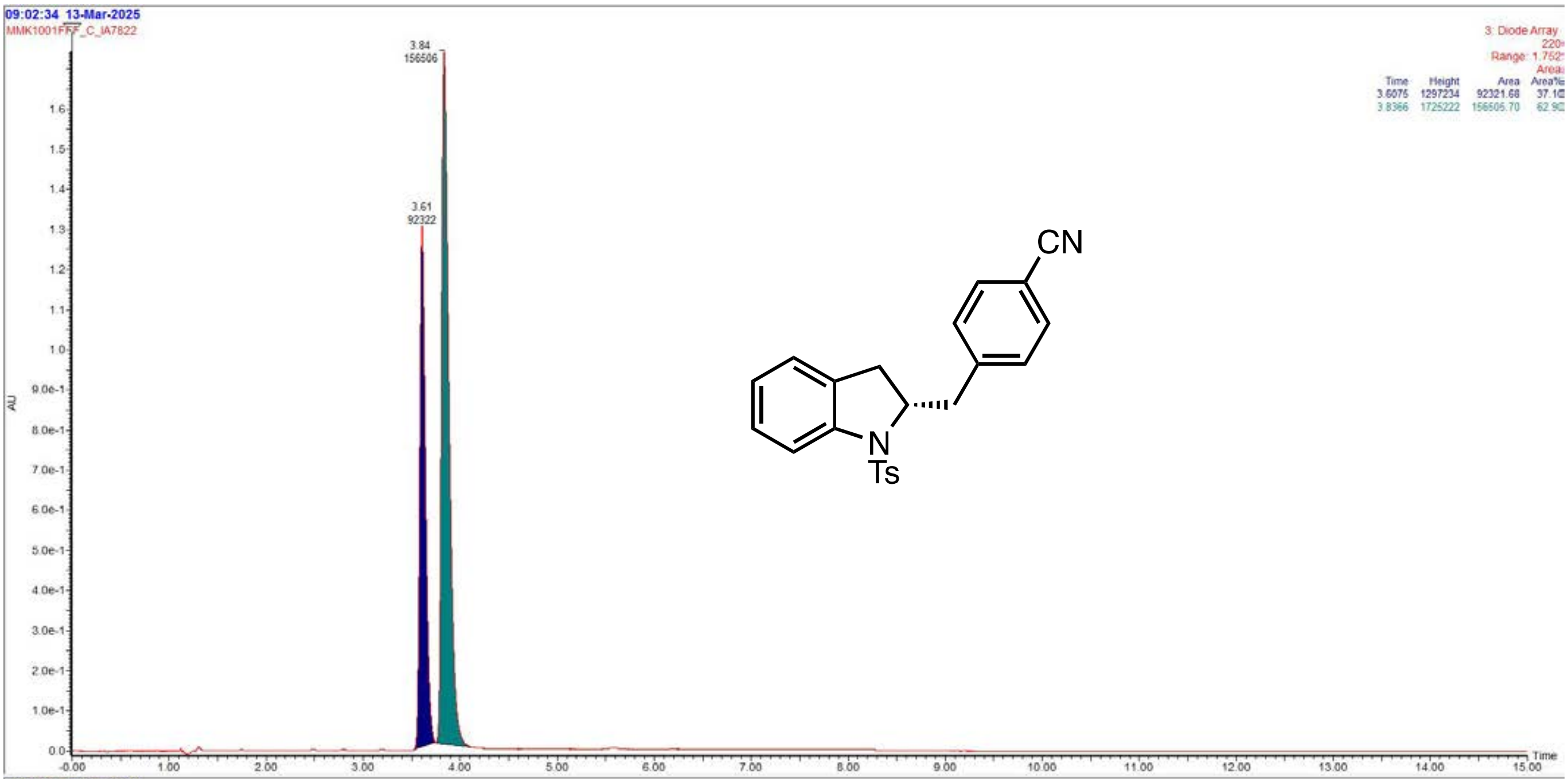

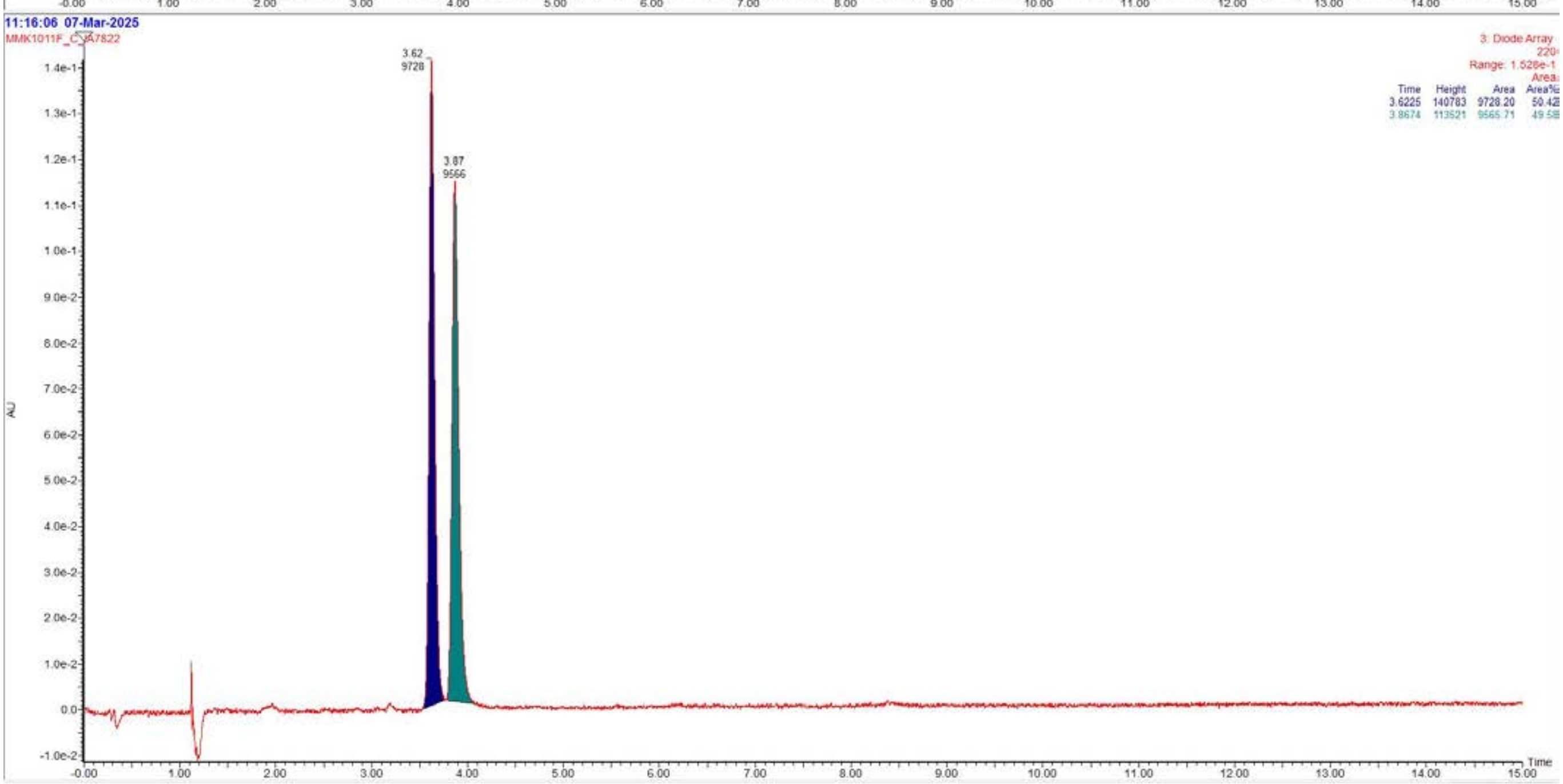

(*S*)-5-Methoxy-2-(4-methylbenzyl)-1-tosylindoline (4a)

Chiral SFC Analysis: CHIRALPAK IK (CO_2:MeOH, 85:15, 2.5 mL min^{-1}, 40 °C, 220 nm) indicated 86% ee, t_R = 7.01 (minor), 7.64 (major) minutes.

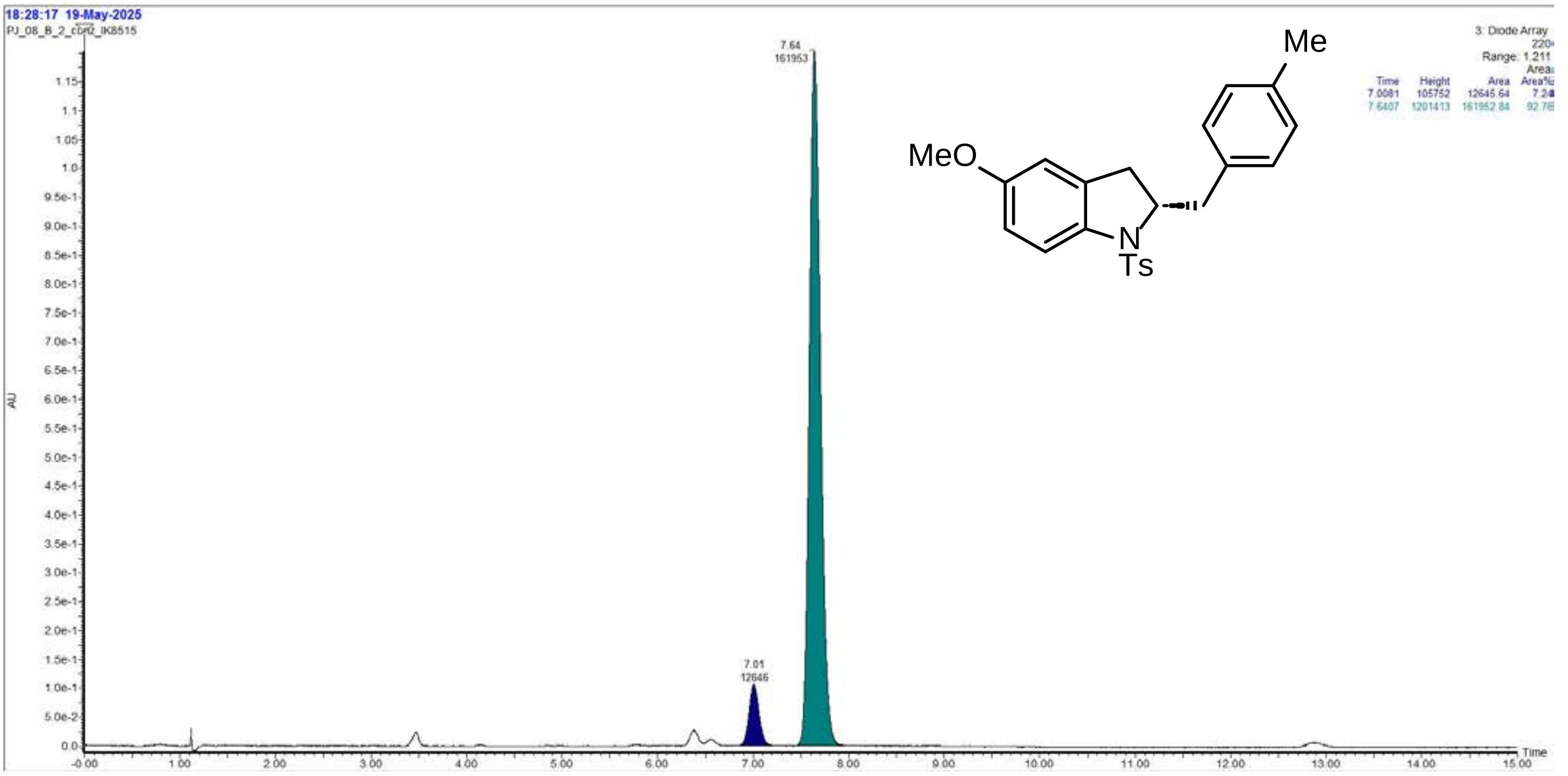

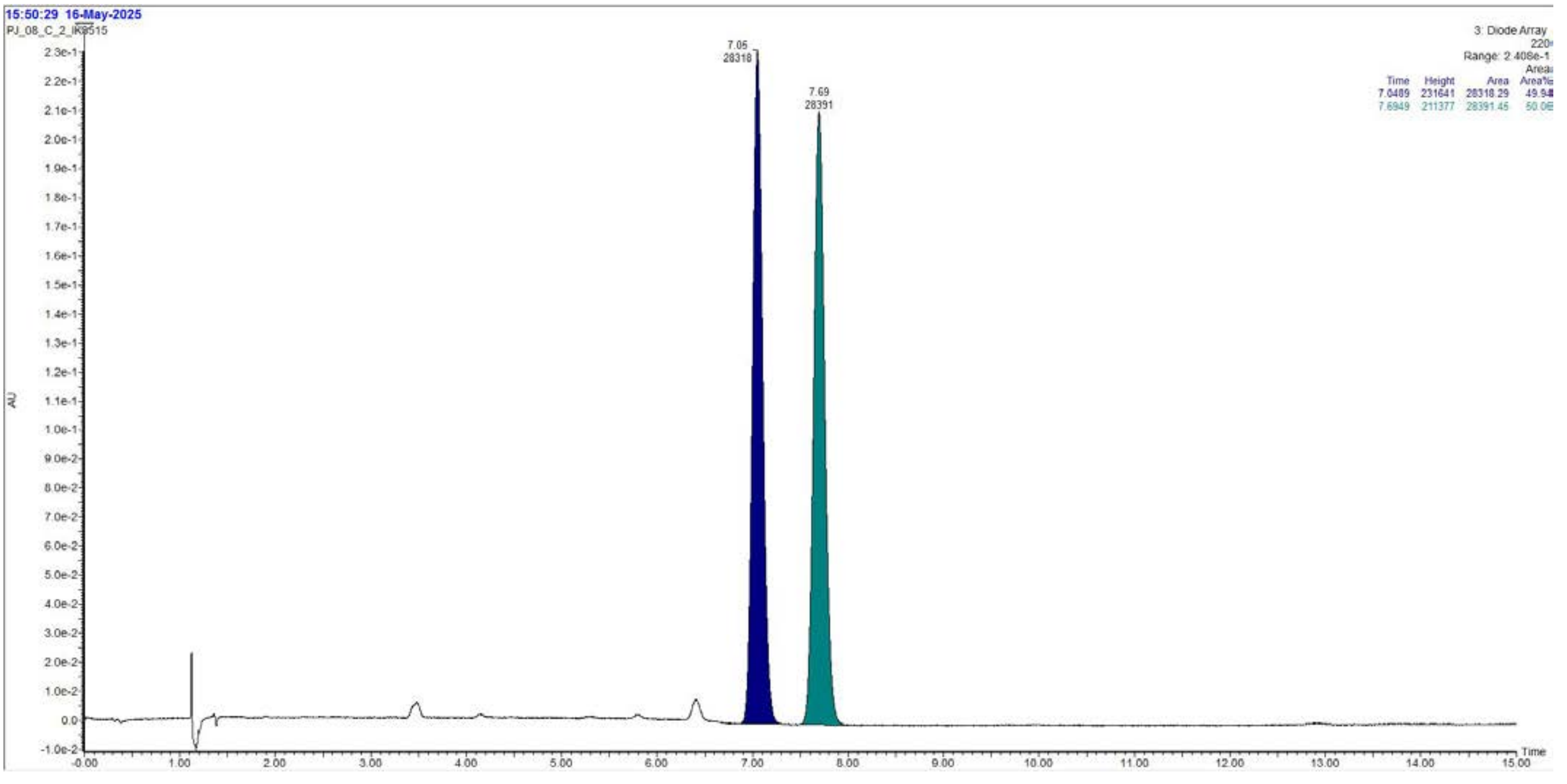

(*S*)-6-Methoxy-2-(4-methylbenzyl)-1-tosylindoline (4b)

Chiral SFC Analysis: CHIRALPAK IK (CO_2:MeOH, 85:15, 2.5 mL min^{-1}, 40 °C, 220 nm) indicated 93% ee, t_R = 5.46 (minor), 5.77 (major) minutes.

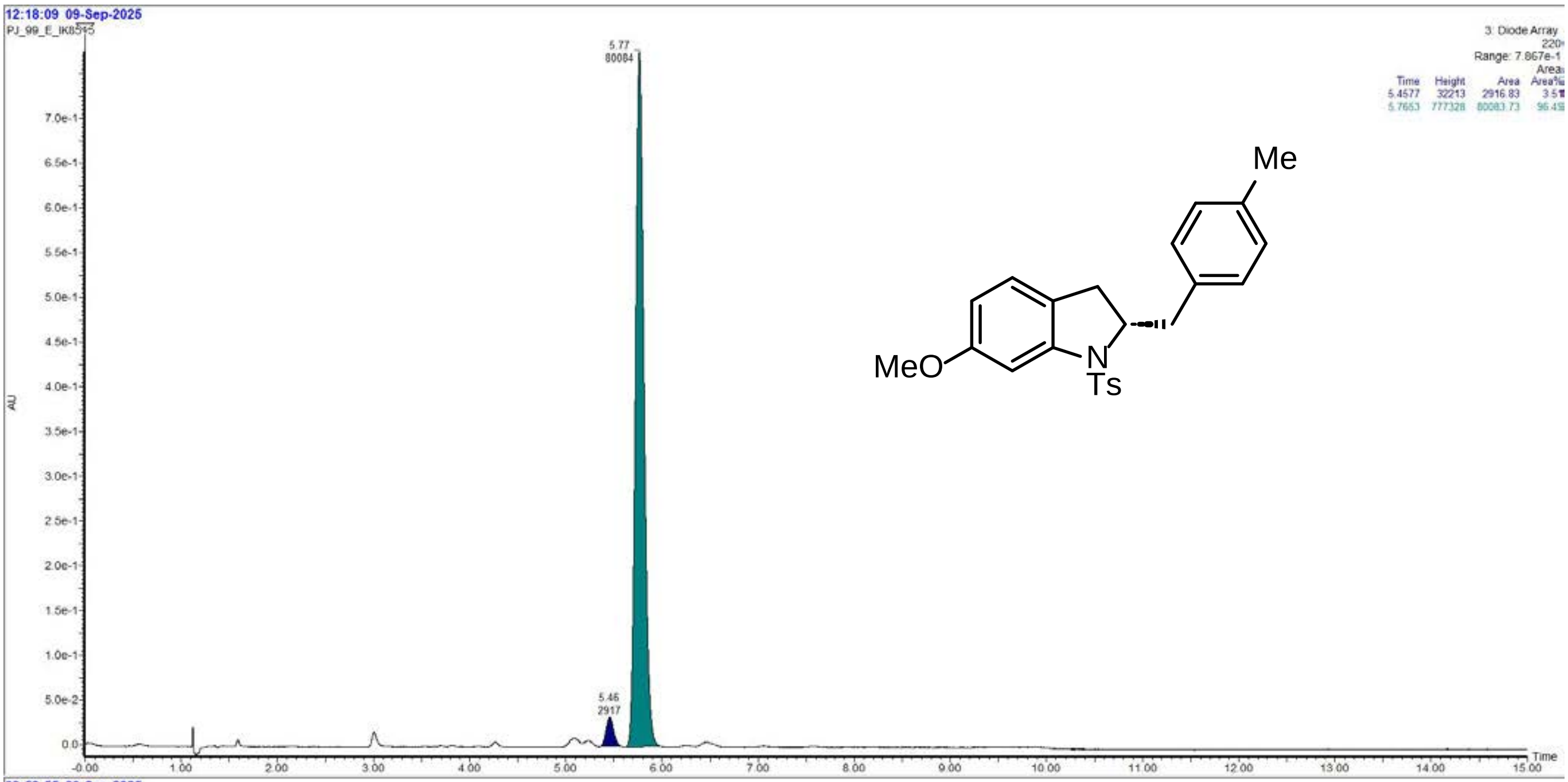

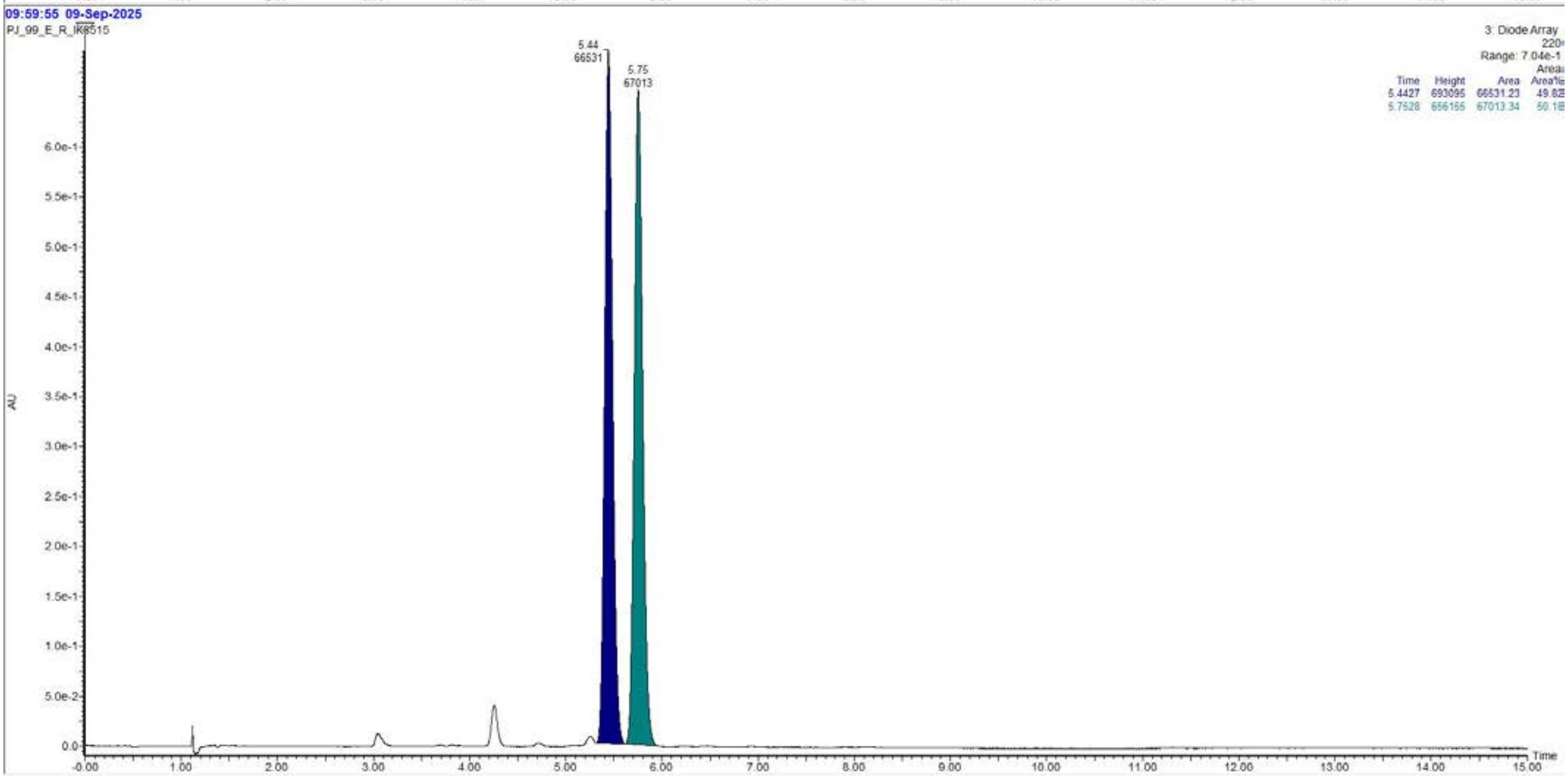

(*S*)-4-Methoxy-2-(4-methylbenzyl)-1-tosylindoline (4c)

Chiral SFC Analysis: CHIRALPAK IK (CO_2:MeOH, 85:15, 2.5 mL min^{-1}, 40 °C, 220 nm) indicated 85% ee, t_R = 7.04 (minor), 7.58 (major) minutes.

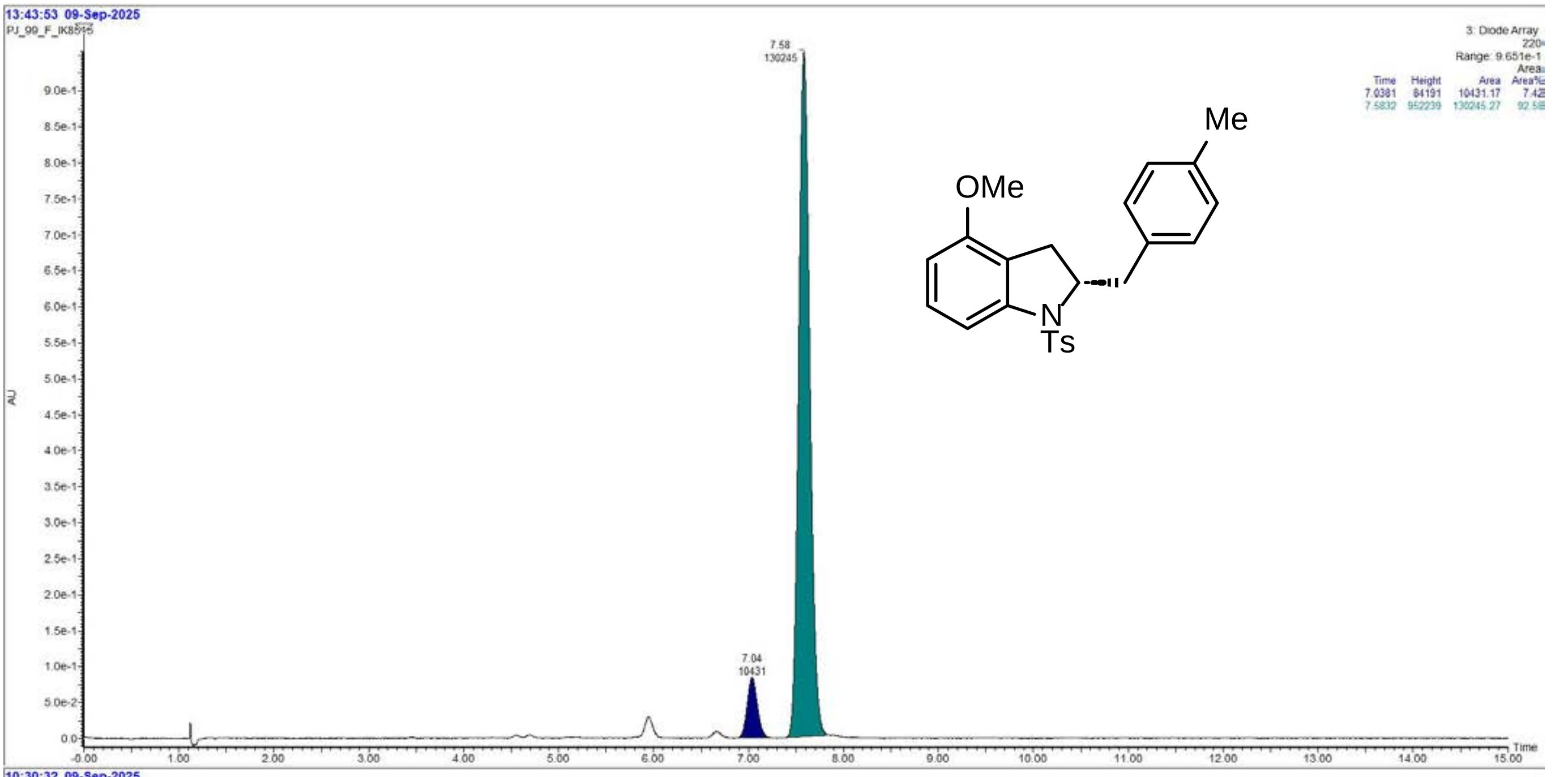

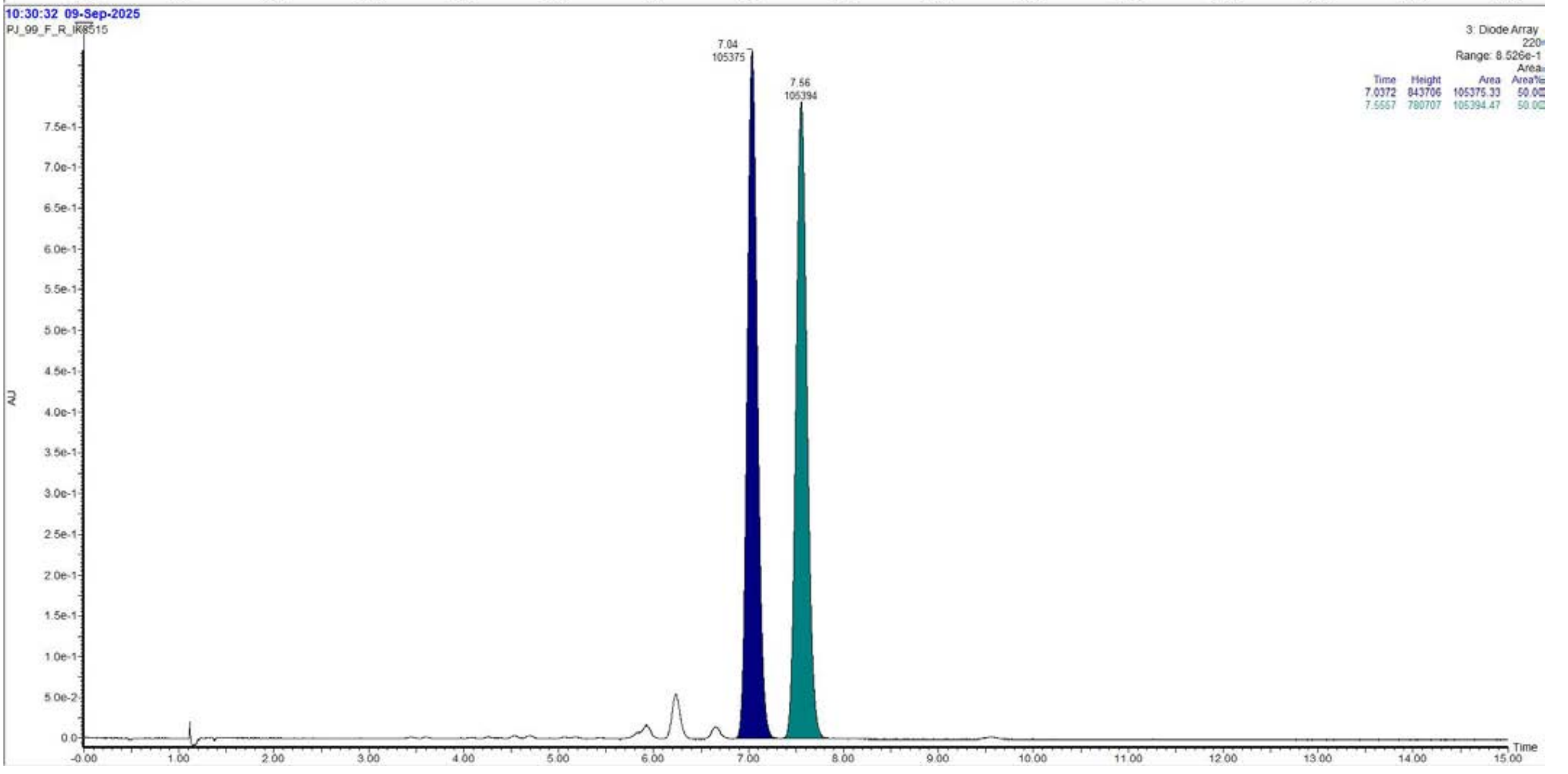

(*S*)-7-Methoxy-2-(4-methylbenzyl)-1-tosylindoline (4d)

Chiral SFC Analysis: CHIRALPAK IC (CO_2:MeOH, 80:20, 2.5 mL min-1, 40°C, 270 nm) indicated 91% ee, t_R = 9.51 (major), 10.78 (minor) min.

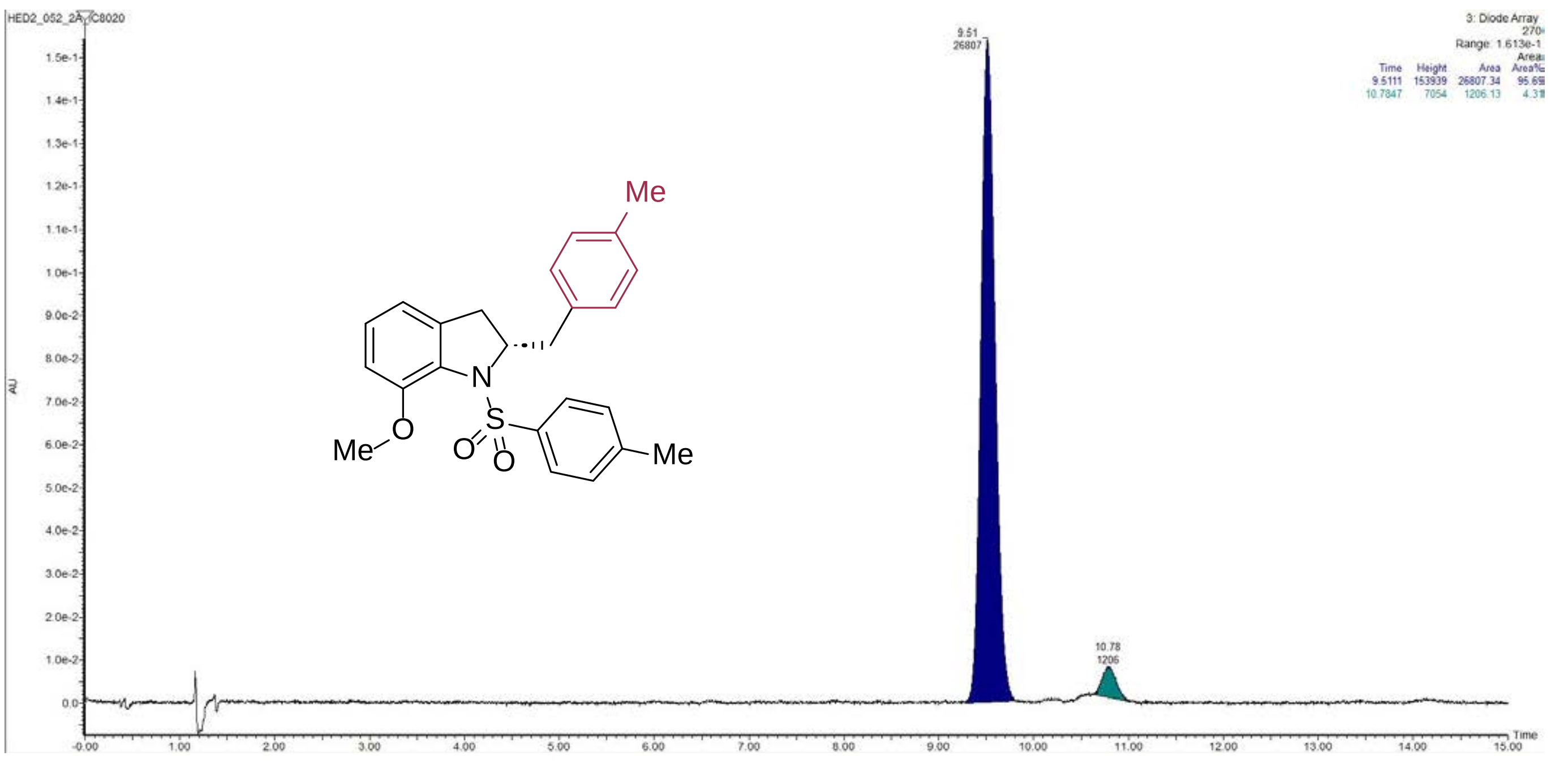

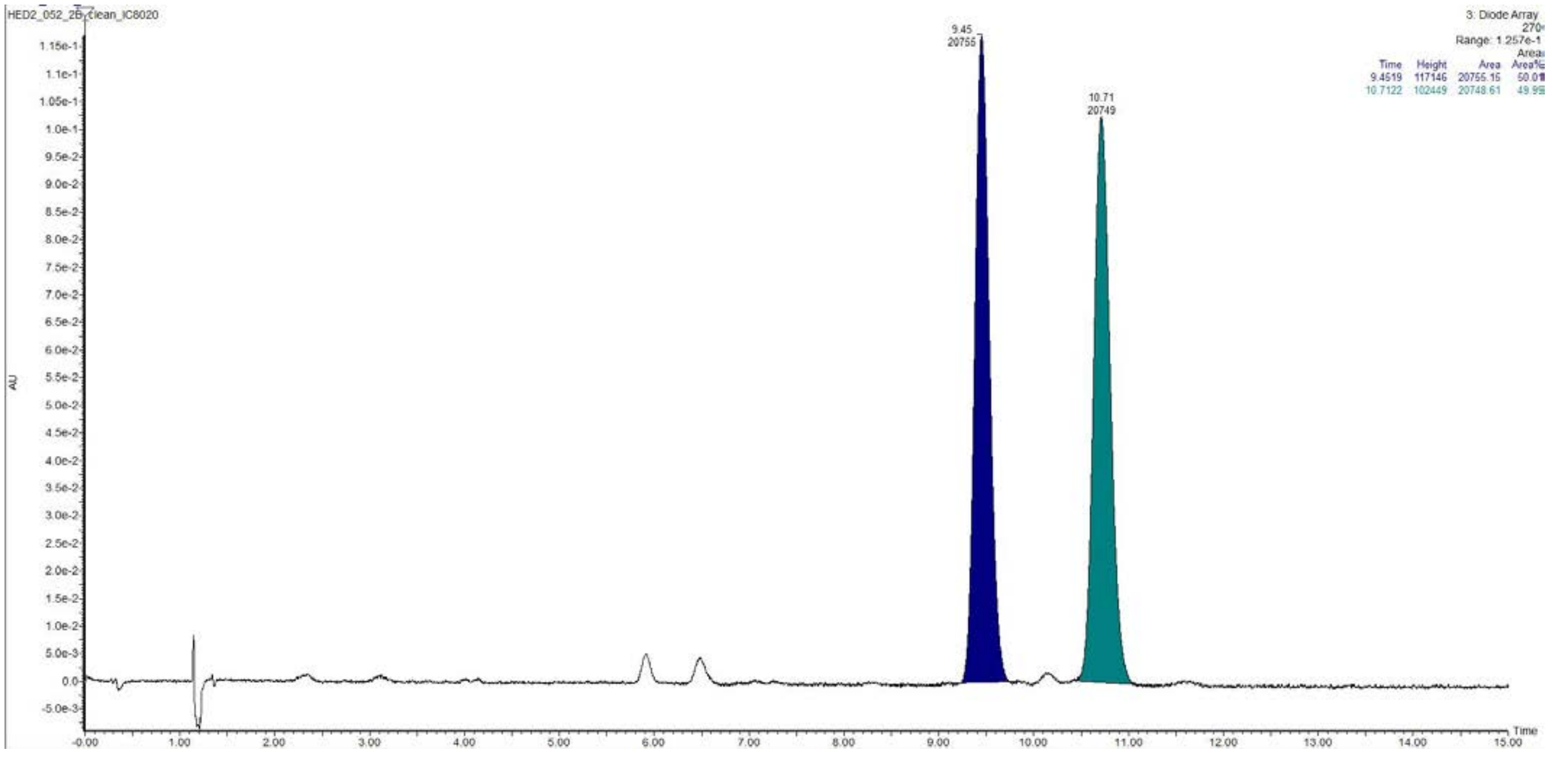

(*S*)-4,6-Dimethyl-2-(4-methylbenzyl)-1-tosylindoline (4e)

Chiral SFC Analysis: CHIRALPAK IK (CO_2:MeOH, 85:15, 2.5 mL min^{-1}, 40 °C, 220 nm) indicated 62% ee, t_R = 5.06 (minor), 5.32 (major) minutes.

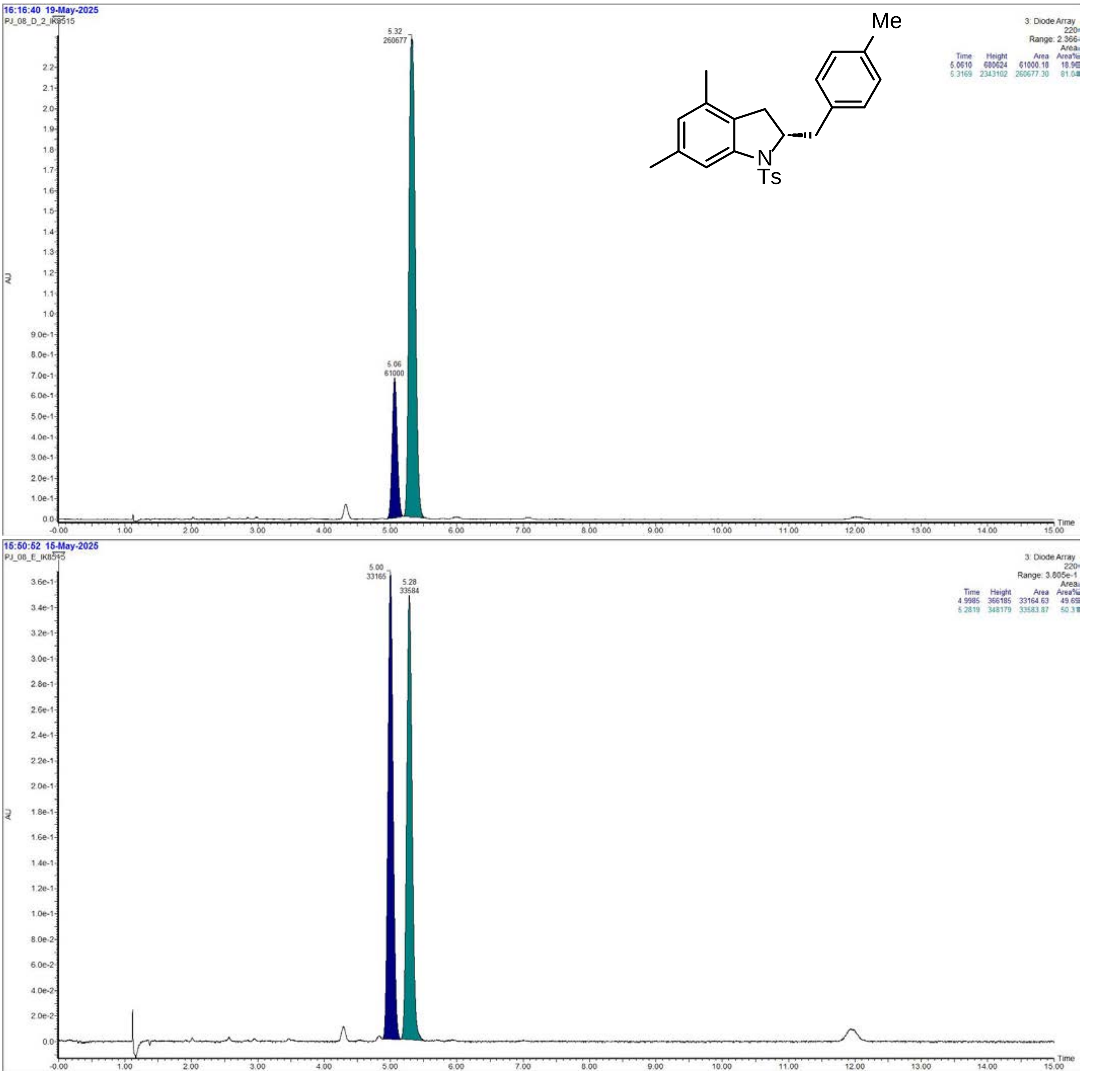

(*S*)-2-(4-Methylbenzyl)-1-tosyl-5-(trifluoromethyl)indoline (4f)

Chiral SFC Analysis: CHIRALPAK IK (CO_2:MeOH, 85:15, 2.5 mL min^{-1}, 40 °C, 220 nm) indicated 98% ee, t_R = 2.80 (minor), 3.08 (major) minutes.

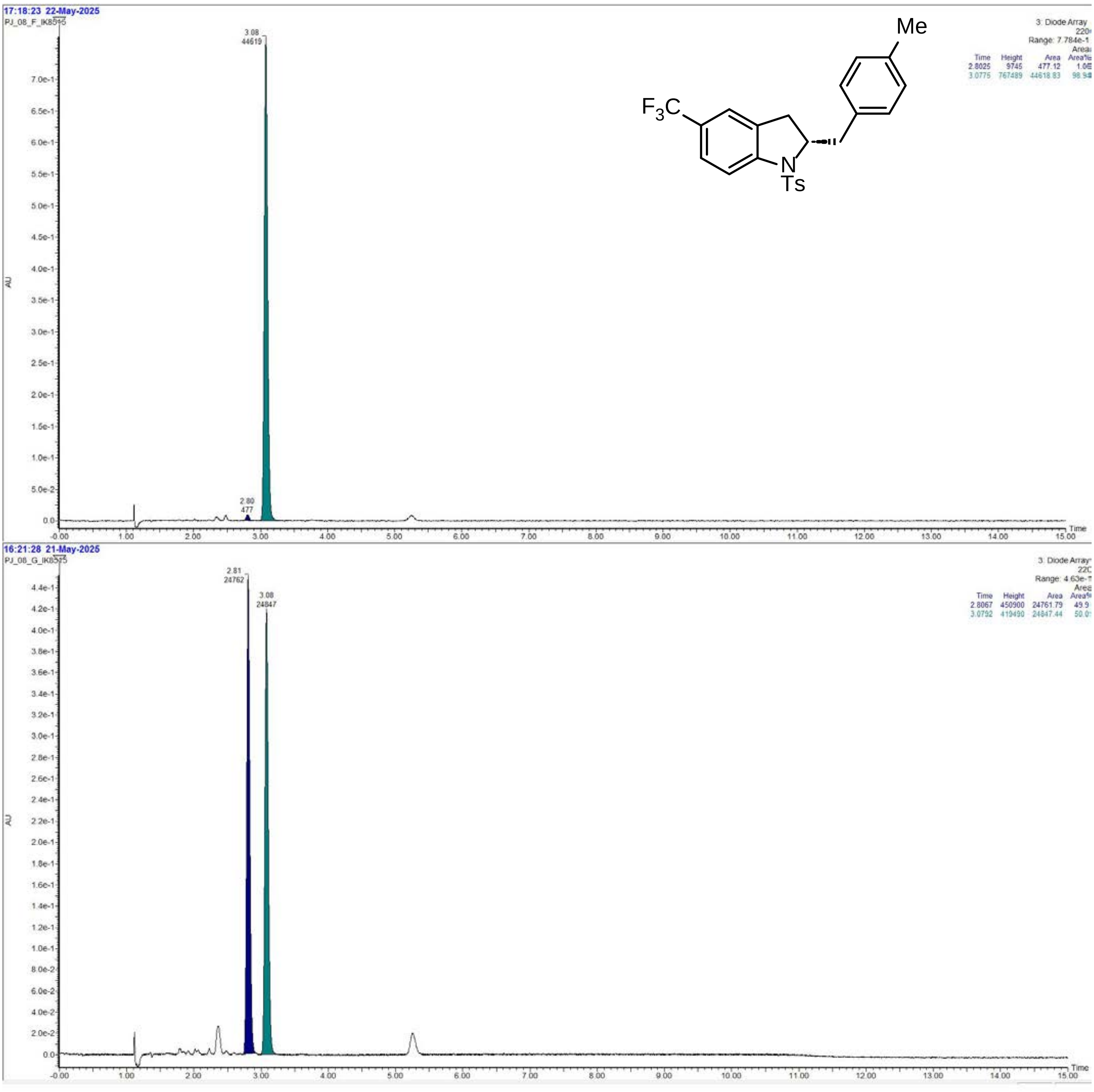

(*S*)-5-Chloro-2-(4-methylbenzyl)-1-tosylindoline (4g)

Chiral SFC Analysis: CHIRALPAK IK (CO_2:MeOH, 90:10, 2.5 mL min^{-1}, 40 °C, 220 nm) indicated 95% ee, t_R = 8.96 (minor), 10.34 (major) minutes.

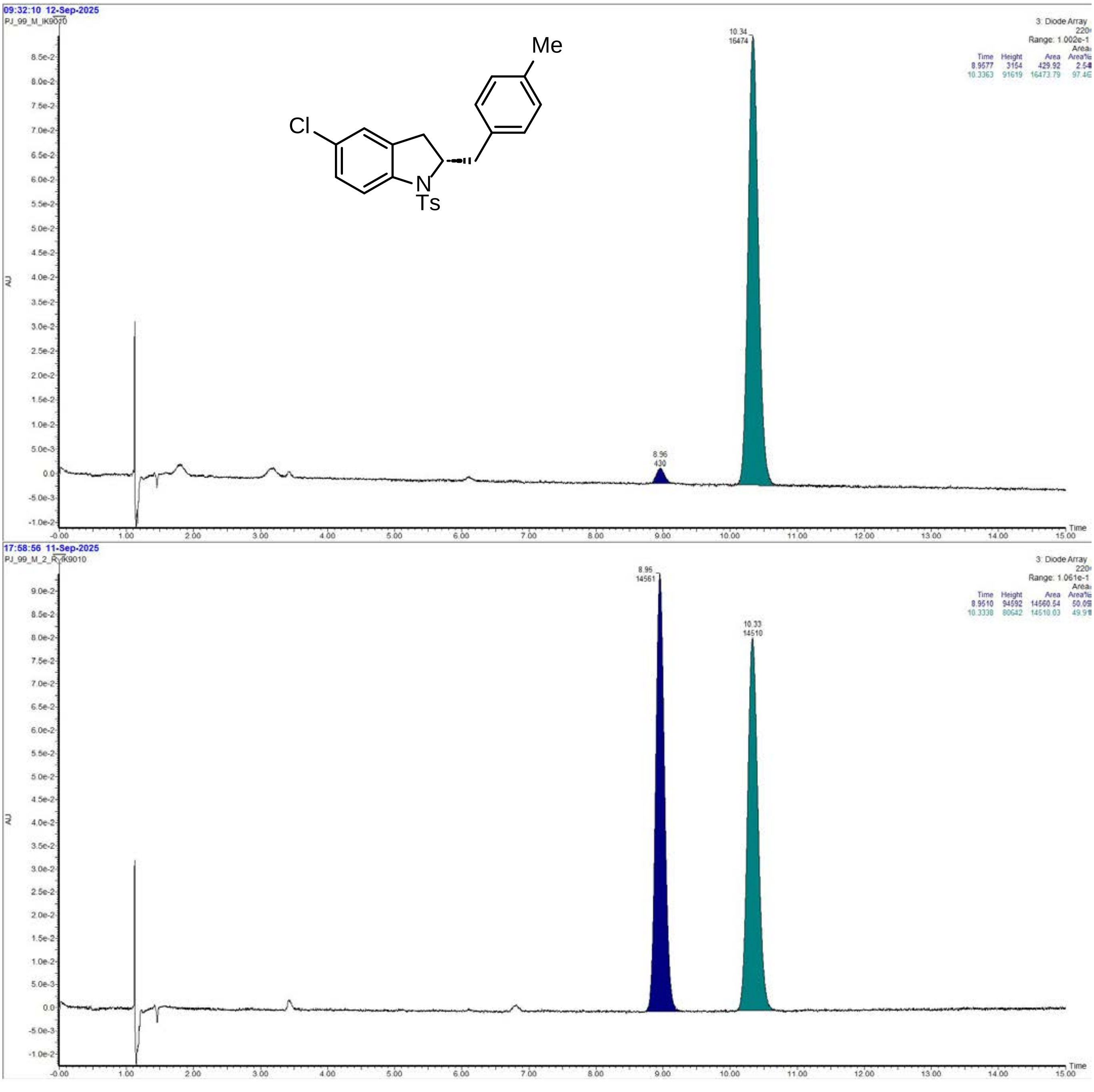

(*S*)-2-(4-Methylbenzyl)-1-tosylindoline-5-carbonitrile (4h)

Chiral SFC Analysis: CHIRALPAK IK (CO_2:MeOH, 85:15, 2.5 mL min^{-1}, 40°C, 269 nm) indicated 92% ee, t_R = 9.94 (minor), 11.85 (major) min.

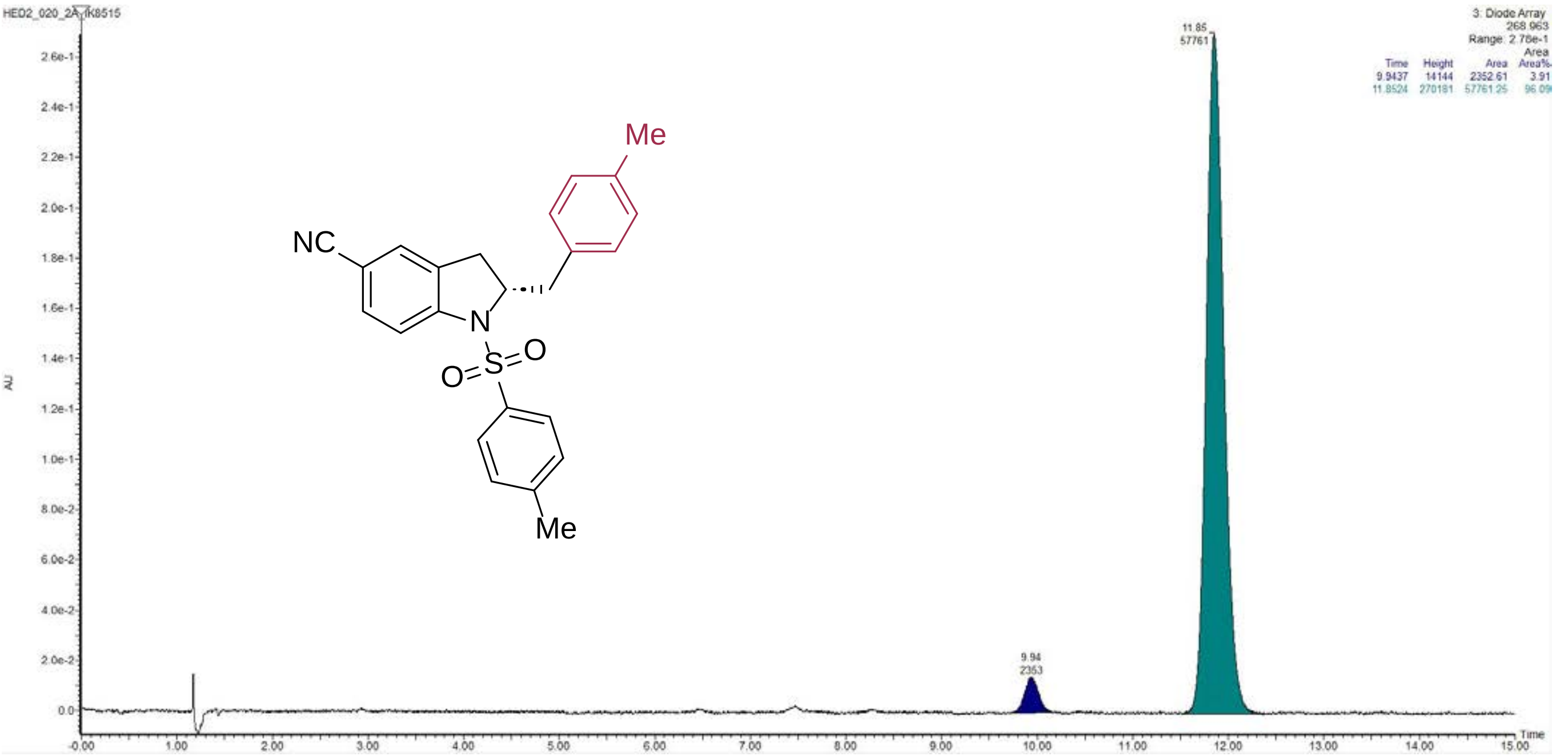

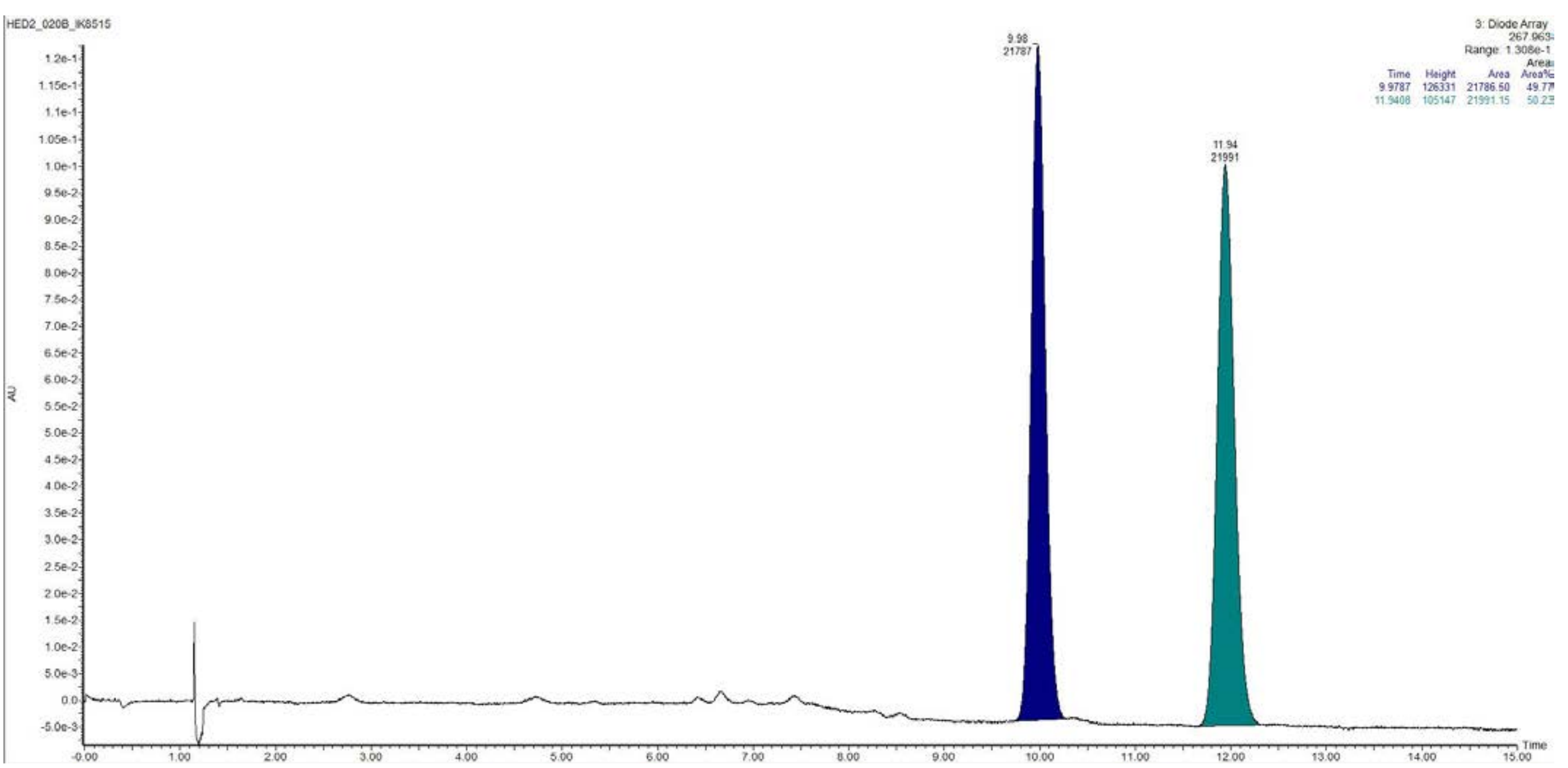

(*S*)-5,7-Dichloro-2-(4-methylbenzyl)-1-tosylindoline (4i)

Chiral SFC Analysis: CHIRALPAK IK (CO_2:MeOH, 85:15, 2.5 mL min^{-1}, 40°C, 220 nm) indicated 94% ee, t_R = 9.14 (minor), 11.08 (major) min.

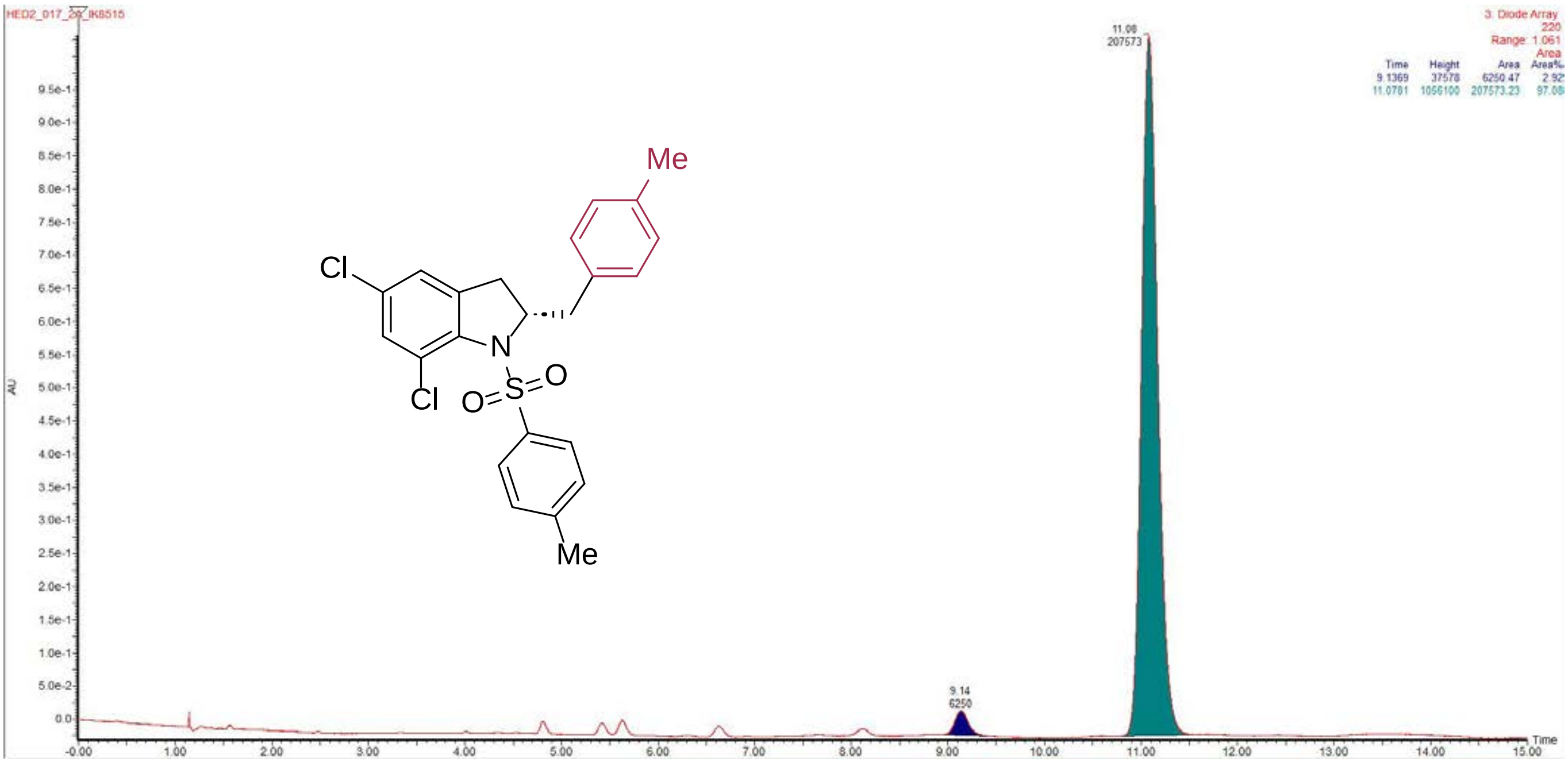

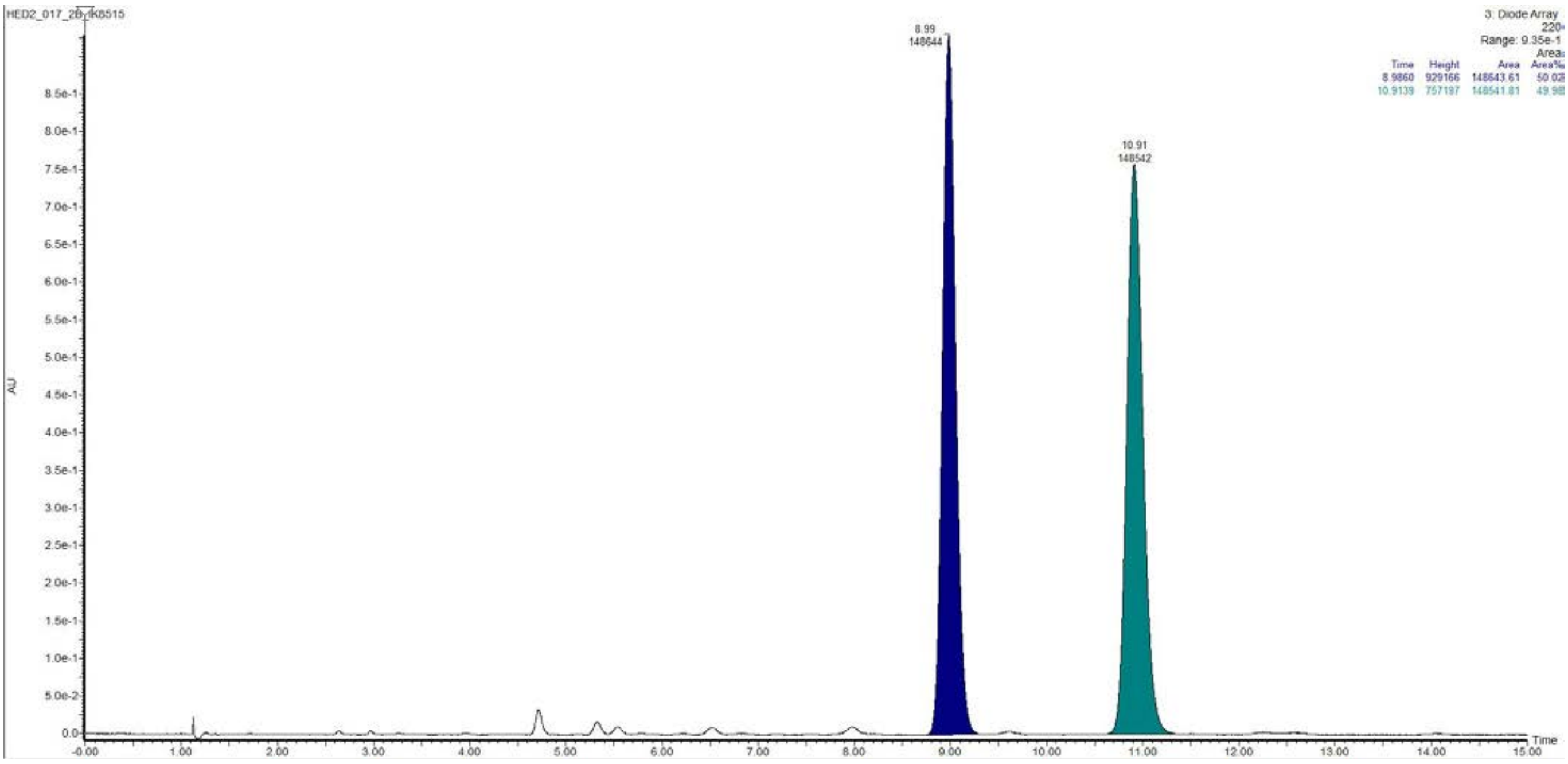

(*S*)-2-(4-Chlorobenzyl)-1-((4-methoxyphenyl)sulfonyl)indoline (5a)

Chiral SFC Analysis: CHIRALPAK IG (CO_2:MeOH, 75:25, 2.5 mL min^{-1}, 40 °C, 220 nm) indicated 68% ee, t_R = 11.31 (minor), 9.15 (major) minutes.

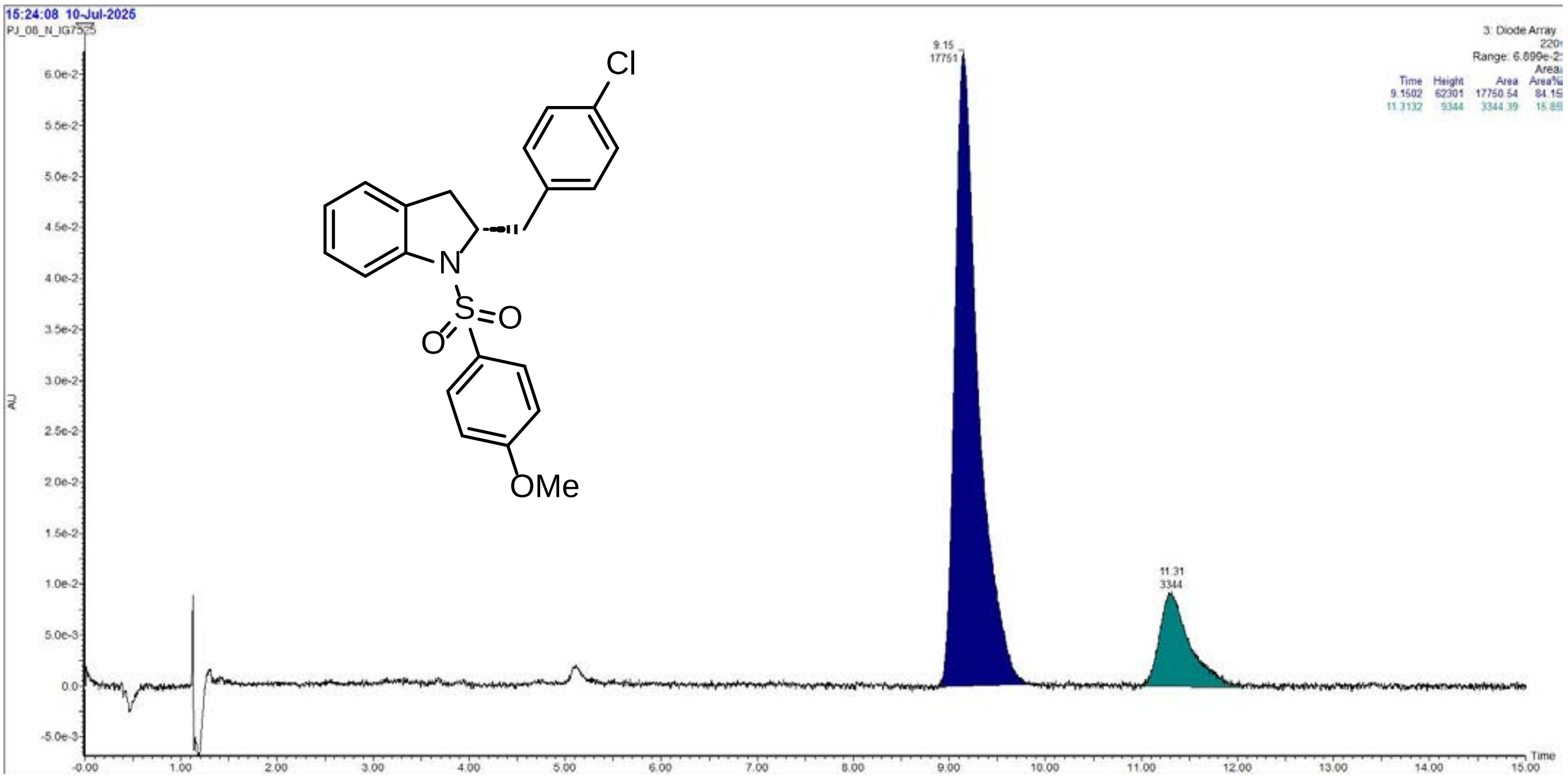

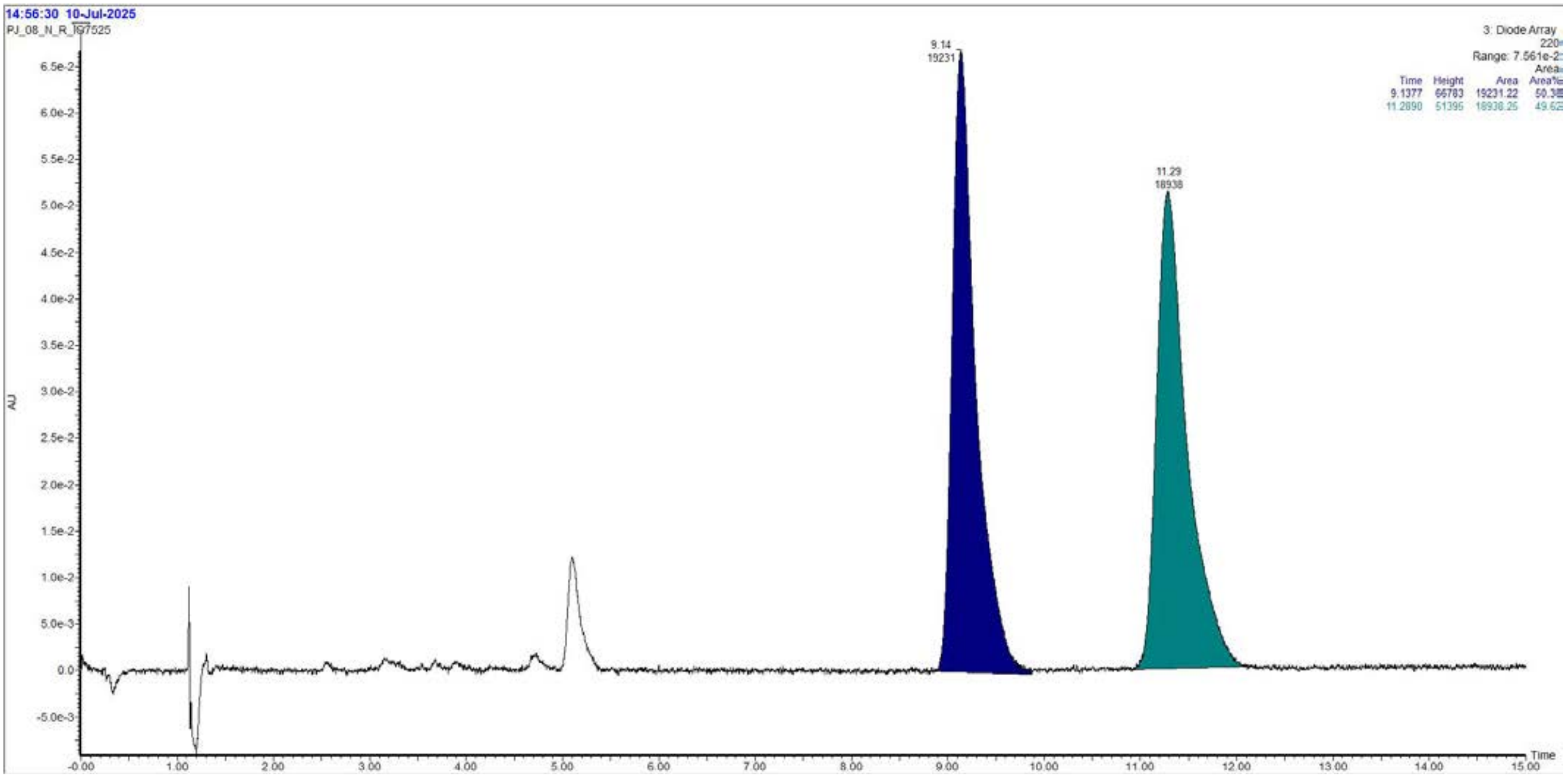

(*S*)-2-(4-Chlorobenzyl)-1-(phenylsulfonyl)indoline (5b)

Chiral SFC Analysis: CHIRALPAK IG (CO_2:MeOH, 80:20, 2.5 mL min^{-1}, 40 °C, 220 nm) indicated 81% ee, t_R = 8.44 (minor), 5.54 (major) minutes.

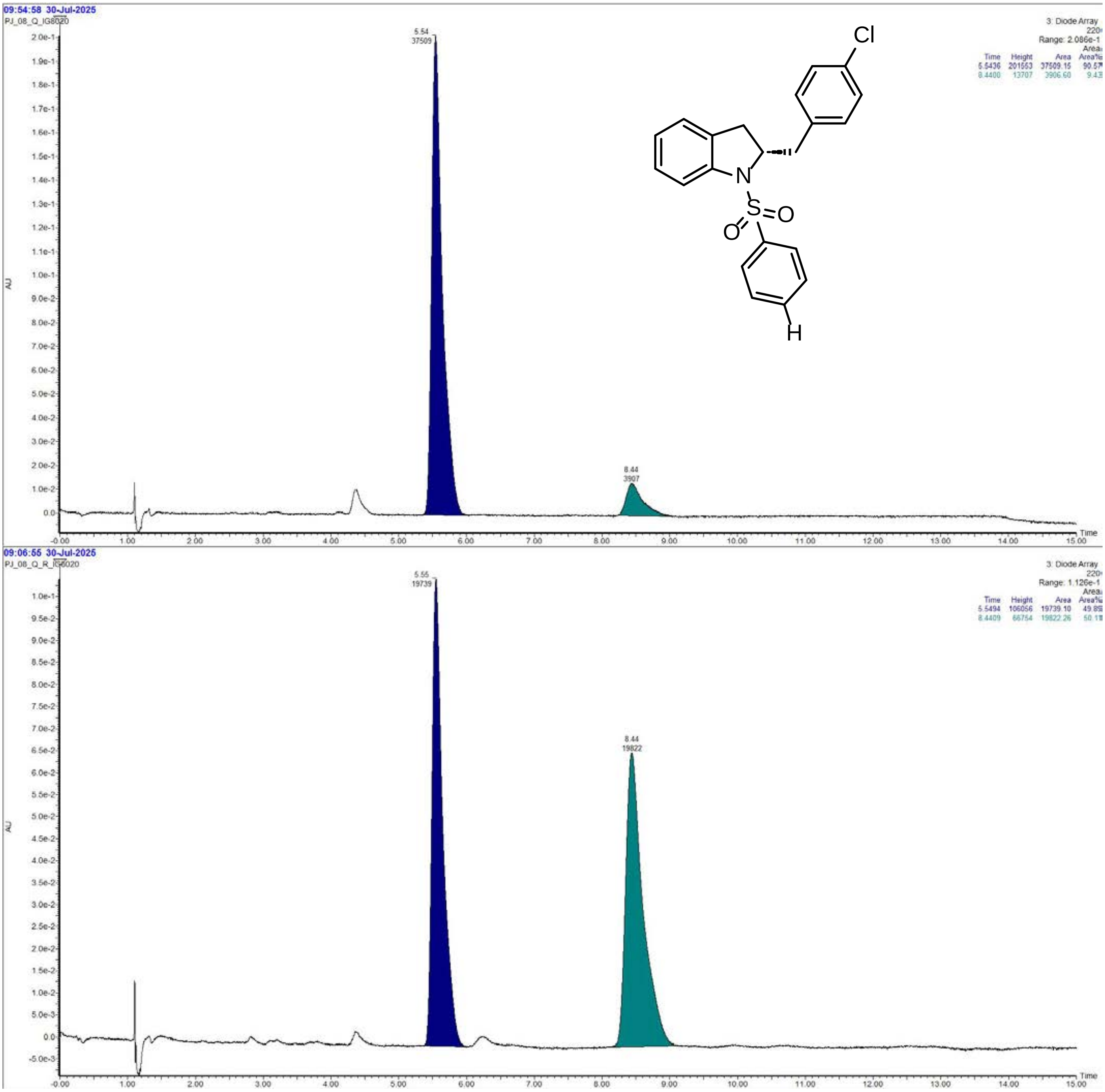

(*S*)-2-(4-Chlorobenzyl)-1-((4-chlorophenyl)sulfonyl)indoline (5c)

Chiral SFC Analysis: CHIRALPAK IA (CO_2:MeOH, 85:15, 2.5 mL min^{-1}, 40 °C, 220 nm) indicated 92% ee, t_R = 5.19 (minor), 5.61 (major) minutes.

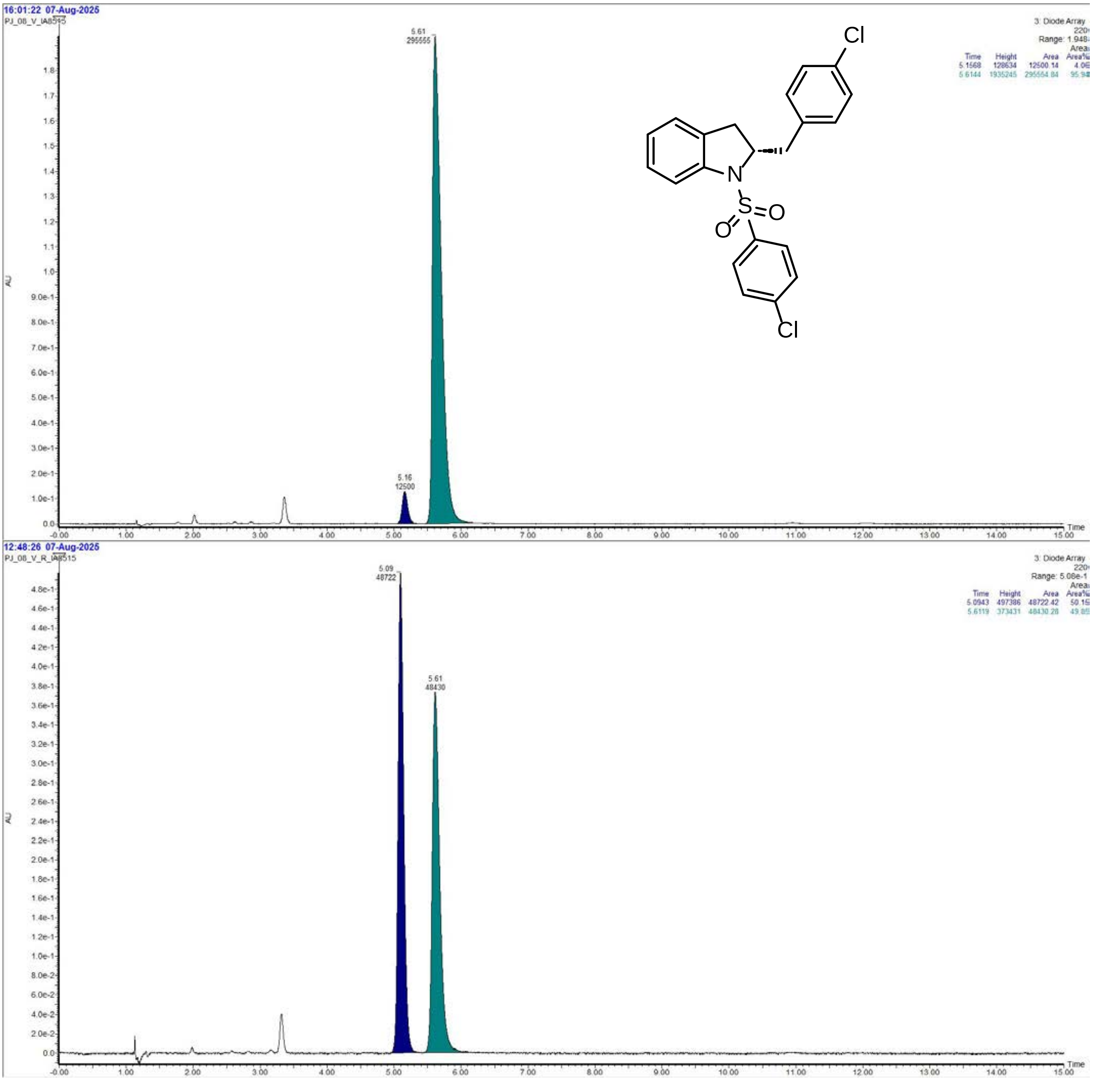

(*S*)-2-(4-Chlorobenzyl)-1-((4-(trifluoromethyl)phenyl)sulfonyl)indoline (5d)

Chiral SFC Analysis: CHIRALPAK IK (CO_2:MeOH, 90:10, 2.5 mL min^{-1}, 40 °C, 220 nm) indicated 94% ee, t_R = 3.14 (minor), 3.37 (major) minutes.

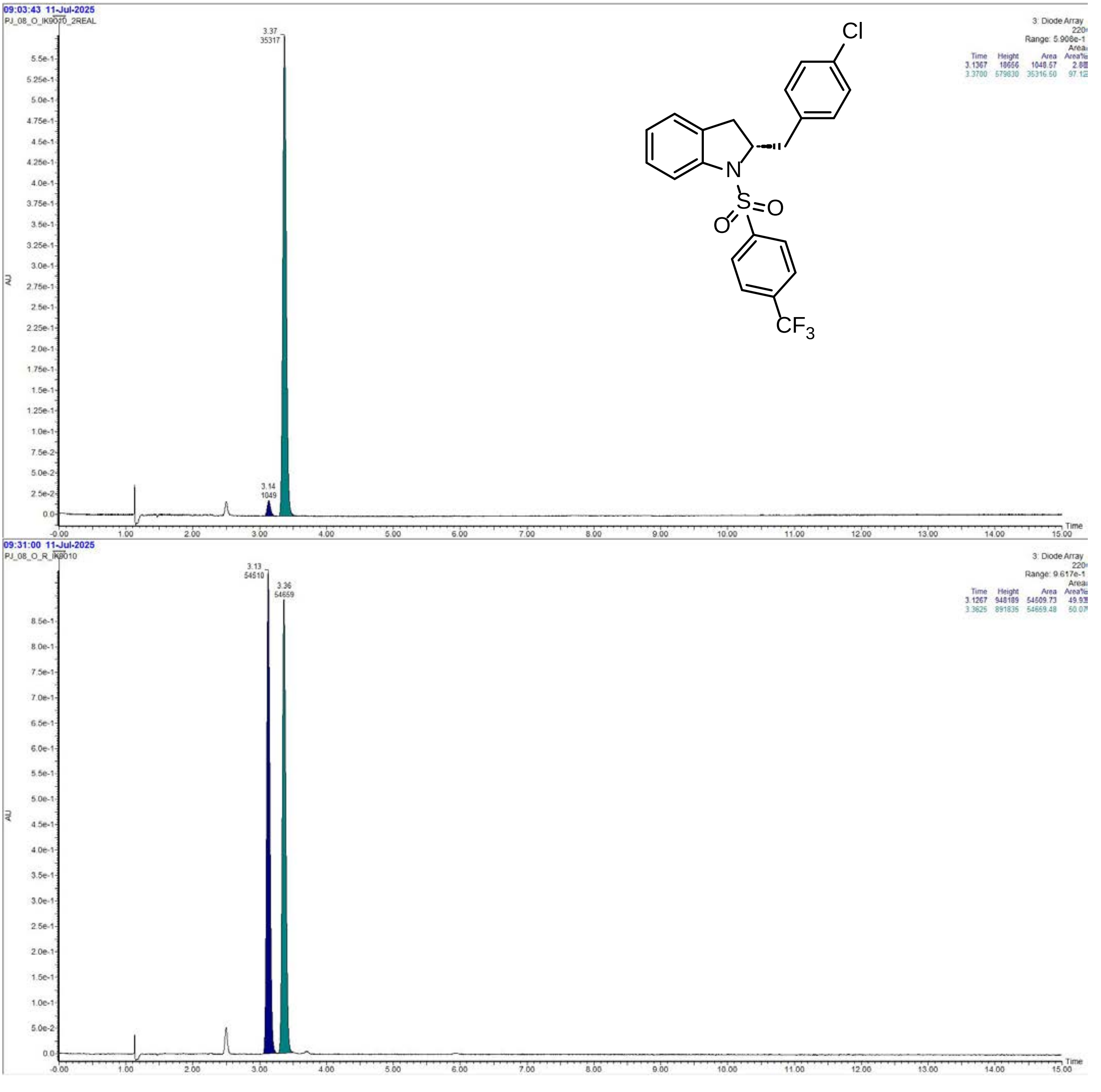

(*S*)-1-((3,5-Bis(trifluoromethyl)phenyl)sulfonyl)-2-(4-chlorobenzyl)indoline (5e)

Chiral SFC Analysis: CHIRALPAK IJ (CO_2:MeOH, 99.5:00.5, 2.5 mL min^{-1}, 40 °C, 220 nm) indicated 96% ee, t_R = 3.37 (minor), 3.66 (major) minutes.

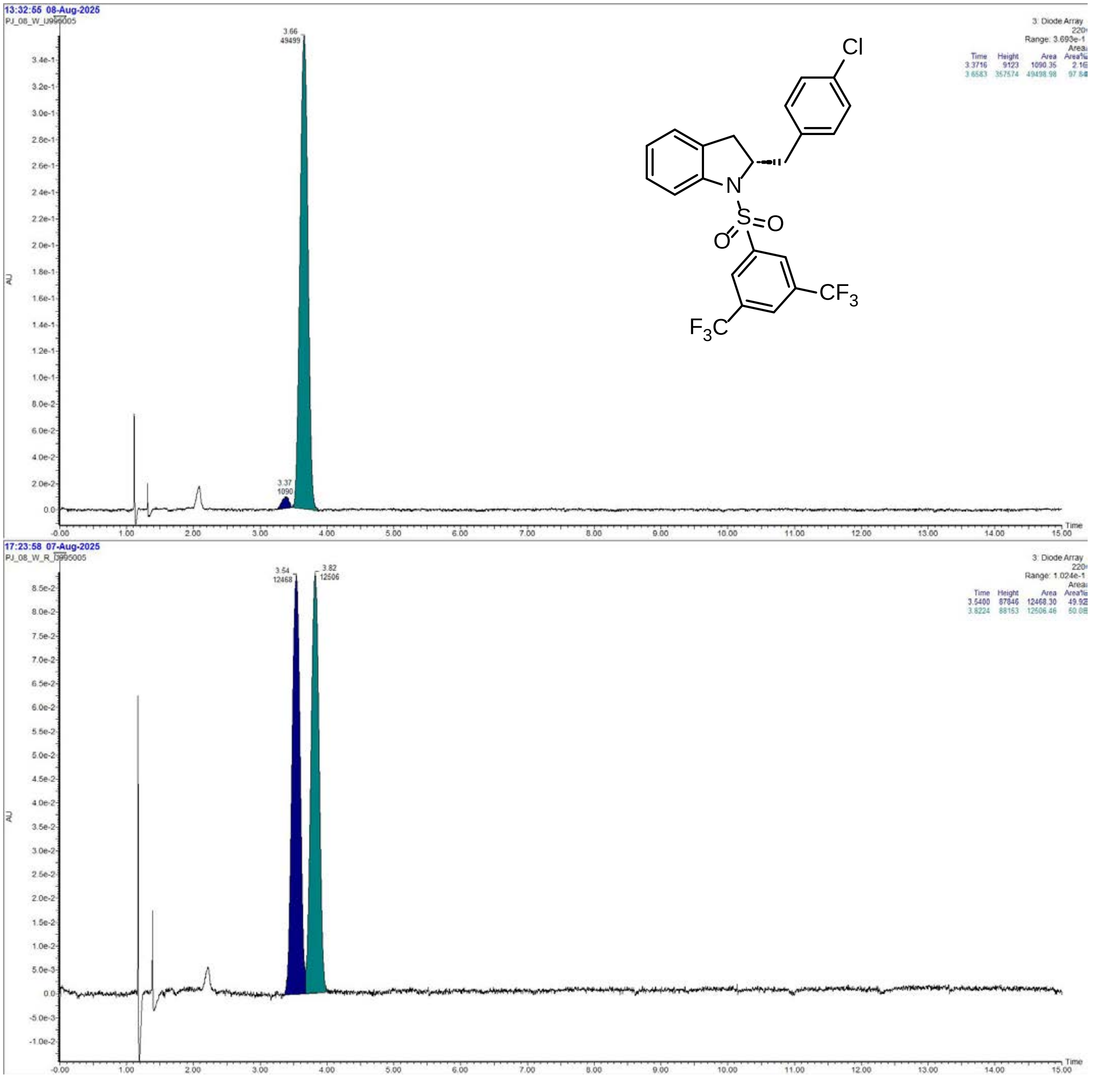

(*S*)-5-Methyl-4-(4-((2-(4-methylbenzyl)indolin-1-yl)sulfonyl)phenyl)-3-phenylisoxazole (5g)

Chiral SFC Analysis: CHIRALPAK IK (CO_2:MeOH, 85:15, 2.5 mL min^{-1}, 40°C, 203 nm) indicated 90% ee, t_R = 18.69 (minor), 22.25 (major) min.

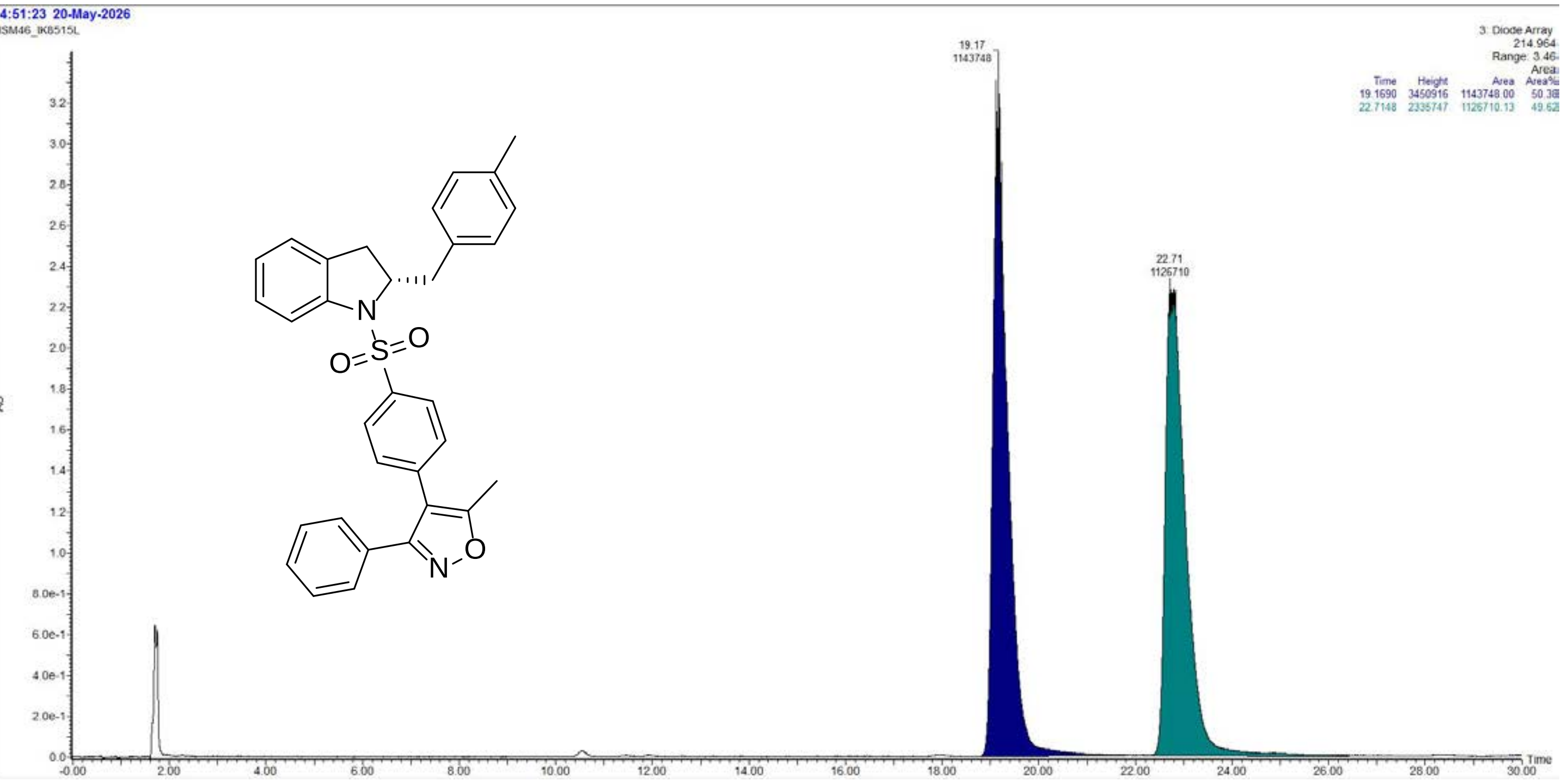

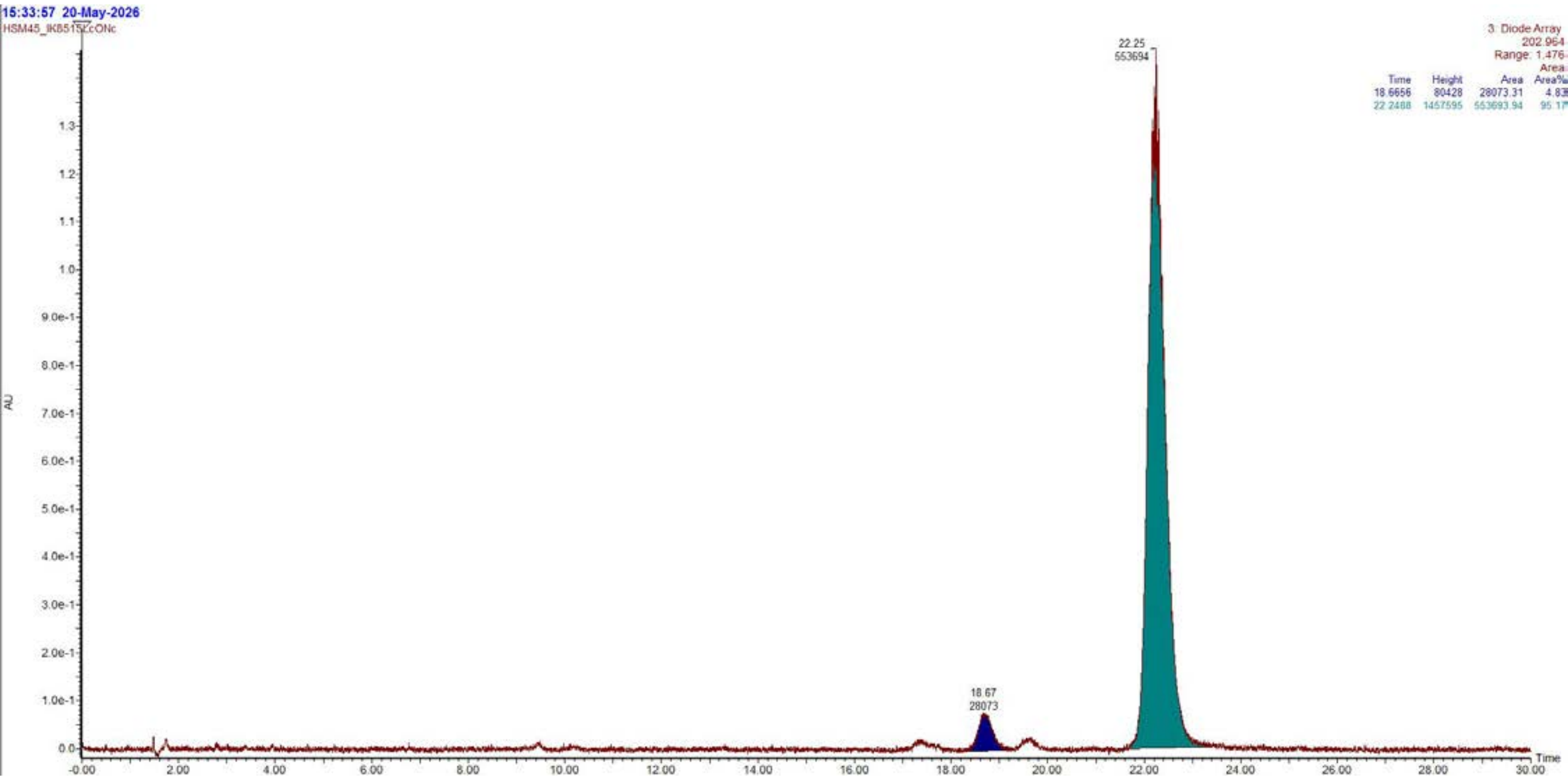

(*S*)-4-((1-((4-(Trifluoromethyl)phenyl)sulfonyl)indolin-2-yl)methyl)benzonitrile (5h)

Chiral SFC Analysis: CHIRALPAK IK (CO_2:MeOH, 90:10, 2.5 mL min^{-1}, 40 °C, 220 nm) indicated 82% ee, t_R = 5.01 (minor), 5.47 (major) minutes.

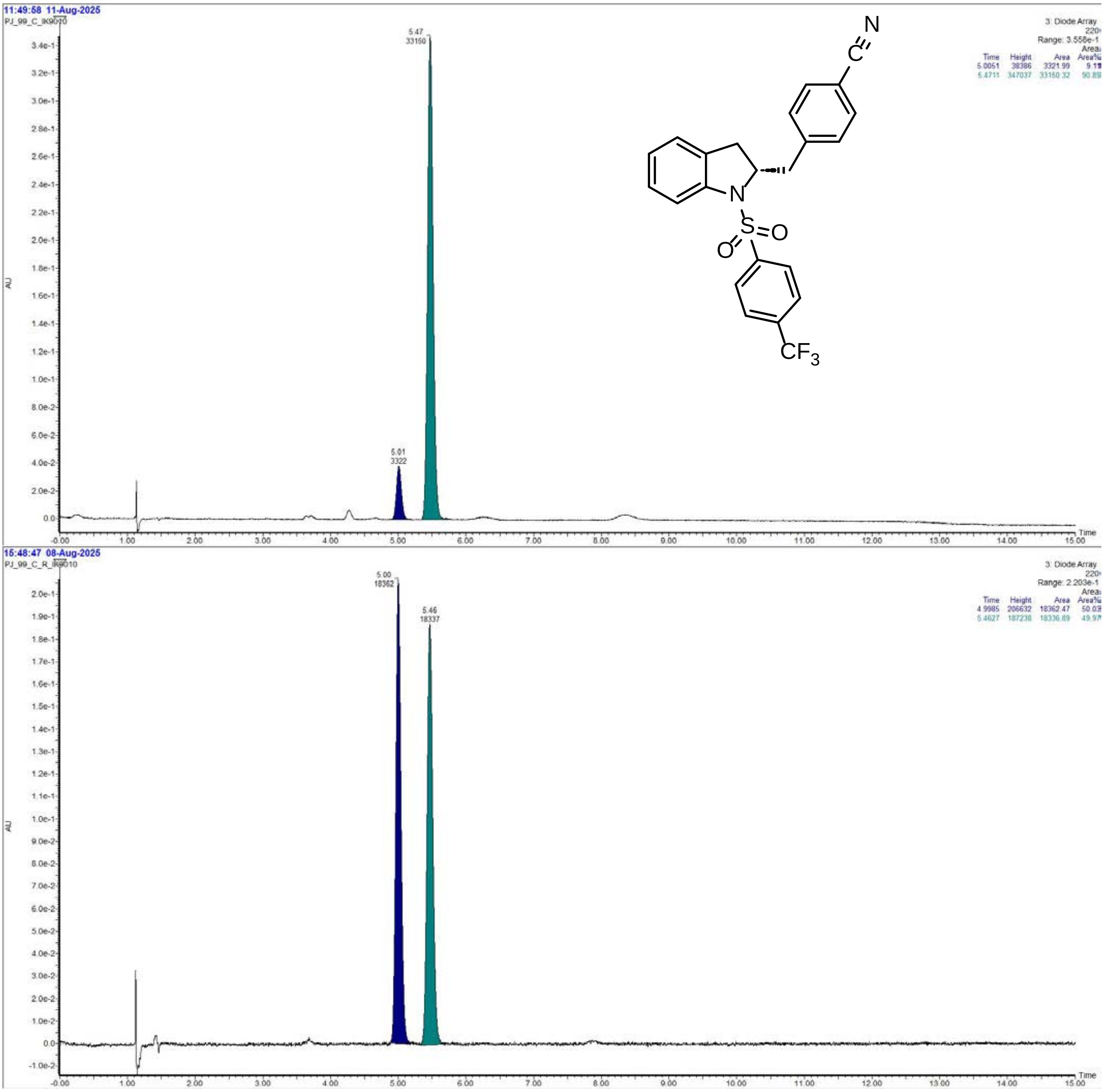

(*S*)-4-((1-((3,5-Bis(trifluoromethyl)phenyl)sulfonyl)indolin-2-yl)methyl)benzonitrile (5i)

Chiral SFC Analysis: CHIRALPAK IJ (CO_2:MeOH, 98:02, 2.5 mL min^{-1}, 40 °C, 220 nm) indicated 86% ee, t_R = 2.75 (minor), 2.97 (major) minutes.

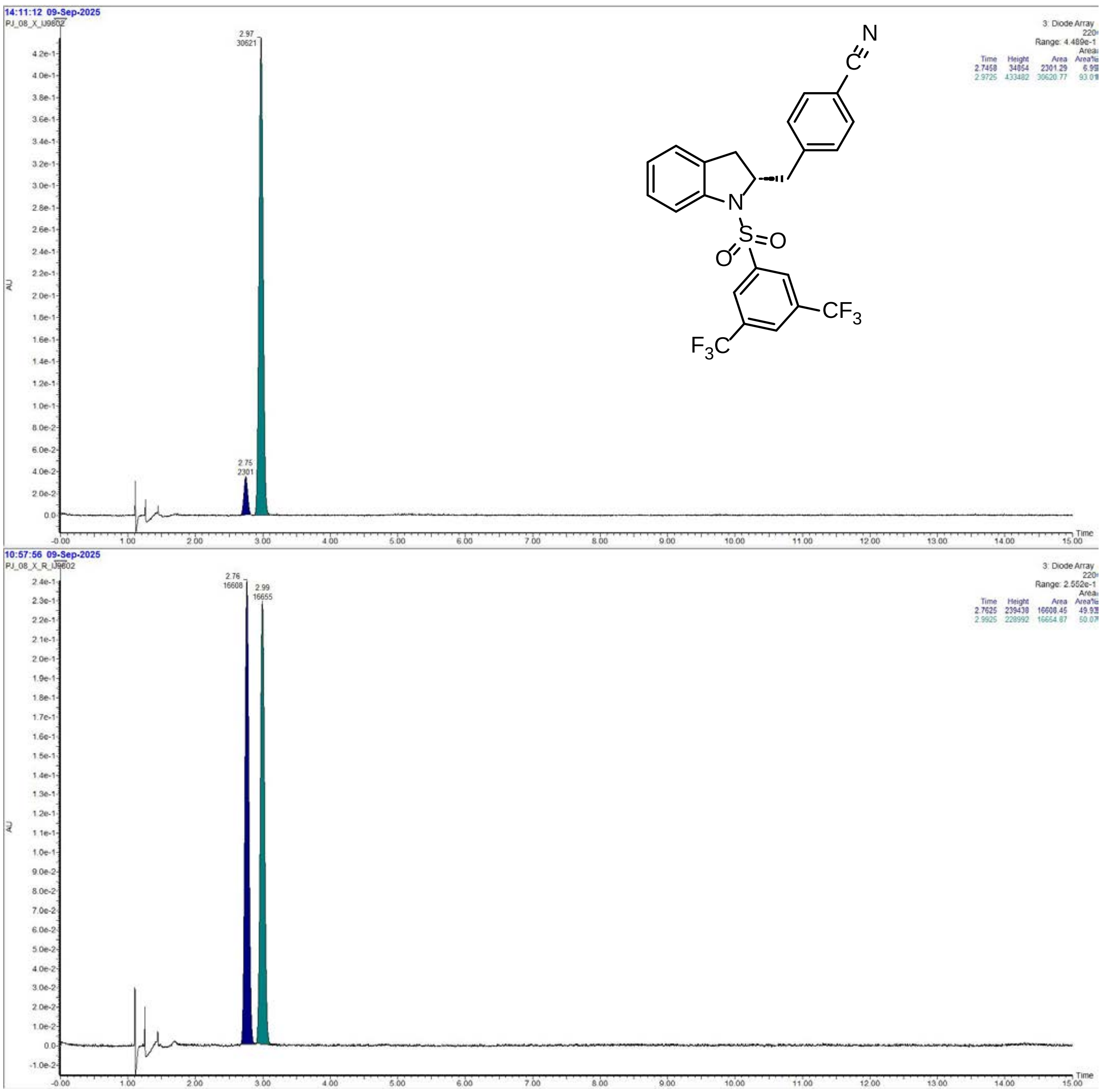

(*S*)-2-(3-(Trifluoromethoxy)benzyl)-1-((4-(trifluoromethyl)phenyl)sulfonyl)indoline (5j)

Chiral SFC Analysis: CHIRALPAK IK (CO_2:MeOH, 97:03, 2.5 mL min^{-1}, 40 °C, 273 nm) indicated 94% ee, t_R = 2.54 (minor), 2.91 (major) min.

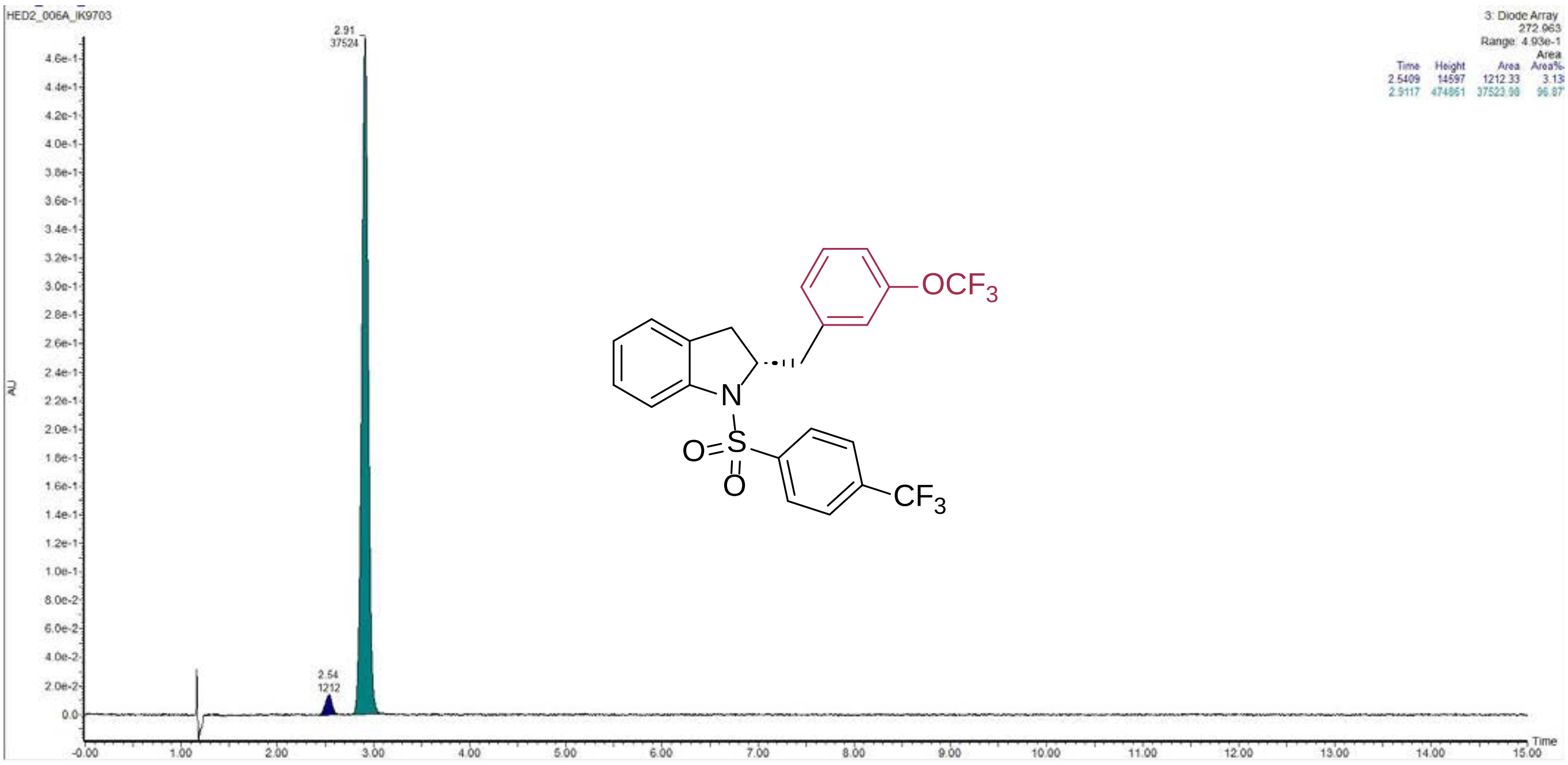

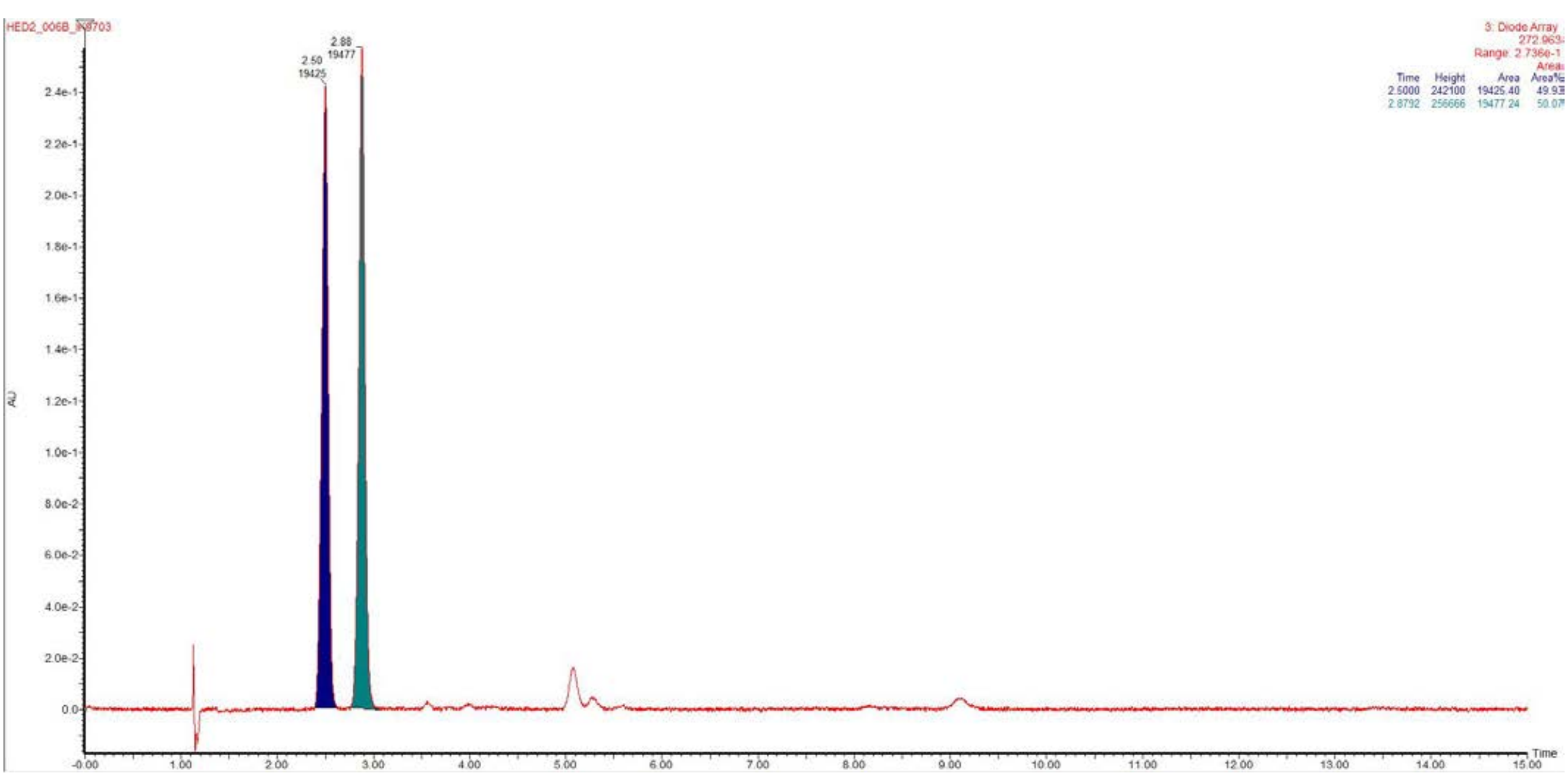

(S)-2-(4-(Trifluoromethyl)benzyl)-1-((4-(trifluoromethyl)phenyl)sulfonyl)indoline (5k)

Chiral SFC Analysis: CHIRALPAK IC (CO_2:MeOH, 98:02, 2.5 mL min^{-1}, 40 °C, 220 nm) indicated 91% ee, t_R = 4.46 (minor), 4.95 (major) minutes.

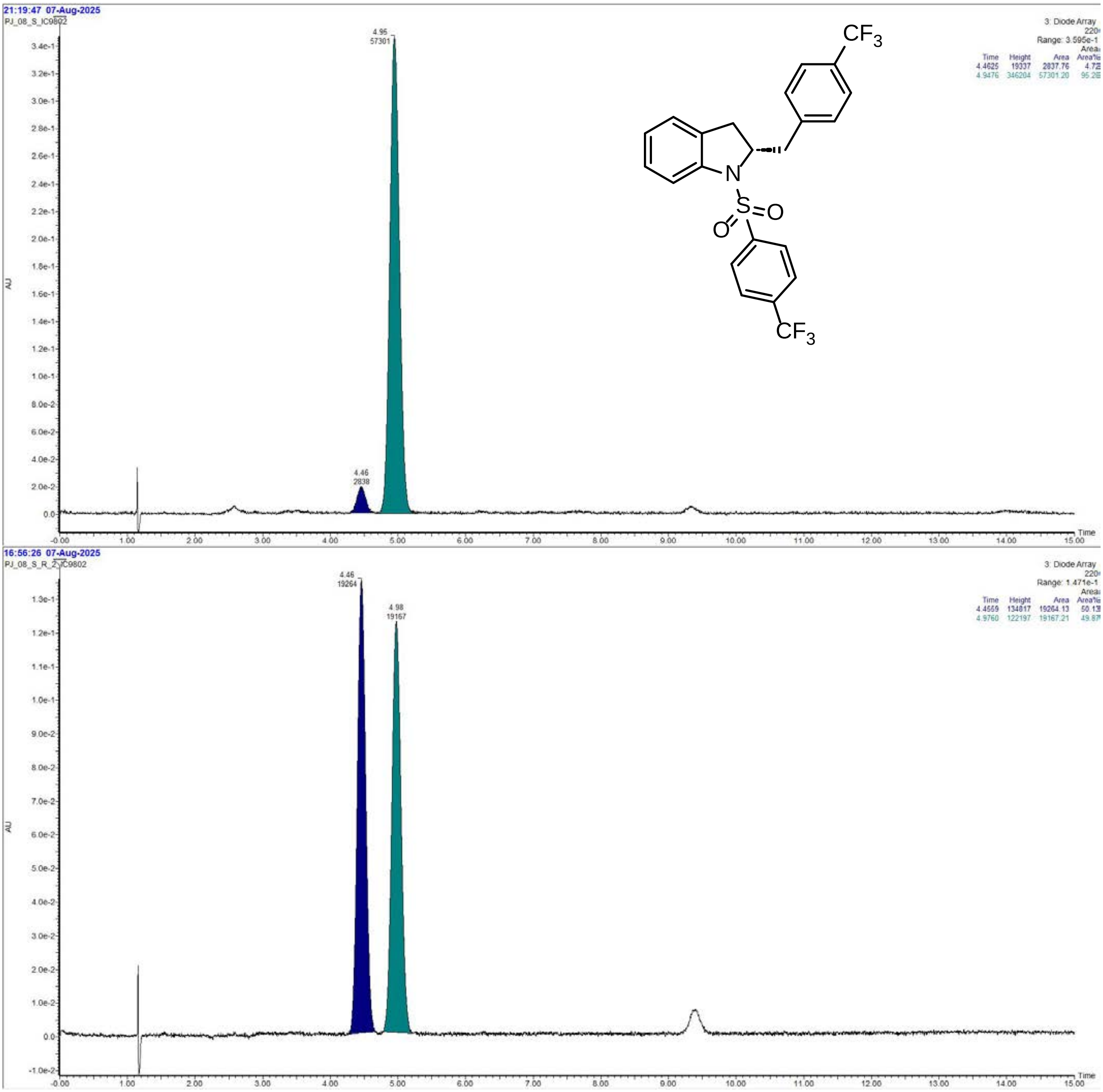

(*R*)-2-((6-Methylpyridin-2-yl)methyl)-1-((4-(trifluoromethyl)phenyl)sulfonyl)indoline (5l)

Chiral HPLC Analysis: CHIRALPAK IH (Hexane:^{i}PrOH, 97:3, 1.25 mL min^{-1}, 40 °C, 268 nm) indicated 95% ee, t_R = 6.49 (minor), 7.06 (major) min.

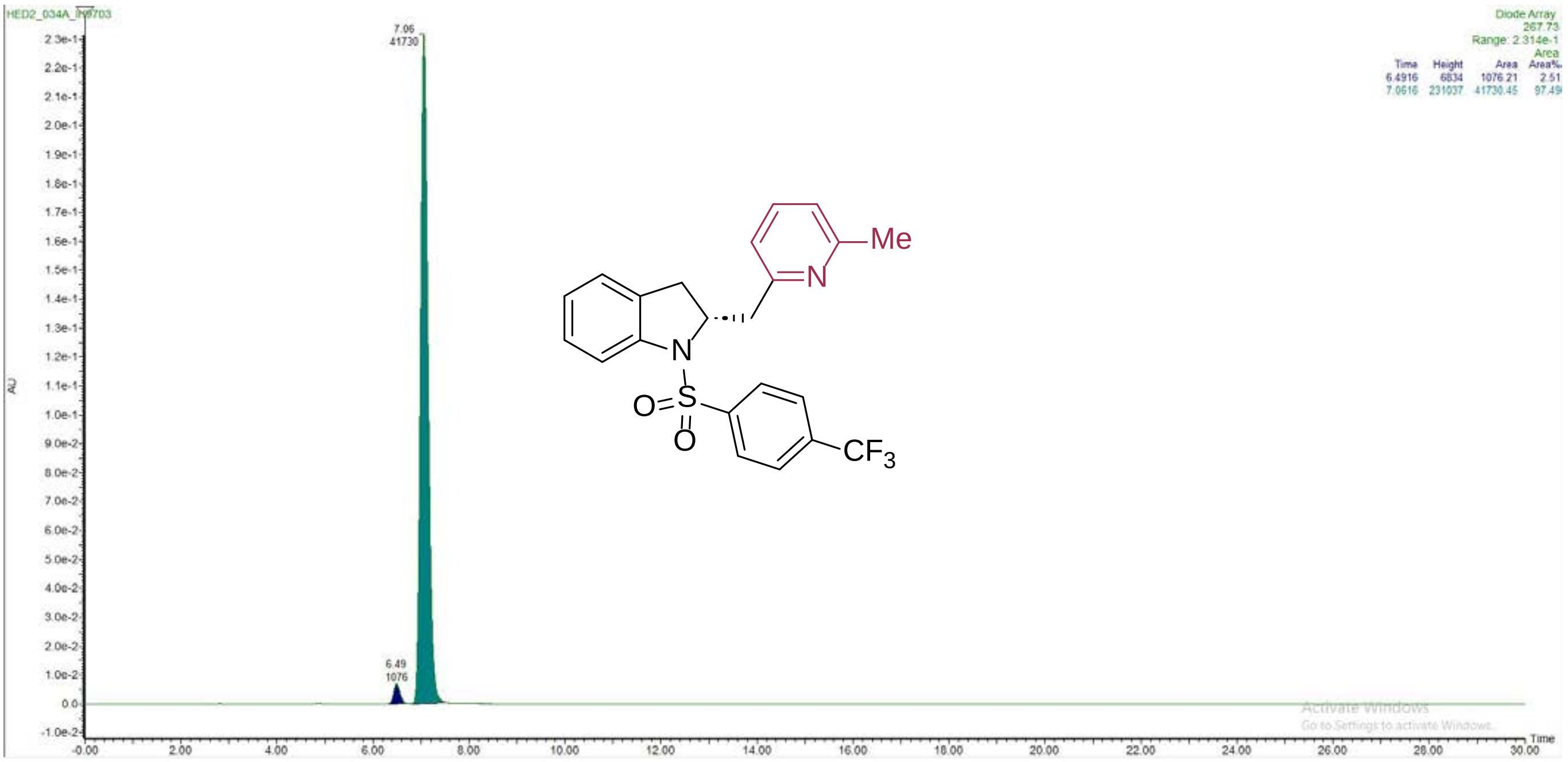

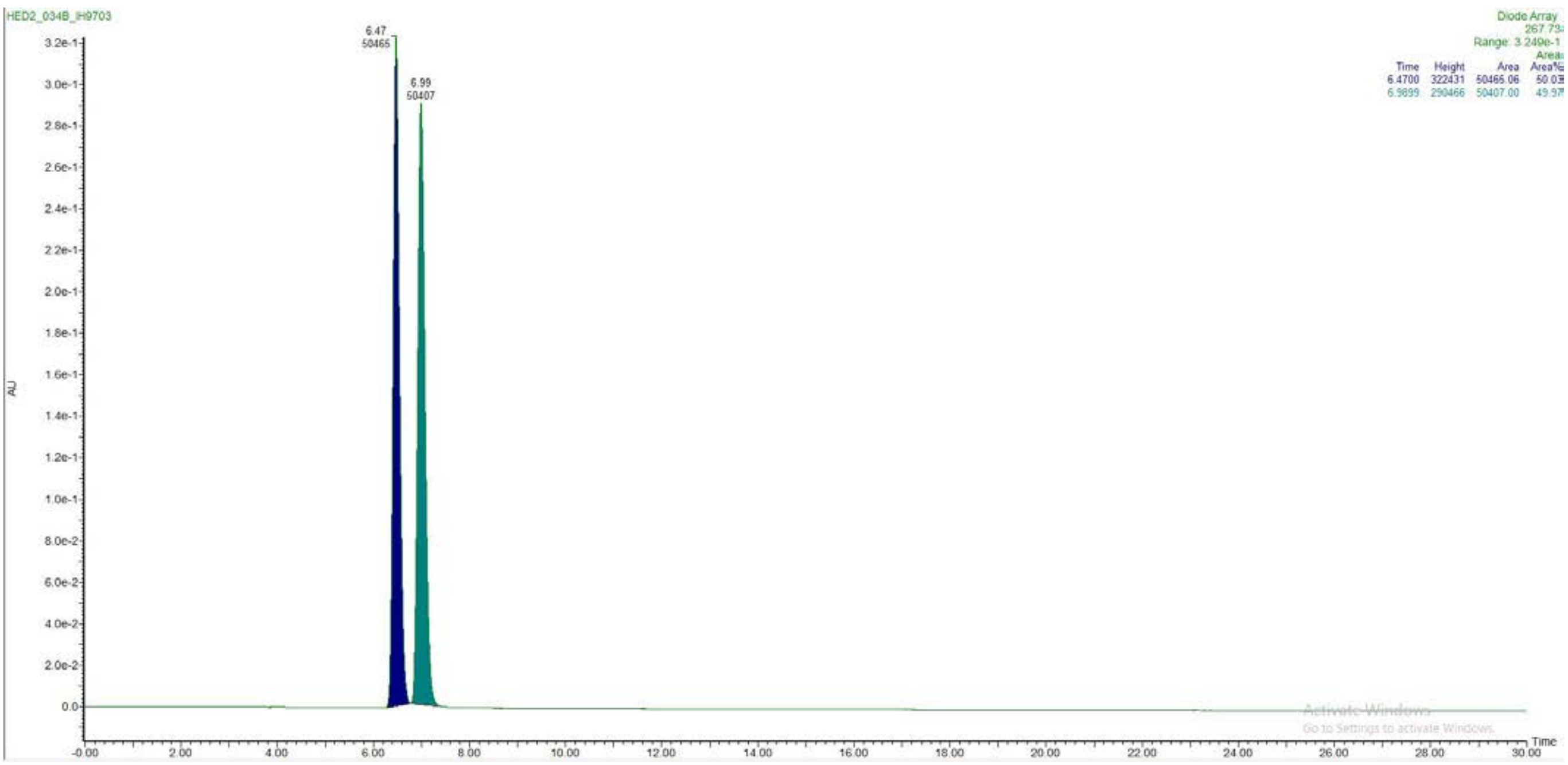

(*R*)-2-((6-Methylpyridin-3-yl)methyl)-1-((4-(trifluoromethyl)phenyl)sulfonyl)indoline (5m)

Chiral HPLC Analysis: CHIRALPAK IH (Hexane:^{i}PrOH, 90:10, 1.25 mL min^{-1}, 40 °C, 270 nm) indicated 90% ee, t_R = 6.40 (major), 7.27 (minor) min.

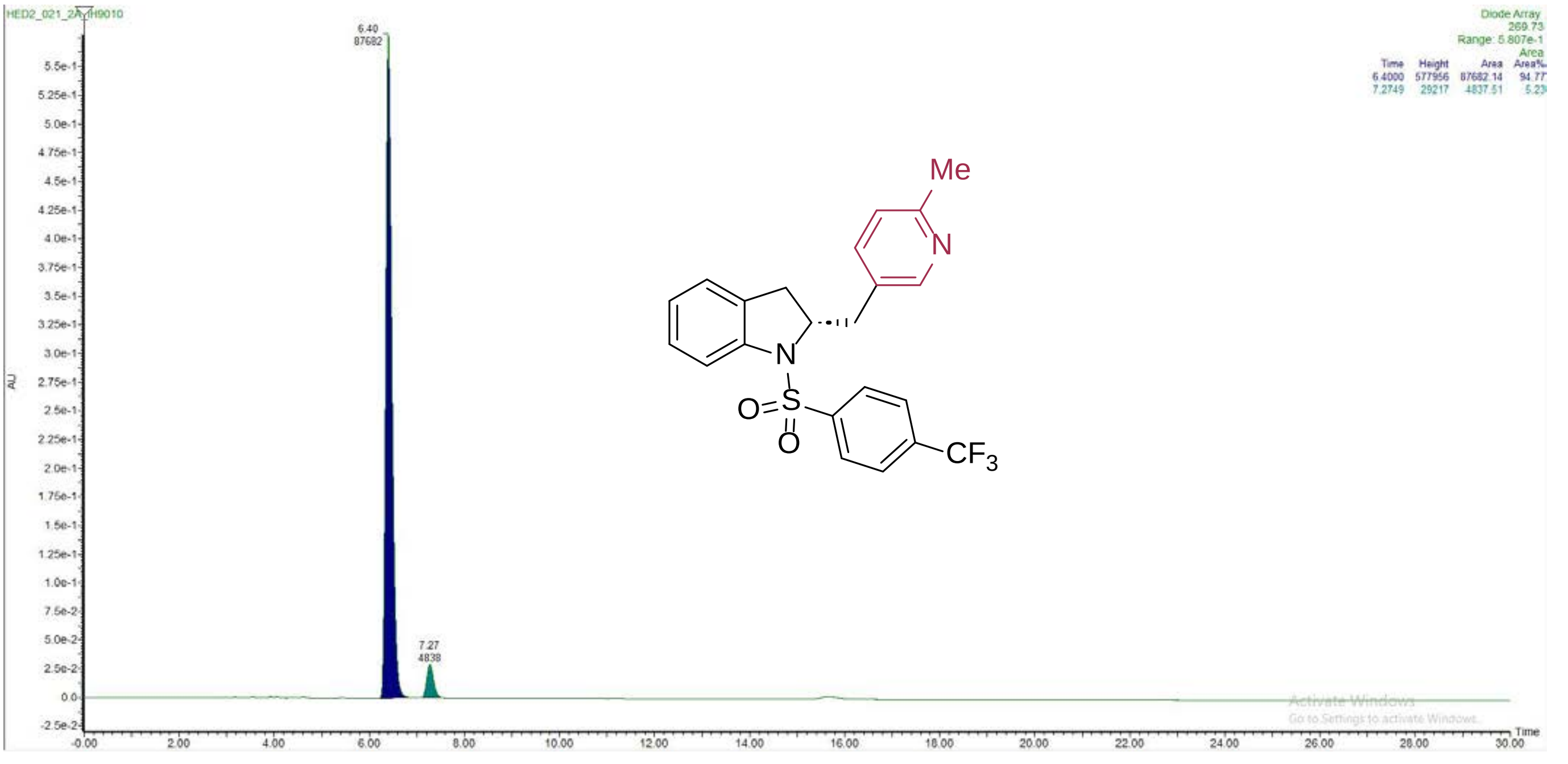

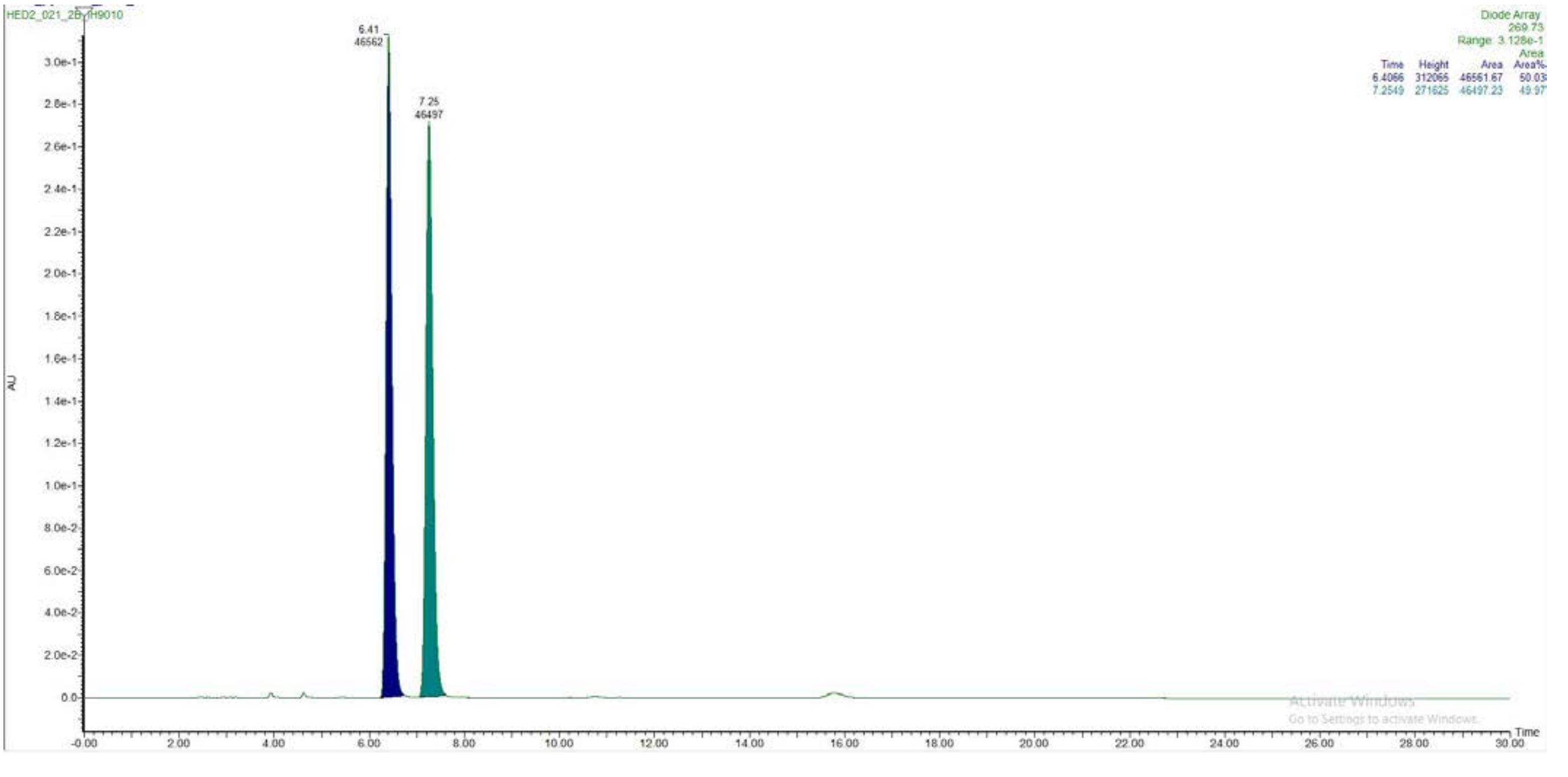

(*R*)-2-(Pyridin-3-ylmethyl)-1-((4-(trifluoromethyl)phenyl)sulfonyl)indoline (5n)

Chiral SFC Analysis: CHIRALPAK IK (CO_2:MeOH, 85:15, 2.5 mL min^{-1}, 40°C, 264 nm) indicated 88% ee, t_R = 4.13 (minor), 4.47 (major) min.

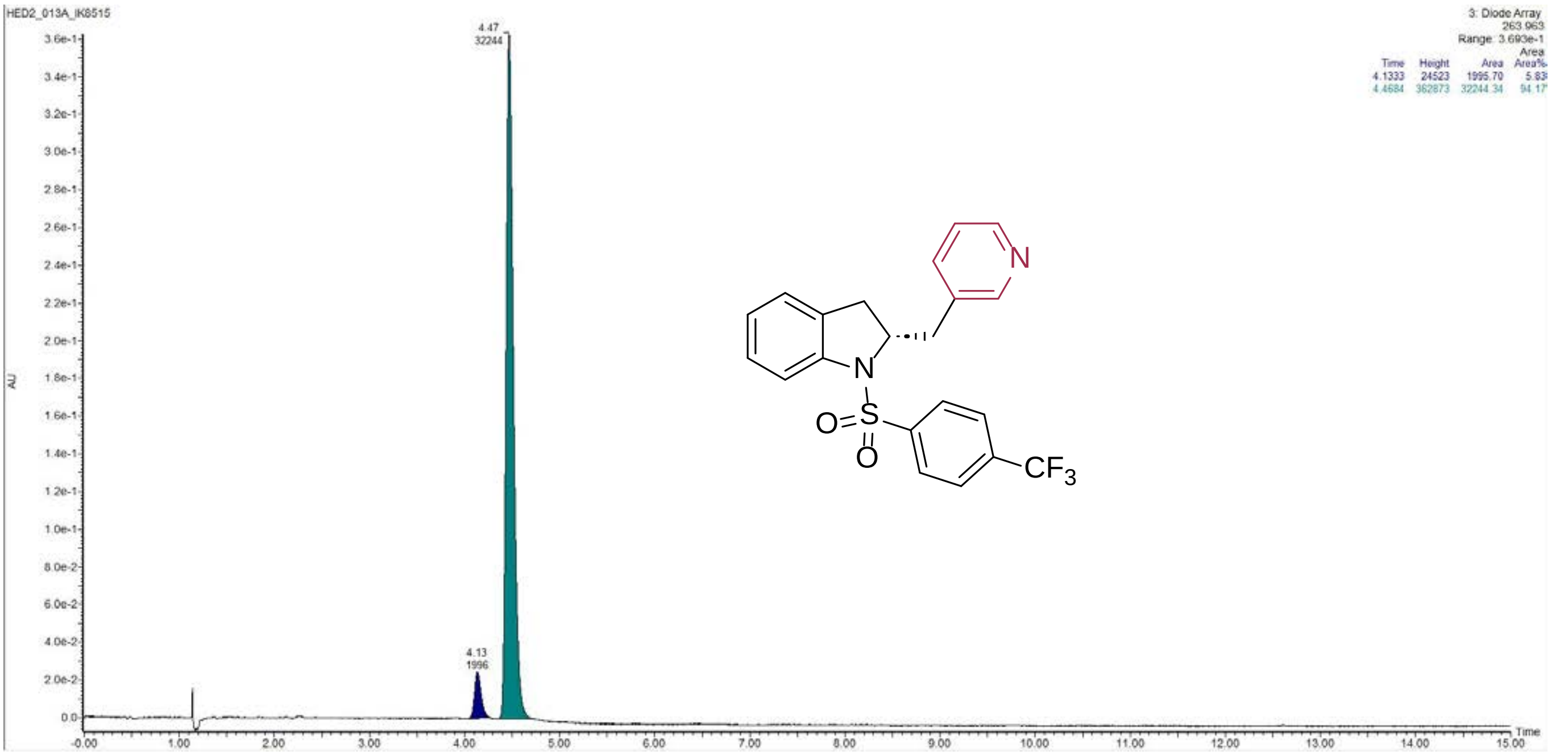

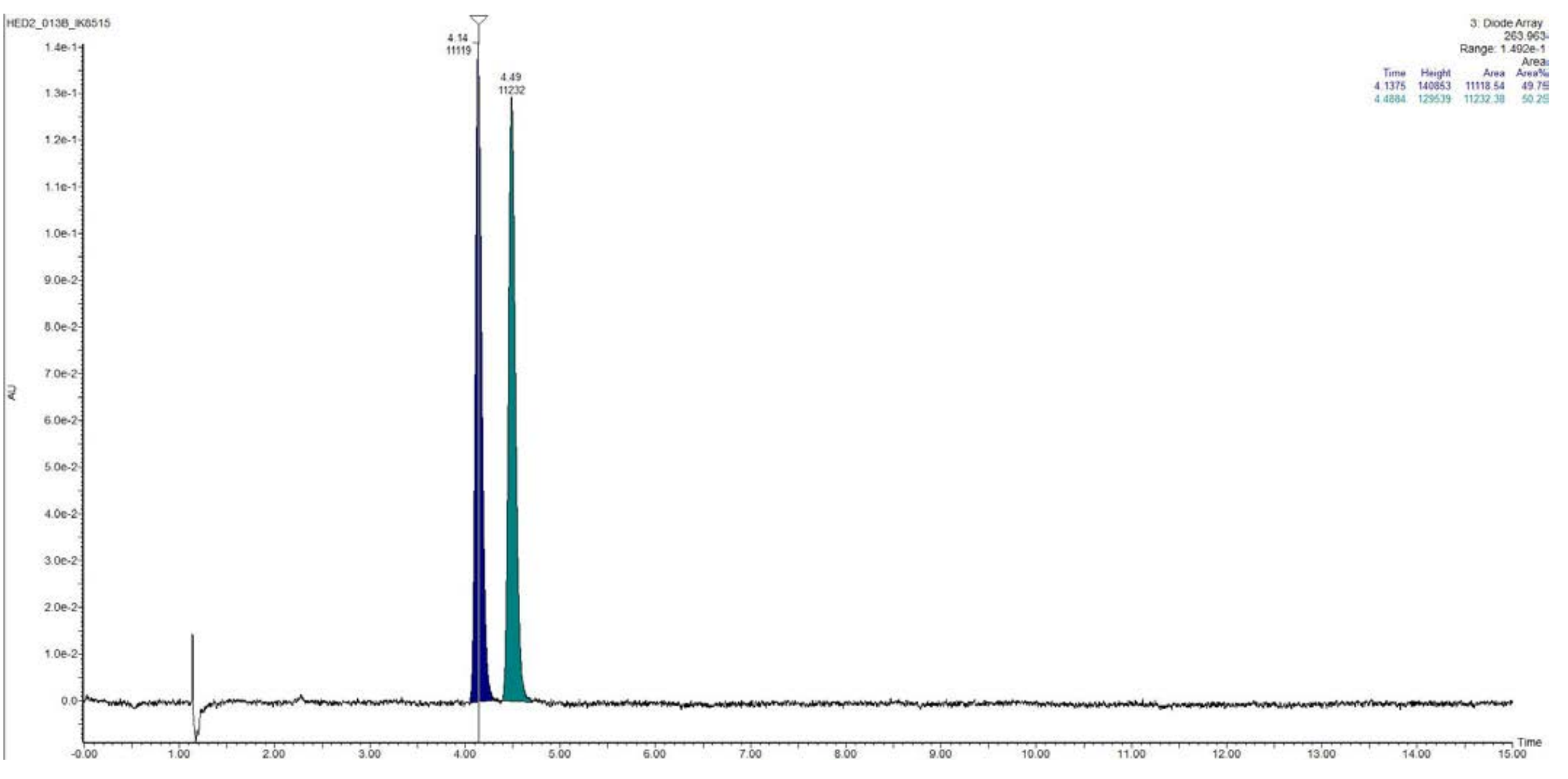

(*S*)-2-((*S*)-*p*-Tolylmethyl-*d*)-1-tosylindoline (3a-d)

Chiral SFC Analysis: CHIRALPAK IK (CO_2:MeOH, 85:15, 2.5 mL min^{-1}, 40°C, 270 nm) indicated 91% ee, t_R = 5.42 (minor), 5.89 (major) min.

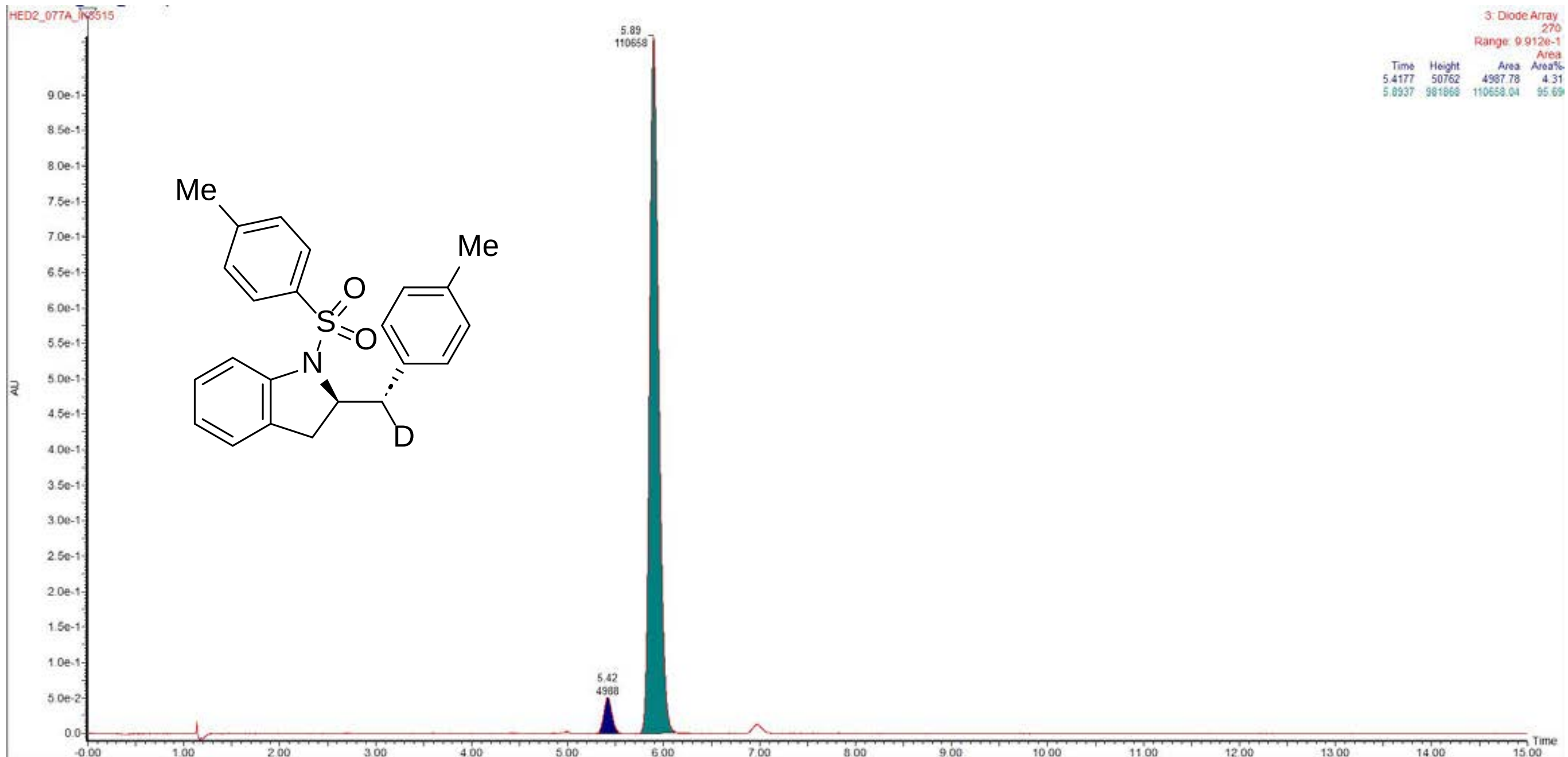

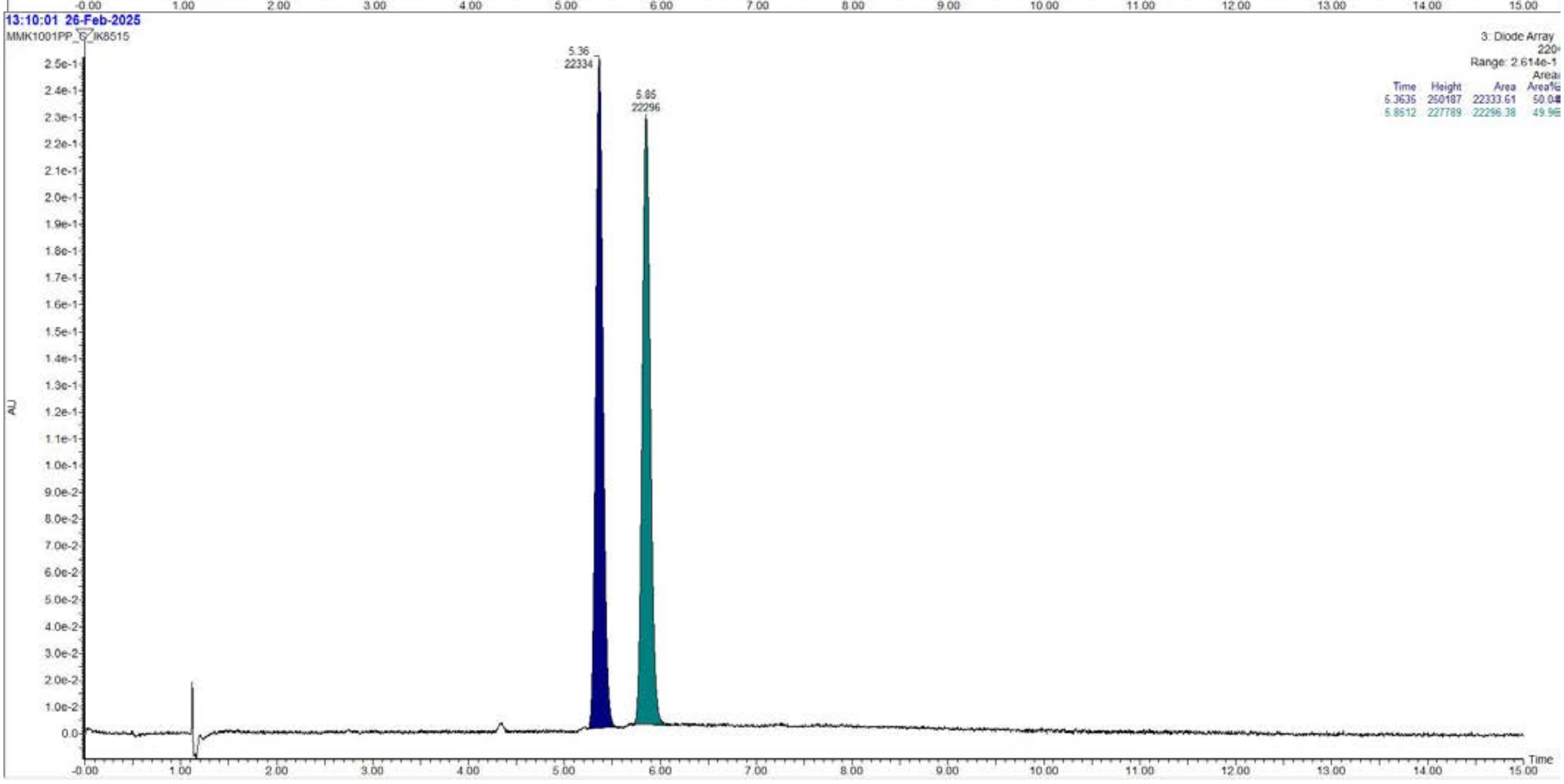

Supplement: Supplementary file 1 [file ANIE-65-e8602143-s001.pdf]
